# Linking activity and function to ecosystem dynamics in a coastal bacterioplankton community

**DOI:** 10.3389/fmicb.2014.00185

**Published:** 2014-04-24

**Authors:** Scott M. Gifford, Shalabh Sharma, Mary Ann Moran

**Affiliations:** Department of Marine Sciences, University of GeorgiaAthens, GA, USA

**Keywords:** bacterioplankton, metatranscriptomics, activity, marine, diel, seasonal

## Abstract

For bacterial communities containing hundreds to thousands of distinct populations, connecting functional processes and environmental dynamics at high taxonomic resolution has remained challenging. Here we use the expression of ribosomal proteins (%RP) as a proxy for *in situ* activity of 200 taxa within 20 metatranscriptomic samples in a coastal ocean time series encompassing both seasonal variability and diel dynamics. %RP patterns grouped the taxa into seven activity clusters with distinct profiles in functional gene expression and correlations with environmental gradients. Clusters 1–3 had their highest potential activity in the winter and fall, and included some of the most active taxa, while Clusters 4–7 had their highest potential activity in the spring and summer. Cluster 1 taxa were characterized by gene expression for motility and complex carbohydrate degradation (dominated by Gammaproteobacteria and Bacteroidetes), and Cluster 2 taxa by transcription of genes for amino acid and aromatic compound metabolism and aerobic anoxygenic phototrophy (Roseobacter). Other activity clusters were enriched in transcripts for proteorhodopsin and methylotrophy (Cluster 4; SAR11 and methylotrophs), photosynthesis and attachment (Clusters 5 and 7; *Synechococcus*, picoeukaryotes, Verucomicrobia, and Planctomycetes), and sulfur oxidation (Cluster 7; Gammaproteobacteria). The seasonal patterns in activity were overlain, and sometimes obscured, by large differences in %RP over shorter day-night timescales. Seventy-eight taxa, many of them heterotrophs, had a higher %RP activity index during the day than night, indicating a strong diel activity rhythm at this coastal site. Emerging from these taxonomically- and time-resolved estimates of *in situ* microbial activity are predictions of specific ecological groupings of microbial taxa in a dynamic coastal environment.

## Introduction

The influence of a bacterial population on ecosystem processes is a function of abundance, metabolic capabilities, and activity rates. Linking these three characteristics at a fine taxonomic resolution in dynamic environments represents a significant challenge for developing a predictive framework for microbial ecology. Much progress has been made in quantifying microbial population abundances and potential function via rRNA genes and metagenomic surveys, but taxonomically-resolved *in situ* measures of activity levels have been more difficult to obtain. Instead, bulk measurements of community production (such as leucine incorporation) or single-gene transcription measures (limited by sequence heterogeneity, incubation steps, or low taxonomic resolution) have been the typical methodologies. Recently, community wide analysis of 16S rRNA:rDNA ratios have provided detailed views of microbial taxa that indicate a decoupling of abundance and activity (Campbell et al., 2011; Hugoni et al., 2013; Hunt et al., 2013). However, this approach is unable to link taxon activity with expressed functional capabilities and cannot account for variations in rDNA copy number or extended ribosome lifetimes (Blazewicz et al., 2013).

Increased sequencing capabilities now allow for genome-wide transcriptional profiles of abundant taxa (defined by similarity binning to the closest sequenced genome) within metatranscriptomic data sets (Gifford et al., 2013; Ottesen et al., 2013). We recently explored the possibility of leveraging ribosomal protein transcription within these reference genome bins as a proxy for *in situ* activity (Gifford et al., 2013). Ribosomal proteins are an essential component of a cell's translation machinery, and their evolutionary conservation makes them valuable for taxonomic identification as well. Although some taxa deviate (Blazewicz et al., 2013), cells generally couple translation to activity, and increases in RP expression have been found to correlate well with increased activity in all three domains of life (Eisen et al., 1998; Wei et al., 2001; Hendrickson et al., 2008). Previous work has shown that the percent of a taxon's transcriptome allotted to RPs provides a relative estimate of activity that agrees well with experimentally-determined growth rates (Gifford et al., 2013).

Here we investigate the potential activity levels and gene expression patterns of bacterioplankton in a dynamic coastal environment over a year-long metatranscriptomic study. The recruitment of transcripts to 200 reference genomes in this time series, encompassing both short term (day-night) and long term (seasonal) variability, uncovered a highly dynamic community with distinct groups of taxa whose activity varied with environmental parameters. The functional genes expressed within these groups revealed metabolic capabilities mapping to the activity patterns.

## Materials and methods

### Sample collection

Sampling occurred at Marsh Landing, Sapelo Island, Georgia, U.S.A. (31°25'4.08 N, 81°17'43.26 W) as part of the Sapelo Island Microbial Observatory program (http://simo.marsci.uga.edu). Samples and environmental measurements were collected quarterly (2008: August 6–7, November 5–7; 2009: February 15–17, May 13–15, August 12–14) with each sampling expedition occurring at four consecutive high tides, resulting in two consecutive pairs of day-night samples per season. Cell collection for RNA extraction was conducted as described previously (Poretsky et al., 2009; Gifford et al., 2011). Briefly, 6–8 L of water was pumped directly from a depth of 1 m and passed through a 3-μm pore-size prefilter (Capsule Pleated Versapor Membrane; Pall Life Sciences, Ann Arbor, MI, USA) and a 0.22-μm pore-size collection filter (Supor polyethersulfone; Pall Life Sciences). The 0.22-μm filter was placed in a WhirlPak bag and flash frozen in liquid nitrogen. Total time from start of filtration to flash freezing was 11–14 min.

#### Sample processing and sequencing

RNA processing is described in Gifford et al. (2011), including the addition of an internal RNA standard to calculate transcript abundances on a per volume basis (Moran et al., 2013; Satinsky et al., 2013). Briefly, 25 ng (4.7 × 10^10^ copies) of the RNA standard constructed from a pGem-3Z plasmid and the frozen filter were added to the bead-lysis solution and RNA was extracted according to RNEasy kit (Qiagen) procedures. Residual DNA was removed using the Turbo DNA-free kit (Applied Biosystems, Austin, TX, USA) and rRNAs reduced first using Epicentre's mRNAOnly isolation kit (Madison, WI, USA) and then with the MICROBExpress and MICROBEnrich kits (Applied Biosystems). The enriched mRNA samples were then linearly amplified using the MessageAmp II-Bacteria kit (Applied Biosystems) and double stranded cDNA synthesized with Promega's Universal RioboClone cDNA synthesis system and random primers. Residual reactants and nucleotides from cDNA synthesis were removed using the QIAquick PCR purification kit. Two samples (FN56 and 57; August 2008) were sequenced by 454 pyrosequencing as described in Gifford et al. (2011), and 4 were sequenced with the Illumina GAIIX platform (described in Gifford et al., 2013). The remaining 16 samples were sheared to ~300 bp with an E210 ultrasonicator (Covaris, Woburn, MA, USA), size selected in the range of 200–400 bp with a Beckman SPRI-TE robot (Beckman Coulter, Brea, CA, USA), and sequenced with Illumina GAIIX to obtain 150 × 150 bp paired end reads. Two samples (FN101B and FN146B) were technical replicates of samples FN101 and FN146, being derived from the same cDNA and Illumina library preparation, but were sequenced in independent lanes. Sequences are deposited in the CAMERA database (http://camera.calit2.net/about-camera/full-datasets) under accessions CAM_PROJ_Sapelo2008, CAM_P_0000917, and CAM-P-0001108. Reads were filtered with a quality score cutoff >20 and a minimum length >100 bp. Overlapping mate pairs were assembled using SHERA (Rodrigue et al., 2010) with a score >0.5. The SHERA assembled reads accounted for 50% of all reads and had a mean assembled length of 180 nt. Non-assembled reads were not considered in the downstream analysis.

### Bioinformatic processing

Reads from all 22 libraries were compared to a custom database containing small and large subunit rRNAs (derived from the SILVA database, www.arb-silva.de, see Gifford et al., 2013) as well as the internal standard sequence using BLASTn. Reads with a bit score >50 to the custom SILVA database were considered rRNA and removed from further analysis. Hits to the internal standard sequence with a score of >50 were tallied and the reads removed from further analysis. The remaining potential protein encoding reads were annotated by a BLASTx homology search against RefSeq version 47, taking the top scoring hit with a bit score >40. Annotated reads were compiled into taxon bins based on the top scoring hit taxon ID from the RefSeq BLAST. The gene content associated with these taxon IDs is derived from isolate genome sequencing projects (metagenomic assemblies are not included) and can be in various states of completion (i.e., draft vs. complete). KEGG orthology (KO) and pathway information for the annotated reads was retrieved from the integrated microbial genomes database (IMG; img.jgi.doe.gov/cgi-bin/w/main.cgi). Proteorhodopsin genes within the top 200 transcript recruiting genomes were identified by a BLASTp homology search using three proteorhodopsin (PR) query sequences (NCBI accessions: 254455918, 118594191, 225010551).

#### RP composition

Ribosomal protein reads (RPs) were initially identified by a text based query for “ribosomal protein” in the RefSeq annotation. To confirm the annotation, the RP reads binning to the top 200 transcript recruiting taxa were compared to the KEGG database in CAMERA via BLASTx. The 1.1 million RPs binned to 9296 different genes, of which 96% fell into KO pathway “ribosome” (ko03010) and 2% hit RP modification KOs. The remaining 2% of reads without an RP KO annotation accounted for <5% of any given taxonomic bin's total RP hits and were kept in the downstream analysis.

For the top 200 transcript-recruiting taxonomic bins, %RP was calculated as the sum of all RP annotated reads in a bin divided by the total number of reads in the bin. Significant differences in %RP abundance were determined by bootstrapping. For day-night differences, the observed mean difference in %RP was calculated for the nine day-night pairs [the two 454 samples had no corresponding day sample and were excluded; samples with a technical replicate (FN101A/B and FN146A/B) were averaged]. The 18 samples were then randomly assigned to a pair and the random mean difference calculated for 10,000 iterations. The *p*-value was the number of random observations greater than or equal to the observed mean value, with *p*-values <0.05 considered significant. The same procedure was used to test for significant differences between day and night absolute RP per L obtained by internal standard normalization (Satinsky et al., 2013). Statistically significant seasonal differences in %RP were obtained by calculating an observed sum of squares difference between the five seasonal groupings (summer1, fall, winter, spring, summer2), and then randomly assigning samples to the seasonal groupings and calculating *p*-value as the number of random sum of squares > to the observed sum of squares.

#### Clustering

The top 200 transcript-recruiting taxonomic bins were clustered by calculating the %RP pairwise Pearson's correlation coefficients among all the bins, converting the coefficients to a distance matrix ([1- corr. coeff.] /2), and clustering with the hclust function in R using the default settings and complete linkage. To compare patterns in %RP and environmental parameters measured during the SIMO sampling, we conducted a canonical correspondence analysis (CCA) using the cca function in the Vegan R package (Oksanen et al., 2013) with %RP as the species abundance metric and the samples as sites. An overall model permutation test using the ANOVA function in R rejected the null hypothesis that %RP abundance and measured environmental gradients were not related (*p* < 0.01 for 1000 iterations).

#### Indicator analysis

Enrichment of a KEGG KO ortholog for a given RP cluster was determined using the indicator species analysis of Dufrêne and Legendre (1997; also see Gifford et al., 2013). For individual KEGG KOs, an indicator value (IV) was calculated for each activity cluster based on both fidelity (the proportion of cluster member samples in which the KO was expressed) and specificity (the proportion that an RP cluster contributed to the total summed KO expression across all clusters). The IV was calculated as follows: A hit was defined as a gene binning to a given KO being expressed in one of the 20 samples [i.e., expression detected (1) or not detected (0)]. The number of hits per taxon for a given KO exceeded 20 in some cases, as some taxa had multiple genes binning to the same KO. The hit count was then normalized by summing all the hits to a KO within an activity cluster and dividing by the number of taxa in that cluster. Specificity was calculated for each activity cluster as the normalized hit count divided by the sum of all the RP cluster normalized hit counts. Fidelity was the total number of samples that had the given KO expressed in an activity cluster divided by the total samples (number of taxa times 20 samples). The indicator value was calculated as the product of fidelity and specificity multiplied by 100.

Statistically significant differences in indicator values between activity clusters were determined by random permutations. Taxa were randomly assigned to an activity cluster (keeping the number of taxa in a cluster the same) and the IV of the random clusters was determined. The process was repeated for 1000 iterations and the *p*-values were calculated as the proportion of random IVs greater than observed IVs.

To identify diel differences in Cluster 2 expression, the indicator analysis was conducted as above except only Cluster 2 KOs were considered and the orthologs were grouped into two sets, those from night samples and those from day samples. Significantly different indicator values between the day and night groups were determined through the random permutation test described above (*p* < 0.05, 10,000 iterations).

## Results

Samples were collected off the coast of Georgia, USA as part of the Sapelo Island Microbial Observatory quarterly sampling program (http://simo.marsci.uga.edu). Samples for RNA, DNA, and other environmental parameters were collected in triplicate over four high tide cycles within 48 h, resulting in samples from two consecutive days and nights (Table S1). Twenty RNA samples encompassing a period from August 2008 to August 2009 were sequenced, representative of the summer, fall, winter, spring, and second summer seasons. Six libraries were previously reported (Table S2): two using 454 pyrosequencing (Gifford et al., 2011) and four using the Illumina GAIIx platform with 100 bp single reads (Gifford et al., 2013). The remaining 14 samples were sequenced using the Illumina GAIIx platform with 150 × 150 paired ends (Table S2; overlapping paired reads were assembled), with two of the samples sequenced in duplicate (i.e., technical replicates). The combined 253 million reads from all 22 libraries were compared to a custom database of SILVA rRNAs (Gifford et al., 2013) using BLASTn to identify and remove any residual rRNAs. The remaining 52 million potential protein encoding reads were compared to NCBI's RefSeq database (version 47) using BLASTx. The 29 million reads that had a significant hit (bit score > 40) fell into 5600 reference genome bins, with the top 200 bins accounting for two-thirds of all annotated reads. Over 1.4 million reads were annotated as RPs, comprising 5% of all RefSeq hits. The contribution of RP transcripts to a reference genome's transcriptome (%RP) was used as a proxy for relative activity.

### Relative activity patterns

**Figure 1:**
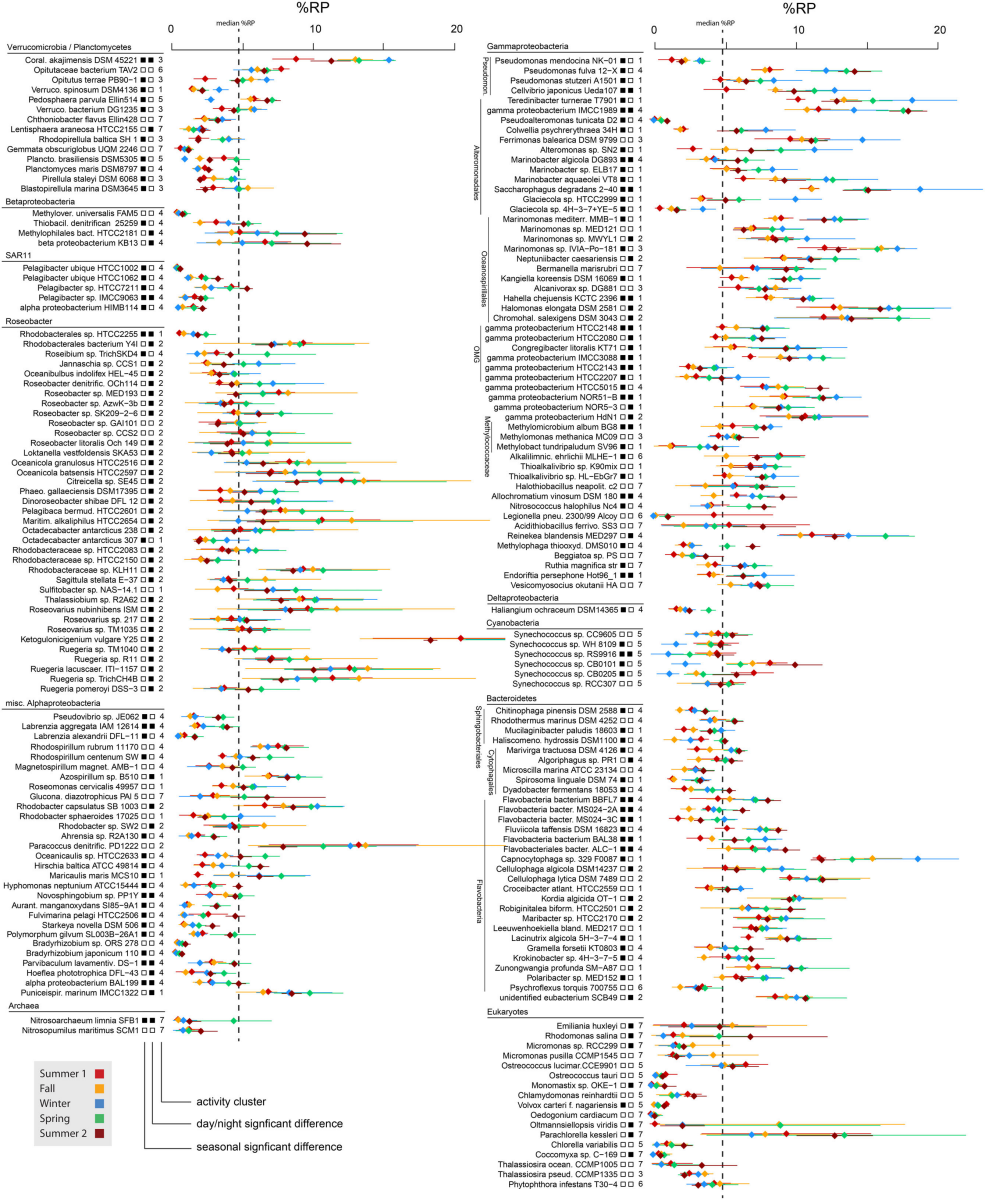
**Seasonal variation in %RP values for the top 200 transcript-recruiting genome bins**. Error bars indicate the 95% confidence intervals determined by bootstrapping (1000 iterations). The activity cluster of each bin is given (see Figure 2 and Figure S1), and bins with significant variation in %RP either between seasons or between day and night samples are indicated.

An examination of the top 200 transcript-recruiting bins (including all 3 domains of life) revealed high diversity in potential activity levels across community members (Figure 1), though often both the magnitude and variance in %RP were conserved among closely related taxa and tended to diverge with increased phylogenetic distance. Variance in activity across the seasons (summer1, winter, fall, spring, and summer2) increased with the magnitude of %RP, with a significant linear relationship between mean %RP and standard deviation (*p* < 0.0001, *R*^2^ = 0.46). Thus the slow-growing taxa tended to maintain their low activity indexes both within and across seasons (Figure 1); these included all SAR11 genomes and several other alphaproteobacterial groups (Rhodospirillales, Rhizobiales, and non-Roseobacter Rhodobacterales), as well as the two archaeal reference bins. In contrast, high %RP dynamics was particularly noticeable for Roseobacters, with mid to upper activity indexes yet high temporal variability. Bacteriodetes fell into two activity groups, with slightly below average indexes for the Cytophagales/Sphingobacteria, and distinctly higher indexes for Flavobacteriales; both groups had relatively low across-season variability. The taxon bins with the highest activity indexes (>10 %RP) were most often Gammaproteobacteria bins (particularly members of the Alteromonadales and Oceanspirillaceae), but also included populations binning to the verrucomicrobium *Coraliomargarita akajimensis*, the roseobacters *Citreicella* and *Ketogulonicigenium*, and the alphaproteobcterium *Paracoccus denitrificans*.

**Figure 2:**
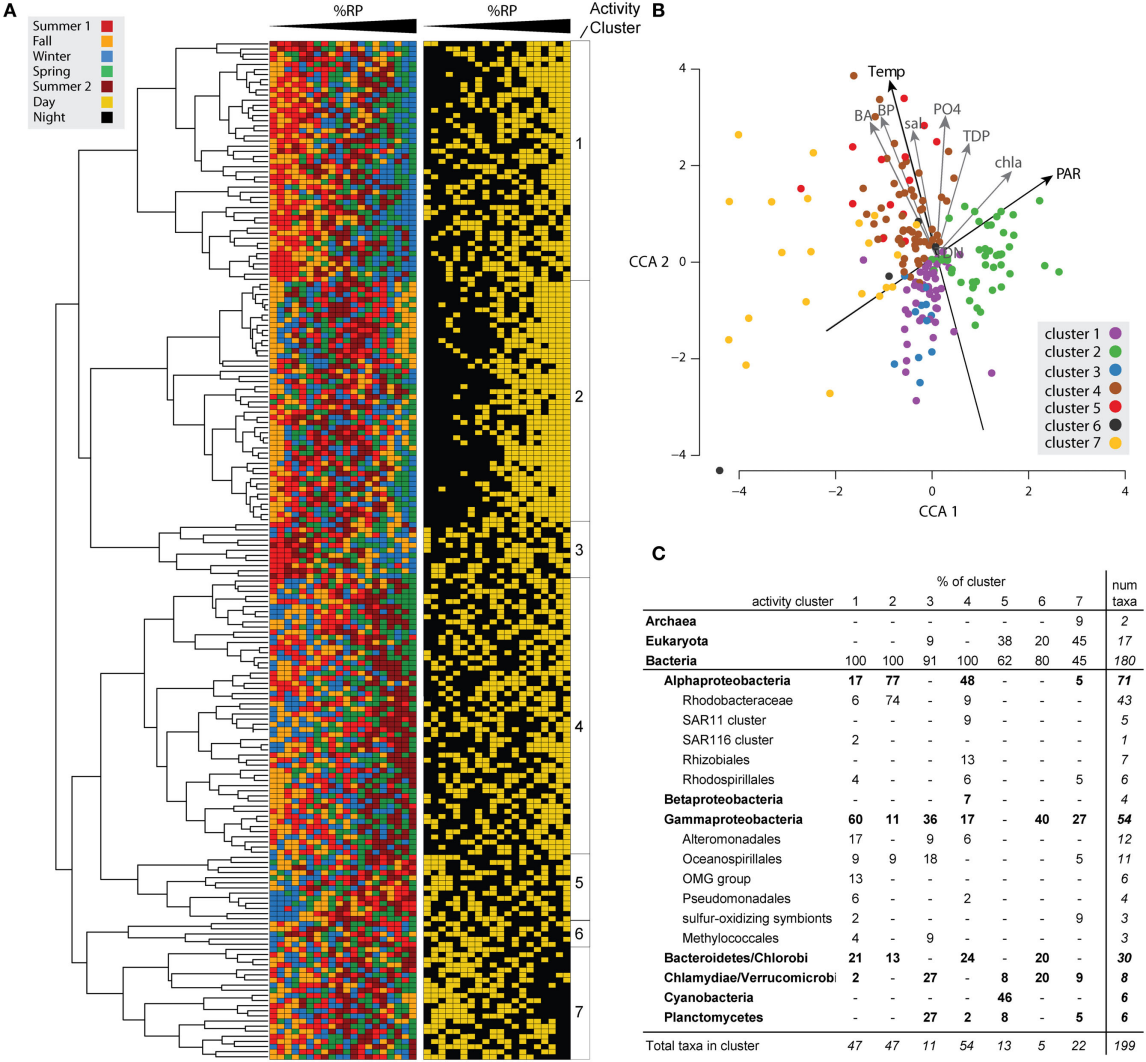
**Patterns in %RP expression among genome bins and in relation to environmental variables. (A)** The top 200 transcript-recruiting taxa were hierarchically clustered based on pairwise Pearson correlations of %RP. To the right of the dendrogram, the 20 samples are arranged in rank order from lowest to highest %RP and colored by the sample's seasonal and day-night origins. See Figure S1 for the same dendrogram with taxon labels included. **(B)** Canonical Correspondence Analysis (CCA) of the 200 taxa ordinated by %RP and environmental variables. The taxa are colored according to their activity cluster as shown in part **(A)**. **(C)** Taxonomic composition of cluster members.

These patterns in %RP are suggestive of distinctive life-histories among coastal community members. Two caveats for interpreting ribosome-related signals, however, are that a significant portion of protein synthesis activity may be related to non-growth functions (Blazewicz et al., 2013), and relationships between ribosome content and activity may be taxon-specific (Lin et al., 2013). We therefore confined our exploration of %RP patterns to examinations of within-taxon temporal patterns, focusing on the 200 highest-recruiting reference genome bins and using hierarchical clustering to group taxa with similar temporal activity patterns across the 20 time points. A bifurcating dendrogram that further resolved into seven deeply branching clusters (hereafter referred to as activity clusters) with distinct temporal patterns in activity and taxonomic membership emerged from this analysis (Figure 2A; Figure S1).

### Linking activity, function, and environmental dynamics

A CCA relating potential activity to temporal gradients in environmental conditions produced similar groupings for the taxa as the hierarchical clustering method and indicated that members of the same activity cluster had similar activity optima along the measured environmental gradients (Figure 2B). Temperature and PAR explained the most variation in taxon activity. The CCA plot also indicated that the major bifurcation evident in the cluster dendrogram (Figure 2A) was related to factors that correlate with temperature, as taxa in Clusters 1–3 fell below the temperature centroid and taxa in Clusters 4–7 fell above it (Figure 2B).

We examined expression of functional genes in the 200 reference genomes to determine if patterns in function further united the activity clusters. Functional characterization was approached in three ways. (1) *Indicator gene expression*: The orthologous gene relationships among 168 of the bacterial genome bins were defined based on their KEGG KO assignments in the integrated microbial genomes (IMG) database (ver 3.5) and used in an indicator species analysis (see Methods) to identify expressed genes characteristic of each cluster (using a random permutation test with 1000 iterations to determine significance; Table S3). These 168 genomes represent 90% of the 188 bacterial members within the top 200 transcript recruiting taxa. The 20 genomes not included in this analysis were not available in IMG. (2) *KEGG pathway enrichment*: Significant indicator genes identified in approach #1 were assigned to KEGG pathways based on their KO assignment, and pathways with significantly higher representation in a cluster were identified by permutation tests (Table S4). (3) 4.1.1. *Highly expressed RefSeq genes*: The most highly expressed RefSeq-annotated genes within individual genome bins were investigated for seasonal and diel dynamics.

#### Activity cluster 1

**Figure 3:**
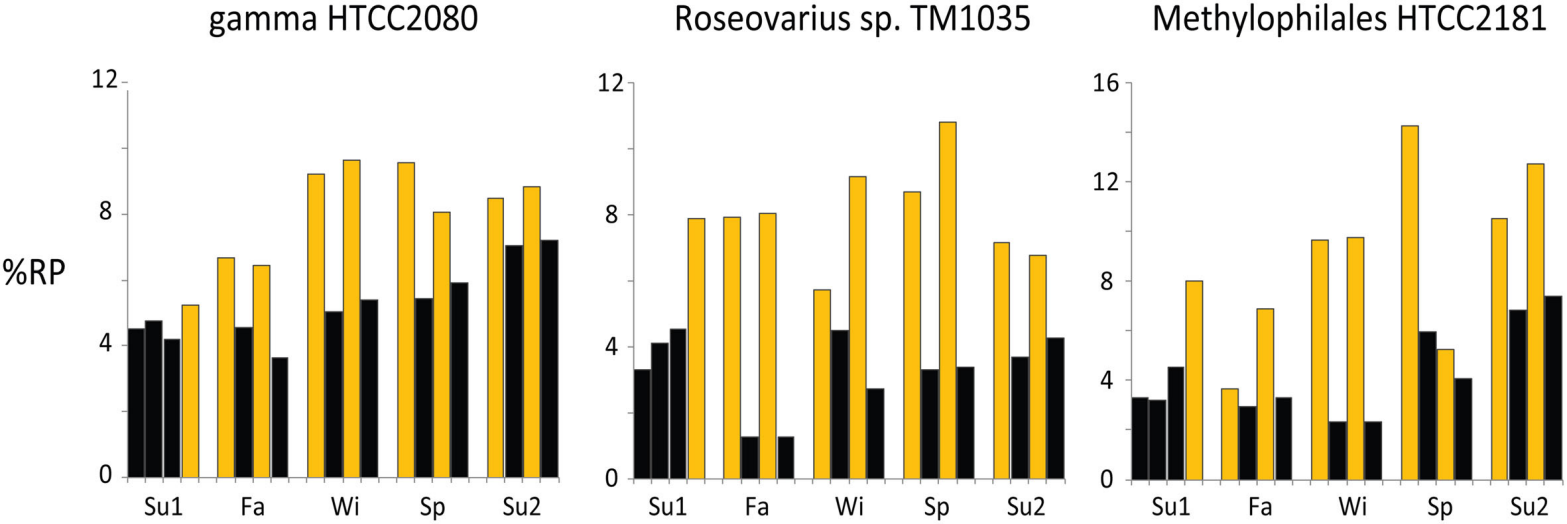
**Daytime enrichment in the transcriptome devoted to ribosomal protein synthesis**. Example bins are from Activity Cluster 1 (gamma proteobacterium HTCC2080), Cluster 2 (Roseobacter *Roseovarius* sp. TM1035), and Cluster 4 (betaproteobacterium Methylophilales HTCC2181). Black bars = night samples, gold bars = day samples. For the %RP graphs of all top 200 transcript recruiting taxa see Figure S2.

Cluster 1 members generally had highest potential activity in the winter and spring samples (Figures 2A,B), with 72% of the 47 members having a significant seasonal activity pattern. Consistent with this cold-water bias, several psychrotolerant reference genome bins were in this group (*Glaciecola* spp*.*, *Colwellia psychrerythraea*, *Polaribacter* sp*.*, and *Octadecabacter antarcticus*; Figure 1). Diel differences in activity were also apparent, with a third of members having significantly higher %RP in the day than night (Figure 3 and Table S5). The cluster was dominated taxonomically by Gammaproteobacteria (related to *Alteromonadales*, *Oceanospirillales*, OMG, and NOR5 groups) with additional members from the Alphaproteobacteria (Roseobacter, SAR116) and Flavobacteria (Figure 2C).

The indicator analysis revealed that Cluster 1 was significantly enriched in gene expression for flagellar biosynthesis, type IV pilus assembly, chemotaxis, secretion systems, and sodium driven transport. Biopolymer transport was also characteristic, with 90% of cluster members expressing TonB dependent transporters. Highly expressed RefSeq genes within these bins included cadherins, extracellular binding proteins, and glycosyl hydrolases, suggesting involvement by members of this cluster in attachment to and degradation of complex carbohydrates. Known polysaccharide degraders among the reference genome bins included *Teredinibacter turnerae*, *Saccharophagus degradans*, and *Zunongwangia profunda.* Cluster 1 was significantly enriched in taxa with PR genes (*p* < 0.05, permutation test, 10,000 iterations), having 13 of the 27 PR-harboring taxa in the top 200 transcript-recruiting bins. PR was the first or second most highly expressed gene for the vast majority of these taxa.

#### Activity cluster 2

Cluster 2 was distinguished by strong day-night differences in activity levels, with almost all high %RP samples collected in the day (Figures 2A, 3) and in association with high PAR levels (Figure 2B). The cluster is dominated by Roseobacters, with 33 of the 37 Roseobacters in the top 200 taxa assigned to Cluster 2 (Figure 2C). The large differences in day-night activity for this cluster is consistent with the high Roseobacter %RP variability seen in Figure 1, as both the highest (day-time) and the lowest (nighttime) activity occur in the fall for these taxa (Figure 3), with substantial day-night divergences also seen in other seasons.

KEGG pathways significantly enriched in Cluster 2 indicator genes (Table S4) included several amino acid (AA) metabolism pathways (144 AA significant indicator genes; three times higher for this cluster than any other; Table S4 and Figure S3). Cluster 2 contained more than half of all transporter indicator genes (Table S4), many of which were ABC transporters for amino acids. Indicator genes for aromatic compound degradation were also characteristic of Cluster 2, including those for aromatic amino acids as well as for a broader array of aromatic substrates such as benzoate (Table S4 and Figure S3). Most striking was a set of 14 indicator genes making up a complete degradation pathway that started with the aromatic compounds salicylate, anthranilate, and vanillate and led into the TCA cycle (Figure S3B). A second aromatic pathway for the degradation of phenylacetic acid was also present, composed of 10 indicator genes (Figure S3B).

Cluster 2 contained half of all the aerobic anoxygenic photosynthetic (AAnP) genomes in the top 200 reference genomes, and three AAnP-related genes (*pufM, pufL*, and a light harvesting protein) were indicators. Other Cluster 2 indicator genes included formate dehydrogenase, DMSO reductase, mercuric reducatase, phosphonate metabolism (*phnGHIJM*), carbon monoxide dehydrogenase, and urease (Table S3). Finally, carboxylic acid metabolism was characteristic of the cluster, which was significantly enriched in KEGG pathways for glyoxylate/dicarboxylate, propanoate, and butanoate metabolism (Table S4).

#### Activity cluster 3

Cluster 3 did not exhibit the strong day-night differences in activity observed for Clusters 1 and 2, but did show strong seasonal differences (Figure 2A) in which potential activity was inversely correlated to water temperature (increasing from summer to winter; Figure 2B). Taxonomically, Cluster 3 was primarily composed of relatives of Verrucomicrobia, Planctomycetes, and Gammaproteobacteria. Functionally, Cluster 3 expression was enriched for indicator genes in KEGG pathways for sugar metabolism (fructose/mannose; pentose/glucuronate interconversions; Table S4). Overrepresentation of glycan metabolism genes and sulfatase genes may indicate roles in the breakdown of polysaccharides (Teeling et al., 2012). Like Cluster 1, Cluster 3 also had indicator genes for motility, chemotaxis, and secretion systems.

#### Activity cluster 4

Cluster 4 was characterized by higher potential activity in the spring and second summer, corresponding with warmer waters (Figure 2B). Like Cluster 1, 80% of members had significant seasonal activity patterns, although this cluster was warm-water biased rather than cold-water biased. Day-night patterns in activity were not consistent across the cluster, with only one-third of members having significant %RP day-night differences (Table S5). Cluster 4 was the largest cluster, containing 54 taxa representing diverse lineages (Figure 2C). Possibly due to this diversity, there were few significant indicator genes that united the cluster, and no KEGG pathways were significantly enriched. The cluster was significantly enriched in PR containing taxa (*p* value < 0.05, permutation test, 10,000 iterations), harboring 12 of the 27 PR taxa in the top 200 taxa.

Within Cluster 4, however, several subclusters had distinct taxonomic and functional gene expression characteristics (Figure S1), one of which contained four of the five SAR11 genomes. The populations recruiting to the SAR11 bins had significantly higher %RP in the summer and spring samples compared to other seasons. Several of the most highly expressed genes within SAR11 bins also had seasonal dynamics (sodium symporter, V-type pyrophosphatases, elongation factors; Figure S4A). The SAR11 PRs showed little temporal variation, fitting with previous observations that this gene is often constitutively expressed by SAR11 members (Figure S4A) (Steindler et al., 2011; Vila-Costa et al., 2013).

Adjacent to the SAR11s was another subcluster containing proteobacteria with a distinct methylotrophy signal (Figure S1). Gene expression of subcluster members Betaproteobacteria KB13 and HTCC2181 was dominated by methanol dehydrogenase (up to 50% of hits; Figure S4B). Although these taxa showed some of the highest day-night variation in potential activity over the entire time series (Figure 3), there was no overall significant day-night difference in expression of methanol dehydrogenase (*t*-test, *p > 0.1*); however, winter expression of this gene was considerably higher at night (Figure S4). The methylotrophy subcluster also included the alphaproteobacterium *Bradyrhizobium* sp. reference genome bin, with a putative ethanol dehydrogenase and methanol dehydrogenase among its most highly expressed genes, as well as populations binning to the gammaproteobacterium *Methylophaga thiooxydans*, also with a methanol/ethanol dehydrogenase as a highly-expressed gene.

#### Activity cluster 5

Strong seasonal variation characterized Cluster 5 members' activity (low in winter, high in both summers; Figure 2A). The cluster was dominated by phytoplankton taxa, including the picoeukaryote reference genomes *Ostreococcus*, *Chlamydomonas*, and *Chlorella* and all six *Synechococcus* genomes (Figure 2C). The *Synechococcus* genomes grouped tightly and showed significant positive correlations between %RP and temperature (Pearson's correlation, *p* < 0.05) (Figure S5). Cluster 5 had many photosynthesis-related indicator genes and significant enrichment of KEGG pathways for glucan, retinol, porphyrin, chlorophyll, and photosystem biosynthesis (Table S4). Interestingly, only one of the cyanobacteria reference bins in this cluster (*Synechococcus* sp. RS9916) had significant day-night differences in %RP, fitting previous observations that *Synechococcus* diel periodicity of growth- and photosystem-related transcription can be muted compared to other phytoplankton taxa (Ottesen et al., 2013). In addition to the photoautotrophs, this cluster included two heterotrophic reference genome bins, *Planctomyces brasiliensis* and verrucomicrobium *Pedosphaera parvula*.

#### Activity cluster 6

Only 5 taxa grouped into Cluster 6, including Gammaproteobacteria, Bacteriodetes, and Verrucomicrobia. This cluster had few defining characteristics except for the general absence of significant day-night or seasonal differences in activity. There were few indicator genes and no significantly enriched pathways.

#### Activity cluster 7

Cluster 7 members were diverse in the seasonal timing of their peak activity, but were distinguished by significantly higher activity in the night vs. the day (Figure 2A and Table S5). The CCA analysis shows Cluster 7 members widely spread along the temperature gradient, but all plotting below the median PAR (Figure 2B). The cluster was taxonomically diverse, including eukaryotic phytoplankton, Archaea, and several heterotrophic bacteria. The eukaryotes' functional gene expression was dominated by photosynthesis machinery, with photosystem II transcripts accounting for two thirds of these bins and significantly enriched in the day (*t*-test, *p* < 0.05), a pattern similarly observed in metatranscriptomic studies of coastal Pacific phytoplankton (Ottesen et al., 2013). The two archaeal reference genomes had relatively stable gene expression across seasons, despite the fact that their populations bloom in the late summer at this site (Hollibaugh et al., 2011, 2014). The heterotrophic bacterioplankton were enriched in sulfur oxidizing Gammaproteobacteria reference genome bins (*Beggiatoa* sp., *Ruthia magnifica*, *Vesicomyosocius okutanii*, *Halothiobacillus neapolitanus*) with high expression of sulfur oxidation genes, adenylylsulfate reductase, rhodanese, and cytochromes.

## Discussion

### Seasonal succession of activity

**Figure 4:**
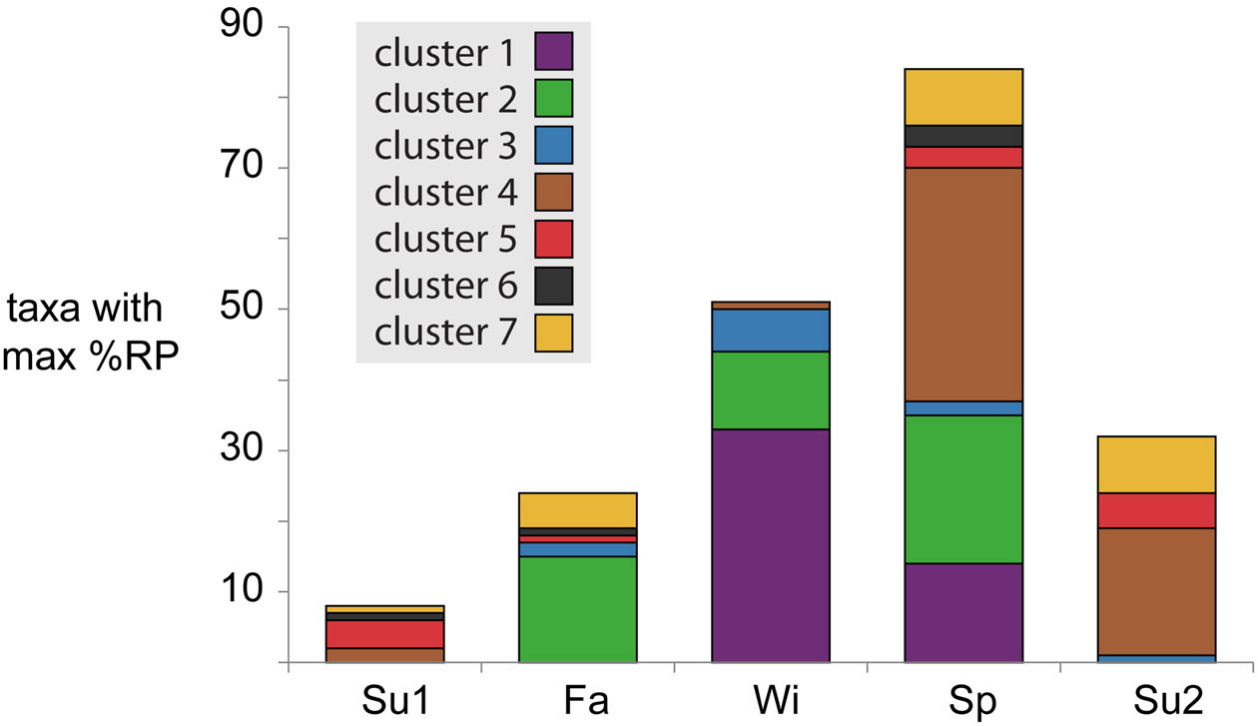
**Number of taxa with maximum %RP occurring in each season**. Bars are colored based on activity cluster assignments.

Temperature shifts reflect the broader seasonal changes in environmental conditions at this site, correlating with the deepest divergence in microbial activity patterns that split taxa into one group with higher potential activity in cold weather (Clusters 1–3) and one group with higher activity in warm weather (Clusters 4–7) (Figure 2). In the spring, as water temperatures warmed from winter lows, the system entered its period of maximum primary production, coinciding with increased activity of phytoplankton in Clusters 5 and 7 (Figure 2). High primary production likely resulted in increased concentrations of labile DOM, driving the greater numbers and phylogenetic diversity of bacterial taxa with maximum potential activity during the spring (Figure 4).

Water temperatures reach their peak in summer, a period characterized by high inorganic nutrient availability, respiration rates, and bacterial abundance (Figure 2, Table S1; Hollibaugh et al., 2014). Both primary and bacterial production are high during this period, suggesting rapid cycling of matter and energy through the microbial loop. Taxa peaking in activity during summers were found almost exclusively in Clusters 4, 5, and 7 (Figure 4), including SAR11s, methylotrophs, and Archaea with gene expression emphasizing the metabolism of low molecular weight organic compounds (methanol, acetate) and transport of inorganic nutrients (ammonia). The gene expression patterns are consistent with a streamlined life-style (Giovannoni et al., 2005), potentially allowing these taxa to more efficiently compete for labile organic matter being released directly from primary producers or resulting from microbial recycling. A comparison of the two summer seasons revealed that summer2 had higher bacterial production rates and cell concentrations and lower nutrient levels (Table S1), suggesting the higher percentage of taxa with greater %RP compared to summer1 were responding to a more productive environment.

Environmental conditions during the fall season reflected a transition period, with decreases from summer highs in water temperature, primary production, nutrient availability, and microbial production. We observed many taxa that had both their highest and lowest %RP occurring in one of the fall samples (Figures 2A, 4). Cluster 2, in particular, was dominated by taxa with high fall activity, with characteristic gene expression for metabolism of amino acids and aromatic compounds (salicylate, vanillate, phenylacetic acid, and anthranilate), reflecting utilization of both labile and refractory substrates that might be linked to the initiation of vascular plants senescence in adjacent marshes.

The system was at its most heterotrophic during the winter, with low water temperatures, nutrient availability, and primary production (Figure 2B, Table S1). Surprisingly, many bacteria had their maximum activity index in the winter samples, particularly those in Clusters 1–3, potentially driven by an ability to processes more refractory substrates or complex carbohydrates during a period when labile compounds released by primary producers maybe in short supply. Expression of TonB dependent transporters, cadherins, extracellular binding proteins, and glycosyl hydrolases characterized these winter-active taxa. Motility and chemotaxis were also characteristic of these groups, suggesting reliance on particles or transient patches of enriched organic matter.

### Diel patterns of activity

Eighty-seven genome bins had significantly different activity indexes in the day than night, many of which were found in Clusters 1, 2, 4, and 7 (35, 92, 30, and 41% of members, respectively). Indications of diel forcing of microbial transcription has been found previously in the Western English Channel (Gilbert et al., 2010), North Subtropical Pacific (Poretsky et al., 2009), and Monterey Bay (Ottesen et al., 2013). Here, we found that in addition to the diel dynamics of photoautotrophs, a significant fraction of the heterotrophic community also exhibited day-night activity dynamics, with day-time %RP enrichment for 78 heterotrophic bacterial genome bins (Table S5 and Figure 3).

**Figure 5:**
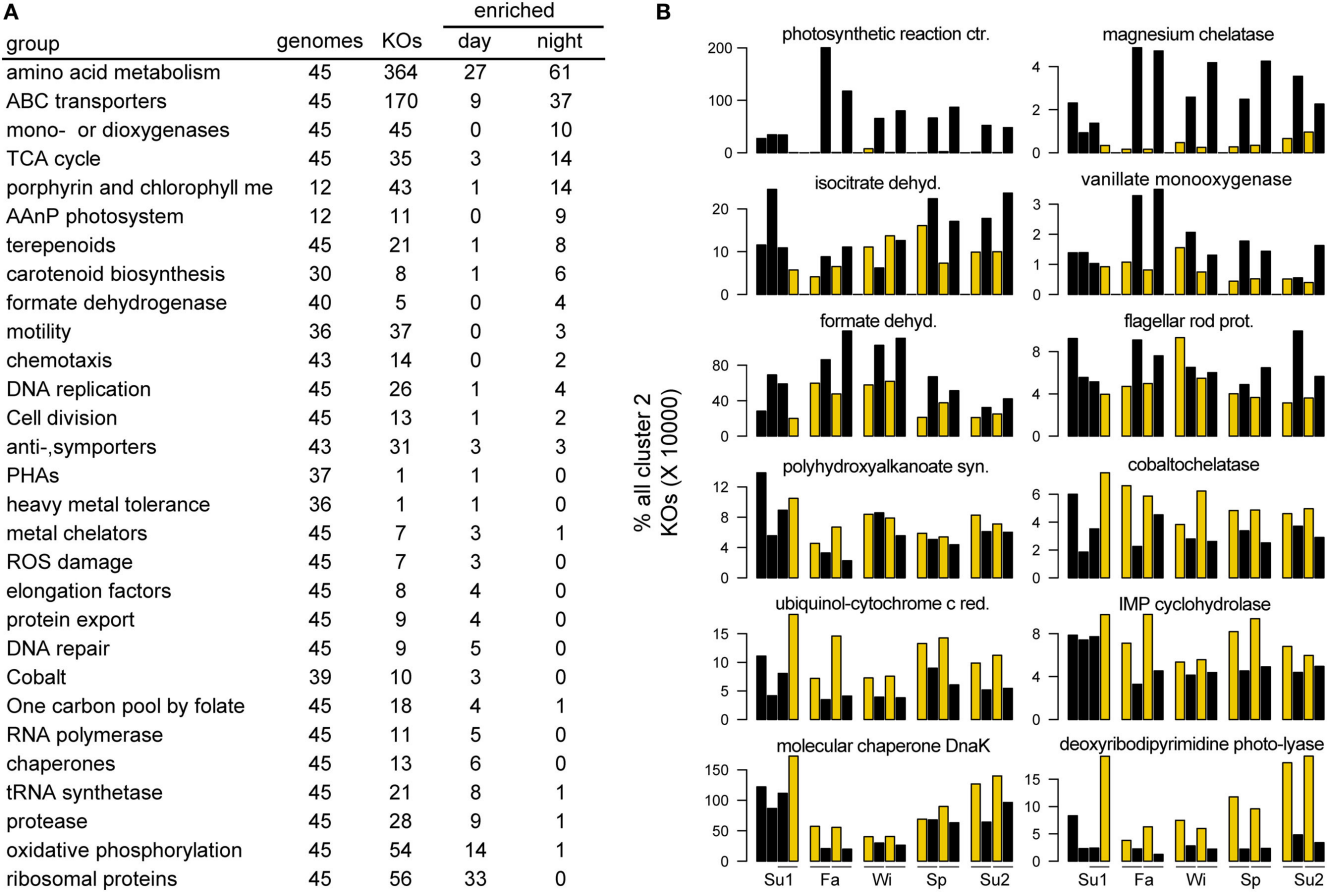
**Cluster 2 day and night indicator genes. (A)** Orthologs (KEGG KOs) that were indicators for day or night samples. “genomes” indicates the number of Cluster 2 genomes expressing orthologs in the given category (out of 45 total genomes in the analysis). “KOs” indicates the number of unique KO classifications in that category that were expressed by Cluster 2 members. “Enriched day” or “Enriched night” indicates the number of those KOs significantly enriched in either the day or night samples. **(B)** Examples of individual KOs in the categories shown in **(A)**.

To characterize the transcriptional activity driving day-night differences in Cluster 2, which had the highest percentage of significant day-time %RP enriched taxa (92%), an indicator analysis was performed to identify gene expression characteristic of each time period (paired permutation test; 1000 iterations; Table S6). The nighttime indicator genes for Cluster 2 were highly enriched for AAnP-related genes: photosystems, light harvesting proteins, bacteriochlorophyll metabolism, and carotenoid biosynthesis (Figure 5). This was the strongest diel signal observed, with an 87-fold average (1232-fold maximum) relative increase in night samples compared to day across 29 AAnP indicator genes (Table S6). While the nighttime enrichment of these phototrophy-related transcripts seems counter intuitive, it is in line with previous laboratory studies of AAnP capable Rosebacters (Yurkov and Beatty, 1998). Nighttime indicator genes also included those for central metabolism, ABC transporters, mono- and dioxygeneases, and formate dehydrogenase (Figure 5). The day-time transcriptomes for Cluster 2 were instead characterized by genes for growth, repair, and energy generation (Figure 5). Indicator genes included nearly two-thirds of the 56 RPs, along with related protein synthesis machinery (RNA polymerases, tRNA synthetases, elongation factors, chaperones, and proteases), genes for energy generation via oxidative phosphorylation (F-type H+-transporting ATPases, cytochrome c enzymes, and NAD(P)H cycling), and genes for DNA repair (exonucleases, DNA ligases, and DNA photolyases) and antioxidant synthesis (glutathione peroxidase and catalase/peroxidase). Cluster 2 also included a number of metal transporter indicator genes, which in the day were biased toward cobalt (via a cobaltochelatases) and iron (via ferrochelatase), potentially linked to vitamin B12 biosynthesis and cytochrome activity, while at night were biased toward magnesium (via magnesium chelatase), potentially tied to the role of magnesium as a coordinating ion for bacteriochlorophyll.

In contrast to the many day-time %RP enriched taxa, only nine taxa had significantly higher nighttime activity indexes, and these all belonged to Cluster 7, a group with high phytoplankton membership. This counterintuitive diel pattern coincided with substantial enrichment of photosynthesis-related transcripts during the day (up to 12-fold). For both this cluster and Cluster 2, we considered the possibility that strong upregulation of phototrophy gene expression would decrease the relative contribution of RP genes to the transcript pool, resulting in %RP changes that were due to shifts in non-RP transcripts rather than changes in RP transcripts. Internal standard mRNAs added to the samples just prior to extraction (see Methods) allowed us to test this by calculating RP transcripts L^−1^ for each taxon and reanalyzing the day-night activity patterns. Of the seven eukaryotic phytoplankton in Cluster 7 with significantly higher nighttime %RP, five were no longer significant when absolute transcript counts were used (Table S5 and Figure S2); only populations binning to *Emiliania huxleyi* and *Parachlorella kessleri* still had higher nighttime expression on an absolute scale. When this same analysis was carried out on Cluster 2, however, 40 of the 43 taxa with significantly higher daytime %RP were still significant when RP transcripts L^−1^ were used instead (Table S5 and Figure S2). In all the clusters combined, 62 of the 78 heterotrophic bacterial taxa with significant %RP day-night differences were also significant with RP transcripts L^−1^ data. This included the majority of photoheterotrophs exhibiting high expression of light capture transcripts, such as most of the AAnP-capable taxa in Clusters 1 and 2 and the proteorhodopsin-capable taxa with enriched day-time %RP. Day-night shifts in activity indexes were therefore artifacts of non-RP gene expression (particularly the strongly upregulated photosynthetic machinery) for the Cluster 7 eukaryotes, but not for most bacteria, potentially due to smaller dynamics in cellular mRNA inventories in prokaryotic cells (Moran et al., 2013).

### Ecological drivers of diel activity

The most obvious ecological driver for the 44% of taxa with diel transcriptional dynamics is solar radiation, for which both direct and indirect effects could be important. Direct light effects on transcriptional patterns are expected for some photoautotrophic taxa (Ottesen et al., 2013), and we observed significant day-night dynamics in photosynthesis related transcription for phytoplankton in Clusters 5 and 7. Direct effects on light driven processes were also evident as increased transcription of phototrophy machinery in some AAnP heterotrophs. The inhibition of bacteriochlorophyll synthesis by light observed in early studies of AAnP cultures (Yurkov and Beatty, 1998) suggested our unexpected finding of nighttime AAnP transcription enrichment was also likely to be regulated by changes in solar radiation. Light regulation of phototrophy gene transcription, however, cannot fully explain the extent of the diurnal heterotrophic activity observed, as only a minority (7%) of the 200 taxa have AAnP genes, and other bacterial light capturing machinery did not exhibit strong diel dynamics (only 2 of 27 PR-capable taxa showed significant day-night differences in PR expression).

**Figure 6:**
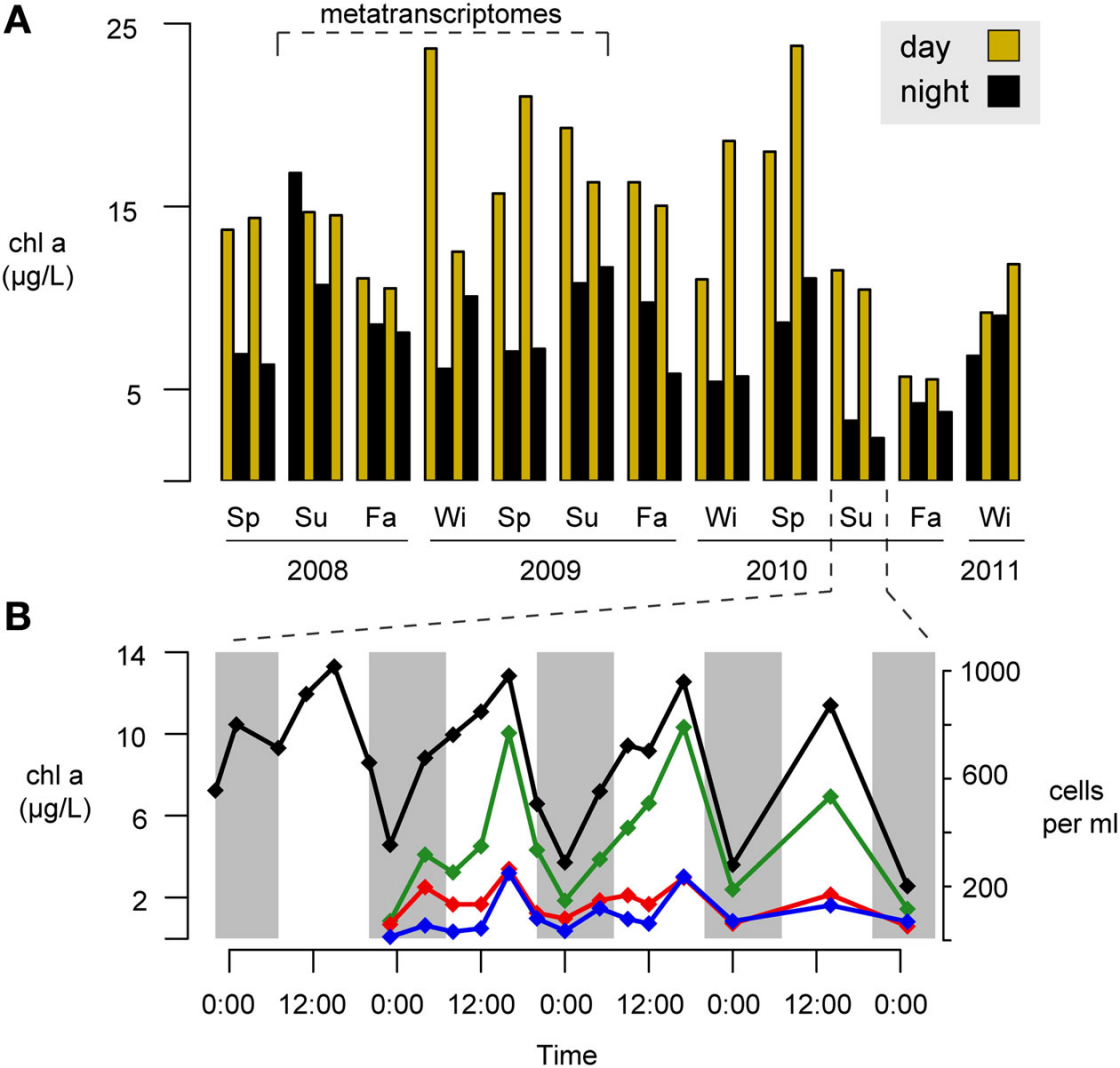
**Seasonal and day-night variability in phytoplankton concentrations at Sapelo Island, Georgia, USA. (A)** Three year time-series of chlorophyll *a* (chl *a*) concentrations. Gold and black bars indicate day and night samples, respectively. Su, summer; Fa, fall; Wi, winter; Sp, spring. **(B)** Higher temporal resolution chlorophyll *a* measurements (black line) and phytoplankton concentrations during a four-day period in Summer 2010. Green line, centric diatoms; red line, pennate diatoms; blue line, dinoflagellates. Night hours are shaded in gray.

A second possible ecological driver of day-night differences in heterotrophic activity is indirect propagation of light effects through trophic interactions with phytoplankton. We have consistently observed strong diel periodicity in phytoplankton biomass at this site based on chlorophyll *a* (Figure 6A) and cell abundance (Figure 6B) measurements. The daytime increases in phytoplankton might enhance opportunities for ecological interactions with the bacterial community, and these could be mediated through leakage and uptake of dissolved organic matter, surface attachment, or nutrient competition (Amin et al., 2012).

This “phytoplankton interaction” hypothesis may be particularly relevant for Cluster 2, which showed the strongest and most coherent day-night differences in activity, and which correlated strongly with chlorophyll *a* concentrations and PAR (Figure 2B). Further, this cluster was highly enriched in roseobacters, which have been found in close association with phytoplankton and linked to seasonal primary production patterns (Gilbert et al., 2011; Amin et al., 2012; Morris et al., 2012). Cluster 2 indicator genes for catabolism of amino acids and the photorespiration product glycolate fit with the expected composition of phytoplankton exudate (Carlucci et al., 1984; Lau et al., 2007). Our observations of a significant nighttime enrichment of amino acid transport and metabolism are in line with several other metatranscriptomic studies (Poretsky et al., 2009; Ottesen et al., 2013; Vila-Costa et al., 2013), and together with the nighttime enrichment of AAnP related transcription suggests that Cluster 2 activities during the dark may center on synthesis of the machinery necessary to take advantage of carbon and energy sources in the light.

Phytoplankton-heterotroph interactions were also suggested by several heterotrophic taxa whose activity patterns closely matched those of cyanobacteria and eukaryotic phytoplankton. In Cluster 5, a planctomycete and verrucomicrobium grouped with autotrophic *Synechococcus* and picoeukaryotes, and transcriptional data indicated that the two heterotrophs had high expression of capsular polysaccharides, twitching motility, and type IV secretion genes that together suggest an attachment lifestyle (Table S3). Members of the planctomycetes and verrucomicrobia have previously been shown to increase in abundance after phytoplankton blooms (Morris et al., 2006; Allen et al., 2012) and were also found in Cluster 7 with other eukaryotic phytoplankton.

## Conclusions

Gene expression data assigned to hundreds of reference genome bins formed a picture of a complex, dynamic bacterial community with diverse relationships to environmental gradients. We approached the analysis of this complex system by using %RP (percent of the transcriptome devoted to RP synthesis) to cluster microbial groups with similar patterns in activity through time, and then looked within the activity clusters for commonalities in taxonomy, function, and relationships to environmental parameters. Temperature was a strong overall correlate with activity patterns, dividing the community into cold-biased and warm-biased superclusters. A significant portion of the heterotrophic bacterial community, however, had even stronger day-night activity dynamics, pointing to either direct solar radiation or products of photosynthesis as the most important activity driver. Day-night differences in gene expression revealed that many heterotrophic taxa are structuring their activities toward the synthesis of transport and metabolic genes at night, while focusing on growth, energy conservation, and repair during the day. Within this seasonal/diel framework, only 55 of the 200 taxa had no detectable temporal pattern in potential activity, suggestive of the wide diversity of microbial responses and ecological interactions within this coastal ecosystem.

### Conflict of interest statement

The authors declare that the research was conducted in the absence of any commercial or financial relationships that could be construed as a potential conflict of interest.
