# Supplementary material for: Linking activity and function to ecosystem dynamics in a coastal bacterioplankton community
Source: Front Microbiol. 2014 Apr 24;5:185. doi: 10.3389/fmicb.2014.00185 (PMC4006046; doi:10.3389/fmicb.2014.00185)
Supplement: Supplementary file 1 [file DataSheet1.PDF]

Linking Activity and Function to Ecosystem Dynamics in a Coastal Bacterioplankton Community

Scott M. Gifford, Shalabh Sharma, and Mary Ann Moran

Supplementary Materials

Supplemental Tables

Table S1. Collection dates and times for RNA samples and the corresponding environmental metadata collected at Marsh Landing, Sapelo Island, GA, U.S.A.

Table S2. Summary statistics for the 22 cDNA libraries.

Table S3. KEGG ortholog (KO) indicator values for the seven activity clusters. "IV.Cluster.#" = the indicator value. "p.clade.#" = p value from the random permutation test for a significant indicator value.

Table S4. Significantly enriched KEGG pathways (summed from KO indicator analysis results; see Table S3) within broad KEGG functional categories. Clusters with a significantly greater number of pathway components within a row were identified based on bootstrapping (1,000 iterations; see Methods).

Table S5. Top 200 transcript recruiting taxa with significant day-night or seasonal differences in potential activity. “%RP day vs night” = p.value from the random permutation test for statistical differences in %RP between day and night sample pairs. %RP per L day vs night = p value from

the random permutation test for statistical differences in RP transcripts L^{-1} between day and night sample pairs. “%RP seasonal difference” **=** p value from the random permutation test for statistical differences in %RP between seasons. Colored cells indicate a p value < 0.05.

Table S6. KEGG orthologs (KOs) from Cluster 2 members significantly enriched in either the day or night samples. “perm.p.value” = p value resulting from the random permutations test. “t.p.value” = p values for pairwise t tests, shown for comparison. The library normalized abundance of a KO was calculated as the hits in a given KO bin divided by the total number of hits to all KO bins within Cluster 2. “obs.mean.diff” = the difference between the mean day sample %KO and the mean night %KO.

Supplemental Figures

Figure S1. The dendrogram in Figure 2 shown with taxon names added to the branches. The SAR11 and methylotroph subclusters within Cluster 4 are boxed in blue and pink, respectively.

Figure S2. %RP values for the top 200 recruiting taxa for each of the 20 metatranscriptome samples. Gold and black bars indicate day and night samples, respectively. The seasonal origin of the samples is indicated on the x-axis: S = summer, F = Fall, W = Winter, P = Spring. Statistical significance of day/night differences as calculated by relative (%RP) and absolute (RP per L) abundance is shown by the p value (%RP/RP L^{-1}). An asterisk (‘*’) indicates a %RP p.value < 0.05.

Figure S3 (a) Significant enrichment of Cluster 2 indicator genes in the KEGG pathways for amino acid metabolism, ABC transport, and xenobiotics metabolism. ‘*’ = Cluster 2

significantly enriched indicator genes compared to the 6 other activity clusters ($p < 0.05$, permutation test). (b) Cluster 2 indicator orthologs (KEGG KO) mapped onto the KEGG pathway ‘Microbial Metabolism in Diverse Environments”, with significant Cluster 2 indicator genes involved in aromatic metabolism highlighted in red.

Figure S4. Seasonal and day-night expression dynamics of highly expressed genes recruiting to the genome bins of (a) SAR11 HTCC7211 and (b) methylotrophic betaproteobacterium KB13. Average percent expression of the gene for the four samples within each season are connected by the red line. Gold and black bars indicate day and night samples, respectively. Su = summer; Fa = fall; Wi = winter; Sp = spring.

Figure S5. *Synechoccocus* seasonal activity profile and relationship to temperature. (a) Relative activity levels for the four *Synechococcus* bins that grouped together in the hierarchical clustering, ordered from lowest to highest %RP and colored by season (orange = fall, blue = winter, green = spring, and red = summer1 and summer 2). (b) %RP correlations with temperature for each *Synechococcus* bin.

Table S1. Collection dates and times for RNA samples and the corresponding environmental metadata collected at Marsh Landing, Sapelo Island, GA, U.S.A.

Library[1]	Date	Sampling Time	Temp.	Salinity	bacterial abundance X 10^6	Chlorophyll a	Respiration rate	bacterial production	PAR	PO4	TDN	TDP	DO
56	8/6/2008	23:35	29.3	33.6	4.2	16.8	0.0177	2.17E-09	0	1.18	15.2	1.92	5.29
57	8/6/2008	23:54	29.3	33.6	4.2	16.8	0.0177	2.17E-09	0	1.18	15.2	1.92	5.29
66	8/7/2008	0:08	29.8	33.3	5.9	10.7	0.0136	2.08E-09	0	1.33	18.1	2.14	5.77
71	8/7/2008	10:44	29.6	33.3	5.5	14.5	0.0198	1.03E-09	1025	1.38	17.5	2.16	5.45
82	11/5/2008	11:34	17.2	31.4	2.9	11.1	0.0089	9.01E-10	315	0.91	19.9	1.58	6.62
86	11/6/2008	1:08	17.1	31.2	2.2	8.5	0.0055	6.50E-10	4	0.84	19.2	1.58	6.24
91	11/6/2008	13:05	17.4	31.2	2.3	10.5	0.0041	4.79E-10	1051	0.87	17.7	1.57	6.62
96	11/7/2008	1:40	17.4	31.3	2.1	8.1	0.004	3.71E-10	4	0.81	17.2	1.54	6.29
101A	2/15/2009	12:45	13.6	28.7	3.4	23.6	0.007	4.43E-10	562	0.31	12.8	0.90	6.83
101B	2/15/2009	12:45	13.6	28.7	3.4	23.6	0.007	4.43E-10	562	0.31	12.8	0.90	6.83
106	2/16/2009	0:08	13.5	28.3	2.3	6.1	0.0041	4.77E-10	2	0.40	16.4	1.03	NA
111	2/16/2009	11:11	13.6	27.1	2.7	12.5	0.0079	2.89E-10	510	0.47	12.6	1.15	NA
116	2/17/2009	0:40	13.0	27.6	3.1	10.1	0.0029	2.66E-10	5	0.50	14.5	1.10	6.43
120	5/13/2009	11:24	24.8	27.9	4.5	15.7	0.0181	1.58E-09	1120	0.62	26.6	1.19	5.25
125	5/14/2009	23:10	24.7	29.6	3.4	7.1	0.0105	1.81E-09	0	0.59	20.6	1.19	5.55
131	5/14/2009	12:51	25.2	26.1	3.5	21.0	0.0239	1.43E-09	1390	0.58	25.5	1.25	5.86
136	5/15/2009	23:58	25.2	27.4	3.8	7.2	0.0073	7.66E-10	0	0.64	24.6	1.23	5.47
140	8/12/2009	11:26	30.2	32.1	7.7	19.3	0.0231	2.42E-09	1292	0.81	13.7	1.36	5.46
146A	8/13/2009	1:14	29.8	32.2	8.6	10.8	0.0144	2.57E-09	0	0.84	13.9	1.29	5.28
146B	8/13/2009	1:14	29.8	32.2	8.6	10.8	0.0144	2.57E-09	0	0.84	13.9	1.29	5.28
151	8/13/2009	13:59	29.6	32.1	7.5	16.3	0.0197	3.27E-09	1460	0.82	14.5	1.26	5.34
158	8/14/2009	2:30	29.6	31.7	9.2	11.7	0.0115	2.82E-09	0	0.99	16.8	1.43	5.03

Units: Temperature = °C; bacterial abundance = cells per ml, chlorophyll a = µg per L, respiration rate = µmol/min, bacterial production = kg C per L per hour ; Phostosynthetically active radiation (PAR) = µE per m^2 per s; PO4 = µM; Total Dissolved Nitrogen (TDN) = µM; Total dissolved phosphorus (TDP) = µM; Dissolved oxygen (DO) = mg per L.

Table S2. Summary statistics for the 22 cDNA libraries.

Library[1]	Date	Sampling Time	Volume Filtered	Sequencing Platform[2]	Internal Standard Added	Internal Standard Recovered	Fraction Recovered	Total Reads	Potential protein sequences recovered	RefSeq hits per library	Estimated Total Transcripts per sample	Estimated Total Transcripts per L
56	8/6/2008	23:35	5.75	GS FLX	46792452830	4,014	8.57831E-08	1,067,363	500,092	264,311	5.83E+12	1.01E+12
57	8/6/2008	23:54	5.75	GS FLX	46792452830	6,865	1.46712E-07	1,114,536	465,519	276,033	3.17E+12	5.52E+11
66	8/7/2008	0:08	5	GA-II/PE	46792452830	21,738	4.64562E-07	13,934,971	3,174,161	1,498,194	6.83E+12	1.37E+12
71	8/7/2008	10:44	6	GA-II/PE	46792452830	47,821	1.02198E-06	21,959,618	6,113,442	4,053,372	5.98E+12	9.97E+11
82	11/5/2008	11:34	5.75	GA-II/PE	47169811321	18,700	3.9644E-07	16,623,173	2,282,091	1,481,223	5.76E+12	1.00E+12
86	11/6/2008	1:08	6.25	GA-II/PE	47169811321	11,517	2.4416E-07	17,972,244	2,138,603	1,230,793	8.76E+12	1.40E+12
91	11/6/2008	13:05	5.25	GA-II/PE	47169811321	6,438	1.36486E-07	14,797,879	2,435,112	1,209,105	1.78E+13	3.40E+12
96	11/7/2008	1:40	7	GA-II	47169811321	2,205	4.6746E-08	4,851,883	1,804,900	425,307	3.86E+13	5.52E+12
101A	2/15/2009	12:45	7.5	GA-II/PE	46603773585	6,088	1.30633E-07	9,135,442	1,435,274	1,046,897	1.10E+13	1.46E+12
101B	2/15/2009	12:45	7.5	GA-II/PE	46603773585	6,247	1.34045E-07	9,341,369	1,498,274	1,088,849	1.12E+13	1.49E+12
106	2/16/2009	0:08	5	GA-II/PE	46603773585	14,120	3.0298E-07	11,694,588	2,222,219	1,148,111	7.33E+12	1.47E+12
111	2/16/2009	11:11	5.75	GA-II/PE	46603773585	7,615	1.63399E-07	10,958,924	1,779,877	1,169,139	1.09E+13	1.89E+12
116	2/17/2009	0:40	6	GA-II	46603773585	14,880	3.19287E-07	9,328,149	3,507,384	1,406,001	1.10E+13	1.83E+12
120	5/13/2009	11:24	7.5	GA-II/PE	47169811321	7,848	1.66378E-07	10,511,063	1,860,764	1,311,635	1.12E+13	1.49E+12
125	5/14/2009	23:10	6	GA-II	47169811321	19,630	4.16156E-07	7,494,746	2,563,203	907,236	6.16E+12	1.03E+12
131	5/14/2009	12:51	6	GA-II/PE	47169811321	19,187	4.06764E-07	11,251,009	1,332,199	667,236	3.28E+12	5.46E+11
136	5/15/2009	23:58	6.25	GA-II/PE	47169811321	22,694	4.81113E-07	20,915,596	2,716,622	1,546,018	5.65E+12	9.03E+11
140	8/12/2009	11:26	6	GA-II/PE	46792452830	9,290	1.98536E-07	14,305,107	2,513,708	1,580,567	1.27E+13	2.11E+12
146A	8/13/2009	1:14	6	GA-II/PE	46792452830	9,734	2.08025E-07	10,782,026	2,270,525	1,396,623	1.09E+13	1.82E+12
146B	8/13/2009	1:14	6	GA-II/PE	46792452830	10,110	2.1606E-07	11,603,140	2,366,038	1,463,680	1.10E+13	1.83E+12
151	8/13/2009	13:59	7.5	GA-II/PE	46792452830	14,436	3.08511E-07	14,376,068	3,025,727	1,960,541	9.81E+12	1.31E+12
158	8/14/2009	2:30	8	GA-II	46792452830	18,174	3.88396E-07	9,533,079	3,899,029	1,451,034	1.00E+13	1.25E+12

1) A and B are technical replicates from the same filter

2) Sequenced on: GS-FLX = a 454 GS FLX pyrosequencer; GA-II = an Illumina GA-II sequencer; GA-II/PE = Paired-end sequenced using an Illumina GA-II sequencer

Supplemental Table 4

Significantly enriched KEGG pathways (summed from KO indicator analysis results. See Supp. Table 3) within broad KEGG functional categories. Clusters with a significantly greater number of pathway components within a row than other clusters were determined by bootstrapping (1000 iterations).

KEGG pathway ID	exp. KOs	pathway description	significant pathway components							row p.values						
**1. Metabolism**																
**1.1 Carbohydrate Metabolism**			1	2	3	4	5	6	7	1	2	3	4	5	6	7
path:ko00010	42	Glycolysis / Gluconeogenesis	1	10	6	1	7	2	2	1.00	0.17	1.00	1.00	1.00	1.00	1.00
path:ko00020	39	Citrate cycle (TCA cycle)	3	11	9	0	1	1	0	1.00	0.34	1.00	1.00	1.00	1.00	1.00
path:ko00030	37	Pentose phosphate pathway	2	8	8	2	9	1	0	1.00	1.00	1.00	1.00	0.75	1.00	1.00
path:ko00040	35	Pentose and glucuronate interconversions	1	3	10	0	4	0	1	1.00	1.00	0.00	1.00	1.00	1.00	1.00
path:ko00051	41	Fructose and mannose metabolism	1	6	13	1	6	0	0	1.00	1.00	0.00	1.00	1.00	1.00	1.00
path:ko00052	22	Galactose metabolism	1	4	2	0	4	0	1	1.00	1.00	1.00	1.00	1.00	1.00	1.00
path:ko00053	15	Ascorbate and aldarate metabolism	1	2	3	0	1	1	0	1.00	1.00	0.58	1.00	1.00	1.00	1.00
path:ko00500	41	Starch and sucrose metabolism	2	2	10	0	16	2	2	1.00	1.00	1.00	1.00	0.01	1.00	1.00
path:ko00520	63	Amino sugar and nucleotide sugar metab.	1	3	11	0	16	3	4	1.00	1.00	1.00	1.00	0.06	1.00	1.00
path:ko00620	58	Pyruvate metabolism	1	16	12	2	3	3	0	1.00	0.10	1.00	1.00	1.00	1.00	1.00
path:ko00630	54	Glyoxylate and dicarboxylate metabolism	0	24	5	2	4	3	0	1.00	0.00	1.00	1.00	1.00	1.00	1.00
path:ko00640	46	Propanoate metabolism	4	15	4	1	1	3	0	1.00	0.00	1.00	1.00	1.00	1.00	1.00
path:ko00650	53	Butanoate metabolism	6	17	7	1	2	2	2	1.00	0.00	1.00	1.00	1.00	1.00	1.00
path:ko00660	11	C5-Branched dibasic acid metabolism	0	3	2	0	1	0	0	1.00	0.60	1.00	1.00	1.00	1.00	1.00
path:ko00562	13	Inositol phosphate metabolism	0	6	1	0	0	0	0	1.00	0.00	1.00	1.00	1.00	1.00	1.00
			24	130	103	10	75	21	12	1.00	0.28	1.00	1.00	1.00	1.00	1.00
**1.2 Energy Metabolism**			1	2	3	4	5	6	7	1	2	3	4	5	6	7
path:ko00190	92	Oxidative phosphorylation	4	10	15	1	19	0	13	1.00	1.00	1.00	1.00	0.16	1.00	1.00
path:ko00195	51	Photosynthesis	0	3	0	0	43	0	0	1.00	1.00	1.00	1.00	0.00	1.00	1.00
path:ko00196	23	Photosynthesis - antenna proteins	0	0	0	0	23	0	0	1.00	1.00	1.00	1.00	0.00	1.00	1.00
path:ko00710	30	Carbon fixation in photosynthetic organ.	1	6	5	2	6	2	0	1.00	1.00	1.00	1.00	1.00	1.00	1.00
path:ko00720	49	Carbon fixation pathways in prokaryotes	2	18	9	0	0	2	1	1.00	0.00	1.00	1.00	1.00	1.00	1.00
path:ko00680	81	Methane metabolism	5	16	15	0	3	2	12	1.00	0.81	1.00	1.00	1.00	1.00	1.00
path:ko00910	51	Nitrogen metabolism	0	6	7	2	8	5	2	1.00	1.00	1.00	1.00	0.75	1.00	1.00
path:ko00920	24	Sulfur metabolism	1	3	7	2	5	2	2	1.00	1.00	0.35	1.00	1.00	1.00	1.00
			13	62	58	7	107	13	30	1.00	1.00	1.00	1.00	0.00	1.00	1.00
**1.3 Lipid Metabolism**			1	2	3	4	5	6	7	1	2	3	4	5	6	7
path:ko00061	17	Fatty acid biosynthesis	2	2	3	0	1	0	0	1.00	1.00	0.57	1.00	1.00	1.00	1.00
path:ko00071	28	Fatty acid metabolism	5	12	2	0	1	0	1	1.00	0.00	1.00	1.00	1.00	1.00	1.00
path:ko00072	7	Synthesis and degradation of ketone bodies	1	4	0	0	0	0	1	1.00	0.02	1.00	1.00	1.00	1.00	1.00
path:ko00100	7	Steroid biosynthesis	0	0	1	0	2	1	0	1.00	1.00	1.00	1.00	0.57	1.00	1.00
path:ko00120	3	Primary bile acid biosynthesis	0	0	0	0	1	0	0	1.00	1.00	1.00	1.00	1.00	1.00	1.00
path:ko00121	1	Secondary bile acid biosynthesis	0	0	0	0	1	0	0	1.00	1.00	1.00	1.00	1.00	1.00	1.00
path:ko00140	9	Steroid hormone biosynthesis	0	0	1	0	0	0	0	1.00	1.00	1.00	1.00	1.00	1.00	1.00
path:ko00561	23	Glycerolipid metabolism	2	3	2	0	6	0	0	1.00	1.00	1.00	1.00	0.09	1.00	1.00
path:ko00564	33	Glycerophospholipid metabolism	2	3	3	0	5	1	0	1.00	1.00	1.00	1.00	0.27	1.00	1.00
path:ko00565	5	Ether lipid metabolism	1	0	0	0	0	0	1	1.00	1.00	1.00	1.00	1.00	1.00	1.00
path:ko00600	10	Sphingolipid metabolism	0	1	5	0	0	1	0	1.00	1.00	0.01	1.00	1.00	1.00	1.00
path:ko00590	5	Arachidonic acid metabolism	0	1	0	1	0	0	0	1.00	1.00	1.00	1.00	1.00	1.00	1.00
path:ko00591	3	Linoleic acid metabolism	0	0	1	0	1	1	0	1.00	1.00	1.00	1.00	1.00	1.00	1.00
path:ko00592	3	alpha-Linolenic acid metabolism	1	0	0	0	0	0	0	1.00	1.00	1.00	1.00	1.00	1.00	1.00
path:ko01040	13	Biosynthesis of unsaturated fatty acids	2	2	3	0	1	0	0	1.00	1.00	0.57	1.00	1.00	1.00	1.00
			16	28	21	1	19	4	3							
**1.4 Nucleotide Metabolism**			1	2	3	4	5	6	7	1	2	3	4	5	6	7
path:ko00230	124	Purine metabolism	4	26	20	1	10	4	19	1.00	0.07	1.00	1.00	1.00	1.00	1.00
path:ko00240	79	Pyrimidine metabolism	1	15	9	0	10	2	19	1.00	1.00	1.00	1.00	1.00	1.00	0.17
			5	41	29	1	20	6	38	1.00	0.45		1.00	1.00	1.00	1.00
**1.5 Amino Acid Metabolism**			1	2	3	4	5	6	7	1	2	3	4	5	6	7
path:ko00250	39	Alanine, aspartate and glutamate metab.	1	5	5	0	5	5	2	1.00	1.00	1.00	1.00	1.00	1.00	1.00
path:ko00260	57	Glycine, serine and threonine metabolism	0	20	7	1	2	4	0	1.00	0.00	1.00	1.00	1.00	1.00	1.00
path:ko00270	48	Cysteine and methionine metabolism	2	9	8	2	9	3	0	1.00	1.00	1.00	1.00	1.00	1.00	1.00
path:ko00280	38	Valine, leucine and isoleucine degradation	6	19	1	0	0	3	1	1.00	0.00	1.00	1.00	1.00	1.00	1.00
path:ko00290	22	Valine, leucine and isoleucine biosynthesis	1	5	4	0	0	0	0	1.00	0.63	1.00	1.00	1.00	1.00	1.00
path:ko00300	27	Lysine biosynthesis	0	4	1	1	1	2	3	1.00	0.66	1.00	1.00	1.00	1.00	1.00
path:ko00310	20	Lysine degradation	3	8	2	0	1	1	0	1.00	0.01	1.00	1.00	1.00	1.00	1.00
path:ko00330	89	Arginine and proline metabolism	3	26	2	2	7	7	1	1.00	0.00	1.00	1.00	1.00	1.00	1.00
path:ko00340	29	Histidine metabolism	0	7	3	0	3	3	1	1.00	0.04	1.00	1.00	1.00	1.00	1.00
path:ko00350	35	Tyrosine metabolism	2	13	1	0	1	1	1	1.00	0.00	1.00	1.00	1.00	1.00	1.00
path:ko00360	33	Phenylalanine metabolism	1	12	1	0	1	4	0	1.00	0.00	1.00	1.00	1.00	1.00	1.00
path:ko00380	30	Tryptophan metabolism	3	12	3	0	2	0	0	1.00	0.00	1.00	1.00	1.00	1.00	1.00
path:ko00400	39	Phenylalanine, tyrosine and tryptophan biosyn.	2	4	8	1	3	2	2	1.00	1.00	0.05	1.00	1.00	1.00	1.00
			24	144	46	7	35	35	11	1.00	0.00	1.00	1.00	1.00	1.00	1.00

1.6 Metabolism of Other Amino Acids			1	2	3	4	5	6	7	1	2	3	4	5	6	7
path:ko00410	24	beta-Alanine metabolism	3	8	2	0	2	2	0	1.00	0.01	1.00	1.00	1.00	1.00	1.00
path:ko00430	14	Taurine and hypotaurine metabolism	0	5	1	1	2	0	0	1.00	0.03	1.00	1.00	1.00	1.00	1.00
path:ko00440	8	Phosphonate and phosphinate metabolism	0	2	0	0	1	0	0	1.00	0.39	1.00	1.00	1.00	1.00	1.00
path:ko00450	17	Selenocompound metabolism	0	1	4	1	2	2	1	1.00	1.00	0.23	1.00	1.00	1.00	1.00
path:ko00460	10	Cyanoamino acid metabolism	1	4	0	0	2	0	0	1.00	0.18	1.00	1.00	1.00	1.00	1.00
path:ko00471	5	D-Glutamine and D-glutamate metabolism	0	3	0	0	0	1	0	1.00	0.08	1.00	1.00	1.00	1.00	1.00
path:ko00472	2	D-Arginine and D-ornithine metabolism	0	1	0	0	0	0	0	1.00	1.00	1.00	1.00	1.00	1.00	1.00
path:ko00473	4	D-Alanine metabolism	0	2	0	0	0	0	0	1.00	0.14	1.00	1.00	1.00	1.00	1.00
path:ko00480	21	Glutathione metabolism	1	7	2	2	1	1	0	1.00	0.01	1.00	1.00	1.00	1.00	1.00
			5	33	9	4	10	6	1	1.00	0.00	1.00	1.00	1.00	1.00	1.00

1.7 Glycan Biosynthesis and Metabolism			1	2	3	4	5	6	7	1	2	3	4	5	6	7
path:ko00510	5	N-Glycan biosynthesis	0	0	0	1	1	0	2	1.00	1.00	1.00	1.00	1.00	1.00	0.60
path:ko00513	1	Various types of N-glycan biosynthesis	0	0	0	0	0	0	1	1.00	1.00	1.00	1.00	1.00	1.00	1.00
path:ko00514	1	Other types of O-glycan biosynthesis	0	0	0	0	0	0	0							
path:ko00532	1	Glycosaminoglycan biosynthesis - chond.sulfate	0	0	1	0	0	0	0	1.00	1.00	1.00	1.00	1.00	1.00	1.00
path:ko00534	1	Glycosaminoglycan biosynthesis - hep. sulfate	0	0	0	0	0	0	1	1.00	1.00	1.00	1.00	1.00	1.00	1.00
path:ko00531	8	Glycosaminoglycan degradation	0	0	5	0	0	0	0	1.00	1.00	0.00	1.00	1.00	1.00	1.00
path:ko00563	1	Glycosylphosphatidylinositol(GPI)-anchor biosyn.	0	0	0	0	0	0	0							
path:ko00601	1	Glycosphingolipid biosynthesis - lacto/neolacto	0	0	0	0	0	0	0							
path:ko00603	3	Glycosphingolipid biosynthesis - globo series	0	1	0	0	0	0	0	1.00	1.00	1.00	1.00	1.00	1.00	1.00
path:ko00604	1	Glycosphingolipid biosynthesis - ganglio series	0	0	0	0	0	0	0							
path:ko00540	21	Lipopolysaccharide biosynthesis	2	0	4	0	0	1	1	1.00	1.00	0.16	1.00	1.00	1.00	1.00
path:ko00550	21	Peptidoglycan biosynthesis	0	5	2	0	2	2	0	1.00	0.06	1.00	1.00	1.00	1.00	1.00
path:ko00511	8	Other glycan degradation	0	0	2	0	1	1	0	1.00	1.00	0.60	1.00	1.00	1.00	1.00
			2	6	14	1	4	4	5	1.00	1.00	0.00	1.00	1.00	1.00	1.00

1.8 Metabolism of Cofactors and Vitamins			1	2	3	4	5	6	7	1	2	3	4	5	6	7
path:ko00730	17	Thiamine metabolism	2	3	0	0	5	0	1	1.00	1.00	1.00	1.00	0.20	1.00	1.00
path:ko00740	15	Riboflavin metabolism	2	2	0	0	3	0	3	1.00	1.00	1.00	1.00	1.00	1.00	1.00
path:ko00750	11	Vitamin B6 metabolism	2	1	2	0	0	0	2	1.00	1.00	1.00	1.00	1.00	1.00	1.00
path:ko00760	26	Nicotinate and nicotinamide metabolism	2	6	4	0	0	1	1	1.00	0.25	1.00	1.00	1.00	1.00	1.00
path:ko00770	25	Pantothenate and CoA biosynthesis	0	5	4	0	2	0	3	1.00	0.69	1.00	1.00	1.00	1.00	1.00
path:ko00780	7	Biotin metabolism	0	0	2	1	3	0	2	1.00	1.00	1.00	1.00	0.57	1.00	1.00
path:ko00785	3	Lipoic acid metabolism	0	1	1	0	1	0	0	1.00	1.00	1.00	1.00	1.00	1.00	1.00
path:ko00790	16	Folate biosynthesis	2	1	2	0	2	1	0	1.00	1.00	1.00	1.00	1.00	1.00	1.00
path:ko00670	20	One carbon pool by folate	0	6	3	0	3	0	0	1.00	0.07	1.00	1.00	1.00	1.00	1.00
path:ko00830	6	Retinol metabolism	0	1	0	0	0	0	1	1.00	1.00	1.00	1.00	1.00	1.00	1.00
path:ko00860	74	Porphyrin and chlorophyll metabolism	2	20	5	0	34	0	4	1.00	1.00	1.00	1.00	0.00	1.00	1.00
path:ko00130	25	Ubiquinone and other terpenoid-quinone biosyn.	1	4	3	0	8	1	0	1.00	1.00	1.00	1.00	0.03	1.00	1.00
			13	50	26	1	61	3	17	1.00	1.00	1.00	1.00	0.02	1.00	1.00

1.9 Metabolism of Terpenoids and Polyketides			1	2	3	4	5	6	7		1	2	3	4	5	6	7
path:ko00900	25	Terpenoid backbone biosynthesis	3	5	1	0	7	1	2		1.00	1.00	1.00	1.00	0.32	1.00	1.00
path:ko00906	16	Carotenoid biosynthesis	0	3	0	0	7	2	1		1.00	1.00	1.00	1.00	0.02	1.00	1.00
path:ko00903	13	Limonene and pinene degradation	3	4	1	0	0	0	0		1.00	0.58	1.00	1.00	1.00	1.00	1.00
path:ko00281	16	Geraniol degradation	7	4	1	0	0	0	0		0.08	1.00	1.00	1.00	1.00	1.00	1.00
path:ko01051	2	Biosynthesis of ansamycins	0	0	1	1	1	0	0		1.00	1.00	1.00	1.00	1.00	1.00	1.00
path:ko00253	4	Tetracycline biosynthesis	0	0	0	0	1	0	0		1.00	1.00	1.00	1.00	1.00	1.00	1.00
path:ko00523	4	Polyketide sugar unit biosynthesis	0	0	1	0	1	1	0		1.00	1.00	1.00	1.00	1.00	1.00	1.00
path:ko01053	6	Biosyn. of siderophore . nonribosomal peptides	0	0	1	0	1	0	0		1.00	1.00	1.00	1.00	1.00	1.00	1.00
path:ko01055	1	Biosyn. of vancomycin group antibiotics	0	0	1	0	1	0	0		1.00	1.00	1.00	1.00	1.00	1.00	1.00
			13	16	7	1	19	4	3	0	1.00	1.00	1.00	1.00	0.31	1.00	1.00

1.10 Biosynthesis of Other Secondary Metabolites			1	2	3	4	5	6	7	1	2	3	4	5	6	7
path:ko00940	8	Phenylpropanoid biosynthesis	1	3	0	0	1	0	0	1.00	0.14	1.00	1.00	1.00	1.00	1.00
path:ko00941	4	Flavonoid biosynthesis	0	0	0	1	1	0	0	1.00	1.00	1.00	1.00	1.00	1.00	1.00
path:ko00944	1	Flavone and flavonol biosynthesis	0	0	1	0	0	0	0	1.00	1.00	1.00	1.00	1.00	1.00	1.00
path:ko00901	1	Indole alkaloid biosynthesis	0	1	0	0	0	0	0	1.00	1.00	1.00	1.00	1.00	1.00	1.00
path:ko00950	8	Isoquinoline alkaloid biosynthesis	1	2	0	0	0	1	0	1.00	0.62	1.00	1.00	1.00	1.00	1.00
path:ko00960	12	Tropane, piperidine and pyridine alkaloid biosyn.	2	2	2	0	1	1	0	1.00	1.00	1.00	1.00	1.00	1.00	1.00
path:ko00232	3	Caffeine metabolism	0	0	0	1	1	0	0	1.00	1.00	1.00	1.00	1.00	1.00	1.00
path:ko00965	1	Betalain biosynthesis	0	1	0	0	0	0	0	1.00	1.00	1.00	1.00	1.00	1.00	1.00
path:ko00311	7	Penicillin and cephalosporin biosynthesis	1	1	0	0	2	0	0	1.00	1.00	1.00	1.00	0.61	1.00	1.00
path:ko00312	1	beta-Lactam resistance	0	0	0	0	1	0	0	1.00	1.00	1.00	1.00	1.00	1.00	1.00
path:ko00521	10	Streptomycin biosynthesis	0	1	1	0	3	1	0	1.00	1.00	1.00	1.00	0.17	1.00	1.00
path:ko00524	1	Butirosin and neomycin biosynthesis	0	0	0	0	1	0	0	1.00	1.00	1.00	1.00	1.00	1.00	1.00
path:ko00401	8	Novobiocin biosynthesis	1	2	2	0	0	1	0	1.00	1.00	1.00	1.00	1.00	1.00	1.00
			6	13	6	2	11	4	0	1.00	0.45	1.00	1.00	1.00	1.00	1.00

1.11 Xenobiotics Biodegradation and Metabolism			1	2	3	4	5	6	7	1	2	3	4	5	6	7
path:ko00362	55	Benzoate degradation	3	12	2	1	0	1	0	1.00	0.00	1.00	1.00	1.00	1.00	1.00
path:ko00627	42	Aminobenzoate degradation	2	7	2	0	4	0	0	1.00	0.09	1.00	1.00	1.00	1.00	1.00
path:ko00364	12	Fluorobenzoate degradation	1	1	0	1	1	0	0	1.00	1.00	1.00	1.00	1.00	1.00	1.00
path:ko00625	16	Chloroalkane and chloroalkene degradation	0	4	1	0	1	0	1	1.00	0.05	1.00	1.00	1.00	1.00	1.00
path:ko00361	17	Chlorocyclohexane and chlorobenzene degrad.	0	3	0	1	1	0	0	1.00	0.11	1.00	1.00	1.00	1.00	1.00
path:ko00623	20	Toluene degradation	1	2	2	1	1	0	0	1.00	1.00	1.00	1.00	1.00	1.00	1.00
path:ko00622	12	Xylene degradation	0	1	1	0	0	1	0	1.00	1.00	1.00	1.00	1.00	1.00	1.00
path:ko00633	10	Nitrotoluene degradation	0	3	1	0	0	2	0	1.00	0.57	1.00	1.00	1.00	1.00	1.00
path:ko00642	8	Ethylbenzene degradation	1	1	0	0	0	0	0	1.00	1.00	1.00	1.00	1.00	1.00	1.00
path:ko00643	12	Styrene degradation	0	6	0	1	1	0	0	1.00	0.00	1.00	1.00	1.00	1.00	1.00
path:ko00791	9	Atrazine degradation	0	4	0	0	1	0	0	1.00	0.01	1.00	1.00	1.00	1.00	1.00
path:ko00930	11	Caprolactam degradation	4	2	3	0	0	0	0	0.61	1.00	1.00	1.00	1.00	1.00	1.00
path:ko00351	5	DDT degradation	0	1	0	0	0	1	0	1.00	1.00	1.00	1.00	1.00	1.00	1.00
path:ko00363	10	Bisphenol degradation	0	1	1	0	0	1	0	1.00	1.00	1.00	1.00	1.00	1.00	1.00
path:ko00621	11	Dioxin degradation	0	1	1	0	0	1	0	1.00	1.00	1.00	1.00	1.00	1.00	1.00
path:ko00626	21	Naphthalene degradation	1	5	0	0	1	0	1	1.00	0.01	1.00	1.00	1.00	1.00	1.00
path:ko00624	17	Polycyclic aromatic hydrocarbon degradation	0	4	1	0	0	1	0	1.00	0.03	1.00	1.00	1.00	1.00	1.00
path:ko00980	6	Metabolism of xenobiotics by cytochrome P450	0	2	0	0	0	0	1	1.00	0.37	1.00	1.00	1.00	1.00	1.00
path:ko00982	8	Drug metabolism - cytochrome P450	0	4	0	0	0	0	1	1.00	0.02	1.00	1.00	1.00	1.00	1.00
path:ko00983	18	Drug metabolism - other enzymes	1	8	2	0	3	0	0	1.00	0.01	1.00	1.00	1.00	1.00	1.00
			14	72	17	5	14	8	4	1.00	0.00	1.00	1.00	1.00	1.00	1.00

2. Genetic Information Processing

2.1 Transcription			1	2	3	4	5	6	7	1	2	3	4	5	6	7
path:ko03020	16	RNA polymerase	0	1	1	0	0	1	12	1.00	1.00	1.00	1.00	1.00	1.00	0.00
path:ko03022	5	Basal transcription factors	0	0	0	0	0	0	4	1.00	1.00	1.00	1.00	1.00	1.00	0.00
path:ko03040	1	Spliceosome	0	0	0	0	0	0	1	1.00	1.00	1.00	1.00	1.00	1.00	1.00
			0	1	1	0	0	1	17	1.00	1.00	1.00	1.00	1.00	1.00	0.00

2.2 Translation			1	2	3	4	5	6	7	1	2	3	4	5	6	7
path:ko03010	83	Ribosome	1	3	9	0	2	1	29	1.00	1.00	1.00	1.00	1.00	1.00	0.00
path:ko00970	33	Aminoacyl-tRNA biosynthesis	3	7	2	0	0	0	2	1.00	0.03	1.00	1.00	1.00	1.00	1.00
path:ko03013	12	RNA transport	1	0	1	0	1	0	8	1.00	1.00	1.00	1.00	1.00	1.00	0.00
path:ko03015	2	mRNA surveillance pathway	0	0	0	0	0	0	2	1.00	1.00	1.00	1.00	1.00	1.00	0.15
path:ko03008	5	Ribosome biogenesis in eukaryotes	0	1	1	0	0	0	2	1.00	1.00	1.00	1.00	1.00	1.00	0.61
			5	11	13	0	3	1	43	1.00	1.00	1.00	1.00	1.00	1.00	0.00

2.3 Folding, Sorting and Degradation			1	2	3	4	5	6	7	1	2	3	4	5	6	7
path:ko03060	20	Protein export	0	3	3	0	0	1	3	1.00	1.00	1.00	1.00	1.00	1.00	1.00
path:ko04122	17	Sulfur relay system	2	3	1	0	3	1	4	1.00	1.00	1.00	1.00	1.00	1.00	0.66
path:ko03050	2	Proteasome	0	0	0	0	0	0	2	1.00	1.00	1.00	1.00	1.00	1.00	0.16
path:ko03018	20	RNA degradation	2	5	3	0	2	0	4	1.00	0.70	1.00	1.00	1.00	1.00	1.00
			4	11	7	0	5	2	13	1.00	1.00	1.00	1.00	1.00	1.00	0.49

2.4 Replication and Repair			1	2	3	4	5	6	7	1	2	3	4	5	6	7
path:ko03030	21	DNA replication	0	0	3	0	0	1	6	1.00	1.00	1.00	1.00	1.00	1.00	0.05
path:ko03410	21	Base excision repair	2	1	4	0	3	0	3	1.00	1.00	0.66	1.00	1.00	1.00	1.00
path:ko03420	14	Nucleotide excision repair	0	1	1	0	1	0	5	1.00	1.00	1.00	1.00	1.00	1.00	0.01
path:ko03430	24	Mismatch repair	0	0	4	0	3	1	4	1.00	1.00	1.00	1.00	1.00	1.00	1.00
path:ko03440	25	Homologous recombination	1	0	4	0	3	1	2	1.00	1.00	0.65	1.00	1.00	1.00	1.00
path:ko03450	3	Non-homologous end-joining	0	0	0	0	1	0	1	1.00	1.00	1.00	1.00	1.00	1.00	1.00
			3	2	16	0	10	3	20	1.00	1.00	1.00	1.00	1.00	1.00	0.16

3. Environmental Information Processing

3.1 Membrane Transport			1	2	3	4	5	6	7	1	2	3	4	5	6	7
path:ko02010	202	ABC transporters	6	73	12	1	19	2	1	1.00	0.00	1.00	1.00	1.00	1.00	1.00
path:ko02060	12	Phosphotransferase system (PTS)	2	3	3	0	1	0	0	1.00	1.00	1.00	1.00	1.00	1.00	1.00
path:ko03070	49	Bacterial secretion system	9	3	10	0	1	1	0	1.00	1.00	0.74	1.00	1.00	1.00	1.00
			17	79	25	1	21	3	1	1.00	0.00	1.00	1.00	1.00	1.00	1.00

3.2 Signal Transduction			1	2	3	4	5	6	7	1	2	3	4	5	6	7
path:ko02020	157	Two-component system	7	22	31	4	11	6	8	1.00	1.00	0.02	1.00	1.00	1.00	1.00
path:ko04012	1	ErbB signaling pathway	0	0	0	0	1	0	0	1.00	1.00	1.00	1.00	1.00	1.00	1.00
path:ko04310	2	Wnt signaling pathway	0	0	0	0	1	0	1	1.00	1.00	1.00	1.00	1.00	1.00	1.00
path:ko04330	1	Notch signaling pathway	0	0	0	0	0	0	1	1.00	1.00	1.00	1.00	1.00	1.00	1.00
path:ko04020	2	Calcium signaling pathway	0	0	0	0	2	0	0	1.00	1.00	1.00	1.00	0.14	1.00	1.00
path:ko04070	3	Phosphatidylinositol signaling system	0	1	0	0	2	0	0	1.00	1.00	1.00	1.00	0.40	1.00	1.00
path:ko04150	1	mTOR signaling pathway	0	0	0	0	0	0	1	1.00	1.00	1.00	1.00	1.00	1.00	1.00
			7	23	31	4	17	6	11	1.00	1.00	0.29	1.00	1.00	1.00	1.00

3.3 Signaling Molecules and Interaction			1	2	3	4	5	6	7	1	2	3	4	5	6	7
Neuroactive ligaand-rece		path:ko04080	0	0	0	0	0	0	0							
ECM-receptor ir:eptor int		path:ko04512	0	0	0	0	0	0	1							

4. Cellular Processes

4.1 Transport and Catabolism

			1	2	3	4	5	6	7	1	2	3	4	5	6	7
path:ko04145	2	Phagosome	0	0	0	0	0	0	2	1.00	1.00	1.00	1.00	1.00	1.00	0.13
path:ko04142	16	Lysosome	0	0	8	0	0	1	2	1.00	1.00	0.00	1.00	1.00	1.00	1.00
path:ko04146	23	Peroxisome	3	3	0	0	2	1	0	1.00	1.00	1.00	1.00	1.00	1.00	1.00
			3	3	8	0	2	2	4	1.00	1.00	0.51	1.00	1.00	1.00	1.00

4.2 Cell Motility

			1	2	3	4	5	6	7	1	2	3	4	5	6	7
path:ko02030	22	Bacterial chemotaxis	2	0	9	0	0	1	0	1.00	1.00	0.00	1.00	1.00	1.00	1.00
path:ko02040	33	Flagellar assembly	13	0	15	0	0	4	0	1.00	1.00	0.42	1.00	1.00	1.00	1.00
			15	0	24	0	0	5	0	1.00	1.00	0.00	1.00	1.00	1.00	1.00

4.3 Cell Growth and Death

			1	2	3	4	5	6	7	1	2	3	4	5	6	7
path:ko04110	2	Cell cycle	0	0	0	0	0	0	2	1.00	1.00	1.00	1.00	1.00	1.00	0.12
path:ko04111	1	Cell cycle - yeast	0	0	0	0	0	0	1	1.00	1.00	1.00	1.00	1.00	1.00	1.00
path:ko04112	26	Cell cycle - Caulobacter	1	6	3	4	1	0	0	1.00	0.27	1.00	1.00	1.00	1.00	1.00
path:ko04113	3	Meiosis - yeast	0	0	1	0	0	0	1	1.00	1.00	1.00	1.00	1.00	1.00	1.00
path:ko04114	1	Oocyte meiosis	0	0	0	0	1	0	0	1.00	1.00	1.00	1.00	1.00	1.00	1.00
path:ko04210	2	Apoptosis	0	1	0	0	0	1	0	1.00	1.00	1.00	1.00	1.00	1.00	1.00
path:ko04115	3	p53 signaling pathway	0	1	0	0	0	0	0	1.00	1.00	1.00	1.00	1.00	1.00	1.00
			1	8	4	4	2	1	4	1.00	0.06	1.00	1.00	1.00	1.00	1.00

4.4 Cell Communication

			1	2	3	4	5	6	7	1	2	3	4	5	6	7
path:ko04510	1	Focal adhesion	0	0	0	0	0	0	1	1.00	1.00	1.00	1.00	1.00	1.00	1.00
			0	0	0	0	0	0	1	1.00	1.00	1.00	1.00	1.00	1.00	1.00

Table S5

		p.values		
activity cluster	taxon name	%RP day vs night	RP per L day vs night	%RP seasonal difference
1	marine gamma proteobacterium HTCC2080	0.0031	0.0353	0.0717
1	Congregibacter litoralis KT71	0.0032	0.0381	0.0686
1	Candidatus Puniceispirillum marinum IMCC1322	0.0034	0.0544	0.3013
1	Saccharophagus degradans 2-40	0.0034	0.0505	0.0093
1	gamma proteobacterium IMCC3088	0.0034	0.0489	0.0045
1	Endoriftia persephone 'Hot96_1+Hot96_2'	0.0035	0.1191	0.0152
1	Flavobacteria bacterium MS024-3C	0.0039	0.0247	0.0009
1	marine gamma proteobacterium HTCC2148	0.004	0.0386	0.0259
1	Flavobacteria bacterium BAL38	0.004	0.0459	0.0045
1	Methylomicrobium album BG8	0.0042	0.0531	0.0049
1	gamma proteobacterium NOR51-B	0.0049	0.0316	0.0429
1	gamma proteobacterium NOR5-3	0.0067	0.0483	0.2711
1	marine gamma proteobacterium HTCC2143	0.0081	0.0744	0.0489
1	Hahella chejuensis KCTC 2396	0.0084	0.0307	0.0236
1	Rhodobacterales bacterium HTCC2255	0.0265	0.0077	0.0105
1	Cellvibrio japonicus Ueda107	0.0318	0.0511	0.0004
1	Azospirillum sp. B510	0.0359	0.1083	0.2276
1	Marinobacter sp. ELB17	0.0571	0.1187	0.0226
1	Leeuwenhoekiella blandensis MED217	0.0709	0.0671	0.8315
1	Octadecabacter antarcticus 307	0.0724	0.0089	0.0372
1	Polaribacter sp. MED152	0.0756	0.0498	0.0196
1	Thioalkalivibrio sulfidophilus HL-EbGr7	0.0784	0.1451	0.0043
1	Marinobacter aquaeolei VT8	0.0849	0.0748	0.0267
1	Sulfitobacter sp. NAS-14.1	0.1083	0.0075	0.2013
1	gamma proteobacterium HTCC2207	0.1113	0.0781	0.0332
1	Kangiella koreensis DSM 16069	0.1216	0.1313	0.0013
1	Rhodobacter sphaeroides ATCC 17025	0.1372	0.1102	0.1091
1	Zunongwangia profunda SM-A87	0.1441	0.0501	0.1227
1	Pseudomonas stutzeri A1501	0.1553	0.1165	0.0311
1	Roseomonas cervicalis ATCC 49957	0.1589	0.1795	0.1548
1	Capnocytophaga sp. oral taxon 329 str. F0087	0.1606	0.0359	0.0061
1	Marinomonas mediterranea MMB-1	0.163	0.0686	0.0153
1	Maricaulis maris MCS10	0.1679	0.115	0.0123
1	Pseudomonas mendocina NK-01	0.185	0.2245	0.0007
1	Marinomonas sp. MED121	0.2152	0.1133	0.2089
1	Croceibacter atlanticus HTCC2559	0.2325	0.0498	0.0612
1	Spirosoma linguale DSM 74	0.3015	0.0522	0.0033
1	Lacinutrix algicola 5H-3-7-4	0.3212	0.0327	0.036
1	Teredinibacter turnerae T7901	0.3522	0.1187	0.0002
1	Thioalkalivibrio sp. K90mix	0.39	0.224	0.1151
1	Glaciecola sp. HTCC2999	0.4334	0.746	0.0004
1	Mucilaginibacter paludis DSM 18603	0.5898	0.0669	0.0066
1	Methylobacter tundripaludum SV96	0.6411	0.2815	0.0276
1	Glaciecola sp. 4H-3-7+YE-5	0.8549	0.2678	0
1	Colwellia psychrerythraea 34H	0.8765	0.3004	0.0004
1	Verrucomicrobium spinosum DSM 4136	0.9317	0.1389	0.0007
1	Alteromonas sp. SN2	0.9782	0.1605	0.0005
2	Robiginitalea biformata HTCC2501	0.0032	0.0197	0.2623
2	Oceanicola batsensis HTCC2597	0.0035	0.0027	0.6705
2	Roseobacter sp. MED193	0.0036	0.0044	0.5448
2	Octadecabacter antarcticus 238	0.0036	0.0038	0.9136
2	Cellulophaga algicola DSM 14237	0.0039	0.0152	0.1743
2	Jannaschia sp. CCS1	0.0041	0.0044	0.0563
2	Marinomonas sp. MWYL1	0.0041	0.0638	0.5404
2	Oceanicola granulosus HTCC2516	0.0042	0.0042	0.5821
2	Roseovarius sp. TM1035	0.0043	0.004	0.9047
2	gamma proteobacterium HdN1	0.0043	0.0316	0.9118
2	Roseobacter sp. AzwK-3b	0.0045	0.0071	0.8598
2	Rhodobacter capsulatus SB 1003	0.0045	0.0083	0.3777
2	Ruegeria sp. R11	0.0047	0.0039	0.731
2	Chromohalobacter salexigens DSM 3043	0.0047	0.0581	0.3389
2	Neptuniibacter caesariensis	0.0049	0.0213	0.2852
2	Roseovarius sp. 217	0.0053	0.0081	0.7698
2	Halomonas elongata DSM 2581	0.0059	0.07	0.1639
2	Phaeobacter gallaeciensis DSM 17395	0.007	0.0077	0.1133
2	Roseobacter sp. SK209-2-6	0.0075	0.0035	0.617
2	Ruegeria pomeroyi DSS-3	0.0077	0.0037	0.2126
2	Rhodobacter sp. SW2	0.0083	0.0324	0.665
2	Rhodobacterales bacterium Y4I	0.0085	0.0036	0.8826
2	Dinoroseobacter shibae DFL 12	0.0085	0.0039	0.4079
2	Maritimibacter alkaliphilus HTCC2654	0.0089	0.0038	0.4601
2	Maribacter sp. HTCC2170	0.01	0.0251	0.8152
2	Loktanella vestfoldensis SKA53	0.011	0.0045	0.6441
2	Pelagibaca bermudensis HTCC2601	0.011	0.0031	0.5039
2	Sagittula stellata E-37	0.0119	0.0045	0.4841
2	Roseovarius nubinhibens ISM	0.0122	0.004	0.9274
2	Ruegeria sp. TM1040	0.0146	0.0032	0.6004
2	Oceanibulbus indolifex HEL-45	0.0155	0.0026	0.3799
2	Rhodobacteraceae bacterium KLH11	0.0155	0.0042	0.9425
2	unidentified eubacterium SCB49	0.0155	0.0399	0.51
2	Silicibacter sp. TrichCH4B	0.0174	0.0032	0.667
2	Ketogulonicigenium vulgare Y25	0.0182	0.0048	0.4909
2	Kordia algicida OT-1	0.0232	0.021	0.9887
2	Citreicella sp. SE45	0.0249	0.0039	0.7796
2	Roseobacter litoralis Och 149	0.0253	0.0377	0.8495
2	Silicibacter lacuscaerulensis ITI-1157	0.0265	0.0033	0.87
2	Rhodobacteraceae bacterium HTCC2083	0.0277	0.0036	0.6402
2	Roseobacter denitrificans OCh 114	0.0315	0.0114	0.1661
2	Rhodobacteraceae bacterium HTCC2150	0.0346	0.0039	0.3251
2	Thalassiobium sp. R2A62	0.0447	0.0038	0.9505
2	Roseobacter sp. CCS2	0.0536	0.0087	0.9203
2	Cellulophaga lytica DSM 7489	0.0552	0.0435	0.637
2	Roseobacter sp. GAI101	0.0554	0.0032	0.4624
2	Paracoccus denitrificans PD1222	0.1067	0.0312	0.736
3	Coraliomargarita akajimensis DSM 45221	0.005	0.1274	0.0006
3	Pirellula staleyi DSM 6068	0.1104	0.7567	0.0068
3	Alcanivorax sp. DG881	0.1989	0.1054	0.2629
3	Thalassiosira pseudonana CCMP1335	0.2618	0.7361	0.0681
3	Methylomonas methanica MC09	0.2905	0.5777	0.3775
3	Rhodopirellula baltica SH 1	0.4637	0.6994	0.0033
3	Verrucomicrobiae bacterium DG1235	0.5692	0.1642	0.0096
3	Blastopirellula marina DSM 3645	0.6038	0.6486	0.0123
3	Marinomonas posidonica IVIA-Po-181	0.7314	0.2664	0.0035
3	Opitutus terrae PB90-1	0.7458	0.1871	0.0001
3	Ferrimonas balearica DSM 9799	0.7723	0.1274	0.073
4	Flavobacteria bacterium BBFL7	0.0029	0.0284	0.0471
4	alpha proteobacterium BAL199	0.0035	0.0724	0.0178
4	Reinekea blandensis MED297	0.0042	0.0485	0.1517
4	Flavobacteria bacterium MS024-2A	0.0043	0.019	0.0009
4	Hoeflea phototrophica DFL-43	0.0044	0.0457	0.0742
4	Methylophilales bacterium HTCC2181	0.0045	0.0129	0.2369
4	beta proteobacterium KB13	0.0069	0.012	0.1249
4	Flavobacteriales bacterium ALC-1	0.0083	0.0295	0.0206
4	Novosphingobium sp. PP1Y	0.0112	0.6906	0.0051
4	Algoriphagus sp. PR1	0.0117	0.0337	0.0824
4	Allochromatium vinosum DSM 180	0.0163	0.0961	0.0005
4	Candidatus Pelagibacter sp. IMCC9063	0.0169	0.1371	0.0395
4	Labrenzia aggregata IAM 12614	0.0231	0.0957	0.0075
4	Parvibaculum lavamentivorans DS-1	0.0295	0.247	0.0202
4	Marinobacter algicola DG893	0.0333	0.0389	0.0467
4	gamma proteobacterium IMCC1989	0.0372	0.0546	0.0016
4	Ahrensia sp. R2A130	0.0551	0.1689	0.0124
4	Candidatus Pelagibacter sp. HTCC7211	0.0582	0.1009	0.0051
4	Fulvimarina pelagi HTCC2506	0.0605	0.266	0.0027
4	Pseudovibrio sp. JE062	0.0634	0.391	0.0038
4	Rhodospirillum centenum SW	0.0727	0.1775	0.0047
4	Rhodospirillum rubrum ATCC 11170	0.078	0.2643	0.3542
4	Gramella forsetii KT0803	0.0858	0.0333	0.0288
4	Roseibium sp. TrichSKD4	0.0915	0.0998	0.0161
4	Haliscomenobacter hydrossis DSM 1100	0.1049	0.057	0
4	Hyphomonas neptunium ATCC 15444	0.1221	0.049	0.0007
4	Dyadobacter fermentans DSM 18053	0.1232	0.0335	0.0131
4	Krokinobacter sp. 4H-3-7-5	0.1503	0.0401	0.0163
4	Candidatus Pelagibacter ubique HTCC1002	0.154	0.155	0.0405
4	Rhodothermus marinus DSM 4252	0.1625	0.155	0.0516
4	Pseudoalteromonas tunicata D2	0.1952	0.0224	0.0162
4	Methyloversatilis universalis FAM5	0.2493	0.6875	0.1132
4	alpha proteobacterium HIMB114	0.2631	0.3566	0.0007
4	Pseudomonas fulva 12-X	0.2674	0.1564	0.0008
4	Marivirga tractuosa DSM 4126	0.3186	0.0344	0.0029
4	Microscilla marina ATCC 23134	0.3214	0.053	0.157
4	Oceanicaulis sp. HTCC2633	0.3246	0.1835	0.0015
4	Bradyrhizobium sp. ORS 278	0.3842	0.5547	0.0804
4	Polymorphum gilvum SL003B-26A1	0.3909	0.7466	0.0015
4	Labrenzia alexandrii DFL-11	0.3923	0.8732	0.0013
4	Haliangium ochraceum DSM 14365	0.4095	0.2598	0.0006
4	Bradyrhizobium japonicum USDA 110	0.4153	0.6511	0.0078
4	Chitinophaga pinensis DSM 2588	0.5129	0.0789	0.001
4	Methylophaga thiooxydans DMS010	0.5171	0.2426	0
4	Fluviicola taffensis DSM 16823	0.5315	0.0686	0.0092
4	gamma proteobacterium HTCC5015	0.6175	0.311	0.0263
4	Magnetospirillum magneticum AMB-1	0.628	0.3918	0.0826
4	Aurantimonas manganoxydans SI85-9A1	0.6713	0.8767	0.0002
4	Hirschia baltica ATCC 49814	0.766	0.2279	0.0005
4	Candidatus Pelagibacter ubique HTCC1062	0.7692	0.1913	0.0004
4	Thiobacillus denitrificans ATCC 25259	0.8106	0.5446	0.0002
4	Starkeya novella DSM 506	0.8532	0.4176	0.0057
4	Planctomyces maris DSM 8797	0.9615	0.4884	0.0001
4	Nitrosococcus halophilus Nc4	0.9918	0.0691	0.0032
5	Synechococcus sp. RS9916	0.0164	0.005	0.017
5	Pedosphaera parvula Ellin514	0.1055	0.9226	0.0001
5	Synechococcus sp. RCC307	0.1229	0.1993	0.4847
5	Synechococcus sp. CB0101	0.1244	0.0576	0.0002
5	Chlamydomonas reinhardtii	0.1884	0.0896	0.1304
5	Chlorella variabilis	0.2054	0.1562	0.2232
5	Volvox carteri f. nagariensis	0.221	0.1689	0.0102
5	Synechococcus sp. CC9605	0.407	0.0364	0.3175
5	Synechococcus sp. CB0205	0.4174	0.0088	0.001
5	Ostreococcus tauri	0.5455	0.3658	0.1864
5	Ostreococcus lucimarinus CCE9901	0.8387	0.3142	0.7415
5	Synechococcus sp. WH 8109	0.8459	0.1166	0.0191
5	Planctomyces brasiliensis DSM 5305	0.8814	0.8357	0.0001
6	Phytophthora infestans T30-4	0.0808	0.7173	0.6409
6	Alkalilimnicola ehrlichii MLHE-1	0.2267	0.1001	0.0293
6	Legionella pneumophila 2300/99 Alcoy	0.364	0.1366	0.1191
6	Opitutaceae bacterium TAV2	0.5265	0.2216	0.1999
6	Psychroflexus torquis ATCC 700755	0.6265	0.0577	0.1348
7	Emiliania huxleyi	0.0029	0.0429	0.7725
7	Monomastix sp. OKE-1	0.0035	0.0906	0.1666
7	Oltmannsiellopsis viridis	0.0039	0.3626	0.1203
7	Candidatus Nitrosoarchaeum limnia SFB1	0.0071	0.0445	0.0199
7	Parachlorella kessleri	0.0124	0.0385	0.5462
7	Micromonas sp. RCC299	0.019	0.4092	0.762
7	Rhodomonas salina	0.0353	0.1749	0.0508
7	Coccomyxa sp. C-169	0.037	0.5879	0.3745
7	Lentisphaera araneosa HTCC2155	0.0487	0.7839	0.3849
7	Beggiatoa sp. PS	0.0557	0.7209	0.1019
7	Candidatus Vesicomyosocius okutanii HA	0.061	0.4879	0.1204
7	Nitrosopumilus maritimus SCM1	0.0622	0.0525	0.5037
7	Gemmata obscuriglobus UQM 2246	0.0651	0.5608	0.4958
7	Micromonas pusilla CCMP1545	0.0996	0.3278	0.3505
7	Gluconacetobacter diazotrophicus PAl 5	0.1131	0.6308	0.088
7	Oedogonium cardiacum	0.2192	0.398	0.7042
7	Candidatus Ruthia magnifica str. Cm Calyptogena magnific	0.2346	0.6996	0.0154
7	Acidithiobacillus ferrivorans SS3	0.3095	0.3178	0.2619
7	Thalassiosira oceanica CCMP1005	0.3281	0.0996	0.2459
7	Chthoniobacter flavus Ellin428	0.3504	0.9496	0.2384
7	Bermanella marisrubri	0.4966	0.1004	0.2858
7	Halothiobacillus neapolitanus c2	0.6697	0.3707	0.3127

Supplemental Table 6. KEGG orthologs (KOs) from RP clade 2 members only significantly enriched in either the day or night samples. "perm.p.value" is the p.value resulting from the random permutations test. The p.values for pairwise t.tests are shown for comparison ("t.p.value"). The library normalized abundance of a KO was calculated as the hits falling into a given KO bin divided by the total number of hits to all KO bins within clade 2. "obs.mean.diff" is the difference between the mean daytime sample %KO and the mean nighttime %KO.

KEGG.KO.id	perm.p.value	t.p.value	obs.mean.diff	ko.desc
KO:K00001	0.0031	0.024	5.00E-04	alcohol dehydrogenase [EC:1.1.1.1]
KO:K00012	0.0459	0.0588	-1.00E-04	UDPglucose 6-dehydrogenase [EC:1.1.1.22]
KO:K00015	0.0363	0.0274	3.00E-04	glyoxylate reductase [EC:1.1.1.26]
KO:K00020	0.0027	0.0188	-2.00E-04	3-hydroxyisobutyrate dehydrogenase [EC:1.1.1.31]
KO:K00023	0.0043	0.0047	3.00E-04	acetoacetyl-CoA reductase [EC:1.1.1.36]
KO:K00031	0.0334	0.0239	-5.00E-04	isocitrate dehydrogenase [EC:1.1.1.42]
KO:K00036	0.0252	0.0246	-1.00E-04	glucose-6-phosphate 1-dehydrogenase [EC:1.1.1.49]
KO:K00037	0.0235	0.0099	-1.00E-04	3-alpha-hydroxysteroid dehydrogenase [EC:1.1.1.50]
KO:K00053	0.0073	0.0264	0.001	ketol-acid reductoisomerase [EC:1.1.1.86]
KO:K00060	0.0172	0.0129	0	threonine 3-dehydrogenase [EC:1.1.1.103]
KO:K00076	0.0142	0.0097	0	7-alpha-hydroxysteroid dehydrogenase [EC:1.1.1.159]
KO:K00077	0.0407	0.0439	3.00E-04	2-dehydropantoate 2-reductase [EC:1.1.1.169]
KO:K00102	0.0231	0.0236	0	D-lactate dehydrogenase (cytochrome) [EC:1.1.2.4]
KO:K00108	0.0043	2.00E-04	-3.00E-04	choline dehydrogenase [EC:1.1.99.1]
KO:K00119	0.0037	0.0023	-1.00E-04	
KO:K00121	0.0105	0.0392	-2.00E-04	S-(hydroxymethyl)glutathione dehydrogenase / alcohol dehydrogenase
KO:K00122	0.0148	0.011	-3.00E-04	formate dehydrogenase [EC:1.2.1.2]
KO:K00123	0.0177	0.0162	-0.0119	formate dehydrogenase, alpha subunit [EC:1.2.1.2]
KO:K00124	0.0037	7.00E-04	-0.0035	formate dehydrogenase, beta subunit [EC:1.2.1.2]
KO:K00127	0.0277	0.0174	-0.0012	formate dehydrogenase, gamma subunit [EC:1.2.1.2]
KO:K00128	0.0136	0.0059	-6.00E-04	aldehyde dehydrogenase (NAD+) [EC:1.2.1.3]
KO:K00130	0.0123	0.0062	-2.00E-04	betaine-aldehyde dehydrogenase [EC:1.2.1.8]
KO:K00133	0.0151	0.0174	4.00E-04	aspartate-semialdehyde dehydrogenase [EC:1.2.1.11]
KO:K00157	0.0141	0.0082	-1.00E-04	aldehyde oxidase [EC:1.2.3.1]
KO:K00162	0.0087	0.0014	-3.00E-04	pyruvate dehydrogenase E1 component subunit beta [EC:1.2.4.1]
KO:K00164	0.0085	0.0115	-9.00E-04	2-oxoglutarate dehydrogenase E1 component [EC:1.2.4.2]
KO:K00183	0.0448	0.0377	-2.00E-04	molybdopterin oxidoreductase, molybdopterin binding subunit
KO:K00207	0.008	3.00E-04	-2.00E-04	dihydropyrimidine dehydrogenase (NADP+) [EC:1.3.1.2]
KO:K00219	0.0135	0.0081	-3.00E-04	2,4-dienoyl-CoA reductase (NADPH2) [EC:1.3.1.34]
KO:K00226	0.0333	0.0154	-1.00E-04	dihydroorotate oxidase [EC:1.3.3.1]
KO:K00228	0.0316	0.0217	-1.00E-04	coproporphyrinogen III oxidase [EC:1.3.3.3]
KO:K00241	0.0079	0.001	-3.00E-04	succinate dehydrogenase cytochrome b-556 subunit [EC:1.3.99.1]
KO:K00242	0.0477	0.0472	-2.00E-04	succinate dehydrogenase hydrophobic membrane anchor protein
KO:K00245	0.0165	0.0126	0	fumarate reductase iron-sulfur protein [EC:1.3.99.1]
KO:K00246	0.045	0.0466	0	fumarate reductase subunit C [EC:1.3.99.1]
KO:K00252	0.042	0.0333	1.00E-04	glutaryl-CoA dehydrogenase [EC:1.3.99.7]
KO:K00253	0.0043	0.0065	4.00E-04	isovaleryl-CoA dehydrogenase [EC:1.3.99.10]
KO:K00259	0.0069	0.0039	-0.001	alanine dehydrogenase [EC:1.4.1.1]
KO:K00262	0.0064	0.0019	-1.00E-04	glutamate dehydrogenase (NADP+) [EC:1.4.1.4]
KO:K00263	0.0085	0.0084	0	leucine dehydrogenase [EC:1.4.1.9]
KO:K00275	0.0041	0.0108	1.00E-04	pyridoxamine 5'-phosphate oxidase [EC:1.4.3.5]
KO:K00285	0.0032	0.0211	-1.00E-04	D-amino-acid dehydrogenase [EC:1.4.99.1]
KO:K00286	0.02	0.0163	-1.00E-04	pyrroline-5-carboxylate reductase [EC:1.5.1.2]
KO:K00311	0.0213	0.0112	2.00E-04	electron-transferring-flavoprotein dehydrogenase [EC:1.5.5.1]
KO:K00314	0.0466	0.03	-1.00E-04	sarcosine dehydrogenase [EC:1.5.99.1]
KO:K00315	0.0168	0.0036	-0.0016	dimethylglycine dehydrogenase [EC:1.5.99.2]
KO:K00324	0.0044	1.00E-04	0.0018	NAD(P) transhydrogenase subunit alpha [EC:1.6.1.2]
KO:K00325	0.0044	0.0031	0.0016	NAD(P) transhydrogenase subunit beta [EC:1.6.1.2]
KO:K00331	0.0215	0.0126	2.00E-04	NADH dehydrogenase I subunit B [EC:1.6.5.3]
KO:K00342	0.034	0.0487	3.00E-04	NADH dehydrogenase I subunit M [EC:1.6.5.3]
KO:K00383	0.0038	0.003	2.00E-04	glutathione reductase (NADPH) [EC:1.8.1.7]
KO:K00386	0.004	0.011	-0.0016	
KO:K00411	0.0038	2.00E-04	6.00E-04	ubiquinol-cytochrome c reductase iron-sulfur subunit [EC:1.10.2.2]

KO:K00412	0.0035	0	0.0029	ubiquinol-cytochrome c reductase cytochrome b subunit [EC:1.10.2.2]
KO:K00413	0.003	4.00E-04	7.00E-04	ubiquinol-cytochrome c reductase cytochrome c1 subunit [EC:1.10.2.2]
KO:K00432	0.0041	8.00E-04	3.00E-04	glutathione peroxidase [EC:1.11.1.9]
KO:K00450	0.0075	0.0123	0	gentisate 1,2-dioxygenase [EC:1.13.11.4]
KO:K00451	0.0047	0.0126	0	homogentisate 1,2-dioxygenase [EC:1.13.11.5]
KO:K00457	0.0159	0.0219	-2.00E-04	4-hydroxyphenylpyruvate dioxygenase [EC:1.13.11.27]
KO:K00459	0.0496	0.0523	0	nitronate monooxygenase [EC:1.13.12.16]
KO:K00462	0.0037	0.0024	0	biphenyl-2,3-diol 1,2-dioxygenase [EC:1.13.11.39]
KO:K00471	0.0319	0.0286	0	gamma-butyrobetaine dioxygenase [EC:1.14.11.1]
KO:K00480	0.0189	0.0395	1.00E-04	salicylate hydroxylase [EC:1.14.13.1]
KO:K00485	0.0268	0.0295	-1.00E-04	dimethylaniline monooxygenase (N-oxide forming) [EC:1.14.13.8]
KO:K00517	0.0197	0.0507	-1.00E-04	
KO:K00518	0.0151	0.0122	1.00E-04	superoxide dismutase [EC:1.15.1.1]
KO:K00520	0.0085	0.0216	2.00E-04	mercuric reductase [EC:1.16.1.1]
KO:K00525	0.0134	0.0218	9.00E-04	ribonucleoside-diphosphate reductase alpha chain [EC:1.17.4.1]
KO:K00528	0.0162	0.0277	-2.00E-04	ferredoxin--NADP+ reductase [EC:1.18.1.2]
KO:K00540	0.0119	0.0156	-3.00E-04	
KO:K00548	0.014	0.0089	-5.00E-04	5-methyltetrahydrofolate--homocysteine methyltransferase
KO:K00568	0.0124	0.0053	1.00E-04	3-demethylubiquinone-9 3-methyltransferase [EC:2.1.1.- 2.1.1.64]
KO:K00571	0.0035	0.002	-1.00E-04	site-specific DNA-methyltransferase (adenine-specific) [EC:2.1.1.72]
KO:K00599	0.0086	0.0045	-0.0011	
KO:K00600	0.0037	0.0025	7.00E-04	glycine hydroxymethyltransferase [EC:2.1.2.1]
KO:K00602	0.0033	7.00E-04	3.00E-04	phosphoribosylaminoimidazolecarboxamide formyltransferase / IMP
KO:K00625	0.0204	0.0222	-1.00E-04	phosphate acetyltransferase [EC:2.3.1.8]
KO:K00627	0.0083	0.0044	-2.00E-04	pyruvate dehydrogenase E2 component (dihydrolipoamide
KO:K00643	0.0035	4.00E-04	-0.0015	5-aminolevulinate synthase [EC:2.3.1.37]
KO:K00648	0.0042	0.0061	-1.00E-04	3-oxoacyl-[acyl-carrier-protein] synthase III [EC:2.3.1.180]
KO:K00651	0.0271	0.0203	1.00E-04	homoserine O-succinyltransferase [EC:2.3.1.46]
KO:K00666	0.0247	0.0096	-3.00E-04	fatty-acyl-CoA synthase [EC:6.2.1.-]
KO:K00674	0.0294	0.0243	2.00E-04	2,3,4,5-tetrahydropyridine-2-carboxylate N-succinyltransferase
KO:K00680	0.0272	0.0245	-1.00E-04	
KO:K00758	0.0037	0.0035	-1.00E-04	thymidine phosphorylase [EC:2.4.2.4]
KO:K00761	0.0397	0.0519	-1.00E-04	uracil phosphoribosyltransferase [EC:2.4.2.9]
KO:K00767	0.0285	0.0472	0	nicotinate-nucleotide pyrophosphorylase (carboxylating)
KO:K00795	0.003	0.0241	-3.00E-04	farnesyl diphosphate synthase [EC:2.5.1.1 2.5.1.10]
KO:K00798	0.0044	0	3.00E-04	cob(I)alamin adenosyltransferase [EC:2.5.1.17]
KO:K00804	0.0039	0.0021	-9.00E-04	geranylgeranyl diphosphate synthase, type III [EC:2.5.1.1 2.5.1.10
KO:K00819	0.0164	0.0368	0	ornithine--oxo-acid transaminase [EC:2.6.1.13]
KO:K00821	0.0162	0.0119	-1.00E-04	acetylornithine/N-succinyldiaminopimelate aminotransferase
KO:K00822	0.0033	0.0034	-2.00E-04	beta-alanine--pyruvate transaminase [EC:2.6.1.18]
KO:K00824	0.0076	0.0215	2.00E-04	D-alanine transaminase [EC:2.6.1.21]
KO:K00831	0.0259	0.0072	1.00E-04	phosphoserine aminotransferase [EC:2.6.1.52]
KO:K00837	0.011	0.0066	-4.00E-04	
KO:K00839	0.0064	0.0061	4.00E-04	aminotransferase [EC:2.6.1.-]
KO:K00845	0.027	0.0269	0	glucokinase [EC:2.7.1.2]
KO:K00850	0.0034	0.0296	-1.00E-04	6-phosphofructokinase [EC:2.7.1.11]
KO:K00858	0.0125	0.0078	-1.00E-04	NAD+ kinase [EC:2.7.1.23]
KO:K00864	0.0244	0.0279	-1.00E-04	glycerol kinase [EC:2.7.1.30]
KO:K00891	0.0268	0.0382	0	shikimate kinase [EC:2.7.1.71]
KO:K00919	0.024	0.018	0	4-diphosphocytidyl-2-C-methyl-D-erythritol kinase [EC:2.7.1.148]
KO:K00928	0.0152	0.0085	1.00E-04	aspartate kinase [EC:2.7.2.4]
KO:K00941	0.0105	0.0077	0	phosphomethylpyrimidine kinase [EC:2.7.4.7]
KO:K00945	0.0288	0.0359	-1.00E-04	cytidylate kinase [EC:2.7.4.14]
KO:K00954	0.0157	0.0088	-1.00E-04	pantetheine-phosphate adenylyltransferase [EC:2.7.7.3]
KO:K00962	0.0095	0.0066	0.0018	polyribonucleotide nucleotidyltransferase [EC:2.7.7.8]
KO:K00970	0.0469	0.0299	0	poly(A) polymerase [EC:2.7.7.19]
KO:K00997	0.0116	0.0031	1.00E-04	holo-[acyl-carrier protein] synthase [EC:2.7.8.7]
KO:K01005	0.0041	0.0059	0	
KO:K01006	0.0161	0.0099	-3.00E-04	pyruvate,orthophosphate dikinase [EC:2.7.9.1]
KO:K01007	0.0389	0.0336	-1.00E-04	pyruvate, water dikinase [EC:2.7.9.2]
KO:K01055	0.0034	0.0074	-1.00E-04	3-oxoadipate enol-lactonase [EC:3.1.1.24]
KO:K01056	0.0244	0.0631	1.00E-04	peptidyl-tRNA hydrolase, PTH1 family [EC:3.1.1.29]
KO:K01061	0.036	0.0213	0	carboxymethylenebutenolidase [EC:3.1.1.45]

KO:K01069	0.0076	0.0247	-1.00E-04	hydroxyacylglutathione hydrolase [EC:3.1.2.6]
KO:K01079	0.0346	0.037	0	phosphoserine phosphatase [EC:3.1.3.3]
KO:K01081	0.0189	0.0319	-1.00E-04	5'-nucleotidase [EC:3.1.3.5]
KO:K01092	0.0044	0.0022	2.00E-04	myo-inositol-1(or 4)-monophosphatase [EC:3.1.3.25]
KO:K01118	0.0044	0.0102	3.00E-04	FMN-dependent NADH-azoreductase [EC:1.7.-.-]
KO:K01128	0.0114	0.005	1.00E-04	
KO:K01133	0.0261	0.0194	-1.00E-04	choline-sulfatase [EC:3.1.6.6]
KO:K01138	0.0266	0.0324	-1.00E-04	
KO:K01139	0.0355	0.0306	1.00E-04	guanosine-3',5'-bis(diphosphate) 3'-pyrophosphohydrolase
KO:K01141	0.0446	0.0608	0	exodeoxyribonuclease I [EC:3.1.11.1]
KO:K01153	0.004	0.0073	0	type I restriction enzyme, R subunit [EC:3.1.21.3]
KO:K01159	0.0036	0.0036	-1.00E-04	crossover junction endodeoxyribonuclease RuvC [EC:3.1.22.4]
KO:K01209	0.0453	0.0643	0	alpha-N-arabinofuranosidase [EC:3.2.1.55]
KO:K01251	0.0031	9.00E-04	5.00E-04	adenosylhomocysteinase [EC:3.3.1.1]
KO:K01271	0.0119	0.0067	-1.00E-04	X-Pro dipeptidase [EC:3.4.13.9]
KO:K01274	0.0076	0.0026	0	D-alanyl-D-alanine dipeptidase [EC:3.4.13.-]
KO:K01284	0.0261	0.0226	1.00E-04	peptidyl-dipeptidase Dcp [EC:3.4.15.5]
KO:K01301	0.0098	0.0114	0	glutamate carboxypeptidase II [EC:3.4.17.21]
KO:K01322	0.0183	0.0523	0	prolyl oligopeptidase [EC:3.4.21.26]
KO:K01356	0.0304	0.0243	-1.00E-04	repressor LexA [EC:3.4.21.88]
KO:K01362	0.0036	0.0014	0.0013	
KO:K01412	0.0028	0.0119	1.00E-04	mitochondrial processing peptidase [EC:3.4.24.64]
KO:K01419	0.048	0.0443	1.00E-04	ATP-dependent HslUV protease, peptidase subunit HslV [EC:3.4.25.-]
KO:K01422	0.0038	1.00E-04	4.00E-04	
KO:K01426	0.015	0.0132	-1.00E-04	amidase [EC:3.5.1.4]
KO:K01429	0.0422	0.04	0	urease subunit beta [EC:3.5.1.5]
KO:K01431	0.0156	0.0156	-1.00E-04	beta-ureidopropionase [EC:3.5.1.6]
KO:K01436	0.0257	0.0302	0	aminoacylase [EC:3.5.1.14]
KO:K01438	0.0154	0.0133	-1.00E-04	acetylornithine deacetylase [EC:3.5.1.16]
KO:K01464	0.0166	0.0061	-4.00E-04	dihydropyrimidinase [EC:3.5.2.2]
KO:K01465	0.013	0.0046	-1.00E-04	dihydroorotase [EC:3.5.2.3]
KO:K01468	0.0046	0.0029	-1.00E-04	imidazolonepropionase [EC:3.5.2.7]
KO:K01473	0.0124	0.0151	-2.00E-04	N-methylhydantoinase A [EC:3.5.2.14]
KO:K01474	0.0231	0.0078	-3.00E-04	N-methylhydantoinase B [EC:3.5.2.14]
KO:K01480	0.0041	3.00E-04	-2.00E-04	agmatinase [EC:3.5.3.11]
KO:K01484	0.0451	0.047	0	succinylarginine dihydrolase [EC:3.5.3.23]
KO:K01486	0.0069	0.007	-1.00E-04	adenine deaminase [EC:3.5.4.2]
KO:K01495	0.0152	0.0088	1.00E-04	GTP cyclohydrolase I [EC:3.5.4.16]
KO:K01506	0.036	0.0455	-1.00E-04	
KO:K01514	0.0043	0.0015	-2.00E-04	exopolyphosphatase [EC:3.6.1.11]
KO:K01556	0.0404	0.0203	0	kynureninase [EC:3.7.1.3]
KO:K01561	0.0187	0.0699	-1.00E-04	haloacetate dehalogenase [EC:3.8.1.3]
KO:K01571	0.0203	0.0172	0	oxaloacetate decarboxylase, alpha subunit [EC:4.1.1.3]
KO:K01579	0.0416	0.0269	0	aspartate 1-decarboxylase [EC:4.1.1.11]
KO:K01589	0.0127	0.0084	1.00E-04	5-(carboxyamino)imidazole ribonucleotide synthase [EC:6.3.4.18]
KO:K01595	0.016	0.073	3.00E-04	phosphoenolpyruvate carboxylase [EC:4.1.1.31]
KO:K01599	0.0033	4.00E-04	-4.00E-04	uroporphyrinogen decarboxylase [EC:4.1.1.37]
KO:K01610	0.0044	0.0062	7.00E-04	phosphoenolpyruvate carboxykinase (ATP) [EC:4.1.1.49]
KO:K01624	0.0033	0.0015	-1.00E-04	fructose-bisphosphate aldolase, class II [EC:4.1.2.13]
KO:K01640	0.0081	0.003	-1.00E-04	hydroxymethylglutaryl-CoA lyase [EC:4.1.3.4]
KO:K01644	0.0042	0.0014	3.00E-04	citrate lyase subunit beta / citryl-CoA lyase [EC:4.1.3.6 4.1.3.34]
KO:K01647	0.0144	0.011	-4.00E-04	citrate synthase [EC:2.3.3.1]
KO:K01662	0.0037	0	-0.0017	1-deoxy-D-xylulose-5-phosphate synthase [EC:2.2.1.7]
KO:K01669	0.0034	0.0016	9.00E-04	deoxyribodipyrimidine photo-lyase [EC:4.1.99.3]
KO:K01676	0.0028	0.0834	0	fumarate hydratase, class I [EC:4.2.1.2]
KO:K01679	0.0238	0.0168	0	fumarate hydratase, class II [EC:4.2.1.2]
KO:K01681	0.0088	0.0048	-4.00E-04	aconitate hydratase 1 [EC:4.2.1.3]
KO:K01682	0.0085	0.003	-4.00E-04	aconitate hydratase 2 [EC:4.2.1.3]
KO:K01695	0.0072	0.0499	1.00E-04	tryptophan synthase alpha chain [EC:4.2.1.20]
KO:K01696	0.0129	0.0058	2.00E-04	tryptophan synthase beta chain [EC:4.2.1.20]
KO:K01697	0.0093	0.0083	-1.00E-04	cystathionine beta-synthase [EC:4.2.1.22]
KO:K01708	0.0262	0.0294	-1.00E-04	galactarate dehydratase [EC:4.2.1.42]
KO:K01711	0.0183	0.0158	-1.00E-04	GDPmannose 4,6-dehydratase [EC:4.2.1.47]

KO:K01721	0.0031	8.00E-04	-2.00E-04	nitrile hydratase [EC:4.2.1.84]
KO:K01733	0.0108	0.0091	1.00E-04	threonine synthase [EC:4.2.3.1]
KO:K01749	0.0038	1.00E-04	-3.00E-04	hydroxymethylbilane synthase [EC:2.5.1.61]
KO:K01752	0.018	0.014	-1.00E-04	L-serine dehydratase [EC:4.3.1.17]
KO:K01759	0.0044	0.0058	-2.00E-04	lactoylglutathione lyase [EC:4.4.1.5]
KO:K01761	0.0038	0.0107	0	methionine-gamma-lyase [EC:4.4.1.11]
KO:K01772	0.0032	3.00E-04	2.00E-04	ferrochelatase [EC:4.99.1.1]
KO:K01777	0.025	0.0205	0	proline racemase [EC:5.1.1.4]
KO:K01781	0.0367	0.0428	0	mandelate racemase [EC:5.1.2.2]
KO:K01790	0.0036	0.0035	0	dTDP-4-dehydrorhamnose 3,5-epimerase [EC:5.1.3.13]
KO:K01791	0.0307	0.0213	0	UDP-N-acetylglucosamine 2-epimerase [EC:5.1.3.14]
KO:K01810	0.0369	0.0437	-1.00E-04	glucose-6-phosphate isomerase [EC:5.3.1.9]
KO:K01816	0.0032	1.00E-04	-1.00E-04	hydroxypyruvate isomerase [EC:5.3.1.22]
KO:K01823	0.0043	0.0018	-2.00E-04	isopentenyl-diphosphate delta-isomerase [EC:5.3.3.2]
KO:K01826	0.0445	0.0411	0	5-carboxymethyl-2-hydroxymuconate isomerase [EC:5.3.3.10]
KO:K01840	0.0237	0.0289	0	phosphomannomutase [EC:5.4.2.8]
KO:K01845	0.0129	0.0069	-2.00E-04	glutamate-1-semialdehyde 2,1-aminomutase [EC:5.4.3.8]
KO:K01866	0.0044	4.00E-04	1.00E-04	tyrosyl-tRNA synthetase [EC:6.1.1.1]
KO:K01873	0.0065	0.0048	1.00E-04	valyl-tRNA synthetase [EC:6.1.1.9]
KO:K01876	0.0032	2.00E-04	4.00E-04	aspartyl-tRNA synthetase [EC:6.1.1.12]
KO:K01878	0.0042	0.0011	1.00E-04	glycyl-tRNA synthetase alpha chain [EC:6.1.1.14]
KO:K01880	0.0335	0.029	0	glycyl-tRNA synthetase [EC:6.1.1.14]
KO:K01887	0.0402	0.0339	2.00E-04	arginyl-tRNA synthetase [EC:6.1.1.19]
KO:K01889	0.0114	0.0075	1.00E-04	phenylalanyl-tRNA synthetase alpha chain [EC:6.1.1.20]
KO:K01903	0.0073	0.002	-8.00E-04	succinyl-CoA synthetase beta subunit [EC:6.2.1.5]
KO:K01916	0.0221	0.0089	1.00E-04	NAD+ synthase [EC:6.3.1.5]
KO:K01919	0.0102	0.0077	1.00E-04	glutamate--cysteine ligase [EC:6.3.2.2]
KO:K01921	0.0339	0.0234	-3.00E-04	D-alanine-D-alanine ligase [EC:6.3.2.4]
KO:K01922	0.0251	0.0448	1.00E-04	phosphopantothenate-cysteine ligase [EC:6.3.2.5]
KO:K01923	0.0334	0.0257	2.00E-04	phosphoribosylaminoimidazole-succinocarboxamide synthase
KO:K01928	0.0283	0.0603	-1.00E-04	UDP-N-acetylmuramoylalanyl-D-glutamate--2,6-diaminopimelate ligase
KO:K01933	0.0299	0.0186	2.00E-04	phosphoribosylformylglycinamidine cyclo-ligase [EC:6.3.3.1]
KO:K01939	0.0081	0.0096	4.00E-04	adenylosuccinate synthase [EC:6.3.4.4]
KO:K01940	0.0089	0.0078	3.00E-04	argininosuccinate synthase [EC:6.3.4.5]
KO:K01950	0.0035	0.0022	1.00E-04	NAD+ synthase (glutamine-hydrolysing) [EC:6.3.5.1]
KO:K01952	0.0165	0.0121	4.00E-04	phosphoribosylformylglycinamidine synthase [EC:6.3.5.3]
KO:K01955	0.0038	0.0074	3.00E-04	carbamoyl-phosphate synthase large subunit [EC:6.3.5.5]
KO:K01959	0.0419	0.0305	0	pyruvate carboxylase subunit A [EC:6.4.1.1]
KO:K01963	0.0036	0.0069	-1.00E-04	acetyl-CoA carboxylase carboxyl transferase subunit beta
KO:K01971	0.0221	0.0264	0	DNA ligase (ATP) [EC:6.5.1.1]
KO:K01999	0.0039	0.004	-0.0043	branched-chain amino acid transport system substrate-binding protein
KO:K02000	0.0275	0.0142	-3.00E-04	glycine betaine/proline transport system ATP-binding protein
KO:K02001	0.0125	0.0108	-6.00E-04	glycine betaine/proline transport system permease protein
KO:K02002	0.0042	3.00E-04	-0.0027	glycine betaine/proline transport system substrate-binding protein
KO:K02013	0.0284	0.0153	0	iron complex transport system ATP-binding protein [EC:3.6.3.34]
KO:K02014	0.0323	0.0282	2.00E-04	iron complex outermembrane recepter protein
KO:K02027	0.016	0.0051	-0.0037	multiple sugar transport system substrate-binding protein
KO:K02028	0.0473	0.064	-3.00E-04	polar amino acid transport system ATP-binding protein [EC:3.6.3.21]
KO:K02029	0.0068	0.0042	-5.00E-04	polar amino acid transport system permease protein
KO:K02031	0.0141	0.0069	-2.00E-04	peptide/nickel transport system ATP-binding protein
KO:K02032	0.0482	0.0375	-6.00E-04	peptide/nickel transport system ATP-binding protein
KO:K02033	0.0132	0.0052	-0.0016	peptide/nickel transport system permease protein
KO:K02034	0.0401	0.0337	-8.00E-04	peptide/nickel transport system permease protein
KO:K02035	0.0045	1.00E-04	-0.0078	peptide/nickel transport system substrate-binding protein
KO:K02036	0.0188	0.0197	1.00E-04	phosphate transport system ATP-binding protein [EC:3.6.3.27]
KO:K02037	0.0083	0.0028	2.00E-04	phosphate transport system permease protein
KO:K02039	0.0078	0.0119	0	phosphate transport system protein
KO:K02040	0.0364	0.0467	2.00E-04	phosphate transport system substrate-binding protein
KO:K02041	0.0041	0.008	-2.00E-04	phosphonate transport system ATP-binding protein
KO:K02042	0.0225	0.0217	-1.00E-04	phosphonate transport system permease protein
KO:K02044	0.0043	0.0014	-2.00E-04	phosphonate transport system substrate-binding protein
KO:K02049	0.0069	0.0021	-3.00E-04	sulfonate/nitrate/taurine transport system ATP-binding protein
KO:K02050	0.0358	0.0395	-5.00E-04	sulfonate/nitrate/taurine transport system permease protein

KO:K02051	0.0034	0.0052	-0.0016	sulfonate/nitrate/taurine transport system substrate-binding protein
KO:K02052	0.0029	9.00E-04	-4.00E-04	putative spermidine/putrescine transport system ATP-binding protein
KO:K02053	0.0377	0.0302	-3.00E-04	putative spermidine/putrescine transport system permease protein
KO:K02054	0.0104	0.004	-5.00E-04	putative spermidine/putrescine transport system permease protein
KO:K02056	0.0112	0.0245	-5.00E-04	simple sugar transport system ATP-binding protein [EC:3.6.3.17]
KO:K02057	0.0417	0.0283	-9.00E-04	simple sugar transport system permease protein
KO:K02058	0.0045	0.0076	-0.0048	simple sugar transport system substrate-binding protein
KO:K02064	0.0225	0.0141	1.00E-04	thiamine transport system substrate-binding protein
KO:K02066	0.0113	0.0133	-1.00E-04	putative ABC transport system permease protein
KO:K02108	0.0038	0.0071	0.0012	F-type H+-transporting ATPase subunit a [EC:3.6.3.14]
KO:K02109	0.0037	0.0067	9.00E-04	F-type H+-transporting ATPase subunit b [EC:3.6.3.14]
KO:K02112	0.0165	0.0146	0.0011	F-type H+-transporting ATPase subunit beta [EC:3.6.3.14]
KO:K02113	0.0042	7.00E-04	8.00E-04	F-type H+-transporting ATPase subunit delta [EC:3.6.3.14]
KO:K02114	0.0133	0.0141	3.00E-04	F-type H+-transporting ATPase subunit epsilon [EC:3.6.3.14]
KO:K02197	0.0248	0.0181	0	cytochrome c-type biogenesis protein CcmE
KO:K02198	0.0104	0.0028	4.00E-04	cytochrome c-type biogenesis protein CcmF
KO:K02259	0.0047	0.0012	3.00E-04	cytochrome c oxidase subunit XV assembly protein
KO:K02274	0.0028	3.00E-04	0.0021	cytochrome c oxidase subunit I [EC:1.9.3.1]
KO:K02275	0.0051	0	0.0016	cytochrome c oxidase subunit II [EC:1.9.3.1]
KO:K02291	0.0047	1.00E-04	-4.00E-04	phytoene synthase [EC:2.5.1.32]
KO:K02301	0.003	2.00E-04	7.00E-04	protoheme IX farnesyltransferase [EC:2.5.1.-]
KO:K02314	0.0105	0.0058	-1.00E-04	replicative DNA helicase [EC:3.6.1.-]
KO:K02338	0.0039	0.0041	-1.00E-04	DNA polymerase III subunit beta [EC:2.7.7.7]
KO:K02355	0.0103	0.0196	0.004	elongation factor EF-G [EC:3.6.5.3]
KO:K02357	0.0046	2.00E-04	9.00E-04	elongation factor EF-Ts
KO:K02358	0.0089	0.0054	0.0035	elongation factor EF-Tu [EC:3.6.5.3]
KO:K02388	0.0465	0.034	-1.00E-04	flagellar basal-body rod protein FlgC
KO:K02392	0.0463	0.0503	-2.00E-04	flagellar basal-body rod protein FlgG
KO:K02400	0.0087	0.0481	-2.00E-04	flagellar biosynthesis protein FlhA
KO:K02437	0.0119	0.0077	-1.00E-04	glycine cleavage system H protein
KO:K02440	0.0482	0.0225	0	glycerol uptake facilitator protein
KO:K02469	0.0225	0.0132	-3.00E-04	DNA gyrase subunit A [EC:5.99.1.3]
KO:K02470	0.01	0.0089	-3.00E-04	DNA gyrase subunit B [EC:5.99.1.3]
KO:K02498	0.0043	6.00E-04	-1.00E-04	HemY protein
KO:K02502	0.0492	0.0355	1.00E-04	ATP phosphoribosyltransferase regulatory subunit
KO:K02510	0.0254	0.0177	-1.00E-04	2,4-dihydroxyhept-2-ene-1,7-dioic acid aldolase [EC:4.1.2.-]
KO:K02517	0.0434	0.0326	1.00E-04	lipid A biosynthesis lauroyl acyltransferase [EC:2.3.1.-]
KO:K02529	0.0376	0.0826	-1.00E-04	LacI family transcriptional regulator
KO:K02535	0.0082	0.0045	-3.00E-04	UDP-3-O-[3-hydroxymyristoyl] N-acetylglucosamine deacetylase
KO:K02556	0.0084	4.00E-04	-4.00E-04	chemotaxis protein MotA
KO:K02557	0.0356	0.0282	-1.00E-04	chemotaxis protein MotB
KO:K02600	0.0128	0.0211	7.00E-04	N utilization substance protein A
KO:K02601	0.0036	0.0025	9.00E-04	transcriptional antiterminator NusG
KO:K02618	0.0216	0.0492	-1.00E-04	phenylacetic acid degradation protein
KO:K02622	0.0342	0.0265	-1.00E-04	topoisomerase IV subunit B [EC:5.99.1.-]
KO:K02806	0.0174	0.0101	2.00E-04	PTS system, nitrogen regulatory IIA component [EC:2.7.1.69]
KO:K02825	0.0103	0.0286	0	pyrimidine operon attenuation protein / uracil
KO:K02863	0.0047	0.0102	0.0011	large subunit ribosomal protein L1
KO:K02864	0.0267	0.0186	0.0069	large subunit ribosomal protein L10
KO:K02867	0.0038	0.0147	3.00E-04	large subunit ribosomal protein L11
KO:K02874	0.004	5.00E-04	6.00E-04	large subunit ribosomal protein L14
KO:K02876	0.0292	0.0162	0.0012	large subunit ribosomal protein L15
KO:K02878	0.0045	9.00E-04	7.00E-04	large subunit ribosomal protein L16
KO:K02879	0.008	0.0115	3.00E-04	large subunit ribosomal protein L17
KO:K02881	0.0043	0.0028	6.00E-04	large subunit ribosomal protein L18
KO:K02886	0.035	0.0298	0.0034	large subunit ribosomal protein L2
KO:K02890	0.0035	3.00E-04	0.001	large subunit ribosomal protein L22
KO:K02892	0.0047	0.0051	6.00E-04	large subunit ribosomal protein L23
KO:K02895	0.0041	3.00E-04	6.00E-04	large subunit ribosomal protein L24
KO:K02906	0.0033	0.0026	0.0013	large subunit ribosomal protein L3
KO:K02907	0.0064	0.0213	4.00E-04	large subunit ribosomal protein L30
KO:K02926	0.0038	0.0017	0.0011	large subunit ribosomal protein L4
KO:K02931	0.0034	0.0055	0.0011	large subunit ribosomal protein L5

KO:K02933	0.0032	0.004	0.001	large subunit ribosomal protein L6
KO:K02935	0.0193	0.0132	0.0062	large subunit ribosomal protein L7/L12
KO:K02939	0.0459	0.0371	0.001	large subunit ribosomal protein L9
KO:K02945	0.0251	0.0388	0.005	small subunit ribosomal protein S1
KO:K02946	0.0034	0.0084	5.00E-04	small subunit ribosomal protein S10
KO:K02948	0.003	0.0069	0.0017	small subunit ribosomal protein S11
KO:K02952	0.0244	0.0289	0.0012	small subunit ribosomal protein S13
KO:K02954	0.003	0.0033	4.00E-04	small subunit ribosomal protein S14
KO:K02961	0.0172	0.0215	7.00E-04	small subunit ribosomal protein S17
KO:K02965	0.0033	0.0021	9.00E-04	small subunit ribosomal protein S19
KO:K02967	0.0156	0.0043	9.00E-04	small subunit ribosomal protein S2
KO:K02982	0.0037	2.00E-04	0.002	small subunit ribosomal protein S3
KO:K02986	0.0367	0.0373	0.0011	small subunit ribosomal protein S4
KO:K02988	0.0037	0.0055	9.00E-04	small subunit ribosomal protein S5
KO:K02992	0.0028	0.0019	0.0015	small subunit ribosomal protein S7
KO:K02994	0.0095	0.0159	8.00E-04	small subunit ribosomal protein S8
KO:K02996	0.0131	0.0128	9.00E-04	small subunit ribosomal protein S9
KO:K03040	0.0043	0	0.0021	DNA-directed RNA polymerase subunit alpha [EC:2.7.7.6]
KO:K03043	0.0204	0.0195	0.0034	DNA-directed RNA polymerase subunit beta [EC:2.7.7.6]
KO:K03046	0.0149	0.0117	0.0037	DNA-directed RNA polymerase subunit beta' [EC:2.7.7.6]
KO:K03070	0.0031	0.0049	3.00E-04	preprotein translocase subunit SecA
KO:K03073	0.0039	0.0044	9.00E-04	preprotein translocase subunit SecE
KO:K03076	0.0026	3.00E-04	0.0017	preprotein translocase subunit SecY
KO:K03088	0.0049	0.0069	0.0013	RNA polymerase sigma-70 factor, ECF subfamily
KO:K03089	0.0049	0.001	0.005	RNA polymerase sigma-32 factor
KO:K03106	0.0105	0.0035	2.00E-04	signal recognition particle subunit SRP54
KO:K03168	0.0038	0.0015	3.00E-04	DNA topoisomerase I [EC:5.99.1.2]
KO:K03182	0.0189	0.014	-1.00E-04	3-octaprenyl-4-hydroxybenzoate carboxy-lyase UbiD [EC:4.1.1.-]
KO:K03185	0.0074	0.0021	2.00E-04	2-octaprenyl-6-methoxyphenol hydroxylase [EC:1.14.13.-]
KO:K03186	0.0311	0.0565	0	3-octaprenyl-4-hydroxybenzoate carboxy-lyase UbiX [EC:4.1.1.-]
KO:K03192	0.0303	0.0434	-1.00E-04	urease accessory protein
KO:K03217	0.0037	0	3.00E-04	preprotein translocase subunit YidC
KO:K03284	0.0367	0.0261	0	metal ion transporter, MIT family
KO:K03286	0.02	0.0117	0	OmpA-OmpF porin, OOP family
KO:K03287	0.0114	0.017	1.00E-04	outer membrane factor, OMF family
KO:K03292	0.0033	1.00E-04	2.00E-04	glycoside/pentoside/hexuronide:cation symporter, GPH family
KO:K03307	0.0079	0.0271	-5.00E-04	solute:Na+ symporter, SSS family
KO:K03310	0.0113	0.006	-2.00E-04	alanine or glycine:cation symporter, AGCS family
KO:K03313	0.0241	0.0408	4.00E-04	Na+:H+ antiporter, NhaA family
KO:K03315	0.0043	1.00E-04	-1.00E-04	Na+:H+ antiporter, NhaC family
KO:K03336	0.0117	0.0032	-2.00E-04	3D-(3,5/4)-trihydroxycyclohexane-1,2-dione hydrolase [EC:3.7.1.-]
KO:K03337	0.0197	0.014	-1.00E-04	5-deoxy-glucuronate isomerase [EC:5.3.1.-]
KO:K03338	0.0097	0.0043	-1.00E-04	5-dehydro-2-deoxygluconokinase [EC:2.7.1.92]
KO:K03366	0.0085	0.012	-1.00E-04	(R,R)-butanediol dehydrogenase / diacetyl reductase [EC:1.1.1.4
KO:K03379	0.0238	0.0191	0	cyclohexanone monooxygenase [EC:1.14.13.22]
KO:K03394	0.0152	0.0205	0	precorrin-2 C20-methyltransferase / cobalt-factor-2
KO:K03403	0.0032	0	-0.0028	magnesium chelatase subunit H [EC:6.6.1.1]
KO:K03404	0.0208	0.0081	0	magnesium chelatase subunit D [EC:6.6.1.1]
KO:K03405	0.0044	2.00E-04	-3.00E-04	magnesium chelatase subunit I [EC:6.6.1.1]
KO:K03426	0.0422	0.048	0	NAD+ diphosphatase [EC:3.6.1.22]
KO:K03428	0.0037	1.00E-04	-8.00E-04	magnesium-protoporphyrin O-methyltransferase [EC:2.1.1.11]
KO:K03430	0.0107	0.0479	-1.00E-04	2-aminoethylphosphonate-pyruvate transaminase [EC:2.6.1.37]
KO:K03437	0.0334	0.0411	0	RNA methyltransferase, TrmH family
KO:K03438	0.0078	0.0057	-1.00E-04	S-adenosyl-methyltransferase [EC:2.1.1.-]
KO:K03442	0.0492	0.0671	-1.00E-04	small conductance mechanosensitive channel
KO:K03451	0.0219	0.0285	0	betaine/carnitine transporter, BCCT family
KO:K03469	0.0087	0.0052	0	ribonuclease HI [EC:3.1.26.4]
KO:K03474	0.022	0.0119	1.00E-04	pyridoxine 5-phosphate synthase [EC:2.6.99.2]
KO:K03499	0.0277	0.0167	-1.00E-04	trk system potassium uptake protein TrkA
KO:K03503	0.0075	0.006	0	DNA polymerase V [EC:3.4.21.-]
KO:K03520	0.0152	0.0348	0.0016	carbon-monoxide dehydrogenase large subunit [EC:1.2.99.2]
KO:K03526	0.0072	8.00E-04	-2.00E-04	(E)-4-hydroxy-3-methylbut-2-enyl-diphosphate synthase [EC:1.17.7.1]
KO:K03527	0.0108	0.0063	-1.00E-04	4-hydroxy-3-methylbut-2-enyl diphosphate reductase [EC:1.17.1.2]

KO:K03529	0.0331	0.0554	-2.00E-04	chromosome segregation protein
KO:K03531	0.0426	0.0453	-3.00E-04	cell division protein FtsZ
KO:K03545	0.0091	0.0046	5.00E-04	trigger factor
KO:K03550	0.0411	0.0299	-1.00E-04	holliday junction DNA helicase RuvA
KO:K03551	0.0196	0.0307	-1.00E-04	holliday junction DNA helicase RuvB
KO:K03553	0.0191	0.0178	-2.00E-04	recombination protein RecA
KO:K03562	0.0477	0.0712	-1.00E-04	biopolymer transport protein TolQ
KO:K03569	0.0411	0.0429	-1.00E-04	rod shape-determining protein MreB and related proteins
KO:K03572	0.0425	0.0377	-1.00E-04	DNA mismatch repair protein MutL
KO:K03576	0.0191	0.013	4.00E-04	LysR family transcriptional regulator, regulator for metE and metH
KO:K03584	0.0165	0.0173	0	DNA repair protein RecO (recombination protein O)
KO:K03588	0.0086	0.0328	-4.00E-04	cell division protein FtsW
KO:K03593	0.0043	5.00E-04	1.00E-04	ATP-binding protein involved in chromosome partitioning
KO:K03596	0.0027	0.0014	3.00E-04	GTP-binding protein LepA
KO:K03601	0.0044	0.0021	-1.00E-04	exodeoxyribonuclease VII large subunit [EC:3.1.11.6]
KO:K03621	0.0156	0.028	2.00E-04	glycerol-3-phosphate acyltransferase PlsX [EC:2.3.1.15]
KO:K03628	0.0289	0.0227	7.00E-04	transcription termination factor Rho
KO:K03667	0.01	0.0076	1.00E-04	ATP-dependent HslUV protease ATP-binding subunit HslU
KO:K03672	0.0039	0.0013	0	thioredoxin 2 [EC:1.8.1.8]
KO:K03686	0.0081	0.0141	6.00E-04	molecular chaperone DnaJ
KO:K03687	0.0097	5.00E-04	2.00E-04	molecular chaperone GrpE
KO:K03701	0.0048	7.00E-04	4.00E-04	excinuclease ABC subunit A
KO:K03702	0.0325	0.02	1.00E-04	excinuclease ABC subunit B
KO:K03718	0.0238	0.0971	-1.00E-04	Lrp/AsnC family transcriptional regulator, regulator for asnA, asnC
KO:K03723	0.0047	2.00E-04	3.00E-04	transcription-repair coupling factor (superfamily II helicase)
KO:K03745	0.0051	0.0066	0	SlyX protein
KO:K03769	0.0154	0.0327	1.00E-04	peptidyl-prolyl cis-trans isomerase C [EC:5.2.1.8]
KO:K03770	0.0296	0.0187	2.00E-04	peptidyl-prolyl cis-trans isomerase D [EC:5.2.1.8]
KO:K03780	0.0088	0.0142	0	L(+)-tartrate dehydratase beta subunit [EC:4.2.1.32]
KO:K03782	0.0121	0.0161	7.00E-04	catalase/peroxidase [EC:1.11.1.6 1.11.1.7]
KO:K03790	0.0202	0.0176	0	ribosomal-protein-alanine N-acetyltransferase [EC:2.3.1.128]
KO:K03797	0.0071	0.0153	4.00E-04	carboxyl-terminal processing protease [EC:3.4.21.102]
KO:K03798	0.0146	0.0154	0.001	cell division protease FtsH [EC:3.4.24.-]
KO:K03821	0.0088	0.0057	2.00E-04	polyhydroxyalkanoate synthase [EC:2.3.1.-]
KO:K03862	0.0041	0.0077	-1.00E-04	vanillate monooxygenase [EC:1.14.13.82]
KO:K03892	0.0417	0.0362	0	ArsR family transcriptional regulator
KO:K03925	0.0349	0.0372	-3.00E-04	MraZ protein
KO:K03927	0.0111	0.0226	0	carboxylesterase type B [EC:3.1.1.1]
KO:K03980	0.0039	0.0159	-1.00E-04	virulence factor
KO:K04035	0.0037	1.00E-04	-0.0012	magnesium-protoporphyrin IX monomethyl ester (oxidative) cyclase
KO:K04037	0.0028	2.00E-04	-0.0016	light-independent protochlorophyllide reductase subunit L
KO:K04038	0.0044	0	-0.0015	light-independent protochlorophyllide reductase subunit N
KO:K04039	0.0038	0	-0.0011	light-independent protochlorophyllide reductase subunit B
KO:K04040	0.0039	9.00E-04	-0.0017	chlorophyll synthase [EC:2.5.1.62]
KO:K04043	0.0034	0.002	0.0032	molecular chaperone DnaK
KO:K04077	0.0069	0.0012	0.0146	chaperonin GroEL
KO:K04078	0.0075	0.0101	0.0017	chaperonin GroES
KO:K04079	0.0041	0.0199	7.00E-04	molecular chaperone HtpG
KO:K04082	0.0429	0.0488	0	molecular chaperone HscB
KO:K04083	0.0494	0.0386	1.00E-04	molecular chaperone Hsp33
KO:K04087	0.0029	0.0076	8.00E-04	membrane protease subunit HflC [EC:3.4.-.-]
KO:K04088	0.0067	0.0018	0.0011	membrane protease subunit HflK [EC:3.4.-.-]
KO:K04105	0.0491	0.055	0	4-hydroxybenzoate-CoA ligase [EC:6.2.1.27]
KO:K04110	0.0282	0.0389	0	benzoate-CoA ligase [EC:6.2.1.25]
KO:K04117	0.017	0.0242	0	cyclohexanecarboxyl-CoA dehydrogenase [EC:1.3.99.-]
KO:K04565	0.0168	0.0553	0	Cu/Zn superoxide dismutase [EC:1.15.1.1]
KO:K04566	0.0034	0.0015	2.00E-04	lysyl-tRNA synthetase, class I [EC:6.1.1.6]
KO:K04751	0.0159	0.0477	4.00E-04	nitrogen regulatory protein P-II 1
KO:K04754	0.0194	0.0173	1.00E-04	lipoprotein
KO:K04764	0.0039	0.0016	-2.00E-04	integration host factor subunit alpha
KO:K04768	0.0085	0.0028	0	acetoin utilization protein AcuC
KO:K04771	0.0306	0.0327	0	serine protease Do [EC:3.4.21.107]
KO:K05301	0.0279	0.069	-3.00E-04	sulfite dehydrogenase [EC:1.8.2.1]

KO:K05515	0.0332	0.0307	-1.00E-04	penicillin-binding protein 2
KO:K05567	0.0091	0.0137	0	multicomponent Na+:H+ antiporter subunit C
KO:K05603	0.0312	0.0153	0	formimidoylglutamate deiminase [EC:3.5.3.13]
KO:K05773	0.0383	0.0324	0	putative tungstate transport system permease protein
KO:K05786	0.0188	0.0207	0	chloramphenicol-sensitive protein RarD
KO:K05788	0.0041	0.0051	-2.00E-04	integration host factor subunit beta
KO:K05813	0.0042	0.0029	-0.002	sn-glycerol 3-phosphate transport system substrate-binding protein
KO:K05814	0.0043	2.00E-04	-2.00E-04	sn-glycerol 3-phosphate transport system permease protein
KO:K05815	0.0082	0.0193	-2.00E-04	sn-glycerol 3-phosphate transport system permease protein
KO:K05816	0.009	0.0014	-1.00E-04	sn-glycerol 3-phosphate transport system ATP-binding protein
KO:K05836	0.0165	0.0033	0	GntR family transcriptional regulator, histidine utilization
KO:K05838	0.0069	0.0046	1.00E-04	putative thioredoxin
KO:K05896	0.0067	0.0026	-1.00E-04	segregation and condensation protein A
KO:K05903	0.0136	0.036	-1.00E-04	NADH dehydrogenase (quinone) [EC:1.6.99.5]
KO:K06015	0.0036	0.0015	0	
KO:K06016	0.0049	0.0032	-2.00E-04	N-carbamoyl-L-amino-acid hydrolase [EC:3.5.1.87]
KO:K06024	0.0415	0.0322	0	segregation and condensation protein B
KO:K06147	0.005	1.00E-04	6.00E-04	ATP-binding cassette, subfamily B, bacterial
KO:K06207	0.0336	0.1369	1.00E-04	GTP-binding protein
KO:K06443	0.0483	0.041	0	lycopene beta cyclase [EC:1.14.-.-]
KO:K06857	0.0329	0.0194	0	putative tungstate transport system ATP-binding protein
KO:K06860	0.0316	0.0148	0	
KO:K06876	0.0037	0.0028	2.00E-04	
KO:K06881	0.0344	0.0663	0	
KO:K06902	0.0252	0.0283	-3.00E-04	MFS transporter, UMF1 family
KO:K06911	0.0041	0.0041	0.0011	
KO:K06917	0.0038	0.0117	0	tRNA 2-selenouridine synthase [EC:2.9.1.-]
KO:K06942	0.0069	0.0059	2.00E-04	
KO:K06954	0.0083	0.0072	9.00E-04	
KO:K06955	0.0035	0.0029	1.00E-04	
KO:K06958	0.0134	0.0411	-1.00E-04	
KO:K06971	0.0131	0.0137	0	
KO:K06978	0.0043	0.0042	-1.00E-04	
KO:K06985	0.0046	0.0045	0	aspartyl protease family protein
KO:K07021	0.0045	0.001	3.00E-04	
KO:K07042	0.0381	0.0383	0	
KO:K07045	0.0333	0.0859	0	
KO:K07047	0.0081	0.0072	-1.00E-04	
KO:K07050	0.0086	0.0054	0	
KO:K07071	0.0282	0.0245	0	
KO:K07080	0.0247	0.1046	-0.0019	
KO:K07088	0.0342	0.028	1.00E-04	
KO:K07089	0.0033	0.0118	-1.00E-04	
KO:K07093	0.0461	0.0368	-1.00E-04	
KO:K07127	0.0262	0.02	-1.00E-04	5-hydroxyisourate hydrolase [EC:3.5.2.17]
KO:K07130	0.0487	0.0411	0	
KO:K07147	0.0471	0.0815	1.00E-04	
KO:K07152	0.0289	0.0265	2.00E-04	
KO:K07156	0.0121	0.0078	0	
KO:K07157	0.0035	1.00E-04	4.00E-04	
KO:K07160	0.0439	0.0428	0	
KO:K07161	0.0454	0.0319	0	
KO:K07167	0.0044	9.00E-04	0.0019	putative transcriptional regulator
KO:K07170	0.0201	0.0179	-1.00E-04	GAF domain-containing protein
KO:K07175	0.0043	5.00E-04	0	PhoH-like ATPase
KO:K07185	0.033	0.0269	-1.00E-04	tryptophan-rich sensory protein
KO:K07222	0.0393	0.0325	0	putative flavoprotein involved in K+ transport
KO:K07240	0.0026	5.00E-04	-1.00E-04	chromate transporter
KO:K07261	0.0035	0.0022	0	penicillin-insensitive murein endopeptidase [EC:3.4.24.-]
KO:K07266	0.0456	0.055	0	capsular polysaccharide export protein
KO:K07274	0.0326	0.0151	0	outer membrane protein
KO:K07304	0.0036	0.0021	1.00E-04	peptide-methionine (S)-S-oxide reductase [EC:1.8.4.11]
KO:K07305	0.0032	0.0037	2.00E-04	peptide-methionine (R)-S-oxide reductase [EC:1.8.4.12]

KO:K07319	0.0086	0.012	-1.00E-04	putative adenine-specific DNA-methyltransferase [EC:2.1.1.72]
KO:K07323	0.0166	0.0052	1.00E-04	putative toluene tolerance protein
KO:K07335	0.0033	0.0342	-7.00E-04	basic membrane protein A and related proteins
KO:K07393	0.0041	0.0057	4.00E-04	putative glutathione S-transferase
KO:K07397	0.0035	0.019	0	putative redox protein
KO:K07589	0.0064	0.015	0	D-erythro-7,8-dihydroneopterin triphosphate epimerase [EC:5.-.-.-]
KO:K07738	0.0074	0.0087	-2.00E-04	transcriptional repressor NrdR
KO:K07740	0.0477	0.0372	0	regulator of sigma D
KO:K07782	0.0121	0.0311	0	LuxR family transcriptional regulator
KO:K07794	0.0039	1.00E-04	-1.00E-04	putative tricarboxylic transport membrane protein
KO:K07795	0.0089	0.0216	-0.0018	putative tricarboxylic transport membrane protein
KO:K07812	0.0045	0.005	-1.00E-04	trimethylamine-N-oxide reductase (cytochrome c) 2 [EC:1.7.2.3]
KO:K08226	0.0043	0	-0.0036	MFS transporter, BCD family, chlorophyll transporter
KO:K08296	0.0451	0.0451	0	phosphohistidine phosphatase [EC:3.1.3.-]
KO:K08299	0.0074	0.0125	-1.00E-04	carnitinyl-CoA dehydratase [EC:4.2.1.-]
KO:K08303	0.0497	0.0557	-1.00E-04	putative protease [EC:3.4.-.-]
KO:K08738	0.0168	0.0118	5.00E-04	cytochrome c
KO:K08926	0.0042	0	-0.0168	light-harvesting complex 1 alpha chain
KO:K08927	0.0033	0.0024	-0.005	light-harvesting complex 1 beta chain
KO:K08928	0.004	0.0012	-0.0082	photosynthetic reaction center L subunit
KO:K08929	0.0043	4.00E-04	-0.0106	photosynthetic reaction center M subunit
KO:K09004	0.0034	0.0223	-2.00E-04	hypothetical protein
KO:K09007	0.0031	0.002	0.0013	hypothetical protein
KO:K09013	0.0469	0.0243	3.00E-04	Fe-S cluster assembly ATP-binding protein
KO:K09014	0.0032	0.0057	7.00E-04	Fe-S cluster assembly protein SufB
KO:K09125	0.004	0.0098	3.00E-04	hypothetical protein
KO:K09472	0.0118	0.0039	-1.00E-04	gamma-glutamyl-gamma-aminobutyraldehyde dehydrogenase [EC:1.2.1.-]
KO:K09689	0.0312	0.0387	0	capsular polysaccharide transport system ATP-binding protein
KO:K09701	0.0278	0.0132	3.00E-04	hypothetical protein
KO:K09709	0.0077	0.0015	3.00E-04	hypothetical protein
KO:K09760	0.0185	0.0113	0	DNA recombination protein RmuC
KO:K09791	0.0474	0.0513	0	hypothetical protein
KO:K09796	0.008	0.0045	1.00E-04	hypothetical protein
KO:K09844	0.0042	0	-2.00E-04	hydroxyneurosporene dehydrogenase
KO:K09845	0.0041	0.0041	-8.00E-04	methoxyneurosporene dehydrogenase [EC:1.14.99.-]
KO:K09846	0.0041	4.00E-04	-3.00E-04	hydroxyneurosporene methyltransferase [EC:2.1.1.-]
KO:K09847	0.0033	0.0049	-3.00E-04	spheroidene monooxygenase [EC:1.-.-.-]
KO:K09861	0.0399	0.0607	2.00E-04	hypothetical protein
KO:K09882	0.0038	7.00E-04	2.00E-04	cobaltochelatase CobS [EC:6.6.1.2]
KO:K09883	0.0313	0.0336	1.00E-04	cobaltochelatase CobT [EC:6.6.1.2]
KO:K09919	0.0034	0.0051	1.00E-04	hypothetical protein
KO:K09921	0.0042	0.0316	-1.00E-04	hypothetical protein
KO:K09949	0.0111	0.0149	1.00E-04	hypothetical protein
KO:K09966	0.0316	0.0214	0	hypothetical protein
KO:K09967	0.0264	0.0289	-1.00E-04	hypothetical protein
KO:K09986	0.0309	0.0242	1.00E-04	hypothetical protein
KO:K09987	0.0036	0.0026	3.00E-04	hypothetical protein
KO:K10013	0.0245	0.0278	0	lysine/arginine/ornithine transport system substrate-binding protein
KO:K10027	0.0037	5.00E-04	-0.0013	phytoene dehydrogenase [EC:1.14.99.-]
KO:K10039	0.0074	0.0194	-2.00E-04	putative glutamine transport system substrate-binding protein
KO:K10040	0.0089	0.0209	0	putative glutamine transport system permease protein
KO:K10108	0.0158	0.0186	0	maltose/maltodextrin transport system substrate-binding protein
KO:K10231	0.0184	0.033	0	kojibiose phosphorylase [EC:2.4.1.230]
KO:K10439	0.0071	0.0119	-1.00E-04	ribose transport system substrate-binding protein
KO:K10441	0.0035	0.0011	0	ribose transport system ATP-binding protein [EC:3.6.3.17]
KO:K10559	0.017	0.0233	0	rhamnose transport system substrate-binding protein
KO:K10562	0.0312	0.045	0	rhamnose transport system ATP-binding protein [EC:3.6.3.17]
KO:K10764	0.0416	0.0253	1.00E-04	O-succinylhomoserine sulfhydrylase [EC:2.5.1.-]
KO:K10806	0.0227	0.0379	0	acyl-CoA thioesterase YciA [EC:3.1.2.-]
KO:K10960	0.0037	1.00E-04	-5.00E-04	geranylgeranyl reductase [EC:1.3.1.83]
KO:K11069	0.0058	0.0077	-3.00E-04	spermidine/putrescine transport system substrate-binding protein
KO:K11070	0.0081	0.0055	0	spermidine/putrescine transport system permease protein
KO:K11071	0.0032	0	-1.00E-04	spermidine/putrescine transport system permease protein

KO:K11073	0.0074	0.0139	-2.00E-04	putrescine transport system substrate-binding protein
KO:K11076	0.0285	0.0204	0	putrescine transport system ATP-binding protein
KO:K11175	0.0036	0.0055	1.00E-04	phosphoribosylglycinamide formyltransferase 1 [EC:2.1.2.2]
KO:K11333	0.004	2.00E-04	-0.0041	chlorophyllide reductase iron protein subunit X [EC:1.18.6.1]
KO:K11334	0.0026	2.00E-04	-0.0022	chlorophyllide reductase subunit Y [EC:1.18.1.-]
KO:K11335	0.0036	1.00E-04	-0.0023	chlorophyllide reductase subunit Z [EC:1.18.-.-]
KO:K11336	0.004	0	-0.0023	3-vinyl bacteriochlorophyllide hydratase [EC:4.2.1.-]
KO:K11337	0.0033	1.00E-04	-0.0034	3-hydroxyethyl bacteriochlorophyllide a dehydrogenase [EC:1.-.-.-]
KO:K11381	0.0382	0.0673	0	2-oxoisovalerate dehydrogenase E1 component [EC:1.2.4.4]
KO:K11707	0.0115	0.0479	1.00E-04	manganese/zinc/iron transport system substrate-binding protein
KO:K11717	0.0045	9.00E-04	3.00E-04	cysteine desulfurase / selenocysteine lyase [EC:2.8.1.7 4.4.1.16]
KO:K11749	0.0199	0.0142	1.00E-04	regulator of sigma E protease [EC:3.4.24.-]
KO:K11752	0.0301	0.0232	-1.00E-04	diaminohydroxyphosphoribosylaminopyrimidine deaminase /
KO:K11787	0.0117	0.0489	1.00E-04	phosphoribosylamine--glycine ligase / phosphoribosylglycinamide
KO:K11900	0.0462	0.075	0	type VI secretion system protein ImpC
KO:K11942	0.0032	0.0012	0	methylmalonyl-CoA mutase [EC:5.4.99.2]
KO:K11956	0.0448	0.0432	0	neutral amino acid transport system permease protein
KO:K12262	0.0312	0.0208	0	cytochrome b561
KO:K12339	0.0067	0.0021	0	cysteine synthase B [EC:2.5.1.47]
KO:K12369	0.003	0.0501	-1.00E-04	dipeptide transport system permease protein
KO:K12370	0.0375	0.0469	-1.00E-04	dipeptide transport system permease protein
KO:K12371	0.0138	0.0531	0	dipeptide transport system ATP-binding protein
KO:K12941	0.025	0.0092	-1.00E-04	aminobenzoyl-glutamate utilization protein B
KO:K13038	0.0355	0.0349	2.00E-04	phosphopantothenoylcysteine decarboxylase /
KO:K13292	0.0473	0.0425	0	phosphatidylglycerol:prolipoprotein diacylglycerol transferase
KO:K13482	0.0209	0.0132	-1.00E-04	xanthine dehydrogenase large subunit [EC:1.17.1.4]
KO:K13581	0.0037	0.004	-2.00E-04	modification methylase [EC:2.1.1.72]
KO:K13789	0.004	0	-1.00E-04	geranylgeranyl diphosphate synthase, type II [EC:2.5.1.1 2.5.1.10
KO:K13893	0.0078	0.0116	0	microcin C transport system substrate-binding protein
KO:K13991	0.0041	0.0024	-5.00E-04	photosynthetic reaction center H subunit
KO:K13992	0.0024	9.00E-04	-0.0026	photosynthetic reaction center cytochrome c subunit
KO:K14083	0.004	0.0019	-1.00E-04	
KO:K14155	0.0043	0.0011	0	
KO:K14446	0.0128	0.0228	0	
KO:K14448	0.0133	0.0055	1.00E-04	
KO:K14470	0.0044	0.0035	1.00E-04	
KO:K14519	0.0422	0.047	0	

Figure S1

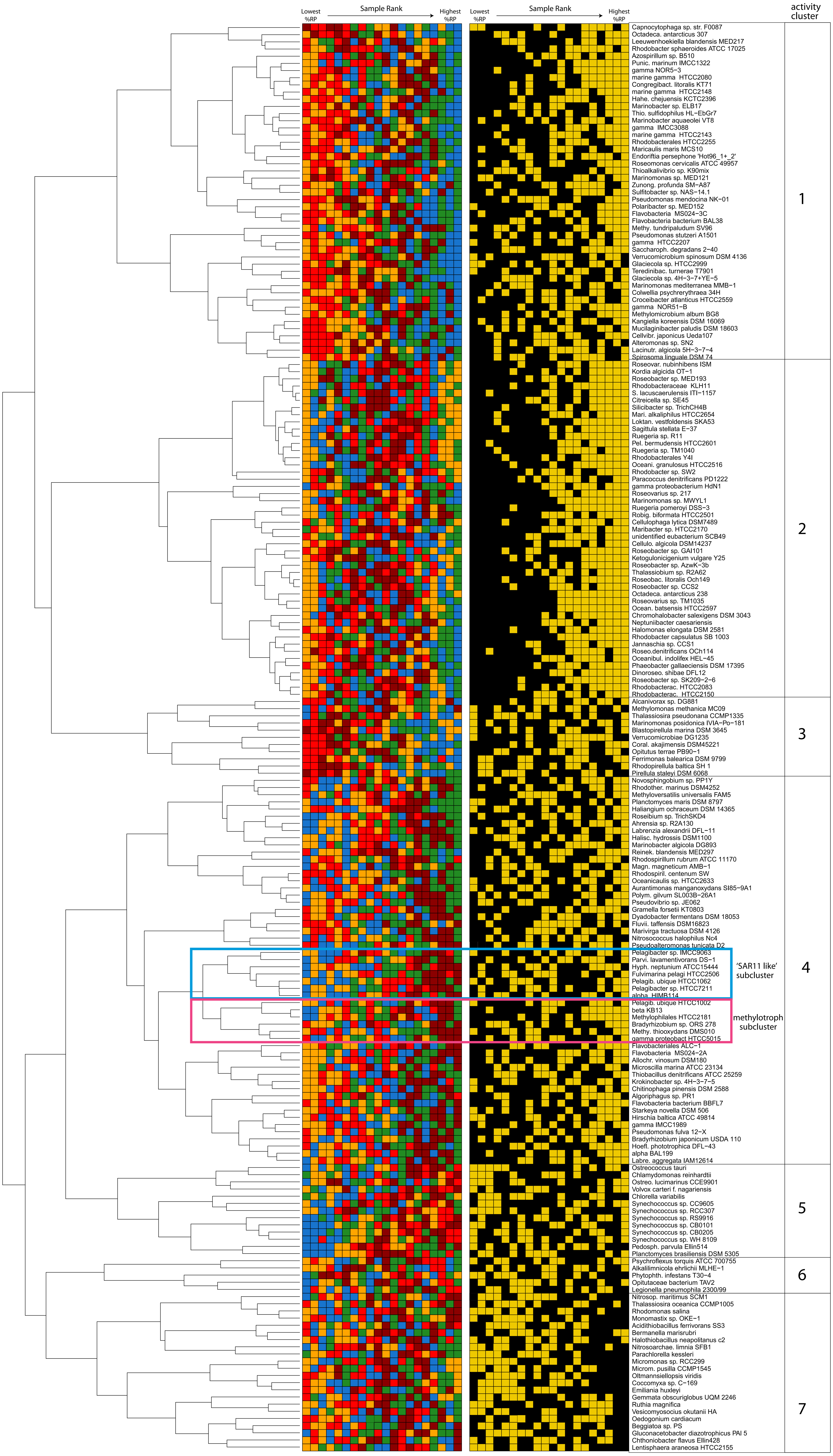

Lowest %RP
Sample Rank
Highest %RP
Lowest %RP
Sample Rank
Highest %RP
activity cluster
Capnocytophaga sp. str. F0087
Octadeca. antarcticus 307
Leeuwenhoekiella blandensis MED217
Rhodobacter sphaeroides ATCC 17025
Azospirillum sp. B510
Punic. marinum IMCC1322
gamma NOR5−3
marine gamma HTCC2080
Congregibact. litoralis KT71
marine gamma HTCC2148
Hahe. chejuensis KCTC2396
Marinobacter sp. ELB17
Thio. sulfidophilus HL−EbGr7
Marinobacter aquaeolei VT8
gamma IMCC3088
marine gamma HTCC2143
Rhodobacterales HTCC2255
Maricaulis maris MCS10
Endoriftia persephone 'Hot96_1+_2'
Roseomonas cervicalis ATCC 49957
Thioalkalivibrio sp. K90mix
Marinomonas sp. MED121
Zunong. profunda SM−A87
Sulfitobacter sp. NAS−14.1
Pseudomonas mendocina NK−01
Polaribacter sp. MED152
Flavobacteria MS024−3C
Flavobacteria bacterium BAL38
Methy. tundripaludum SV96
Pseudomonas stutzeri A1501
gamma HTCC2207
Saccharoph. degradans 2−40
Verrucomicrobium spinosum DSM 4136
Glaciecola sp. HTCC2999
Teredinibac. turnerae T7901
Glaciecola sp. 4H−3−7+YE−5
Marinomonas mediterranea MMB−1
Colwellia psychrerythraea 34H
Croceibacter atlanticus HTCC2559
gamma NOR51−B
Methylomicrobium album BG8
Kangiella koreensis DSM 16069
Mucilaginibacter paludis DSM 18603
Cellvibr. japonicus Ueda107
Alteromonas sp. SN2
Lacinutr. algicola 5H−3−7−4
Spirosoma linguale DSM 74
1
Roseovar. nubinhibens ISM
Kordia algicida OT−1
Roseobacter sp. MED193
Rhodobacteraceae KLH11
S. lacuscaerulensis ITI−1157
Citreicella sp. SE45
Silicibacter sp. TrichCH4B
Mari. alkaliphilus HTCC2654
Loktan. vestfoldensis SKA53
Sagittula stellata E−37
Ruegeria sp. R11
Pel. bermudensis HTCC2601
Ruegeria sp. TM1040
Rhodobacterales Y4I
Oceani. granulosus HTCC2516
Rhodobacter sp. SW2
Paracoccus denitrificans PD1222
gamma proteobacterium HdN1
Roseovarius sp. 217
Marinomonas sp. MWYL1
Ruegeria pomeroyi DSS−3
Robig. biformata HTCC2501
Cellulophaga lytica DSM7489
Maribacter sp. HTCC2170
unidentified eubacterium SCB49
Cellulo. algicola DSM14237
Roseobacter sp. GAI101
Ketogulonicigenium vulgare Y25
Roseobacter sp. AzwK−3b
Thalassiobium sp. R2A62
Roseobac. litoralis Och149
Roseobacter sp. CCS2
Octadeca. antarcticus 238
Roseovarius sp. TM1035
Ocean. batsensis HTCC2597
Chromohalobacter salexigens DSM 3043
Neptuniibacter caesariensis
Halomonas elongata DSM 2581
Rhodobacter capsulatus SB 1003
Jannaschia sp. CCS1
Roseo.denitrificans OCh114
Oceanibul. indolifex HEL−45
Phaeobacter gallaeciensis DSM 17395
Dinoroseo. shibae DFL12
Roseobacter sp. SK209−2−6
Rhodobacterac. HTCC2083
Rhodobacterac. HTCC2150
2
Alcanivorax sp. DG881
Methylomonas methanica MC09
Thalassiosira pseudonana CCMP1335
Marinomonas posidonica IVIA−Po−181
Blastopirellula marina DSM 3645
Verrucomicrobiae DG1235
Coral. akajimensis DSM45221
Opitutus terrae PB90−1
Ferrimonas balearica DSM 9799
Rhodopirellula baltica SH 1
Pirellula staleyi DSM 6068
3
Novosphingobium sp. PP1Y
Rhodother. marinus DSM4252
Methyloversatilis universalis FAM5
Planctomyces maris DSM 8797
Haliangium ochraceum DSM 14365
Roseibium sp. TrichSKD4
Ahrensia sp. R2A130
Labrenzia alexandrii DFL−11
Halisc. hydrossis DSM1100
Marinobacter algicola DG893
Reinek. blandensis MED297
Rhodospirillum rubrum ATCC 11170
Magn. magneticum AMB−1
Rhodospiril. centenum SW
Oceanicaulis sp. HTCC2633
Aurantimonas manganoxydans SI85−9A1
Polym. gilvum SL003B−26A1
Pseudovibrio sp. JE062
Gramella forsetii KT0803
Dyadobacter fermentans DSM 18053
Fluvii. taffensis DSM16823
Marivirga tractuosa DSM 4126
Nitrosococcus halophilus Nc4
Pseudoalteromonas tunicata D2
Pelagibacter sp. IMCC9063
Parvi. lavamentivorans DS−1
Hyph. neptunium ATCC15444
Fulvimarina pelagi HTCC2506
Pelagib. ubique HTCC1062
Pelagibacter sp. HTCC7211
alpha HIMB114
'SAR11 like' subcluster
Pelagib. ubique HTCC1002
beta KB13
Methylophilales HTCC2181
Bradyrhizobium sp. ORS 278
Methy. thiooxydans DMS010
gamma proteobact HTCC5015
methylotroph subcluster
Flavobacteriales ALC−1
Flavobacteria MS024−2A
Allochr. vinosum DSM180
Microscilla marina ATCC 23134
Thiobacillus denitrificans ATCC 25259
Krokinobacter sp. 4H−3−7−5
Chitinophaga pinensis DSM 2588
Algoriphagus sp. PR1
Flavobacteria bacterium BBFL7
Starkeya novella DSM 506
Hirschia baltica ATCC 49814
gamma IMCC1989
Pseudomonas fulva 12−X
Bradyrhizobium japonicum USDA 110
Hoefl. phototrophica DFL−43
alpha BAL199
Labre. aggregata IAM12614
4
Ostreococcus tauri
Chlamydomonas reinhardtii
Ostreo. lucimarinus CCE9901
Volvox carteri f. nagariensis
Chlorella variabilis
Synechococcus sp. CC9605
Synechococcus sp. RCC307
Synechococcus sp. RS9916
Synechococcus sp. CB0101
Synechococcus sp. CB0205
Synechococcus sp. WH 8109
Pedosph. parvula Ellin514
Planctomyces brasiliensis DSM 5305
5
Psychroflexus torquis ATCC 700755
Alkalilimnicola ehrlichii MLHE−1
Phytophth. infestans T30−4
Opitutaceae bacterium TAV2
Legionella pneumophila 2300/99
6
Nitrosop. maritimus SCM1
Thalassiosira oceanica CCMP1005
Rhodomonas salina
Monomastix sp. OKE−1
Acidithiobacillus ferrivorans SS3
Bermanella marisrubri
Halothiobacillus neapolitanus c2
Nitrosoarchae. limnia SFB1
Parachlorella kessleri
Micromonas sp. RCC299
Microm. pusilla CCMP1545
Oltmannsiellopsis viridis
Coccomyxa sp. C−169
Emiliania huxleyi
Gemmata obscuriglobus UQM 2246
Ruthia magnifica
Vesicomyosocius okutanii HA
Oedogonium cardiacum
Beggiatoa sp. PS
Gluconacetobacter diazotrophicus PAI 5
Chthoniobacter flavus Ellin428
Lentisphaera araneosa HTCC2155
7

Figure S2

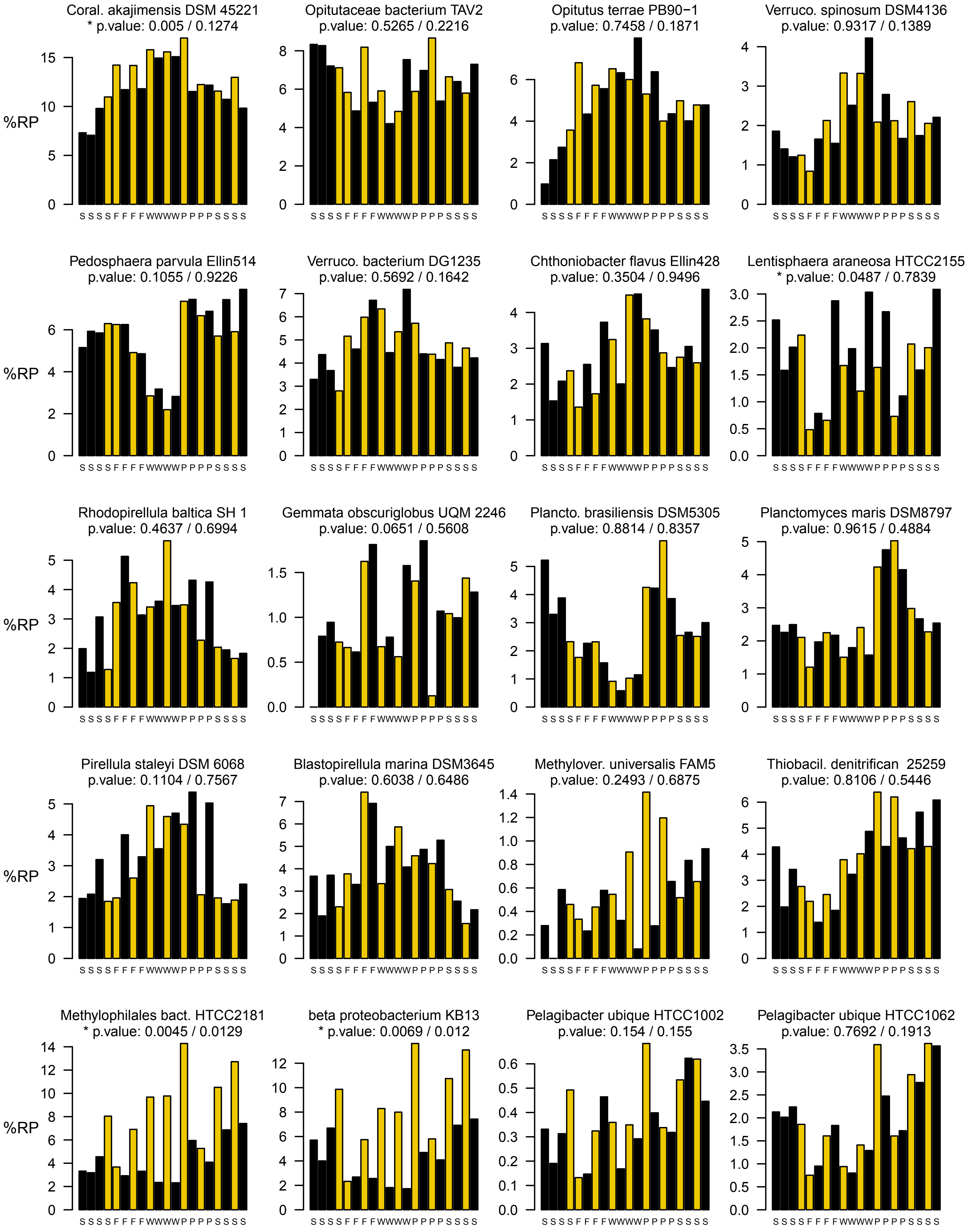
Coral. akajimensis DSM 45221
* p.value: 0.005 / 0.1274
Opitutaceae bacterium TAV2
p.value: 0.5265 / 0.2216
Opitutus terrae PB90−1
p.value: 0.7458 / 0.1871
Verruco. spinosum DSM4136
p.value: 0.9317 / 0.1389
Pedosphaera parvula Ellin514
p.value: 0.1055 / 0.9226
Verruco. bacterium DG1235
p.value: 0.5692 / 0.1642
Chthoniobacter flavus Ellin428
p.value: 0.3504 / 0.9496
Lentisphaera araneosa HTCC2155
* p.value: 0.0487 / 0.7839
Rhodopirellula baltica SH 1
p.value: 0.4637 / 0.6994
Gemmata obscuriglobus UQM 2246
p.value: 0.0651 / 0.5608
Plancto. brasiliensis DSM5305
p.value: 0.8814 / 0.8357
Planctomyces maris DSM8797
p.value: 0.9615 / 0.4884
Pirellula staleyi DSM 6068
p.value: 0.1104 / 0.7567
Blastopirellula marina DSM3645
p.value: 0.6038 / 0.6486
Methylover. universalis FAM5
p.value: 0.2493 / 0.6875
Thiobacil. denitrifican 25259
p.value: 0.8106 / 0.5446
Methylophilales bact. HTCC2181
* p.value: 0.0045 / 0.0129
beta proteobacterium KB13
* p.value: 0.0069 / 0.012
Pelagibacter ubique HTCC1002
p.value: 0.154 / 0.155
Pelagibacter ubique HTCC1062
p.value: 0.7692 / 0.1913
%RP
S S S S F F F F W W W W W P P P P S S S S

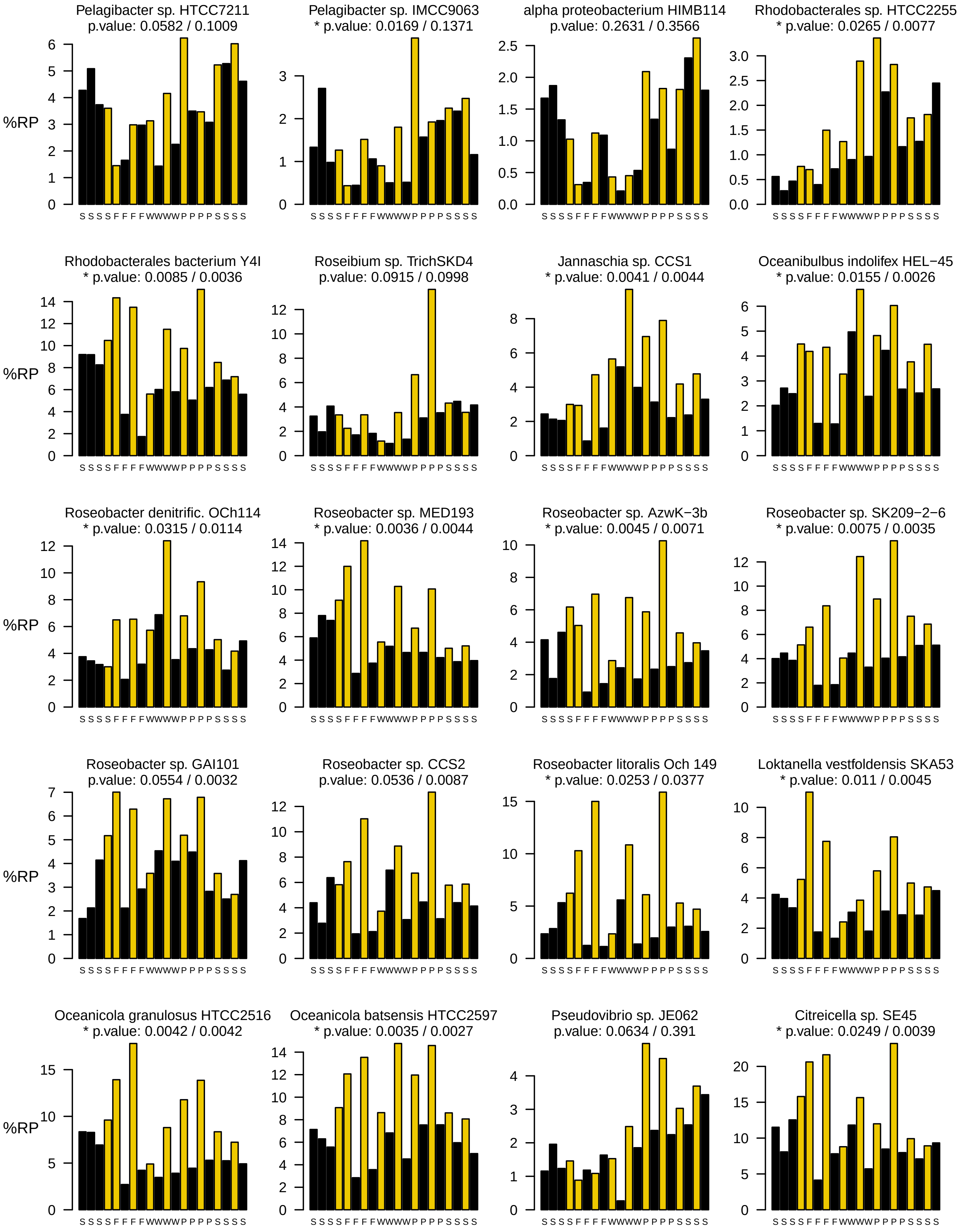

Pelagibacter sp. HTCC7211
p.value: 0.0582 / 0.1009
Pelagibacter sp. IMCC9063
* p.value: 0.0169 / 0.1371
alpha proteobacterium HIMB114
p.value: 0.2631 / 0.3566
Rhodobacterales sp. HTCC2255
* p.value: 0.0265 / 0.0077
Rhodobacterales bacterium Y4I
* p.value: 0.0085 / 0.0036
Roseibium sp. TrichSKD4
p.value: 0.0915 / 0.0998
Jannaschia sp. CCS1
* p.value: 0.0041 / 0.0044
Oceanibulbus indolifex HEL−45
* p.value: 0.0155 / 0.0026
Roseobacter denitrific. OCh114
* p.value: 0.0315 / 0.0114
Roseobacter sp. MED193
* p.value: 0.0036 / 0.0044
Roseobacter sp. AzwK−3b
* p.value: 0.0045 / 0.0071
Roseobacter sp. SK209−2−6
* p.value: 0.0075 / 0.0035
Roseobacter sp. GAI101
p.value: 0.0554 / 0.0032
Roseobacter sp. CCS2
p.value: 0.0536 / 0.0087
Roseobacter litoralis Och 149
* p.value: 0.0253 / 0.0377
Loktanella vestfoldensis SKA53
* p.value: 0.011 / 0.0045
Oceanicola granulosus HTCC2516
* p.value: 0.0042 / 0.0042
Oceanicola batsensis HTCC2597
* p.value: 0.0035 / 0.0027
Pseudovibrio sp. JE062
p.value: 0.0634 / 0.391
Citreicella sp. SE45
* p.value: 0.0249 / 0.0039
%RP
S S S S F F F F W W W W W P P P P S S S S

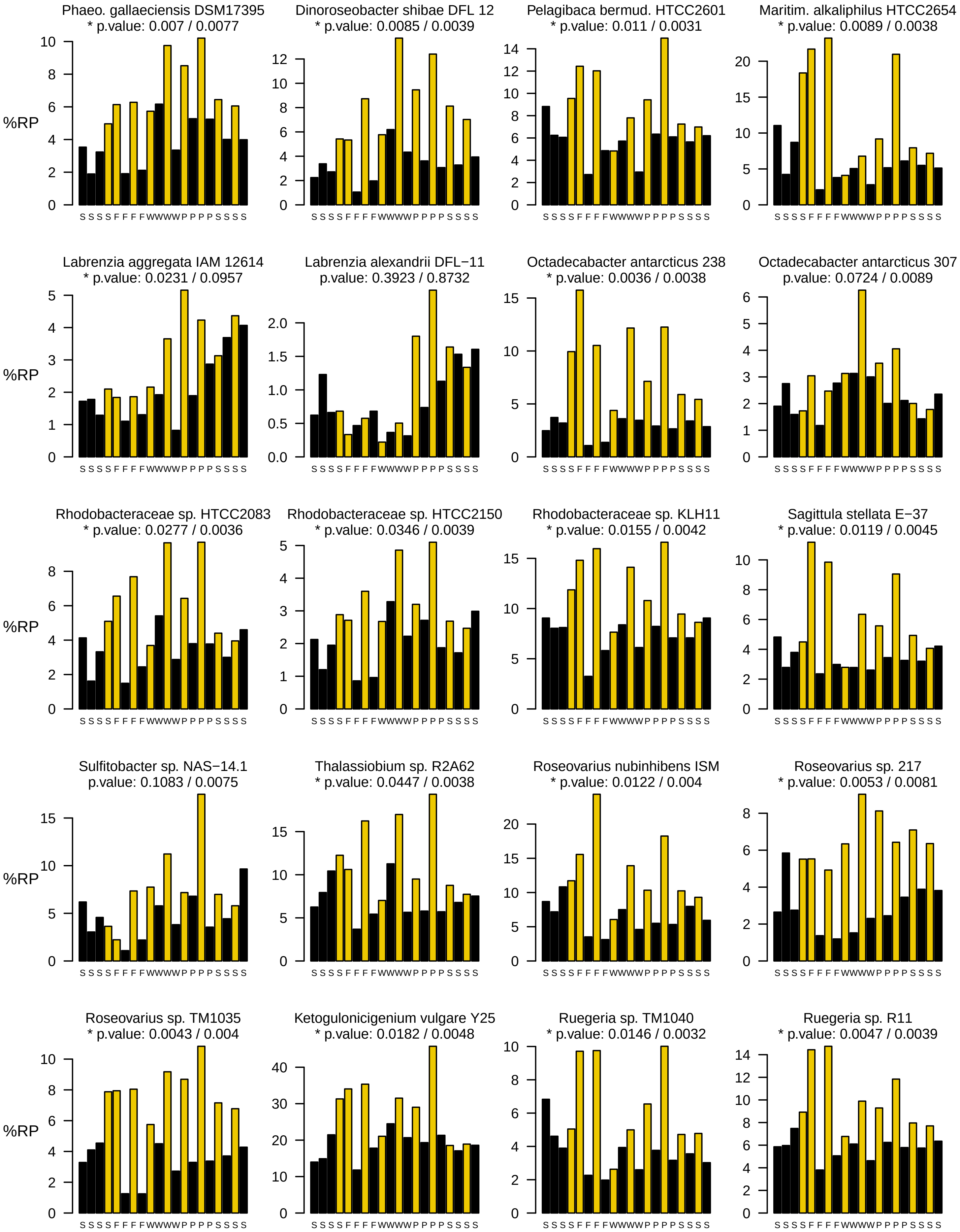
Phaeo. gallaeciensis DSM17395
* p.value: 0.007 / 0.0077
Dinoroseobacter shibae DFL 12
* p.value: 0.0085 / 0.0039
Pelagibaca bermud. HTCC2601
* p.value: 0.011 / 0.0031
Maritim. alkaliphilus HTCC2654
* p.value: 0.0089 / 0.0038
Labrenzia aggregata IAM 12614
* p.value: 0.0231 / 0.0957
Labrenzia alexandrii DFL−11
p.value: 0.3923 / 0.8732
Octadecabacter antarcticus 238
* p.value: 0.0036 / 0.0038
Octadecabacter antarcticus 307
p.value: 0.0724 / 0.0089
Rhodobacteraceae sp. HTCC2083
* p.value: 0.0277 / 0.0036
Rhodobacteraceae sp. HTCC2150
* p.value: 0.0346 / 0.0039
Rhodobacteraceae sp. KLH11
* p.value: 0.0155 / 0.0042
Sagittula stellata E−37
* p.value: 0.0119 / 0.0045
Sulfitobacter sp. NAS−14.1
p.value: 0.1083 / 0.0075
Thalassiobium sp. R2A62
* p.value: 0.0447 / 0.0038
Roseovarius nubinhibens ISM
* p.value: 0.0122 / 0.004
Roseovarius sp. 217
* p.value: 0.0053 / 0.0081
Roseovarius sp. TM1035
* p.value: 0.0043 / 0.004
Ketogulonicigenium vulgare Y25
* p.value: 0.0182 / 0.0048
Ruegeria sp. TM1040
* p.value: 0.0146 / 0.0032
Ruegeria sp. R11
* p.value: 0.0047 / 0.0039
%RP
S S S S F F F F W W W W W P P P P S S S S

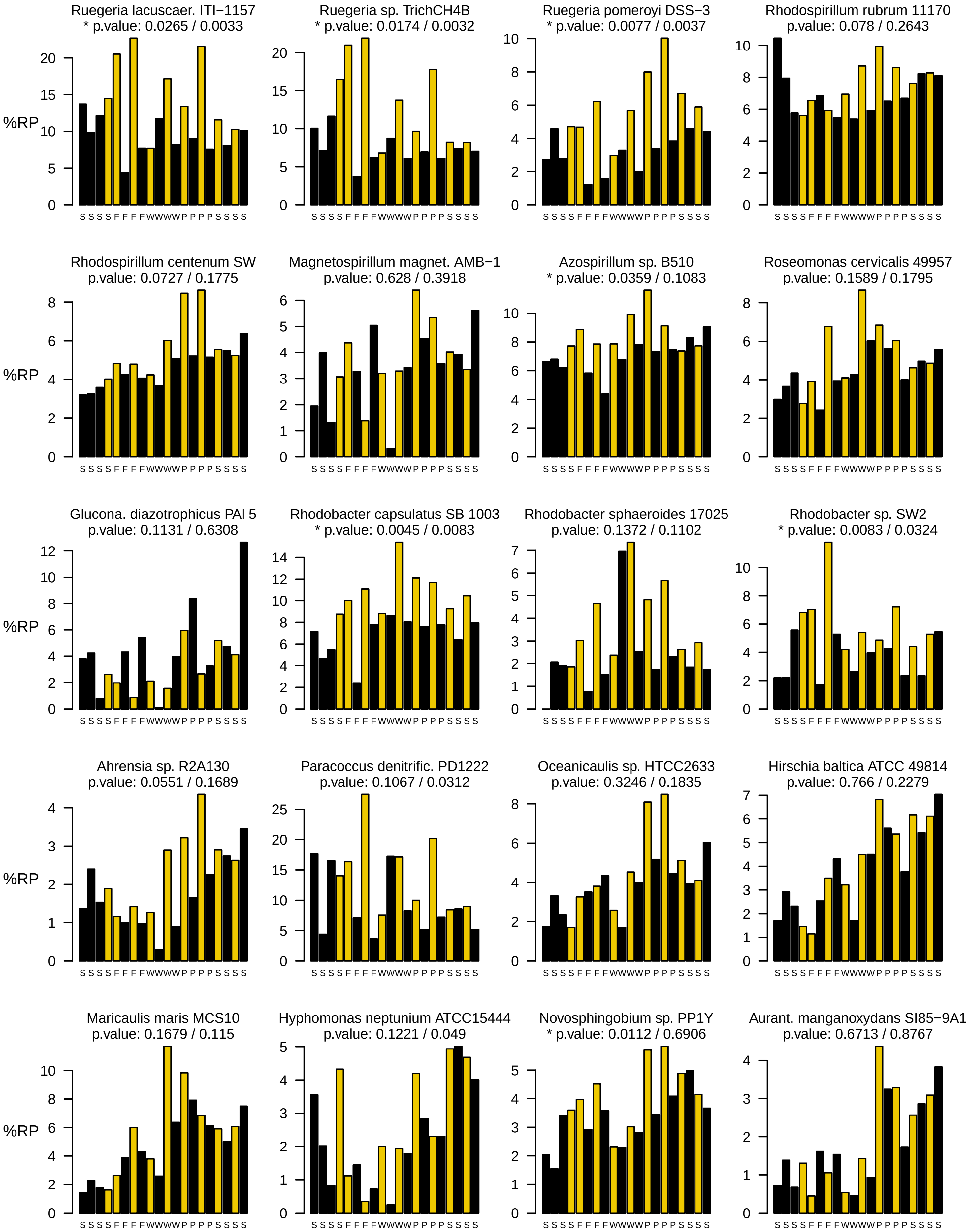

Ruegeria lacuscaer. ITI−1157
* p.value: 0.0265 / 0.0033
Ruegeria sp. TrichCH4B
* p.value: 0.0174 / 0.0032
Ruegeria pomeroyi DSS−3
* p.value: 0.0077 / 0.0037
Rhodospirillum rubrum 11170
p.value: 0.078 / 0.2643
Rhodospirillum centenum SW
p.value: 0.0727 / 0.1775
Magnetospirillum magnet. AMB−1
p.value: 0.628 / 0.3918
Azospirillum sp. B510
* p.value: 0.0359 / 0.1083
Roseomonas cervicalis 49957
p.value: 0.1589 / 0.1795
Glucona. diazotrophicus PAl 5
p.value: 0.1131 / 0.6308
Rhodobacter capsulatus SB 1003
* p.value: 0.0045 / 0.0083
Rhodobacter sphaeroides 17025
p.value: 0.1372 / 0.1102
Rhodobacter sp. SW2
* p.value: 0.0083 / 0.0324
Ahrensia sp. R2A130
p.value: 0.0551 / 0.1689
Paracoccus denitrific. PD1222
p.value: 0.1067 / 0.0312
Oceanicaulis sp. HTCC2633
p.value: 0.3246 / 0.1835
Hirschia baltica ATCC 49814
p.value: 0.766 / 0.2279
Maricaulis maris MCS10
p.value: 0.1679 / 0.115
Hyphomonas neptunium ATCC15444
p.value: 0.1221 / 0.049
Novosphingobium sp. PP1Y
* p.value: 0.0112 / 0.6906
Aurant. manganoxydans SI85−9A1
p.value: 0.6713 / 0.8767
%RP

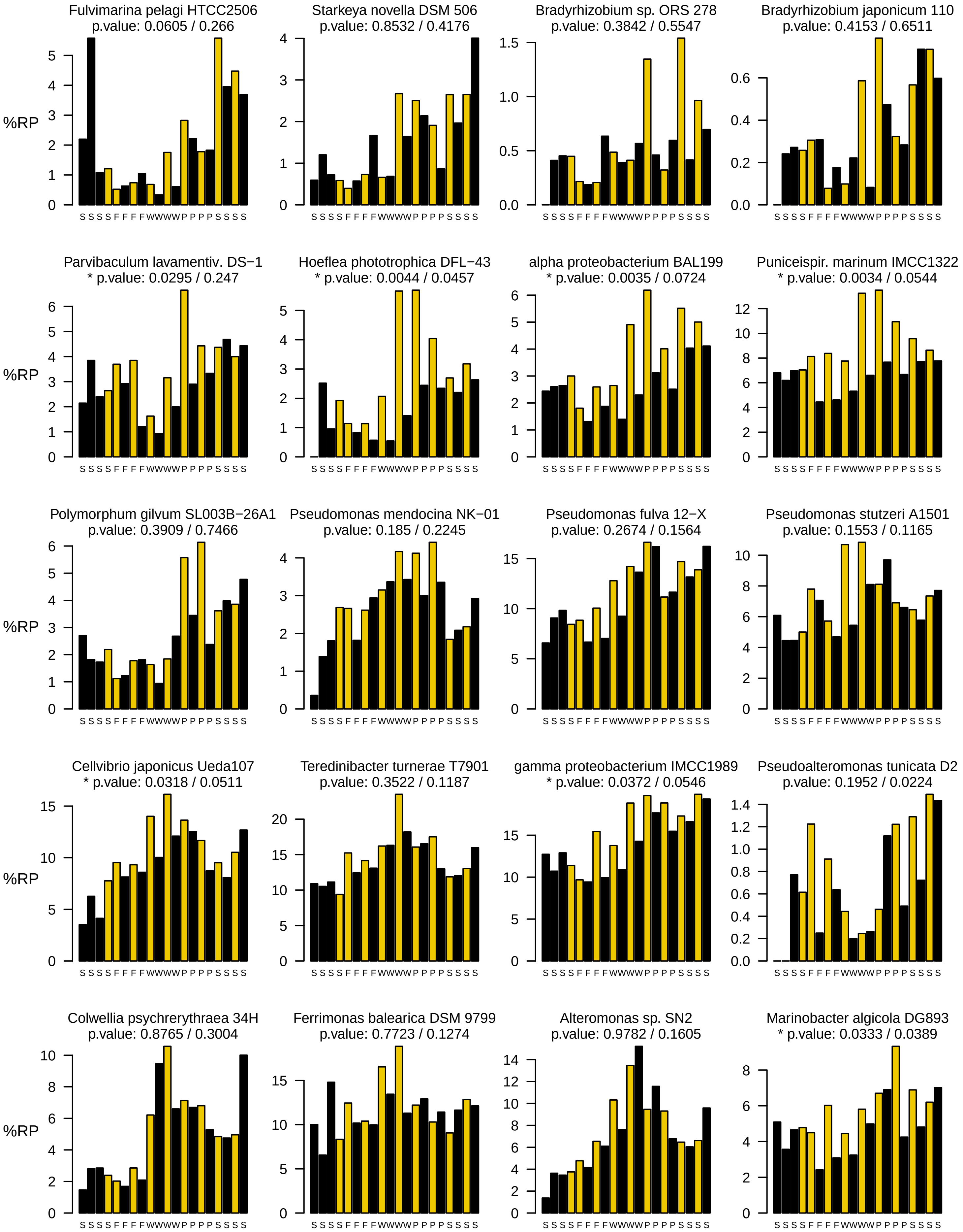
Fulvimarina pelagi HTCC2506
p.value: 0.0605 / 0.266
Starkeya novella DSM 506
p.value: 0.8532 / 0.4176
Bradyrhizobium sp. ORS 278
p.value: 0.3842 / 0.5547
Bradyrhizobium japonicum 110
p.value: 0.4153 / 0.6511
Parvibaculum lavamentiv. DS−1
* p.value: 0.0295 / 0.247
Hoeflea phototrophica DFL−43
* p.value: 0.0044 / 0.0457
alpha proteobacterium BAL199
* p.value: 0.0035 / 0.0724
Puniceispir. marinum IMCC1322
* p.value: 0.0034 / 0.0544
Polymorphum gilvum SL003B−26A1
p.value: 0.3909 / 0.7466
Pseudomonas mendocina NK−01
p.value: 0.185 / 0.2245
Pseudomonas fulva 12−X
p.value: 0.2674 / 0.1564
Pseudomonas stutzeri A1501
p.value: 0.1553 / 0.1165
Cellvibrio japonicus Ueda107
* p.value: 0.0318 / 0.0511
Teredinibacter turnerae T7901
p.value: 0.3522 / 0.1187
gamma proteobacterium IMCC1989
* p.value: 0.0372 / 0.0546
Pseudoalteromonas tunicata D2
p.value: 0.1952 / 0.0224
Colwellia psychrerythraea 34H
p.value: 0.8765 / 0.3004
Ferrimonas balearica DSM 9799
p.value: 0.7723 / 0.1274
Alteromonas sp. SN2
p.value: 0.9782 / 0.1605
Marinobacter algicola DG893
* p.value: 0.0333 / 0.0389
%RP
S S S S F F F F W W W W W P P P P S S S S

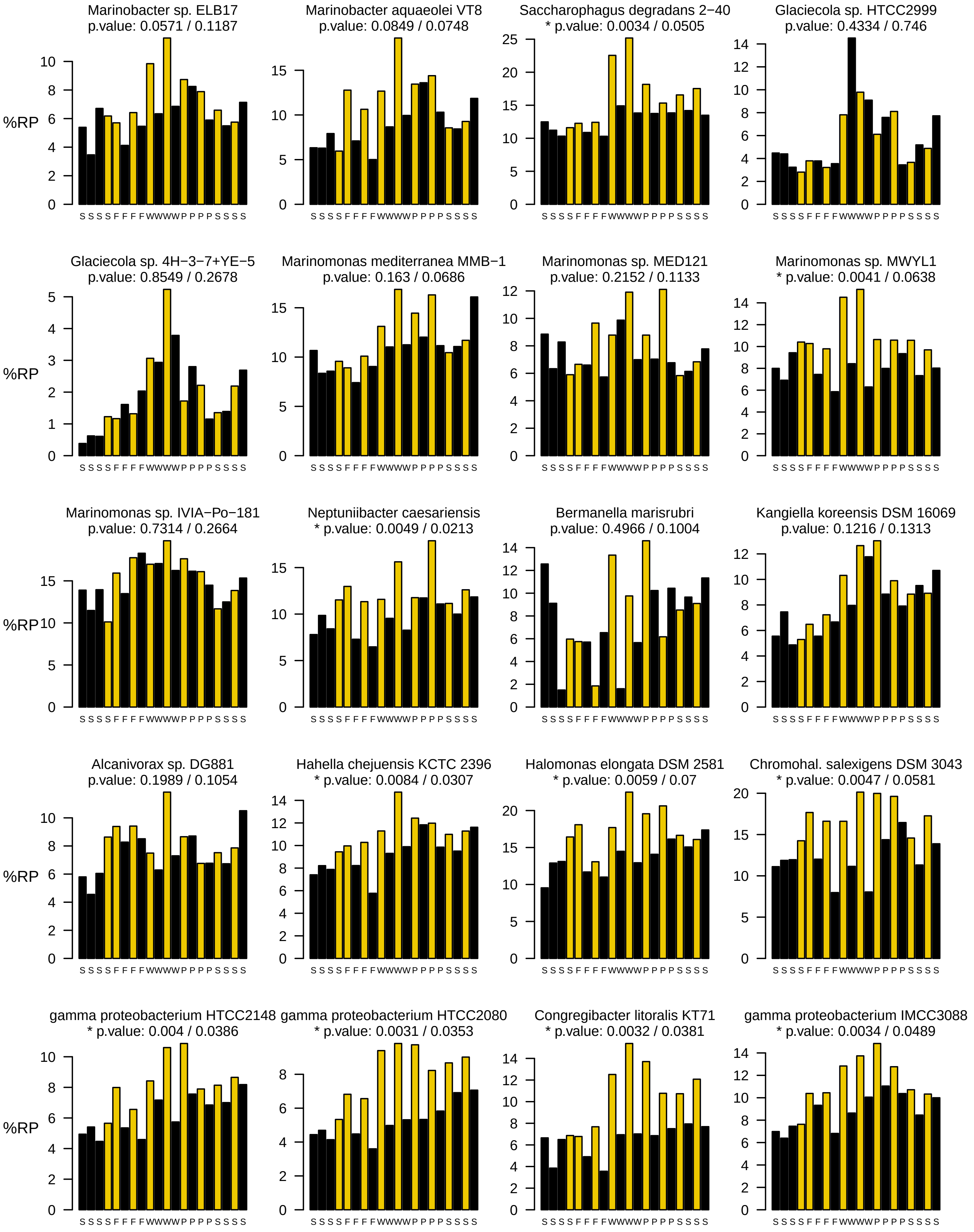
Marinobacter sp. ELB17
p.value: 0.0571 / 0.1187
Marinobacter aquaeolei VT8
p.value: 0.0849 / 0.0748
Saccharophagus degradans 2−40
* p.value: 0.0034 / 0.0505
Glaciecola sp. HTCC2999
p.value: 0.4334 / 0.746
Glaciecola sp. 4H−3−7+YE−5
p.value: 0.8549 / 0.2678
Marinomonas mediterranea MMB−1
p.value: 0.163 / 0.0686
Marinomonas sp. MED121
p.value: 0.2152 / 0.1133
Marinomonas sp. MWYL1
* p.value: 0.0041 / 0.0638
Marinomonas sp. IVIA−Po−181
p.value: 0.7314 / 0.2664
Neptuniibacter caesariensis
* p.value: 0.0049 / 0.0213
Bermanella marisrubri
p.value: 0.4966 / 0.1004
Kangiella koreensis DSM 16069
p.value: 0.1216 / 0.1313
Alcanivorax sp. DG881
p.value: 0.1989 / 0.1054
Hahella chejuensis KCTC 2396
* p.value: 0.0084 / 0.0307
Halomonas elongata DSM 2581
* p.value: 0.0059 / 0.07
Chromohal. salexigens DSM 3043
* p.value: 0.0047 / 0.0581
gamma proteobacterium HTCC2148
* p.value: 0.004 / 0.0386
gamma proteobacterium HTCC2080
* p.value: 0.0031 / 0.0353
Congregibacter litoralis KT71
* p.value: 0.0032 / 0.0381
gamma proteobacterium IMCC3088
* p.value: 0.0034 / 0.0489
%RP
S S S S F F F F W W W W P P P P S S S S

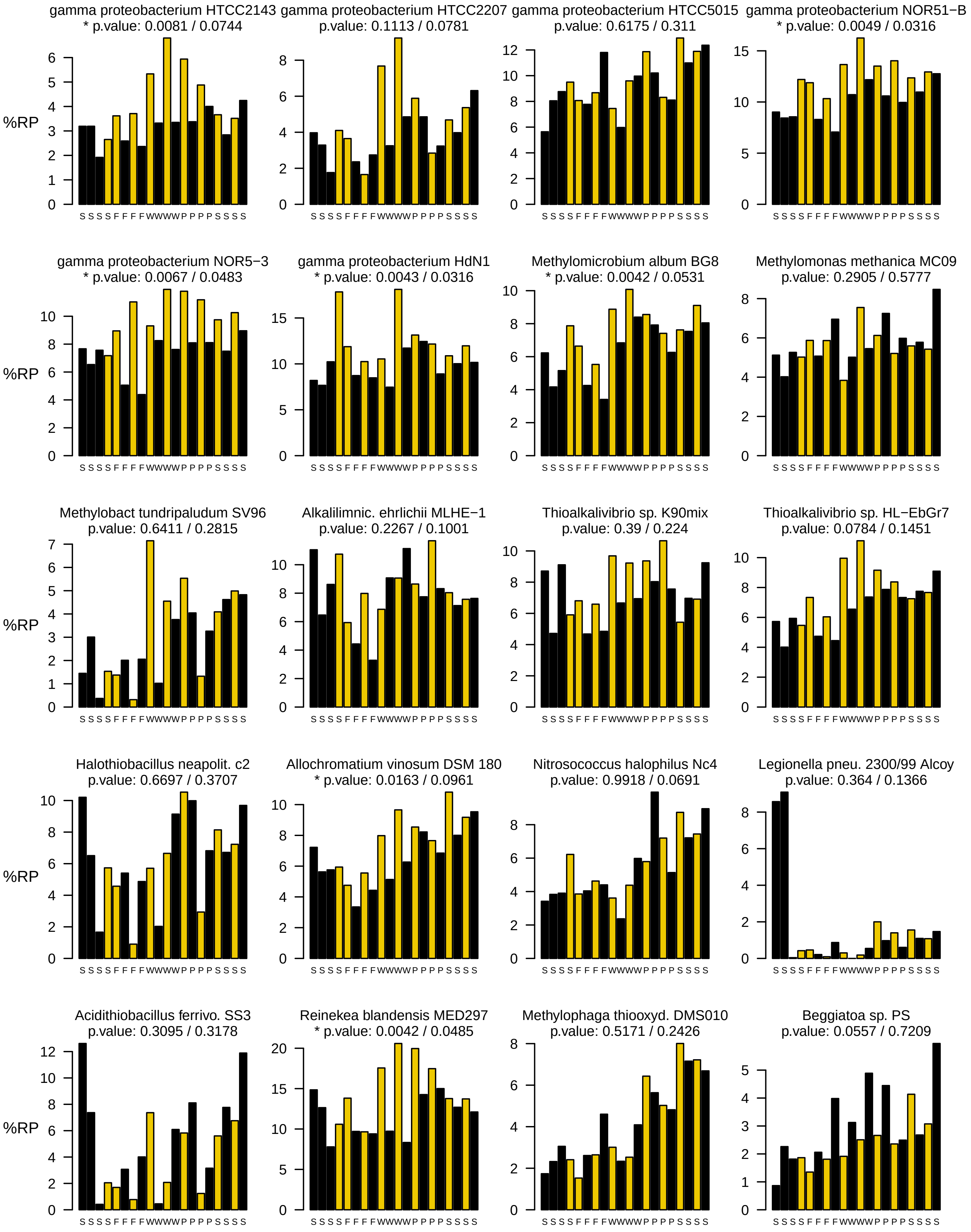
gamma proteobacterium HTCC2143
* p.value: 0.0081 / 0.0744
gamma proteobacterium HTCC2207
p.value: 0.1113 / 0.0781
gamma proteobacterium HTCC5015
p.value: 0.6175 / 0.311
gamma proteobacterium NOR51−B
* p.value: 0.0049 / 0.0316
gamma proteobacterium NOR5−3
* p.value: 0.0067 / 0.0483
gamma proteobacterium HdN1
* p.value: 0.0043 / 0.0316
Methylomicrobium album BG8
* p.value: 0.0042 / 0.0531
Methylomonas methanica MC09
p.value: 0.2905 / 0.5777
Methylobact tundripaludum SV96
p.value: 0.6411 / 0.2815
Alkalilimnic. ehrlichii MLHE−1
p.value: 0.2267 / 0.1001
Thioalkalivibrio sp. K90mix
p.value: 0.39 / 0.224
Thioalkalivibrio sp. HL−EbGr7
p.value: 0.0784 / 0.1451
Halothiobacillus neapolit. c2
p.value: 0.6697 / 0.3707
Allochromatium vinosum DSM 180
* p.value: 0.0163 / 0.0961
Nitrosococcus halophilus Nc4
p.value: 0.9918 / 0.0691
Legionella pneu. 2300/99 Alcoy
p.value: 0.364 / 0.1366
Acidithiobacillus ferrivo. SS3
p.value: 0.3095 / 0.3178
Reinekea blandensis MED297
* p.value: 0.0042 / 0.0485
Methylophaga thiooxyd. DMS010
p.value: 0.5171 / 0.2426
Beggiatoa sp. PS
p.value: 0.0557 / 0.7209
%RP
S S S S F F F F W W W W W P P P P P S S S S

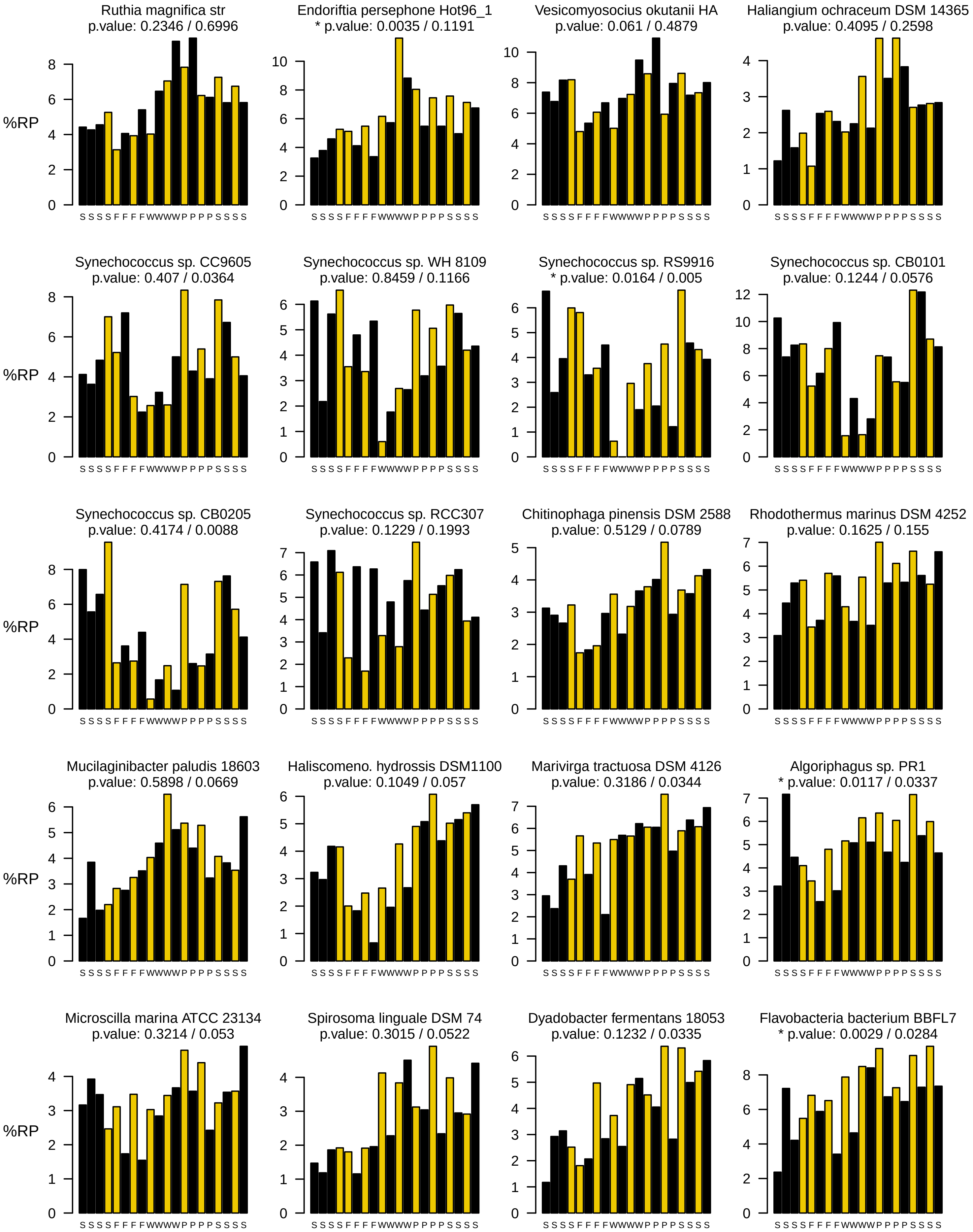
Ruthia magnifica str
p.value: 0.2346 / 0.6996
Endoriftia persephone Hot96_1
* p.value: 0.0035 / 0.1191
Vesicomyosocius okutanii HA
p.value: 0.061 / 0.4879
Haliangium ochraceum DSM 14365
p.value: 0.4095 / 0.2598
Synechococcus sp. CC9605
p.value: 0.407 / 0.0364
Synechococcus sp. WH 8109
p.value: 0.8459 / 0.1166
Synechococcus sp. RS9916
* p.value: 0.0164 / 0.005
Synechococcus sp. CB0101
p.value: 0.1244 / 0.0576
Synechococcus sp. CB0205
p.value: 0.4174 / 0.0088
Synechococcus sp. RCC307
p.value: 0.1229 / 0.1993
Chitinophaga pinensis DSM 2588
p.value: 0.5129 / 0.0789
Rhodothermus marinus DSM 4252
p.value: 0.1625 / 0.155
Mucilaginibacter paludis 18603
p.value: 0.5898 / 0.0669
Haliscomeno. hydrossis DSM1100
p.value: 0.1049 / 0.057
Marivirga tractuosa DSM 4126
p.value: 0.3186 / 0.0344
Algoriphagus sp. PR1
* p.value: 0.0117 / 0.0337
Microscilla marina ATCC 23134
p.value: 0.3214 / 0.053
Spirosoma linguale DSM 74
p.value: 0.3015 / 0.0522
Dyadobacter fermentans 18053
p.value: 0.1232 / 0.0335
Flavobacteria bacterium BBFL7
* p.value: 0.0029 / 0.0284
%RP
S S S S F F F F W W W W P P P P S S S S

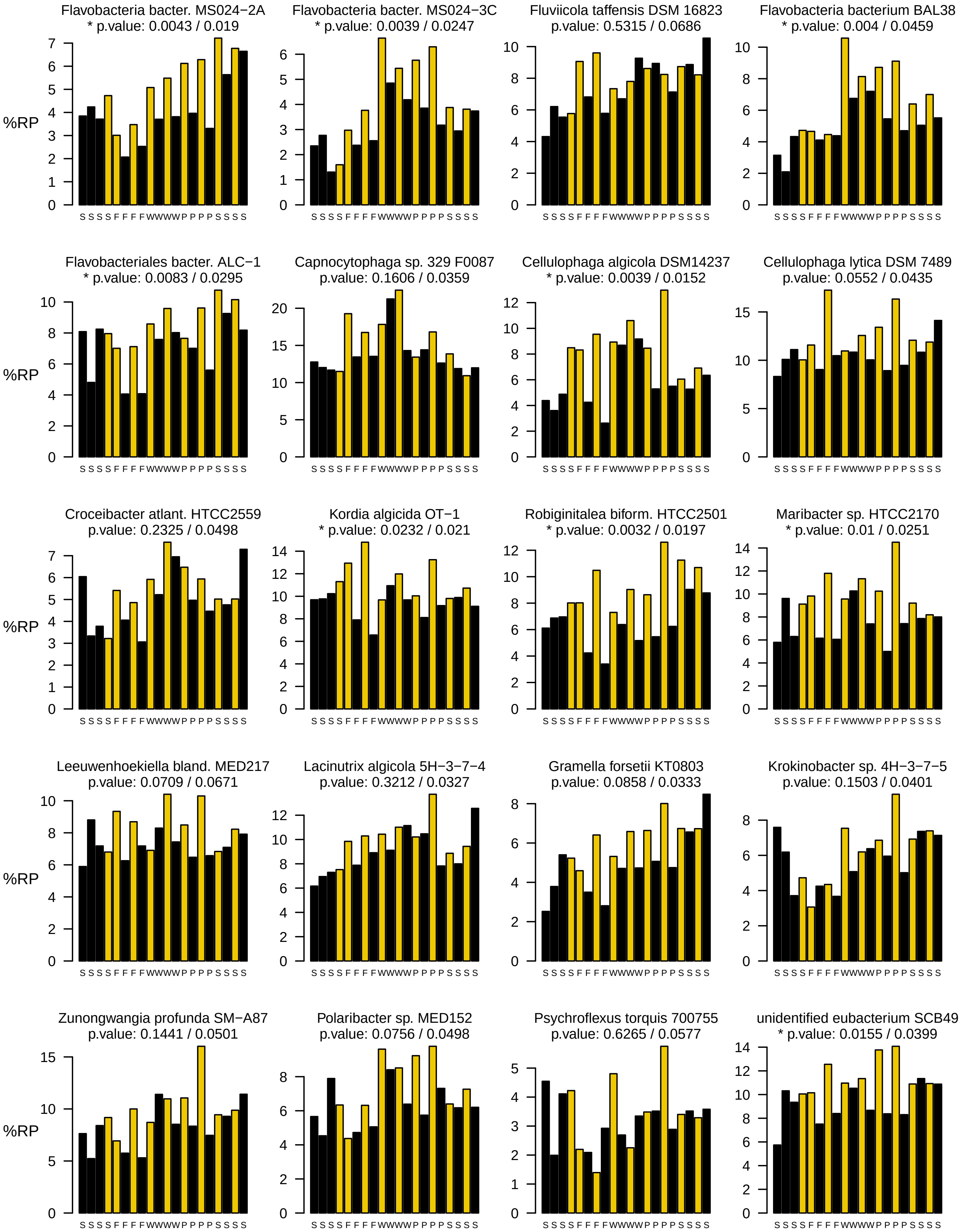
Flavobacteria bacter. MS024−2A
* p.value: 0.0043 / 0.019
Flavobacteria bacter. MS024−3C
* p.value: 0.0039 / 0.0247
Fluviicola taffensis DSM 16823
p.value: 0.5315 / 0.0686
Flavobacteria bacterium BAL38
* p.value: 0.004 / 0.0459
Flavobacteriales bacter. ALC−1
* p.value: 0.0083 / 0.0295
Capnocytophaga sp. 329 F0087
p.value: 0.1606 / 0.0359
Cellulophaga algicola DSM14237
* p.value: 0.0039 / 0.0152
Cellulophaga lytica DSM 7489
p.value: 0.0552 / 0.0435
Croceibacter atlant. HTCC2559
p.value: 0.2325 / 0.0498
Kordia algicida OT−1
* p.value: 0.0232 / 0.021
Robiginitalea biform. HTCC2501
* p.value: 0.0032 / 0.0197
Maribacter sp. HTCC2170
* p.value: 0.01 / 0.0251
Leeuwenhoekiella bland. MED217
p.value: 0.0709 / 0.0671
Lacinutrix algicola 5H−3−7−4
p.value: 0.3212 / 0.0327
Gramella forsetii KT0803
p.value: 0.0858 / 0.0333
Krokinobacter sp. 4H−3−7−5
p.value: 0.1503 / 0.0401
Zunongwangia profunda SM−A87
p.value: 0.1441 / 0.0501
Polaribacter sp. MED152
p.value: 0.0756 / 0.0498
Psychroflexus torquis 700755
p.value: 0.6265 / 0.0577
unidentified eubacterium SCB49
* p.value: 0.0155 / 0.0399
%RP

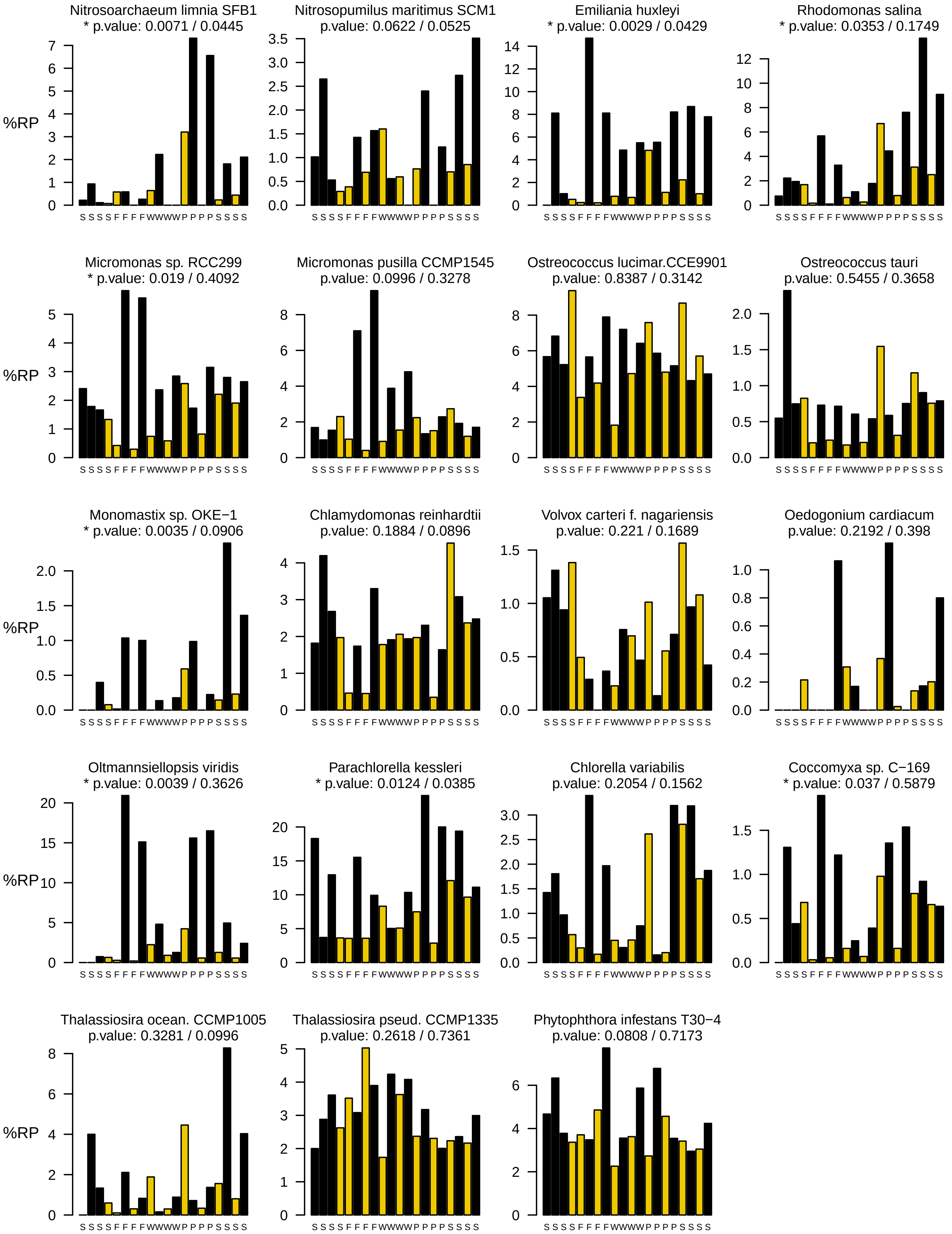
Nitrosoarchaeum limnia SFB1
* p.value: 0.0071 / 0.0445
Nitrosopumilus maritimus SCM1
p.value: 0.0622 / 0.0525
Emiliania huxleyi
* p.value: 0.0029 / 0.0429
Rhodomonas salina
* p.value: 0.0353 / 0.1749
Micromonas sp. RCC299
* p.value: 0.019 / 0.4092
Micromonas pusilla CCMP1545
p.value: 0.0996 / 0.3278
Ostreococcus lucimar.CCE9901
p.value: 0.8387 / 0.3142
Ostreococcus tauri
p.value: 0.5455 / 0.3658
Monomastix sp. OKE−1
* p.value: 0.0035 / 0.0906
Chlamydomonas reinhardtii
p.value: 0.1884 / 0.0896
Volvox carteri f. nagariensis
p.value: 0.221 / 0.1689
Oedogonium cardiacum
p.value: 0.2192 / 0.398
Oltmannsiellopsis viridis
* p.value: 0.0039 / 0.3626
Parachlorella kessleri
* p.value: 0.0124 / 0.0385
Chlorella variabilis
p.value: 0.2054 / 0.1562
Coccomyxa sp. C−169
* p.value: 0.037 / 0.5879
Thalassiosira ocean. CCMP1005
p.value: 0.3281 / 0.0996
Thalassiosira pseud. CCMP1335
p.value: 0.2618 / 0.7361
Phytophthora infestans T30−4
p.value: 0.0808 / 0.7173
%RP
S S S S F F F F W W W W P P P P S S S S

Figure S3

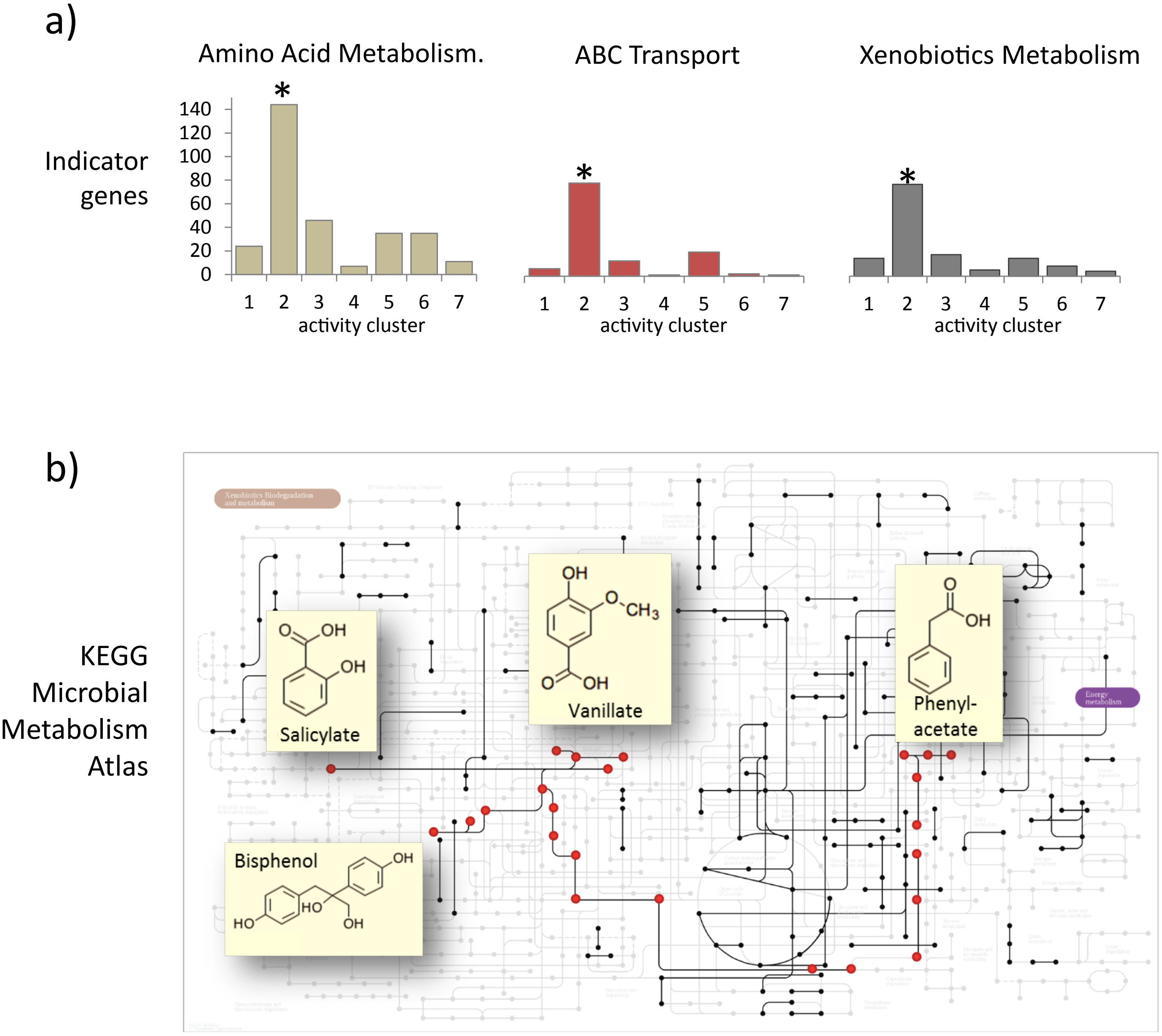

a)
Amino Acid Metabolism.
ABC Transport
Xenobiotics Metabolism
Indicator genes
140
120
100
80
60
40
20
0
*
1 2 3 4 5 6 7
activity cluster
b)
KEGG Microbial Metabolism Atlas
Salicylate
Vanillate
Phenyl-acetate
Bisphenol
OH
O
CH3
HO

Figure S4

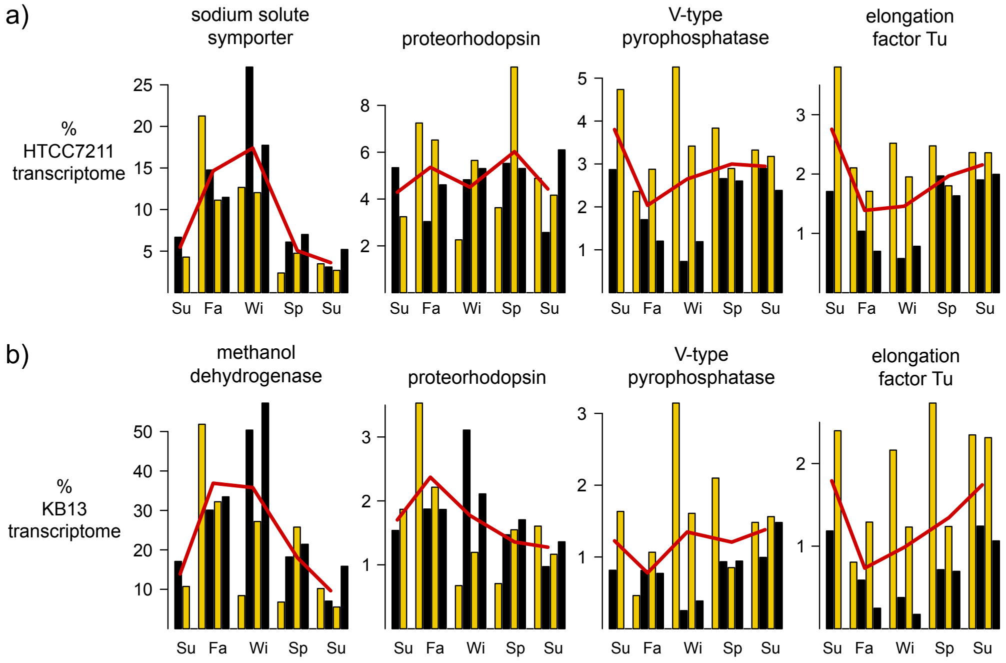

a)
sodium solute symporter
proteorhodopsin
V-type pyrophosphatase
elongation factor Tu
% HTCC7211 transcriptome
25
20
15
10
5
8
6
4
2
5
4
3
2
1
3
2
1
Su Fa Wi Sp Su
Su Fa Wi Sp Su
Su Fa Wi Sp Su
Su Fa Wi Sp Su
b)
methanol dehydrogenase
proteorhodopsin
V-type pyrophosphatase
elongation factor Tu
% KB13 transcriptome
50
40
30
20
10
3
2
1
3
2
1
2
1
Su Fa Wi Sp Su
Su Fa Wi Sp Su
Su Fa Wi Sp Su
Su Fa Wi Sp Su

Figure S5

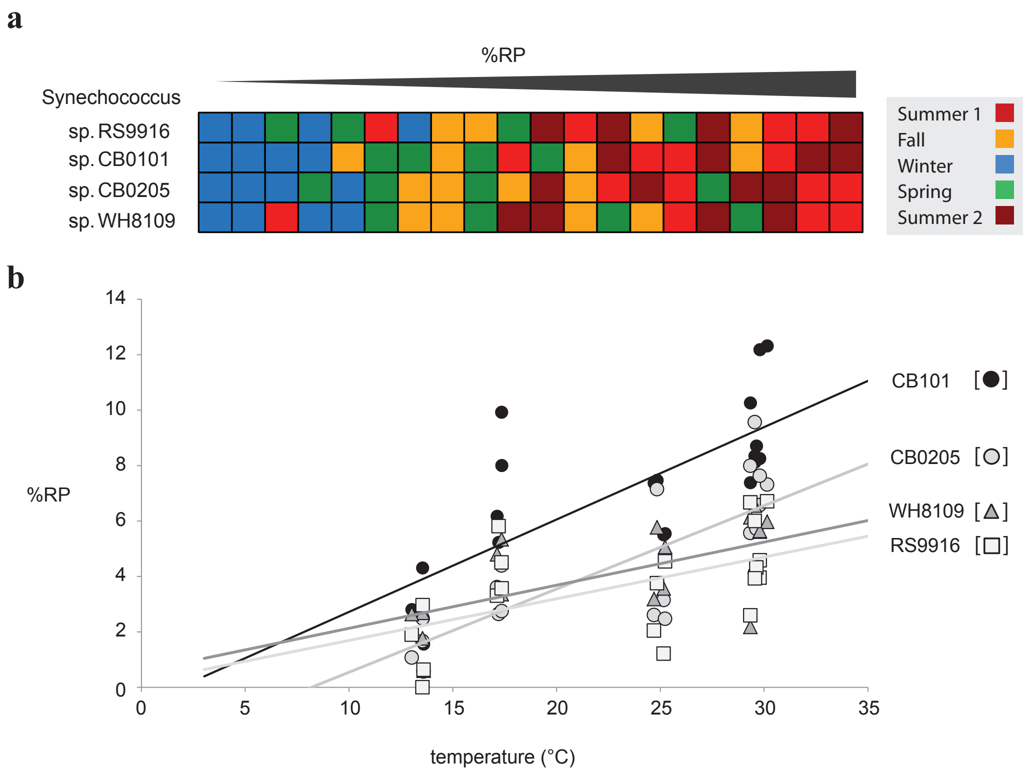
a
%RP
Synechococcus
sp. RS9916
sp. CB0101
sp. CB0205
sp. WH8109
Summer 1
Fall
Winter
Spring
Summer 2
b
%RP
14
12
10
8
6
4
2
0
0
5
10
15
20
25
30
35
temperature (°C)
CB101
CB0205
WH8109
RS9916
