# Supplementary material for: Linking activity and function to ecosystem dynamics in a coastal bacterioplankton community
Source: Front Microbiol. 2014 Apr 24;5:185. doi: 10.3389/fmicb.2014.00185 (PMC4006046; doi:10.3389/fmicb.2014.00185)
Supplement: Supplementary file 2 [file DataSheet2.PDF]

Table S3. KEGG ortholog (KOs) indicator values for the seven RP clusters. "IV.clade.#" = the indicator value for a particular cluster. "p.value.#" is the p value from the random permutation test for a signficancant indicator value in a given cluster.

KEGG.KO.id	IV.clade.1	IV.clade.2	IV.clade.3	IV.clade.4	IV.clade.5	IV.clade.6	IV.clade.7	p.clade.1	p.clade.2	p.clade.3	p.clade.4	p.clade.5	p.clade.6	p.clade.7	KEGG.KO.description
KO:K00001	5.68	7.30	8.28	5.84	2.28	0.30	0.93	2.97E-01	1.63E-01	1.18E-01	2.83E-01	8.16E-01	9.70E-01	9.35E-01	alcohol dehydrogenase [EC:1.1.1.1]
KO:K00002	0.18	0.17	0.00	0.85	0.00	0.00	0.47	4.21E-01	4.40E-01	1.00E+00	1.13E-01	1.00E+00	1.00E+00	2.11E-01	alcohol dehydrogenase (NADP+) [EC:1.1.1.2]
KO:K00003	5.93	8.55	10.83	5.28	6.36	1.61	5.03	5.37E-01	1.73E-01	6.94E-02	6.63E-01	4.50E-01	9.71E-01	7.06E-01	homoserine dehydrogenase [EC:1.1.1.3]
KO:K00005	0.00	0.00	0.00	0.00	30.00	0.00	0.00	1.00E+00	1.00E+00	1.00E+00	1.00E+00	0.00E+00	1.00E+00	1.00E+00	glycerol dehydrogenase [EC:1.1.1.6]
KO:K00007	0.01	1.03	0.00	0.31	0.00	0.00	0.00	3.68E-01	1.14E-01	1.00E+00	2.05E-01	1.00E+00	1.00E+00	1.00E+00	D-arabinitol 4-dehydrogenase [EC:1.1.1.11]
KO:K00008	0.56	14.20	1.06	1.62	0.00	0.00	0.56	7.83E-01	1.20E-02	6.58E-01	5.25E-01	1.00E+00	1.00E+00	7.82E-01	L-iditol 2-dehydrogenase [EC:1.1.1.14]
KO:K00010	0.41	6.10	2.51	0.87	2.51	0.00	0.23	7.49E-01	6.40E-02	2.45E-01	6.08E-01	2.45E-01	1.00E+00	7.97E-01	myo-inositol 2-dehydrogenase [EC:1.1.1.18]
KO:K00011	0.93	0.00	3.70	0.00	1.78	0.00	0.00	1.89E-01	1.00E+00	6.09E-02	1.00E+00	1.25E-01	1.00E+00	1.00E+00	aldehyde reductase [EC:1.1.1.21]
KO:K00012	4.88	3.64	15.74	6.00	11.74	2.97	1.33	6.30E-01	8.35E-01	9.71E-03	3.97E-01	3.39E-02	8.99E-01	9.78E-01	UDPglucose 6-dehydrogenase [EC:1.1.1.22]
KO:K00013	5.63	20.13	5.80	7.65	4.72	0.99	2.65	7.04E-01	7.00E-03	6.75E-01	3.63E-01	8.20E-01	9.91E-01	9.51E-01	histidinol dehydrogenase [EC:1.1.1.23]
KO:K00014	4.05	4.68	5.84	3.23	2.70	5.45	2.23	4.41E-01	2.95E-01	1.40E-01	6.51E-01	7.74E-01	2.31E-01	8.52E-01	shikimate dehydrogenase [EC:1.1.1.25]
KO:K00015	2.73	12.40	0.00	6.94	0.43	0.00	0.09	4.81E-01	2.10E-02	1.00E+00	9.01E-02	8.90E-01	1.00E+00	9.37E-01	glyoxylate reductase [EC:1.1.1.26]
KO:K00016	0.00	0.06	31.46	0.07	0.00	0.00	0.00	1.00E+00	5.01E-01	1.43E-04	4.97E-01	1.00E+00	1.00E+00	1.00E+00	L-lactate dehydrogenase [EC:1.1.1.27]
KO:K00018	1.01	0.05	1.65	0.47	0.26	11.74	0.03	3.34E-01	7.75E-01	2.09E-01	5.24E-01	6.29E-01	1.01E-02	7.83E-01	glycerate dehydrogenase [EC:1.1.1.29]
KO:K00019	1.58	8.42	0.00	2.57	0.00	0.07	0.00	3.93E-01	2.19E-02	1.00E+00	1.97E-01	1.00E+00	9.03E-01	1.00E+00	3-hydroxybutyrate dehydrogenase [EC:1.1.1.3]
KO:K00020	5.08	25.91	0.20	7.04	2.61	0.97	0.62	5.61E-01	4.14E-03	9.78E-01	2.92E-01	8.58E-01	9.48E-01	9.62E-01	3-hydroxyisobutyrate dehydrogenase [EC:1.1.1.31]
KO:K00021	6.00	0.17	0.00	0.09	0.00	0.00	0.00	3.61E-02	4.74E-01	1.00E+00	5.27E-01	1.00E+00	1.00E+00	1.00E+00	3-hydroxy-3-methylglutaryl-CoA reductase [EC:1.1.1.34]
KO:K00022	8.80	4.23	0.00	2.86	0.00	5.99	0.01	4.40E-02	2.16E-01	1.00E+00	3.95E-01	1.00E+00	1.11E-01	9.42E-01	3-hydroxyacyl-CoA dehydrogenase [EC:1.1.1.35]
KO:K00023	2.45	17.69	0.29	4.60	0.01	2.07	0.10	6.62E-01	7.57E-03	9.50E-01	2.70E-01	9.77E-01	7.36E-01	9.65E-01	acetoacetyl-CoA reductase [EC:1.1.1.36]
KO:K00024	3.24	8.54	13.29	4.50	0.47	0.00	0.03	5.38E-01	8.16E-02	2.99E-02	3.36E-01	9.19E-01	1.00E+00	9.51E-01	malate dehydrogenase [EC:1.1.1.37]
KO:K00025	1.81	6.99	1.24	4.18	0.00	0.00	0.01	4.42E-01	6.30E-02	5.84E-01	1.56E-01	1.00E+00	1.00E+00	9.05E-01	malate dehydrogenase [EC:1.1.1.37]
KO:K00026	6.15	3.87	1.66	3.78	1.28	12.65	1.48	2.09E-01	4.99E-01	8.52E-01	5.14E-01	8.88E-01	3.77E-02	8.71E-01	malate dehydrogenase [EC:1.1.1.37]
KO:K00027	0.57	0.03	4.12	0.03	0.04	1.17	3.09	3.75E-01	7.05E-01	4.99E-02	7.05E-01	6.91E-01	2.13E-01	7.01E-02	malate dehydrogenase (oxaloacetate-decarboxylating) [EC:1.1.1.38]
KO:K00028	0.02	8.84	0.00	0.09	0.00	0.00	0.00	5.99E-01	2.07E-02	1.00E+00	5.48E-01	1.00E+00	1.00E+00	1.00E+00	malate dehydrogenase (decarboxylating) [EC:1.1.1.39]
KO:K00029	7.48	14.38	0.04	6.83	0.00	3.68	0.47	1.33E-01	1.67E-02	9.87E-01	1.74E-01	1.00E+00	6.25E-01	9.69E-01	malate dehydrogenase (oxaloacetate-decarboxylating)(NADP+)
KO:K00030	0.08	0.00	6.88	0.11	4.78	2.66	0.00	6.48E-01	7.38E-01	2.11E-02	6.20E-01	3.90E-02	8.69E-02	7.37E-01	isocitrate dehydrogenase (NAD+) [EC:1.1.1.41]
KO:K00031	11.16	15.14	10.87	7.33	0.72	4.86	3.65	1.23E-01	2.31E-02	1.38E-01	6.28E-01	9.97E-01	9.06E-01	9.59E-01	isocitrate dehydrogenase [EC:1.1.1.42]
KO:K00033	1.06	0.10	21.37	0.64	18.11	0.00	0.71	6.73E-01	9.14E-01	2.43E-03	7.93E-01	4.43E-03	1.00E+00	7.77E-01	6-phosphogluconate dehydrogenase [EC:1.1.1.44]
KO:K00034	0.26	0.26	3.64	0.73	5.10	0.00	0.02	6.32E-01	6.36E-01	6.50E-02	3.92E-01	3.64E-02	1.00E+00	8.15E-01	glucose 1-dehydrogenase [EC:1.1.1.47]
KO:K00035	0.01	0.55	0.00	0.04	0.00	0.00	0.00	4.07E-01	6.14E-02	1.00E+00	3.36E-01	1.00E+00	1.00E+00	1.00E+00	D-galactose 1-dehydrogenase [EC:1.1.1.48]
KO:K00036	4.17	11.05	10.86	1.19	4.97	0.00	0.71	3.98E-01	2.49E-02	2.64E-02	9.23E-01	2.59E-01	1.00E+00	9.56E-01	glucose-6-phosphate 1-dehydrogenase [EC:1.1.1.49]
KO:K00037	0.25	5.09	0.00	0.00	0.00	0.00	0.00	3.34E-01	5.60E-02	1.00E+00	1.00E+00	1.00E+00	1.00E+00	1.00E+00	3-alpha-hydroxysteroid dehydrogenase [EC:1.1.1.5]
KO:K00038	2.08	0.05	0.00	0.13	0.00	0.00	0.00	6.64E-02	3.82E-01	1.00E+00	3.21E-01	1.00E+00	1.00E+00	1.00E+00	3alpha(or 2beta)-hydroxysteroid dehydrogenase [EC:1.1.1.53]
KO:K00039	0.01	1.11	0.00	0.20	0.00	0.00	0.00	6.11E-01	4.50E-02	1.00E+00	2.55E-01	1.00E+00	1.00E+00	1.00E+00	ribitol 2-dehydrogenase [EC:1.1.1.56]
KO:K00040	2.11	2.16	0.00	0.25	0.00	0.00	0.00	9.23E-02	9.01E-02	1.00E+00	5.40E-01	1.00E+00	1.00E+00	1.00E+00	fructuronate reductase [EC:1.1.1.57]
KO:K00041	0.42	0.00	0.15	0.06	0.00	0.00	0.07	1.20E-01	5.35E-01	2.49E-01	3.65E-01	1.00E+00	1.00E+00	3.59E-01	tagaturonate reductase [EC:1.1.1.58]
KO:K00042	1.58	2.47	0.50	2.71	2.99	0.00	0.46	4.35E-01	2.60E-01	7.54E-01	2.28E-01	1.98E-01	1.00E+00	7.66E-01	2-hydroxy-3-oxopropionate reductase [EC:1.1.1.6]
KO:K00043	0.46	10.85	0.00	0.23	0.00	0.00	0.00	5.57E-01	1.30E-02	1.00E+00	6.58E-01	1.00E+00	1.00E+00	1.00E+00	4-hydroxybutyrate dehydrogenase [EC:1.1.1.61]
KO:K00044	0.03	0.00	0.00	0.22	0.00	0.00	0.00	2.20E-01	1.00E+00	1.00E+00	1.03E-01	1.00E+00	1.00E+00	1.00E+00	estradiol 17beta-dehydrogenase [EC:1.1.1.62]
KO:K00045	1.44	12.72	0.00	2.05	0.00	0.00	0.00	4.86E-01	1.04E-02	1.00E+00	3.41E-01	1.00E+00	1.00E+00	1.00E+00	mannitol 2-dehydrogenase [EC:1.1.1.67]
KO:K00046	0.41	8.71	9.24	3.01	0.63	0.00	0.50	8.78E-01	4.31E-02	3.91E-02	3.64E-01	8.46E-01	1.00E+00	8.65E-01	gluconate 5-dehydrogenase [EC:1.1.1.69]
KO:K00050	0.62	11.96	0.03	1.30	0.01	0.50	0.17	7.45E-01	9.14E-03	9.44E-01	5.09E-01	9.55E-01	7.82E-01	8.80E-01	hydroxypyruvate reductase [EC:1.1.1.81]
KO:K00051	0.00	0.19	0.00	1.53	0.00	0.00	0.00	1.00E+00	1.98E-01	1.00E+00	1.30E-01	1.00E+00	1.00E+00	1.00E+00	malate dehydrogenase (NADP+) [EC:1.1.1.82]
KO:K00052	5.09	11.70	11.16	7.77	3.55	1.08	4.55	7.22E-01	6.37E-02	7.63E-02	2.79E-01	8.95E-01	9.86E-01	7.98E-01	3-isopropylmalate dehydrogenase [EC:1.1.1.85]
KO:K00053	7.05	9.82	8.88	9.36	8.14	4.83	8.02	7.34E-01	2.46E-01	3.87E-01	3.10E-01	5.24E-01	9.27E-01	5.49E-01	ketol-acid reductoisomerase [EC:1.1.1.86]
KO:K00054	2.02	3.13	0.00	0.93	0.00	3.25	0.27	2.58E-01	1.47E-01	1.00E+00	5.23E-01	1.00E+00	1.41E-01	7.69E-01	hydroxymethylglutaryl-CoA reductase [EC:1.1.1.88]
KO:K00055	0.55	0.00	0.00	1.95	0.00	0.00	0.00	2.00E-01	5.40E-01	1.00E+00	8.37E-02	1.00E+00	1.00E+00	1.00E+00	aryl-alcohol dehydrogenase [EC:1.1.1.9]
KO:K00057	7.58	4.55	2.69	2.58	2.22	2.96	1.42	6.24E-02	2.82E-01	7.14E-01	7.37E-01	8.12E-01	6.46E-01	9.11E-01	glycerol-3-phosphate dehydrogenase (NAD(P)+) [EC:1.1.1.94]
KO:K00058	8.23	7.40	16.41	6.45	10.42	2.40	3.95	3.34E-01	4.74E-01	1.26E-02	6.49E-01	1.21E-01	9.75E-01	9.17E-01	D-3-phosphoglycerate dehydrogenase [EC:1.1.1.95]
KO:K00059	13.23	16.28	14.05	13.89	2.56	2.79	4.27	1.41E-01	5.34E-02	1.04E-01	1.10E-01	9.90E-01	9.89E-01	9.60E-01	3-oxoacyl-[acyl-carrier protein] reductase [EC:1.1.1.1]
KO:K00060	1.13	1.47	1.12	1.50	1.12	0.00	0.08	3.59E-01	2.64E-01	3.63E-01	2.57E-01	3.63E-01	1.00E+00	8.61E-01	threonine 3-dehydrogenase [EC:1.1.1.13]
KO:K00064	0.12	6.40	0.17	0.44	0.00	0.00	0.13	6.38E-01	3.34E-02	6.09E-01	4.61E-01	1.00E+00	1.00E+00	6.34E-01	D-threo-aldose 1-dehydrogenase [EC:1.1.1.122]
KO:K00065	6.12	7.72	1.19	0.69	0.05	0.00	0.00	8.83E-02	5.91E-02	6.03E-01	7.32E-01	8.71E-01	1.00E+00	1.00E+00	2-deoxy-D-gluconate 3-dehydrogenase [EC:1.1.1.125]
KO:K00066	0.16	0.09	0.00	0.59	0.00	0.00	0.00	2.85E-01	3.56E-01	1.00E+00	9.67E-02	1.00E+00	1.00E+00	1.00E+00	GDP-mannose 6-dehydrogenase [EC:1.1.1.132]
KO:K00067	2.56	1.80	5.62	1.63	6.33	0.09	1.52	3.84E-01	6.08E-01	6.97E-02	6.64E-01	5.26E-02	9.80E-01	6.97E-01	dTDP-4-dehydrorhamnose reductase [EC:1.1.1.133]
KO:K00071	0.12	0.00	0.00	0.00	0.00	0.00	0.00	6.01E-02	1.00E+00	1.00E+00	1.00E+00	1.00E+00	1.00E+00	1.00E+00	11beta-hydroxysteroid dehydrogenase [EC:1.1.1.146]
KO:K00073	0.00	0.00	0.00	0.20	0.00	0.00	0.00	1.00E+00	1.00E+00	1.00E+00	1.43E-01	1.00E+00	1.00E+00	1.00E+00	ureidoglycolate dehydrogenase [EC:1.1.1.154]
KO:K00074	1.05	10.94	0.00	7.40	0.00	2.61	0.00	8.27E-01	1.77E-02	1.00E+00	5.39E-02	1.00E+00	4.34E-01	1.00E+00	3-hydroxybutyryl-CoA dehydrogenase [EC:1.1.1.157]
KO:K00075	3.09	5.31	1.27	4.28	3.10	8.24	0.78	6.07E-01	1.53E-01	9.33E-01	3.02E-01	6.03E-01	3.76E-02	9.67E-01	UDP-N-acetylmuramate dehydrogenase [EC:1.1.1.158]
KO:K00076	2.78	1.25	0.00	0.00	0.00	0.00	0.00	7.93E-02	1.65E-01	1.00E+00	1.00E+00	1.00E+00	1.00E+00	1.00E+00	7-alpha-hydroxysteroid dehydrogenase [EC:1.1.1.159]
KO:K00077	1.98	2.54	9.26	1.91	1.43	0.00	0.02	4.05E-01	3.08E-01	3.51E-02	4.19E-01	5.29E-01	1.00E+00	9.17E-01	2-dehydropantoate 2-reductase [EC:1.1.1.169]
KO:K00079	0.61	0.01	0.00	3.77	0.00	0.00	0.00	2.76E-01	6.37E-01	1.00E+00	4.39E-02	1.00E+00	1.00E+00	1.00E+00	carbonyl reductase (NADPH) [EC:1.1.1.184]
KO:K00082	0.00	0.00	0.00	0.00	10.13	0.00	0.13	1.00E+00	1.00E+00	1.00E+00	1.00E+00	1.43E-04	1.00E+00	3.17E-01	5-amino-6-(5-phosphoribosylamino)uracil reductase [EC:1.1.1.193]
KO:K00087	1.96	18.43	0.00	7.53	0.00	0.00	0.37	6.61E-01	1.43E-02	1.00E+00	1.14E-01	1.00E+00	1.00E+00	8.90E-01	xanthine dehydrogenase molybdenum-binding subunit [EC:1.17.1.4]
KO:K00088	10.51	11.12	13.89	6.74	3.82	7.23	8.36	2.20E-01	1.60E-01	4.74E-02	8.42E-01	9.72E-01	7.87E-01	5.90E-01	IMP dehydrogenase [EC:1.1.1.25]
KO:K00090	0.07	2.54	0.00	0.51	0.00	0.00	0.00	5.39E-01	4.81E-02	1.00E+00	2.71E-01	1.00E+00	1.00E+00	1.00E+00	gluconate 2-dehydrogenase [EC:1.1.1.215]
KO:K00091	0.18	0.88	0.64	5.91	0.00	0.00	0.58	7.22E-01	4.32E-01	5.10E-01	4.63E-02	1.00E+00	1.00E+00	5.35E-01	dihydroflavonol-4-reductase [EC:1.1.1.219]
KO:K00096	0.00	0.40	0.00	0.44	0.00	0.00	1.39	1.00E+00	2.34E-01	1.00E+00	2.26E-01	1.00E+00	1.00E+00	9.91E-02	glycerol-1-phosphate dehydrogenase [NAD(P)] [EC:1.1.1.261]
KO:K00097	5.00	4.06	2.09	4.06	4.14	0.73	0.93	1.61E-01	2.82E-01	7.70E-01	2.81E-01	2.67E-01	9.58E-01	9.41E-01	4-hydroxythreonine-4-phosphate dehydrogenase [EC:1.1.1.262]
KO:K00098	0.06	1.69	0.00	2.83	0.00	0.00	0.00	6.45E-01	1.38E-01	1.00E+00	7.51E-02	1.00E+00	1.00E+00	1.00E+00	L-idonate 5-dehydrogenase [EC:1.1.1.264]
KO:K00099	4.70	2.62	8.49	3.05	5.18	1.78	2.79	2.74E-01	7.61E-01	3.96E-02	6.63E-01	2.05E-01	8.82E-01	7.24E-01	1-deoxy-D-xylulose-5-phosphate reductoisomerase [EC:1.1.1.267]
KO:K00100	5.08	6.90	13.66	7.97	2.76	0.43	3.70	5.71E-01	3.18E-01	4.63E-02	2.17E-01	8.65E-01	9.86E-01	7.70E-01	
KO:K00101	2.22	35.65	0.00	2.26	0.00	0.00	0.55	7.13E-01	4.29E-04	1.00E+00	7.04E-01	1.00E+00	1.00E+00	9.20E-01	L-lactate dehydrogenase (cytochrome) [EC:1.1.2.3]
KO:K00102	0.86	8.04	0.00	12.29	0.00	0.00	0.19	7.87E-01	5.63E-02	1.00E+00	2.00E-02	1.00E+00	1.00E+00	9.05E-01	D-lactate dehydrogenase (cytochrome) [EC:1.1.2.4]
KO:K00103	0.00	0.00	0.00	0.92	0.00	0.00	0.00	1.00E+00	1.00E+00	1.00E+00	1.43E-01	1.00E+00	1.00E+00	1.00E+00	L-gulonolactone oxidase [EC:1.1.3.8]
KO:K00104	2.99	10.51	4.65	5.54	4.58	3.30	5.97	8.55E-01	8.46E-02	6.35E-01	4.91E-01	6.49E-01	8.27E-01	4.21E-01	glycolate oxidase [EC:1.1.3.15]
KO:K00105	6.11	0.35	0.00	0.26	0.00	0.00	0.00	4.49E-02	4.02E-01	1.00E+00	4.25E-01	1.00E+00	1.00E+00	1.00E+00	alpha-glycerophosphate oxidase [EC:1.1.3.21]
KO:K00106	0.00	0.00	0.00	0.00	4.38	0.00	0.00	1.00E+00	1.00E+00	1.00E+00	1.00E+00	1.60E-02	1.00E+00	1.00E+00	xanthine dehydrogenase/oxidase [EC:1.17.1.4 1.17.3.2]
KO:K00108	2.83	23.91	0.00	7.76	0.00	0.28	0.26	6.45E-01	3.43E-03	1.00E+00	1.18E-01	1.00E+00	9.44E-01	9.45E-01	choline dehydrogenase [EC:1.1.99.1]
KO:K00111	4.21	23.52	0.51	3.07	0.97	0.09	0.16	4.19E-01	3.71E-03	9.49E-01	6.25E-01	9.15E-01	9.80E-01	9.75E-01	glycerol-3-phosphate dehydrogenase [EC:1.1.5.3]
KO:K00113	0.00	0.00	0.00	5.92	0.00	0.00	0.00	1.00E+00	1.00E+00	1.00E+00	5.03E-02	1.00E+00	1.00E+00	1.00E+00	glycerol-3-phosphate dehydrogenase subunit C [EC:1.1.5.3]
KO:K00114	6.94	1.33	4.84	4.07	1.87	0.20	1.90	1.40E-01	7.57E-01	2.53E-01	3.32E-01	6.63E-01	9.04E-01	6.59E-01	alcohol dehydrogenase (cytochrome c) [EC:1.1.2.8]
KO:K00115	0.00	0.00	0.00	1.94	0.00	0.00	0.00	1.00E+00	1.00E+00	1.00E+00	1.43E-01	1.00E+00	1.00E+00	1.00E+00	glucose dehydrogenase (acceptor) [EC:1.1.99.1]
KO:K00116	0.90	0.02	4.17	0.22	6.28	0.00	0.38	3.40E-01	7.88E-01	6.03E-02	6.22E-01	2.91E-02	1.00E+00	5.34E-01	malate dehydrogenase (quinone) [EC:1.1.5.4]
KO:K00117	2.81	0.45	16.32	2.61	1.58	0.61	3.37	4.43E-01	8.67E-01	1.36E-02	4.71E-01	6.74E-01	8.47E-01	3.62E-01	quinoprotein glucose dehydrogenase [EC:1.1.5.2]
KO:K00118	0.09	2.30	0.18	0.39	0.00	0.00	0.00	4.64E-01	8.23E-02	3.90E-01	3.05E-01	1.00E+00	1.00E+00	1.00E+00	glucose-fructose oxidoreductase [EC:1.1.99.28]
KO:K00119	1.14	10.38	0.87	4.80	1.27	0.00	0.01	7.51E-01	3.17E-02	8.04E-01	1.59E-01	7.19E-01	1.00E+00	9.54E-01	
KO:K00120	1.54	0.45	0.00	2.43	0.00	0.00	0.00	1.94E-01	3.87E-01	1.00E+00	1.27E-01	1.00E+00	1.00E+00	1.00E+00	
KO:K00121	7.38	13.66	1.80	5.68	0.00	0.00	0.83	1.26E-01	2.57E-02	8.43E-01	2.42E-01	1.00E+00	1.00E+00	9.29E-01	S-(hydroxymethyl)glutathione dehydrogenase / alcohol dehydrogenase
KO:K00122	3.16	12.64	2.19	0.18	0.00	0.00	0.27	2.66E-01	2.16E-02	3.90E-01	8.11E-01	1.00E+00	1.00E+00	8.00E-01	formate dehydrogenase [EC:1.2.1.2]

KO:K00123	4.12	27.34	4.64	7.45	0.36	4.60	0.15	7.91E-01	2.57E-03	7.32E-01	3.54E-01	9.80E-01	7.37E-01	9.86E-01	formate dehydrogenase, alpha subunit [EC:1.2.1.2]
KO:K00124	2.66	15.37	0.16	3.51	0.00	5.23	0.00	5.50E-01	1.84E-02	9.17E-01	4.13E-01	1.00E+00	2.22E-01	1.00E+00	formate dehydrogenase, beta subunit [EC:1.2.1.2]
KO:K00126	0.28	0.10	0.00	0.85	0.00	0.00	0.00	2.40E-01	3.80E-01	1.00E+00	1.03E-01	1.00E+00	1.00E+00	1.00E+00	formate dehydrogenase, delta subunit [EC:1.2.1.2]
KO:K00127	1.52	18.89	0.71	3.60	0.00	9.48	0.00	8.07E-01	1.10E-02	8.93E-01	4.79E-01	1.00E+00	6.96E-02	1.00E+00	formate dehydrogenase, gamma subunit [EC:1.2.1.2]
KO:K00128	8.14	29.38	3.71	10.61	2.15	1.88	0.21	4.44E-01	5.71E-04	9.17E-01	1.93E-01	9.68E-01	9.74E-01	9.95E-01	aldehyde dehydrogenase (NAD+) [EC:1.2.1.3]
KO:K00129	0.22	0.00	0.00	0.27	0.00	0.00	0.00	1.55E-01	1.00E+00	1.00E+00	1.47E-01	1.00E+00	1.00E+00	1.00E+00	aldehyde dehydrogenase (NAD(P)+) [EC:1.2.1.5]
KO:K00130	1.25	18.58	0.00	3.52	0.00	0.04	0.01	6.66E-01	8.71E-03	9.49E-01	2.65E-01	1.00E+00	9.18E-01	9.47E-01	betaine-aldehyde dehydrogenase [EC:1.2.1.8]
KO:K00131	1.42	2.02	0.00	0.48	0.00	6.54	0.00	2.95E-01	2.19E-01	1.00E+00	5.07E-01	1.00E+00	4.90E-02	1.00E+00	glyceraldehyde-3-phosphate dehydrogenase (NADP) [EC:1.2.1.9]
KO:K00133	6.84	9.41	4.66	4.71	6.17	5.76	5.76	3.90E-01	1.11E-01	8.08E-01	8.01E-01	5.31E-01	6.18E-01	6.18E-01	aspartate-semialdehyde dehydrogenase [EC:1.2.1.11]
KO:K00134	12.14	15.17	5.90	7.65	4.29	7.93	4.63	1.24E-01	3.54E-02	8.93E-01	7.19E-01	9.60E-01	6.77E-01	9.49E-01	glyceraldehyde 3-phosphate dehydrogenase [EC:1.2.1.12]
KO:K00135	5.73	14.60	0.07	5.68	0.04	0.92	1.61	2.30E-01	1.96E-02	9.82E-01	2.35E-01	9.85E-01	9.21E-01	8.65E-01	succinate-semialdehyde dehydrogenase (NADP+) [EC:1.2.1.16]
KO:K00136	0.00	0.00	0.00	1.02	0.00	0.00	0.00	1.00E+00	1.00E+00	1.00E+00	1.43E-01	1.00E+00	1.00E+00	1.00E+00	malonate-semialdehyde dehydrogenase (acetylating) [EC:1.2.1.18]
KO:K00137	0.00	3.22	0.00	0.00	0.00	0.00	0.00	1.00E+00	7.04E-02	1.00E+00	1.00E+00	1.00E+00	1.00E+00	1.00E+00	aminobutyraldehyde dehydrogenase [EC:1.2.1.19]
KO:K00139	0.00	0.44	0.00	0.00	0.00	0.00	0.00	1.00E+00	1.00E-01	1.00E+00	1.00E+00	1.00E+00	1.00E+00	1.00E+00	succinate-semialdehyde dehydrogenase [EC:1.2.1.24]
KO:K00140	4.19	21.66	0.99	4.81	0.03	0.00	0.73	4.51E-01	4.71E-03	9.14E-01	3.53E-01	9.85E-01	1.00E+00	9.34E-01	methylmalonate-semialdehyde dehydrogenase [EC:1.2.1.27]
KO:K00141	0.00	0.33	0.00	0.00	0.00	0.00	0.00	1.00E+00	9.99E-02	1.00E+00	1.00E+00	1.00E+00	1.00E+00	1.00E+00	benzaldehyde dehydrogenase (NAD) [EC:1.2.1.28]
KO:K00145	5.66	6.39	4.50	4.61	6.61	0.96	6.54	3.75E-01	2.55E-01	6.18E-01	5.92E-01	2.31E-01	9.77E-01	2.38E-01	N-acetyl-gamma-glutamyl-phosphate reductase [EC:1.2.1.38]
KO:K00146	0.00	0.00	0.00	0.10	0.00	0.00	0.00	1.00E+00	1.00E+00	1.00E+00	1.43E-01	1.00E+00	1.00E+00	1.00E+00	phenylacetaldehyde dehydrogenase [EC:1.2.1.39]
KO:K00147	6.18	5.71	5.37	5.19	7.05	1.02	2.84	2.47E-01	3.23E-01	3.88E-01	4.28E-01	1.56E-01	9.79E-01	8.75E-01	glutamate-5-semialdehyde dehydrogenase [EC:1.2.1.41]
KO:K00148	0.49	0.25	1.37	0.09	0.00	0.00	0.00	2.26E-01	3.24E-01	9.79E-02	4.30E-01	1.00E+00	1.00E+00	1.00E+00	glutathione-independent formaldehyde dehydrogenase [EC:1.2.1.46]
KO:K00150	0.00	0.00	0.00	0.00	38.70	0.00	0.14	1.00E+00	5.75E-01	1.00E+00	1.00E+00	0.00E+00	1.00E+00	4.55E-01	glyceraldehyde-3-phosphate dehydrogenase (NAD(P)) [EC:1.2.1.59]
KO:K00151	2.38	6.06	0.00	1.91	0.00	0.00	0.00	1.89E-01	4.37E-02	1.00E+00	2.59E-01	1.00E+00	1.00E+00	1.00E+00	5-carboxymethyl-2-hydroxymuconic-semialdehyde dehydrogenase
KO:K00152	0.00	0.00	0.00	0.10	0.00	0.00	0.00	1.00E+00	1.00E+00	1.00E+00	1.43E-01	1.00E+00	1.00E+00	1.00E+00	salicylaldehyde dehydrogenase [EC:1.2.1.65]
KO:K00153	1.36	8.59	0.00	0.00	0.00	0.00	0.00	2.66E-01	2.53E-02	1.00E+00	1.00E+00	1.00E+00	1.00E+00	1.00E+00	S-(hydroxymethyl)mycothiol dehydrogenase [EC:1.1.1.36]
KO:K00154	12.56	0.07	0.01	0.73	0.00	0.00	0.08	1.94E-02	7.30E-01	7.87E-01	4.51E-01	1.00E+00	1.00E+00	7.20E-01	coniferyl-aldehyde dehydrogenase [EC:1.2.1.68]
KO:K00155	2.15	13.83	1.53	1.75	0.00	0.00	0.00	4.34E-01	1.23E-02	6.03E-01	5.36E-01	1.00E+00	1.00E+00	9.53E-01	
KO:K00156	0.00	0.11	0.00	0.00	0.00	0.00	0.00	1.00E+00	1.02E-01	1.00E+00	1.00E+00	1.00E+00	1.00E+00	1.00E+00	pyruvate dehydrogenase (cytochrome) [EC:1.2.2.2]
KO:K00157	0.24	10.52	0.00	0.99	0.00	0.00	0.00	6.24E-01	3.00E-02	1.00E+00	4.17E-01	1.00E+00	1.00E+00	1.00E+00	aldehyde oxidase [EC:1.2.3.1]
KO:K00161	4.77	18.75	5.27	5.09	7.28	2.14	3.11	7.82E-01	1.31E-02	7.20E-01	7.43E-01	4.29E-01	9.47E-01	9.04E-01	pyruvate dehydrogenase E1 component subunit alpha [EC:1.2.4.1]
KO:K00162	5.55	16.96	4.52	5.62	5.07	2.05	2.89	6.49E-01	1.50E-02	7.87E-01	6.36E-01	7.18E-01	9.49E-01	9.11E-01	pyruvate dehydrogenase E1 component subunit beta [EC:1.2.4.1]
KO:K00163	10.29	2.33	8.13	7.21	1.51	4.46	3.82	9.59E-02	8.81E-01	1.83E-01	2.51E-01	9.26E-01	6.16E-01	7.19E-01	pyruvate dehydrogenase E1 component [EC:1.2.4.1]
KO:K00164	12.96	15.88	8.81	9.19	1.14	7.12	1.90	7.23E-02	1.97E-02	4.65E-01	3.97E-01	9.96E-01	7.59E-01	9.90E-01	2-oxoglutarate dehydrogenase E1 component [EC:1.2.4.2]
KO:K00166	1.25	0.02	0.86	0.28	0.00	0.00	0.00	8.30E-02	6.75E-01	1.28E-01	3.35E-01	1.00E+00	1.00E+00	1.00E+00	2-oxoisovalerate dehydrogenase E1 component, alpha subunit
KO:K00167	1.10	0.25	0.07	0.39	0.00	0.00	0.00	9.63E-02	3.59E-01	5.93E-01	2.64E-01	1.00E+00	1.00E+00	1.00E+00	2-oxoisovalerate dehydrogenase E1 component, beta subunit
KO:K00174	0.87	0.01	10.08	1.62	2.84	7.00	7.71	7.21E-01	8.50E-01	3.81E-02	5.37E-01	3.41E-01	8.89E-02	7.50E-02	2-oxoglutarate ferredoxin oxidoreductase subunit alpha [EC:1.2.7.3]
KO:K00175	0.64	0.01	10.83	1.71	2.36	6.18	3.48	7.28E-01	8.50E-01	2.16E-02	4.55E-01	3.41E-01	8.34E-02	2.16E-01	2-oxoglutarate ferredoxin oxidoreductase subunit beta [EC:1.2.7.3]
KO:K00179	1.47	2.86	0.00	0.71	0.00	0.00	0.00	2.09E-01	1.08E-01	1.00E+00	3.50E-01	1.00E+00	1.00E+00	1.00E+00	indolepyruvate ferredoxin oxidoreductase, alpha subunit [EC:1.2.7.8]
KO:K00180	1.73	1.53	0.00	0.11	0.00	0.00	0.00	1.07E-01	1.24E-01	1.00E+00	5.15E-01	1.00E+00	1.00E+00	1.00E+00	indolepyruvate ferredoxin oxidoreductase, beta subunit [EC:1.2.7.8]
KO:K00183	0.94	29.50	0.00	1.61	0.00	2.50	0.05	8.26E-01	2.14E-03	1.00E+00	7.14E-01	1.00E+00	5.56E-01	9.17E-01	molybdopterin oxidoreductase, molybdopterin binding subunit
KO:K00184	1.62	0.72	14.26	2.57	1.46	5.62	5.58	7.18E-01	8.57E-01	2.43E-02	5.47E-01	7.43E-01	1.81E-01	1.82E-01	molybdopterin oxidoreductase, iron-sulfur binding subunit
KO:K00185	1.59	0.65	14.29	2.54	3.06	5.43	6.79	7.38E-01	8.76E-01	2.33E-02	5.82E-01	4.94E-01	2.06E-01	1.23E-01	molybdopterin oxidoreductase, membrane subunit [EC:1.2.7.-]
KO:K00200	0.15	0.00	0.27	0.44	0.07	0.00	0.27	3.37E-01	1.00E+00	2.44E-01	1.64E-01	4.58E-01	1.00E+00	2.44E-01	formylmethanofuran dehydrogenase subunit A [EC:1.2.99.5]
KO:K00201	0.00	0.00	0.00	0.03	0.00	0.00	2.14	4.24E-01	1.00E+00	1.00E+00	2.89E-01	1.00E+00	1.00E+00	1.96E-02	formylmethanofuran dehydrogenase subunit B [EC:1.2.99.5]
KO:K00202	0.00	0.00	2.42	0.46	0.00	0.00	0.03	1.00E+00	1.00E+00	3.27E-02	1.62E-01	1.00E+00	1.00E+00	3.96E-01	formylmethanofuran dehydrogenase subunit C [EC:1.2.99.5]
KO:K00206	0.18	6.49	0.00	0.00	0.00	0.00	0.00	4.32E-01	2.56E-02	1.00E+00	1.00E+00	1.00E+00	1.00E+00	1.00E+00	
KO:K00207	0.11	15.34	0.00	0.52	0.00	0.00	0.00	7.76E-01	4.00E-03	1.00E+00	6.37E-01	1.00E+00	1.00E+00	1.00E+00	dihydropyrimidine dehydrogenase (NADP+) [EC:1.3.1.2]
KO:K00208	3.54	17.82	6.61	8.14	4.47	2.86	2.26	9.01E-01	1.13E-02	4.96E-01	2.69E-01	8.30E-01	9.32E-01	9.54E-01	enoyl-[acyl-carrier protein] reductase I [EC:1.3.1.9]
KO:K00210	0.81	4.82	0.00	1.39	6.59	5.98	0.88	7.88E-01	1.27E-01	1.00E+00	6.41E-01	6.40E-02	8.01E-02	7.72E-01	prephenate dehydrogenase [EC:1.3.1.12]
KO:K00215	6.11	5.48	8.13	4.47	6.54	1.51	1.80	2.38E-01	3.40E-01	8.09E-02	5.76E-01	1.87E-01	9.57E-01	9.42E-01	dihydrodipicolinate reductase [EC:1.3.1.26]
KO:K00216	0.00	0.22	0.00	0.00	0.00	0.00	0.00	1.00E+00	6.87E-02	1.00E+00	1.00E+00	1.00E+00	1.00E+00	1.00E+00	2,3-dihydro-2,3-dihydroxybenzoate dehydrogenase [EC:1.3.1.28]
KO:K00217	1.52	0.29	0.00	0.25	0.00	0.00	0.00	9.89E-02	2.82E-01	1.00E+00	3.01E-01	1.00E+00	1.00E+00	1.00E+00	maleylacetate reductase [EC:1.3.1.32]
KO:K00218	0.00	0.00	0.00	0.01	24.47	0.00	0.00	1.00E+00	5.72E-01	1.00E+00	5.50E-01	0.00E+00	1.00E+00	1.00E+00	protochlorophyllide reductase [EC:1.3.1.33]
KO:K00219	4.08	33.95	0.07	0.22	0.00	0.00	0.17	2.94E-01	2.86E-04	9.48E-01	9.23E-01	1.00E+00	1.00E+00	9.33E-01	2,4-dienoyl-CoA reductase (NADPH2) [EC:1.3.1.34]
KO:K00220	0.00	6.40	0.00	0.52	0.00	0.00	0.00	1.00E+00	1.71E-02	1.00E+00	3.72E-01	1.00E+00	1.00E+00	1.00E+00	cyclohexadienyl dehydrogenase [EC:1.3.1.43]
KO:K00222	0.93	0.00	0.00	0.00	0.00	0.00	0.00	5.57E-02	1.00E+00	1.00E+00	1.00E+00	1.00E+00	1.00E+00	1.00E+00	delta14-sterol reductase [EC:1.3.1.7]
KO:K00224	0.70	0.00	0.00	0.00	0.00	0.00	0.00	6.01E-02	1.00E+00	1.00E+00	1.00E+00	1.00E+00	1.00E+00	1.00E+00	
KO:K00226	8.05	5.97	3.41	2.04	6.46	3.21	3.01	8.03E-02	2.17E-01	7.35E-01	9.11E-01	1.65E-01	7.76E-01	8.08E-01	dihydroorotate oxidase [EC:1.3.3.1]
KO:K00227	0.00	0.00	0.00	0.00	1.25	0.00	0.00	1.00E+00	1.00E+00	1.00E+00	1.00E+00	1.43E-02	1.00E+00	1.00E+00	lathosterol oxidase [EC:1.14.21.6]
KO:K00228	7.27	4.85	4.43	4.22	3.89	2.31	2.78	1.13E-01	4.18E-01	5.08E-01	5.55E-01	6.28E-01	8.86E-01	8.35E-01	coproporphyrinogen III oxidase [EC:1.3.3.3]
KO:K00231	0.00	0.00	12.62	0.17	0.07	0.00	0.28	1.00E+00	1.00E+00	5.71E-03	4.00E-01	4.88E-01	1.00E+00	3.24E-01	protoporphyrinogen oxidase [EC:1.3.3.4]
KO:K00232	0.00	0.15	0.00	0.03	0.00	0.00	0.00	1.00E+00	1.12E-01	1.00E+00	2.23E-01	1.00E+00	1.00E+00	1.00E+00	acyl-CoA oxidase [EC:1.3.3.6]
KO:K00234	0.00	0.00	0.00	1.84	0.00	0.00	0.00	1.00E+00	1.00E+00	1.00E+00	1.43E-01	1.00E+00	1.00E+00	1.00E+00	succinate dehydrogenase (ubiquinone) flavoprotein subunit
KO:K00235	2.56	0.00	0.00	0.00	0.00	0.00	0.00	3.46E-02	1.00E+00	1.00E+00	1.00E+00	1.00E+00	1.00E+00	1.00E+00	succinate dehydrogenase (ubiquinone) iron-sulfur protein
KO:K00239	12.59	10.23	17.93	9.89	1.30	7.41	7.47	1.23E-01	3.60E-01	7.57E-03	4.21E-01	9.98E-01	8.35E-01	8.30E-01	succinate dehydrogenase flavoprotein subunit [EC:1.3.99.1]
KO:K00240	7.80	7.31	16.05	5.31	1.06	3.74	2.57	1.93E-01	2.42E-01	7.57E-03	6.11E-01	9.82E-01	8.61E-01	9.40E-01	succinate dehydrogenase iron-sulfur protein [EC:1.3.99.1]
KO:K00241	9.70	5.01	9.38	4.25	1.54	9.68	4.31	1.08E-01	6.35E-01	1.21E-01	7.54E-01	9.59E-01	1.08E-01	7.47E-01	succinate dehydrogenase cytochrome b-556 subunit [EC:1.3.99.1]
KO:K00242	12.64	5.61	0.63	3.71	0.00	0.82	2.17	3.10E-02	2.16E-01	9.17E-01	4.61E-01	1.00E+00	9.00E-01	7.48E-01	succinate dehydrogenase hydrophobic membrane anchor protein
KO:K00243	1.25	0.35	1.21	0.18	2.16	0.00	0.17	1.65E-01	5.93E-01	1.70E-01	7.42E-01	7.03E-02	1.00E+00	7.51E-01	hypothetical protein
KO:K00244	0.03	9.65	0.02	1.68	0.00	0.00	0.00	7.46E-01	2.94E-02	7.57E-01	2.64E-01	1.00E+00	1.00E+00	1.00E+00	fumarate reductase flavoprotein subunit [EC:1.3.99.1]
KO:K00245	0.00	5.08	0.00	0.37	0.00	0.00	0.00	1.00E+00	5.01E-02	1.00E+00	3.14E-01	1.00E+00	1.00E+00	1.00E+00	fumarate reductase iron-sulfur protein [EC:1.3.99.1]
KO:K00246	0.00	7.03	0.00	0.01	0.00	0.00	0.00	5.45E-01	1.97E-02	1.00E+00	5.27E-01	1.00E+00	1.00E+00	1.00E+00	fumarate reductase subunit C [EC:1.3.99.1]
KO:K00247	0.00	0.78	0.00	0.00	0.00	0.00	0.00	1.00E+00	9.69E-02	1.00E+00	1.00E+00	1.00E+00	1.00E+00	1.00E+00	fumarate reductase subunit D [EC:1.3.99.1]
KO:K00248	9.97	2.13	0.00	3.22	0.00	0.67	0.08	2.79E-02	4.01E-01	1.00E+00	2.36E-01	1.00E+00	7.77E-01	9.12E-01	butyryl-CoA dehydrogenase [EC:1.3.99.2]
KO:K00249	14.53	20.57	0.80	9.71	0.00	0.09	0.00	5.77E-02	2.09E-02	9.47E-01	1.65E-01	1.00E+00	9.79E-01	9.87E-01	acyl-CoA dehydrogenase [EC:1.3.99.3]
KO:K00252	9.60	18.86	2.06	7.10	0.00	2.99	0.00	1.10E-01	9.57E-03	9.11E-01	2.63E-01	1.00E+00	8.42E-01	1.00E+00	glutaryl-CoA dehydrogenase [EC:1.3.99.7]
KO:K00253	4.47	22.69	1.79	3.07	0.00	0.00	0.19	3.36E-01	2.57E-03	8.07E-01	5.80E-01	1.00E+00	1.00E+00	9.56E-01	isovaleryl-CoA dehydrogenase [EC:1.3.99.1]
KO:K00254	0.00	0.00	0.00	1.02	0.00	0.00	0.00	1.00E+00	1.00E+00	1.00E+00	6.30E-02	1.00E+00	1.00E+00	1.00E+00	dihydroorotate dehydrogenase [EC:1.3.5.2]
KO:K00255	0.79	8.76	4.31	0.00	0.00	0.00	0.00	4.78E-01	4.59E-02	1.02E-01	7.28E-01	1.00E+00	1.00E+00	1.00E+00	long-chain-acyl-CoA dehydrogenase [EC:1.3.99.13]
KO:K00256	0.89	0.00	0.86	2.10	1.42	0.00	0.00	2.87E-01	1.00E+00	2.91E-01	1.54E-01	2.14E-01	1.00E+00	1.00E+00	isoquinoline 1-oxidoreductase [EC:1.3.99.16]
KO:K00257	21.24	24.57	0.75	10.49	0.00	3.77	0.67	1.51E-02	8.29E-03	9.90E-01	2.51E-01	1.00E+00	9.12E-01	9.91E-01	
KO:K00258	0.17	0.47	0.01	0.24	0.00	12.43	0.00	5.23E-01	3.61E-01	6.74E-01	4.77E-01	1.00E+00	5.29E-03	1.00E+00	
KO:K00259	9.80	16.33	0.49	10.46	3.49	2.91	0.87	1.40E-01	1.11E-02	9.94E-01	1.07E-01	9.16E-01	9.42E-01	9.89E-01	alanine dehydrogenase [EC:1.4.1.1]
KO:K00260	2.76	0.18	0.03	1.66	0.00	7.63	0.75	1.11E-01	8.63E-01	9.12E-01	2.66E-01	1.00E+00	1.07E-02	6.17E-01	glutamate dehydrogenase [EC:1.4.1.2]
KO:K00261	1.40	19.67	3.87	1.68	1.08	0.00	0.43	8.03E-01	5.71E-03	3.53E-01	7.64E-01	8.44E-01	1.00E+00	9.12E-01	glutamate dehydrogenase (NAD(P)+) [EC:1.4.1.3]
KO:K00262	4.03	1.23	2.17	2.25	0.00	5.03	0.31	1.84E-01	6.02E-01	3.85E-01	3.72E-01	1.00E+00	1.31E-01	8.12E-01	glutamate dehydrogenase (NADP+) [EC:1.4.1.4]
KO:K00263	5.32	1.09	0.24	2.15	4.92	0.00	0.00	6.54E-02	6.19E-01	8.70E-01	3.09E-01	7.73E-02	1.00E+00	9.50E-01	leucine dehydrogenase [EC:1.4.1.9]
KO:K00264	0.00	0.00	0.00	1.32	0.00	0.00	0.96	1.00E+00	1.00E+00	1.00E+00	9.80E-02	1.00E+00	1.00E+00	1.16E-01	glutamate synthase (NADPH/NADH) [EC:1.4.1.13 1.4.1.14]
KO:K00265	10.09	12.66	17.17	10.60	1.89	2.35	5.67	3.11E-01	1.22E-01	2.29E-02	2.55E-01	9.86E-01	9.80E-01	8.72E-01	glutamate synthase (NADPH/NADH) large chain [EC:1.4.1.13 1.4.1.14]
KO:K00266	7.54	17.75	14.70	7.70	0.89	1.36	5.23	5.34E-01	8.43E-03	2.80E-02	5.05E-01	9.95E-01	9.91E-01	8.64E-01	glutamate synthase (NADPH/NADH) small chain [EC:1.4.1.13 1.4.1.14]
KO:K00273	0.63	0.00	0.00	1.24	0.08	0.00	0.00	1.86E-01	1.00E+00	1.00E+00	1.07E-01	3.70E-01	1.00E+00	1.00E+00	D-amino-acid oxidase [EC:1.4.3.3]
KO:K00274	2.56	0.19	0.00	0.40	0.72	0.17	0.00	7.91E-02	5.37E-01	1.00E+00	4.06E-01	2.80E-01	5.56E-01	1.00E+00	monoamine oxidase [EC:1.4.3.4]
KO:K00275	4.65	5.85	2.02	5.11	0.79	0.10	1.22	1.77E-01	9.80E-02	7.78E-01	1.35E-01	9.43E-01	9.92E-01	9.03E-01	pyridoxamine 5'-phosphate oxidase [EC:1.4.3.5]
KO:K00276	1.28	0.00	0.00	0.00	0.00	0.00	0.00	6.46E-02	1.00E+00	1.00E+00	1.00E+00	1.00E+00	1.00E+00	1.00E+00	primary-amine oxidase [EC:1.4.3.21]
KO:K00278	3.34	0.03	11.53	1.68	6.03	1.57	2.35	2.46E-01	9.70E-01	1.31E-02	6.26E-01	7.03E-02	6.56E-01	4.35E-01	L-aspartate oxidase [EC:1.4.3.16]
KO:K00281	4.12	10.47	5.16	1.62	4.42	0.00	0.00	3.12E-01	4.13E-02	2.04E-01	7.74E-01	2.75E-01	1.00E+00	1.00E+00	glycine dehydrogenase [EC:1.4.4.2]
KO:K00282	0.58	0.00	5.09	2.79	0.07	4.34	0.03	5.43E-01	1.00E+00	5.47E-02	1.40E-01	7.74E-01	7.01E-02	7.96E-01	glycine dehydrogenase subunit 1 [EC:1.4.4.2]

KO:K00283	5.11	4.35	1.71	5.99	3.10	4.70	3.05	3.38E-01	4.64E-01	8.84E-01	2.32E-01	7.03E-01	4.04E-01	7.13E-01	glycine dehydrogenase subunit 2 [EC:1.4.4.2]
KO:K00284	0.00	2.65	0.17	0.66	30.02	0.00	0.36	8.14E-01	2.23E-01	7.94E-01	6.53E-01	7.14E-04	1.00E+00	7.44E-01	glutamate synthase (ferredoxin) [EC:1.4.7.1]
KO:K00285	2.19	16.14	0.93	5.62	0.00	0.03	0.23	6.51E-01	1.01E-02	8.66E-01	1.54E-01	1.00E+00	9.79E-01	9.44E-01	D-amino-acid dehydrogenase [EC:1.4.99.1]
KO:K00286	3.74	7.70	1.78	3.16	5.86	1.40	1.40	4.74E-01	6.47E-02	8.79E-01	6.14E-01	1.48E-01	9.13E-01	9.13E-01	pyrroline-5-carboxylate reductase [EC:1.5.1.2]
KO:K00287	4.57	0.21	2.09	1.35	1.13	0.66	1.24	7.09E-02	8.94E-01	2.71E-01	4.77E-01	5.59E-01	7.51E-01	5.16E-01	dihydrofolate reductase [EC:1.5.1.3]
KO:K00288	0.00	0.00	1.62	0.33	0.00	0.00	0.00	4.74E-01	4.76E-01	2.64E-02	1.69E-01	1.00E+00	1.00E+00	1.00E+00	methylenetetrahydrofolate dehydrogenase (NADP+) /
KO:K00290	0.81	4.65	1.94	1.42	0.00	0.00	1.11	6.33E-01	6.11E-02	2.74E-01	4.05E-01	1.00E+00	1.00E+00	5.10E-01	saccharopine dehydrogenase (NAD+, L-lysine forming) [EC:1.5.1.7]
KO:K00292	1.39	0.54	0.00	0.91	0.00	5.16	0.00	2.19E-01	4.73E-01	1.00E+00	3.28E-01	1.00E+00	3.24E-02	1.00E+00	saccharopine dehydrogenase (NAD+, L-glutamate forming) [EC:1.5.1.9]
KO:K00293	0.00	15.61	0.00	0.00	0.00	0.00	0.15	7.33E-01	5.00E-03	1.00E+00	1.00E+00	1.00E+00	1.00E+00	6.61E-01	saccharopine dehydrogenase (NADP+, L-glutamate forming)
KO:K00294	2.36	1.81	0.00	2.32	0.00	13.70	0.00	2.91E-01	3.91E-01	1.00E+00	2.96E-01	1.00E+00	1.07E-02	1.00E+00	1-pyrroline-5-carboxylate dehydrogenase [EC:1.5.1.12]
KO:K00297	3.80	11.14	11.75	3.70	6.91	0.01	0.89	6.82E-01	3.49E-02	2.84E-02	7.01E-01	1.80E-01	9.99E-01	9.72E-01	methylenetetrahydrofolate reductase (NADPH) [EC:1.5.1.2]
KO:K00299	0.00	0.00	0.54	0.00	8.62	0.00	0.34	1.00E+00	1.00E+00	1.81E-01	1.00E+00	1.64E-02	1.00E+00	2.01E-01	FMN reductase [EC:1.5.1.29]
KO:K00300	0.00	0.00	5.94	0.05	0.00	0.00	0.17	1.00E+00	1.00E+00	3.71E-03	4.03E-01	1.00E+00	1.00E+00	2.85E-01	
KO:K00301	0.10	0.13	0.02	0.15	5.25	0.00	0.74	5.75E-01	5.51E-01	6.84E-01	5.29E-01	1.27E-02	1.00E+00	2.22E-01	sarcosine oxidase [EC:1.5.3.1]
KO:K00302	4.43	43.55	0.00	2.85	0.00	0.00	0.00	4.53E-01	1.43E-04	1.00E+00	6.95E-01	1.00E+00	1.00E+00	1.00E+00	sarcosine oxidase, subunit alpha [EC:1.5.3.1]
KO:K00303	3.40	47.32	0.00	2.57	0.00	0.00	0.00	6.06E-01	0.00E+00	1.00E+00	7.33E-01	1.00E+00	1.00E+00	1.00E+00	sarcosine oxidase, subunit beta [EC:1.5.3.1]
KO:K00304	2.33	11.93	0.00	1.53	0.00	0.00	0.00	2.79E-01	1.49E-02	1.00E+00	4.48E-01	1.00E+00	1.00E+00	1.00E+00	sarcosine oxidase, subunit delta [EC:1.5.3.1]
KO:K00305	2.23	18.46	0.00	0.94	0.00	0.00	0.00	3.60E-01	4.14E-03	1.00E+00	6.93E-01	1.00E+00	1.00E+00	1.00E+00	sarcosine oxidase, subunit gamma [EC:1.5.3.1]
KO:K00308	0.00	0.00	0.00	0.20	0.00	0.00	0.00	1.00E+00	1.00E+00	1.00E+00	1.43E-01	1.00E+00	1.00E+00	1.00E+00	N1-acetylpolyamine oxidase [EC:1.5.3.13]
KO:K00309	0.00	2.78	0.00	0.00	0.00	0.00	0.00	4.53E-01	4.37E-02	1.00E+00	1.00E+00	1.00E+00	1.00E+00	1.00E+00	
KO:K00311	14.32	18.20	1.45	4.13	0.00	0.00	0.44	2.79E-02	9.43E-03	9.25E-01	6.10E-01	1.00E+00	1.00E+00	9.72E-01	electron-transferring-flavoprotein dehydrogenase [EC:1.5.5.1]
KO:K00313	0.00	0.00	0.00	1.94	0.00	0.00	0.00	1.00E+00	1.00E+00	1.00E+00	1.43E-01	1.00E+00	1.00E+00	1.00E+00	electron transfer flavoprotein-quinone oxidoreductase [EC:1.5.5.-]
KO:K00314	1.83	18.34	0.00	3.69	0.00	0.00	0.00	5.42E-01	1.00E-02	1.00E+00	2.62E-01	1.00E+00	1.00E+00	1.00E+00	sarcosine dehydrogenase [EC:1.5.99.1]
KO:K00315	1.69	50.26	0.38	1.37	0.00	0.00	0.00	7.68E-01	0.00E+00	9.02E-01	8.07E-01	1.00E+00	1.00E+00	1.00E+00	dimethylglycine dehydrogenase [EC:1.5.99.2]
KO:K00316	10.58	0.00	0.00	0.00	0.00	0.00	0.00	1.79E-02	1.00E+00	1.00E+00	1.00E+00	1.00E+00	1.00E+00	1.00E+00	spermidine dehydrogenase [EC:1.5.99.6]
KO:K00317	0.00	0.56	0.00	0.00	0.00	0.00	0.00	1.00E+00	1.02E-01	1.00E+00	1.00E+00	1.00E+00	1.00E+00	1.00E+00	trimethylamine dehydrogenase [EC:1.5.8.2]
KO:K00318	3.42	18.89	0.84	2.85	0.01	0.66	0.18	4.44E-01	7.14E-04	9.38E-01	5.88E-01	9.87E-01	9.49E-01	9.78E-01	proline dehydrogenase [EC:1.5.99.8]
KO:K00320	8.32	0.00	0.00	0.00	0.00	0.00	0.02	2.71E-02	1.00E+00	1.00E+00	1.00E+00	1.00E+00	1.00E+00	4.78E-01	coenzyme F42-dependent N5,N1-methenyltetrahydromethanopterin
KO:K00322	0.92	1.48	1.61	0.78	0.24	7.95	1.32	6.20E-01	4.18E-01	3.82E-01	6.74E-01	8.55E-01	1.80E-02	4.73E-01	NAD(P) transhydrogenase [EC:1.6.1.1]
KO:K00324	8.63	13.09	9.94	7.63	8.16	0.28	0.49	2.81E-01	6.34E-02	1.70E-01	4.04E-01	3.33E-01	9.93E-01	9.90E-01	NAD(P) transhydrogenase subunit alpha [EC:1.6.1.2]
KO:K00325	7.82	13.16	9.65	8.49	8.33	1.60	0.17	3.72E-01	5.23E-02	1.80E-01	2.85E-01	3.07E-01	9.74E-01	9.94E-01	NAD(P) transhydrogenase subunit beta [EC:1.6.1.2]
KO:K00329	0.87	12.02	0.00	0.19	0.00	0.00	0.00	4.47E-01	1.14E-02	1.00E+00	7.51E-01	1.00E+00	1.00E+00	1.00E+00	NADH dehydrogenase [EC:1.6.5.3]
KO:K00330	1.68	4.81	9.36	4.35	1.25	2.86	7.77	8.72E-01	3.70E-01	6.77E-02	4.43E-01	9.07E-01	7.18E-01	1.12E-01	NADH dehydrogenase I subunit A [EC:1.6.5.3]
KO:K00331	3.01	4.37	10.29	3.83	0.79	0.06	14.91	6.87E-01	4.38E-01	5.64E-02	5.40E-01	9.36E-01	9.78E-01	1.74E-02	NADH dehydrogenase I subunit B [EC:1.6.5.3]
KO:K00332	0.78	6.46	11.26	3.76	1.11	0.81	3.61	9.22E-01	1.20E-01	2.70E-02	4.03E-01	8.92E-01	9.19E-01	4.34E-01	NADH dehydrogenase I subunit C [EC:1.6.5.3]
KO:K00333	0.88	8.12	11.78	4.92	0.96	2.06	7.39	9.52E-01	1.15E-01	3.59E-02	4.03E-01	9.48E-01	8.71E-01	1.50E-01	NADH dehydrogenase I subunit D [EC:1.6.5.3]
KO:K00334	1.63	11.81	5.87	1.84	1.08	0.02	1.24	7.77E-01	2.17E-02	1.19E-01	7.37E-01	8.58E-01	9.69E-01	8.36E-01	NADH dehydrogenase I subunit E [EC:1.6.5.3]
KO:K00335	3.25	14.92	6.54	6.99	0.60	1.69	1.98	7.72E-01	2.73E-02	2.80E-01	2.36E-01	9.71E-01	9.16E-01	8.95E-01	NADH dehydrogenase I subunit F [EC:1.6.5.3]
KO:K00336	3.10	11.68	10.31	6.49	0.53	1.06	3.39	8.23E-01	4.44E-02	7.01E-02	2.81E-01	9.77E-01	9.60E-01	7.85E-01	NADH dehydrogenase I subunit G [EC:1.6.5.3]
KO:K00337	1.55	7.09	11.77	4.95	0.89	2.99	6.65	9.15E-01	1.75E-01	3.59E-02	4.22E-01	9.52E-01	7.71E-01	2.09E-01	NADH dehydrogenase I subunit H [EC:1.6.5.3]
KO:K00338	1.45	3.61	10.62	1.88	0.76	0.00	5.64	7.56E-01	2.71E-01	1.94E-02	6.53E-01	8.83E-01	1.00E+00	1.02E-01	NADH dehydrogenase I subunit I [EC:1.6.5.3]
KO:K00339	1.33	7.44	2.62	5.04	0.77	0.02	4.98	8.59E-01	6.59E-02	6.15E-01	1.81E-01	9.16E-01	9.85E-01	1.85E-01	NADH dehydrogenase I subunit J [EC:1.6.5.3]
KO:K00340	1.86	2.05	8.73	2.34	1.02	0.00	0.97	5.01E-01	4.51E-01	2.83E-02	3.76E-01	7.40E-01	1.00E+00	7.53E-01	NADH dehydrogenase I subunit K [EC:1.6.5.3]
KO:K00341	2.01	9.87	14.61	6.82	0.82	4.61	5.48	9.33E-01	9.94E-02	2.21E-02	3.26E-01	9.78E-01	6.81E-01	5.32E-01	NADH dehydrogenase I subunit L [EC:1.6.5.3]
KO:K00342	2.35	8.87	11.44	7.20	1.25	0.30	5.85	8.83E-01	1.20E-01	5.37E-02	2.17E-01	9.47E-01	9.84E-01	3.73E-01	NADH dehydrogenase I subunit M [EC:1.6.5.3]
KO:K00343	2.48	10.09	9.56	7.76	0.40	1.54	4.72	8.92E-01	6.50E-02	7.81E-02	1.55E-01	9.84E-01	9.47E-01	5.62E-01	NADH dehydrogenase I subunit N [EC:1.6.5.3]
KO:K00344	10.27	16.11	5.91	10.87	0.30	1.68	0.74	1.50E-01	3.26E-02	5.76E-01	1.23E-01	9.89E-01	9.50E-01	9.78E-01	NADPH2:quinone reductase [EC:1.6.5.5]
KO:K00346	15.58	2.85	7.86	3.25	0.77	4.42	1.04	2.70E-02	7.58E-01	1.81E-01	7.04E-01	9.25E-01	5.23E-01	9.10E-01	Na+-transporting NADH:ubiquinone oxidoreductase subunit A
KO:K00347	14.60	4.75	6.49	3.78	0.63	4.45	0.67	2.97E-02	4.97E-01	2.78E-01	6.66E-01	9.52E-01	5.48E-01	9.51E-01	Na+-transporting NADH:ubiquinone oxidoreductase subunit B
KO:K00348	11.26	1.76	3.17	3.17	0.52	2.20	0.61	2.97E-02	7.56E-01	4.73E-01	4.73E-01	9.16E-01	6.69E-01	9.10E-01	Na+-transporting NADH:ubiquinone oxidoreductase subunit C
KO:K00349	11.13	3.94	3.99	2.97	0.21	3.66	0.62	3.84E-02	4.37E-01	4.28E-01	6.25E-01	9.63E-01	4.90E-01	9.36E-01	Na+-transporting NADH:ubiquinone oxidoreductase subunit D
KO:K00350	12.47	2.40	5.18	2.48	0.35	5.64	0.53	2.87E-02	7.10E-01	2.71E-01	6.96E-01	9.36E-01	2.18E-01	9.25E-01	Na+-transporting NADH:ubiquinone oxidoreductase subunit E
KO:K00351	11.99	3.48	7.32	3.64	0.47	3.83	1.08	3.89E-02	6.42E-01	1.64E-01	6.13E-01	9.55E-01	5.76E-01	9.27E-01	Na+-transporting NADH:ubiquinone oxidoreductase subunit F
KO:K00353	0.31	0.94	0.00	0.57	0.00	0.00	0.00	2.78E-01	1.71E-01	1.00E+00	2.24E-01	1.00E+00	1.00E+00	1.00E+00	
KO:K00354	0.02	0.17	0.00	6.16	0.00	0.00	0.00	5.49E-01	4.39E-01	1.00E+00	4.13E-02	1.00E+00	1.00E+00	1.00E+00	NADPH2 dehydrogenase [EC:1.6.99.1]
KO:K00355	0.00	0.18	0.00	4.78	0.00	0.00	0.00	1.00E+00	3.70E-01	1.00E+00	5.03E-02	1.00E+00	1.00E+00	1.00E+00	NAD(P)H dehydrogenase (quinone) [EC:1.6.5.2]
KO:K00356	1.44	28.77	0.10	7.40	0.00	0.17	0.31	8.62E-01	1.29E-03	9.70E-01	1.37E-01	9.85E-01	9.61E-01	9.53E-01	NADH dehydrogenase [EC:1.6.99.3]
KO:K00358	0.27	0.00	0.00	0.02	0.00	0.00	0.00	6.59E-02	1.00E+00	1.00E+00	2.80E-01	1.00E+00	1.00E+00	1.00E+00	
KO:K00359	0.15	0.61	0.03	0.54	0.00	0.99	0.06	4.53E-01	2.44E-01	6.08E-01	2.63E-01	1.00E+00	1.68E-01	5.42E-01	NADH oxidase [EC:1.6.-.-]
KO:K00360	0.00	0.00	0.00	0.00	0.00	4.89	0.00	1.00E+00	2.53E-01	1.00E+00	1.00E+00	1.00E+00	1.43E-03	1.00E+00	nitrate reductase (NADH) [EC:1.7.1.1]
KO:K00362	0.02	0.02	0.62	0.07	0.00	0.00	0.07	5.38E-01	5.51E-01	3.51E-02	3.72E-01	1.00E+00	1.00E+00	3.61E-01	nitrite reductase (NAD(P)H) large subunit [EC:1.7.1.4]
KO:K00363	0.00	0.00	4.86	0.02	0.04	0.00	0.41	1.00E+00	1.00E+00	8.14E-03	4.47E-01	3.93E-01	1.00E+00	1.62E-01	nitrite reductase (NAD(P)H) small subunit [EC:1.7.1.4]
KO:K00364	1.31	0.18	4.22	0.11	0.00	0.00	0.00	1.92E-01	4.06E-01	6.39E-02	4.51E-01	1.00E+00	1.00E+00	1.00E+00	GMP reductase [EC:1.7.1.7]

KO:K00365	0.00	0.00	0.00	2.35	0.00	0.00	0.00	1.00E+00	1.00E+00	1.00E+00	4.50E-02	1.00E+00	1.00E+00	1.00E+00	urate oxidase [EC:1.7.3.3]
KO:K00366	0.00	0.00	0.02	0.01	15.41	0.00	0.37	6.45E-01	1.00E+00	6.02E-01	6.22E-01	0.00E+00	1.00E+00	2.71E-01	ferredoxin-nitrite reductase [EC:1.7.7.1]
KO:K00367	0.00	0.00	0.00	0.00	10.63	0.00	0.00	1.00E+00	1.00E+00	1.00E+00	1.00E+00	1.43E-03	1.00E+00	1.00E+00	ferredoxin-nitrate reductase [EC:1.7.7.2]
KO:K00368	0.20	0.51	0.05	0.01	0.00	0.00	1.72	3.56E-01	1.86E-01	5.37E-01	6.41E-01	1.00E+00	1.00E+00	6.39E-02	nitrite reductase (NO-forming) [EC:1.7.2.1]
KO:K00369	0.80	0.01	0.00	0.00	0.00	0.00	0.28	8.26E-02	4.47E-01	1.00E+00	4.60E-01	1.00E+00	1.00E+00	1.72E-01	nitrate reductase [EC:1.7.99.4]
KO:K00370	0.00	0.11	0.00	0.00	0.00	0.00	0.00	1.00E+00	1.03E-01	1.00E+00	1.00E+00	1.00E+00	1.00E+00	1.00E+00	nitrate reductase 1, alpha subunit [EC:1.7.99.4]
KO:K00371	0.00	0.11	0.00	0.00	0.00	0.00	0.00	1.00E+00	9.89E-02	1.00E+00	1.00E+00	1.00E+00	1.00E+00	1.00E+00	nitrate reductase 1, beta subunit [EC:1.7.99.4]
KO:K00372	0.00	0.00	0.21	0.02	0.21	0.00	0.09	1.00E+00	1.00E+00	8.86E-02	3.21E-01	8.86E-02	1.00E+00	1.82E-01	nitrate reductase catalytic subunit [EC:1.7.99.4]
KO:K00373	0.12	0.00	0.00	0.00	0.00	0.00	0.00	5.83E-02	1.00E+00	1.00E+00	1.00E+00	1.00E+00	1.00E+00	1.00E+00	nitrate reductase 1, delta subunit [EC:1.7.99.4]
KO:K00374	0.00	0.11	0.00	0.00	0.00	0.00	0.00	1.00E+00	1.04E-01	1.00E+00	1.00E+00	1.00E+00	1.00E+00	1.00E+00	nitrate reductase 1, gamma subunit [EC:1.7.99.4]
KO:K00375	0.48	13.20	0.00	0.02	0.00	0.00	0.00	5.50E-01	8.29E-03	1.00E+00	8.49E-01	1.00E+00	1.00E+00	1.00E+00	GntR family transcriptional regulator / MocR family aminotransferase
KO:K00376	0.00	0.11	0.00	0.00	0.00	0.00	0.00	1.00E+00	9.81E-02	1.00E+00	1.00E+00	1.00E+00	1.00E+00	1.00E+00	nitrous-oxide reductase [EC:1.7.99.6]
KO:K00380	0.63	0.01	16.80	0.00	0.40	0.00	0.37	3.36E-01	7.43E-01	6.29E-03	7.55E-01	4.24E-01	1.00E+00	4.42E-01	sulfite reductase (NADPH) flavoprotein alpha-component [EC:1.8.1.2]
KO:K00381	4.61	6.67	10.90	1.68	2.09	0.08	0.93	2.21E-01	7.84E-02	1.70E-02	8.51E-01	7.81E-01	9.90E-01	9.35E-01	sulfite reductase (NADPH) hemoprotein beta-component [EC:1.8.1.2]
KO:K00382	12.05	19.20	10.93	10.48	5.68	7.60	3.95	2.66E-01	4.71E-03	4.54E-01	5.47E-01	9.75E-01	9.19E-01	9.92E-01	dihydrolipoamide dehydrogenase [EC:1.8.1.4]
KO:K00383	5.41	16.79	0.06	2.72	3.39	0.63	2.54	3.04E-01	7.71E-03	9.87E-01	7.86E-01	6.80E-01	9.59E-01	8.12E-01	glutathione reductase (NADPH) [EC:1.8.1.7]
KO:K00384	9.91	6.87	9.78	10.73	5.46	5.50	2.91	1.80E-01	6.09E-01	1.92E-01	1.25E-01	8.16E-01	8.10E-01	9.63E-01	thioredoxin reductase (NADPH) [EC:1.8.1.9]
KO:K00386	0.63	15.51	0.00	0.15	0.00	0.00	0.02	5.48E-01	1.71E-02	1.00E+00	6.70E-01	1.00E+00	1.00E+00	7.07E-01	
KO:K00387	1.35	0.00	0.00	2.69	0.00	0.00	0.00	1.80E-01	1.00E+00	1.00E+00	1.11E-01	1.00E+00	1.00E+00	1.00E+00	sulfite oxidase [EC:1.8.3.1]
KO:K00389	0.00	0.01	0.47	0.01	0.00	0.00	0.00	1.00E+00	3.06E-01	1.99E-02	3.18E-01	1.00E+00	1.00E+00	1.00E+00	putative membrane protein
KO:K00390	1.44	0.66	7.76	0.56	9.11	2.90	0.25	5.71E-01	8.55E-01	1.14E-02	8.85E-01	7.43E-03	1.53E-01	9.49E-01	phosphoadenosine phosphosulfate reductase [EC:1.8.4.8]
KO:K00391	0.12	0.00	0.00	0.00	0.00	0.00	0.00	6.60E-02	1.00E+00	1.00E+00	1.00E+00	1.00E+00	1.00E+00	1.00E+00	
KO:K00392	0.07	0.00	3.05	0.34	16.35	0.00	0.02	6.87E-01	7.47E-01	7.79E-02	4.98E-01	2.29E-03	1.00E+00	7.32E-01	sulfite reductase (ferredoxin) [EC:1.8.7.1]
KO:K00394	0.66	0.00	0.00	2.60	0.00	0.00	8.93	3.96E-01	1.00E+00	1.00E+00	1.89E-01	1.00E+00	1.00E+00	3.61E-02	adenylylsulfate reductase, subunit A [EC:1.8.99.2]
KO:K00395	0.61	0.00	0.00	3.04	0.00	0.00	8.73	4.28E-01	1.00E+00	1.00E+00	1.52E-01	1.00E+00	1.00E+00	4.04E-02	adenylylsulfate reductase, subunit B [EC:1.8.99.2]
KO:K00397	0.00	0.00	0.00	0.92	0.00	0.00	0.00	1.00E+00	1.00E+00	1.00E+00	6.30E-02	1.00E+00	1.00E+00	1.00E+00	anaerobic dimethyl sulfoxide reductase [EC:1.8.99.-]
KO:K00404	4.09	0.33	8.61	0.63	0.73	0.00	10.55	1.71E-01	8.55E-01	4.13E-02	7.99E-01	7.81E-01	1.00E+00	2.24E-02	cb-type cytochrome c oxidase subunit I [EC:1.9.3.1]
KO:K00405	1.09	0.08	13.36	0.22	1.19	0.00	6.51	4.72E-01	8.49E-01	9.00E-03	7.94E-01	4.36E-01	1.00E+00	4.23E-02	cb-type cytochrome c oxidase subunit II [EC:1.9.3.1]
KO:K00406	3.22	0.52	0.24	0.68	0.00	0.00	6.41	9.63E-02	6.38E-01	7.82E-01	5.56E-01	1.00E+00	1.00E+00	3.06E-02	cb-type cytochrome c oxidase subunit III [EC:1.9.3.1]
KO:K00407	0.27	0.01	0.00	0.31	0.00	0.68	0.00	2.11E-01	6.39E-01	1.00E+00	1.93E-01	1.00E+00	7.10E-02	1.00E+00	cb-type cytochrome c oxidase subunit IV [EC:1.9.3.1]
KO:K00411	10.44	8.23	0.82	9.95	0.00	4.78	3.94	9.47E-02	1.93E-01	9.66E-01	1.10E-01	1.00E+00	6.21E-01	7.49E-01	ubiquinol-cytochrome c reductase iron-sulfur subunit [EC:1.1.2.2]
KO:K00412	13.34	14.49	1.50	9.28	0.01	8.84	4.13	7.09E-02	5.13E-02	9.78E-01	2.77E-01	9.96E-01	3.26E-01	8.82E-01	ubiquinol-cytochrome c reductase cytochrome b subunit [EC:1.1.2.2]
KO:K00413	4.80	10.48	0.18	8.18	0.00	2.40	2.40	3.72E-01	5.31E-02	9.71E-01	1.04E-01	1.00E+00	7.93E-01	7.93E-01	ubiquinol-cytochrome c reductase cytochrome c1 subunit [EC:1.1.2.2]
KO:K00423	0.21	0.05	1.53	0.01	0.00	0.00	0.00	2.10E-01	4.14E-01	3.07E-02	5.14E-01	1.00E+00	1.00E+00	1.00E+00	L-ascorbate oxidase [EC:1.1.3.3]
KO:K00425	0.63	3.37	5.41	3.08	0.00	0.00	0.00	6.74E-01	1.26E-01	5.41E-02	1.46E-01	1.00E+00	1.00E+00	1.00E+00	cytochrome bd-I oxidase subunit I [EC:1.1.3.-]
KO:K00426	0.45	1.82	5.24	1.14	0.00	0.31	0.00	6.07E-01	1.91E-01	3.83E-02	3.24E-01	1.00E+00	6.88E-01	9.20E-01	cytochrome bd-I oxidase subunit II [EC:1.1.3.-]
KO:K00428	1.73	10.11	9.96	2.89	1.00	0.00	0.22	7.47E-01	2.77E-02	2.89E-02	4.90E-01	8.74E-01	1.00E+00	9.54E-01	cytochrome c peroxidase [EC:1.11.1.5]
KO:K00429	0.12	0.00	0.00	0.00	0.00	0.00	0.00	6.14E-02	1.00E+00	1.00E+00	1.00E+00	1.00E+00	1.00E+00	1.00E+00	catalase [EC:1.11.1.6]
KO:K00430	0.01	8.15	1.51	0.02	0.07	0.00	0.03	7.95E-01	2.31E-02	2.23E-01	7.63E-01	7.14E-01	1.00E+00	7.53E-01	peroxidase [EC:1.11.1.7]
KO:K00432	7.31	2.23	5.78	3.67	5.47	1.92	3.23	1.10E-01	8.30E-01	2.14E-01	5.68E-01	2.49E-01	8.69E-01	6.60E-01	glutathione peroxidase [EC:1.11.1.9]
KO:K00433	0.09	0.00	0.43	0.00	0.00	0.77	0.00	2.29E-01	1.00E+00	9.59E-02	4.09E-01	1.00E+00	4.99E-02	1.00E+00	chloride peroxidase [EC:1.11.1.1]
KO:K00435	2.63	0.88	1.32	0.33	0.00	4.18	0.04	1.63E-01	4.42E-01	3.33E-01	6.31E-01	1.00E+00	8.21E-02	7.74E-01	peroxiredoxin [EC:1.11.1.-]
KO:K00436	0.04	0.00	0.00	0.83	0.00	0.00	0.00	1.82E-01	1.00E+00	1.00E+00	1.22E-01	1.00E+00	1.00E+00	1.00E+00	hydrogen dehydrogenase [EC:1.12.1.2]
KO:K00437	0.00	0.00	0.00	0.20	0.00	0.00	0.00	1.00E+00	1.00E+00	1.00E+00	8.39E-02	1.00E+00	1.00E+00	1.00E+00	cytochrome-c3 hydrogenase [EC:1.12.2.1]
KO:K00441	0.05	0.07	0.34	0.20	7.68	0.00	0.00	5.64E-01	5.30E-01	3.18E-01	3.92E-01	1.19E-02	1.00E+00	1.00E+00	coenzyme F42 hydrogenase beta subunit [EC:1.12.98.1]
KO:K00446	0.33	3.49	0.00	1.40	0.00	0.00	0.00	5.34E-01	4.99E-02	1.00E+00	1.82E-01	1.00E+00	1.00E+00	1.00E+00	catechol 2,3-dioxygenase [EC:1.13.11.2]
KO:K00448	0.03	4.45	0.00	0.17	0.00	0.00	0.00	7.67E-01	1.10E-02	1.00E+00	5.51E-01	1.00E+00	1.00E+00	1.00E+00	protocatechuate 3,4-dioxygenase, alpha subunit [EC:1.13.11.3]
KO:K00449	0.00	0.75	3.70	0.27	1.64	0.00	0.07	8.36E-01	2.41E-01	2.93E-02	5.17E-01	9.46E-02	1.00E+00	7.46E-01	protocatechuate 3,4-dioxygenase, beta subunit [EC:1.13.11.3]
KO:K00450	0.00	3.99	0.00	0.44	0.00	0.00	0.00	1.00E+00	4.40E-02	1.00E+00	3.15E-01	1.00E+00	1.00E+00	1.00E+00	gentisate 1,2-dioxygenase [EC:1.13.11.4]
KO:K00451	2.18	7.11	0.05	1.35	0.00	0.08	0.00	2.17E-01	1.91E-02	9.18E-01	4.12E-01	1.00E+00	8.94E-01	1.00E+00	homogentisate 1,2-dioxygenase [EC:1.13.11.5]
KO:K00452	1.44	0.52	0.00	2.17	0.00	0.00	0.00	1.68E-01	3.73E-01	1.00E+00	1.03E-01	1.00E+00	1.00E+00	1.00E+00	3-hydroxyanthranilate 3,4-dioxygenase [EC:1.13.11.6]
KO:K00453	1.11	0.98	0.00	1.61	0.00	0.19	0.01	2.12E-01	2.40E-01	1.00E+00	1.34E-01	1.00E+00	6.52E-01	8.44E-01	tryptophan 2,3-dioxygenase [EC:1.13.11.11]
KO:K00455	1.13	0.06	0.00	0.00	0.00	0.00	0.00	5.96E-02	2.97E-01	1.00E+00	1.00E+00	1.00E+00	1.00E+00	1.00E+00	3,4-dihydroxyphenylacetate 2,3-dioxygenase [EC:1.13.11.15]
KO:K00456	0.00	0.00	0.00	0.20	0.00	0.00	0.00	1.00E+00	1.00E+00	1.00E+00	1.43E-01	1.00E+00	1.00E+00	1.00E+00	cysteine dioxygenase [EC:1.13.11.2]
KO:K00457	3.22	12.62	0.00	2.50	0.00	6.58	0.00	3.42E-01	1.14E-02	1.00E+00	5.11E-01	1.00E+00	7.56E-02	9.74E-01	4-hydroxyphenylpyruvate dioxygenase [EC:1.13.11.27]
KO:K00459	5.97	0.18	2.85	7.23	0.00	0.00	0.26	8.23E-02	8.65E-01	2.79E-01	5.97E-02	1.00E+00	1.00E+00	8.46E-01	nitronate monooxygenase [EC:1.13.12.16]
KO:K00462	2.76	0.60	0.00	0.51	0.00	0.00	0.00	1.14E-01	2.80E-01	1.00E+00	2.95E-01	1.00E+00	1.00E+00	1.00E+00	biphenyl-2,3-diol 1,2-dioxygenase [EC:1.13.11.39]
KO:K00464	0.15	0.00	0.00	0.00	24.93	0.00	0.00	4.10E-01	1.00E+00	1.00E+00	1.00E+00	2.86E-04	1.00E+00	1.00E+00	lignostilbene-alpha,beta-dioxygenase [EC:1.13.11.43]
KO:K00465	0.00	0.00	3.23	1.78	0.00	0.00	0.00	1.00E+00	1.00E+00	6.23E-02	1.10E-01	1.00E+00	1.00E+00	1.00E+00	
KO:K00466	0.00	0.00	0.00	0.10	0.00	0.00	0.00	1.00E+00	1.00E+00	1.00E+00	1.43E-01	1.00E+00	1.00E+00	1.00E+00	tryptophan 2-monooxygenase [EC:1.13.12.3]
KO:K00467	0.00	0.00	0.00	0.20	0.00	0.00	0.00	1.00E+00	1.00E+00	1.00E+00	1.43E-01	1.00E+00	1.00E+00	1.00E+00	lactate 2-monooxygenase [EC:1.13.12.4]
KO:K00469	0.53	0.16	0.00	0.76	1.06	0.00	0.00	2.65E-01	4.13E-01	1.00E+00	2.15E-01	1.76E-01	1.00E+00	1.00E+00	inositol oxygenase [EC:1.13.99.1]
KO:K00470	0.16	3.98	0.34	0.00	0.00	0.00	0.07	4.17E-01	5.96E-02	3.32E-01	1.00E+00	1.00E+00	1.00E+00	4.82E-01	
KO:K00471	0.70	4.30	0.00	1.41	0.00	0.00	0.00	4.01E-01	6.39E-02	1.00E+00	2.48E-01	1.00E+00	1.00E+00	1.00E+00	gamma-butyrobetaine dioxygenase [EC:1.14.11.1]
KO:K00472	1.22	0.01	0.00	0.01	1.01	0.00	0.00	1.33E-01	3.71E-01	1.00E+00	3.79E-01	1.51E-01	1.00E+00	1.00E+00	prolyl 4-hydroxylase [EC:1.14.11.2]
KO:K00475	1.86	0.00	0.00	0.00	0.00	0.00	0.00	6.29E-02	1.00E+00	1.00E+00	1.00E+00	1.00E+00	1.00E+00	1.00E+00	naringenin 3-dioxygenase [EC:1.14.11.9]
KO:K00476	0.00	0.00	0.00	0.61	0.00	0.00	0.00	1.00E+00	1.00E+00	1.00E+00	1.43E-01	1.00E+00	1.00E+00	1.00E+00	aspartate beta-hydroxylase [EC:1.14.11.16]
KO:K00477	0.00	0.48	0.00	1.49	0.06	0.00	0.00	1.00E+00	2.05E-01	1.00E+00	1.14E-01	2.77E-01	1.00E+00	1.00E+00	phytanoyl-CoA hydroxylase [EC:1.14.11.18]
KO:K00478	0.00	0.00	0.00	0.00	4.38	0.00	0.00	1.00E+00	1.00E+00	1.00E+00	1.00E+00	1.71E-03	1.00E+00	1.00E+00	
KO:K00479	3.30	2.27	0.00	0.03	0.00	0.00	0.00	8.80E-02	1.31E-01	1.00E+00	6.28E-01	1.00E+00	1.00E+00	1.00E+00	Rieske 2Fe-2S family protein
KO:K00480	1.09	16.00	0.00	1.19	0.00	0.00	0.00	5.84E-01	5.14E-03	1.00E+00	5.48E-01	1.00E+00	1.00E+00	1.00E+00	salicylate hydroxylase [EC:1.14.13.1]
KO:K00481	0.28	19.79	0.00	1.75	0.00	0.00	0.00	8.44E-01	1.71E-03	1.00E+00	4.08E-01	1.00E+00	1.00E+00	1.00E+00	p-hydroxybenzoate 3-monooxygenase [EC:1.14.13.2]
KO:K00483	1.78	0.32	0.91	1.14	0.00	0.00	0.00	1.66E-01	4.48E-01	2.80E-01	2.39E-01	1.00E+00	1.00E+00	1.00E+00	4-hydroxyphenylacetate-3-hydroxylase large chain [EC:1.14.13.3]
KO:K00484	2.32	0.00	0.00	0.05	0.00	0.00	0.00	4.09E-02	1.00E+00	1.00E+00	3.98E-01	1.00E+00	1.00E+00	1.00E+00	4-hydroxyphenylacetate-3-hydroxylase small chain [EC:1.14.13.3]
KO:K00485	0.17	13.36	0.05	1.75	0.00	0.00	0.00	7.60E-01	1.34E-02	7.95E-01	3.33E-01	1.00E+00	1.00E+00	1.00E+00	dimethylaniline monooxygenase (N-oxide forming) [EC:1.14.13.8]
KO:K00486	0.26	1.28	0.00	1.71	0.00	0.24	0.00	5.05E-01	1.58E-01	1.00E+00	1.20E-01	1.00E+00	5.21E-01	1.00E+00	kynurenine 3-monooxygenase [EC:1.14.13.9]
KO:K00492	1.48	2.39	1.67	2.22	0.00	0.00	0.00	3.06E-01	1.70E-01	2.70E-01	1.90E-01	1.00E+00	1.00E+00	1.00E+00	
KO:K00493	0.16	9.80	0.00	0.04	0.00	0.00	0.00	6.83E-01	5.57E-03	1.00E+00	7.84E-01	1.00E+00	1.00E+00	1.00E+00	unspecific monooxygenase [EC:1.14.14.1]
KO:K00494	1.66	0.00	0.26	1.41	0.11	0.00	0.01	1.41E-01	1.00E+00	3.89E-01	1.58E-01	4.87E-01	1.00E+00	6.44E-01	alkanal monooxygenase (FMN-linked) [EC:1.14.14.3]
KO:K00495	0.00	0.00	0.00	2.45	0.00	0.00	0.00	1.00E+00	1.00E+00	1.00E+00	8.26E-02	1.00E+00	1.00E+00	1.00E+00	
KO:K00496	2.29	16.00	0.11	1.76	0.00	0.00	0.03	3.92E-01	6.86E-03	9.17E-01	5.20E-01	1.00E+00	1.00E+00	9.38E-01	alkane 1-monooxygenase [EC:1.14.15.3]
KO:K00499	0.00	8.85	0.00	0.00	0.00	0.00	0.13	1.00E+00	2.43E-02	1.00E+00	1.00E+00	1.00E+00	1.00E+00	4.70E-01	choline monooxygenase [EC:1.14.15.7]
KO:K00500	2.64	0.13	0.02	0.04	0.00	2.77	0.00	5.26E-02	6.02E-01	7.80E-01	7.26E-01	1.00E+00	4.84E-02	1.00E+00	phenylalanine-4-hydroxylase [EC:1.14.16.1]
KO:K00504	0.01	0.31	0.23	2.13	0.00	0.00	0.00	5.97E-01	3.15E-01	3.51E-01	7.70E-02	1.00E+00	1.00E+00	1.00E+00	peptidylglycine monooxygenase [EC:1.14.17.3]
KO:K00507	0.92	0.00	18.17	1.67	15.54	1.14	3.28	7.99E-01	9.58E-01	3.43E-03	6.32E-01	5.14E-03	7.56E-01	2.94E-01	stearoyl-CoA desaturase (delta-9 desaturase) [EC:1.14.19.1]
KO:K00508	2.22	0.61	0.00	2.30	0.00	9.53	0.11	2.54E-01	6.06E-01	1.00E+00	2.47E-01	1.00E+00	2.30E-02	7.59E-01	linoleoyl-CoA desaturase [EC:1.14.19.3]
KO:K00510	0.00	0.00	0.00	0.00	33.55	0.00	0.00	1.00E+00	1.00E+00	1.00E+00	5.42E-01	0.00E+00	1.00E+00	1.00E+00	heme oxygenase [EC:1.14.99.3]
KO:K00514	0.00	0.00	0.74	0.00	30.55	0.00	0.02	1.00E+00	1.00E+00	2.43E-01	6.44E-01	0.00E+00	1.00E+00	6.21E-01	zeta-carotene desaturase [EC:1.14.99.3]
KO:K00517	3.76	4.90	4.79	2.49	1.64	0.00	0.37	2.64E-01	1.65E-01	1.73E-01	4.49E-01	6.29E-01	1.00E+00	8.85E-01	
KO:K00518	0.00	5.27	0.00	0.00	5.75	0.00	0.00	1.00E+00	5.47E-02	1.00E+00	1.00E+00	4.89E-02	1.00E+00	1.00E+00	superoxide dismutase [EC:1.15.1.1]
KO:K00520	1.51	14.71	14.36	0.70	0.03	0.02	1.23	7.98E-01	8.43E-03	8.86E-03	9.11E-01	9.76E-01	9.79E-01	8.44E-01	mercuric reductase [EC:1.16.1.1]
KO:K00522	0.00	0.00	0.00	1.12	0.00	0.00	0.00	1.00E+00	1.00E+00	1.00E+00	1.43E-01	1.00E+00	1.00E+00	1.00E+00	ferritin heavy chain [EC:1.16.3.1]
KO:K00523	1.33	0.10	0.00	0.65	0.00	0.93	3.70	2.24E-01	6.84E-01	1.00E+00	3.85E-01	1.00E+00	3.01E-01	6.07E-02	CDP-4-dehydro-6-deoxyglucose reductase [EC:1.17.1.1]
KO:K00524	0.00	0.00	0.00	0.00	33.75	0.00	0.00	1.00E+00	1.00E+00	1.00E+00	1.00E+00	0.00E+00	1.00E+00	1.00E+00	ribonucleotide reductase, class II [EC:1.17.4.1]
KO:K00525	10.64	19.36	13.85	13.77	0.78	7.09	8.36	5.10E-01	1.14E-02	1.31E-01	1.35E-01	9.99E-01	9.15E-01	8.40E-01	ribonucleoside-diphosphate reductase alpha chain [EC:1.17.4.1]
KO:K00526	8.86	0.80	10.54	5.53	0.21	3.34	3.57	8.06E-02	9.23E-01	5.43E-02	2.55E-01	9.65E-01	6.02E-01	5.58E-01	ribonucleoside-diphosphate reductase beta chain [EC:1.17.4.1]

KO:K00527	0.00	0.00	0.00	0.02	0.00	0.00	6.28	1.00E+00	1.00E+00	1.00E+00	3.44E-01	1.00E+00	1.00E+00	5.29E-03	ribonucleoside-triphosphate reductase [EC:1.17.4.2]
KO:K00528	2.07	11.88	0.12	2.26	0.08	0.00	1.37	4.86E-01	1.76E-02	9.44E-01	4.44E-01	9.57E-01	1.00E+00	6.61E-01	ferredoxin--NADP+ reductase [EC:1.18.1.2]
KO:K00529	2.12	12.30	0.00	0.88	0.00	0.27	0.00	2.97E-01	9.57E-03	1.00E+00	6.54E-01	1.00E+00	8.44E-01	1.00E+00	ferredoxin--NAD+ reductase [EC:1.18.1.3]
KO:K00530	1.66	0.00	0.03	0.00	0.00	5.06	0.00	1.07E-01	1.00E+00	4.08E-01	1.00E+00	1.00E+00	4.06E-02	1.00E+00	
KO:K00535	0.00	0.00	0.00	0.00	33.13	0.00	0.00	1.00E+00	1.00E+00	1.00E+00	1.00E+00	0.00E+00	1.00E+00	1.00E+00	
KO:K00537	2.41	3.03	0.50	1.84	2.63	1.48	0.18	2.78E-01	1.70E-01	8.90E-01	4.42E-01	2.32E-01	5.69E-01	9.53E-01	arsenate reductase [EC:1.2.4.1]
KO:K00540	5.98	3.21	16.93	7.18	10.37	3.82	7.26	6.30E-01	8.92E-01	3.24E-02	4.66E-01	1.72E-01	8.56E-01	4.55E-01	
KO:K00543	0.00	0.00	2.94	0.70	0.00	0.00	0.00	1.00E+00	1.00E+00	3.53E-02	1.62E-01	1.00E+00	1.00E+00	1.00E+00	acetylserotonin N-methyltransferase [EC:2.1.1.4]
KO:K00544	2.67	0.10	0.00	2.58	0.00	0.00	0.00	1.29E-01	5.57E-01	1.00E+00	1.33E-01	1.00E+00	1.00E+00	1.00E+00	betaine-homocysteine S-methyltransferase [EC:2.1.1.5]
KO:K00547	0.30	7.66	0.00	2.13	0.02	0.00	0.00	7.18E-01	2.67E-02	1.00E+00	1.88E-01	8.46E-01	1.00E+00	1.00E+00	homocysteine S-methyltransferase [EC:2.1.1.1]
KO:K00548	9.44	21.98	6.63	7.27	6.79	1.63	2.45	3.04E-01	5.29E-03	7.22E-01	6.26E-01	6.99E-01	9.91E-01	9.79E-01	5-methyltetrahydrofolate--homocysteine methyltransferase
KO:K00549	4.06	7.82	0.01	1.64	0.00	0.00	1.99	1.88E-01	5.80E-02	9.07E-01	5.23E-01	1.00E+00	1.00E+00	4.42E-01	5-methyltetrahydropteroyltriglutamate--homocysteine
KO:K00551	1.98	0.02	0.00	0.18	0.02	4.07	0.00	9.07E-02	7.05E-01	1.00E+00	5.04E-01	6.96E-01	3.03E-02	1.00E+00	phosphatidylethanolamine N-methyltransferase [EC:2.1.1.17]
KO:K00554	7.68	4.30	11.55	7.70	3.33	7.13	3.82	2.54E-01	8.35E-01	5.16E-02	2.53E-01	9.12E-01	3.38E-01	8.79E-01	tRNA (guanine-N1-)-methyltransferase [EC:2.1.1.31]
KO:K00555	0.00	0.00	0.00	0.00	9.27	0.00	0.73	1.00E+00	1.00E+00	1.00E+00	1.00E+00	1.29E-03	1.00E+00	1.27E-01	tRNA (guanine-N2-)-methyltransferase [EC:2.1.1.32]
KO:K00556	0.28	0.01	0.02	0.03	14.78	0.00	0.06	4.47E-01	7.97E-01	7.92E-01	7.59E-01	0.00E+00	1.00E+00	7.19E-01	tRNA (guanosine-2'-O-)-methyltransferase [EC:2.1.1.34]
KO:K00557	2.40	0.19	0.00	0.01	0.00	0.00	0.24	2.00E-02	4.60E-01	1.00E+00	7.59E-01	1.00E+00	1.00E+00	3.98E-01	tRNA (uracil-5-)-methyltransferase [EC:2.1.1.35]
KO:K00558	1.38	1.66	4.66	0.11	0.10	0.30	0.04	2.26E-01	1.77E-01	3.16E-02	8.24E-01	8.38E-01	7.19E-01	8.81E-01	DNA (cytosine-5-)-methyltransferase [EC:2.1.1.37]
KO:K00559	0.00	0.33	0.00	0.00	0.00	0.00	0.00	1.00E+00	1.03E-01	1.00E+00	1.00E+00	1.00E+00	1.00E+00	1.00E+00	sterol 24-C-methyltransferase [EC:2.1.1.41]
KO:K00560	6.83	0.86	5.02	4.58	1.19	0.94	0.10	5.29E-02	8.90E-01	1.09E-01	1.37E-01	8.37E-01	8.80E-01	9.79E-01	thymidylate synthase [EC:2.1.1.45]
KO:K00563	1.17	0.02	0.57	0.03	0.00	0.00	0.11	4.41E-02	7.48E-01	1.09E-01	7.22E-01	1.00E+00	1.00E+00	4.95E-01	rRNA (guanine-N1-)-methyltransferase [EC:2.1.1.51]
KO:K00564	0.62	11.48	0.72	0.01	0.00	0.00	0.00	5.86E-01	1.57E-03	5.31E-01	9.32E-01	1.00E+00	1.00E+00	1.00E+00	ribosomal RNA small subunit methyltransferase C [EC:2.1.1.172]
KO:K00566	4.43	10.11	5.25	4.25	5.44	3.05	2.25	6.66E-01	3.61E-02	4.60E-01	7.09E-01	4.14E-01	8.91E-01	9.44E-01	tRNA (5-methylaminomethyl-2-thiouridylate)-methyltransferase
KO:K00567	0.48	0.27	0.07	2.88	0.07	0.51	0.28	4.45E-01	5.78E-01	7.82E-01	8.14E-02	7.82E-01	4.33E-01	5.69E-01	methylated-DNA-[protein]-cysteine S-methyltransferase [EC:2.1.1.63]
KO:K00568	6.95	5.65	0.56	4.06	0.00	0.09	7.07	7.17E-02	1.40E-01	9.56E-01	3.24E-01	1.00E+00	9.84E-01	6.90E-02	3-demethylubiquinone-9 3-methyltransferase [EC:2.1.1.- 2.1.1.64]
KO:K00569	2.01	0.02	0.00	1.03	0.00	0.00	0.02	5.64E-02	7.66E-01	1.00E+00	1.29E-01	1.00E+00	1.00E+00	7.38E-01	thiopurine S-methyltransferase [EC:2.1.1.67]
KO:K00570	0.12	0.00	0.00	0.00	0.00	0.00	0.00	6.41E-02	1.00E+00	1.00E+00	1.00E+00	1.00E+00	1.00E+00	1.00E+00	phosphatidyl-N-methylethanolamine N-methyltransferase [EC:2.1.1.71]
KO:K00571	0.49	5.40	0.00	0.93	0.00	1.31	0.41	6.26E-01	4.43E-02	1.00E+00	4.49E-01	1.00E+00	3.39E-01	6.61E-01	site-specific DNA-methyltransferase (adenine-specific) [EC:2.1.1.72]
KO:K00573	6.70	12.60	0.16	4.24	0.00	4.43	2.09	1.29E-01	1.77E-02	9.91E-01	4.47E-01	9.98E-01	4.13E-01	8.71E-01	protein-L-isoaspartate(D-aspartate) O-methyltransferase
KO:K00574	5.68	8.76	12.40	6.56	0.97	0.28	1.97	3.91E-01	1.13E-01	3.57E-02	2.68E-01	9.66E-01	9.90E-01	9.21E-01	cyclopropane-fatty-acyl-phospholipid synthase [EC:2.1.1.79]
KO:K00575	0.94	0.35	3.05	1.30	0.00	0.00	0.19	2.64E-01	6.28E-01	3.63E-02	1.61E-01	1.00E+00	1.00E+00	7.66E-01	chemotaxis protein methyltransferase CheR [EC:2.1.1.8]
KO:K00577	0.00	0.00	0.00	0.00	0.00	0.00	0.83	1.00E+00	1.00E+00	1.00E+00	1.00E+00	1.00E+00	1.00E+00	2.66E-02	tetrahydromethanopterin S-methyltransferase subunit A [EC:2.1.1.86]
KO:K00586	0.00	0.00	0.00	0.00	0.00	0.00	1.67	1.00E+00	1.00E+00	1.00E+00	1.00E+00	1.00E+00	1.00E+00	2.79E-02	diphthine synthase [EC:2.1.1.98]
KO:K00587	0.47	0.00	0.00	0.00	0.00	0.00	0.00	6.26E-02	1.00E+00	1.00E+00	1.00E+00	1.00E+00	1.00E+00	1.00E+00	protein-S-isoprenylcysteine O-methyltransferase [EC:2.1.1.1]
KO:K00588	0.96	0.10	0.00	0.57	0.05	0.34	0.00	1.42E-01	5.03E-01	1.00E+00	2.21E-01	5.75E-01	3.01E-01	1.00E+00	caffeoyl-CoA O-methyltransferase [EC:2.1.1.14]
KO:K00590	0.00	0.00	0.00	0.07	0.00	0.00	0.28	1.00E+00	1.00E+00	1.00E+00	1.52E-01	1.00E+00	1.00E+00	7.71E-02	site-specific DNA-methyltransferase (cytosine-N4-specific)
KO:K00594	0.00	0.00	0.00	0.51	0.00	0.00	0.00	1.00E+00	1.00E+00	1.00E+00	5.87E-02	1.00E+00	1.00E+00	1.00E+00	xylitol oxidase [EC:1.1.3.41]
KO:K00595	0.23	3.39	2.15	0.32	4.76	0.00	0.00	7.77E-01	8.00E-02	1.64E-01	7.30E-01	4.43E-02	1.00E+00	1.00E+00	precorrin-6Y C5,15-methyltransferase / precorrin-8W decarboxylase
KO:K00596	0.00	0.00	0.00	0.10	0.00	0.00	0.00	1.00E+00	1.00E+00	1.00E+00	1.43E-01	1.00E+00	1.00E+00	1.00E+00	2,2-dialkylglycine decarboxylase (pyruvate) [EC:4.1.1.64]
KO:K00598	0.01	0.08	0.00	1.41	0.00	0.00	0.00	4.59E-01	3.19E-01	1.00E+00	6.69E-02	1.00E+00	1.00E+00	1.00E+00	trans-aconitate 2-methyltransferase [EC:2.1.1.144]
KO:K00599	2.60	28.14	0.75	3.37	7.82	1.37	0.42	8.54E-01	2.71E-03	9.66E-01	7.68E-01	1.86E-01	9.38E-01	9.78E-01	
KO:K00600	10.36	11.91	8.78	11.77	4.46	4.42	4.16	2.19E-01	9.99E-02	4.67E-01	1.06E-01	9.57E-01	9.59E-01	9.68E-01	glycine hydroxymethyltransferase [EC:2.1.2.1]
KO:K00601	0.00	0.00	0.00	0.20	0.00	0.00	0.00	1.00E+00	1.00E+00	1.00E+00	1.43E-01	1.00E+00	1.00E+00	1.00E+00	phosphoribosylglycinamide formyltransferase [EC:2.1.2.2]
KO:K00602	7.21	10.42	5.95	4.86	10.26	3.05	2.72	3.28E-01	6.10E-02	6.07E-01	8.09E-01	6.64E-02	9.52E-01	9.64E-01	phosphoribosylaminoimidazolecarboxamide formyltransferase / IMP
KO:K00604	2.21	5.43	5.87	3.51	4.91	0.93	0.30	7.13E-01	1.17E-01	9.33E-02	3.65E-01	1.52E-01	9.40E-01	9.89E-01	methionyl-tRNA formyltransferase [EC:2.1.2.9]
KO:K00605	8.51	27.06	4.71	10.07	1.65	0.65	1.27	3.55E-01	4.29E-03	8.18E-01	2.24E-01	9.76E-01	9.93E-01	9.84E-01	aminomethyltransferase [EC:2.1.2.1]
KO:K00606	5.05	1.38	6.75	5.64	4.19	2.70	3.47	2.76E-01	9.09E-01	1.31E-01	2.09E-01	4.26E-01	7.31E-01	5.74E-01	3-methyl-2-oxobutanoate hydroxymethyltransferase [EC:2.1.2.11]
KO:K00607	0.00	0.00	0.45	0.05	0.00	0.81	0.00	1.00E+00	4.56E-01	6.76E-02	2.93E-01	1.00E+00	4.04E-02	1.00E+00	
KO:K00609	5.42	5.14	7.54	4.12	9.81	0.89	3.85	4.07E-01	4.70E-01	1.44E-01	6.88E-01	5.50E-02	9.81E-01	7.44E-01	aspartate carbamoyltransferase catalytic subunit [EC:2.1.3.2]
KO:K00610	0.00	0.00	0.00	0.00	0.00	0.00	2.92	1.00E+00	1.00E+00	1.00E+00	1.00E+00	1.00E+00	1.00E+00	1.14E-02	aspartate carbamoyltransferase regulatory subunit
KO:K00611	0.82	1.03	4.90	1.31	8.50	1.02	5.64	8.49E-01	8.01E-01	9.10E-02	7.23E-01	2.07E-02	8.06E-01	6.57E-02	ornithine carbamoyltransferase [EC:2.1.3.3]
KO:K00612	2.14	0.29	2.80	5.83	5.18	0.00	0.45	4.07E-01	8.59E-01	2.89E-01	8.07E-02	1.03E-01	1.00E+00	8.27E-01	carbamoyltransferase [EC:2.1.3.-]
KO:K00613	0.69	7.67	0.00	1.47	0.00	0.00	0.06	5.15E-01	5.94E-02	1.00E+00	3.44E-01	1.00E+00	1.00E+00	6.92E-01	glycine amidinotransferase [EC:2.1.4.1]
KO:K00614	0.07	0.00	1.11	0.05	0.00	0.00	0.05	3.32E-01	1.00E+00	2.31E-02	3.62E-01	1.00E+00	1.00E+00	3.56E-01	
KO:K00615	10.93	11.63	9.38	13.78	3.39	1.56	6.84	1.80E-01	1.23E-01	3.89E-01	4.60E-02	9.80E-01	9.96E-01	8.36E-01	transketolase [EC:2.2.1.1]
KO:K00616	4.55	7.18	1.49	5.70	5.49	1.56	1.45	4.13E-01	1.11E-01	9.33E-01	2.26E-01	2.51E-01	9.27E-01	9.35E-01	transaldolase [EC:2.2.1.2]
KO:K00617	0.17	0.98	0.00	0.95	0.00	11.50	0.00	5.27E-01	3.11E-01	1.00E+00	3.17E-01	1.00E+00	2.39E-02	1.00E+00	
KO:K00619	4.13	0.11	1.47	0.61	2.08	0.66	2.62	8.01E-02	8.90E-01	4.03E-01	7.32E-01	2.57E-01	7.14E-01	1.81E-01	amino-acid N-acetyltransferase [EC:2.3.1.1]
KO:K00620	4.92	6.16	5.72	2.93	8.75	0.66	3.23	3.72E-01	1.88E-01	2.44E-01	8.02E-01	5.47E-02	9.79E-01	7.49E-01	glutamate N-acetyltransferase / amino-acid N-acetyltransferase
KO:K00621	0.00	0.00	0.00	0.00	0.00	0.00	1.25	1.00E+00	1.00E+00	1.00E+00	1.00E+00	1.00E+00	1.00E+00	2.53E-02	glucosamine-phosphate N-acetyltransferase [EC:2.3.1.4]
KO:K00622	0.00	0.00	0.00	0.20	0.00	0.00	0.00	1.00E+00	1.00E+00	1.00E+00	1.43E-01	1.00E+00	1.00E+00	1.00E+00	arylamine N-acetyltransferase [EC:2.3.1.5]
KO:K00625	1.51	15.08	0.04	4.76	1.76	0.00	0.22	7.40E-01	1.06E-02	9.47E-01	2.04E-01	6.89E-01	1.00E+00	9.21E-01	phosphate acetyltransferase [EC:2.3.1.8]
KO:K00626	14.56	28.45	1.08	11.88	0.20	3.70	1.18	8.03E-02	1.71E-03	9.87E-01	1.68E-01	9.97E-01	9.24E-01	9.84E-01	acetyl-CoA C-acetyltransferase [EC:2.3.1.9]
KO:K00627	13.04	9.84	9.27	7.19	6.77	5.73	1.76	4.07E-02	1.81E-01	2.45E-01	6.47E-01	7.31E-01	8.67E-01	9.95E-01	pyruvate dehydrogenase E2 component (dihydrolipoamide
KO:K00631	3.44	0.73	0.44	0.07	0.00	0.00	0.01	3.76E-02	2.56E-01	4.03E-01	7.86E-01	1.00E+00	1.00E+00	8.82E-01	glycerol-3-phosphate O-acyltransferase [EC:2.3.1.15]
KO:K00632	9.81	7.58	1.34	4.34	0.00	5.26	0.09	4.50E-02	9.40E-02	8.97E-01	3.81E-01	1.00E+00	2.50E-01	9.80E-01	acetyl-CoA acyltransferase [EC:2.3.1.16]
KO:K00633	0.38	0.11	0.55	0.00	0.00	0.00	0.00	1.45E-01	2.59E-01	1.17E-01	1.00E+00	1.00E+00	1.00E+00	1.00E+00	galactoside O-acetyltransferase [EC:2.3.1.18]
KO:K00634	0.08	0.40	0.00	0.67	0.00	0.00	0.15	3.88E-01	2.19E-01	1.00E+00	1.58E-01	1.00E+00	1.00E+00	3.04E-01	phosphate butyryltransferase [EC:2.3.1.19]
KO:K00635	1.13	0.00	1.24	0.00	0.00	0.00	0.00	5.34E-02	1.00E+00	4.81E-02	5.92E-01	1.00E+00	1.00E+00	1.00E+00	diacylglycerol O-acyltransferase [EC:2.3.1.2]
KO:K00638	0.16	0.06	0.00	0.05	0.00	0.00	0.00	1.59E-01	2.85E-01	1.00E+00	3.02E-01	1.00E+00	1.00E+00	1.00E+00	chloramphenicol O-acetyltransferase [EC:2.3.1.28]
KO:K00639	3.37	2.86	0.11	3.39	1.28	8.88	0.02	2.78E-01	3.53E-01	9.29E-01	2.75E-01	6.96E-01	4.64E-02	9.58E-01	glycine C-acetyltransferase [EC:2.3.1.29]
KO:K00640	5.75	11.81	5.50	4.39	2.83	0.01	3.84	3.59E-01	3.76E-02	3.99E-01	6.02E-01	8.48E-01	9.98E-01	7.01E-01	serine O-acetyltransferase [EC:2.3.1.3]
KO:K00641	4.47	0.60	2.66	8.37	0.15	0.27	1.38	1.57E-01	8.86E-01	4.24E-01	3.54E-02	9.44E-01	9.29E-01	7.52E-01	homoserine O-acetyltransferase [EC:2.3.1.31]
KO:K00643	0.91	36.81	0.00	6.82	0.00	0.00	0.00	8.91E-01	4.29E-04	1.00E+00	1.55E-01	1.00E+00	1.00E+00	1.00E+00	5-aminolevulinate synthase [EC:2.3.1.37]
KO:K00645	8.68	10.58	7.60	7.19	4.53	0.54	1.79	1.20E-01	4.96E-02	2.23E-01	2.93E-01	8.41E-01	9.97E-01	9.81E-01	[acyl-carrier-protein] S-malonyltransferase [EC:2.3.1.39]
KO:K00647	9.32	11.78	4.25	7.17	0.00	3.22	3.17	1.25E-01	5.54E-02	7.54E-01	2.91E-01	1.00E+00	8.67E-01	8.70E-01	3-oxoacyl-[acyl-carrier-protein] synthase I [EC:2.3.1.41]
KO:K00648	8.92	9.20	11.87	6.69	4.45	1.11	1.01	1.47E-01	1.32E-01	5.56E-02	3.90E-01	7.76E-01	9.87E-01	9.89E-01	3-oxoacyl-[acyl-carrier-protein] synthase III [EC:2.3.1.18]
KO:K00651	0.70	6.54	0.00	0.39	13.48	0.00	0.42	7.43E-01	4.31E-02	1.00E+00	8.27E-01	6.43E-03	1.00E+00	8.19E-01	homoserine O-succinyltransferase [EC:2.3.1.46]
KO:K00652	0.73	0.13	8.17	1.15	2.26	0.04	2.11	6.19E-01	8.72E-01	1.41E-02	4.40E-01	1.75E-01	9.32E-01	1.98E-01	8-amino-7-oxononanoate synthase [EC:2.3.1.47]
KO:K00655	6.24	8.37	2.89	3.62	2.59	1.29	0.67	1.26E-01	5.20E-02	7.25E-01	5.51E-01	7.88E-01	9.46E-01	9.78E-01	1-acyl-sn-glycerol-3-phosphate acyltransferase [EC:2.3.1.51]
KO:K00656	0.00	0.00	1.16	0.01	0.00	0.00	0.00	1.00E+00	1.00E+00	6.29E-03	3.28E-01	1.00E+00	1.00E+00	1.00E+00	formate C-acetyltransferase [EC:2.3.1.54]
KO:K00657	0.62	1.16	0.00	0.04	0.00	0.00	0.00	1.42E-01	6.60E-02	1.00E+00	5.98E-01	1.00E+00	1.00E+00	1.00E+00	diamine N-acetyltransferase [EC:2.3.1.57]
KO:K00658	11.94	8.39	4.37	7.64	0.49	12.05	1.35	5.57E-02	2.31E-01	8.43E-01	3.18E-01	9.96E-01	5.33E-02	9.83E-01	2-oxoglutarate dehydrogenase E2 component (dihydrolipoamide
KO:K00660	0.01	0.00	0.55	0.23	4.16	0.00	0.00	5.65E-01	1.00E+00	1.82E-01	3.06E-01	1.77E-02	1.00E+00	1.00E+00	chalcone synthase [EC:2.3.1.74]
KO:K00661	0.12	0.00	0.87	0.01	0.00	0.00	0.39	2.65E-01	1.00E+00	4.94E-02	5.06E-01	1.00E+00	1.00E+00	1.13E-01	maltose O-acetyltransferase [EC:2.3.1.79]
KO:K00662	0.00	0.06	1.98	0.05	0.00	0.00	0.61	1.00E+00	3.91E-01	3.41E-02	4.10E-01	1.00E+00	1.00E+00	1.34E-01	aminoglycoside N3'-acetyltransferase [EC:2.3.1.81]
KO:K00663	0.00	0.00	0.00	0.31	0.00	0.00	0.00	1.00E+00	1.00E+00	1.00E+00	7.24E-02	1.00E+00	1.00E+00	1.00E+00	aminoglycoside N6'-acetyltransferase [EC:2.3.1.82]
KO:K00666	11.71	20.55	0.11	11.39	0.00	0.00	0.23	8.87E-02	1.59E-02	9.78E-01	9.47E-02	1.00E+00	1.00E+00	9.74E-01	fatty-acyl-CoA synthase [EC:6.2.1.-]
KO:K00672	0.00	0.00	2.82	0.04	0.00	0.00	2.81	5.00E-01	1.00E+00	4.10E-02	4.18E-01	1.00E+00	1.00E+00	4.13E-02	formylmethanofuran--tetrahydromethanopterin N-formyltransferase
KO:K00673	0.67	0.02	0.11	0.37	2.15	0.00	0.19	2.18E-01	7.53E-01	6.23E-01	3.55E-01	5.84E-02	1.00E+00	5.15E-01	arginine N-succinyltransferase [EC:2.3.1.19]
KO:K00674	9.27	11.46	1.31	11.22	0.00	1.66	3.98	9.94E-02	4.19E-02	9.81E-01	4.57E-02	1.00E+00	9.72E-01	8.33E-01	2,3,4,5-tetrahydropyridine-2-carboxylate N-succinyltransferase
KO:K00676	0.00	1.00	0.00	0.00	4.03	0.00	0.00	5.90E-01	1.24E-01	1.00E+00	5.65E-01	2.89E-02	1.00E+00	1.00E+00	ribosomal-protein-alanine N-acetyltransferase [EC:2.3.1.128]
KO:K00677	5.43	5.86	14.74	3.74	7.88	6.73	3.53	6.27E-01	5.34E-01	1.13E-02	8.76E-01	1.93E-01	3.62E-01	8.91E-01	UDP-N-acetylglucosamine acyltransferase [EC:2.3.1.129]
KO:K00680	4.55	4.71	1.43	3.54	0.07	3.61	0.05	2.02E-01	1.89E-01	7.04E-01	3.05E-01	9.40E-01	2.98E-01	9.51E-01	
KO:K00681	7.81	13.13	5.13	5.36	1.76	11.64	0.88	2.57E-01	4.19E-02	6.75E-01	6.38E-01	9.63E-01	6.36E-02	9.85E-01	gamma-glutamyltranspeptidase [EC:2.3.2.2]
KO:K00683	1.58	0.23	0.00	0.16	0.00	8.69	0.00	1.49E-01	5.39E-01	1.00E+00	5.92E-01	1.00E+00	1.21E-02	1.00E+00	glutaminyl-peptide cyclotransferase [EC:2.3.2.5]

KO:K00684	2.76	2.52	0.51	1.19	0.51	2.89	0.14	1.26E-01	1.53E-01	8.85E-01	5.96E-01	8.85E-01	1.09E-01	9.71E-01	leucyl/phenylalanyl-tRNA--protein transferase [EC:2.3.2.6]
KO:K00685	5.71	11.67	0.19	0.81	0.00	0.00	0.01	5.80E-02	9.00E-03	9.32E-01	8.05E-01	1.00E+00	1.00E+00	9.76E-01	arginine-tRNA-protein transferase [EC:2.3.2.8]
KO:K00688	0.52	0.04	23.20	0.27	17.94	0.00	1.34	7.54E-01	9.15E-01	1.14E-03	8.24E-01	3.43E-03	1.00E+00	5.09E-01	starch phosphorylase [EC:2.4.1.1]
KO:K00690	1.76	0.19	8.78	0.33	2.99	0.00	0.30	2.22E-01	8.30E-01	1.34E-02	7.57E-01	1.01E-01	1.00E+00	7.72E-01	sucrose phosphorylase [EC:2.4.1.7]
KO:K00693	0.00	0.00	0.00	1.02	0.00	0.00	0.00	1.00E+00	1.00E+00	1.00E+00	8.31E-02	1.00E+00	1.00E+00	1.00E+00	glycogen(starch) synthase [EC:2.4.1.11]
KO:K00694	0.00	0.01	0.00	0.00	5.58	0.00	0.02	4.95E-01	4.21E-01	1.00E+00	1.00E+00	7.00E-03	1.00E+00	3.71E-01	cellulose synthase (UDP-forming) [EC:2.4.1.12]
KO:K00696	0.00	0.00	0.10	0.00	29.11	0.00	0.00	6.42E-01	1.00E+00	4.75E-01	6.33E-01	0.00E+00	1.00E+00	1.00E+00	sucrose-phosphate synthase [EC:2.4.1.14]
KO:K00697	0.59	0.36	0.00	0.09	0.00	0.00	0.00	1.09E-01	1.80E-01	1.00E+00	3.97E-01	1.00E+00	1.00E+00	1.00E+00	alpha,alpha-trehalose-phosphate synthase (UDP-forming) [EC:2.4.1.15]
KO:K00700	1.91	1.42	7.69	0.77	14.60	1.58	0.31	5.72E-01	7.20E-01	3.29E-02	8.68E-01	2.57E-03	6.75E-01	9.35E-01	1,4-alpha-glucan branching enzyme [EC:2.4.1.18]
KO:K00701	0.39	0.00	4.07	0.02	0.00	0.00	0.00	1.96E-01	1.00E+00	3.91E-02	3.24E-01	1.00E+00	1.00E+00	1.00E+00	cyclomaltodextrin glucanotransferase [EC:2.4.1.19]
KO:K00702	0.06	0.00	0.00	0.05	0.00	0.00	0.00	1.07E-01	1.00E+00	1.00E+00	2.06E-01	1.00E+00	1.00E+00	1.00E+00	cellobiose phosphorylase [EC:2.4.1.2]
KO:K00703	3.39	2.24	2.05	1.68	13.24	0.40	0.01	2.69E-01	4.78E-01	5.21E-01	6.21E-01	1.11E-02	8.83E-01	9.76E-01	starch synthase [EC:2.4.1.21]
KO:K00705	0.34	0.00	8.70	0.16	7.81	0.19	0.03	5.71E-01	1.00E+00	1.01E-02	6.93E-01	1.31E-02	6.70E-01	8.23E-01	4-alpha-glucanotransferase [EC:2.4.1.25]
KO:K00712	0.00	0.12	0.92	0.00	0.00	0.00	0.00	1.00E+00	1.60E-01	3.76E-02	1.00E+00	1.00E+00	1.00E+00	1.00E+00	poly(glycerol-phosphate) alpha-glucosyltransferase [EC:2.4.1.52]
KO:K00721	1.79	0.13	6.06	1.60	14.64	0.00	0.20	4.25E-01	9.01E-01	5.39E-02	4.76E-01	6.29E-03	1.00E+00	8.78E-01	dolichol-phosphate mannosyltransferase [EC:2.4.1.83]
KO:K00729	0.00	0.00	0.00	1.33	0.00	0.00	0.00	1.00E+00	1.00E+00	1.00E+00	1.43E-01	1.00E+00	1.00E+00	1.00E+00	dolichyl-phosphate beta-glucosyltransferase [EC:2.4.1.117]
KO:K00737	0.00	0.00	0.00	1.73	0.00	0.00	0.00	1.00E+00	1.00E+00	1.00E+00	3.93E-02	1.00E+00	1.00E+00	1.00E+00	beta-1,4-mannosyl-glycoprotein
KO:K00743	0.00	0.56	0.00	0.00	0.00	0.00	0.00	1.00E+00	1.04E-01	1.00E+00	1.00E+00	1.00E+00	1.00E+00	1.00E+00	N-acetyllactosaminide 3-alpha-galactosyltransferase [EC:2.4.1.87]
KO:K00748	3.46	2.94	6.83	1.23	6.36	6.36	2.35	4.34E-01	5.83E-01	5.96E-02	9.19E-01	7.53E-02	7.53E-02	7.45E-01	lipid-A-disaccharide synthase [EC:2.4.1.182]
KO:K00752	0.00	0.11	0.00	0.00	0.00	0.00	0.00	1.00E+00	1.00E-01	1.00E+00	1.00E+00	1.00E+00	1.00E+00	1.00E+00	hyaluronan synthase [EC:2.4.1.212]
KO:K00754	1.45	1.50	7.10	2.59	9.73	1.83	3.32	8.01E-01	7.92E-01	8.00E-02	5.62E-01	3.54E-02	7.27E-01	4.09E-01	
KO:K00756	0.00	0.00	36.92	0.00	0.01	0.00	0.00	1.00E+00	1.00E+00	0.00E+00	1.00E+00	4.11E-01	1.00E+00	1.00E+00	pyrimidine-nucleoside phosphorylase [EC:2.4.2.2]
KO:K00757	0.82	0.44	0.00	0.18	0.00	0.00	0.04	9.61E-02	2.04E-01	1.00E+00	4.31E-01	1.00E+00	1.00E+00	6.76E-01	uridine phosphorylase [EC:2.4.2.3]
KO:K00758	0.33	15.71	1.14	0.65	0.00	0.00	0.00	8.43E-01	2.14E-03	5.58E-01	7.45E-01	1.00E+00	1.00E+00	1.00E+00	thymidine phosphorylase [EC:2.4.2.4]
KO:K00759	0.79	3.35	1.05	1.82	14.45	0.00	2.10	8.47E-01	2.18E-01	7.89E-01	5.74E-01	2.86E-03	1.00E+00	4.90E-01	adenine phosphoribosyltransferase [EC:2.4.2.7]
KO:K00760	0.71	5.21	2.47	0.89	0.27	0.22	0.27	6.89E-01	4.23E-02	1.76E-01	6.11E-01	8.54E-01	8.69E-01	8.54E-01	hypoxanthine phosphoribosyltransferase [EC:2.4.2.8]
KO:K00761	0.27	7.23	0.06	0.80	7.31	0.00	0.11	8.73E-01	1.84E-02	9.41E-01	6.95E-01	1.77E-02	1.00E+00	9.29E-01	uracil phosphoribosyltransferase [EC:2.4.2.9]
KO:K00762	5.91	3.44	7.66	4.00	3.29	2.47	1.77	1.62E-01	6.42E-01	7.41E-02	4.88E-01	6.80E-01	8.34E-01	9.11E-01	orotate phosphoribosyltransferase [EC:2.4.2.10]
KO:K00763	0.83	8.31	2.07	1.37	0.55	0.00	1.40	7.47E-01	3.66E-02	4.17E-01	5.96E-01	8.17E-01	1.00E+00	5.85E-01	nicotinate phosphoribosyltransferase [EC:2.4.2.11]
KO:K00764	9.80	10.67	9.99	7.07	7.22	5.57	4.99	2.31E-01	1.54E-01	2.10E-01	6.90E-01	6.67E-01	8.70E-01	9.05E-01	amidophosphoribosyltransferase [EC:2.4.2.14]
KO:K00765	4.57	3.59	13.51	2.70	6.93	1.01	3.91	4.19E-01	6.38E-01	1.26E-02	8.08E-01	1.31E-01	9.55E-01	5.63E-01	ATP phosphoribosyltransferase [EC:2.4.2.17]
KO:K00766	6.88	3.56	3.72	3.62	7.19	2.11	5.26	1.51E-01	7.04E-01	6.70E-01	6.91E-01	1.32E-01	9.01E-01	3.52E-01	anthranilate phosphoribosyltransferase [EC:2.4.2.18]
KO:K00767	1.35	0.33	2.53	1.71	4.32	3.83	2.40	6.08E-01	9.22E-01	2.28E-01	4.59E-01	6.56E-02	8.44E-02	2.52E-01	nicotinate-nucleotide pyrophosphorylase (carboxylating)
KO:K00768	1.71	26.48	0.01	1.23	0.00	0.00	0.00	6.05E-01	4.29E-04	9.59E-01	7.28E-01	1.00E+00	1.00E+00	1.00E+00	nicotinate-nucleotide--dimethylbenzimidazole
KO:K00769	0.64	11.26	0.00	0.45	2.65	0.00	0.00	6.82E-01	1.26E-02	1.00E+00	7.52E-01	1.80E-01	1.00E+00	9.39E-01	xanthine phosphoribosyltransferase [EC:2.4.2.22]
KO:K00772	0.64	3.54	0.23	2.72	7.83	0.03	1.86	8.68E-01	1.42E-01	9.46E-01	2.53E-01	2.10E-02	9.77E-01	4.84E-01	5'-methylthioadenosine phosphorylase [EC:2.4.2.28]
KO:K00773	7.57	7.91	6.42	3.30	3.19	2.07	3.65	1.20E-01	1.03E-01	2.26E-01	8.51E-01	8.63E-01	9.48E-01	8.06E-01	queuine tRNA-ribosyltransferase [EC:2.4.2.29]
KO:K00777	0.00	0.73	3.41	3.01	0.00	0.00	0.00	1.00E+00	3.03E-01	1.16E-01	1.32E-01	1.00E+00	1.00E+00	1.00E+00	
KO:K00782	0.32	0.00	0.19	0.72	0.19	0.00	0.33	3.02E-01	7.70E-01	4.36E-01	1.36E-01	4.36E-01	1.00E+00	2.90E-01	hypothetical protein
KO:K00783	5.84	5.41	0.04	0.98	1.53	2.44	0.75	6.34E-02	7.59E-02	9.82E-01	8.47E-01	7.21E-01	4.34E-01	8.85E-01	hypothetical protein
KO:K00784	7.02	0.37	0.00	2.98	2.63	0.68	0.17	4.94E-02	8.31E-01	1.00E+00	2.12E-01	2.55E-01	7.39E-01	8.79E-01	ribonuclease Z [EC:3.1.26.11]
KO:K00785	6.26	4.10	0.12	2.25	2.27	1.17	2.64	8.87E-02	2.41E-01	9.67E-01	6.17E-01	6.10E-01	8.49E-01	5.13E-01	beta-galactosamide-alpha-2,3-sialyltransferase [EC:2.4.99.-]
KO:K00786	1.22	7.27	1.54	1.04	0.00	0.22	0.22	5.45E-01	2.23E-02	4.23E-01	6.15E-01	1.00E+00	8.84E-01	8.84E-01	
KO:K00788	0.49	4.61	0.89	0.75	5.84	1.94	0.84	8.80E-01	6.34E-02	7.66E-01	8.13E-01	3.46E-02	3.84E-01	7.85E-01	thiamine-phosphate pyrophosphorylase [EC:2.5.1.3]
KO:K00789	9.60	8.99	15.78	9.21	7.77	0.36	8.31	2.65E-01	3.68E-01	1.57E-02	3.26E-01	6.18E-01	1.00E+00	5.06E-01	S-adenosylmethionine synthetase [EC:2.5.1.6]
KO:K00790	9.55	9.45	2.43	6.36	6.44	6.82	3.38	1.12E-01	1.17E-01	9.74E-01	5.80E-01	5.61E-01	4.77E-01	9.41E-01	UDP-N-acetylglucosamine 1-carboxyvinyltransferase [EC:2.5.1.7]
KO:K00791	4.66	1.57	4.93	1.25	1.45	0.71	1.12	6.29E-02	6.71E-01	5.46E-02	7.81E-01	7.13E-01	9.08E-01	8.18E-01	tRNA dimethylallyltransferase [EC:2.5.1.75]
KO:K00793	6.04	9.97	4.98	3.57	5.49	0.35	2.04	2.51E-01	4.19E-02	4.45E-01	7.54E-01	3.42E-01	9.94E-01	9.21E-01	riboflavin synthase alpha chain [EC:2.5.1.9]
KO:K00794	7.22	6.96	2.03	4.36	6.76	3.61	1.95	1.43E-01	1.60E-01	9.31E-01	6.06E-01	1.78E-01	7.60E-01	9.36E-01	riboflavin synthase beta chain [EC:2.5.1.-]
KO:K00795	2.02	5.29	1.81	4.25	0.04	0.13	2.33	5.62E-01	8.97E-02	6.26E-01	1.42E-01	9.86E-01	9.71E-01	4.71E-01	farnesyl diphosphate synthase [EC:2.5.1.1 2.5.1.1]
KO:K00796	4.91	4.83	1.35	2.27	6.48	1.81	1.63	1.55E-01	1.64E-01	9.26E-01	7.93E-01	6.59E-02	8.69E-01	8.89E-01	dihydropteroate synthase [EC:2.5.1.15]
KO:K00797	0.91	3.56	1.24	2.26	4.63	0.35	0.04	6.60E-01	1.70E-01	5.71E-01	3.27E-01	1.13E-01	8.07E-01	9.11E-01	spermidine synthase [EC:2.5.1.16]
KO:K00798	2.50	9.48	0.14	0.68	10.22	0.08	1.29	5.51E-01	2.91E-02	9.62E-01	9.04E-01	2.24E-02	9.71E-01	8.27E-01	cob(I)alamin adenosyltransferase [EC:2.5.1.17]
KO:K00799	5.26	20.57	0.62	6.02	6.45	0.01	0.50	4.80E-01	8.71E-03	9.69E-01	3.69E-01	3.12E-01	9.94E-01	9.74E-01	glutathione S-transferase [EC:2.5.1.18]
KO:K00800	3.78	8.84	8.31	3.78	7.04	3.04	4.31	8.06E-01	8.26E-02	1.05E-01	8.06E-01	2.04E-01	8.89E-01	7.17E-01	3-phosphoshikimate 1-carboxyvinyltransferase [EC:2.5.1.19]
KO:K00801	0.01	0.00	0.04	0.07	1.51	0.00	1.51	5.20E-01	1.00E+00	4.58E-01	4.14E-01	5.76E-02	1.00E+00	5.76E-02	farnesyl-diphosphate farnesyltransferase [EC:2.5.1.21]
KO:K00803	5.21	0.00	0.00	0.17	0.00	0.00	0.08	4.06E-02	6.30E-01	1.00E+00	4.10E-01	1.00E+00	1.00E+00	4.69E-01	alkyldihydroxyacetonephosphate synthase [EC:2.5.1.26]
KO:K00804	1.45	2.62	3.88	0.66	1.72	0.00	0.03	3.84E-01	1.81E-01	9.53E-02	6.56E-01	3.23E-01	1.00E+00	8.69E-01	geranylgeranyl diphosphate synthase, type III [EC:2.5.1.1 2.5.1.1

KO:K00805	2.96	0.00	0.00	0.02	0.00	0.00	0.14	4.71E-02	1.00E+00	1.00E+00	4.03E-01	1.00E+00	1.00E+00	3.10E-01	heptaprenyl diphosphate synthase [EC:2.5.1.3]
KO:K00806	5.40	6.65	3.56	3.07	5.89	3.23	1.30	2.14E-01	1.04E-01	6.76E-01	7.91E-01	1.56E-01	7.58E-01	9.68E-01	undecaprenyl diphosphate synthase [EC:2.5.1.31]
KO:K00808	0.00	0.63	3.19	0.03	0.00	0.00	0.00	6.55E-01	1.42E-01	1.57E-02	5.54E-01	1.00E+00	1.00E+00	1.00E+00	homospermidine synthase [EC:2.5.1.44]
KO:K00809	0.36	0.05	1.04	2.29	3.11	0.00	1.89	6.57E-01	8.12E-01	3.77E-01	1.59E-01	1.03E-01	1.00E+00	2.01E-01	deoxyhypusine synthase [EC:2.5.1.46]
KO:K00810	2.20	0.75	1.11	0.84	1.42	6.29	0.03	2.55E-01	5.91E-01	4.87E-01	5.61E-01	4.03E-01	6.14E-02	8.17E-01	
KO:K00811	1.24	0.97	0.00	0.79	0.00	7.07	0.00	2.86E-01	3.51E-01	1.00E+00	4.06E-01	1.00E+00	2.27E-02	1.00E+00	aspartate aminotransferase [EC:2.6.1.1]
KO:K00812	2.01	5.67	4.21	6.70	5.50	3.79	3.14	9.06E-01	2.55E-01	5.39E-01	1.54E-01	2.80E-01	6.33E-01	7.71E-01	aspartate aminotransferase [EC:2.6.1.1]
KO:K00813	9.89	0.09	5.77	0.12	0.00	0.00	0.00	2.26E-02	7.74E-01	5.93E-02	7.63E-01	1.00E+00	1.00E+00	1.00E+00	aspartate aminotransferase [EC:2.6.1.1]
KO:K00817	4.82	10.74	13.09	4.13	5.59	0.29	5.22	6.82E-01	6.11E-02	3.04E-02	7.90E-01	5.38E-01	9.96E-01	6.08E-01	histidinol-phosphate aminotransferase [EC:2.6.1.9]
KO:K00818	3.66	0.34	0.43	1.62	2.20	9.03	2.38	1.85E-01	8.70E-01	8.56E-01	5.48E-01	4.08E-01	2.60E-02	3.69E-01	acetylornithine aminotransferase [EC:2.6.1.11]
KO:K00819	1.96	1.24	0.00	3.64	0.03	8.38	0.00	3.33E-01	4.94E-01	1.00E+00	1.60E-01	8.28E-01	3.39E-02	1.00E+00	ornithine--oxo-acid transaminase [EC:2.6.1.13]
KO:K00820	9.28	6.91	10.88	5.46	8.04	3.03	4.74	1.17E-01	4.70E-01	5.50E-02	7.89E-01	2.46E-01	9.68E-01	8.80E-01	glucosamine--fructose-6-phosphate aminotransferase (isomerizing)
KO:K00821	2.25	17.24	9.80	5.14	3.07	0.00	0.24	8.67E-01	7.57E-03	6.73E-02	3.93E-01	7.65E-01	1.00E+00	9.84E-01	acetylornithine/N-succinyldiaminopimelate aminotransferase
KO:K00822	3.39	4.28	0.05	6.72	0.33	0.00	1.65	3.05E-01	2.14E-01	9.18E-01	9.09E-02	8.77E-01	1.00E+00	6.13E-01	beta-alanine--pyruvate transaminase [EC:2.6.1.18]
KO:K00823	1.34	0.14	0.04	0.68	0.00	0.00	0.42	1.42E-01	5.18E-01	6.13E-01	2.53E-01	1.00E+00	1.00E+00	3.45E-01	4-aminobutyrate aminotransferase [EC:2.6.1.19]
KO:K00824	3.78	12.52	0.56	1.73	0.00	1.36	1.66	2.89E-01	8.57E-03	9.27E-01	7.57E-01	1.00E+00	8.26E-01	7.70E-01	D-alanine transaminase [EC:2.6.1.21]
KO:K00825	0.00	0.00	0.00	5.31	0.00	0.00	0.00	1.00E+00	1.00E+00	1.00E+00	5.64E-02	1.00E+00	1.00E+00	1.00E+00	2-aminoadipate transaminase [EC:2.6.1.39]
KO:K00826	10.16	12.53	7.68	11.04	4.18	4.03	2.97	2.05E-01	7.67E-02	5.68E-01	1.40E-01	9.33E-01	9.40E-01	9.71E-01	branched-chain amino acid aminotransferase [EC:2.6.1.42]
KO:K00829	0.00	0.00	0.00	0.09	0.00	0.00	4.38	1.00E+00	1.00E+00	1.00E+00	2.38E-01	1.00E+00	1.00E+00	2.13E-02	diaminobutyrate-pyruvate transaminase [EC:2.6.1.46]
KO:K00830	0.77	4.02	0.00	0.82	7.36	0.00	2.04	5.95E-01	1.47E-01	1.00E+00	5.79E-01	6.10E-02	1.00E+00	3.29E-01	alanine--glyoxylate transaminase / serine--pyruvate aminotransferase
KO:K00831	8.45	9.33	11.49	9.52	0.02	2.49	2.83	1.71E-01	1.13E-01	4.70E-02	1.04E-01	9.99E-01	9.61E-01	9.48E-01	phosphoserine aminotransferase [EC:2.6.1.52]
KO:K00832	0.85	29.02	1.01	0.00	0.00	0.00	0.00	7.24E-01	8.57E-04	6.82E-01	9.17E-01	1.00E+00	1.00E+00	1.00E+00	aromatic-amino-acid transaminase [EC:2.6.1.57]
KO:K00833	0.43	0.01	0.86	0.23	5.95	0.00	7.87	6.39E-01	9.24E-01	3.97E-01	7.82E-01	1.76E-02	1.00E+00	7.14E-03	adenosylmethionine-8-amino-7-oxononanoate aminotransferase
KO:K00835	4.81	0.00	0.00	0.05	0.00	0.00	1.15	3.43E-02	1.00E+00	1.00E+00	5.95E-01	1.00E+00	1.00E+00	1.81E-01	valine--pyruvate aminotransferase [EC:2.6.1.66]
KO:K00836	0.12	0.17	0.59	0.64	0.09	1.51	0.04	5.95E-01	5.23E-01	2.45E-01	2.26E-01	6.25E-01	8.31E-02	7.06E-01	diaminobutyrate-2-oxoglutarate transaminase [EC:2.6.1.76]
KO:K00837	3.63	32.44	3.35	3.07	1.41	0.17	0.03	6.70E-01	1.29E-03	7.09E-01	7.46E-01	9.01E-01	9.70E-01	9.82E-01	
KO:K00839	4.96	0.42	5.70	1.20	1.42	0.00	4.72	1.47E-01	8.13E-01	1.18E-01	6.41E-01	5.87E-01	1.00E+00	1.58E-01	aminotransferase [EC:2.6.1.-]
KO:K00840	0.00	0.56	0.00	0.00	0.00	0.00	0.00	1.00E+00	4.81E-02	1.00E+00	1.00E+00	1.00E+00	1.00E+00	1.00E+00	succinylornithine aminotransferase [EC:2.6.1.81]
KO:K00842	0.68	17.89	1.48	0.34	0.00	0.00	0.12	7.46E-01	5.29E-03	5.10E-01	8.27E-01	1.00E+00	1.00E+00	8.75E-01	aminotransferase [EC:2.6.1.-]
KO:K00843	0.00	0.81	0.00	1.17	0.00	0.00	0.00	1.00E+00	1.56E-01	1.00E+00	1.14E-01	1.00E+00	1.00E+00	1.00E+00	
KO:K00845	4.27	2.58	1.93	0.54	11.33	0.00	0.11	1.08E-01	3.16E-01	4.92E-01	8.84E-01	8.14E-03	1.00E+00	9.55E-01	glucokinase [EC:2.7.1.2]
KO:K00846	0.00	0.00	0.00	0.82	0.00	0.00	0.00	1.00E+00	1.00E+00	1.00E+00	6.14E-02	1.00E+00	1.00E+00	1.00E+00	ketohexokinase [EC:2.7.1.3]
KO:K00847	5.75	1.19	2.07	3.12	14.29	0.00	3.79	1.52E-01	8.63E-01	7.24E-01	5.02E-01	1.21E-02	1.00E+00	3.75E-01	fructokinase [EC:2.7.1.4]
KO:K00848	0.00	0.00	8.79	0.09	3.83	0.00	2.07	1.00E+00	1.00E+00	2.09E-02	5.20E-01	7.50E-02	1.00E+00	1.41E-01	rhamnulokinase [EC:2.7.1.5]
KO:K00849	1.16	0.05	2.62	1.89	0.02	0.00	0.03	2.18E-01	7.95E-01	7.06E-02	1.18E-01	8.26E-01	1.00E+00	8.08E-01	galactokinase [EC:2.7.1.6]
KO:K00850	6.25	0.50	22.37	4.65	1.59	2.46	2.92	2.01E-01	9.45E-01	5.14E-03	3.83E-01	8.69E-01	7.57E-01	6.78E-01	6-phosphofructokinase [EC:2.7.1.11]
KO:K00851	0.04	0.03	0.46	2.05	0.00	0.00	0.09	6.07E-01	6.62E-01	2.43E-01	5.67E-02	1.00E+00	1.00E+00	5.17E-01	gluconokinase [EC:2.7.1.12]
KO:K00852	0.59	7.56	1.97	0.66	3.22	0.00	0.07	7.40E-01	3.44E-02	3.38E-01	7.16E-01	1.60E-01	1.00E+00	9.12E-01	ribokinase [EC:2.7.1.15]
KO:K00853	0.19	0.00	6.32	0.07	0.03	0.00	0.11	3.71E-01	1.00E+00	5.86E-03	4.72E-01	5.65E-01	1.00E+00	4.33E-01	L-ribulokinase [EC:2.7.1.16]
KO:K00854	0.26	7.89	1.55	1.64	11.58	0.00	0.05	9.06E-01	3.63E-02	6.00E-01	5.71E-01	1.23E-02	1.00E+00	9.52E-01	xylulokinase [EC:2.7.1.17]
KO:K00855	0.09	0.03	0.00	0.67	31.03	0.00	0.58	7.78E-01	8.28E-01	1.00E+00	4.72E-01	0.00E+00	1.00E+00	5.05E-01	phosphoribulokinase [EC:2.7.1.19]
KO:K00856	1.24	0.07	0.00	1.17	0.00	0.00	1.97	1.87E-01	6.58E-01	1.00E+00	1.98E-01	1.00E+00	1.00E+00	1.13E-01	adenosine kinase [EC:2.7.1.2]
KO:K00857	2.83	7.12	1.64	1.07	0.00	2.59	0.00	2.21E-01	2.73E-02	5.30E-01	7.27E-01	1.00E+00	2.66E-01	1.00E+00	thymidine kinase [EC:2.7.1.21]
KO:K00858	5.67	5.23	5.11	3.62	8.32	3.90	3.55	3.47E-01	4.34E-01	4.56E-01	7.75E-01	9.09E-02	7.26E-01	7.88E-01	NAD+ kinase [EC:2.7.1.23]
KO:K00859	2.52	1.42	2.48	1.91	7.24	0.00	1.20	2.85E-01	6.76E-01	2.94E-01	4.85E-01	1.90E-02	1.00E+00	7.52E-01	dephospho-CoA kinase [EC:2.7.1.24]
KO:K00860	4.12	0.06	6.34	1.22	6.34	0.03	0.98	1.21E-01	9.17E-01	5.54E-02	5.88E-01	5.54E-02	9.45E-01	6.65E-01	adenylylsulfate kinase [EC:2.7.1.25]
KO:K00862	0.00	0.57	0.00	1.37	0.00	0.00	0.00	1.00E+00	1.76E-01	1.00E+00	1.05E-01	1.00E+00	1.00E+00	1.00E+00	erythritol kinase [EC:2.7.1.27]
KO:K00863	0.01	4.70	0.00	0.69	0.00	0.00	0.00	7.02E-01	5.71E-02	1.00E+00	2.43E-01	1.00E+00	1.00E+00	1.00E+00	dihydroxyacetone kinase [EC:2.7.1.29]
KO:K00864	4.54	10.09	3.00	3.50	2.33	1.49	0.28	3.22E-01	2.91E-02	6.65E-01	5.52E-01	8.01E-01	9.05E-01	9.82E-01	glycerol kinase [EC:2.7.1.3]
KO:K00865	0.08	0.02	14.13	0.02	2.14	0.00	0.04	5.63E-01	7.02E-01	4.43E-03	6.93E-01	1.01E-01	1.00E+00	6.13E-01	glycerate kinase [EC:2.7.1.31]
KO:K00867	0.25	0.00	0.00	0.19	0.00	0.00	0.00	1.28E-01	1.00E+00	1.00E+00	1.59E-01	1.00E+00	1.00E+00	1.00E+00	type I pantothenate kinase [EC:2.7.1.33]
KO:K00868	1.14	0.00	0.00	0.79	0.00	0.00	0.00	1.08E-01	1.00E+00	1.00E+00	1.55E-01	1.00E+00	1.00E+00	1.00E+00	pyridoxine kinase [EC:2.7.1.35]
KO:K00869	1.91	1.31	0.00	0.62	0.00	16.05	0.03	2.69E-01	3.86E-01	1.00E+00	5.84E-01	1.00E+00	6.57E-03	7.73E-01	mevalonate kinase [EC:2.7.1.36]
KO:K00870	0.00	0.00	0.21	0.00	0.83	0.00	0.00	1.00E+00	1.00E+00	7.94E-02	1.00E+00	1.70E-02	1.00E+00	1.00E+00	protein kinase [EC:2.7.1.37]
KO:K00872	0.24	0.11	0.08	0.11	8.42	3.26	0.10	6.03E-01	7.20E-01	7.43E-01	7.17E-01	7.57E-03	4.40E-02	7.23E-01	homoserine kinase [EC:2.7.1.39]
KO:K00873	6.98	11.66	7.52	3.12	5.28	6.10	1.98	2.94E-01	3.71E-02	2.28E-01	9.14E-01	6.36E-01	4.60E-01	9.66E-01	pyruvate kinase [EC:2.7.1.4]
KO:K00874	1.58	1.41	13.54	0.66	0.95	0.00	0.12	3.83E-01	4.35E-01	4.14E-03	6.93E-01	5.93E-01	1.00E+00	8.83E-01	2-dehydro-3-deoxygluconokinase [EC:2.7.1.45]
KO:K00875	0.00	0.11	0.00	0.00	0.00	0.00	0.00	1.00E+00	1.00E-01	1.00E+00	1.00E+00	1.00E+00	1.00E+00	1.00E+00	D-ribulokinase [EC:2.7.1.47]
KO:K00876	0.54	0.09	0.06	0.48	6.46	0.42	0.00	3.85E-01	7.43E-01	7.77E-01	4.19E-01	1.24E-02	4.57E-01	1.00E+00	uridine kinase [EC:2.7.1.48]
KO:K00877	0.03	0.21	0.00	0.04	0.00	0.00	1.16	4.30E-01	2.35E-01	1.00E+00	4.02E-01	1.00E+00	1.00E+00	5.24E-02	hydroxymethylpyrimidine kinase [EC:2.7.1.49]
KO:K00878	0.75	0.68	0.00	0.00	0.00	0.00	0.00	1.05E-01	1.17E-01	1.00E+00	5.99E-01	1.00E+00	1.00E+00	1.00E+00	hydroxyethylthiazole kinase [EC:2.7.1.5]
KO:K00882	0.00	0.00	1.88	0.00	0.00	0.00	0.00	1.00E+00	1.00E+00	3.71E-03	1.00E+00	1.00E+00	1.00E+00	1.00E+00	1-phosphofructokinase [EC:2.7.1.56]
KO:K00883	0.39	10.58	0.01	1.53	0.00	0.00	0.00	7.69E-01	4.71E-03	9.14E-01	3.03E-01	1.00E+00	1.00E+00	1.00E+00	2-dehydro-3-deoxygalactonokinase [EC:2.7.1.58]
KO:K00884	0.03	1.08	0.57	0.91	0.00	0.00	0.14	6.11E-01	1.74E-01	2.67E-01	1.98E-01	1.00E+00	1.00E+00	4.82E-01	N-acetylglucosamine kinase [EC:2.7.1.59]
KO:K00885	0.00	0.01	0.00	0.02	0.00	0.00	1.40	1.00E+00	3.96E-01	1.00E+00	3.45E-01	1.00E+00	1.00E+00	1.13E-02	N-acylmannosamine kinase [EC:2.7.1.6]
KO:K00886	0.23	1.63	0.00	0.11	0.00	0.00	0.12	3.40E-01	9.64E-02	1.00E+00	4.32E-01	1.00E+00	1.00E+00	4.27E-01	polyphosphate glucokinase [EC:2.7.1.63]
KO:K00891	5.42	5.38	2.63	1.84	4.92	2.33	2.05	1.63E-01	1.65E-01	6.85E-01	8.42E-01	2.17E-01	7.49E-01	8.04E-01	shikimate kinase [EC:2.7.1.71]
KO:K00892	0.00	0.06	0.00	0.05	0.00	0.00	0.00	1.00E+00	1.46E-01	1.00E+00	1.90E-01	1.00E+00	1.00E+00	1.00E+00	inosine kinase [EC:2.7.1.73]
KO:K00895	0.00	0.09	10.85	0.02	0.00	0.00	0.76	6.65E-01	4.89E-01	4.86E-03	6.20E-01	1.00E+00	1.00E+00	1.87E-01	pyrophosphate--fructose-6-phosphate 1-phosphotransferase
KO:K00899	0.00	0.79	0.00	0.39	0.00	4.96	0.00	1.00E+00	1.83E-01	1.00E+00	3.08E-01	1.00E+00	1.87E-02	1.00E+00	5-methylthioribose kinase [EC:2.7.1.1]
KO:K00901	1.10	0.70	1.19	0.30	4.75	0.00	0.35	3.22E-01	4.66E-01	2.96E-01	6.64E-01	4.64E-02	1.00E+00	6.32E-01	diacylglycerol kinase [EC:2.7.1.17]
KO:K00903	2.84	0.48	0.28	0.37	0.00	0.00	0.00	5.37E-02	3.95E-01	5.21E-01	4.66E-01	1.00E+00	1.00E+00	1.00E+00	protein-tyrosine kinase [EC:2.7.1.-]
KO:K00904	5.04	0.00	1.46	0.48	0.00	0.00	0.00	3.27E-02	1.00E+00	1.74E-01	4.36E-01	1.00E+00	1.00E+00	1.00E+00	deoxyguanosine kinase [EC:2.7.1.113]
KO:K00906	6.40	0.00	0.00	0.25	0.00	0.00	0.00	1.96E-02	1.00E+00	1.00E+00	4.47E-01	1.00E+00	1.00E+00	1.00E+00	isocitrate dehydrogenase kinase/phosphatase [EC:2.7.11.5 3.1.3.-]
KO:K00908	0.03	0.00	0.00	0.22	0.00	0.00	0.00	2.12E-01	1.00E+00	1.00E+00	1.11E-01	1.00E+00	1.00E+00	1.00E+00	Ca2+/calmodulin-dependent protein kinase [EC:2.7.11.17]
KO:K00912	5.83	2.54	6.51	1.79	0.50	2.54	1.60	6.53E-02	5.58E-01	4.79E-02	7.91E-01	9.71E-01	5.58E-01	8.31E-01	tetraacyldisaccharide 4'-kinase [EC:2.7.1.13]
KO:K00917	0.51	0.37	0.00	0.10	0.00	0.00	0.00	1.54E-01	1.97E-01	1.00E+00	3.18E-01	1.00E+00	1.00E+00	1.00E+00	tagatose 6-phosphate kinase [EC:2.7.1.144]
KO:K00919	5.38	1.24	2.82	2.64	5.01	0.80	1.70	9.81E-02	8.36E-01	4.31E-01	4.81E-01	1.19E-01	9.06E-01	7.41E-01	4-diphosphocytidyl-2-C-methyl-D-erythritol kinase [EC:2.7.1.148]
KO:K00924	0.27	0.05	33.41	0.58	1.43	1.04	1.31	7.64E-01	8.62E-01	1.71E-03	6.48E-01	4.43E-01	5.25E-01	4.69E-01	
KO:K00925	0.01	0.67	33.29	0.18	2.28	0.13	0.01	8.48E-01	5.18E-01	4.29E-04	6.98E-01	2.01E-01	7.15E-01	8.76E-01	acetate kinase [EC:2.7.2.1]
KO:K00926	0.02	0.00	0.00	0.51	0.00	0.00	0.00	3.13E-01	1.00E+00	1.00E+00	5.89E-02	1.00E+00	1.00E+00	1.00E+00	carbamate kinase [EC:2.7.2.2]
KO:K00927	8.03	4.57	7.90	6.11	8.30	6.08	3.69	1.71E-01	8.25E-01	1.86E-01	5.17E-01	1.47E-01	5.21E-01	9.10E-01	phosphoglycerate kinase [EC:2.7.2.3]
KO:K00928	9.37	7.94	10.25	7.15	4.50	2.28	4.76	1.59E-01	3.22E-01	1.08E-01	4.50E-01	8.73E-01	9.72E-01	8.47E-01	aspartate kinase [EC:2.7.2.4]
KO:K00930	7.17	3.85	7.72	3.83	6.40	0.99	4.65	1.49E-01	6.85E-01	1.15E-01	6.88E-01	2.22E-01	9.68E-01	5.14E-01	acetylglutamate kinase [EC:2.7.2.8]
KO:K00931	6.18	7.04	7.63	3.97	2.51	1.30	3.66	2.35E-01	1.51E-01	1.15E-01	6.70E-01	8.85E-01	9.61E-01	7.34E-01	glutamate 5-kinase [EC:2.7.2.11]
KO:K00934	0.00	0.00	17.14	0.00	0.00	0.00	0.48	1.00E+00	1.00E+00	5.57E-03	1.00E+00	1.00E+00	1.00E+00	2.01E-01	arginine kinase [EC:2.7.3.3]
KO:K00935	0.00	0.00	0.63	0.00	0.00	0.00	0.00	1.00E+00	1.00E+00	1.30E-02	1.00E+00	1.00E+00	1.00E+00	1.00E+00	
KO:K00936	1.52	18.79	6.50	3.48	4.34	5.82	1.51	9.37E-01	8.86E-03	3.02E-01	7.84E-01	6.50E-01	3.98E-01	9.37E-01	
KO:K00937	3.85	1.52	5.35	4.30	7.76	3.11	1.08	3.72E-01	8.77E-01	1.59E-01	2.91E-01	5.64E-02	5.50E-01	9.22E-01	polyphosphate kinase [EC:2.7.4.1]
KO:K00938	0.12	0.00	0.00	0.00	0.00	0.00	0.00	6.19E-02	1.00E+00	1.00E+00	1.00E+00	1.00E+00	1.00E+00	1.00E+00	phosphomevalonate kinase [EC:2.7.4.2]
KO:K00939	7.04	12.82	10.76	4.33	3.95	2.47	2.40	3.52E-01	2.43E-02	6.13E-02	8.50E-01	8.86E-01	9.64E-01	9.65E-01	adenylate kinase [EC:2.7.4.3]
KO:K00940	8.58	6.15	15.97	4.73	2.58	1.57	2.29	1.36E-01	3.86E-01	1.23E-02	6.53E-01	9.11E-01	9.57E-01	9.25E-01	nucleoside-diphosphate kinase [EC:2.7.4.6]
KO:K00941	0.40	1.67	0.01	3.18	1.00	0.53	1.78	7.98E-01	2.89E-01	9.66E-01	9.63E-02	5.26E-01	7.34E-01	2.65E-01	phosphomethylpyrimidine kinase [EC:2.7.4.7]
KO:K00942	2.72	2.55	4.71	3.24	4.97	0.39	0.34	4.02E-01	4.51E-01	9.86E-02	2.66E-01	8.69E-02	9.72E-01	9.77E-01	guanylate kinase [EC:2.7.4.8]
KO:K00943	2.26	2.09	4.97	1.72	7.58	0.69	2.58	5.78E-01	6.25E-01	9.91E-02	7.42E-01	3.36E-02	9.31E-01	4.80E-01	dTMP kinase [EC:2.7.4.9]
KO:K00945	6.77	3.00	3.44	2.42	0.60	3.70	2.83	6.01E-02	5.49E-01	4.25E-01	7.15E-01	9.67E-01	3.62E-01	6.02E-01	cytidylate kinase [EC:2.7.4.14]

KO:K00946	7.12	0.69	0.70	2.58	3.36	6.32	2.69	5.29E-02	9.22E-01	9.21E-01	5.54E-01	3.65E-01	7.43E-02	5.27E-01	thiamine-monophosphate kinase [EC:2.7.4.16]
KO:K00947	0.01	0.00	6.57	0.22	1.64	0.00	0.00	6.28E-01	1.00E+00	7.00E-03	3.84E-01	7.06E-02	1.00E+00	1.00E+00	
KO:K00948	14.57	6.50	6.31	10.05	6.33	6.38	3.27	3.74E-02	7.78E-01	8.02E-01	2.18E-01	8.00E-01	7.92E-01	9.70E-01	ribose-phosphate pyrophosphokinase [EC:2.7.6.1]
KO:K00949	0.81	8.23	0.00	0.01	0.00	0.00	0.00	3.31E-01	1.34E-02	1.00E+00	8.19E-01	1.00E+00	1.00E+00	1.00E+00	thiamine pyrophosphokinase [EC:2.7.6.2]
KO:K00950	1.68	1.46	2.86	0.99	2.86	2.79	1.73	4.90E-01	5.68E-01	1.94E-01	7.49E-01	1.93E-01	2.04E-01	4.69E-01	2-amino-4-hydroxy-6-hydroxymethyldihydropteridine diphosphokinase
KO:K00951	11.23	10.70	0.02	4.90	0.00	2.80	2.25	3.43E-02	4.04E-02	9.92E-01	4.04E-01	1.00E+00	7.89E-01	8.57E-01	GTP pyrophosphokinase [EC:2.7.6.5]
KO:K00952	0.00	0.00	0.00	0.00	0.00	0.00	3.75	1.00E+00	1.00E+00	1.00E+00	1.00E+00	1.00E+00	1.00E+00	6.71E-03	nicotinamide-nucleotide adenylyltransferase [EC:2.7.7.1]
KO:K00954	6.38	5.25	3.98	2.78	2.90	2.04	1.52	1.21E-01	2.15E-01	4.34E-01	7.13E-01	6.88E-01	8.41E-01	9.00E-01	pantetheine-phosphate adenylyltransferase [EC:2.7.7.3]
KO:K00955	0.12	0.34	0.00	1.44	0.00	7.32	0.00	5.62E-01	4.42E-01	1.00E+00	1.75E-01	1.00E+00	1.90E-02	1.00E+00	bifunctional enzyme CysN/CysC [EC:2.7.7.4 2.7.1.25]
KO:K00956	1.39	0.53	16.10	2.14	0.03	7.75	0.94	6.38E-01	8.49E-01	5.71E-03	4.33E-01	9.55E-01	4.49E-02	7.61E-01	sulfate adenylyltransferase subunit 1 [EC:2.7.7.4]
KO:K00957	1.68	0.51	12.89	3.00	0.00	3.53	0.43	5.09E-01	8.51E-01	7.29E-03	2.23E-01	9.65E-01	1.71E-01	8.67E-01	sulfate adenylyltransferase subunit 2 [EC:2.7.7.4]
KO:K00958	3.97	3.29	0.60	0.35	19.39	0.00	0.83	1.93E-01	2.72E-01	8.57E-01	9.00E-01	3.14E-03	1.00E+00	8.17E-01	sulfate adenylyltransferase [EC:2.7.7.4]
KO:K00960	0.00	3.44	0.00	0.00	0.00	0.00	0.00	1.00E+00	1.81E-02	1.00E+00	1.00E+00	1.00E+00	1.00E+00	1.00E+00	DNA-directed RNA polymerase [EC:2.7.7.6]
KO:K00962	13.43	15.43	10.63	10.76	9.05	6.76	6.96	1.59E-01	4.87E-02	6.24E-01	5.96E-01	8.60E-01	9.65E-01	9.60E-01	polyribonucleotide nucleotidyltransferase [EC:2.7.7.8]
KO:K00963	11.58	1.05	0.25	4.82	0.31	0.69	2.28	1.86E-02	8.56E-01	9.55E-01	1.76E-01	9.50E-01	9.02E-01	5.97E-01	UTP--glucose-1-phosphate uridylyltransferase [EC:2.7.7.9]
KO:K00964	0.01	0.00	0.00	0.82	0.00	0.00	0.00	1.82E-01	1.00E+00	1.00E+00	1.22E-01	1.00E+00	1.00E+00	1.00E+00	galactose-1-phosphate uridylyltransferase [EC:2.7.7.1]
KO:K00965	1.06	0.01	0.00	0.13	0.45	0.00	2.44	1.37E-01	7.58E-01	1.00E+00	5.79E-01	3.06E-01	1.00E+00	4.64E-02	UDPglucose--hexose-1-phosphate uridylyltransferase [EC:2.7.7.12]
KO:K00966	2.31	0.16	0.02	1.78	21.01	0.00	0.63	2.60E-01	8.92E-01	9.50E-01	3.77E-01	2.86E-04	1.00E+00	7.69E-01	mannose-1-phosphate guanylyltransferase [EC:2.7.7.13]
KO:K00968	0.04	1.03	0.00	0.00	0.00	0.00	0.00	1.96E-01	9.41E-02	1.00E+00	1.00E+00	1.00E+00	1.00E+00	1.00E+00	choline-phosphate cytidylyltransferase [EC:2.7.7.15]
KO:K00969	4.48	4.23	1.15	1.44	1.79	0.51	0.72	9.63E-02	1.12E-01	8.08E-01	7.18E-01	6.05E-01	9.24E-01	8.91E-01	nicotinate-nucleotide adenylyltransferase [EC:2.7.7.18]
KO:K00970	5.74	2.26	1.29	2.86	0.67	0.45	2.83	6.76E-02	5.41E-01	8.21E-01	3.75E-01	9.13E-01	9.39E-01	3.81E-01	poly(A) polymerase [EC:2.7.7.19]
KO:K00971	2.15	2.70	6.27	0.26	17.12	0.48	0.48	5.15E-01	3.69E-01	6.27E-02	9.30E-01	2.71E-03	8.99E-01	8.99E-01	mannose-1-phosphate guanylyltransferase [EC:2.7.7.22]
KO:K00972	0.00	0.00	1.34	0.00	1.93	0.00	0.02	1.00E+00	1.00E+00	3.29E-02	1.00E+00	2.96E-02	1.00E+00	3.32E-01	UDP-N-acetylglucosamine pyrophosphorylase [EC:2.7.7.23]
KO:K00973	4.17	2.80	2.04	3.49	6.54	10.02	0.96	3.53E-01	6.62E-01	8.16E-01	4.97E-01	1.14E-01	3.04E-02	9.35E-01	glucose-1-phosphate thymidylyltransferase [EC:2.7.7.24]
KO:K00974	2.55	0.91	2.20	0.88	6.81	0.27	2.02	2.43E-01	7.69E-01	3.26E-01	7.78E-01	2.44E-02	9.23E-01	3.75E-01	tRNA nucleotidyltransferase (CCA-adding enzyme) [EC:2.7.7.25
KO:K00975	0.32	0.58	18.30	0.30	20.03	0.08	0.53	8.53E-01	7.88E-01	2.43E-03	8.57E-01	2.00E-03	9.13E-01	8.01E-01	glucose-1-phosphate adenylyltransferase [EC:2.7.7.27]
KO:K00978	0.80	0.13	4.13	1.94	1.49	0.00	3.20	5.90E-01	8.42E-01	7.47E-02	2.45E-01	3.45E-01	1.00E+00	1.14E-01	glucose-1-phosphate cytidylyltransferase [EC:2.7.7.33]
KO:K00979	5.27	1.98	3.07	2.78	1.72	2.00	2.65	1.08E-01	7.39E-01	4.29E-01	5.06E-01	7.99E-01	7.31E-01	5.41E-01	3-deoxy-manno-octulosonate cytidylyltransferase (CMP-KDO synthetase)
KO:K00980	2.45	0.00	0.04	0.00	0.00	0.00	1.67	8.23E-02	5.76E-01	4.24E-01	1.00E+00	1.00E+00	1.00E+00	9.70E-02	glycerol-3-phosphate cytidylyltransferase [EC:2.7.7.39]
KO:K00981	5.90	3.55	7.43	1.77	8.51	0.80	0.69	1.18E-01	4.55E-01	5.67E-02	8.61E-01	3.81E-02	9.64E-01	9.70E-01	phosphatidate cytidylyltransferase [EC:2.7.7.41]
KO:K00982	4.58	6.72	0.88	1.66	0.04	1.56	0.88	1.19E-01	4.29E-02	8.72E-01	6.93E-01	9.82E-01	7.21E-01	8.72E-01	glutamate-ammonia-ligase adenylyltransferase [EC:2.7.7.42]
KO:K00983	1.25	1.17	0.00	2.03	0.65	12.38	0.24	5.06E-01	5.37E-01	1.00E+00	2.83E-01	7.30E-01	6.14E-03	8.53E-01	N-acylneuraminate cytidylyltransferase [EC:2.7.7.43]
KO:K00986	0.15	1.01	0.47	0.02	0.00	2.31	2.06	5.43E-01	2.15E-01	3.49E-01	7.14E-01	1.00E+00	9.76E-02	1.11E-01	RNA-directed DNA polymerase [EC:2.7.7.49]
KO:K00988	0.00	0.00	0.00	0.00	11.88	0.00	0.00	1.00E+00	1.00E+00	1.00E+00	1.00E+00	5.71E-04	1.00E+00	1.00E+00	ATP adenylyltransferase [EC:2.7.7.53]
KO:K00989	5.45	4.58	8.37	2.79	2.73	0.47	0.52	1.62E-01	2.53E-01	5.07E-02	6.24E-01	6.38E-01	9.56E-01	9.53E-01	ribonuclease PH [EC:2.7.7.56]
KO:K00990	4.59	4.37	12.71	2.40	0.59	2.38	1.73	2.66E-01	3.02E-01	9.57E-03	7.55E-01	9.62E-01	7.58E-01	8.63E-01	[protein-PII] uridylyltransferase [EC:2.7.7.59]
KO:K00991	2.56	0.01	4.75	1.04	8.44	2.61	0.79	2.38E-01	9.58E-01	7.86E-02	6.55E-01	2.21E-02	2.29E-01	7.39E-01	2-C-methyl-D-erythritol 4-phosphate cytidylyltransferase
KO:K00992	0.03	0.00	18.55	0.01	0.00	0.00	0.24	5.55E-01	1.00E+00	3.43E-03	5.80E-01	1.00E+00	1.00E+00	3.46E-01	
KO:K00995	4.13	4.06	7.67	2.72	4.03	0.87	5.03	4.28E-01	4.41E-01	8.76E-02	7.34E-01	4.47E-01	9.47E-01	2.71E-01	CDP-diacylglycerol--glycerol-3-phosphate 3-phosphatidyltransferase
KO:K00996	0.17	0.04	0.32	0.33	13.74	0.74	0.88	7.02E-01	8.14E-01	5.97E-01	5.92E-01	2.86E-03	3.89E-01	3.42E-01	undecaprenyl-phosphate galactose phosphotransferase [EC:2.7.8.6]
KO:K00997	0.88	1.88	2.32	1.87	0.01	0.00	1.61	5.60E-01	2.16E-01	1.46E-01	2.17E-01	9.59E-01	1.00E+00	2.79E-01	holo-[acyl-carrier protein] synthase [EC:2.7.8.7]
KO:K00998	7.53	3.55	0.83	6.59	0.59	1.98	7.56	1.09E-01	6.20E-01	9.56E-01	1.63E-01	9.67E-01	8.75E-01	1.08E-01	phosphatidylserine synthase [EC:2.7.8.8]
KO:K01000	8.10	14.41	4.50	5.12	4.50	2.29	3.40	2.31E-01	1.50E-02	8.54E-01	7.72E-01	8.54E-01	9.70E-01	9.32E-01	phospho-N-acetylmuramoyl-pentapeptide-transferase [EC:2.7.8.13]
KO:K01001	0.00	0.00	0.00	0.00	0.00	0.00	0.42	1.00E+00	1.00E+00	1.00E+00	1.00E+00	1.00E+00	1.00E+00	2.70E-02	UDP-N-acetylglucosamine--dolichyl-phosphate
KO:K01002	1.40	0.00	0.00	0.00	0.00	0.00	0.00	6.36E-02	1.00E+00	1.00E+00	1.00E+00	1.00E+00	1.00E+00	1.00E+00	phosphoglycerol transferase [EC:2.7.8.2]
KO:K01003	0.00	0.72	0.01	7.22	2.09	0.00	0.14	8.40E-01	4.83E-01	8.24E-01	3.23E-02	2.03E-01	1.00E+00	7.40E-01	carboxyvinyl-carboxyphosphonate phosphorylmutase [EC:2.7.8.23]
KO:K01004	0.19	25.14	0.00	0.12	0.00	0.00	0.00	7.93E-01	1.00E-03	1.00E+00	8.17E-01	1.00E+00	1.00E+00	1.00E+00	phosphatidylcholine synthase [EC:2.7.8.24]
KO:K01005	0.88	0.84	0.00	0.73	0.18	7.83	0.00	3.67E-01	3.82E-01	1.00E+00	4.15E-01	6.30E-01	2.73E-02	1.00E+00	
KO:K01006	1.06	12.73	17.94	2.82	2.60	0.00	2.08	9.18E-01	2.91E-02	8.14E-03	7.17E-01	7.59E-01	1.00E+00	8.27E-01	pyruvate,orthophosphate dikinase [EC:2.7.9.1]
KO:K01007	3.55	1.07	2.44	2.54	0.29	11.32	0.16	2.51E-01	7.05E-01	4.01E-01	3.85E-01	8.53E-01	2.31E-02	8.71E-01	pyruvate, water dikinase [EC:2.7.9.2]
KO:K01008	4.25	1.20	0.12	0.36	5.23	0.06	0.22	6.27E-02	4.46E-01	8.79E-01	7.87E-01	4.16E-02	9.17E-01	8.40E-01	selenide, water dikinase [EC:2.7.9.3]
KO:K01010	0.00	0.00	0.00	0.68	0.00	0.00	0.61	1.00E+00	1.00E+00	1.00E+00	1.43E-01	1.00E+00	1.00E+00	1.54E-01	thiosulfate sulfurtransferase [EC:2.8.1.1]
KO:K01011	2.08	12.33	0.02	3.81	0.00	3.83	0.70	6.55E-01	1.37E-02	9.83E-01	2.87E-01	1.00E+00	2.85E-01	8.99E-01	3-mercaptopyruvate sulfurtransferase [EC:2.8.1.2]
KO:K01012	1.28	0.17	2.31	0.85	11.99	0.24	4.97	5.71E-01	9.19E-01	2.66E-01	7.29E-01	5.57E-03	9.06E-01	6.11E-02	biotin synthetase [EC:2.8.1.6]
KO:K01013	0.00	0.00	0.00	0.10	0.00	0.00	0.00	1.00E+00	1.00E+00	1.00E+00	1.43E-01	1.00E+00	1.00E+00	1.00E+00	
KO:K01014	0.49	0.05	0.00	1.73	0.05	0.00	0.00	2.54E-01	4.10E-01	1.00E+00	1.07E-01	4.18E-01	1.00E+00	1.00E+00	aryl sulfotransferase [EC:2.8.2.1]
KO:K01015	0.00	0.89	0.00	0.00	0.00	0.00	0.00	1.00E+00	5.07E-02	1.00E+00	1.00E+00	1.00E+00	1.00E+00	1.00E+00	alcohol sulfotransferase [EC:2.8.2.2]
KO:K01023	0.00	1.00	0.00	0.00	0.00	0.00	0.00	1.00E+00	1.02E-01	1.00E+00	1.00E+00	1.00E+00	1.00E+00	1.00E+00	arylsulfate sulfotransferase [EC:2.8.2.22]
KO:K01025	0.00	0.00	0.00	2.76	0.00	0.00	0.00	1.00E+00	1.00E+00	1.00E+00	7.79E-02	1.00E+00	1.00E+00	1.00E+00	
KO:K01026	0.00	0.01	0.00	1.22	0.00	0.00	0.00	1.00E+00	4.15E-01	1.00E+00	4.84E-02	1.00E+00	1.00E+00	1.00E+00	propionate CoA-transferase [EC:2.8.3.1]
KO:K01028	1.36	20.16	0.01	1.38	0.00	0.20	0.01	6.57E-01	1.29E-03	9.72E-01	6.52E-01	1.00E+00	9.25E-01	9.72E-01	3-oxoacid CoA-transferase subunit A [EC:2.8.3.5]
KO:K01029	0.92	10.45	0.38	0.95	0.00	0.08	0.00	5.90E-01	8.29E-03	7.91E-01	5.74E-01	1.00E+00	8.92E-01	1.00E+00	3-oxoacid CoA-transferase subunit B [EC:2.8.3.5]
KO:K01031	0.35	1.27	0.00	0.00	0.00	0.00	0.00	2.04E-01	7.69E-02	1.00E+00	5.31E-01	1.00E+00	1.00E+00	1.00E+00	3-oxoadipate CoA-transferase, alpha subunit [EC:2.8.3.6]
KO:K01032	0.05	8.67	0.00	0.04	0.00	0.00	0.00	6.99E-01	1.10E-02	1.00E+00	7.03E-01	1.00E+00	1.00E+00	1.00E+00	3-oxoadipate CoA-transferase, beta subunit [EC:2.8.3.6]
KO:K01034	0.00	0.22	0.00	0.00	0.00	0.00	0.00	1.00E+00	9.74E-02	1.00E+00	1.00E+00	1.00E+00	1.00E+00	1.00E+00	acetate CoA-transferase alpha subunit [EC:2.8.3.8]
KO:K01035	0.00	0.00	0.00	0.20	0.00	0.00	0.00	1.00E+00	1.00E+00	1.00E+00	1.43E-01	1.00E+00	1.00E+00	1.00E+00	acetate CoA-transferase beta subunit [EC:2.8.3.8]
KO:K01036	0.00	0.11	0.00	0.00	0.00	0.00	0.00	1.00E+00	1.03E-01	1.00E+00	1.00E+00	1.00E+00	1.00E+00	1.00E+00	butyrate-acetoacetate CoA-transferase [EC:2.8.3.9]
KO:K01039	0.83	0.16	0.00	2.23	0.00	0.00	0.00	2.23E-01	4.70E-01	1.00E+00	8.33E-02	1.00E+00	1.00E+00	1.00E+00	glutaconate CoA-transferase, subunit A [EC:2.8.3.12]
KO:K01040	1.24	0.04	0.00	0.88	0.00	0.00	0.21	1.25E-01	5.68E-01	1.00E+00	1.63E-01	1.00E+00	1.00E+00	4.04E-01	glutaconate CoA-transferase, subunit B [EC:2.8.3.12]
KO:K01041	0.73	0.09	0.00	0.12	0.00	0.00	0.00	1.03E-01	2.87E-01	1.00E+00	2.61E-01	1.00E+00	1.00E+00	1.00E+00	
KO:K01042	1.15	0.69	0.00	1.67	0.00	0.00	0.06	2.06E-01	3.04E-01	1.00E+00	1.50E-01	1.00E+00	1.00E+00	6.54E-01	L-seryl-tRNA(Ser) seleniumtransferase [EC:2.9.1.1]
KO:K01043	1.41	1.63	0.31	0.68	1.36	14.19	0.03	4.30E-01	3.82E-01	7.39E-01	6.40E-01	4.43E-01	1.03E-02	8.39E-01	
KO:K01044	3.10	6.18	0.00	1.44	0.00	0.07	0.04	1.58E-01	4.74E-02	1.00E+00	4.01E-01	1.00E+00	8.51E-01	8.73E-01	carboxylesterase [EC:3.1.1.1]
KO:K01045	0.29	2.98	0.00	1.16	0.00	0.00	0.29	5.72E-01	6.94E-02	1.00E+00	2.20E-01	1.00E+00	1.00E+00	5.73E-01	arylesterase / paraoxonase [EC:3.1.1.2 3.1.8.1]
KO:K01046	0.46	0.04	3.86	0.09	4.94	0.00	0.45	4.12E-01	6.94E-01	6.13E-02	6.46E-01	3.87E-02	1.00E+00	4.14E-01	triacylglycerol lipase [EC:3.1.1.3]
KO:K01048	1.26	15.01	0.18	0.04	0.00	0.00	0.08	4.44E-01	2.43E-03	8.58E-01	9.15E-01	1.00E+00	1.00E+00	8.89E-01	lysophospholipase [EC:3.1.1.5]
KO:K01049	0.00	0.00	0.00	0.71	0.00	0.00	0.00	1.00E+00	1.00E+00	1.00E+00	1.43E-01	1.00E+00	1.00E+00	1.00E+00	acetylcholinesterase [EC:3.1.1.7]
KO:K01051	0.03	0.00	0.22	0.05	0.22	0.00	0.00	2.94E-01	1.00E+00	1.31E-01	2.68E-01	1.31E-01	1.00E+00	1.00E+00	pectinesterase [EC:3.1.1.11]
KO:K01053	0.64	0.36	10.92	4.33	2.13	0.00	1.32	7.56E-01	8.30E-01	2.29E-02	1.30E-01	3.75E-01	1.00E+00	5.63E-01	gluconolactonase [EC:3.1.1.17]
KO:K01054	4.91	0.00	0.00	0.13	0.00	0.00	0.00	4.06E-02	1.00E+00	1.00E+00	3.94E-01	1.00E+00	1.00E+00	1.00E+00	acylglycerol lipase [EC:3.1.1.23]
KO:K01055	0.10	20.19	0.00	2.72	0.00	0.00	0.00	8.86E-01	2.57E-03	1.00E+00	2.47E-01	1.00E+00	1.00E+00	1.00E+00	3-oxoadipate enol-lactonase [EC:3.1.1.24]
KO:K01056	3.84	5.02	6.23	3.93	6.44	4.60	1.99	6.23E-01	3.32E-01	1.52E-01	5.99E-01	1.37E-01	4.25E-01	9.20E-01	peptidyl-tRNA hydrolase, PTH1 family [EC:3.1.1.29]
KO:K01057	1.96	4.21	0.25	0.61	6.30	0.00	0.05	2.87E-01	5.77E-02	8.90E-01	7.84E-01	2.13E-02	1.00E+00	9.47E-01	6-phosphogluconolactonase [EC:3.1.1.31]
KO:K01058	0.23	0.00	0.00	0.00	0.00	0.00	0.00	5.89E-02	1.00E+00	1.00E+00	1.00E+00	1.00E+00	1.00E+00	1.00E+00	phospholipase A1 [EC:3.1.1.32]
KO:K01060	0.23	0.00	0.00	0.00	0.00	0.00	0.00	6.29E-02	1.00E+00	1.00E+00	1.00E+00	1.00E+00	1.00E+00	1.00E+00	cephalosporin-C deacetylase [EC:3.1.1.41]
KO:K01061	2.44	0.40	4.90	7.83	2.09	0.07	7.05	5.48E-01	9.09E-01	2.04E-01	7.89E-02	6.23E-01	9.59E-01	9.67E-02	carboxymethylenebutenolidase [EC:3.1.1.45]
KO:K01062	0.00	0.00	0.00	0.00	0.00	0.00	2.50	1.00E+00	1.00E+00	1.00E+00	1.00E+00	1.00E+00	1.00E+00	2.51E-02	1-alkyl-2-acetylglycerophosphocholine esterase [EC:3.1.1.47]
KO:K01066	3.52	0.60	4.00	3.00	0.00	0.00	0.02	1.25E-01	6.56E-01	1.00E-01	1.59E-01	1.00E+00	1.00E+00	8.90E-01	esterase / lipase [EC:3.1.1.-]
KO:K01067	0.00	0.00	0.00	0.00	0.00	1.67	0.00	1.00E+00	1.00E+00	1.00E+00	1.00E+00	1.00E+00	2.43E-03	1.00E+00	acetyl-CoA hydrolase [EC:3.1.2.1]
KO:K01068	0.92	0.00	0.00	0.24	0.00	0.00	0.00	9.51E-02	1.00E+00	1.00E+00	2.24E-01	1.00E+00	1.00E+00	1.00E+00	palmitoyl-CoA hydrolase [EC:3.1.2.2]
KO:K01069	7.15	3.63	1.40	5.12	2.69	0.00	4.64	9.09E-02	4.87E-01	9.02E-01	2.34E-01	7.06E-01	1.00E+00	2.95E-01	hydroxyacylglutathione hydrolase [EC:3.1.2.6]
KO:K01070	3.06	5.85	0.06	0.63	0.00	0.00	0.01	1.15E-01	3.94E-02	8.66E-01	6.07E-01	1.00E+00	1.00E+00	9.16E-01	S-formylglutathione hydrolase [EC:3.1.2.12]
KO:K01071	0.00	0.06	0.00	0.05	0.00	0.00	0.00	1.00E+00	1.46E-01	1.00E+00	1.89E-01	1.00E+00	1.00E+00	1.00E+00	oleoyl-[acyl-carrier-protein] hydrolase [EC:3.1.2.14]
KO:K01073	0.00	0.00	0.00	0.00	0.00	0.00	1.67	1.00E+00	1.00E+00	1.00E+00	1.00E+00	1.00E+00	1.00E+00	2.49E-02	acyl-CoA hydrolase [EC:3.1.2.2]
KO:K01075	0.60	1.74	1.09	0.16	0.86	0.86	0.00	4.24E-01	1.55E-01	2.56E-01	6.88E-01	3.24E-01	3.24E-01	1.00E+00	4-hydroxybenzoyl-CoA thioesterase [EC:3.1.2.23]
KO:K01076	3.05	3.45	0.29	1.16	0.01	4.03	0.00	1.60E-01	1.29E-01	8.40E-01	5.24E-01	9.53E-01	9.77E-02	1.00E+00	

KO:K01077	2.80	0.08	15.40	1.64	1.12	0.02	1.31	2.41E-01	9.06E-01	7.43E-03	4.74E-01	6.18E-01	9.27E-01	5.63E-01	alkaline phosphatase [EC:3.1.3.1]
KO:K01078	0.00	0.00	0.00	0.50	2.10	0.00	0.00	1.00E+00	1.00E+00	1.00E+00	1.51E-01	3.57E-02	1.00E+00	1.00E+00	acid phosphatase [EC:3.1.3.2]
KO:K01079	1.66	6.90	0.01	0.88	0.00	3.58	1.10	4.46E-01	3.24E-02	9.68E-01	7.17E-01	1.00E+00	1.25E-01	6.36E-01	phosphoserine phosphatase [EC:3.1.3.3]
KO:K01081	9.60	6.66	0.66	1.34	0.00	0.38	4.64	4.47E-02	1.02E-01	9.05E-01	8.23E-01	1.00E+00	9.31E-01	2.35E-01	5'-nucleotidase [EC:3.1.3.5]
KO:K01082	0.00	0.00	1.84	0.16	0.30	0.00	0.00	1.00E+00	1.00E+00	2.39E-02	2.33E-01	1.69E-01	1.00E+00	1.00E+00	3'(2'), 5'-bisphosphate nucleotidase [EC:3.1.3.7]
KO:K01083	0.55	0.00	0.00	0.54	0.00	0.00	0.78	1.73E-01	1.00E+00	1.00E+00	1.75E-01	1.00E+00	1.00E+00	1.35E-01	3-phytase [EC:3.1.3.8]
KO:K01087	0.00	0.01	0.00	0.00	0.00	6.25	0.00	4.10E-01	3.71E-01	1.00E+00	4.11E-01	1.00E+00	7.14E-04	1.00E+00	trehalose-phosphatase [EC:3.1.3.12]
KO:K01089	1.59	0.49	0.00	0.56	0.00	1.59	0.00	1.21E-01	3.69E-01	1.00E+00	3.38E-01	1.00E+00	1.21E-01	1.00E+00	imidazoleglycerol-phosphate dehydratase / histidinol-phosphatase
KO:K01090	0.42	0.24	10.79	0.61	0.30	0.00	0.54	6.10E-01	7.15E-01	5.00E-03	4.99E-01	6.78E-01	1.00E+00	5.44E-01	protein phosphatase [EC:3.1.3.16]
KO:K01091	2.23	11.45	4.63	4.62	2.44	0.06	2.10	8.22E-01	2.06E-02	3.28E-01	3.29E-01	7.90E-01	9.96E-01	8.41E-01	phosphoglycolate phosphatase [EC:3.1.3.18]
KO:K01092	4.99	16.57	5.64	4.21	9.68	0.55	2.06	6.80E-01	1.30E-02	5.58E-01	8.01E-01	1.04E-01	9.93E-01	9.52E-01	myo-inositol-1(or 4)-monophosphatase [EC:3.1.3.25]
KO:K01095	5.36	3.19	2.81	0.98	0.00	0.18	0.41	5.14E-02	1.57E-01	2.06E-01	7.27E-01	1.00E+00	9.25E-01	8.79E-01	phosphatidylglycerophosphatase A [EC:3.1.3.27]
KO:K01101	0.35	0.00	0.00	0.00	0.00	0.00	0.00	6.16E-02	1.00E+00	1.00E+00	1.00E+00	1.00E+00	1.00E+00	1.00E+00	4-nitrophenyl phosphatase [EC:3.1.3.41]
KO:K01104	2.76	0.18	11.15	0.37	5.06	0.44	0.62	1.48E-01	9.29E-01	4.00E-03	8.82E-01	4.13E-02	8.61E-01	8.05E-01	protein-tyrosine phosphatase [EC:3.1.3.48]
KO:K01112	1.51	12.92	0.50	0.89	0.00	0.06	0.00	4.55E-01	1.43E-02	7.51E-01	6.33E-01	1.00E+00	8.88E-01	1.00E+00	
KO:K01113	1.74	0.04	4.00	1.13	0.00	0.00	2.16	2.19E-01	7.98E-01	7.10E-02	3.28E-01	1.00E+00	1.00E+00	1.75E-01	alkaline phosphatase D [EC:3.1.3.1]
KO:K01114	0.00	0.00	0.00	1.12	0.00	0.00	0.00	1.00E+00	1.00E+00	1.00E+00	6.01E-02	1.00E+00	1.00E+00	1.00E+00	phospholipase C [EC:3.1.4.3]
KO:K01115	0.23	0.00	0.00	0.00	0.00	0.00	0.00	6.51E-02	1.00E+00	1.00E+00	1.00E+00	1.00E+00	1.00E+00	1.00E+00	phospholipase D [EC:3.1.4.4]
KO:K01118	0.86	3.73	0.60	6.10	0.00	0.00	0.02	6.37E-01	1.14E-01	7.30E-01	4.99E-02	1.00E+00	1.00E+00	9.22E-01	FMN-dependent NADH-azoreductase [EC:1.7.-.-]
KO:K01119	0.00	0.00	0.54	0.01	0.00	0.00	0.00	1.00E+00	1.00E+00	1.53E-02	2.53E-01	1.00E+00	1.00E+00	1.00E+00	2',3'-cyclic-nucleotide 2'-phosphodiesterase [EC:3.1.4.16]
KO:K01120	0.00	0.00	3.42	0.27	0.00	0.00	0.00	1.00E+00	1.00E+00	2.16E-02	2.02E-01	1.00E+00	1.00E+00	1.00E+00	3',5'-cyclic-nucleotide phosphodiesterase [EC:3.1.4.17]
KO:K01122	0.12	0.00	0.00	0.00	0.00	0.00	0.00	6.66E-02	1.00E+00	1.00E+00	1.00E+00	1.00E+00	1.00E+00	1.00E+00	ectonucleotide pyrophosphatase/phosphodiesterase family member 2
KO:K01126	2.90	2.74	0.68	2.64	0.45	0.64	0.90	2.14E-01	2.41E-01	8.11E-01	2.56E-01	8.62E-01	8.20E-01	7.54E-01	glycerophosphoryl diester phosphodiesterase [EC:3.1.4.46]
KO:K01127	0.00	0.00	0.00	0.82	0.00	0.00	0.00	1.00E+00	1.00E+00	1.00E+00	1.43E-01	1.00E+00	1.00E+00	1.00E+00	glycosylphosphatidylinositol phospholipase D [EC:3.1.4.5]
KO:K01128	0.04	28.03	0.00	0.00	0.00	0.00	0.00	8.69E-01	5.71E-04	1.00E+00	8.95E-01	1.00E+00	1.00E+00	1.00E+00	
KO:K01129	2.13	4.66	0.94	2.16	0.82	1.91	1.57	4.95E-01	9.83E-02	8.37E-01	4.84E-01	8.64E-01	5.57E-01	6.72E-01	dGTPase [EC:3.1.5.1]
KO:K01130	1.95	0.43	14.47	0.16	6.36	0.00	6.28	4.76E-01	7.91E-01	1.97E-02	8.53E-01	1.11E-01	1.00E+00	1.14E-01	arylsulfatase [EC:3.1.6.1]
KO:K01132	0.00	0.00	3.62	0.14	1.53	0.00	2.89	1.00E+00	1.00E+00	7.09E-02	3.91E-01	1.44E-01	1.00E+00	8.77E-02	N-acetylgalactosamine-6-sulfatase [EC:3.1.6.4]
KO:K01133	0.21	9.06	12.96	0.25	0.00	0.00	2.25	8.97E-01	3.47E-02	1.39E-02	8.83E-01	1.00E+00	1.00E+00	3.68E-01	choline-sulfatase [EC:3.1.6.6]
KO:K01134	0.05	0.02	22.72	0.29	1.05	0.00	6.04	7.16E-01	7.36E-01	7.14E-03	5.81E-01	3.93E-01	1.00E+00	8.71E-02	arylsulfatase A [EC:3.1.6.8]
KO:K01135	0.00	0.07	20.49	0.03	0.69	0.00	1.86	1.00E+00	5.53E-01	5.14E-03	5.80E-01	3.48E-01	1.00E+00	1.84E-01	arylsulfatase B [EC:3.1.6.12]
KO:K01136	0.11	0.02	14.74	0.15	1.99	0.00	1.46	5.77E-01	6.56E-01	1.44E-02	5.57E-01	1.92E-01	1.00E+00	2.43E-01	iduronate 2-sulfatase [EC:3.1.6.13]
KO:K01137	0.19	0.01	8.09	0.06	2.68	0.00	5.33	5.53E-01	6.58E-01	3.47E-02	6.24E-01	1.59E-01	1.00E+00	6.36E-02	N-acetylglucosamine-6-sulfatase [EC:3.1.6.14]
KO:K01138	1.39	0.14	23.14	0.51	2.91	0.00	5.16	5.56E-01	8.34E-01	7.43E-03	7.45E-01	3.32E-01	1.00E+00	1.74E-01	
KO:K01139	4.52	3.46	0.30	1.03	7.87	0.13	0.17	1.43E-01	2.29E-01	8.71E-01	7.19E-01	5.11E-02	8.98E-01	8.91E-01	guanosine-3',5'-bis(diphosphate) 3'-pyrophosphohydrolase
KO:K01141	3.53	0.83	0.03	3.70	0.00	0.00	4.52	1.35E-01	6.65E-01	9.11E-01	1.25E-01	1.00E+00	1.00E+00	8.87E-02	exodeoxyribonuclease I [EC:3.1.11.1]
KO:K01142	7.60	12.90	0.67	5.70	1.01	1.48	1.27	1.10E-01	1.90E-02	9.85E-01	2.86E-01	9.73E-01	9.49E-01	9.62E-01	exodeoxyribonuclease III [EC:3.1.11.2]
KO:K01144	0.27	11.82	0.00	0.32	3.48	0.06	0.00	8.74E-01	3.57E-03	1.00E+00	8.62E-01	7.97E-02	9.35E-01	9.66E-01	exodeoxyribonuclease V [EC:3.1.11.5]
KO:K01146	2.98	0.00	0.00	0.18	0.00	0.00	0.00	3.33E-02	1.00E+00	1.00E+00	3.44E-01	1.00E+00	1.00E+00	1.00E+00	
KO:K01147	0.48	0.00	0.00	0.12	21.75	0.00	0.02	3.80E-01	7.65E-01	1.00E+00	5.81E-01	4.29E-04	1.00E+00	7.12E-01	exoribonuclease II [EC:3.1.13.1]
KO:K01150	0.00	0.00	5.33	0.01	0.00	0.00	0.00	1.00E+00	5.04E-01	4.00E-03	4.39E-01	1.00E+00	1.00E+00	1.00E+00	deoxyribonuclease I [EC:3.1.21.1]
KO:K01151	0.03	0.00	0.05	0.19	0.00	0.36	1.82	4.35E-01	1.00E+00	4.01E-01	2.63E-01	1.00E+00	2.01E-01	5.61E-02	deoxyribonuclease IV [EC:3.1.21.2]
KO:K01153	1.15	0.38	1.26	0.56	0.60	7.16	0.48	3.69E-01	7.88E-01	3.31E-01	6.89E-01	6.64E-01	9.00E-03	7.39E-01	type I restriction enzyme, R subunit [EC:3.1.21.3]
KO:K01154	0.26	0.23	1.12	0.18	0.50	0.28	0.14	5.19E-01	5.79E-01	7.24E-02	6.57E-01	2.63E-01	4.91E-01	7.22E-01	type I restriction enzyme, S subunit [EC:3.1.21.3]
KO:K01155	0.00	0.15	0.00	0.03	0.00	0.00	0.00	1.00E+00	1.14E-01	1.00E+00	2.17E-01	1.00E+00	1.00E+00	1.00E+00	type II restriction enzyme [EC:3.1.21.4]
KO:K01156	1.03	1.18	0.00	0.31	0.00	7.11	0.09	3.04E-01	2.68E-01	1.00E+00	5.99E-01	1.00E+00	2.01E-02	7.49E-01	type III restriction enzyme [EC:3.1.21.5]
KO:K01157	0.00	0.00	0.70	1.97	8.93	0.00	0.00	1.00E+00	1.00E+00	3.06E-01	1.45E-01	1.87E-02	1.00E+00	1.00E+00	
KO:K01159	4.73	3.70	5.08	2.27	4.63	2.69	0.30	1.85E-01	3.48E-01	1.55E-01	7.06E-01	1.96E-01	5.97E-01	9.75E-01	crossover junction endodeoxyribonuclease RuvC [EC:3.1.22.4]
KO:K01162	0.00	0.00	0.00	0.20	0.00	0.00	0.00	1.00E+00	1.00E+00	1.00E+00	1.43E-01	1.00E+00	1.00E+00	1.00E+00	ribonuclease III [EC:3.1.26.3]
KO:K01166	0.23	0.00	0.00	0.00	0.00	0.00	0.00	6.44E-02	1.00E+00	1.00E+00	1.00E+00	1.00E+00	1.00E+00	1.00E+00	ribonuclease T2 [EC:3.1.27.1]
KO:K01170	0.00	0.00	0.00	0.00	0.00	0.00	2.50	1.00E+00	1.00E+00	1.00E+00	1.00E+00	1.00E+00	1.00E+00	2.70E-02	tRNA-intron endonuclease [EC:3.1.27.9]
KO:K01172	0.00	5.56	0.00	0.00	0.00	0.00	0.00	1.00E+00	7.29E-03	1.00E+00	1.00E+00	1.00E+00	1.00E+00	1.00E+00	
KO:K01173	0.60	1.07	0.00	0.08	0.00	10.02	0.00	3.70E-01	2.50E-01	1.00E+00	6.25E-01	1.00E+00	1.11E-02	1.00E+00	endonuclease [EC:3.1.3.-]
KO:K01174	0.08	0.00	0.00	0.26	0.00	0.00	0.00	1.99E-01	1.00E+00	1.00E+00	1.12E-01	1.00E+00	1.00E+00	1.00E+00	micrococcal nuclease [EC:3.1.31.1]
KO:K01175	6.60	3.20	12.13	2.62	0.58	1.44	0.63	8.99E-02	5.01E-01	1.47E-02	6.46E-01	9.55E-01	8.66E-01	9.52E-01	
KO:K01176	0.65	0.23	0.00	0.12	4.57	0.00	0.32	2.85E-01	5.01E-01	1.00E+00	5.92E-01	2.66E-02	1.00E+00	4.43E-01	alpha-amylase [EC:3.2.1.1]
KO:K01178	0.06	0.00	0.00	0.05	0.00	0.00	0.00	1.05E-01	1.00E+00	1.00E+00	2.09E-01	1.00E+00	1.00E+00	1.00E+00	glucoamylase [EC:3.2.1.3]
KO:K01179	0.03	0.36	14.76	0.89	0.00	0.00	0.61	8.20E-01	6.01E-01	3.00E-03	3.32E-01	1.00E+00	1.00E+00	4.58E-01	endoglucanase [EC:3.2.1.4]
KO:K01181	0.18	0.04	6.36	0.03	1.00	0.00	0.03	4.11E-01	5.72E-01	1.54E-02	5.85E-01	1.68E-01	1.00E+00	5.99E-01	endo-1,4-beta-xylanase [EC:3.2.1.8]
KO:K01182	0.31	0.00	0.00	0.08	0.00	6.19	0.00	2.43E-01	5.73E-01	1.00E+00	4.14E-01	1.00E+00	5.43E-03	1.00E+00	oligo-1,6-glucosidase [EC:3.2.1.1]
KO:K01183	0.35	0.00	0.22	0.29	5.47	0.00	0.00	3.05E-01	1.00E+00	3.67E-01	3.32E-01	2.47E-02	1.00E+00	1.00E+00	chitinase [EC:3.2.1.14]
KO:K01185	0.00	0.00	0.00	0.04	14.24	0.00	0.00	5.99E-01	1.00E+00	1.00E+00	4.80E-01	0.00E+00	1.00E+00	1.00E+00	lysozyme [EC:3.2.1.17]
KO:K01186	0.11	0.00	11.02	0.56	0.05	0.00	0.36	5.14E-01	1.00E+00	1.37E-02	3.23E-01	5.66E-01	1.00E+00	3.80E-01	sialidase-1 [EC:3.2.1.18]
KO:K01187	2.40	5.68	0.51	1.28	0.00	2.52	0.14	2.81E-01	4.67E-02	8.27E-01	5.92E-01	1.00E+00	2.57E-01	9.15E-01	alpha-glucosidase [EC:3.2.1.2]
KO:K01188	20.70	0.04	0.00	2.60	0.00	0.00	0.00	4.43E-03	8.44E-01	1.00E+00	2.76E-01	1.00E+00	1.00E+00	8.67E-01	beta-glucosidase [EC:3.2.1.21]
KO:K01190	0.25	0.03	8.09	0.03	0.00	0.00	0.25	3.83E-01	6.47E-01	3.71E-03	6.34E-01	1.00E+00	1.00E+00	3.85E-01	beta-galactosidase [EC:3.2.1.23]
KO:K01191	0.00	0.03	0.00	0.00	26.64	0.00	0.00	1.00E+00	5.36E-01	1.00E+00	1.00E+00	1.43E-04	1.00E+00	1.00E+00	alpha-mannosidase [EC:3.2.1.24]
KO:K01192	0.08	0.00	0.35	1.16	0.00	0.00	0.00	3.50E-01	5.34E-01	2.04E-01	7.93E-02	1.00E+00	1.00E+00	1.00E+00	beta-mannosidase [EC:3.2.1.25]
KO:K01193	0.08	0.14	0.00	0.00	19.77	0.00	1.16	5.80E-01	5.34E-01	1.00E+00	1.00E+00	1.43E-03	1.00E+00	2.05E-01	beta-fructofuranosidase [EC:3.2.1.26]
KO:K01194	0.87	0.38	0.00	0.05	0.00	0.00	0.00	8.99E-02	1.84E-01	1.00E+00	4.49E-01	1.00E+00	1.00E+00	1.00E+00	alpha,alpha-trehalase [EC:3.2.1.28]
KO:K01195	0.71	0.13	2.67	0.01	0.00	0.00	0.23	1.90E-01	4.16E-01	4.91E-02	6.12E-01	1.00E+00	1.00E+00	3.25E-01	beta-glucuronidase [EC:3.2.1.31]
KO:K01198	0.06	0.06	5.40	0.17	0.00	0.00	0.00	5.04E-01	5.14E-01	8.00E-03	3.66E-01	1.00E+00	1.00E+00	1.00E+00	xylan 1,4-beta-xylosidase [EC:3.2.1.37]
KO:K01199	4.92	0.15	0.00	0.94	0.00	0.00	0.00	6.34E-02	5.35E-01	1.00E+00	2.81E-01	1.00E+00	1.00E+00	1.00E+00	glucan endo-1,3-beta-D-glucosidase [EC:3.2.1.39]
KO:K01200	2.45	0.00	0.00	0.05	0.00	0.00	0.00	4.29E-02	1.00E+00	1.00E+00	3.85E-01	1.00E+00	1.00E+00	1.00E+00	pullulanase [EC:3.2.1.41]
KO:K01201	0.68	0.11	0.05	0.12	0.00	16.26	0.00	2.91E-01	5.39E-01	6.00E-01	5.26E-01	1.00E+00	4.43E-03	1.00E+00	glucosylceramidase [EC:3.2.1.45]
KO:K01205	0.23	0.00	0.00	0.00	0.00	0.00	0.00	6.51E-02	1.00E+00	1.00E+00	1.00E+00	1.00E+00	1.00E+00	1.00E+00	alpha-N-acetylglucosaminidase [EC:3.2.1.5]
KO:K01206	0.26	0.21	1.71	1.26	0.19	0.00	0.04	4.13E-01	4.43E-01	1.35E-01	1.71E-01	4.62E-01	1.00E+00	6.27E-01	alpha-L-fucosidase [EC:3.2.1.51]
KO:K01207	1.62	2.69	0.09	3.15	0.00	3.19	2.37	5.05E-01	2.12E-01	9.69E-01	1.51E-01	1.00E+00	1.47E-01	2.74E-01	beta-N-acetylhexosaminidase [EC:3.2.1.52]
KO:K01209	1.98	0.66	1.39	0.63	0.00	0.00	0.01	1.44E-01	3.67E-01	2.05E-01	3.77E-01	1.00E+00	1.00E+00	7.48E-01	alpha-N-arabinofuranosidase [EC:3.2.1.55]
KO:K01212	0.81	0.01	0.02	0.54	0.00	0.00	5.70	2.63E-01	7.01E-01	6.70E-01	3.32E-01	1.00E+00	1.00E+00	2.73E-02	levanase [EC:3.2.1.65]
KO:K01213	0.00	0.00	0.00	0.00	0.00	0.00	0.42	1.00E+00	1.00E+00	1.00E+00	1.00E+00	1.00E+00	1.00E+00	2.50E-02	galacturan 1,4-alpha-galacturonidase [EC:3.2.1.67]
KO:K01216	5.16	0.12	0.00	0.00	0.00	0.00	0.00	3.96E-02	3.85E-01	1.00E+00	1.00E+00	1.00E+00	1.00E+00	1.00E+00	licheninase [EC:3.2.1.73]
KO:K01218	0.00	0.00	0.00	0.10	0.00	0.00	0.00	1.00E+00	1.00E+00	1.00E+00	1.43E-01	1.00E+00	1.00E+00	1.00E+00	mannan endo-1,4-beta-mannosidase [EC:3.2.1.78]
KO:K01219	0.08	0.00	5.22	0.00	0.00	0.00	0.02	3.14E-01	1.00E+00	9.57E-03	1.00E+00	1.00E+00	1.00E+00	3.61E-01	agarase [EC:3.2.1.81]
KO:K01224	1.86	0.00	0.00	0.00	0.00	0.00	0.00	2.80E-02	1.00E+00	1.00E+00	1.00E+00	1.00E+00	1.00E+00	1.00E+00	arabinogalactan endo-1,4-beta-galactosidase [EC:3.2.1.89]
KO:K01234	0.10	0.13	0.00	0.04	11.92	0.00	0.00	4.92E-01	4.71E-01	1.00E+00	5.42E-01	4.14E-03	1.00E+00	1.00E+00	neopullulanase [EC:3.2.1.135]
KO:K01235	0.06	0.00	0.74	0.00	0.00	0.00	0.08	2.69E-01	1.00E+00	3.67E-02	4.52E-01	1.00E+00	1.00E+00	2.25E-01	alpha-glucuronidase [EC:3.2.1.139]
KO:K01236	0.23	0.00	0.00	0.00	0.00	0.00	0.00	5.91E-02	1.00E+00	1.00E+00	1.00E+00	1.00E+00	1.00E+00	1.00E+00	maltooligosyltrehalose trehalohydrolase [EC:3.2.1.141]
KO:K01238	2.02	1.07	0.01	1.33	0.00	16.64	0.00	3.00E-01	4.87E-01	8.00E-01	4.28E-01	1.00E+00	1.01E-02	1.00E+00	
KO:K01239	0.31	14.89	0.80	0.32	0.16	0.00	0.00	8.24E-01	1.57E-03	6.55E-01	8.18E-01	8.72E-01	1.00E+00	1.00E+00	purine nucleosidase [EC:3.2.2.1]
KO:K01241	0.43	4.76	4.77	0.31	0.00	0.00	0.12	6.58E-01	4.59E-02	4.57E-02	7.12E-01	1.00E+00	1.00E+00	8.16E-01	AMP nucleosidase [EC:3.2.2.4]
KO:K01243	0.02	0.04	0.00	4.58	1.60	0.00	0.04	6.33E-01	6.07E-01	1.00E+00	5.86E-02	1.74E-01	1.00E+00	6.02E-01	S-adenosylhomocysteine/5'-methylthioadenosine nucleosidase
KO:K01244	0.12	0.00	0.00	0.00	0.00	0.00	0.00	6.47E-02	1.00E+00	1.00E+00	1.00E+00	1.00E+00	1.00E+00	1.00E+00	5'-methylthioadenosine nucleosidase [EC:3.2.2.16]
KO:K01246	1.46	0.91	1.89	1.66	0.00	0.00	0.03	1.70E-01	3.47E-01	1.11E-01	1.40E-01	1.00E+00	1.00E+00	9.36E-01	DNA-3-methyladenine glycosylase I [EC:3.2.2.2]
KO:K01247	2.01	3.08	0.01	1.36	0.06	0.00	0.31	1.95E-01	1.04E-01	9.11E-01	3.10E-01	8.73E-01	1.00E+00	7.06E-01	DNA-3-methyladenine glycosylase II [EC:3.2.2.21]
KO:K01249	0.13	0.28	0.00	1.13	2.52	0.00	0.00	5.64E-01	4.25E-01	1.00E+00	1.53E-01	5.54E-02	1.00E+00	1.00E+00	DNA glycosylase [EC:3.2.2.-]

KO:K01250	0.00	0.00	0.00	0.20	0.00	0.00	0.00	1.00E+00	1.00E+00	1.00E+00	7.63E-02	1.00E+00	1.00E+00	1.00E+00	pyrimidine-specific ribonucleoside hydrolase [EC:3.2.-.-]
KO:K01251	7.97	10.32	12.32	8.78	7.88	14.02	9.77	8.02E-01	3.76E-01	1.35E-01	6.77E-01	8.10E-01	5.83E-02	4.91E-01	adenosylhomocysteinase [EC:3.3.1.1]
KO:K01253	0.50	0.73	0.00	4.08	0.00	0.00	0.00	4.11E-01	3.35E-01	1.00E+00	5.93E-02	1.00E+00	1.00E+00	1.00E+00	microsomal epoxide hydrolase [EC:3.3.2.9]
KO:K01254	0.81	0.00	0.00	0.00	0.00	0.00	0.00	6.34E-02	1.00E+00	1.00E+00	1.00E+00	1.00E+00	1.00E+00	1.00E+00	leukotriene-A4 hydrolase [EC:3.3.2.6]
KO:K01255	8.18	11.40	2.26	8.57	1.69	1.66	7.75	2.28E-01	6.67E-02	9.58E-01	1.91E-01	9.75E-01	9.77E-01	2.79E-01	leucyl aminopeptidase [EC:3.4.11.1]
KO:K01256	8.94	4.60	1.92	1.93	7.68	3.42	0.64	4.34E-02	3.27E-01	8.75E-01	8.74E-01	7.23E-02	6.06E-01	9.73E-01	aminopeptidase N [EC:3.4.11.2]
KO:K01258	0.11	0.59	0.52	0.83	0.52	0.00	0.00	6.43E-01	2.56E-01	2.92E-01	1.73E-01	2.92E-01	1.00E+00	1.00E+00	tripeptide aminopeptidase [EC:3.4.11.4]
KO:K01259	7.95	15.94	0.00	2.73	0.00	0.03	0.49	6.60E-02	7.43E-03	9.84E-01	5.81E-01	1.00E+00	9.76E-01	9.34E-01	proline iminopeptidase [EC:3.4.11.5]
KO:K01262	11.09	4.62	7.97	5.48	4.46	1.91	1.13	3.47E-02	6.39E-01	1.18E-01	4.45E-01	6.73E-01	9.59E-01	9.85E-01	X-Pro aminopeptidase [EC:3.4.11.9]
KO:K01263	0.58	0.00	0.00	0.00	0.00	0.00	0.00	3.87E-02	1.00E+00	1.00E+00	1.00E+00	1.00E+00	1.00E+00	1.00E+00	cytosol alanyl aminopeptidase [EC:3.4.11.14]
KO:K01265	7.99	8.38	5.91	4.67	8.76	2.27	2.84	1.88E-01	1.58E-01	5.08E-01	7.53E-01	1.30E-01	9.58E-01	9.32E-01	methionyl aminopeptidase [EC:3.4.11.18]
KO:K01266	0.01	13.15	0.00	0.23	0.73	0.00	0.00	8.94E-01	3.14E-03	1.00E+00	7.83E-01	5.09E-01	1.00E+00	1.00E+00	D-aminopeptidase [EC:3.4.11.19]
KO:K01267	13.59	0.32	0.00	0.11	0.00	0.11	0.00	1.49E-02	5.90E-01	1.00E+00	6.64E-01	1.00E+00	6.63E-01	1.00E+00	aspartyl aminopeptidase [EC:3.4.11.21]
KO:K01268	0.00	0.00	0.00	0.00	0.00	0.00	2.08	1.00E+00	1.00E+00	1.00E+00	1.00E+00	1.00E+00	1.00E+00	3.03E-02	aminopeptidase I [EC:3.4.11.22]
KO:K01269	4.77	1.91	18.67	2.26	1.64	1.21	0.24	2.33E-01	6.93E-01	7.43E-03	6.23E-01	7.46E-01	8.18E-01	9.31E-01	aminopeptidase [EC:3.4.11.-]
KO:K01270	1.32	1.06	0.00	0.27	0.00	7.42	0.01	2.27E-01	2.96E-01	1.00E+00	6.31E-01	1.00E+00	1.16E-02	7.99E-01	aminoacylhistidine dipeptidase [EC:3.4.13.3]
KO:K01271	1.21	9.91	1.35	6.26	0.00	0.00	0.17	7.15E-01	3.61E-02	6.83E-01	9.93E-02	1.00E+00	1.00E+00	9.07E-01	X-Pro dipeptidase [EC:3.4.13.9]
KO:K01273	4.58	7.58	0.77	1.69	0.05	0.00	0.57	1.37E-01	5.59E-02	7.84E-01	5.51E-01	9.30E-01	1.00E+00	8.28E-01	membrane dipeptidase [EC:3.4.13.19]
KO:K01274	0.28	0.87	0.00	1.94	0.00	0.00	0.00	3.54E-01	2.23E-01	1.00E+00	1.32E-01	1.00E+00	1.00E+00	1.00E+00	D-alanyl-D-alanine dipeptidase [EC:3.4.13.-]
KO:K01277	0.29	1.34	2.54	1.04	0.00	0.00	0.00	4.74E-01	2.42E-01	1.29E-01	2.85E-01	1.00E+00	1.00E+00	1.00E+00	dipeptidyl-peptidase III [EC:3.4.14.4]
KO:K01278	2.45	0.82	0.71	2.15	0.00	2.61	0.00	1.82E-01	5.42E-01	5.91E-01	2.18E-01	1.00E+00	1.65E-01	8.89E-01	dipeptidyl-peptidase 4 [EC:3.4.14.5]
KO:K01280	0.00	0.00	2.50	0.00	0.00	0.00	0.00	1.00E+00	1.00E+00	1.63E-02	1.00E+00	1.00E+00	1.00E+00	1.00E+00	tripeptidyl-peptidase II [EC:3.4.14.1]
KO:K01281	0.24	0.44	3.48	0.11	0.00	0.00	0.00	3.82E-01	2.87E-01	3.49E-02	4.63E-01	1.00E+00	1.00E+00	1.00E+00	X-Pro dipeptidyl-peptidase [EC:3.4.14.11]
KO:K01283	6.16	0.00	0.00	2.11	0.00	0.00	0.00	4.37E-02	1.00E+00	1.00E+00	1.76E-01	1.00E+00	1.00E+00	1.00E+00	peptidyl-dipeptidase A [EC:3.4.15.1]
KO:K01284	2.22	16.97	1.78	0.60	0.00	0.35	0.00	4.60E-01	3.00E-03	5.83E-01	8.64E-01	1.00E+00	9.04E-01	1.00E+00	peptidyl-dipeptidase Dcp [EC:3.4.15.5]
KO:K01286	0.62	21.57	0.06	1.98	0.00	0.05	0.00	8.19E-01	2.57E-03	9.30E-01	4.63E-01	1.00E+00	9.33E-01	1.00E+00	D-alanyl-D-alanine carboxypeptidase [EC:3.4.16.4]
KO:K01289	0.80	0.95	0.17	1.20	0.00	1.20	0.01	3.41E-01	3.06E-01	6.05E-01	2.54E-01	1.00E+00	2.54E-01	7.17E-01	carboxypeptidase [EC:3.4.16.-]
KO:K01293	0.78	1.19	0.00	0.07	0.00	0.00	0.00	1.78E-01	1.30E-01	1.00E+00	3.66E-01	1.00E+00	1.00E+00	1.00E+00	Gly-Xaa carboxypeptidase [EC:3.4.17.4]
KO:K01295	2.83	0.93	0.02	3.00	0.06	0.00	0.00	1.31E-01	4.06E-01	8.10E-01	1.22E-01	7.64E-01	1.00E+00	1.00E+00	glutamate carboxypeptidase [EC:3.4.17.11]
KO:K01297	1.30	0.00	0.00	0.04	11.50	0.00	0.00	1.45E-01	7.61E-01	1.00E+00	6.79E-01	4.29E-03	1.00E+00	1.00E+00	muramoyltetrapeptide carboxypeptidase [EC:3.4.17.13]
KO:K01299	0.16	14.30	2.96	0.50	3.72	0.00	0.25	9.38E-01	2.86E-03	2.48E-01	8.86E-01	1.45E-01	1.00E+00	9.26E-01	carboxypeptidase Taq [EC:3.4.17.19]
KO:K01301	0.06	0.25	0.00	0.72	0.00	7.23	0.00	4.63E-01	3.74E-01	1.00E+00	2.39E-01	1.00E+00	2.37E-02	1.00E+00	glutamate carboxypeptidase II [EC:3.4.17.21]
KO:K01302	0.24	0.46	0.00	1.13	13.77	0.93	0.00	6.86E-01	5.80E-01	1.00E+00	3.48E-01	7.29E-03	4.01E-01	1.00E+00	
KO:K01303	1.77	0.13	0.00	2.14	0.00	2.01	0.00	2.09E-01	5.71E-01	1.00E+00	1.73E-01	1.00E+00	1.85E-01	1.00E+00	acylaminoacyl-peptidase [EC:3.4.19.1]
KO:K01304	0.00	0.00	0.63	0.00	0.00	0.00	0.00	1.00E+00	1.00E+00	1.56E-02	1.00E+00	1.00E+00	1.00E+00	1.00E+00	pyroglutamyl-peptidase [EC:3.4.19.3]
KO:K01305	0.26	0.00	0.37	0.02	0.00	0.65	0.00	1.75E-01	1.00E+00	1.42E-01	3.92E-01	1.00E+00	8.20E-02	1.00E+00	beta-aspartyl-dipeptidase (metallo-type) [EC:3.4.19.-]
KO:K01322	2.31	0.51	3.86	2.00	3.67	3.53	0.34	3.66E-01	8.24E-01	1.62E-01	4.35E-01	1.80E-01	1.95E-01	8.59E-01	prolyl oligopeptidase [EC:3.4.21.26]
KO:K01338	14.99	14.66	5.92	11.94	4.03	8.74	1.84	4.64E-02	5.37E-02	9.31E-01	1.78E-01	9.74E-01	6.72E-01	9.94E-01	ATP-dependent Lon protease [EC:3.4.21.53]
KO:K01341	0.00	0.11	0.00	0.00	0.00	0.00	0.00	1.00E+00	1.01E-01	1.00E+00	1.00E+00	1.00E+00	1.00E+00	1.00E+00	kexin [EC:3.4.21.61]
KO:K01342	1.70	0.03	0.00	0.11	0.00	0.00	0.00	7.83E-02	3.97E-01	1.00E+00	3.29E-01	1.00E+00	1.00E+00	1.00E+00	subtilisin [EC:3.4.21.62]
KO:K01354	1.76	1.34	0.00	5.92	0.00	5.24	0.00	3.76E-01	4.94E-01	1.00E+00	5.41E-02	1.00E+00	6.86E-02	1.00E+00	oligopeptidase B [EC:3.4.21.83]
KO:K01356	6.26	5.07	1.54	3.35	2.79	2.50	0.25	1.18E-01	2.03E-01	8.34E-01	4.65E-01	5.90E-01	6.62E-01	9.64E-01	repressor LexA [EC:3.4.21.88]
KO:K01358	8.32	3.66	13.65	6.22	14.27	5.19	4.31	3.30E-01	9.39E-01	3.19E-02	7.09E-01	2.46E-02	8.49E-01	9.07E-01	ATP-dependent Clp protease, protease subunit [EC:3.4.21.92]
KO:K01362	7.02	14.29	9.69	13.83	5.87	6.64	1.55	8.16E-01	6.17E-02	4.25E-01	7.76E-02	9.00E-01	8.48E-01	9.94E-01	
KO:K01365	0.00	0.00	0.00	0.00	0.00	0.00	5.42	1.00E+00	1.00E+00	1.00E+00	1.00E+00	1.00E+00	1.00E+00	2.70E-02	cathepsin L [EC:3.4.22.15]
KO:K01372	0.48	0.14	0.00	1.62	0.00	0.00	0.00	2.43E-01	4.12E-01	1.00E+00	8.83E-02	1.00E+00	1.00E+00	1.00E+00	bleomycin hydrolase [EC:3.4.22.4]
KO:K01376	0.72	0.01	4.27	0.00	0.00	0.00	0.00	1.47E-01	4.26E-01	2.91E-02	1.00E+00	1.00E+00	1.00E+00	1.00E+00	
KO:K01387	0.00	0.00	0.00	0.03	0.00	0.00	5.04	1.00E+00	1.00E+00	1.00E+00	2.41E-01	1.00E+00	1.00E+00	2.04E-02	microbial collagenase [EC:3.4.24.3]
KO:K01392	3.72	0.00	0.00	0.00	0.00	0.00	0.00	3.94E-02	1.00E+00	1.00E+00	1.00E+00	1.00E+00	1.00E+00	1.00E+00	thimet oligopeptidase [EC:3.4.24.15]
KO:K01404	0.00	0.00	0.63	0.00	0.00	0.00	0.00	1.00E+00	1.00E+00	1.63E-02	1.00E+00	1.00E+00	1.00E+00	1.00E+00	leishmanolysin [EC:3.4.24.36]
KO:K01406	4.15	0.06	0.00	0.00	0.00	0.00	0.00	6.06E-02	3.00E-01	1.00E+00	1.00E+00	1.00E+00	1.00E+00	1.00E+00	serralysin [EC:3.4.24.4]
KO:K01407	3.08	0.00	0.00	0.25	0.00	0.00	0.00	7.31E-02	1.00E+00	1.00E+00	2.82E-01	1.00E+00	1.00E+00	1.00E+00	protease III [EC:3.4.24.55]
KO:K01408	0.00	0.00	0.00	1.33	0.00	0.00	0.00	1.00E+00	1.00E+00	1.00E+00	1.43E-01	1.00E+00	1.00E+00	1.00E+00	insulysin [EC:3.4.24.56]
KO:K01409	7.32	5.78	4.56	3.35	6.73	3.19	2.02	1.21E-01	2.75E-01	5.24E-01	7.95E-01	1.62E-01	8.19E-01	9.33E-01	O-sialoglycoprotein endopeptidase [EC:3.4.24.57]
KO:K01412	1.16	9.34	0.00	3.11	0.00	0.00	0.95	5.96E-01	2.77E-02	1.00E+00	2.18E-01	1.00E+00	1.00E+00	6.67E-01	mitochondrial processing peptidase [EC:3.4.24.64]
KO:K01414	2.86	0.17	12.35	0.96	6.22	0.37	1.75	2.56E-01	9.28E-01	7.14E-03	7.75E-01	5.19E-02	9.09E-01	5.32E-01	oligopeptidase A [EC:3.4.24.7]
KO:K01415	1.11	0.12	2.70	1.28	0.00	0.00	0.00	2.25E-01	6.31E-01	8.99E-02	1.95E-01	1.00E+00	1.00E+00	1.00E+00	endothelin-converting enzyme [EC:3.4.24.71]
KO:K01417	2.71	9.26	3.57	2.52	8.78	4.48	0.00	6.91E-01	6.76E-02	5.20E-01	7.26E-01	7.91E-02	3.64E-01	1.00E+00	
KO:K01419	2.87	11.88	0.02	8.20	0.00	0.30	0.13	4.93E-01	1.73E-02	9.90E-01	4.67E-02	9.91E-01	9.53E-01	9.68E-01	ATP-dependent HslUV protease, peptidase subunit HslV [EC:3.4.25.-]
KO:K01420	4.36	2.81	1.79	1.28	3.61	0.32	0.01	8.47E-02	2.32E-01	4.88E-01	6.78E-01	1.31E-01	9.24E-01	9.85E-01	CRP/FNR family transcriptional regulator, anaerobic regulatory
KO:K01422	0.16	27.37	0.43	1.57	0.00	0.00	0.23	9.03E-01	2.43E-03	8.53E-01	6.06E-01	1.00E+00	1.00E+00	8.86E-01	
KO:K01423	5.79	1.00	0.40	4.99	3.06	5.12	0.26	1.42E-01	7.82E-01	8.82E-01	1.86E-01	3.92E-01	1.76E-01	9.02E-01	
KO:K01424	1.55	0.31	0.02	1.69	9.39	1.10	0.39	3.54E-01	8.13E-01	9.40E-01	3.19E-01	1.37E-02	4.97E-01	7.81E-01	L-asparaginase [EC:3.5.1.1]
KO:K01425	0.06	0.00	0.21	0.05	0.00	3.31	0.00	3.71E-01	1.00E+00	2.09E-01	4.00E-01	1.00E+00	6.86E-03	1.00E+00	glutaminase [EC:3.5.1.2]
KO:K01426	3.00	15.61	0.00	4.39	0.71	2.39	0.12	5.19E-01	1.47E-02	9.77E-01	3.05E-01	8.89E-01	6.44E-01	9.53E-01	amidase [EC:3.5.1.4]
KO:K01427	0.70	0.00	0.00	0.00	0.00	0.00	0.00	6.31E-02	1.00E+00	1.00E+00	1.00E+00	1.00E+00	1.00E+00	1.00E+00	urease [EC:3.5.1.5]
KO:K01428	1.13	10.36	0.38	1.01	2.03	4.62	0.33	7.86E-01	1.63E-02	9.19E-01	8.09E-01	5.53E-01	1.33E-01	9.24E-01	urease subunit alpha [EC:3.5.1.5]
KO:K01429	0.22	1.89	0.11	0.22	0.71	0.00	0.05	6.28E-01	4.66E-02	7.71E-01	6.32E-01	2.17E-01	1.00E+00	8.39E-01	urease subunit beta [EC:3.5.1.5]
KO:K01430	0.06	2.87	1.27	0.13	0.98	0.00	0.00	7.92E-01	4.43E-02	1.53E-01	7.17E-01	2.12E-01	1.00E+00	1.00E+00	urease subunit gamma [EC:3.5.1.5]
KO:K01431	0.71	13.88	0.00	0.69	0.00	0.00	0.00	5.97E-01	5.57E-03	1.00E+00	6.02E-01	1.00E+00	1.00E+00	1.00E+00	beta-ureidopropionase [EC:3.5.1.6]
KO:K01433	1.03	0.25	6.27	0.19	13.17	1.78	0.25	5.43E-01	8.65E-01	2.73E-02	8.93E-01	3.57E-03	2.84E-01	8.65E-01	formyltetrahydrofolate deformylase [EC:3.5.1.1]
KO:K01434	3.27	15.67	0.14	0.37	0.00	0.00	0.00	1.65E-01	3.71E-03	9.16E-01	8.61E-01	1.00E+00	1.00E+00	1.00E+00	penicillin amidase [EC:3.5.1.11]
KO:K01436	3.98	1.49	0.00	2.32	0.02	0.00	0.00	9.04E-02	3.10E-01	1.00E+00	1.96E-01	7.57E-01	1.00E+00	1.00E+00	aminoacylase [EC:3.5.1.14]
KO:K01437	0.00	0.00	0.00	0.00	19.26	0.00	0.00	5.47E-01	1.00E+00	1.00E+00	1.00E+00	0.00E+00	1.00E+00	1.00E+00	aspartoacylase [EC:3.5.1.15]
KO:K01438	2.71	13.68	0.19	3.89	2.24	0.59	0.09	5.76E-01	9.57E-03	9.70E-01	3.16E-01	6.89E-01	9.36E-01	9.78E-01	acetylornithine deacetylase [EC:3.5.1.16]
KO:K01439	5.44	6.92	3.70	5.59	0.52	4.21	2.66	2.95E-01	1.41E-01	6.51E-01	2.74E-01	9.80E-01	5.42E-01	8.39E-01	succinyl-diaminopimelate desuccinylase [EC:3.5.1.18]
KO:K01442	0.02	0.00	0.36	0.00	5.72	0.00	0.00	3.91E-01	1.00E+00	1.83E-01	1.00E+00	3.71E-03	1.00E+00	1.00E+00	choloylglycine hydrolase [EC:3.5.1.24]
KO:K01443	0.21	0.88	5.20	0.07	3.89	0.00	0.97	6.90E-01	3.68E-01	4.06E-02	7.92E-01	6.77E-02	1.00E+00	3.41E-01	N-acetylglucosamine-6-phosphate deacetylase [EC:3.5.1.25]
KO:K01444	2.19	0.99	0.54	1.07	0.00	0.00	0.00	1.36E-01	3.13E-01	4.60E-01	2.92E-01	1.00E+00	1.00E+00	1.00E+00	N4-(beta-N-acetylglucosaminyl)-L-asparaginase [EC:3.5.1.26]
KO:K01447	2.16	2.11	0.00	2.51	0.00	0.00	0.00	1.19E-01	1.23E-01	1.00E+00	9.57E-02	1.00E+00	1.00E+00	1.00E+00	N-acetylmuramoyl-L-alanine amidase [EC:3.5.1.28]
KO:K01448	11.91	5.26	0.22	4.11	0.49	1.54	0.25	1.11E-02	1.57E-01	9.83E-01	3.12E-01	9.66E-01	8.76E-01	9.82E-01	N-acetylmuramoyl-L-alanine amidase [EC:3.5.1.28]
KO:K01451	3.86	28.37	0.11	3.36	0.00	0.00	0.03	4.05E-01	1.57E-03	9.61E-01	4.89E-01	1.00E+00	1.00E+00	9.69E-01	hippurate hydrolase [EC:3.5.1.32]
KO:K01452	0.74	9.39	0.00	0.89	0.00	0.00	0.00	5.11E-01	1.99E-02	1.00E+00	4.51E-01	1.00E+00	1.00E+00	1.00E+00	chitin deacetylase [EC:3.5.1.41]
KO:K01453	7.39	1.25	0.00	0.88	1.32	0.00	0.50	4.61E-02	4.54E-01	1.00E+00	5.60E-01	4.32E-01	1.00E+00	6.77E-01	
KO:K01454	0.00	0.00	0.00	1.02	0.00	0.00	0.00	1.00E+00	1.00E+00	1.00E+00	8.69E-02	1.00E+00	1.00E+00	1.00E+00	
KO:K01455	3.61	1.29	0.00	0.12	0.03	0.00	0.01	6.84E-02	1.83E-01	1.00E+00	6.14E-01	7.11E-01	1.00E+00	7.62E-01	formamidase [EC:3.5.1.49]
KO:K01457	0.39	2.02	0.03	0.39	0.00	0.21	0.05	4.45E-01	5.04E-02	8.33E-01	4.40E-01	1.00E+00	6.10E-01	8.12E-01	allophanate hydrolase [EC:3.5.1.54]
KO:K01458	0.13	7.64	0.00	0.87	0.00	0.00	0.00	7.58E-01	1.36E-02	1.00E+00	3.45E-01	1.00E+00	1.00E+00	1.00E+00	N-formylglutamate deformylase [EC:3.5.1.68]
KO:K01459	0.01	4.69	0.00	2.06	0.00	0.00	0.00	8.15E-01	3.44E-02	1.00E+00	1.16E-01	1.00E+00	1.00E+00	1.00E+00	
KO:K01460	0.00	1.44	0.00	0.00	0.00	0.00	0.00	1.00E+00	1.03E-01	1.00E+00	1.00E+00	1.00E+00	1.00E+00	1.00E+00	glutathionylspermidine amidase/synthetase [EC:3.5.1.78 6.3.1.8]
KO:K01461	2.73	0.29	0.00	0.07	0.00	0.00	0.29	7.60E-02	3.44E-01	1.00E+00	4.89E-01	1.00E+00	1.00E+00	3.44E-01	
KO:K01462	7.44	6.57	4.44	4.67	7.90	0.34	0.88	1.06E-01	1.62E-01	5.10E-01	4.57E-01	8.54E-02	9.94E-01	9.80E-01	
KO:K01463	1.25	1.39	0.31	6.35	0.00	0.00	0.00	3.80E-01	3.46E-01	7.62E-01	2.93E-02	1.00E+00	1.00E+00	1.00E+00	
KO:K01464	0.57	25.85	0.00	1.93	0.00	0.00	0.21	8.56E-01	1.29E-03	1.00E+00	5.33E-01	1.00E+00	1.00E+00	9.11E-01	dihydropyrimidinase [EC:3.5.2.2]
KO:K01465	6.82	10.42	5.52	8.54	8.40	2.17	2.42	4.83E-01	8.19E-02	7.37E-01	1.97E-01	2.12E-01	9.78E-01	9.71E-01	dihydroorotase [EC:3.5.2.3]
KO:K01466	0.20	0.00	0.00	0.04	0.16	0.00	0.28	1.88E-01	1.00E+00	1.00E+00	3.48E-01	2.14E-01	1.00E+00	1.40E-01	allantoinase [EC:3.5.2.5]

KO:K01467	0.45	0.01	0.42	2.57	7.00	0.00	0.00	5.56E-01	8.45E-01	5.73E-01	9.30E-02	1.80E-02	1.00E+00	1.00E+00	beta-lactamase [EC:3.5.2.6]
KO:K01468	3.92	1.58	0.09	1.27	0.00	10.72	0.00	1.12E-01	4.14E-01	9.07E-01	5.04E-01	1.00E+00	2.06E-02	1.00E+00	imidazolonepropionase [EC:3.5.2.7]
KO:K01469	1.35	2.72	5.48	2.57	3.94	0.00	0.00	5.73E-01	2.81E-01	8.53E-02	3.04E-01	1.58E-01	1.00E+00	1.00E+00	5-oxoprolinase (ATP-hydrolysing) [EC:3.5.2.9]
KO:K01470	0.38	0.05	0.03	0.21	19.23	0.00	0.48	5.64E-01	8.19E-01	8.38E-01	6.85E-01	7.14E-04	1.00E+00	4.99E-01	creatinine amidohydrolase [EC:3.5.2.1]
KO:K01471	0.87	0.00	0.00	0.57	0.00	0.00	0.00	1.09E-01	1.00E+00	1.00E+00	1.68E-01	1.00E+00	1.00E+00	1.00E+00	
KO:K01473	5.36	10.63	0.35	1.56	0.00	5.30	0.19	1.73E-01	4.37E-02	8.85E-01	6.81E-01	1.00E+00	1.77E-01	9.07E-01	N-methylhydantoinase A [EC:3.5.2.14]
KO:K01474	4.44	10.21	0.71	1.47	0.00	7.11	0.24	2.40E-01	4.99E-02	8.35E-01	6.99E-01	1.00E+00	9.91E-02	8.97E-01	N-methylhydantoinase B [EC:3.5.2.14]
KO:K01476	0.09	7.99	0.00	0.72	0.00	0.00	0.00	8.05E-01	1.04E-02	1.00E+00	3.79E-01	1.00E+00	1.00E+00	1.00E+00	arginase [EC:3.5.3.1]
KO:K01477	2.70	0.16	0.00	1.77	0.00	0.00	0.05	1.27E-01	5.00E-01	1.00E+00	1.88E-01	1.00E+00	1.00E+00	5.73E-01	allantoicase [EC:3.5.3.4]
KO:K01478	0.00	0.00	0.00	0.71	0.00	0.00	0.00	1.00E+00	1.00E+00	1.00E+00	3.93E-02	1.00E+00	1.00E+00	1.00E+00	arginine deiminase [EC:3.5.3.6]
KO:K01479	0.36	0.01	0.00	0.50	0.00	0.67	3.42	3.50E-01	8.01E-01	1.00E+00	2.88E-01	1.00E+00	2.24E-01	3.56E-02	formiminoglutamase [EC:3.5.3.8]
KO:K01480	2.16	19.91	0.00	4.77	2.30	0.07	0.40	7.04E-01	7.14E-03	9.90E-01	2.93E-01	6.79E-01	9.75E-01	9.30E-01	agmatinase [EC:3.5.3.11]
KO:K01482	0.00	4.51	0.00	0.19	0.00	0.00	0.00	1.00E+00	1.11E-02	1.00E+00	4.74E-01	1.00E+00	1.00E+00	1.00E+00	dimethylargininase [EC:3.5.3.18]
KO:K01483	0.64	7.03	0.00	0.81	0.00	0.00	0.26	5.47E-01	1.64E-02	1.00E+00	4.63E-01	1.00E+00	1.00E+00	7.47E-01	ureidoglycolate hydrolase [EC:3.5.3.19]
KO:K01484	0.51	0.25	0.41	0.24	0.00	0.00	0.33	2.26E-01	3.93E-01	2.72E-01	3.97E-01	1.00E+00	1.00E+00	3.24E-01	succinylarginine dihydrolase [EC:3.5.3.23]
KO:K01485	0.07	5.94	0.00	0.95	9.05	0.00	0.01	8.73E-01	4.79E-02	1.00E+00	5.28E-01	2.04E-02	1.00E+00	8.99E-01	cytosine deaminase [EC:3.5.4.1]
KO:K01486	0.14	8.35	2.47	0.13	0.00	0.00	0.59	8.37E-01	1.23E-02	1.32E-01	8.39E-01	1.00E+00	1.00E+00	6.15E-01	adenine deaminase [EC:3.5.4.2]
KO:K01487	0.59	11.00	0.96	0.07	0.00	0.00	0.13	6.78E-01	3.43E-03	4.85E-01	9.14E-01	1.00E+00	1.00E+00	8.95E-01	guanine deaminase [EC:3.5.4.3]
KO:K01488	4.37	2.62	9.88	0.17	0.00	0.00	0.00	9.94E-02	2.01E-01	2.61E-02	8.21E-01	1.00E+00	1.00E+00	9.22E-01	adenosine deaminase [EC:3.5.4.4]
KO:K01489	0.58	10.32	0.00	0.09	1.76	0.00	0.00	6.55E-01	4.43E-03	1.00E+00	8.91E-01	2.09E-01	1.00E+00	1.00E+00	cytidine deaminase [EC:3.5.4.5]
KO:K01491	5.06	13.38	4.62	4.43	7.27	0.81	0.81	5.09E-01	2.26E-02	6.00E-01	6.40E-01	1.88E-01	9.80E-01	9.80E-01	methylenetetrahydrofolate dehydrogenase (NADP+) /
KO:K01492	0.00	0.00	1.88	0.00	0.00	0.00	0.00	1.00E+00	1.00E+00	1.47E-02	1.00E+00	1.00E+00	1.00E+00	1.00E+00	phosphoribosylaminoimidazolecarboxamide formyltransferase
KO:K01493	0.71	0.51	0.82	0.41	0.00	0.00	9.55	4.58E-01	5.46E-01	4.15E-01	5.98E-01	1.00E+00	1.00E+00	1.29E-02	dCMP deaminase [EC:3.5.4.12]
KO:K01494	5.21	11.77	3.04	2.75	4.42	0.30	0.09	2.32E-01	1.60E-02	6.78E-01	7.41E-01	3.62E-01	9.82E-01	9.90E-01	dCTP deaminase [EC:3.5.4.13]
KO:K01495	12.56	1.13	2.81	6.61	7.72	8.66	0.98	3.73E-02	9.52E-01	8.28E-01	2.49E-01	1.64E-01	1.14E-01	9.60E-01	GTP cyclohydrolase I [EC:3.5.4.16]
KO:K01496	1.70	2.14	2.81	2.89	1.43	2.55	2.18	6.16E-01	4.96E-01	3.36E-01	3.20E-01	6.91E-01	3.94E-01	4.84E-01	phosphoribosyl-AMP cyclohydrolase [EC:3.5.4.19]
KO:K01497	8.62	1.16	0.89	1.89	0.53	0.00	1.59	4.23E-02	6.54E-01	7.22E-01	4.74E-01	8.04E-01	1.00E+00	5.46E-01	GTP cyclohydrolase II [EC:3.5.4.25]
KO:K01499	0.03	0.00	1.40	1.09	0.06	0.00	0.00	5.60E-01	1.00E+00	6.77E-02	8.41E-02	5.09E-01	1.00E+00	1.00E+00	methenyltetrahydromethanopterin cyclohydrolase [EC:3.5.4.27]
KO:K01500	0.31	0.07	1.59	0.48	4.26	0.37	0.00	5.34E-01	7.52E-01	1.26E-01	4.16E-01	2.89E-02	4.89E-01	1.00E+00	
KO:K01501	1.31	0.16	0.03	0.51	10.91	0.91	0.09	2.73E-01	8.17E-01	8.93E-01	6.19E-01	5.00E-03	4.17E-01	8.54E-01	nitrilase [EC:3.5.5.1]
KO:K01502	0.11	0.54	0.00	0.00	0.00	0.00	0.00	2.13E-01	9.57E-02	1.00E+00	1.00E+00	1.00E+00	1.00E+00	1.00E+00	aliphatic nitrilase [EC:3.5.5.7]
KO:K01504	0.00	0.44	0.00	0.00	0.00	0.00	0.00	1.00E+00	4.00E-02	1.00E+00	1.00E+00	1.00E+00	1.00E+00	1.00E+00	glucosamine-6-phosphate isomerase [EC:3.5.99.6]
KO:K01505	4.14	0.66	0.00	0.98	0.00	0.16	0.00	3.31E-02	4.29E-01	1.00E+00	2.96E-01	1.00E+00	7.50E-01	1.00E+00	1-aminocyclopropane-1-carboxylate deaminase [EC:3.5.99.7]
KO:K01506	3.60	2.90	0.00	7.32	0.00	5.06	0.00	2.55E-01	3.45E-01	1.00E+00	7.57E-02	1.00E+00	1.46E-01	1.00E+00	
KO:K01507	9.74	4.23	4.18	18.58	9.41	0.61	5.18	2.54E-01	9.09E-01	9.11E-01	1.69E-02	2.90E-01	9.94E-01	8.51E-01	inorganic pyrophosphatase [EC:3.6.1.1]
KO:K01512	0.00	0.00	0.04	0.02	27.01	0.00	0.17	7.49E-01	7.53E-01	6.19E-01	6.99E-01	0.00E+00	1.00E+00	4.54E-01	acylphosphatase [EC:3.6.1.7]
KO:K01514	2.36	14.80	1.21	0.70	5.34	1.06	0.05	6.06E-01	5.29E-03	8.47E-01	9.13E-01	1.29E-01	8.69E-01	9.87E-01	exopolyphosphatase [EC:3.6.1.11]
KO:K01515	3.47	0.05	1.90	0.55	6.37	0.37	0.67	8.96E-02	9.28E-01	2.59E-01	7.22E-01	2.79E-02	7.98E-01	6.72E-01	ADP-ribose pyrophosphatase [EC:3.6.1.13]
KO:K01516	1.11	4.28	1.60	2.07	6.63	0.65	0.71	8.48E-01	8.93E-02	7.03E-01	5.22E-01	2.80E-02	9.30E-01	9.17E-01	nucleoside-triphosphatase [EC:3.6.1.15]
KO:K01520	1.28	4.34	4.95	2.88	0.00	0.35	0.48	6.80E-01	8.11E-02	5.76E-02	2.07E-01	1.00E+00	9.15E-01	8.97E-01	dUTP pyrophosphatase [EC:3.6.1.23]
KO:K01523	2.85	1.01	1.03	1.64	0.00	5.39	1.71	1.97E-01	6.52E-01	6.43E-01	4.49E-01	1.00E+00	5.73E-02	4.29E-01	phosphoribosyl-ATP pyrophosphohydrolase [EC:3.6.1.31]
KO:K01524	1.60	0.03	0.00	0.63	0.00	0.00	0.03	7.67E-02	6.17E-01	1.00E+00	1.90E-01	1.00E+00	1.00E+00	6.25E-01	guanosine-5'-triphosphate,3'-diphosphate pyrophosphatase
KO:K01525	3.61	0.05	0.01	1.47	0.00	0.38	1.73	5.87E-02	8.69E-01	8.97E-01	2.28E-01	1.00E+00	6.69E-01	1.79E-01	bis(5'-nucleosyl)-tetraphosphatase (symmetrical) [EC:3.6.1.41]
KO:K01529	0.13	0.00	0.36	0.10	17.24	0.00	0.02	5.99E-01	8.44E-01	3.79E-01	6.53E-01	3.43E-03	1.00E+00	8.11E-01	
KO:K01533	1.84	4.58	1.90	1.01	15.53	0.18	1.16	7.01E-01	1.03E-01	6.83E-01	8.96E-01	1.14E-03	9.84E-01	8.68E-01	Cu2+-exporting ATPase [EC:3.6.3.4]
KO:K01534	0.00	0.02	3.58	0.00	0.00	0.00	0.03	5.60E-01	4.48E-01	3.43E-03	5.65E-01	1.00E+00	1.00E+00	4.00E-01	Cd2+/Zn2+-exporting ATPase [EC:3.6.3.3 3.6.3.5]
KO:K01535	0.00	0.00	0.00	0.02	0.00	0.00	1.90	1.00E+00	1.00E+00	1.00E+00	3.17E-01	1.00E+00	1.00E+00	8.14E-03	H+-transporting ATPase [EC:3.6.3.6]
KO:K01537	0.18	0.03	0.10	0.23	0.10	0.00	0.05	2.60E-01	4.97E-01	3.47E-01	2.28E-01	3.47E-01	1.00E+00	4.55E-01	Ca2+-transporting ATPase [EC:3.6.3.8]
KO:K01539	0.04	0.00	0.00	0.13	0.00	0.00	0.00	1.79E-01	1.00E+00	1.00E+00	1.45E-01	1.00E+00	1.00E+00	1.00E+00	sodium/potassium-transporting ATPase subunit alpha [EC:3.6.3.9]
KO:K01551	0.36	0.00	0.37	0.00	0.04	2.61	0.00	1.85E-01	1.00E+00	1.85E-01	4.77E-01	3.39E-01	3.04E-02	1.00E+00	arsenite-transporting ATPase [EC:3.6.3.16]
KO:K01552	0.06	0.14	2.54	0.11	0.76	0.00	0.18	6.73E-01	5.47E-01	2.51E-02	5.80E-01	1.35E-01	1.00E+00	4.82E-01	
KO:K01554	0.14	0.00	0.00	0.25	0.00	0.00	0.12	2.02E-01	1.00E+00	1.00E+00	1.50E-01	1.00E+00	1.00E+00	2.22E-01	
KO:K01555	0.29	6.40	0.01	0.81	0.00	3.76	0.00	7.84E-01	2.27E-02	9.10E-01	5.29E-01	1.00E+00	6.54E-02	1.00E+00	fumarylacetoacetase [EC:3.7.1.2]
KO:K01556	3.25	8.86	0.00	2.60	0.00	0.06	0.00	1.52E-01	1.29E-02	1.00E+00	2.32E-01	1.00E+00	9.49E-01	1.00E+00	kynureninase [EC:3.7.1.3]
KO:K01557	0.03	1.15	0.00	0.52	0.00	0.00	0.00	4.21E-01	1.10E-01	1.00E+00	1.96E-01	1.00E+00	1.00E+00	1.00E+00	acylpyruvate hydrolase [EC:3.7.1.5]
KO:K01559	0.06	0.00	0.00	1.25	0.00	0.00	0.00	3.12E-01	1.00E+00	1.00E+00	6.21E-02	1.00E+00	1.00E+00	1.00E+00	
KO:K01560	0.30	9.11	0.00	4.32	0.00	0.28	0.00	8.34E-01	2.01E-02	1.00E+00	8.11E-02	1.00E+00	8.43E-01	1.00E+00	2-haloacid dehalogenase [EC:3.8.1.2]
KO:K01561	0.02	10.34	0.00	0.02	0.00	0.00	0.00	6.82E-01	8.29E-03	1.00E+00	6.79E-01	1.00E+00	1.00E+00	1.00E+00	haloacetate dehalogenase [EC:3.8.1.3]
KO:K01563	4.26	0.11	4.59	0.81	0.26	0.00	0.64	8.43E-02	7.60E-01	7.66E-02	4.76E-01	6.87E-01	1.00E+00	5.32E-01	haloalkane dehalogenase [EC:3.8.1.5]
KO:K01564	1.47	0.00	0.87	0.04	0.00	0.00	0.10	8.34E-02	6.10E-01	1.34E-01	5.13E-01	1.00E+00	1.00E+00	4.24E-01	
KO:K01565	0.01	0.00	10.32	0.06	0.27	0.00	2.27	5.39E-01	1.00E+00	1.19E-02	4.87E-01	3.59E-01	1.00E+00	1.04E-01	N-sulfoglucosamine sulfohydrolase [EC:3.1.1.1]
KO:K01567	3.44	1.04	0.83	2.01	0.96	0.31	2.40	1.63E-01	6.31E-01	6.94E-01	3.63E-01	6.52E-01	8.46E-01	2.90E-01	
KO:K01571	7.78	0.76	3.53	1.77	0.00	0.00	6.13	6.16E-02	7.91E-01	2.65E-01	5.63E-01	1.00E+00	1.00E+00	1.04E-01	oxaloacetate decarboxylase, alpha subunit [EC:4.1.1.3]
KO:K01572	5.31	0.61	10.15	0.58	0.00	0.00	3.22	9.47E-02	7.58E-01	2.14E-02	7.65E-01	1.00E+00	1.00E+00	2.07E-01	oxaloacetate decarboxylase, beta subunit [EC:4.1.1.3]
KO:K01573	1.24	0.28	0.04	0.00	0.00	0.00	2.25	1.24E-01	3.71E-01	6.47E-01	1.00E+00	1.00E+00	1.00E+00	5.37E-02	oxaloacetate decarboxylase, gamma subunit [EC:4.1.1.3]
KO:K01574	0.00	0.00	0.00	0.20	0.00	0.00	0.00	1.00E+00	1.00E+00	1.00E+00	1.43E-01	1.00E+00	1.00E+00	1.00E+00	acetoacetate decarboxylase [EC:4.1.1.4]
KO:K01575	0.09	0.00	0.00	0.00	2.56	0.00	0.00	2.56E-01	1.00E+00	1.00E+00	4.06E-01	1.71E-02	1.00E+00	1.00E+00	acetolactate decarboxylase [EC:4.1.1.5]
KO:K01576	0.00	0.00	0.43	0.69	5.27	0.00	0.00	1.00E+00	5.33E-01	2.42E-01	1.88E-01	3.07E-02	1.00E+00	1.00E+00	benzoylformate decarboxylase [EC:4.1.1.7]
KO:K01577	0.20	0.21	0.00	6.22	0.00	0.00	0.00	4.75E-01	4.70E-01	1.00E+00	3.77E-02	1.00E+00	1.00E+00	1.00E+00	oxalyl-CoA decarboxylase [EC:4.1.1.8]
KO:K01578	1.20	8.51	0.07	2.75	0.00	1.33	0.00	5.88E-01	2.66E-02	9.04E-01	2.25E-01	1.00E+00	5.44E-01	1.00E+00	malonyl-CoA decarboxylase [EC:4.1.1.9]
KO:K01579	1.36	0.27	3.50	2.33	0.00	0.05	5.69	4.26E-01	8.14E-01	1.10E-01	2.14E-01	1.00E+00	9.11E-01	5.21E-02	aspartate 1-decarboxylase [EC:4.1.1.11]
KO:K01580	0.07	0.00	0.04	0.03	6.55	0.00	0.00	4.43E-01	1.00E+00	5.13E-01	5.49E-01	3.00E-03	1.00E+00	1.00E+00	glutamate decarboxylase [EC:4.1.1.15]
KO:K01581	0.29	7.78	1.01	6.95	0.00	0.00	0.05	8.71E-01	3.94E-02	6.81E-01	4.91E-02	1.00E+00	1.00E+00	9.24E-01	ornithine decarboxylase [EC:4.1.1.17]
KO:K01582	0.03	0.01	0.00	0.00	6.78	0.00	0.00	4.26E-01	4.57E-01	1.00E+00	1.00E+00	2.43E-03	1.00E+00	1.00E+00	lysine decarboxylase [EC:4.1.1.18]
KO:K01583	0.00	0.00	0.00	0.00	3.01	0.00	0.00	2.44E-01	1.00E+00	1.00E+00	1.00E+00	1.33E-02	1.00E+00	1.00E+00	arginine decarboxylase [EC:4.1.1.19]
KO:K01584	0.00	0.19	0.00	2.09	0.00	0.00	0.43	1.00E+00	3.64E-01	1.00E+00	7.94E-02	1.00E+00	1.00E+00	2.52E-01	arginine decarboxylase [EC:4.1.1.19]
KO:K01585	1.42	1.22	5.38	0.41	8.33	3.28	1.20	5.93E-01	6.58E-01	6.93E-02	8.68E-01	2.49E-02	1.87E-01	6.65E-01	arginine decarboxylase [EC:4.1.1.19]
KO:K01586	4.30	9.07	7.04	3.98	9.51	2.27	2.52	7.24E-01	6.79E-02	1.91E-01	7.81E-01	5.66E-02	9.45E-01	9.31E-01	diaminopimelate decarboxylase [EC:4.1.1.2]
KO:K01588	6.65	5.51	3.01	3.83	6.47	0.61	0.51	1.12E-01	2.02E-01	6.93E-01	4.89E-01	1.21E-01	9.70E-01	9.76E-01	5-(carboxyamino)imidazole ribonucleotide mutase [EC:5.4.99.18]
KO:K01589	2.25	7.48	2.69	2.97	8.05	2.36	4.53	8.63E-01	8.27E-02	8.03E-01	7.49E-01	6.66E-02	8.49E-01	3.76E-01	5-(carboxyamino)imidazole ribonucleotide synthase [EC:6.3.4.18]
KO:K01590	0.00	0.58	0.00	0.01	0.00	0.00	0.00	1.00E+00	9.07E-02	1.00E+00	2.21E-01	1.00E+00	1.00E+00	1.00E+00	histidine decarboxylase [EC:4.1.1.22]
KO:K01591	4.16	3.97	4.05	2.65	5.24	2.92	3.93	3.63E-01	4.12E-01	3.92E-01	7.81E-01	1.79E-01	7.30E-01	4.23E-01	orotidine-5'-phosphate decarboxylase [EC:4.1.1.23]
KO:K01592	0.00	0.33	0.00	0.00	0.00	0.00	0.00	1.00E+00	1.01E-01	1.00E+00	1.00E+00	1.00E+00	1.00E+00	1.00E+00	tyrosine decarboxylase [EC:4.1.1.25]
KO:K01593	0.29	4.21	0.00	0.44	0.00	0.00	0.00	5.19E-01	2.30E-02	1.00E+00	4.14E-01	1.00E+00	1.00E+00	1.00E+00	aromatic-L-amino-acid decarboxylase [EC:4.1.1.28]
KO:K01594	0.00	0.00	0.00	0.14	7.14	0.00	0.00	1.00E+00	1.00E+00	1.00E+00	2.83E-01	5.86E-03	1.00E+00	1.00E+00	sulfinoalanine decarboxylase [EC:4.1.1.29]
KO:K01595	3.67	0.67	11.91	1.75	7.68	0.31	1.24	2.65E-01	8.86E-01	1.51E-02	6.74E-01	5.07E-02	9.35E-01	7.86E-01	phosphoenolpyruvate carboxylase [EC:4.1.1.31]
KO:K01596	0.34	0.00	14.36	0.51	0.14	0.00	1.29	4.72E-01	1.00E+00	1.61E-02	4.19E-01	5.52E-01	1.00E+00	2.72E-01	phosphoenolpyruvate carboxykinase (GTP) [EC:4.1.1.32]
KO:K01597	1.90	1.26	0.00	0.85	0.00	5.39	0.00	2.07E-01	3.16E-01	1.00E+00	4.31E-01	1.00E+00	5.36E-02	1.00E+00	diphosphomevalonate decarboxylase [EC:4.1.1.33]
KO:K01599	5.70	6.74	6.71	3.19	5.73	3.55	4.95	3.72E-01	2.10E-01	2.13E-01	8.50E-01	3.67E-01	8.05E-01	5.34E-01	uroporphyrinogen decarboxylase [EC:4.1.1.37]
KO:K01601	0.18	0.12	0.00	1.19	30.81	4.39	3.49	7.99E-01	8.10E-01	1.00E+00	5.31E-01	2.29E-03	1.37E-01	1.86E-01	ribulose-bisphosphate carboxylase large chain [EC:4.1.1.39]
KO:K01602	0.02	0.03	0.00	0.36	28.15	0.39	1.19	7.32E-01	7.19E-01	1.00E+00	5.24E-01	8.57E-04	5.13E-01	2.96E-01	ribulose-bisphosphate carboxylase small chain [EC:4.1.1.39]
KO:K01607	0.66	16.12	0.00	2.20	0.01	0.00	0.00	7.27E-01	6.00E-03	1.00E+00	3.23E-01	9.15E-01	1.00E+00	1.00E+00	4-carboxymuconolactone decarboxylase [EC:4.1.1.44]
KO:K01609	3.00	2.41	4.99	1.44	10.41	1.12	2.52	4.49E-01	6.32E-01	1.10E-01	8.68E-01	9.43E-03	9.08E-01	5.98E-01	indole-3-glycerol phosphate synthase [EC:4.1.1.48]
KO:K01610	9.11	16.98	4.25	4.72	0.04	5.43	2.44	1.42E-01	8.86E-03	8.05E-01	7.46E-01	9.95E-01	6.26E-01	9.33E-01	phosphoenolpyruvate carboxykinase (ATP) [EC:4.1.1.49]
KO:K01611	0.29	2.68	0.07	0.61	12.22	0.05	0.16	7.19E-01	1.77E-01	8.06E-01	5.96E-01	1.26E-02	8.15E-01	7.68E-01	S-adenosylmethionine decarboxylase [EC:4.1.1.5]
KO:K01613	5.12	4.72	11.04	6.20	0.00	0.18	0.78	2.13E-01	2.61E-01	2.66E-02	1.29E-01	9.97E-01	9.82E-01	9.38E-01	phosphatidylserine decarboxylase [EC:4.1.1.65]

KO:K01617	2.20	0.00	0.00	0.59	0.00	0.75	0.29	8.03E-02	1.00E+00	1.00E+00	2.91E-01	1.00E+00	2.49E-01	4.16E-01	4-oxalocrotonate decarboxylase [EC:4.1.1.77]
KO:K01618	1.69	0.71	0.14	1.15	4.78	0.06	0.26	2.62E-01	5.20E-01	7.96E-01	3.81E-01	6.27E-02	8.41E-01	7.31E-01	
KO:K01619	0.02	5.89	3.11	0.09	9.16	0.00	0.13	9.35E-01	3.71E-02	1.14E-01	9.02E-01	1.24E-02	1.00E+00	8.93E-01	deoxyribose-phosphate aldolase [EC:4.1.2.4]
KO:K01620	2.61	8.78	0.22	3.17	1.12	1.53	0.02	4.21E-01	3.06E-02	9.54E-01	3.01E-01	8.12E-01	7.08E-01	9.87E-01	threonine aldolase [EC:4.1.2.5]
KO:K01621	0.12	0.00	0.00	0.00	0.00	0.00	0.00	6.64E-02	1.00E+00	1.00E+00	1.00E+00	1.00E+00	1.00E+00	1.00E+00	phosphoketolase [EC:4.1.2.9]
KO:K01622	0.00	0.00	0.00	0.00	0.00	0.00	7.82	1.00E+00	1.00E+00	1.00E+00	3.36E-01	1.00E+00	1.00E+00	5.57E-03	
KO:K01623	0.92	4.98	1.93	11.35	1.08	0.25	0.14	8.70E-01	1.65E-01	6.89E-01	2.16E-02	8.48E-01	9.45E-01	9.59E-01	fructose-bisphosphate aldolase, class I [EC:4.1.2.13]
KO:K01624	6.97	3.35	9.18	3.94	8.33	5.78	5.78	2.60E-01	8.35E-01	1.07E-01	7.66E-01	1.51E-01	4.34E-01	4.34E-01	fructose-bisphosphate aldolase, class II [EC:4.1.2.13]
KO:K01625	1.05	4.30	2.48	0.84	9.91	0.00	0.13	7.46E-01	7.13E-02	2.45E-01	8.15E-01	5.71E-03	1.00E+00	9.67E-01	2-dehydro-3-deoxyphosphogluconate aldolase /
KO:K01626	9.53	14.02	9.68	5.37	4.89	2.45	1.46	1.86E-01	3.76E-02	1.75E-01	7.54E-01	8.14E-01	9.55E-01	9.78E-01	3-deoxy-7-phosphoheptulonate synthase [EC:2.5.1.54]
KO:K01627	6.26	2.57	7.31	5.11	2.41	4.31	5.77	2.01E-01	8.82E-01	1.18E-01	3.85E-01	8.94E-01	5.75E-01	2.64E-01	2-dehydro-3-deoxyphosphooctonate aldolase (KDO 8-P synthase)
KO:K01628	0.03	0.02	6.95	3.66	2.84	0.21	0.00	8.30E-01	8.37E-01	3.39E-02	8.24E-02	1.22E-01	7.14E-01	1.00E+00	L-fuculose-phosphate aldolase [EC:4.1.2.17]
KO:K01630	0.00	0.30	1.06	0.33	0.68	0.00	0.00	5.33E-01	2.90E-01	1.20E-01	2.80E-01	1.83E-01	1.00E+00	1.00E+00	2-dehydro-3-deoxyglucarate aldolase [EC:4.1.2.2]
KO:K01631	2.09	7.22	0.00	1.44	0.00	0.00	0.31	2.61E-01	2.71E-02	1.00E+00	4.04E-01	1.00E+00	1.00E+00	8.09E-01	2-dehydro-3-deoxyphosphogalactonate aldolase [EC:4.1.2.21]
KO:K01632	0.00	0.11	0.00	0.00	0.00	0.00	0.00	1.00E+00	1.04E-01	1.00E+00	1.00E+00	1.00E+00	1.00E+00	1.00E+00	fructose-6-phosphate phosphoketolase [EC:4.1.2.22]
KO:K01633	3.93	8.02	0.45	2.10	3.84	0.24	0.67	2.34E-01	3.70E-02	9.49E-01	6.61E-01	2.48E-01	9.69E-01	9.23E-01	dihydroneopterin aldolase [EC:4.1.2.25]
KO:K01634	1.88	0.00	0.00	1.92	0.00	0.00	0.00	1.33E-01	1.00E+00	1.00E+00	1.32E-01	1.00E+00	1.00E+00	1.00E+00	sphinganine-1-phosphate aldolase [EC:4.1.2.27]
KO:K01635	0.00	0.00	0.00	3.37	0.00	0.00	0.00	1.00E+00	1.00E+00	1.00E+00	7.66E-02	1.00E+00	1.00E+00	1.00E+00	tagatose 1,6-diphosphate aldolase [EC:4.1.2.4]
KO:K01636	0.00	0.03	0.02	0.00	6.54	0.00	0.39	5.71E-01	4.51E-01	4.62E-01	5.74E-01	6.14E-03	1.00E+00	2.02E-01	
KO:K01637	10.74	0.39	4.24	5.43	0.00	8.32	0.92	5.40E-02	9.07E-01	3.43E-01	2.27E-01	1.00E+00	8.69E-02	8.64E-01	isocitrate lyase [EC:4.1.3.1]
KO:K01638	7.46	16.81	0.68	8.78	0.48	6.52	0.16	2.71E-01	1.80E-02	9.76E-01	1.74E-01	9.81E-01	3.78E-01	9.87E-01	malate synthase [EC:2.3.3.9]
KO:K01639	0.01	0.00	5.52	0.02	1.38	0.00	0.76	6.20E-01	6.42E-01	1.36E-02	6.06E-01	9.71E-02	1.00E+00	1.72E-01	N-acetylneuraminate lyase [EC:4.1.3.3]
KO:K01640	7.94	5.93	0.02	4.03	0.00	1.31	0.00	3.21E-02	6.83E-02	9.87E-01	1.76E-01	1.00E+00	7.93E-01	1.00E+00	hydroxymethylglutaryl-CoA lyase [EC:4.1.3.4]
KO:K01641	0.72	0.25	0.00	0.01	0.00	0.00	4.46	2.18E-01	3.48E-01	1.00E+00	5.30E-01	1.00E+00	1.00E+00	4.43E-02	hydroxymethylglutaryl-CoA synthase [EC:2.3.3.1]
KO:K01644	1.20	6.94	0.00	8.21	0.00	0.00	0.52	6.43E-01	7.14E-02	1.00E+00	5.03E-02	1.00E+00	1.00E+00	8.05E-01	citrate lyase subunit beta / citryl-CoA lyase [EC:4.1.3.6 4.1.3.34]
KO:K01647	10.33	14.23	16.98	9.15	2.56	7.77	1.41	2.43E-01	4.99E-02	1.74E-02	4.14E-01	9.85E-01	6.53E-01	9.95E-01	citrate synthase [EC:2.3.3.1]
KO:K01649	6.19	10.63	16.68	7.78	6.78	3.43	6.90	8.08E-01	1.63E-01	1.40E-02	5.55E-01	7.31E-01	9.67E-01	7.12E-01	2-isopropylmalate synthase [EC:2.3.3.13]
KO:K01652	10.94	16.80	10.01	14.52	7.01	1.85	7.32	3.70E-01	3.59E-02	5.12E-01	8.41E-02	8.84E-01	9.96E-01	8.65E-01	acetolactate synthase I/II/III large subunit [EC:2.2.1.6]
KO:K01653	2.46	3.19	14.46	3.37	8.13	1.47	4.27	8.21E-01	6.96E-01	8.57E-03	6.55E-01	7.70E-02	9.22E-01	4.54E-01	acetolactate synthase I/III small subunit [EC:2.2.1.6]
KO:K01654	2.31	1.79	1.46	3.61	0.92	4.40	2.14	4.76E-01	6.12E-01	6.94E-01	2.37E-01	8.17E-01	1.56E-01	5.15E-01	N-acetylneuraminate synthase [EC:2.5.1.56]
KO:K01655	0.00	0.00	0.00	0.20	0.00	0.00	0.00	1.00E+00	1.00E+00	1.00E+00	1.43E-01	1.00E+00	1.00E+00	1.00E+00	homocitrate synthase [EC:2.3.3.14]
KO:K01657	6.97	5.26	9.03	3.19	6.46	2.37	5.16	2.10E-01	4.82E-01	8.77E-02	8.44E-01	2.70E-01	9.12E-01	5.03E-01	anthranilate synthase component I [EC:4.1.3.27]
KO:K01658	6.52	3.87	3.87	2.75	0.70	0.70	4.12	8.21E-02	3.48E-01	3.49E-01	6.37E-01	9.56E-01	9.56E-01	2.98E-01	anthranilate synthase component II [EC:4.1.3.27]
KO:K01659	1.36	0.43	3.30	1.21	1.52	8.82	2.96	5.51E-01	8.04E-01	2.30E-01	5.92E-01	5.10E-01	3.66E-02	2.66E-01	2-methylcitrate synthase [EC:2.3.3.5]
KO:K01661	0.80	0.23	5.77	0.57	5.77	0.73	0.00	4.88E-01	7.58E-01	4.13E-02	5.83E-01	4.13E-02	5.15E-01	1.00E+00	naphthoate synthase [EC:4.1.3.36]
KO:K01662	6.74	13.29	9.34	8.67	12.87	1.33	2.27	6.99E-01	3.21E-02	2.18E-01	3.07E-01	4.11E-02	9.97E-01	9.90E-01	1-deoxy-D-xylulose-5-phosphate synthase [EC:2.2.1.7]
KO:K01663	1.23	30.85	0.00	1.16	0.00	0.00	0.00	6.97E-01	1.00E-03	1.00E+00	7.12E-01	1.00E+00	1.00E+00	1.00E+00	
KO:K01664	0.39	0.02	0.00	0.26	14.00	0.00	0.00	3.82E-01	6.77E-01	1.00E+00	4.49E-01	5.00E-03	1.00E+00	1.00E+00	para-aminobenzoate synthetase component II [EC:2.6.1.85]
KO:K01665	1.33	2.30	0.19	2.10	2.41	0.85	0.05	4.60E-01	1.76E-01	9.22E-01	2.12E-01	1.58E-01	6.91E-01	9.58E-01	para-aminobenzoate synthetase component I [EC:2.6.1.85]
KO:K01666	0.86	0.04	0.00	0.44	2.51	5.65	0.21	3.19E-01	7.20E-01	1.00E+00	4.77E-01	1.05E-01	3.13E-02	6.13E-01	4-hydroxy 2-oxovalerate aldolase [EC:4.1.3.39]
KO:K01667	0.00	0.32	0.00	1.96	0.00	0.00	0.00	1.00E+00	2.42E-01	1.00E+00	8.54E-02	1.00E+00	1.00E+00	1.00E+00	tryptophanase [EC:4.1.99.1]
KO:K01669	9.65	8.37	8.14	6.92	5.01	6.32	1.36	1.25E-01	2.21E-01	2.50E-01	4.38E-01	7.94E-01	5.66E-01	9.89E-01	deoxyribodipyrimidine photo-lyase [EC:4.1.99.3]
KO:K01672	0.34	0.00	0.00	0.17	0.00	0.00	0.00	9.46E-02	1.00E+00	1.00E+00	2.12E-01	1.00E+00	1.00E+00	1.00E+00	carbonic anhydrase [EC:4.2.1.1]
KO:K01673	4.00	12.24	5.22	1.79	2.89	3.81	0.79	5.30E-01	2.69E-02	3.10E-01	8.78E-01	7.41E-01	5.67E-01	9.51E-01	carbonic anhydrase [EC:4.2.1.1]
KO:K01674	0.00	0.00	0.00	2.04	0.00	0.00	0.00	1.00E+00	1.00E+00	1.00E+00	1.37E-02	1.00E+00	1.00E+00	1.00E+00	carbonic anhydrase [EC:4.2.1.1]
KO:K01676	8.87	0.77	6.35	3.21	0.00	3.31	3.70	5.81E-02	8.95E-01	1.24E-01	4.60E-01	1.00E+00	4.42E-01	3.71E-01	fumarate hydratase, class I [EC:4.2.1.2]
KO:K01677	0.00	0.96	0.00	0.71	0.00	0.00	0.00	1.00E+00	1.13E-01	1.00E+00	2.00E-01	1.00E+00	1.00E+00	1.00E+00	fumarate hydratase subunit alpha [EC:4.2.1.2]
KO:K01679	3.05	3.95	0.78	3.81	4.77	5.55	0.81	5.01E-01	2.99E-01	9.28E-01	3.25E-01	1.86E-01	1.22E-01	9.24E-01	fumarate hydratase, class II [EC:4.2.1.2]
KO:K01681	5.37	10.44	11.77	10.98	0.97	7.85	1.49	7.80E-01	9.79E-02	5.61E-02	7.77E-02	9.90E-01	3.42E-01	9.83E-01	aconitate hydratase 1 [EC:4.2.1.3]
KO:K01682	9.45	4.40	5.27	3.71	3.76	4.46	5.39	1.32E-01	6.07E-01	4.70E-01	7.25E-01	7.16E-01	5.98E-01	4.52E-01	aconitate hydratase 2 [EC:4.2.1.3]
KO:K01684	0.08	0.34	2.83	0.29	0.37	0.00	0.04	5.19E-01	3.24E-01	5.84E-02	3.51E-01	3.11E-01	1.00E+00	5.81E-01	galactonate dehydratase [EC:4.2.1.6]
KO:K01685	1.65	4.75	0.05	6.49	0.05	0.00	1.17	5.04E-01	1.43E-01	8.78E-01	8.50E-02	8.78E-01	1.00E+00	6.14E-01	altronate hydrolase [EC:4.2.1.7]
KO:K01686	2.67	1.29	2.90	1.38	0.00	0.00	0.00	1.36E-01	3.24E-01	1.19E-01	3.01E-01	1.00E+00	1.00E+00	1.00E+00	mannonate dehydratase [EC:4.2.1.8]
KO:K01687	5.03	12.86	16.64	11.92	7.58	0.83	6.01	9.02E-01	8.23E-02	2.50E-02	1.16E-01	6.19E-01	9.97E-01	8.33E-01	dihydroxy-acid dehydratase [EC:4.2.1.9]
KO:K01689	9.49	7.38	12.63	5.45	5.71	7.60	5.55	1.49E-01	5.03E-01	2.86E-02	8.65E-01	8.37E-01	4.51E-01	8.54E-01	enolase [EC:4.2.1.11]
KO:K01690	8.20	13.49	0.58	2.07	0.00	0.00	0.08	4.63E-02	9.14E-03	9.26E-01	6.80E-01	1.00E+00	1.00E+00	9.75E-01	phosphogluconate dehydratase [EC:4.2.1.12]
KO:K01692	17.28	23.86	0.26	14.16	0.00	2.80	0.11	4.66E-02	1.50E-02	9.87E-01	8.37E-02	9.97E-01	9.03E-01	9.92E-01	enoyl-CoA hydratase [EC:4.2.1.17]
KO:K01693	3.03	5.20	9.29	5.03	5.57	0.05	7.08	7.74E-01	3.60E-01	6.64E-02	3.91E-01	3.04E-01	9.95E-01	1.55E-01	imidazoleglycerol-phosphate dehydratase [EC:4.2.1.19]
KO:K01695	4.60	4.07	11.79	2.28	5.82	2.67	4.52	4.36E-01	5.49E-01	2.20E-02	8.60E-01	2.37E-01	8.12E-01	4.54E-01	tryptophan synthase alpha chain [EC:4.2.1.2]
KO:K01696	6.59	9.40	11.42	6.45	8.19	1.66	5.72	5.81E-01	1.64E-01	7.27E-02	6.09E-01	2.90E-01	9.85E-01	7.38E-01	tryptophan synthase beta chain [EC:4.2.1.2]
KO:K01697	3.99	2.08	0.00	2.96	0.00	6.36	0.02	1.56E-01	3.60E-01	1.00E+00	2.41E-01	1.00E+00	7.87E-02	8.39E-01	cystathionine beta-synthase [EC:4.2.1.22]
KO:K01698	5.44	4.19	9.49	4.45	7.73	0.21	3.23	2.73E-01	5.96E-01	2.63E-02	5.22E-01	6.86E-02	9.99E-01	8.15E-01	porphobilinogen synthase [EC:4.2.1.24]
KO:K01702	0.00	0.01	0.00	0.62	0.00	0.00	0.00	1.00E+00	1.99E-01	1.00E+00	1.10E-01	1.00E+00	1.00E+00	1.00E+00	3-isopropylmalate dehydratase [EC:4.2.1.33]
KO:K01703	7.52	9.81	13.07	7.98	9.24	2.10	5.33	5.64E-01	1.79E-01	4.13E-02	4.59E-01	2.46E-01	9.90E-01	8.87E-01	3-isopropylmalate/(R)-2-methylmalate dehydratase large subunit
KO:K01704	5.42	8.63	6.18	2.78	7.44	3.30	2.20	3.98E-01	7.83E-02	2.65E-01	8.90E-01	1.31E-01	8.31E-01	9.31E-01	3-isopropylmalate/(R)-2-methylmalate dehydratase small subunit
KO:K01706	1.20	0.01	0.00	0.48	0.00	0.00	0.00	8.10E-02	3.95E-01	1.00E+00	2.08E-01	1.00E+00	1.00E+00	1.00E+00	glucarate dehydratase [EC:4.2.1.4]
KO:K01707	0.00	0.06	0.00	0.05	0.00	0.00	0.00	1.00E+00	1.47E-01	1.00E+00	1.88E-01	1.00E+00	1.00E+00	1.00E+00	5-dehydro-4-deoxyglucarate dehydratase [EC:4.2.1.41]
KO:K01708	0.07	5.33	0.20	6.40	0.00	0.00	0.00	7.59E-01	9.21E-02	7.33E-01	6.77E-02	1.00E+00	1.00E+00	1.00E+00	galactarate dehydratase [EC:4.2.1.42]
KO:K01709	0.92	0.04	2.45	1.52	1.66	0.00	2.36	4.42E-01	8.54E-01	1.39E-01	2.63E-01	2.37E-01	1.00E+00	1.48E-01	CDP-glucose 4,6-dehydratase [EC:4.2.1.45]
KO:K01710	3.32	3.21	10.69	5.88	13.64	3.36	2.20	8.40E-01	8.54E-01	3.99E-02	3.53E-01	1.24E-02	8.34E-01	9.34E-01	dTDP-glucose 4,6-dehydratase [EC:4.2.1.46]
KO:K01711	2.42	5.07	14.11	3.28	7.80	0.00	2.60	7.81E-01	2.94E-01	1.50E-02	6.27E-01	9.69E-02	1.00E+00	7.55E-01	GDPmannose 4,6-dehydratase [EC:4.2.1.47]
KO:K01712	2.06	5.56	0.01	3.17	0.00	1.47	0.00	3.13E-01	4.94E-02	9.55E-01	1.45E-01	1.00E+00	4.90E-01	1.00E+00	urocanate hydratase [EC:4.2.1.49]
KO:K01713	0.70	0.00	0.00	0.00	0.00	0.00	0.00	3.14E-02	1.00E+00	1.00E+00	1.00E+00	1.00E+00	1.00E+00	1.00E+00	prephenate dehydratase [EC:4.2.1.51]
KO:K01714	6.41	12.75	13.42	10.73	6.57	1.63	1.95	6.89E-01	6.61E-02	5.50E-02	1.40E-01	6.65E-01	9.86E-01	9.81E-01	dihydrodipicolinate synthase [EC:4.2.1.52]
KO:K01715	1.33	0.97	0.00	0.64	0.00	1.34	2.77	2.78E-01	3.87E-01	1.00E+00	5.13E-01	1.00E+00	2.73E-01	1.13E-01	3-hydroxybutyryl-CoA dehydratase [EC:4.2.1.55]
KO:K01716	11.52	5.53	0.17	9.33	0.00	0.00	2.30	3.59E-02	2.44E-01	9.68E-01	6.50E-02	1.00E+00	1.00E+00	7.82E-01	3-hydroxydecanoyl-[acyl-carrier-protein] dehydratase [EC:4.2.1.6]
KO:K01718	0.02	0.00	0.00	0.00	11.96	0.00	0.00	5.15E-01	1.00E+00	1.00E+00	5.74E-01	1.43E-04	1.00E+00	1.00E+00	pseudouridylate synthase [EC:4.2.1.7]
KO:K01719	2.84	1.63	5.35	2.41	1.21	6.60	0.49	3.76E-01	6.57E-01	1.14E-01	4.64E-01	7.65E-01	7.27E-02	8.90E-01	uroporphyrinogen-III synthase [EC:4.2.1.75]
KO:K01720	0.38	0.44	2.76	2.69	0.11	5.94	0.03	6.54E-01	6.36E-01	1.68E-01	1.74E-01	7.59E-01	6.16E-02	8.13E-01	2-methylcitrate dehydratase [EC:4.2.1.79]
KO:K01721	0.08	22.63	0.00	1.56	0.00	0.00	0.00	8.55E-01	3.71E-03	1.00E+00	4.54E-01	1.00E+00	1.00E+00	1.00E+00	nitrile hydratase [EC:4.2.1.84]
KO:K01724	2.20	0.39	1.47	3.20	13.45	0.00	0.65	3.73E-01	9.05E-01	5.98E-01	1.84E-01	6.00E-03	1.00E+00	8.50E-01	4a-hydroxytetrahydrobiopterin dehydratase [EC:4.2.1.96]
KO:K01725	0.03	0.00	0.00	0.00	10.07	0.00	0.01	5.98E-01	6.88E-01	1.00E+00	6.72E-01	1.43E-04	1.00E+00	6.39E-01	cyanate lyase [EC:4.2.1.14]
KO:K01726	1.46	0.64	0.00	1.57	0.00	3.19	1.47	2.92E-01	5.06E-01	1.00E+00	2.74E-01	1.00E+00	1.16E-01	2.90E-01	
KO:K01728	0.00	0.00	2.68	0.30	0.00	0.00	0.56	1.00E+00	1.00E+00	4.76E-02	2.20E-01	1.00E+00	1.00E+00	1.80E-01	pectate lyase [EC:4.2.2.2]
KO:K01729	0.06	0.00	0.00	0.05	0.00	0.00	0.00	1.04E-01	1.00E+00	1.00E+00	2.10E-01	1.00E+00	1.00E+00	1.00E+00	poly(beta-D-mannuronate) lyase [EC:4.2.2.3]
KO:K01733	5.55	7.89	12.38	4.54	7.02	0.71	6.82	6.04E-01	2.17E-01	3.31E-02	7.85E-01	3.25E-01	9.92E-01	3.61E-01	threonine synthase [EC:4.2.3.1]
KO:K01734	0.54	0.00	0.00	0.42	0.37	0.00	0.00	1.29E-01	6.26E-01	1.00E+00	1.63E-01	1.86E-01	1.00E+00	1.00E+00	methylglyoxal synthase [EC:4.2.3.3]
KO:K01735	6.53	6.16	7.95	3.65	8.61	1.73	3.33	2.62E-01	3.21E-01	1.28E-01	8.24E-01	9.11E-02	9.63E-01	8.57E-01	3-dehydroquinate synthase [EC:4.2.3.4]
KO:K01736	10.45	5.87	12.44	5.87	10.34	0.42	6.79	8.59E-02	7.30E-01	3.64E-02	7.30E-01	9.07E-02	9.98E-01	5.40E-01	chorismate synthase [EC:4.2.3.5]
KO:K01737	2.91	0.25	5.63	1.25	18.08	0.99	2.08	3.55E-01	9.19E-01	1.00E-01	7.60E-01	5.14E-03	8.07E-01	5.47E-01	6-pyruvoyl tetrahydrobiopterin synthase [EC:4.2.3.12]
KO:K01738	3.34	10.12	4.43	4.56	14.83	1.62	0.18	7.48E-01	6.84E-02	5.57E-01	5.33E-01	1.76E-02	9.21E-01	9.85E-01	cysteine synthase A [EC:2.5.1.47]
KO:K01739	0.53	5.33	9.36	0.87	5.29	0.00	0.00	8.40E-01	6.77E-02	1.54E-02	7.55E-01	6.93E-02	1.00E+00	1.00E+00	cystathionine gamma-synthase [EC:2.5.1.48]
KO:K01740	6.96	8.06	2.68	10.69	8.11	2.44	0.27	3.09E-01	2.07E-01	8.78E-01	8.14E-02	2.04E-01	8.95E-01	9.88E-01	O-acetylhomoserine (thiol)-lyase [EC:2.5.1.49]
KO:K01743	0.00	0.67	0.00	0.00	0.00	0.00	0.00	1.00E+00	4.51E-02	1.00E+00	1.00E+00	1.00E+00	1.00E+00	1.00E+00	
KO:K01744	0.03	0.01	0.00	0.05	0.00	0.00	5.10	4.90E-01	5.37E-01	1.00E+00	4.32E-01	1.00E+00	1.00E+00	1.67E-02	aspartate ammonia-lyase [EC:4.3.1.1]
KO:K01745	0.99	1.26	0.00	1.69	0.00	5.40	0.00	3.70E-01	2.77E-01	1.00E+00	1.85E-01	1.00E+00	2.44E-02	1.00E+00	histidine ammonia-lyase [EC:4.3.1.3]

KO:K01749	3.52	5.09	8.52	1.51	8.27	1.39	3.13	5.40E-01	2.32E-01	5.03E-02	9.06E-01	5.64E-02	9.14E-01	6.33E-01	hydroxymethylbilane synthase [EC:2.5.1.61]
KO:K01750	0.99	36.28	0.09	2.37	0.00	0.00	0.10	8.43E-01	1.00E-03	9.38E-01	6.15E-01	9.61E-01	1.00E+00	9.37E-01	ornithine cyclodeaminase [EC:4.3.1.12]
KO:K01751	1.39	11.56	0.00	1.84	0.00	0.00	0.00	4.56E-01	1.26E-02	1.00E+00	3.49E-01	1.00E+00	1.00E+00	1.00E+00	diaminopropionate ammonia-lyase [EC:4.3.1.15]
KO:K01752	4.19	18.20	0.23	0.20	0.00	2.76	0.01	1.83E-01	4.71E-03	9.37E-01	9.45E-01	1.00E+00	3.82E-01	9.83E-01	L-serine dehydratase [EC:4.3.1.17]
KO:K01753	0.00	0.23	0.00	0.00	0.00	0.00	0.20	1.00E+00	1.40E-01	1.00E+00	1.00E+00	1.00E+00	1.00E+00	1.49E-01	D-serine dehydratase [EC:4.3.1.18]
KO:K01754	8.78	21.96	2.13	4.01	2.64	1.39	1.23	1.60E-01	4.86E-03	9.29E-01	7.77E-01	9.00E-01	9.61E-01	9.68E-01	threonine dehydratase [EC:4.3.1.19]
KO:K01755	4.88	8.90	8.87	3.98	9.56	1.04	4.15	6.41E-01	9.80E-02	9.89E-02	8.01E-01	7.60E-02	9.83E-01	7.77E-01	argininosuccinate lyase [EC:4.3.2.1]
KO:K01756	5.61	6.43	8.20	6.23	5.66	5.20	3.46	5.46E-01	3.63E-01	1.35E-01	4.06E-01	5.33E-01	6.47E-01	8.98E-01	adenylosuccinate lyase [EC:4.3.2.2]
KO:K01758	1.29	0.00	10.46	0.22	0.00	0.00	0.03	1.94E-01	6.87E-01	1.10E-02	4.94E-01	1.00E+00	1.00E+00	6.43E-01	cystathionine gamma-lyase [EC:4.4.1.1]
KO:K01759	4.22	7.62	4.64	4.47	6.22	1.19	0.33	4.49E-01	9.86E-02	3.68E-01	3.97E-01	1.74E-01	9.26E-01	9.77E-01	lactoylglutathione lyase [EC:4.4.1.5]
KO:K01760	2.56	3.69	0.00	8.88	0.00	3.51	0.00	3.98E-01	2.47E-01	1.00E+00	4.79E-02	1.00E+00	2.67E-01	1.00E+00	cystathionine beta-lyase [EC:4.4.1.8]
KO:K01761	1.46	1.03	4.10	0.98	0.00	0.00	0.00	2.28E-01	3.30E-01	5.67E-02	3.45E-01	1.00E+00	1.00E+00	1.00E+00	methionine-gamma-lyase [EC:4.4.1.11]
KO:K01763	0.00	0.00	0.00	0.00	0.00	0.00	0.83	1.00E+00	1.00E+00	1.00E+00	1.00E+00	1.00E+00	1.00E+00	2.80E-02	selenocysteine lyase [EC:4.4.1.16]
KO:K01766	2.19	0.24	0.00	0.10	0.00	0.00	0.00	8.14E-02	3.03E-01	1.00E+00	3.85E-01	1.00E+00	1.00E+00	1.00E+00	cysteine sulfinate desulfinase [EC:4.4.1.-]
KO:K01768	0.33	1.33	11.20	5.59	0.36	0.34	2.55	8.64E-01	6.27E-01	2.69E-02	1.03E-01	8.58E-01	8.63E-01	3.61E-01	adenylate cyclase [EC:4.6.1.1]
KO:K01769	0.72	0.00	0.00	0.01	0.00	0.00	0.00	6.79E-02	1.00E+00	1.00E+00	2.34E-01	1.00E+00	1.00E+00	1.00E+00	guanylate cyclase, other [EC:4.6.1.2]
KO:K01770	1.62	0.01	0.60	0.81	14.22	0.88	0.97	3.09E-01	9.41E-01	7.27E-01	6.36E-01	1.00E-03	6.01E-01	5.59E-01	2-C-methyl-D-erythritol 2,4-cyclodiphosphate synthase [EC:4.6.1.12]
KO:K01772	3.77	3.77	5.57	5.82	9.66	4.09	0.58	6.39E-01	6.39E-01	2.43E-01	2.11E-01	3.34E-02	5.56E-01	9.86E-01	ferrochelatase [EC:4.99.1.1]
KO:K01774	0.00	0.00	3.75	0.00	0.00	0.00	0.00	1.00E+00	1.00E+00	3.00E-03	1.00E+00	1.00E+00	1.00E+00	1.00E+00	
KO:K01775	3.02	5.94	4.76	2.98	1.37	0.99	2.12	5.23E-01	7.64E-02	1.52E-01	5.37E-01	9.11E-01	9.47E-01	7.91E-01	alanine racemase [EC:5.1.1.1]
KO:K01776	0.13	18.16	0.00	0.28	2.78	0.72	0.04	9.23E-01	2.29E-03	1.00E+00	8.80E-01	2.33E-01	7.69E-01	9.45E-01	glutamate racemase [EC:5.1.1.3]
KO:K01777	0.16	1.98	0.15	0.17	0.00	0.00	0.41	4.96E-01	8.30E-02	5.03E-01	4.83E-01	1.00E+00	1.00E+00	3.25E-01	proline racemase [EC:5.1.1.4]
KO:K01778	3.22	10.01	3.76	2.61	6.97	8.82	1.03	7.91E-01	3.71E-02	6.85E-01	8.74E-01	1.42E-01	6.19E-02	9.74E-01	diaminopimelate epimerase [EC:5.1.1.7]
KO:K01779	0.15	1.96	0.03	2.90	0.00	0.00	0.00	6.37E-01	1.22E-01	7.55E-01	7.01E-02	1.00E+00	1.00E+00	1.00E+00	aspartate racemase [EC:5.1.1.13]
KO:K01780	0.00	0.00	0.00	0.20	0.00	0.00	0.00	1.00E+00	1.00E+00	1.00E+00	1.43E-01	1.00E+00	1.00E+00	1.00E+00	
KO:K01781	2.26	4.77	0.00	2.11	0.00	0.00	0.00	2.06E-01	7.41E-02	1.00E+00	2.21E-01	1.00E+00	1.00E+00	1.00E+00	mandelate racemase [EC:5.1.2.2]
KO:K01782	4.31	27.23	0.10	0.45	0.26	0.03	0.46	2.76E-01	1.57E-03	9.52E-01	9.13E-01	9.33E-01	9.63E-01	9.12E-01	3-hydroxyacyl-CoA dehydrogenase / enoyl-CoA hydratase /
KO:K01783	5.12	4.64	4.99	6.26	7.01	3.11	2.22	4.24E-01	5.34E-01	4.49E-01	2.27E-01	1.51E-01	8.35E-01	9.23E-01	ribulose-phosphate 3-epimerase [EC:5.1.3.1]
KO:K01784	3.42	2.10	4.95	8.08	11.42	4.62	4.38	7.62E-01	9.02E-01	4.84E-01	1.25E-01	4.49E-02	5.45E-01	5.92E-01	UDP-glucose 4-epimerase [EC:5.1.3.2]
KO:K01785	1.33	1.76	10.56	0.73	0.68	0.00	0.60	4.82E-01	3.53E-01	7.71E-03	7.13E-01	7.27E-01	1.00E+00	7.59E-01	aldose 1-epimerase [EC:5.1.3.3]
KO:K01786	0.11	0.10	0.00	0.00	0.00	0.00	0.16	1.98E-01	2.04E-01	1.00E+00	1.00E+00	1.00E+00	1.00E+00	1.54E-01	L-ribulose-5-phosphate 4-epimerase [EC:5.1.3.4]
KO:K01787	0.23	0.25	3.74	0.00	0.00	0.00	0.00	2.70E-01	2.63E-01	3.54E-02	1.00E+00	1.00E+00	1.00E+00	1.00E+00	N-acylglucosamine 2-epimerase [EC:5.1.3.8]
KO:K01788	0.00	0.00	0.00	0.00	17.39	0.00	0.00	1.00E+00	5.40E-01	1.00E+00	1.00E+00	4.29E-04	1.00E+00	1.00E+00	N-acylglucosamine-6-phosphate 2-epimerase [EC:5.1.3.9]
KO:K01789	0.02	9.04	0.00	1.03	0.16	0.00	0.00	8.41E-01	1.40E-02	1.00E+00	3.46E-01	7.28E-01	1.00E+00	1.00E+00	UDP-glucuronate 5'-epimerase [EC:5.1.3.12]
KO:K01790	1.77	4.63	0.04	1.92	5.27	0.77	1.34	5.63E-01	8.64E-02	9.79E-01	5.15E-01	6.54E-02	8.56E-01	7.09E-01	dTDP-4-dehydrorhamnose 3,5-epimerase [EC:5.1.3.13]
KO:K01791	1.50	1.24	0.03	1.99	7.68	0.04	0.59	4.04E-01	4.82E-01	9.32E-01	2.86E-01	3.19E-02	9.17E-01	7.16E-01	UDP-N-acetylglucosamine 2-epimerase [EC:5.1.3.14]
KO:K01792	0.38	0.00	0.00	0.11	0.00	0.00	0.00	8.29E-02	1.00E+00	1.00E+00	2.20E-01	1.00E+00	1.00E+00	1.00E+00	glucose-6-phosphate 1-epimerase [EC:5.1.3.15]
KO:K01793	0.00	0.00	0.00	0.00	0.00	0.00	0.42	1.00E+00	1.00E+00	1.00E+00	1.00E+00	1.00E+00	1.00E+00	2.74E-02	heparosan-N-sulfate-glucuronate 5-epimerase [EC:5.1.3.17]
KO:K01795	2.85	0.68	9.28	2.23	14.17	3.91	1.09	5.35E-01	9.33E-01	3.24E-02	6.83E-01	7.57E-03	3.16E-01	8.87E-01	
KO:K01796	5.63	0.11	0.00	2.79	0.00	0.00	0.11	5.64E-02	7.13E-01	1.00E+00	1.47E-01	1.00E+00	1.00E+00	7.13E-01	alpha-methylacyl-CoA racemase [EC:5.1.99.4]
KO:K01797	2.40	7.23	0.00	3.02	0.00	0.00	0.00	2.51E-01	4.60E-02	1.00E+00	1.83E-01	1.00E+00	1.00E+00	1.00E+00	
KO:K01799	1.08	1.93	0.00	0.13	0.00	0.00	0.00	1.78E-01	9.76E-02	1.00E+00	4.54E-01	1.00E+00	1.00E+00	1.00E+00	maleate isomerase [EC:5.2.1.1]
KO:K01800	0.19	3.63	0.00	0.45	0.04	0.00	0.00	6.28E-01	1.76E-02	1.00E+00	3.88E-01	8.14E-01	1.00E+00	1.00E+00	maleylacetoacetate isomerase [EC:5.2.1.2]
KO:K01801	0.32	0.00	0.00	0.06	0.00	0.00	0.00	9.23E-02	1.00E+00	1.00E+00	2.32E-01	1.00E+00	1.00E+00	1.00E+00	maleylpyruvate isomerase [EC:5.2.1.4]
KO:K01802	3.39	10.01	1.95	5.70	7.21	5.57	0.57	7.53E-01	8.29E-02	9.02E-01	3.81E-01	2.11E-01	4.00E-01	9.75E-01	peptidylprolyl isomerase [EC:5.2.1.8]
KO:K01803	8.33	2.52	11.99	5.59	7.29	3.55	7.09	1.47E-01	9.50E-01	3.20E-02	5.76E-01	2.50E-01	8.89E-01	2.82E-01	triosephosphate isomerase (TIM) [EC:5.3.1.1]
KO:K01804	0.66	0.00	0.53	0.01	0.00	0.00	0.00	7.20E-02	5.41E-01	1.00E-01	4.90E-01	1.00E+00	1.00E+00	1.00E+00	L-arabinose isomerase [EC:5.3.1.4]
KO:K01805	0.33	2.42	21.00	0.45	4.00	0.00	0.82	8.49E-01	3.11E-01	2.43E-03	8.20E-01	1.36E-01	1.00E+00	7.20E-01	xylose isomerase [EC:5.3.1.5]
KO:K01807	2.46	5.36	0.49	2.34	9.48	0.52	2.40	6.00E-01	1.18E-01	9.48E-01	6.29E-01	2.53E-02	9.45E-01	6.16E-01	ribose 5-phosphate isomerase A [EC:5.3.1.6]
KO:K01808	1.04	0.19	12.64	2.49	2.74	0.13	0.05	5.99E-01	8.65E-01	8.14E-03	2.37E-01	2.03E-01	8.85E-01	9.07E-01	ribose 5-phosphate isomerase B [EC:5.3.1.6]
KO:K01809	2.66	4.97	7.14	2.17	0.08	4.37	0.07	4.44E-01	1.37E-01	5.83E-02	5.59E-01	9.58E-01	1.82E-01	9.60E-01	mannose-6-phosphate isomerase [EC:5.3.1.8]
KO:K01810	3.55	7.06	5.55	4.16	9.06	2.29	0.71	6.96E-01	1.02E-01	2.41E-01	5.43E-01	3.84E-02	8.96E-01	9.82E-01	glucose-6-phosphate isomerase [EC:5.3.1.9]
KO:K01811	1.07	0.00	0.00	0.01	0.00	0.00	0.00	2.36E-02	1.00E+00	1.00E+00	4.88E-01	1.00E+00	1.00E+00	1.00E+00	putative family 31 glucosidase
KO:K01812	2.53	0.04	7.26	0.48	0.00	0.00	1.49	1.26E-01	8.49E-01	2.60E-02	5.65E-01	1.00E+00	1.00E+00	2.45E-01	glucuronate isomerase [EC:5.3.1.12]
KO:K01813	0.00	0.66	2.93	0.00	0.58	0.00	0.00	1.00E+00	1.65E-01	3.11E-02	1.00E+00	1.85E-01	1.00E+00	1.00E+00	L-rhamnose isomerase [EC:5.3.1.14]
KO:K01814	5.63	6.06	3.07	3.63	3.39	3.23	2.52	2.25E-01	1.80E-01	7.29E-01	6.05E-01	6.60E-01	6.97E-01	8.22E-01	phosphoribosylformimino-5-aminoimidazole carboxamide ribotide
KO:K01815	0.07	0.00	0.22	0.37	0.00	0.00	0.00	2.36E-01	1.00E+00	1.76E-01	1.47E-01	1.00E+00	1.00E+00	1.00E+00	4-deoxy-L-threo-5-hexosulose-uronate ketol-isomerase [EC:5.3.1.17]
KO:K01816	0.80	5.29	7.79	1.97	3.63	0.00	0.73	8.30E-01	1.05E-01	4.63E-02	5.45E-01	2.28E-01	1.00E+00	8.44E-01	hydroxypyruvate isomerase [EC:5.3.1.22]
KO:K01817	3.80	2.40	5.75	3.60	3.28	0.30	4.30	3.43E-01	6.90E-01	1.10E-01	3.82E-01	4.61E-01	9.80E-01	2.50E-01	phosphoribosylanthranilate isomerase [EC:5.3.1.24]
KO:K01818	0.00	0.00	11.13	0.00	0.00	0.00	0.00	2.45E-01	1.00E+00	1.51E-02	1.00E+00	1.00E+00	1.00E+00	1.00E+00	L-fucose isomerase [EC:5.3.1.25]
KO:K01820	0.08	0.00	10.59	0.01	8.12	0.00	3.09	6.26E-01	1.00E+00	1.57E-02	6.58E-01	2.69E-02	1.00E+00	1.07E-01	
KO:K01821	0.00	0.12	0.00	0.53	1.62	0.00	0.32	5.55E-01	3.69E-01	1.00E+00	2.15E-01	7.66E-02	1.00E+00	2.71E-01	4-oxalocrotonate tautomerase [EC:5.3.2.-]
KO:K01822	3.03	0.00	0.00	0.97	0.00	0.00	0.08	5.31E-02	1.00E+00	1.00E+00	2.01E-01	1.00E+00	1.00E+00	5.58E-01	steroid delta-isomerase [EC:5.3.3.1]
KO:K01823	8.72	10.01	0.00	1.37	0.00	0.04	0.32	2.97E-02	2.09E-02	1.00E+00	7.29E-01	1.00E+00	9.58E-01	9.21E-01	isopentenyl-diphosphate delta-isomerase [EC:5.3.3.2]
KO:K01825	8.97	0.35	8.69	0.60	0.00	0.00	0.92	3.74E-02	7.93E-01	4.10E-02	7.30E-01	1.00E+00	1.00E+00	6.52E-01	3-hydroxyacyl-CoA dehydrogenase / enoyl-CoA hydratase /
KO:K01826	0.70	0.68	0.00	1.45	0.00	0.00	0.00	2.27E-01	2.32E-01	1.00E+00	1.14E-01	1.00E+00	1.00E+00	1.00E+00	5-carboxymethyl-2-hydroxymuconate isomerase [EC:5.3.3.1]
KO:K01828	2.63	10.90	0.01	2.61	0.00	0.00	0.75	3.28E-01	1.73E-02	9.45E-01	3.31E-01	1.00E+00	1.00E+00	7.97E-01	
KO:K01829	0.03	0.00	0.49	0.01	0.22	0.00	1.56	4.09E-01	1.00E+00	1.29E-01	5.05E-01	2.26E-01	1.00E+00	3.84E-02	protein disulfide-isomerase [EC:5.3.4.1]
KO:K01834	6.27	7.34	4.13	5.81	13.81	0.99	5.46	4.66E-01	2.56E-01	8.67E-01	5.74E-01	1.23E-02	9.96E-01	6.54E-01	phosphoglycerate mutase [EC:5.4.2.1]
KO:K01835	0.40	1.32	0.00	0.23	9.49	2.55	0.12	6.72E-01	2.78E-01	1.00E+00	7.70E-01	8.43E-03	1.08E-01	8.26E-01	phosphoglucomutase [EC:5.4.2.2]
KO:K01838	0.64	0.47	0.00	0.74	3.04	1.54	0.00	3.99E-01	4.85E-01	1.00E+00	3.55E-01	7.01E-02	1.73E-01	1.00E+00	beta-phosphoglucomutase [EC:5.4.2.6]
KO:K01839	0.03	6.32	0.08	0.18	0.00	1.23	0.00	8.76E-01	4.71E-03	8.44E-01	7.64E-01	1.00E+00	1.77E-01	1.00E+00	phosphopentomutase [EC:5.4.2.7]
KO:K01840	8.00	1.43	10.43	6.53	5.70	0.29	5.17	1.24E-01	9.32E-01	5.44E-02	2.18E-01	3.23E-01	9.85E-01	4.11E-01	phosphomannomutase [EC:5.4.2.8]
KO:K01841	0.46	0.55	0.00	0.02	0.00	0.00	0.75	2.23E-01	2.03E-01	1.00E+00	4.07E-01	1.00E+00	1.00E+00	1.50E-01	phosphoenolpyruvate phosphomutase [EC:5.4.2.9]
KO:K01842	1.50	0.38	0.23	0.19	0.00	11.97	0.00	2.32E-01	5.10E-01	5.84E-01	6.02E-01	1.00E+00	1.03E-02	1.00E+00	
KO:K01843	0.50	0.04	19.30	0.06	2.64	0.35	0.41	5.42E-01	8.28E-01	1.57E-03	8.05E-01	1.27E-01	6.20E-01	5.85E-01	lysine 2,3-aminomutase [EC:5.4.3.2]
KO:K01845	4.09	1.74	8.97	3.16	11.31	2.75	6.38	5.25E-01	9.02E-01	6.51E-02	7.07E-01	3.03E-02	7.81E-01	1.80E-01	glutamate-1-semialdehyde 2,1-aminomutase [EC:5.4.3.8]
KO:K01846	0.02	0.20	0.00	0.02	0.00	0.00	0.00	2.29E-01	1.31E-01	1.00E+00	2.73E-01	1.00E+00	1.00E+00	1.00E+00	methylaspartate mutase [EC:5.4.99.1]
KO:K01847	7.88	25.33	0.48	3.16	0.00	0.18	0.00	1.06E-01	1.71E-03	9.60E-01	6.55E-01	1.00E+00	9.73E-01	1.00E+00	methylmalonyl-CoA mutase [EC:5.4.99.2]
KO:K01848	0.53	0.00	0.00	1.10	0.00	0.00	2.04	2.74E-01	1.00E+00	1.00E+00	1.65E-01	1.00E+00	1.00E+00	8.74E-02	methylmalonyl-CoA mutase, N-terminal domain [EC:5.4.99.2]
KO:K01849	0.33	0.00	0.00	1.36	0.00	0.00	3.73	3.80E-01	1.00E+00	1.00E+00	1.67E-01	1.00E+00	1.00E+00	5.39E-02	methylmalonyl-CoA mutase, C-terminal domain [EC:5.4.99.2]
KO:K01850	0.18	0.00	0.00	2.22	0.00	0.00	0.00	3.44E-01	1.00E+00	1.00E+00	3.79E-02	1.00E+00	1.00E+00	1.00E+00	chorismate mutase [EC:5.4.99.5]
KO:K01854	0.02	0.03	3.40	0.46	0.00	0.00	0.93	6.57E-01	6.34E-01	3.20E-02	2.50E-01	1.00E+00	1.00E+00	1.52E-01	UDP-galactopyranose mutase [EC:5.4.99.9]
KO:K01856	0.49	3.75	0.68	2.90	0.00	0.00	0.01	6.35E-01	7.24E-02	5.47E-01	1.09E-01	1.00E+00	1.00E+00	9.07E-01	muconate cycloisomerase [EC:5.5.1.1]
KO:K01857	0.07	5.97	0.00	0.11	0.00	0.00	0.02	7.18E-01	1.43E-02	1.00E+00	6.63E-01	1.00E+00	1.00E+00	8.03E-01	3-carboxy-cis,cis-muconate cycloisomerase [EC:5.5.1.2]
KO:K01858	0.09	0.00	0.93	0.69	0.93	0.00	2.16	5.27E-01	1.00E+00	2.20E-01	2.63E-01	2.20E-01	1.00E+00	1.03E-01	myo-inositol-1-phosphate synthase [EC:5.5.1.4]
KO:K01860	0.00	0.00	0.00	0.03	8.68	0.00	0.00	4.01E-01	1.00E+00	1.00E+00	3.22E-01	4.71E-03	1.00E+00	1.00E+00	chloromuconate cycloisomerase [EC:5.5.1.7]
KO:K01861	0.00	0.00	0.00	0.00	10.63	0.00	0.00	1.00E+00	1.00E+00	1.00E+00	1.00E+00	1.86E-03	1.00E+00	1.00E+00	
KO:K01865	0.04	0.00	15.54	0.02	4.78	0.00	0.00	6.05E-01	7.11E-01	5.86E-03	6.61E-01	4.06E-02	1.00E+00	1.00E+00	
KO:K01866	12.94	6.78	5.81	4.51	3.66	4.59	2.34	1.89E-02	3.45E-01	5.50E-01	7.99E-01	8.90E-01	7.90E-01	9.56E-01	tyrosyl-tRNA synthetase [EC:6.1.1.1]
KO:K01867	7.27	6.70	6.96	6.10	5.65	2.84	5.54	2.71E-01	3.67E-01	3.22E-01	5.00E-01	5.96E-01	9.45E-01	6.24E-01	tryptophanyl-tRNA synthetase [EC:6.1.1.2]
KO:K01868	12.72	9.51	8.30	8.29	6.14	9.34	4.79	7.46E-02	3.58E-01	5.99E-01	6.00E-01	8.86E-01	3.93E-01	9.50E-01	threonyl-tRNA synthetase [EC:6.1.1.3]
KO:K01869	9.65	8.83	10.47	7.04	6.77	3.83	4.85	1.65E-01	2.58E-01	1.05E-01	6.27E-01	6.84E-01	9.60E-01	9.19E-01	leucyl-tRNA synthetase [EC:6.1.1.4]
KO:K01870	11.11	13.18	8.84	7.39	6.11	6.69	4.80	1.42E-01	4.74E-02	4.86E-01	7.77E-01	9.06E-01	8.61E-01	9.60E-01	isoleucyl-tRNA synthetase [EC:6.1.1.5]
KO:K01872	11.41	14.60	8.35	9.29	6.83	5.65	4.81	1.50E-01	2.31E-02	7.54E-01	5.46E-01	9.11E-01	9.60E-01	9.76E-01	alanyl-tRNA synthetase [EC:6.1.1.7]
KO:K01873	9.39	9.62	9.73	6.04	6.01	10.41	4.87	2.09E-01	1.80E-01	1.66E-01	8.58E-01	8.61E-01	1.14E-01	9.39E-01	valyl-tRNA synthetase [EC:6.1.1.9]

KO:K01874	8.27	6.92	8.62	6.23	4.72	1.75	3.83	1.58E-01	3.20E-01	1.32E-01	4.53E-01	7.74E-01	9.77E-01	8.77E-01	methionyl-tRNA synthetase [EC:6.1.1.1]
KO:K01875	8.14	6.20	9.94	6.31	5.42	2.90	2.57	1.46E-01	4.48E-01	6.00E-02	4.20E-01	6.41E-01	9.52E-01	9.64E-01	seryl-tRNA synthetase [EC:6.1.1.11]
KO:K01876	11.10	11.03	10.25	7.32	4.47	6.60	7.41	1.54E-01	1.60E-01	2.40E-01	7.57E-01	9.60E-01	8.47E-01	7.46E-01	aspartyl-tRNA synthetase [EC:6.1.1.12]
KO:K01878	6.90	8.63	0.06	3.14	4.04	1.79	2.27	1.33E-01	6.81E-02	9.86E-01	6.62E-01	4.69E-01	8.68E-01	8.11E-01	glycyl-tRNA synthetase alpha chain [EC:6.1.1.14]
KO:K01879	5.42	12.49	0.27	4.96	6.89	0.39	2.16	3.47E-01	2.27E-02	9.89E-01	4.33E-01	1.78E-01	9.86E-01	9.06E-01	glycyl-tRNA synthetase beta chain [EC:6.1.1.14]
KO:K01880	1.99	0.63	23.53	0.68	1.31	1.04	1.91	4.69E-01	8.05E-01	2.14E-03	7.92E-01	6.48E-01	7.14E-01	4.88E-01	glycyl-tRNA synthetase [EC:6.1.1.14]
KO:K01881	8.37	8.48	7.61	7.89	4.72	5.28	5.18	2.73E-01	2.56E-01	4.12E-01	3.57E-01	9.01E-01	8.52E-01	8.62E-01	prolyl-tRNA synthetase [EC:6.1.1.15]
KO:K01883	7.23	7.99	4.73	4.97	2.69	7.54	4.35	2.06E-01	1.33E-01	7.55E-01	7.03E-01	9.55E-01	1.73E-01	8.23E-01	cysteinyl-tRNA synthetase [EC:6.1.1.16]
KO:K01885	8.84	16.99	4.32	4.41	5.45	2.99	2.95	1.92E-01	6.86E-03	8.95E-01	8.89E-01	7.65E-01	9.57E-01	9.58E-01	glutamyl-tRNA synthetase [EC:6.1.1.17]
KO:K01886	12.35	1.04	5.52	3.55	2.29	0.17	1.48	2.30E-02	8.92E-01	1.92E-01	4.65E-01	7.14E-01	9.66E-01	8.45E-01	glutaminyl-tRNA synthetase [EC:6.1.1.18]
KO:K01887	7.14	13.37	5.98	4.94	2.99	3.26	4.33	3.42E-01	1.80E-02	6.00E-01	7.94E-01	9.47E-01	9.36E-01	8.63E-01	arginyl-tRNA synthetase [EC:6.1.1.19]
KO:K01889	8.90	7.42	7.25	5.41	4.38	6.87	2.89	1.53E-01	3.09E-01	3.35E-01	6.75E-01	8.28E-01	3.95E-01	9.34E-01	phenylalanyl-tRNA synthetase alpha chain [EC:6.1.1.2]
KO:K01890	11.94	10.92	11.80	6.23	6.43	6.60	2.79	7.17E-02	1.23E-01	7.79E-02	8.54E-01	8.32E-01	8.16E-01	9.88E-01	phenylalanyl-tRNA synthetase beta chain [EC:6.1.1.2]
KO:K01892	8.38	9.54	8.67	3.77	6.02	2.93	2.21	1.53E-01	8.91E-02	1.33E-01	8.66E-01	4.86E-01	9.25E-01	9.58E-01	histidyl-tRNA synthetase [EC:6.1.1.21]
KO:K01893	6.31	1.31	4.05	1.10	0.15	3.63	0.37	7.04E-02	6.51E-01	1.75E-01	7.05E-01	8.96E-01	2.09E-01	8.65E-01	asparaginyl-tRNA synthetase [EC:6.1.1.22]
KO:K01894	5.57	1.94	1.31	0.30	0.00	1.99	0.06	1.84E-02	2.30E-01	4.51E-01	8.87E-01	1.00E+00	2.20E-01	9.51E-01	glutamyl-Q tRNA(Asp) synthetase [EC:6.1.1.-]
KO:K01895	9.55	15.71	8.56	13.70	7.53	2.05	2.10	3.88E-01	2.60E-02	5.76E-01	5.80E-02	7.45E-01	9.93E-01	9.92E-01	acetyl-CoA synthetase [EC:6.2.1.1]
KO:K01897	10.11	21.42	1.26	10.72	3.12	3.46	0.29	1.86E-01	1.06E-02	9.79E-01	1.51E-01	9.13E-01	8.96E-01	9.95E-01	long-chain acyl-CoA synthetase [EC:6.2.1.3]
KO:K01899	0.70	0.00	0.00	0.00	0.00	0.00	0.00	6.29E-02	1.00E+00	1.00E+00	1.00E+00	1.00E+00	1.00E+00	1.00E+00	succinyl-CoA synthetase alpha subunit [EC:6.2.1.4 6.2.1.5]
KO:K01902	10.70	11.33	11.77	7.17	0.61	8.94	2.73	1.23E-01	9.51E-02	7.93E-02	5.54E-01	9.96E-01	2.70E-01	9.65E-01	succinyl-CoA synthetase alpha subunit [EC:6.2.1.5]
KO:K01903	12.46	17.89	12.49	9.79	0.62	2.47	3.52	9.44E-02	9.86E-03	9.27E-02	3.22E-01	9.98E-01	9.88E-01	9.72E-01	succinyl-CoA synthetase beta subunit [EC:6.2.1.5]
KO:K01904	0.00	8.09	0.00	0.19	0.00	0.00	0.00	1.00E+00	2.66E-02	1.00E+00	4.62E-01	1.00E+00	1.00E+00	1.00E+00	4-coumarate--CoA ligase [EC:6.2.1.12]
KO:K01906	0.02	0.00	0.00	2.37	0.00	0.00	0.00	3.71E-01	4.28E-01	1.00E+00	4.86E-02	1.00E+00	1.00E+00	1.00E+00	6-carboxyhexanoate--CoA ligase [EC:6.2.1.14]
KO:K01907	4.31	0.83	0.24	6.12	0.00	1.70	0.14	1.35E-01	7.26E-01	8.66E-01	6.54E-02	1.00E+00	4.75E-01	8.84E-01	acetoacetyl-CoA synthetase [EC:6.2.1.16]
KO:K01908	4.72	16.87	0.87	5.58	0.00	6.16	0.00	3.94E-01	1.03E-02	9.44E-01	2.70E-01	1.00E+00	2.17E-01	1.00E+00	propionyl-CoA synthetase [EC:6.2.1.17]
KO:K01909	0.00	0.00	0.00	0.10	0.00	0.00	0.00	1.00E+00	1.00E+00	1.00E+00	1.43E-01	1.00E+00	1.00E+00	1.00E+00	long-chain-fatty-acid--[acyl-carrier-protein] ligase [EC:6.2.1.2]
KO:K01911	1.23	0.13	0.62	0.98	4.09	0.09	0.00	2.34E-01	7.65E-01	4.43E-01	2.97E-01	4.17E-02	8.03E-01	1.00E+00	O-succinylbenzoic acid--CoA ligase [EC:6.2.1.26]
KO:K01912	2.06	15.55	1.91	0.44	1.29	0.00	1.39	6.04E-01	8.14E-03	6.36E-01	8.96E-01	7.62E-01	1.00E+00	7.44E-01	phenylacetate-CoA ligase [EC:6.2.1.3]
KO:K01913	7.21	0.21	0.00	0.56	0.00	0.00	0.00	3.81E-02	4.96E-01	1.00E+00	3.91E-01	1.00E+00	1.00E+00	1.00E+00	
KO:K01915	11.85	22.55	11.61	11.05	7.42	2.66	6.01	3.05E-01	4.71E-03	3.40E-01	4.24E-01	8.93E-01	9.96E-01	9.48E-01	glutamine synthetase [EC:6.3.1.2]
KO:K01916	0.72	7.78	6.83	2.53	1.71	4.13	0.08	8.78E-01	6.43E-02	9.09E-02	5.28E-01	7.12E-01	2.61E-01	9.51E-01	NAD+ synthase [EC:6.3.1.5]
KO:K01917	0.01	0.00	0.00	1.83	0.00	0.00	0.00	4.57E-01	1.00E+00	1.00E+00	4.01E-02	1.00E+00	1.00E+00	1.00E+00	glutathionylspermidine synthase [EC:6.3.1.8]
KO:K01918	2.72	1.49	5.44	2.22	2.35	0.28	0.39	2.25E-01	6.49E-01	3.36E-02	3.60E-01	3.19E-01	9.70E-01	9.54E-01	pantoate--beta-alanine ligase [EC:6.3.2.1]
KO:K01919	5.48	12.72	0.21	3.50	0.76	4.19	1.12	2.23E-01	1.21E-02	9.88E-01	5.99E-01	9.64E-01	4.47E-01	9.44E-01	glutamate--cysteine ligase [EC:6.3.2.2]
KO:K01920	3.47	7.04	0.15	5.38	6.33	0.00	2.01	4.57E-01	6.49E-02	9.88E-01	1.45E-01	8.76E-02	1.00E+00	8.19E-01	glutathione synthase [EC:6.3.2.3]
KO:K01921	4.81	11.14	7.28	2.54	6.48	1.06	0.94	4.55E-01	2.90E-02	1.38E-01	8.60E-01	2.01E-01	9.65E-01	9.70E-01	D-alanine-D-alanine ligase [EC:6.3.2.4]
KO:K01922	2.84	5.24	0.09	2.32	0.60	7.24	1.60	4.14E-01	1.26E-01	9.51E-01	5.37E-01	8.92E-01	5.70E-02	7.20E-01	phosphopantothenate-cysteine ligase [EC:6.3.2.5]
KO:K01923	5.31	11.61	2.65	4.96	6.29	0.34	2.24	4.19E-01	2.59E-02	8.93E-01	4.99E-01	2.46E-01	9.96E-01	9.24E-01	phosphoribosylaminoimidazole-succinocarboxamide synthase
KO:K01924	6.70	7.36	0.34	3.45	2.59	3.80	2.14	1.01E-01	7.74E-02	9.91E-01	6.25E-01	8.16E-01	5.30E-01	8.74E-01	UDP-N-acetylmuramate--alanine ligase [EC:6.3.2.8]
KO:K01925	5.83	8.15	4.63	2.84	4.22	0.80	1.04	1.54E-01	4.80E-02	3.44E-01	7.89E-01	4.43E-01	9.80E-01	9.71E-01	UDP-N-acetylmuramoylalanine--D-glutamate ligase [EC:6.3.2.9]
KO:K01926	0.01	0.00	1.04	0.00	4.68	0.00	2.60	5.07E-01	1.00E+00	1.43E-01	1.00E+00	4.43E-02	1.00E+00	6.91E-02	AT-rich DNA-binding protein
KO:K01928	6.23	4.67	10.85	1.64	3.21	1.67	0.34	9.76E-02	2.30E-01	1.44E-02	9.01E-01	5.63E-01	8.96E-01	9.91E-01	UDP-N-acetylmuramoylalanyl-D-glutamate--2,6-diaminopimelate ligase
KO:K01929	4.23	3.97	4.80	3.34	2.91	3.80	1.22	2.83E-01	3.43E-01	1.88E-01	5.22E-01	6.65E-01	3.89E-01	9.49E-01	UDP-N-acetylmuramoylalanyl-D-glutamyl-2,6-diaminopimelate--D-alanyl-
KO:K01930	0.13	0.04	6.48	0.25	1.16	2.45	0.02	5.84E-01	6.92E-01	2.66E-02	5.09E-01	2.15E-01	9.41E-02	7.41E-01	folylpolyglutamate synthase [EC:6.3.2.17]
KO:K01932	0.88	1.74	0.00	0.17	0.00	0.00	0.00	1.83E-01	9.64E-02	1.00E+00	4.52E-01	1.00E+00	1.00E+00	1.00E+00	
KO:K01933	7.41	9.84	5.30	4.57	8.75	3.43	3.99	3.01E-01	9.11E-02	7.04E-01	8.15E-01	1.53E-01	9.14E-01	8.76E-01	phosphoribosylformylglycinamidine cyclo-ligase [EC:6.3.3.1]
KO:K01934	1.96	3.26	1.11	1.86	0.36	0.64	1.57	3.75E-01	1.28E-01	7.00E-01	4.11E-01	9.10E-01	8.43E-01	5.16E-01	5-formyltetrahydrofolate cyclo-ligase [EC:6.3.3.2]
KO:K01935	0.23	0.04	0.00	0.10	6.62	0.00	1.65	5.97E-01	8.33E-01	1.00E+00	7.81E-01	8.57E-04	1.00E+00	4.70E-02	dethiobiotin synthetase [EC:6.3.3.3]
KO:K01937	10.10	9.40	10.99	8.95	5.87	3.50	7.63	2.22E-01	3.19E-01	1.40E-01	4.02E-01	8.91E-01	9.76E-01	6.70E-01	CTP synthase [EC:6.3.4.2]
KO:K01938	2.83	30.88	0.00	8.16	0.00	0.36	0.00	7.21E-01	1.14E-03	9.69E-01	1.37E-01	1.00E+00	9.43E-01	1.00E+00	formate--tetrahydrofolate ligase [EC:6.3.4.3]
KO:K01939	9.82	12.39	11.00	6.25	6.90	2.48	5.49	1.84E-01	5.16E-02	1.01E-01	8.34E-01	7.39E-01	9.92E-01	9.02E-01	adenylosuccinate synthase [EC:6.3.4.4]
KO:K01940	6.68	7.29	10.68	4.97	8.97	2.38	9.25	5.10E-01	4.02E-01	9.50E-02	8.05E-01	1.91E-01	9.67E-01	1.67E-01	argininosuccinate synthase [EC:6.3.4.5]
KO:K01941	0.12	2.45	0.14	0.24	0.14	0.00	0.00	4.95E-01	7.90E-02	4.79E-01	4.06E-01	4.79E-01	1.00E+00	1.00E+00	urea carboxylase [EC:6.3.4.6]
KO:K01945	6.72	8.21	7.81	4.37	4.96	1.17	2.89	2.20E-01	9.41E-02	1.17E-01	7.16E-01	5.81E-01	9.88E-01	9.14E-01	phosphoribosylamine--glycine ligase [EC:6.3.4.13]
KO:K01946	0.00	0.00	0.00	0.20	0.00	0.00	0.00	1.00E+00	1.00E+00	1.00E+00	1.43E-01	1.00E+00	1.00E+00	1.00E+00	
KO:K01950	5.75	1.72	14.42	2.30	3.03	0.51	5.89	2.04E-01	8.34E-01	1.57E-02	7.57E-01	6.18E-01	9.44E-01	1.96E-01	NAD+ synthase (glutamine-hydrolysing) [EC:6.3.5.1]
KO:K01951	9.17	12.66	8.82	7.27	8.55	3.56	6.01	3.07E-01	5.36E-02	3.72E-01	6.91E-01	4.25E-01	9.77E-01	8.65E-01	GMP synthase (glutamine-hydrolysing) [EC:6.3.5.2]
KO:K01952	8.83	15.88	6.71	6.96	15.09	3.18	5.71	5.24E-01	2.50E-02	8.52E-01	8.24E-01	3.34E-02	9.83E-01	9.18E-01	phosphoribosylformylglycinamidine synthase [EC:6.3.5.3]
KO:K01953	2.69	0.31	6.00	3.28	10.48	6.53	4.15	5.80E-01	9.48E-01	1.30E-01	4.54E-01	3.01E-02	1.06E-01	3.01E-01	asparagine synthase (glutamine-hydrolysing) [EC:6.3.5.4]
KO:K01955	10.34	12.72	16.22	7.33	4.33	7.48	5.26	2.54E-01	6.86E-02	9.00E-03	8.38E-01	9.77E-01	8.18E-01	9.61E-01	carbamoyl-phosphate synthase large subunit [EC:6.3.5.5]
KO:K01956	9.18	8.12	14.77	6.70	8.31	0.84	4.43	1.65E-01	3.09E-01	1.36E-02	6.30E-01	2.77E-01	9.99E-01	9.30E-01	carbamoyl-phosphate synthase small subunit [EC:6.3.5.5]
KO:K01957	0.00	0.00	0.00	0.31	0.00	0.00	0.00	1.00E+00	1.00E+00	1.00E+00	1.43E-01	1.00E+00	1.00E+00	1.00E+00	glutamyl-tRNA (Gln) amidotransferase [EC:6.3.5.-]
KO:K01958	0.16	3.03	0.00	0.08	3.95	0.00	0.00	5.26E-01	9.30E-02	1.00E+00	5.79E-01	6.66E-02	1.00E+00	1.00E+00	pyruvate carboxylase [EC:6.4.1.1]
KO:K01959	0.59	0.22	7.55	1.89	0.00	0.24	0.95	5.30E-01	6.79E-01	3.87E-02	2.41E-01	1.00E+00	6.71E-01	4.19E-01	pyruvate carboxylase subunit A [EC:6.4.1.1]
KO:K01960	0.65	15.53	2.14	1.15	1.27	0.56	3.80	8.96E-01	1.11E-02	6.52E-01	8.31E-01	8.14E-01	9.03E-01	3.37E-01	pyruvate carboxylase subunit B [EC:6.4.1.1]
KO:K01961	7.56	7.98	9.49	4.83	5.48	9.04	1.54	2.72E-01	2.21E-01	1.03E-01	7.80E-01	6.68E-01	1.26E-01	9.86E-01	acetyl-CoA carboxylase, biotin carboxylase subunit [EC:6.4.1.2
KO:K01962	7.33	8.50	9.41	5.69	7.50	3.80	1.73	3.06E-01	1.79E-01	1.18E-01	6.11E-01	2.82E-01	8.77E-01	9.77E-01	acetyl-CoA carboxylase carboxyl transferase subunit alpha
KO:K01963	6.83	9.34	9.18	5.05	7.29	5.01	1.54	3.77E-01	1.09E-01	1.18E-01	7.38E-01	2.95E-01	7.43E-01	9.84E-01	acetyl-CoA carboxylase carboxyl transferase subunit beta
KO:K01965	0.98	30.66	0.00	3.64	0.00	0.00	0.00	8.45E-01	5.71E-04	1.00E+00	3.32E-01	1.00E+00	1.00E+00	1.00E+00	propionyl-CoA carboxylase alpha chain [EC:6.4.1.3]
KO:K01966	6.82	25.08	0.36	10.71	0.00	1.68	0.18	3.44E-01	3.00E-03	9.81E-01	9.09E-02	1.00E+00	9.39E-01	9.85E-01	propionyl-CoA carboxylase beta chain [EC:6.4.1.3]
KO:K01968	3.76	9.93	0.18	1.79	0.00	0.00	0.08	1.10E-01	8.71E-03	9.46E-01	4.81E-01	1.00E+00	1.00E+00	9.65E-01	3-methylcrotonyl-CoA carboxylase alpha subunit [EC:6.4.1.4]
KO:K01969	1.51	8.47	0.22	0.57	0.00	0.00	0.00	3.03E-01	1.21E-02	8.15E-01	6.71E-01	1.00E+00	1.00E+00	1.00E+00	3-methylcrotonyl-CoA carboxylase beta subunit [EC:6.4.1.4]
KO:K01970	0.07	0.08	2.49	0.02	0.00	1.06	0.18	5.74E-01	5.54E-01	1.84E-02	7.15E-01	1.00E+00	7.16E-02	4.08E-01	
KO:K01971	0.30	0.90	0.51	0.55	9.13	0.22	0.35	8.14E-01	4.43E-01	7.01E-01	6.72E-01	2.14E-03	8.56E-01	7.83E-01	DNA ligase (ATP) [EC:6.5.1.1]
KO:K01972	7.22	4.78	7.46	4.72	4.32	2.66	2.58	1.22E-01	5.41E-01	1.06E-01	5.56E-01	6.58E-01	9.15E-01	9.21E-01	DNA ligase (NAD+) [EC:6.5.1.2]
KO:K01974	0.03	0.00	0.10	0.00	0.00	0.00	2.19	3.59E-01	1.00E+00	2.54E-01	1.00E+00	1.00E+00	1.00E+00	1.64E-02	RNA 3'-terminal phosphate cyclase [EC:6.5.1.4]
KO:K01975	0.05	0.05	0.37	0.04	0.00	0.00	0.66	4.63E-01	4.75E-01	1.33E-01	5.03E-01	1.00E+00	1.00E+00	6.73E-02	2'-5' RNA ligase [EC:6.5.1.-]
KO:K01976	2.37	0.09	0.00	0.55	0.00	0.00	0.00	8.11E-02	4.08E-01	1.00E+00	2.47E-01	1.00E+00	1.00E+00	1.00E+00	
KO:K01989	0.00	0.32	0.00	0.17	0.00	0.00	0.00	1.00E+00	1.18E-01	1.00E+00	1.72E-01	1.00E+00	1.00E+00	1.00E+00	putative ABC transport system substrate-binding protein
KO:K01990	1.07	1.96	24.42	1.30	7.21	1.01	0.65	8.61E-01	6.95E-01	2.00E-03	8.25E-01	7.13E-02	8.69E-01	9.15E-01	ABC-2 type transport system ATP-binding protein
KO:K01991	0.77	2.06	8.07	1.48	4.90	2.85	0.04	8.19E-01	4.21E-01	2.39E-02	6.02E-01	7.47E-02	2.39E-01	9.72E-01	polysaccharide export outer membrane protein
KO:K01992	4.42	3.60	6.74	5.13	6.51	0.23	0.92	3.67E-01	5.32E-01	1.25E-01	2.53E-01	1.37E-01	9.83E-01	9.38E-01	ABC-2 type transport system permease protein
KO:K01993	0.00	0.00	7.01	0.02	0.00	0.00	0.00	1.00E+00	5.67E-01	2.86E-03	3.98E-01	1.00E+00	1.00E+00	1.00E+00	HlyD family secretion protein
KO:K01995	1.61	36.69	0.35	9.86	0.01	0.00	0.00	8.70E-01	2.86E-04	9.50E-01	1.04E-01	9.72E-01	1.00E+00	1.00E+00	branched-chain amino acid transport system ATP-binding protein
KO:K01996	1.52	34.23	1.22	9.52	0.00	0.17	0.01	8.77E-01	1.14E-03	9.01E-01	1.12E-01	9.85E-01	9.57E-01	9.73E-01	branched-chain amino acid transport system ATP-binding protein
KO:K01997	1.21	40.81	0.78	12.48	0.02	0.00	0.02	9.20E-01	1.43E-04	9.36E-01	7.29E-02	9.74E-01	1.00E+00	9.71E-01	branched-chain amino acid transport system permease protein
KO:K01998	1.74	40.90	0.49	10.91	0.03	0.00	0.02	8.89E-01	2.86E-04	9.54E-01	9.84E-02	9.78E-01	1.00E+00	9.81E-01	branched-chain amino acid transport system permease protein
KO:K01999	2.54	41.49	0.64	14.17	0.05	0.00	0.23	8.65E-01	2.86E-04	9.57E-01	6.17E-02	9.80E-01	1.00E+00	9.70E-01	branched-chain amino acid transport system substrate-binding protein
KO:K02000	1.85	18.09	0.00	4.29	0.00	0.44	0.01	5.96E-01	1.13E-02	1.00E+00	2.40E-01	1.00E+00	8.50E-01	9.30E-01	glycine betaine/proline transport system ATP-binding protein
KO:K02001	1.48	22.86	0.00	8.14	0.00	0.02	0.03	7.75E-01	5.71E-03	1.00E+00	9.51E-02	1.00E+00	9.37E-01	9.36E-01	glycine betaine/proline transport system permease protein
KO:K02002	2.04	29.23	0.00	7.13	0.00	0.00	0.05	7.23E-01	2.86E-03	1.00E+00	1.64E-01	1.00E+00	1.00E+00	9.29E-01	glycine betaine/proline transport system substrate-binding protein
KO:K02003	5.80	4.88	13.77	3.21	9.69	2.01	1.96	3.83E-01	5.71E-01	1.44E-02	8.62E-01	6.77E-02	9.44E-01	9.47E-01	
KO:K02004	8.14	7.92	6.85	4.91	14.59	2.20	0.65	2.05E-01	2.29E-01	3.81E-01	7.37E-01	1.50E-02	9.64E-01	9.94E-01	
KO:K02005	1.76	1.17	0.32	1.62	17.42	0.00	0.04	4.27E-01	6.03E-01	8.49E-01	4.63E-01	3.29E-03	1.00E+00	9.11E-01	HlyD family secretion protein
KO:K02006	0.19	0.77	0.00	0.02	10.92	0.33	0.00	6.09E-01	2.76E-01	1.00E+00	7.95E-01	3.57E-03	5.07E-01	1.00E+00	cobalt/nickel transport system ATP-binding protein
KO:K02007	0.00	0.22	0.00	0.00	0.00	0.00	0.00	1.00E+00	7.17E-02	1.00E+00	1.00E+00	1.00E+00	1.00E+00	1.00E+00	cobalt/nickel transport system permease protein

KO:K02008	0.01	0.30	0.00	0.00	21.43	0.00	0.00	7.26E-01	4.08E-01	1.00E+00	1.00E+00	0.00E+00	1.00E+00	1.00E+00	cobalt/nickel transport system permease protein
KO:K02010	1.71	14.95	0.01	2.16	4.16	0.41	0.36	7.29E-01	6.86E-03	9.88E-01	6.21E-01	2.39E-01	9.30E-01	9.35E-01	iron(III) transport system ATP-binding protein [EC:3.6.3.3]
KO:K02011	2.37	9.27	0.20	6.84	2.56	2.56	0.87	7.01E-01	6.67E-02	9.62E-01	1.35E-01	6.64E-01	6.64E-01	9.04E-01	iron(III) transport system permease protein
KO:K02012	2.26	9.74	2.09	5.10	2.16	3.48	0.99	7.42E-01	6.23E-02	7.69E-01	2.79E-01	7.58E-01	5.23E-01	8.98E-01	iron(III) transport system substrate-binding protein
KO:K02013	0.44	0.25	0.83	0.17	11.89	0.00	0.04	5.06E-01	6.60E-01	2.94E-01	7.25E-01	3.14E-03	1.00E+00	8.68E-01	iron complex transport system ATP-binding protein [EC:3.6.3.34]
KO:K02014	21.53	1.92	5.18	7.56	0.16	11.44	0.25	1.10E-02	8.94E-01	5.34E-01	2.60E-01	9.77E-01	9.40E-02	9.75E-01	iron complex outermembrane recepter protein
KO:K02015	1.53	2.30	0.90	1.08	0.00	1.26	0.00	2.28E-01	1.05E-01	4.69E-01	3.79E-01	1.00E+00	3.10E-01	1.00E+00	iron complex transport system permease protein
KO:K02016	0.54	0.60	0.31	0.22	0.45	2.22	0.67	4.19E-01	3.87E-01	6.26E-01	7.12E-01	4.96E-01	5.87E-02	3.44E-01	iron complex transport system substrate-binding protein
KO:K02017	1.89	3.22	0.02	1.35	0.00	0.00	0.03	1.03E-01	3.26E-02	9.53E-01	2.02E-01	1.00E+00	1.00E+00	9.53E-01	molybdate transport system ATP-binding protein [EC:3.6.3.29]
KO:K02018	1.97	2.39	0.52	2.16	0.00	0.08	0.39	1.88E-01	1.27E-01	7.56E-01	1.57E-01	1.00E+00	9.32E-01	8.18E-01	molybdate transport system permease protein
KO:K02019	0.10	1.05	0.00	0.10	0.00	0.00	0.92	4.62E-01	1.32E-01	1.00E+00	4.61E-01	1.00E+00	1.00E+00	1.49E-01	molybdate transport system regulatory protein
KO:K02020	0.78	0.58	0.79	1.16	0.00	0.00	0.35	2.74E-01	3.83E-01	2.67E-01	1.51E-01	1.00E+00	1.00E+00	5.92E-01	molybdate transport system substrate-binding protein
KO:K02021	0.03	0.87	0.00	2.71	2.35	0.00	0.36	7.73E-01	3.42E-01	1.00E+00	9.47E-02	1.14E-01	1.00E+00	5.48E-01	putative ABC transport system ATP-binding protein
KO:K02022	4.71	2.06	0.18	1.36	4.77	1.53	0.07	9.74E-02	3.84E-01	9.27E-01	5.86E-01	9.47E-02	5.29E-01	9.63E-01	
KO:K02023	1.11	29.43	0.00	6.94	3.09	0.00	0.00	8.67E-01	3.57E-03	9.68E-01	1.73E-01	6.13E-01	1.00E+00	1.00E+00	multiple sugar transport system ATP-binding protein
KO:K02024	0.00	0.00	0.00	0.10	0.00	0.00	0.00	1.00E+00	1.00E+00	1.00E+00	1.43E-01	1.00E+00	1.00E+00	1.00E+00	maltoporin
KO:K02025	0.82	32.27	0.00	8.57	1.05	0.00	0.00	8.88E-01	1.71E-03	9.67E-01	1.13E-01	8.73E-01	1.00E+00	1.00E+00	multiple sugar transport system permease protein
KO:K02026	1.24	22.35	0.00	8.83	0.59	0.00	0.00	8.22E-01	5.00E-03	9.69E-01	7.69E-02	8.97E-01	1.00E+00	1.00E+00	multiple sugar transport system permease protein
KO:K02027	1.80	38.59	0.00	9.33	2.29	0.00	0.00	8.64E-01	1.14E-03	1.00E+00	1.52E-01	8.24E-01	1.00E+00	1.00E+00	multiple sugar transport system substrate-binding protein
KO:K02028	1.06	22.21	0.02	1.18	1.51	0.00	0.00	7.25E-01	3.57E-03	9.39E-01	7.02E-01	6.26E-01	1.00E+00	9.50E-01	polar amino acid transport system ATP-binding protein [EC:3.6.3.21]
KO:K02029	1.46	38.87	0.01	2.05	0.00	0.00	0.00	7.73E-01	5.71E-04	9.58E-01	6.62E-01	1.00E+00	1.00E+00	9.64E-01	polar amino acid transport system permease protein
KO:K02030	1.60	40.93	0.00	3.89	3.03	0.00	0.00	8.40E-01	7.14E-04	1.00E+00	5.44E-01	6.64E-01	1.00E+00	9.78E-01	polar amino acid transport system substrate-binding protein
KO:K02031	1.85	10.38	2.49	3.82	0.00	0.68	0.95	6.43E-01	3.90E-02	5.07E-01	2.98E-01	1.00E+00	8.61E-01	8.17E-01	peptide/nickel transport system ATP-binding protein
KO:K02032	3.70	32.86	2.54	6.23	0.79	0.01	1.02	7.27E-01	1.29E-03	8.43E-01	3.85E-01	9.52E-01	9.93E-01	9.41E-01	peptide/nickel transport system ATP-binding protein
KO:K02033	6.47	38.73	0.97	7.77	0.50	1.64	0.36	5.15E-01	8.57E-04	9.65E-01	3.68E-01	9.78E-01	9.42E-01	9.83E-01	peptide/nickel transport system permease protein
KO:K02034	5.74	31.05	3.03	7.00	2.84	2.12	0.09	5.83E-01	1.86E-03	8.73E-01	4.27E-01	8.84E-01	9.22E-01	9.93E-01	peptide/nickel transport system permease protein
KO:K02035	5.70	44.43	6.26	9.64	0.24	0.17	0.66	7.21E-01	0.00E+00	6.61E-01	3.07E-01	9.91E-01	9.92E-01	9.83E-01	peptide/nickel transport system substrate-binding protein
KO:K02036	2.03	7.09	1.64	5.94	6.28	0.02	0.50	7.91E-01	6.44E-02	8.59E-01	1.05E-01	8.81E-02	9.92E-01	9.63E-01	phosphate transport system ATP-binding protein [EC:3.6.3.27]
KO:K02037	3.26	7.05	3.42	6.91	8.66	0.00	0.88	6.70E-01	1.24E-01	6.35E-01	1.32E-01	6.67E-02	1.00E+00	9.53E-01	phosphate transport system permease protein
KO:K02038	2.00	3.68	1.35	6.12	9.76	0.09	0.66	7.46E-01	3.26E-01	8.64E-01	8.40E-02	2.14E-02	9.86E-01	9.44E-01	phosphate transport system permease protein
KO:K02039	2.71	3.85	3.42	2.59	2.08	0.03	1.57	3.80E-01	1.90E-01	2.47E-01	4.14E-01	5.57E-01	9.88E-01	7.12E-01	phosphate transport system protein
KO:K02040	2.73	5.81	4.66	8.09	10.58	0.00	0.62	7.98E-01	2.31E-01	4.04E-01	8.80E-02	3.59E-02	1.00E+00	9.73E-01	phosphate transport system substrate-binding protein
KO:K02041	0.63	4.06	0.08	1.56	4.17	0.00	0.10	6.28E-01	1.03E-01	8.23E-01	3.64E-01	9.74E-02	1.00E+00	8.15E-01	phosphonate transport system ATP-binding protein
KO:K02042	0.67	5.34	0.00	3.95	6.56	0.00	0.21	7.48E-01	9.86E-02	1.00E+00	1.62E-01	6.66E-02	1.00E+00	8.58E-01	phosphonate transport system permease protein
KO:K02043	0.00	1.07	0.00	0.13	0.00	0.00	0.00	1.00E+00	2.63E-02	1.00E+00	2.93E-01	1.00E+00	1.00E+00	1.00E+00	GntR family transcriptional regulator, phosphonate transport system
KO:K02044	0.88	4.39	0.39	3.31	3.69	9.09	0.20	7.97E-01	1.89E-01	8.73E-01	3.02E-01	2.56E-01	4.61E-02	9.03E-01	phosphonate transport system substrate-binding protein
KO:K02045	0.01	0.01	0.71	0.04	0.00	0.00	0.08	5.02E-01	5.09E-01	3.17E-02	3.43E-01	1.00E+00	1.00E+00	2.66E-01	sulfate transport system ATP-binding protein [EC:3.6.3.25]
KO:K02046	0.06	0.05	0.00	0.00	0.00	0.00	0.00	1.24E-01	1.66E-01	1.00E+00	1.00E+00	1.00E+00	1.00E+00	1.00E+00	sulfate transport system permease protein
KO:K02047	0.00	0.22	0.00	0.00	0.00	0.00	0.00	1.00E+00	6.79E-02	1.00E+00	1.00E+00	1.00E+00	1.00E+00	1.00E+00	sulfate transport system permease protein
KO:K02049	1.27	22.97	0.46	4.48	0.27	0.02	0.39	8.21E-01	3.14E-03	9.14E-01	2.84E-01	9.33E-01	9.69E-01	9.22E-01	sulfonate/nitrate/taurine transport system ATP-binding protein
KO:K02050	1.56	34.20	0.09	3.76	0.09	0.00	0.04	8.11E-01	8.57E-04	9.58E-01	4.32E-01	9.58E-01	1.00E+00	9.67E-01	sulfonate/nitrate/taurine transport system permease protein
KO:K02051	2.56	36.24	0.47	6.47	0.79	0.00	0.16	8.07E-01	1.00E-03	9.56E-01	2.86E-01	9.37E-01	1.00E+00	9.71E-01	sulfonate/nitrate/taurine transport system substrate-binding protein
KO:K02052	1.95	37.64	0.00	3.88	0.00	0.00	0.00	7.43E-01	7.14E-04	1.00E+00	4.32E-01	1.00E+00	1.00E+00	9.52E-01	putative spermidine/putrescine transport system ATP-binding protein
KO:K02053	1.38	28.15	0.00	4.93	0.00	0.00	0.00	7.61E-01	2.71E-03	1.00E+00	2.27E-01	1.00E+00	1.00E+00	1.00E+00	putative spermidine/putrescine transport system permease protein
KO:K02054	1.20	35.23	0.00	4.25	0.00	0.00	0.00	8.10E-01	5.71E-04	1.00E+00	3.35E-01	1.00E+00	1.00E+00	1.00E+00	putative spermidine/putrescine transport system permease protein
KO:K02055	1.68	41.70	0.01	5.88	0.00	0.00	0.00	8.13E-01	2.86E-04	9.58E-01	2.83E-01	1.00E+00	1.00E+00	9.62E-01	putative spermidine/putrescine transport system substrate-binding
KO:K02056	1.51	39.32	0.61	3.30	0.00	0.00	0.03	8.31E-01	2.86E-04	9.14E-01	5.58E-01	9.66E-01	1.00E+00	9.60E-01	simple sugar transport system ATP-binding protein [EC:3.6.3.17]
KO:K02057	1.82	44.79	0.13	3.39	0.00	0.00	0.09	8.05E-01	1.43E-04	9.53E-01	5.81E-01	9.68E-01	1.00E+00	9.55E-01	simple sugar transport system permease protein
KO:K02058	1.67	47.31	0.00	4.77	0.00	0.00	0.04	8.34E-01	1.43E-04	1.00E+00	4.24E-01	9.54E-01	1.00E+00	9.50E-01	simple sugar transport system substrate-binding protein
KO:K02061	0.00	0.00	0.00	0.51	0.00	0.00	0.00	1.00E+00	1.00E+00	1.00E+00	1.43E-01	1.00E+00	1.00E+00	1.00E+00	putative sulfate transport system substrate-binding protein
KO:K02062	0.27	2.99	0.00	0.00	0.00	0.00	0.00	3.47E-01	1.09E-02	1.00E+00	1.00E+00	1.00E+00	1.00E+00	1.00E+00	thiamine transport system ATP-binding protein
KO:K02063	0.14	10.57	0.00	0.02	0.00	0.00	0.00	7.28E-01	3.00E-03	1.00E+00	8.31E-01	1.00E+00	1.00E+00	1.00E+00	thiamine transport system permease protein
KO:K02064	1.19	10.86	0.00	0.04	0.00	0.00	0.00	3.10E-01	2.09E-02	1.00E+00	8.29E-01	1.00E+00	1.00E+00	1.00E+00	thiamine transport system substrate-binding protein
KO:K02065	2.71	2.70	1.46	3.72	6.37	2.49	0.74	4.83E-01	4.84E-01	8.12E-01	2.66E-01	6.84E-02	5.50E-01	9.20E-01	putative ABC transport system ATP-binding protein
KO:K02066	3.50	5.26	3.12	5.38	6.56	0.08	0.04	4.38E-01	1.85E-01	5.19E-01	1.77E-01	1.05E-01	9.81E-01	9.87E-01	putative ABC transport system permease protein
KO:K02067	1.65	8.76	0.02	2.06	9.77	0.03	0.00	6.28E-01	3.11E-02	9.65E-01	5.19E-01	2.37E-02	9.54E-01	9.69E-01	putative ABC transport system substrate-binding protein
KO:K02068	0.00	0.00	0.00	0.00	0.00	0.00	0.42	1.00E+00	1.00E+00	1.00E+00	1.00E+00	1.00E+00	1.00E+00	3.03E-02	putative ABC transport system ATP-binding protein
KO:K02069	0.37	0.00	0.96	0.10	0.00	0.00	0.00	1.53E-01	1.00E+00	4.93E-02	3.38E-01	1.00E+00	1.00E+00	1.00E+00	putative ABC transport system permease protein
KO:K02071	0.00	0.01	0.00	1.33	0.00	0.00	0.00	1.00E+00	2.00E-01	1.00E+00	1.10E-01	1.00E+00	1.00E+00	1.00E+00	D-methionine transport system ATP-binding protein
KO:K02073	0.00	0.11	0.00	0.00	0.00	0.00	0.00	1.00E+00	1.03E-01	1.00E+00	1.00E+00	1.00E+00	1.00E+00	1.00E+00	D-methionine transport system substrate-binding protein
KO:K02074	0.00	0.05	0.00	0.31	10.60	0.00	0.00	1.00E+00	4.96E-01	1.00E+00	2.99E-01	5.57E-03	1.00E+00	1.00E+00	zinc/manganese transport system ATP-binding protein
KO:K02075	0.00	0.10	0.00	0.40	13.93	0.00	0.03	1.00E+00	5.19E-01	1.00E+00	3.26E-01	5.86E-03	1.00E+00	6.08E-01	zinc/manganese transport system permease protein
KO:K02077	0.05	0.07	0.19	0.43	6.71	0.00	0.04	6.26E-01	5.92E-01	4.41E-01	3.07E-01	1.76E-02	1.00E+00	6.46E-01	zinc/manganese transport system substrate-binding protein
KO:K02078	9.67	6.12	3.91	6.54	4.97	2.10	4.17	1.12E-01	4.37E-01	8.02E-01	3.74E-01	6.43E-01	9.32E-01	7.71E-01	acyl carrier protein
KO:K02080	0.00	0.11	0.00	0.00	0.00	0.00	0.00	1.00E+00	9.79E-02	1.00E+00	1.00E+00	1.00E+00	1.00E+00	1.00E+00	galactosamine-6-phosphate isomerase [EC:5.3.1.-]
KO:K02081	0.00	0.21	0.00	0.08	0.00	0.00	0.00	1.00E+00	1.17E-01	1.00E+00	1.98E-01	1.00E+00	1.00E+00	1.00E+00	DeoR family transcriptional regulator, aga operon transcriptional
KO:K02082	0.15	0.31	0.00	0.07	0.00	0.00	0.00	2.02E-01	1.61E-01	1.00E+00	2.84E-01	1.00E+00	1.00E+00	1.00E+00	tagatose-6-phosphate ketose/aldose isomerase [EC:5.-.-.-]
KO:K02083	0.23	0.00	0.00	0.00	0.00	0.00	0.00	6.50E-02	1.00E+00	1.00E+00	1.00E+00	1.00E+00	1.00E+00	1.00E+00	allantoate deiminase [EC:3.5.3.9]
KO:K02092	0.00	0.00	0.00	0.00	38.13	0.00	0.00	1.00E+00	1.00E+00	1.00E+00	1.00E+00	0.00E+00	1.00E+00	1.00E+00	allophycocyanin alpha subunit
KO:K02093	0.00	0.00	0.00	0.00	41.88	0.00	0.00	1.00E+00	1.00E+00	1.00E+00	1.00E+00	1.43E-04	1.00E+00	1.00E+00	allophycocyanin beta subunit
KO:K02094	0.00	0.00	0.00	0.00	23.13	0.00	0.00	1.00E+00	1.00E+00	1.00E+00	1.00E+00	0.00E+00	1.00E+00	1.00E+00	phycobilisome core linker protein
KO:K02095	0.00	0.00	0.00	0.00	31.25	0.00	0.00	1.00E+00	1.00E+00	1.00E+00	1.00E+00	2.86E-04	1.00E+00	1.00E+00	allophycocyanin-B
KO:K02096	0.00	0.00	0.00	0.00	41.25	0.00	0.00	1.00E+00	1.00E+00	1.00E+00	1.00E+00	0.00E+00	1.00E+00	1.00E+00	phycobilisome core-membrane linker protein
KO:K02097	0.00	0.00	0.00	0.00	32.50	0.00	0.00	1.00E+00	1.00E+00	1.00E+00	1.00E+00	0.00E+00	1.00E+00	1.00E+00	phycobilisome core component
KO:K02106	0.32	1.40	0.00	0.25	0.00	0.00	0.00	2.75E-01	1.10E-01	1.00E+00	3.07E-01	1.00E+00	1.00E+00	1.00E+00	short-chain fatty acids transporter
KO:K02108	12.78	12.42	7.05	12.00	7.05	3.67	4.87	1.23E-01	1.43E-01	8.61E-01	1.73E-01	8.61E-01	9.79E-01	9.56E-01	F-type H+-transporting ATPase subunit a [EC:3.6.3.14]
KO:K02109	10.03	16.14	5.24	6.02	11.45	2.46	2.93	1.96E-01	1.50E-02	8.96E-01	8.28E-01	9.93E-02	9.87E-01	9.80E-01	F-type H+-transporting ATPase subunit b [EC:3.6.3.14]
KO:K02110	6.07	3.15	1.74	4.65	6.07	4.46	3.64	2.31E-01	6.94E-01	8.81E-01	4.16E-01	2.31E-01	4.45E-01	6.03E-01	F-type H+-transporting ATPase subunit c [EC:3.6.3.14]
KO:K02111	12.94	13.71	11.94	11.87	12.32	5.92	5.92	1.82E-01	1.10E-01	3.41E-01	3.52E-01	2.71E-01	9.82E-01	9.82E-01	F-type H+-transporting ATPase subunit alpha [EC:3.6.3.14]
KO:K02112	12.58	13.29	13.74	10.56	11.86	3.21	4.02	1.53E-01	1.04E-01	8.44E-02	4.41E-01	2.23E-01	9.93E-01	9.88E-01	F-type H+-transporting ATPase subunit beta [EC:3.6.3.14]
KO:K02113	8.35	13.80	2.76	6.43	7.73	1.53	1.86	2.11E-01	2.59E-02	9.41E-01	5.06E-01	2.87E-01	9.81E-01	9.73E-01	F-type H+-transporting ATPase subunit delta [EC:3.6.3.14]
KO:K02114	6.48	6.17	8.22	3.63	7.54	1.67	2.70	2.54E-01	2.97E-01	1.18E-01	7.64E-01	1.57E-01	9.44E-01	8.73E-01	F-type H+-transporting ATPase subunit epsilon [EC:3.6.3.14]
KO:K02115	11.74	14.99	8.11	8.14	6.87	2.41	4.56	9.99E-02	2.03E-02	6.52E-01	6.45E-01	8.47E-01	9.95E-01	9.69E-01	F-type H+-transporting ATPase subunit gamma [EC:3.6.3.14]
KO:K02116	1.92	11.39	0.00	2.44	7.12	4.06	1.99	7.80E-01	3.23E-02	1.00E+00	6.95E-01	1.12E-01	3.96E-01	7.72E-01	ATP synthase protein I
KO:K02117	0.00	0.00	0.00	0.00	0.00	0.00	9.38	1.00E+00	1.00E+00	1.00E+00	3.87E-01	1.00E+00	1.00E+00	5.57E-03	V-type H+-transporting ATPase subunit A [EC:3.6.3.14]
KO:K02118	0.00	0.00	0.00	0.00	0.00	0.00	7.40	1.00E+00	1.00E+00	1.00E+00	3.31E-01	1.00E+00	1.00E+00	5.86E-03	V-type H+-transporting ATPase subunit B [EC:3.6.3.14]
KO:K02119	0.00	0.00	0.00	0.00	0.00	0.00	3.33	1.00E+00	1.00E+00	1.00E+00	1.00E+00	1.00E+00	1.00E+00	1.41E-02	V-type H+-transporting ATPase subunit C [EC:3.6.3.14]
KO:K02120	0.00	0.00	0.00	0.00	0.00	0.00	4.58	1.00E+00	1.00E+00	1.00E+00	1.00E+00	1.00E+00	1.00E+00	6.71E-03	V-type H+-transporting ATPase subunit D [EC:3.6.3.14]
KO:K02121	0.00	0.00	0.00	0.00	0.00	0.00	7.08	1.00E+00	1.00E+00	1.00E+00	1.00E+00	1.00E+00	1.00E+00	1.40E-02	V-type H+-transporting ATPase subunit E [EC:3.6.3.14]
KO:K02122	0.00	0.00	0.00	0.00	0.00	0.00	3.33	1.00E+00	1.00E+00	1.00E+00	1.00E+00	1.00E+00	1.00E+00	1.79E-02	V-type H+-transporting ATPase subunit F [EC:3.6.3.14]
KO:K02123	0.00	0.00	0.00	0.01	0.00	0.00	7.30	1.00E+00	1.00E+00	1.00E+00	3.37E-01	1.00E+00	1.00E+00	8.14E-03	V-type H+-transporting ATPase subunit I [EC:3.6.3.14]
KO:K02124	0.00	0.00	0.00	0.00	0.00	0.00	5.31	1.00E+00	2.37E-01	1.00E+00	1.00E+00	1.00E+00	1.00E+00	2.34E-02	V-type H+-transporting ATPase subunit K [EC:3.6.3.14]
KO:K02134	0.00	0.00	0.00	2.04	0.00	0.00	0.00	1.00E+00	1.00E+00	1.00E+00	1.43E-01	1.00E+00	1.00E+00	1.00E+00	F-type H+-transporting ATPase subunit delta [EC:3.6.3.14]
KO:K02160	5.38	3.95	3.02	2.85	11.77	3.61	0.79	2.13E-01	4.78E-01	7.04E-01	7.41E-01	1.59E-02	5.58E-01	9.67E-01	acetyl-CoA carboxylase biotin carboxyl carrier protein
KO:K02164	0.00	0.00	0.00	0.00	0.00	3.23	0.00	1.00E+00	2.50E-01	1.00E+00	1.00E+00	1.00E+00	1.14E-03	1.00E+00	nitric-oxide reductase NorE protein [EC:1.7.99.7]
KO:K02167	0.03	8.50	0.00	0.00	0.00	0.00	0.00	6.58E-01	1.27E-02	1.00E+00	7.73E-01	1.00E+00	1.00E+00	1.00E+00	TetR/AcrR family transcriptional regulator, transcriptional

KO:K02168	4.62	0.97	0.02	1.68	0.00	8.15	0.02	8.97E-02	5.95E-01	9.13E-01	3.91E-01	1.00E+00	3.14E-02	9.13E-01	high-affinity choline transport protein
KO:K02169	0.06	0.02	0.15	0.59	0.06	4.14	1.22	7.22E-01	8.10E-01	6.03E-01	2.71E-01	7.12E-01	1.74E-02	1.08E-01	biotin synthesis protein BioC
KO:K02170	1.84	0.00	0.00	0.09	0.00	0.00	2.90	8.64E-02	6.92E-01	1.00E+00	5.05E-01	1.00E+00	1.00E+00	6.10E-02	biotin biosynthesis protein BioH
KO:K02182	3.82	0.11	0.00	0.57	0.00	0.00	0.00	5.47E-02	5.21E-01	1.00E+00	2.95E-01	1.00E+00	1.00E+00	1.00E+00	crotonobetaine/carnitine-CoA ligase [EC:6.2.1.-]
KO:K02188	0.43	0.28	0.00	0.37	9.49	0.00	0.65	4.98E-01	5.96E-01	1.00E+00	5.34E-01	1.00E-02	1.00E+00	3.86E-01	cobalamin biosynthesis protein CbiD
KO:K02189	0.58	0.25	0.00	0.00	0.00	0.00	1.42	1.77E-01	2.51E-01	1.00E+00	1.00E+00	1.00E+00	1.00E+00	8.73E-02	cobalamin biosynthesis protein CbiG
KO:K02191	0.00	0.00	0.00	0.00	2.25	0.00	1.00	1.00E+00	1.00E+00	1.00E+00	1.00E+00	3.17E-02	1.00E+00	7.80E-02	precorrin-8W decarboxylase [EC:1.-.-.-]
KO:K02192	1.89	0.04	0.00	0.28	0.00	0.00	0.00	5.27E-02	5.48E-01	1.00E+00	2.90E-01	1.00E+00	1.00E+00	1.00E+00	bacterioferritin-associated ferredoxin
KO:K02193	5.11	9.89	0.11	0.67	0.00	0.00	0.80	6.66E-02	1.07E-02	9.57E-01	8.38E-01	1.00E+00	1.00E+00	8.06E-01	heme exporter protein A [EC:3.6.3.41]
KO:K02194	5.85	8.32	0.17	0.61	0.00	0.00	1.10	5.20E-02	2.17E-02	9.35E-01	8.49E-01	1.00E+00	1.00E+00	7.19E-01	heme exporter protein B
KO:K02195	13.37	8.16	0.17	5.49	0.00	0.69	3.63	1.97E-02	1.07E-01	9.82E-01	3.12E-01	1.00E+00	9.59E-01	6.33E-01	heme exporter protein C
KO:K02196	3.62	0.58	0.60	0.49	0.00	0.00	0.48	4.59E-02	5.02E-01	4.87E-01	5.53E-01	1.00E+00	1.00E+00	5.60E-01	heme exporter protein D
KO:K02197	8.57	4.57	0.00	3.09	0.00	1.01	4.40	3.77E-02	1.92E-01	1.00E+00	4.26E-01	1.00E+00	8.74E-01	2.09E-01	cytochrome c-type biogenesis protein CcmE
KO:K02198	14.72	21.01	0.49	5.85	0.00	1.29	3.10	3.00E-02	5.43E-03	9.87E-01	5.92E-01	1.00E+00	9.73E-01	9.01E-01	cytochrome c-type biogenesis protein CcmF
KO:K02199	6.87	3.61	0.16	1.71	0.00	0.18	1.25	3.36E-02	1.38E-01	9.46E-01	5.16E-01	1.00E+00	9.42E-01	6.83E-01	cytochrome c biogenesis protein CcmG, thiol:disulfide interchange
KO:K02200	6.41	14.65	0.10	1.29	0.00	0.03	0.94	8.01E-02	7.57E-03	9.67E-01	8.05E-01	1.00E+00	9.79E-01	8.66E-01	cytochrome c-type biogenesis protein CcmH
KO:K02201	0.00	0.00	0.00	0.00	0.00	0.00	1.25	1.00E+00	1.00E+00	1.00E+00	1.00E+00	1.00E+00	1.00E+00	2.54E-02	pantetheine-phosphate adenylyltransferase [EC:2.7.7.3]
KO:K02203	2.20	0.02	8.76	0.43	0.58	2.62	0.37	2.15E-01	7.92E-01	3.17E-02	6.56E-01	6.02E-01	1.75E-01	6.84E-01	phosphoserine / homoserine phosphotransferase [EC:3.1.3.3 2.7.1.39]
KO:K02204	0.74	0.03	0.00	7.67	0.00	0.00	0.43	4.17E-01	8.64E-01	1.00E+00	1.49E-02	1.00E+00	1.00E+00	5.85E-01	homoserine kinase type II [EC:2.7.1.39]
KO:K02217	0.00	0.54	0.40	0.02	0.40	0.00	3.22	7.09E-01	2.51E-01	3.02E-01	6.74E-01	3.02E-01	1.00E+00	2.96E-02	ferritin [EC:1.16.3.1]
KO:K02221	5.63	1.71	0.03	1.06	11.25	0.43	0.43	8.81E-02	5.90E-01	9.48E-01	7.57E-01	1.61E-02	8.78E-01	8.78E-01	YggT family protein
KO:K02224	0.12	3.22	1.85	0.43	2.48	1.78	2.79	8.84E-01	1.34E-01	3.05E-01	7.75E-01	2.00E-01	3.18E-01	1.70E-01	cobyrinic acid a,c-diamide synthase [EC:6.3.5.9 6.3.1.-]
KO:K02225	0.15	3.25	0.00	0.05	0.00	0.00	0.00	5.59E-01	9.29E-03	1.00E+00	7.37E-01	1.00E+00	1.00E+00	1.00E+00	cobalamin biosynthetic protein CobC
KO:K02227	0.05	3.38	0.00	0.52	6.40	0.00	0.13	9.11E-01	4.56E-02	1.00E+00	5.79E-01	1.16E-02	1.00E+00	8.39E-01	adenosylcobinamide-phosphate synthase CobD [EC:6.3.1.1]
KO:K02228	0.00	5.16	0.00	0.02	0.00	0.00	0.00	7.23E-01	1.09E-02	1.00E+00	7.02E-01	1.00E+00	1.00E+00	1.00E+00	precorrin-6A synthase [EC:2.1.1.152]
KO:K02229	0.01	4.28	0.00	0.06	0.00	0.00	0.00	7.27E-01	1.26E-02	1.00E+00	5.84E-01	1.00E+00	1.00E+00	1.00E+00	precorrin-3B synthase [EC:1.14.13.83]
KO:K02230	0.12	12.91	0.00	1.04	8.60	0.00	0.00	9.06E-01	8.29E-03	1.00E+00	6.57E-01	2.80E-02	1.00E+00	1.00E+00	cobaltochelatase CobN [EC:6.6.1.2]
KO:K02231	0.74	0.78	0.00	0.05	4.30	0.00	1.91	3.23E-01	3.12E-01	1.00E+00	8.08E-01	4.36E-02	1.00E+00	1.25E-01	adenosylcobinamide kinase / adenosylcobinamide-phosphate
KO:K02232	0.21	9.30	0.00	0.74	4.30	0.00	0.04	8.51E-01	1.36E-02	1.00E+00	6.43E-01	6.57E-02	1.00E+00	9.42E-01	adenosylcobyric acid synthase [EC:6.3.5.1]
KO:K02233	0.47	3.31	0.00	0.07	7.83	0.00	2.48	7.01E-01	9.07E-02	1.00E+00	8.85E-01	1.50E-02	1.00E+00	1.41E-01	adenosylcobinamide-GDP ribazoletransferase [EC:2.7.8.26]
KO:K02234	0.11	7.32	0.00	0.06	17.99	0.00	0.00	8.25E-01	3.99E-02	1.00E+00	8.52E-01	3.43E-03	1.00E+00	1.00E+00	cobalamin biosynthesis protein CobW
KO:K02237	5.01	0.00	0.00	0.10	0.00	0.00	0.00	3.17E-02	7.08E-01	1.00E+00	4.73E-01	1.00E+00	1.00E+00	1.00E+00	competence protein ComEA
KO:K02238	0.83	0.54	0.82	0.61	1.35	0.00	0.12	2.51E-01	4.09E-01	2.58E-01	3.64E-01	1.20E-01	1.00E+00	8.49E-01	competence protein ComEC
KO:K02257	0.00	0.00	0.00	0.00	1.25	0.00	0.00	1.00E+00	1.00E+00	1.00E+00	1.00E+00	1.76E-02	1.00E+00	1.00E+00	protoheme IX farnesyltransferase [EC:2.5.1.-]
KO:K02258	10.80	5.79	1.20	4.26	0.00	2.35	2.46	4.34E-02	2.21E-01	9.03E-01	4.20E-01	1.00E+00	7.73E-01	7.60E-01	cytochrome c oxidase subunit XI assembly protein
KO:K02259	8.88	11.07	3.62	7.66	2.39	3.62	1.68	1.31E-01	5.63E-02	8.93E-01	2.45E-01	9.56E-01	8.93E-01	9.75E-01	cytochrome c oxidase subunit XV assembly protein
KO:K02274	11.08	11.22	18.84	12.26	7.36	5.74	5.16	4.34E-01	4.11E-01	8.00E-03	2.50E-01	9.09E-01	9.62E-01	9.72E-01	cytochrome c oxidase subunit I [EC:1.9.3.1]
KO:K02275	11.62	8.81	17.53	8.61	2.42	12.31	3.39	1.59E-01	5.06E-01	1.57E-02	5.37E-01	9.85E-01	1.22E-01	9.70E-01	cytochrome c oxidase subunit II [EC:1.9.3.1]
KO:K02276	12.27	6.23	11.66	13.25	2.47	5.63	2.55	1.10E-01	7.73E-01	1.35E-01	7.86E-02	9.71E-01	8.33E-01	9.70E-01	cytochrome c oxidase subunit III [EC:1.9.3.1]
KO:K02277	0.00	0.00	2.15	0.06	0.00	0.00	0.00	1.00E+00	1.00E+00	1.51E-02	2.49E-01	1.00E+00	1.00E+00	1.00E+00	cytochrome c oxidase subunit IV [EC:1.9.3.1]
KO:K02278	0.03	0.06	17.74	0.02	2.71	0.00	0.00	6.38E-01	5.98E-01	4.14E-03	7.00E-01	9.10E-02	1.00E+00	1.00E+00	prepilin peptidase CpaA [EC:3.4.23.43]
KO:K02279	0.00	0.03	5.88	0.52	0.00	0.00	0.05	7.46E-01	6.53E-01	5.43E-03	2.07E-01	1.00E+00	1.00E+00	5.91E-01	pilus assembly protein CpaB
KO:K02280	0.08	2.39	0.97	1.47	0.00	0.00	0.14	8.51E-01	5.77E-02	2.53E-01	1.28E-01	1.00E+00	1.00E+00	7.97E-01	pilus assembly protein CpaC
KO:K02281	0.00	0.00	0.00	1.22	0.00	0.00	0.00	1.00E+00	1.00E+00	1.00E+00	3.49E-02	1.00E+00	1.00E+00	1.00E+00	pilus assembly protein CpaD
KO:K02282	0.00	0.01	6.91	0.10	0.29	0.00	0.66	7.32E-01	6.98E-01	1.00E-02	4.80E-01	3.05E-01	1.00E+00	1.81E-01	pilus assembly protein CpaE
KO:K02283	0.08	0.02	12.84	1.61	0.74	0.00	0.66	8.06E-01	8.59E-01	2.29E-03	2.08E-01	4.62E-01	1.00E+00	4.97E-01	pilus assembly protein CpaF
KO:K02284	0.00	0.00	0.00	0.00	27.50	0.00	0.00	1.00E+00	1.00E+00	1.00E+00	1.00E+00	0.00E+00	1.00E+00	1.00E+00	phycocyanin alpha chain
KO:K02285	0.00	0.00	0.00	0.00	35.63	0.00	0.00	1.00E+00	1.00E+00	1.00E+00	1.00E+00	1.43E-04	1.00E+00	1.00E+00	phycocyanin beta chain
KO:K02286	0.00	0.00	0.00	0.00	10.00	0.00	0.00	1.00E+00	1.00E+00	1.00E+00	1.00E+00	2.29E-03	1.00E+00	1.00E+00	phycocyanin-associated rod linker protein
KO:K02288	0.00	0.00	0.00	0.00	8.13	0.00	0.00	1.00E+00	1.00E+00	1.00E+00	1.00E+00	3.29E-03	1.00E+00	1.00E+00	phycocyanobilin lyase alpha subunit [EC:4.-.-.-]
KO:K02289	0.00	0.00	0.00	0.00	6.88	0.00	0.00	1.00E+00	1.00E+00	1.00E+00	1.00E+00	2.00E-03	1.00E+00	1.00E+00	phycocyanobilin lyase beta subunit
KO:K02290	0.00	0.00	0.00	0.00	30.63	0.00	0.00	1.00E+00	1.00E+00	1.00E+00	1.00E+00	0.00E+00	1.00E+00	1.00E+00	phycobilisome rod-core linker protein
KO:K02291	2.86	5.20	0.02	2.18	11.22	0.00	0.19	3.27E-01	9.23E-02	9.70E-01	4.88E-01	1.20E-02	1.00E+00	9.39E-01	phytoene synthase [EC:2.5.1.32]
KO:K02293	0.00	0.00	0.00	0.00	35.63	0.00	0.00	1.00E+00	1.00E+00	1.00E+00	1.00E+00	0.00E+00	1.00E+00	1.00E+00	phytoene dehydrogenase, phytoene desaturase [EC:1.14.99.-]
KO:K02294	0.50	0.62	0.00	0.50	15.27	6.29	0.00	6.50E-01	6.01E-01	1.00E+00	6.49E-01	4.71E-03	4.30E-02	1.00E+00	beta-carotene hydroxylase [EC:1.14.13.-]
KO:K02297	0.00	0.33	0.00	0.00	0.00	0.00	0.00	1.00E+00	1.00E-01	1.00E+00	1.00E+00	1.00E+00	1.00E+00	1.00E+00	cytochrome o ubiquinol oxidase subunit II [EC:1.1.3.-]
KO:K02298	0.08	0.13	0.26	0.01	0.00	0.00	0.00	2.82E-01	2.13E-01	1.07E-01	4.93E-01	1.00E+00	1.00E+00	1.00E+00	cytochrome o ubiquinol oxidase subunit I [EC:1.1.3.-]
KO:K02299	0.04	0.11	8.58	0.03	0.00	0.00	0.00	3.87E-01	3.13E-01	1.29E-02	3.95E-01	1.00E+00	1.00E+00	1.00E+00	cytochrome o ubiquinol oxidase subunit III [EC:1.1.3.-]
KO:K02301	8.34	11.14	11.38	6.81	1.34	8.46	3.72	3.24E-01	9.09E-02	8.14E-02	6.01E-01	9.90E-01	3.10E-01	9.34E-01	protoheme IX farnesyltransferase [EC:2.5.1.-]
KO:K02302	3.50	7.13	0.04	0.74	0.00	0.14	8.38	2.17E-01	4.44E-02	9.52E-01	8.39E-01	1.00E+00	9.38E-01	2.89E-02	uroporphyrin-III C-methyltransferase / precorrin-2 dehydrogenase /
KO:K02303	0.43	3.01	0.01	0.30	16.02	0.00	0.79	7.90E-01	1.08E-01	9.52E-01	8.39E-01	1.43E-03	1.00E+00	6.35E-01	uroporphyrin-III C-methyltransferase [EC:2.1.1.17]
KO:K02304	0.00	0.00	0.00	0.73	0.00	0.00	0.34	1.00E+00	1.00E+00	1.00E+00	1.23E-01	1.00E+00	1.00E+00	1.57E-01	precorrin-2 dehydrogenase / sirohydrochlorin ferrochelatase
KO:K02313	10.11	14.42	10.96	5.43	2.45	3.25	2.45	1.62E-01	3.34E-02	1.15E-01	7.83E-01	9.61E-01	9.34E-01	9.61E-01	chromosomal replication initiator protein
KO:K02314	8.55	8.63	9.68	4.76	3.46	6.15	3.82	1.88E-01	1.82E-01	1.15E-01	7.97E-01	9.13E-01	5.67E-01	8.90E-01	replicative DNA helicase [EC:3.6.1.-]
KO:K02316	7.31	9.73	3.33	5.51	4.19	0.90	1.08	1.42E-01	5.10E-02	8.31E-01	3.76E-01	6.78E-01	9.85E-01	9.80E-01	DNA primase [EC:2.7.7.-]
KO:K02319	0.00	0.00	0.00	0.00	0.00	0.00	3.33	1.00E+00	1.00E+00	1.00E+00	1.00E+00	1.00E+00	1.00E+00	1.53E-02	DNA polymerase I [EC:2.7.7.7]
KO:K02322	0.00	0.00	0.00	0.00	0.00	0.00	5.42	1.00E+00	1.00E+00	1.00E+00	1.00E+00	1.00E+00	1.00E+00	6.00E-03	DNA polymerase II large subunit [EC:2.7.7.7]
KO:K02323	0.00	0.00	0.00	0.00	0.00	0.00	4.58	1.00E+00	1.00E+00	1.00E+00	1.00E+00	1.00E+00	1.00E+00	4.43E-03	DNA polymerase II small subunit [EC:2.7.7.7]
KO:K02334	0.37	3.92	6.04	0.80	2.22	0.04	0.56	8.53E-01	8.63E-02	3.73E-02	7.05E-01	2.44E-01	9.58E-01	7.96E-01	DNA polymerase bacteriophage-type [EC:2.7.7.7]
KO:K02335	9.65	8.35	6.72	6.45	3.21	4.56	1.68	6.43E-02	1.32E-01	3.72E-01	4.39E-01	9.43E-01	8.39E-01	9.89E-01	DNA polymerase I [EC:2.7.7.7]
KO:K02336	1.54	0.12	0.19	0.10	0.00	0.00	0.33	7.36E-02	5.43E-01	4.71E-01	5.65E-01	1.00E+00	1.00E+00	3.46E-01	DNA polymerase II [EC:2.7.7.7]
KO:K02337	11.15	10.05	12.07	6.04	6.13	13.95	2.22	1.16E-01	2.06E-01	7.46E-02	8.54E-01	8.46E-01	3.40E-02	9.91E-01	DNA polymerase III subunit alpha [EC:2.7.7.7]
KO:K02338	9.37	9.69	12.03	8.17	6.85	3.29	2.10	2.01E-01	1.71E-01	5.84E-02	3.79E-01	6.51E-01	9.68E-01	9.88E-01	DNA polymerase III subunit beta [EC:2.7.7.7]
KO:K02339	4.31	4.52	0.37	0.46	0.00	0.00	0.46	6.43E-02	5.91E-02	8.01E-01	7.68E-01	1.00E+00	1.00E+00	7.69E-01	DNA polymerase III subunit chi [EC:2.7.7.7]
KO:K02340	5.25	2.83	4.94	1.68	0.68	0.03	1.84	7.49E-02	3.30E-01	8.90E-02	6.95E-01	9.17E-01	9.91E-01	6.42E-01	DNA polymerase III subunit delta [EC:2.7.7.7]
KO:K02341	3.55	1.82	2.11	1.99	3.66	0.28	0.85	1.29E-01	5.48E-01	4.34E-01	4.83E-01	1.19E-01	9.62E-01	8.68E-01	DNA polymerase III subunit delta' [EC:2.7.7.7]
KO:K02342	7.24	5.02	2.86	4.49	0.82	1.95	2.19	7.40E-02	2.45E-01	7.44E-01	3.40E-01	9.70E-01	8.85E-01	8.56E-01	DNA polymerase III subunit epsilon [EC:2.7.7.7]
KO:K02343	7.95	4.54	11.73	5.97	3.61	4.77	3.56	1.69E-01	7.60E-01	3.24E-02	4.66E-01	8.76E-01	7.20E-01	8.81E-01	DNA polymerase III subunit gamma/tau [EC:2.7.7.7]
KO:K02346	1.12	7.34	0.74	1.22	0.74	0.21	0.03	6.04E-01	1.60E-02	7.62E-01	5.65E-01	7.62E-01	9.22E-01	9.84E-01	DNA polymerase IV [EC:2.7.7.7]
KO:K02347	0.03	0.00	15.63	0.05	0.15	0.00	0.03	5.01E-01	1.00E+00	3.14E-03	4.67E-01	3.97E-01	1.00E+00	4.99E-01	DNA polymerase (family X)
KO:K02348	2.05	0.45	0.00	0.75	0.00	2.23	0.00	8.61E-02	4.72E-01	1.00E+00	3.19E-01	1.00E+00	7.53E-02	1.00E+00	ElaA protein
KO:K02355	13.77	13.23	20.28	10.60	12.44	5.88	5.36	1.87E-01	2.57E-01	5.57E-03	7.57E-01	4.02E-01	9.86E-01	9.90E-01	elongation factor EF-G [EC:3.6.5.3]
KO:K02356	6.59	6.26	7.79	6.25	6.93	1.13	2.46	3.00E-01	3.50E-01	1.63E-01	3.52E-01	2.53E-01	9.76E-01	9.22E-01	elongation factor EF-P
KO:K02357	10.08	11.67	10.19	9.11	6.39	4.80	5.08	2.74E-01	1.27E-01	2.60E-01	4.34E-01	8.63E-01	9.41E-01	9.33E-01	elongation factor EF-Ts
KO:K02358	12.95	12.86	14.82	12.84	9.99	7.05	13.01	4.01E-01	4.21E-01	1.27E-01	4.24E-01	8.87E-01	9.71E-01	3.88E-01	elongation factor EF-Tu [EC:3.6.5.3]
KO:K02361	0.87	0.34	0.00	1.44	0.00	0.27	0.00	1.96E-01	4.29E-01	1.00E+00	1.08E-01	1.00E+00	4.88E-01	1.00E+00	isochorismate synthase [EC:5.4.4.2]
KO:K02362	0.00	0.00	0.00	0.10	0.00	0.00	0.00	1.00E+00	1.00E+00	1.00E+00	1.43E-01	1.00E+00	1.00E+00	1.00E+00	enterobactin synthetase component D [EC:2.7.8.-]
KO:K02364	0.00	0.06	0.00	0.05	0.00	0.00	0.00	1.00E+00	1.47E-01	1.00E+00	1.88E-01	1.00E+00	1.00E+00	1.00E+00	enterobactin synthetase component F [EC:2.7.7.-]
KO:K02371	8.54	0.39	0.00	2.69	0.00	1.08	0.02	2.94E-02	7.57E-01	1.00E+00	2.06E-01	1.00E+00	5.19E-01	8.83E-01	enoyl-[acyl carrier protein] reductase II [EC:1.3.1.-]
KO:K02372	3.82	2.99	26.66	2.15	7.19	0.00	1.61	5.08E-01	6.71E-01	8.57E-04	8.10E-01	1.22E-01	1.00E+00	8.71E-01	3R-hydroxymyristoyl ACP dehydrase [EC:4.2.1.-]
KO:K02377	2.08	1.81	5.80	1.21	4.93	2.46	2.09	5.51E-01	6.28E-01	6.91E-02	7.93E-01	1.07E-01	4.33E-01	5.49E-01	GDP-L-fucose synthase [EC:1.1.1.271]
KO:K02379	2.69	10.48	1.50	2.92	0.19	0.00	0.11	3.91E-01	1.80E-02	6.94E-01	3.43E-01	9.41E-01	1.00E+00	9.50E-01	FdhD protein
KO:K02385	0.00	0.00	1.07	0.03	0.00	0.00	0.00	1.00E+00	1.00E+00	1.30E-02	2.42E-01	1.00E+00	1.00E+00	1.00E+00	flagellar protein FlbD
KO:K02386	2.32	0.20	0.44	0.13	0.00	4.26	0.35	9.90E-02	6.64E-01	4.94E-01	7.26E-01	1.00E+00	4.27E-02	5.57E-01	flagella basal body P-ring formation protein FlgA
KO:K02387	9.42	0.92	7.94	0.18	0.00	0.05	0.00	3.07E-02	5.92E-01	4.11E-02	8.22E-01	1.00E+00	8.76E-01	9.34E-01	flagellar basal-body rod protein FlgB
KO:K02388	4.65	0.92	4.40	0.27	0.01	2.91	1.52	1.03E-01	6.17E-01	1.12E-01	8.04E-01	9.38E-01	2.06E-01	4.48E-01	flagellar basal-body rod protein FlgC

KO:K02389	10.82	0.80	1.04	0.74	0.00	0.24	0.03	1.91E-02	6.24E-01	5.46E-01	6.42E-01	1.00E+00	7.96E-01	8.99E-01	flagellar basal-body rod modification protein FlgD
KO:K02390	7.50	0.35	7.92	2.17	0.00	0.52	0.59	4.90E-02	8.66E-01	4.47E-02	4.26E-01	1.00E+00	8.32E-01	8.19E-01	flagellar hook protein FlgE
KO:K02391	8.95	1.87	1.77	1.53	0.00	0.97	0.24	3.79E-02	4.86E-01	5.09E-01	5.71E-01	1.00E+00	7.13E-01	8.70E-01	flagellar basal-body rod protein FlgF
KO:K02392	4.67	1.16	17.94	1.38	0.12	4.90	1.08	2.03E-01	7.77E-01	1.00E-02	7.35E-01	9.39E-01	1.90E-01	7.91E-01	flagellar basal-body rod protein FlgG
KO:K02393	5.37	0.81	2.66	0.21	0.00	5.63	0.55	7.93E-02	6.10E-01	2.29E-01	8.01E-01	1.00E+00	7.26E-02	6.93E-01	flagellar L-ring protein precursor FlgH
KO:K02394	7.20	1.46	8.53	1.91	0.00	0.97	0.89	6.63E-02	6.98E-01	4.57E-02	5.82E-01	1.00E+00	8.05E-01	8.19E-01	flagellar P-ring protein precursor FlgI
KO:K02395	5.88	0.29	0.00	0.07	0.00	0.15	0.58	2.51E-02	5.65E-01	1.00E+00	7.52E-01	1.00E+00	6.67E-01	4.00E-01	flagellar protein FlgJ
KO:K02396	5.61	0.59	8.54	0.83	0.00	2.20	0.22	8.13E-02	7.58E-01	3.97E-02	6.89E-01	9.45E-01	3.38E-01	8.49E-01	flagellar hook-associated protein 1 FlgK
KO:K02397	5.31	0.50	7.65	0.30	0.00	2.11	0.04	6.27E-02	7.15E-01	3.66E-02	7.82E-01	1.00E+00	2.72E-01	8.77E-01	flagellar hook-associated protein 3 FlgL
KO:K02398	8.89	0.16	0.20	0.12	0.00	0.00	0.00	1.90E-02	6.00E-01	5.69E-01	6.36E-01	1.00E+00	1.00E+00	1.00E+00	negative regulator of flagellin synthesis FlgM
KO:K02399	0.49	0.02	0.00	0.00	0.00	0.00	0.00	2.81E-02	3.83E-01	1.00E+00	1.00E+00	1.00E+00	1.00E+00	1.00E+00	flagella synthesis protein FlgN
KO:K02400	9.40	0.79	12.24	1.60	0.03	3.22	0.07	4.29E-02	8.42E-01	2.06E-02	6.97E-01	9.54E-01	3.51E-01	9.44E-01	flagellar biosynthesis protein FlhA
KO:K02401	6.88	0.58	7.01	0.92	0.01	0.91	0.51	4.46E-02	7.98E-01	4.34E-02	7.05E-01	9.54E-01	7.07E-01	8.19E-01	flagellar biosynthetic protein FlhB
KO:K02404	1.74	0.01	11.16	0.27	0.03	3.82	0.07	2.12E-01	8.73E-01	1.01E-02	6.90E-01	8.53E-01	6.57E-02	8.10E-01	flagellar biosynthesis protein FlhF
KO:K02405	4.32	0.50	20.54	0.66	0.94	2.98	0.57	1.74E-01	8.51E-01	3.86E-03	8.26E-01	7.63E-01	3.22E-01	8.40E-01	RNA polymerase sigma factor for flagellar operon FliA
KO:K02406	10.64	0.88	27.16	3.36	0.00	3.98	1.25	8.69E-02	9.18E-01	3.00E-03	6.67E-01	1.00E+00	5.77E-01	8.99E-01	flagellin
KO:K02407	5.86	0.48	12.50	0.90	0.08	4.04	0.66	1.05E-01	8.40E-01	1.69E-02	7.50E-01	8.99E-01	1.98E-01	8.05E-01	flagellar hook-associated protein 2
KO:K02408	3.21	0.34	9.46	0.24	0.00	0.07	0.00	1.04E-01	6.05E-01	2.49E-02	6.51E-01	1.00E+00	7.76E-01	8.67E-01	flagellar hook-basal body complex protein FliE
KO:K02409	5.70	0.78	4.77	1.84	0.00	8.02	0.82	9.40E-02	8.18E-01	1.38E-01	5.63E-01	1.00E+00	4.69E-02	8.11E-01	flagellar M-ring protein FliF
KO:K02410	7.75	1.46	6.00	1.20	0.00	0.82	0.40	5.77E-02	6.08E-01	9.13E-02	6.76E-01	9.55E-01	7.65E-01	8.44E-01	flagellar motor switch protein FliG
KO:K02411	0.61	0.08	0.44	0.13	0.00	0.35	0.02	1.42E-01	5.67E-01	2.06E-01	4.84E-01	1.00E+00	2.65E-01	7.18E-01	flagellar assembly protein FliH
KO:K02412	8.33	0.62	7.88	1.04	0.06	5.59	0.16	4.66E-02	8.39E-01	5.33E-02	7.53E-01	9.42E-01	1.03E-01	9.21E-01	flagellum-specific ATP synthase [EC:3.6.3.14]
KO:K02413	0.16	0.00	0.73	0.04	0.00	0.00	0.00	1.74E-01	1.00E+00	2.90E-02	4.04E-01	1.00E+00	1.00E+00	1.00E+00	flagellar FliJ protein
KO:K02414	0.49	0.28	0.00	0.03	0.00	0.00	0.06	1.11E-01	1.82E-01	1.00E+00	4.99E-01	1.00E+00	1.00E+00	4.21E-01	flagellar hook-length control protein FliK
KO:K02415	6.08	0.92	0.70	0.56	0.00	0.98	0.47	5.51E-02	5.25E-01	6.01E-01	6.42E-01	1.00E+00	5.06E-01	6.82E-01	flagellar FliL protein
KO:K02416	4.97	0.44	22.84	0.79	0.02	0.03	0.16	1.16E-01	7.89E-01	3.57E-03	6.99E-01	9.20E-01	9.13E-01	8.65E-01	flagellar motor switch protein FliM
KO:K02417	6.81	0.25	7.87	0.42	0.00	1.56	0.04	4.80E-02	8.01E-01	3.79E-02	7.43E-01	1.00E+00	3.90E-01	8.91E-01	flagellar motor switch protein FliN/FliY
KO:K02418	4.52	0.05	0.10	0.04	0.00	0.72	0.04	2.17E-02	7.59E-01	6.98E-01	7.68E-01	1.00E+00	2.62E-01	7.66E-01	flagellar protein FliO/FliZ
KO:K02419	5.06	0.52	4.93	0.39	0.07	8.77	0.27	1.05E-01	7.36E-01	1.08E-01	7.70E-01	8.78E-01	4.67E-02	8.09E-01	flagellar biosynthetic protein FliP
KO:K02420	4.51	0.67	0.43	0.41	0.00	0.00	0.28	6.09E-02	4.29E-01	5.22E-01	5.30E-01	1.00E+00	1.00E+00	5.99E-01	flagellar biosynthetic protein FliQ
KO:K02421	7.26	0.35	2.59	0.51	0.00	0.51	0.43	3.50E-02	7.55E-01	1.85E-01	6.91E-01	1.00E+00	6.91E-01	7.21E-01	flagellar biosynthetic protein FliR
KO:K02422	6.44	0.77	7.67	0.34	0.07	2.63	0.29	7.07E-02	7.04E-01	5.17E-02	8.09E-01	8.74E-01	2.83E-01	8.21E-01	flagellar protein FliS
KO:K02426	1.30	5.79	2.55	1.92	2.97	1.86	0.02	7.37E-01	6.39E-02	3.62E-01	5.36E-01	2.71E-01	5.55E-01	9.85E-01	cysteine desulfuration protein SufE
KO:K02427	7.93	7.82	0.00	2.31	0.00	4.84	1.48	5.19E-02	5.40E-02	9.91E-01	6.77E-01	1.00E+00	1.93E-01	8.39E-01	ribosomal RNA large subunit methyltransferase E [EC:2.1.1.-]
KO:K02428	4.87	3.52	6.30	1.19	3.76	1.13	0.07	9.90E-02	2.33E-01	4.53E-02	8.69E-01	1.96E-01	8.78E-01	9.92E-01	nucleoside-triphosphate pyrophosphatase [EC:3.6.1.19]
KO:K02429	3.36	0.62	3.08	0.59	0.68	0.00	0.00	8.93E-02	5.52E-01	1.02E-01	5.67E-01	5.29E-01	1.00E+00	1.00E+00	MFS transporter, FHS family, L-fucose permease
KO:K02431	0.01	1.53	0.04	0.48	0.16	0.00	0.16	6.59E-01	1.08E-01	5.93E-01	2.81E-01	4.51E-01	1.00E+00	4.51E-01	L-fucose mutarotase [EC:5.1.3.-]
KO:K02433	6.53	16.87	4.97	9.23	5.76	1.61	1.95	5.83E-01	1.27E-02	8.16E-01	2.07E-01	7.08E-01	9.82E-01	9.77E-01	aspartyl-tRNA(Asn)/glutamyl-tRNA (Gln) amidotransferase subunit A
KO:K02434	3.41	6.83	5.61	4.76	9.64	1.19	4.40	8.04E-01	1.85E-01	3.49E-01	5.23E-01	5.01E-02	9.70E-01	6.11E-01	aspartyl-tRNA(Asn)/glutamyl-tRNA (Gln) amidotransferase subunit B
KO:K02435	1.19	2.43	3.82	2.58	4.66	0.03	0.52	6.98E-01	3.19E-01	1.32E-01	2.83E-01	8.93E-02	9.77E-01	8.76E-01	aspartyl-tRNA(Asn)/glutamyl-tRNA (Gln) amidotransferase subunit C
KO:K02437	6.92	4.38	4.42	3.79	5.01	2.05	2.22	1.32E-01	4.62E-01	4.52E-01	5.98E-01	3.35E-01	8.88E-01	8.72E-01	glycine cleavage system H protein
KO:K02438	0.11	0.90	2.15	0.18	12.48	0.00	0.12	8.00E-01	3.60E-01	1.26E-01	7.50E-01	6.29E-03	1.00E+00	7.92E-01	glycogen operon protein GlgX [EC:3.2.1.-]
KO:K02439	0.70	0.04	2.45	0.10	0.00	0.00	0.07	1.79E-01	5.77E-01	3.79E-02	4.98E-01	1.00E+00	1.00E+00	5.38E-01	thiosulfate sulfurtransferase [EC:2.8.1.1]
KO:K02440	0.50	0.23	1.50	0.67	0.00	1.18	0.07	3.70E-01	5.21E-01	1.47E-01	3.12E-01	1.00E+00	1.89E-01	6.57E-01	glycerol uptake facilitator protein
KO:K02441	2.71	0.33	1.66	0.18	8.15	0.00	0.00	1.37E-01	6.59E-01	2.53E-01	7.34E-01	2.33E-02	1.00E+00	1.00E+00	GlpG protein
KO:K02444	0.50	13.34	0.01	4.23	0.00	0.00	0.00	7.95E-01	1.03E-02	9.09E-01	1.34E-01	1.00E+00	1.00E+00	9.25E-01	DeoR family transcriptional regulator, glycerol-3-phosphate regulon
KO:K02445	0.33	0.00	0.06	0.15	1.70	0.00	0.00	2.20E-01	1.00E+00	3.60E-01	3.03E-01	7.54E-02	1.00E+00	1.00E+00	MFS transporter, OPA family, glycerol-3-phosphate transporter
KO:K02446	0.11	15.33	0.00	4.85	0.00	0.00	0.13	9.17E-01	7.14E-03	1.00E+00	9.70E-02	1.00E+00	1.00E+00	9.10E-01	fructose-1,6-bisphosphatase II [EC:3.1.3.11]
KO:K02448	0.00	0.06	0.00	0.05	0.00	0.00	0.00	1.00E+00	1.47E-01	1.00E+00	1.89E-01	1.00E+00	1.00E+00	1.00E+00	nitric-oxide reductase NorD protein [EC:1.7.99.7]
KO:K02450	4.81	0.00	2.70	0.10	0.17	0.00	0.00	3.53E-02	1.00E+00	8.50E-02	6.82E-01	6.04E-01	1.00E+00	1.00E+00	general secretion pathway protein A
KO:K02451	0.93	0.00	0.00	0.00	0.00	0.00	0.00	2.49E-02	1.00E+00	1.00E+00	1.00E+00	1.00E+00	1.00E+00	1.00E+00	general secretion pathway protein B
KO:K02452	6.23	0.00	1.28	0.02	0.00	0.00	0.01	3.06E-02	1.00E+00	1.97E-01	6.43E-01	1.00E+00	1.00E+00	6.73E-01	general secretion pathway protein C
KO:K02453	7.42	0.00	35.07	0.69	0.00	0.17	0.84	5.97E-02	9.44E-01	8.57E-04	7.84E-01	1.00E+00	8.83E-01	7.51E-01	general secretion pathway protein D
KO:K02454	7.21	0.00	19.60	0.84	3.26	0.36	2.34	7.73E-02	9.49E-01	6.00E-03	7.92E-01	3.16E-01	8.71E-01	4.84E-01	general secretion pathway protein E
KO:K02455	8.66	0.00	10.82	0.30	0.00	0.04	0.90	2.73E-02	9.20E-01	1.87E-02	7.82E-01	1.00E+00	8.93E-01	5.48E-01	general secretion pathway protein F
KO:K02456	4.71	0.00	15.64	0.72	0.07	0.03	1.89	8.17E-02	9.37E-01	6.43E-03	6.74E-01	8.83E-01	9.10E-01	3.30E-01	general secretion pathway protein G
KO:K02457	3.70	0.00	0.23	0.04	0.00	0.00	0.00	2.93E-02	1.00E+00	3.47E-01	5.91E-01	1.00E+00	1.00E+00	1.00E+00	general secretion pathway protein H
KO:K02458	4.31	0.00	0.00	0.01	0.00	0.00	0.03	2.81E-02	1.00E+00	1.00E+00	5.78E-01	1.00E+00	1.00E+00	4.80E-01	general secretion pathway protein I
KO:K02459	6.05	0.00	0.00	0.00	0.00	0.27	0.00	2.29E-02	1.00E+00	1.00E+00	7.07E-01	1.00E+00	3.67E-01	1.00E+00	general secretion pathway protein J
KO:K02460	5.41	0.00	0.81	0.06	0.00	0.00	0.00	2.33E-02	1.00E+00	2.34E-01	6.27E-01	1.00E+00	1.00E+00	1.00E+00	general secretion pathway protein K
KO:K02461	8.60	0.00	0.00	0.03	0.00	0.00	0.00	1.64E-02	1.00E+00	1.00E+00	5.95E-01	1.00E+00	1.00E+00	1.00E+00	general secretion pathway protein L
KO:K02462	6.56	0.00	0.00	0.01	0.00	0.00	0.00	2.54E-02	1.00E+00	1.00E+00	6.91E-01	1.00E+00	1.00E+00	1.00E+00	general secretion pathway protein M
KO:K02463	3.27	0.00	0.00	0.00	0.00	0.00	0.04	5.06E-02	1.00E+00	1.00E+00	4.95E-01	1.00E+00	1.00E+00	3.90E-01	general secretion pathway protein N
KO:K02469	11.56	9.51	16.55	8.49	11.89	4.03	4.03	1.72E-01	4.87E-01	1.27E-02	6.90E-01	1.44E-01	9.81E-01	9.81E-01	DNA gyrase subunit A [EC:5.99.1.3]
KO:K02470	12.32	9.62	14.85	9.50	7.42	11.04	3.73	1.11E-01	5.18E-01	2.66E-02	5.47E-01	8.86E-01	2.34E-01	9.90E-01	DNA gyrase subunit B [EC:5.99.1.3]
KO:K02471	0.00	0.00	0.00	0.00	33.45	0.00	0.02	5.74E-01	1.00E+00	1.00E+00	1.00E+00	0.00E+00	1.00E+00	5.29E-01	putative ATP-binding cassette transporter
KO:K02472	0.90	2.84	0.00	0.03	0.00	0.00	0.63	2.52E-01	6.63E-02	1.00E+00	6.82E-01	1.00E+00	1.00E+00	3.33E-01	UDP-N-acetyl-D-mannosaminuronic acid dehydrogenase [EC:1.1.1.-]
KO:K02473	0.00	0.00	3.65	0.00	0.00	0.00	0.00	1.00E+00	1.00E+00	7.71E-03	2.50E-01	1.00E+00	1.00E+00	1.00E+00	UDP-N-acetylglucosamine 4-epimerase [EC:5.1.3.7]
KO:K02474	6.41	1.51	0.19	4.83	0.00	6.94	0.90	6.21E-02	7.41E-01	9.53E-01	1.30E-01	1.00E+00	5.11E-02	8.60E-01	UDP-N-acetyl-D-galactosamine dehydrogenase [EC:1.1.1.-]
KO:K02475	0.81	0.00	0.00	0.00	0.00	0.00	0.00	3.43E-02	1.00E+00	1.00E+00	1.00E+00	1.00E+00	1.00E+00	1.00E+00	two-component system, CitB family, response regulator
KO:K02476	0.31	0.07	0.00	0.00	0.00	0.00	0.00	9.93E-02	2.24E-01	1.00E+00	1.00E+00	1.00E+00	1.00E+00	1.00E+00	two-component system, CitB family, sensor kinase [EC:2.7.13.3]
KO:K02477	10.87	0.22	0.39	1.25	0.00	0.00	0.00	1.61E-02	7.78E-01	6.89E-01	3.80E-01	1.00E+00	1.00E+00	9.05E-01	two-component system, LytT family, response regulator
KO:K02478	0.00	0.00	0.00	0.10	0.00	0.00	0.00	1.00E+00	1.00E+00	1.00E+00	1.43E-01	1.00E+00	1.00E+00	1.00E+00	two-component system, LytT family, sensor kinase [EC:2.7.13.3]
KO:K02479	0.00	0.06	0.13	0.00	0.13	0.00	0.51	1.00E+00	3.29E-01	2.08E-01	5.66E-01	2.08E-01	1.00E+00	5.41E-02	two-component system, NarL family, response regulator
KO:K02480	0.00	0.91	0.00	0.19	0.00	0.00	0.00	1.00E+00	3.17E-02	1.00E+00	2.08E-01	1.00E+00	1.00E+00	1.00E+00	two-component system, NarL family, sensor kinase [EC:2.7.13.3]
KO:K02481	0.35	0.00	17.47	0.90	0.13	0.58	1.41	6.76E-01	9.13E-01	1.43E-03	4.25E-01	7.98E-01	5.60E-01	2.80E-01	two-component system, NtrC family, response regulator
KO:K02482	0.00	3.20	0.16	0.03	0.00	0.28	0.07	7.62E-01	3.67E-02	4.71E-01	6.79E-01	1.00E+00	3.51E-01	5.70E-01	two-component system, NtrC family, sensor kinase [EC:2.7.13.3]
KO:K02483	6.94	4.81	2.63	5.28	1.49	0.00	0.39	8.76E-02	2.14E-01	6.16E-01	1.70E-01	8.40E-01	1.00E+00	9.57E-01	two-component system, OmpR family, response regulator
KO:K02484	0.48	0.00	0.00	0.09	0.00	0.00	0.00	6.90E-02	1.00E+00	1.00E+00	2.50E-01	1.00E+00	1.00E+00	1.00E+00	two-component system, OmpR family, sensor kinase [EC:2.7.13.3]
KO:K02485	0.18	0.02	0.26	0.04	0.26	0.00	0.46	2.84E-01	6.70E-01	2.25E-01	5.71E-01	2.25E-01	1.00E+00	1.19E-01	two-component system, unclassified family, response regulator
KO:K02486	0.00	0.07	1.24	0.00	2.21	0.25	0.00	1.00E+00	3.32E-01	8.74E-02	1.00E+00	5.84E-02	2.21E-01	1.00E+00	two-component system, unclassified family, sensor kinase
KO:K02487	0.87	0.00	0.00	0.00	0.00	3.31	0.00	9.61E-02	1.00E+00	1.00E+00	1.00E+00	1.00E+00	3.21E-02	1.00E+00	type IV pili sensor histidine kinase and response regulator
KO:K02488	0.33	0.22	0.00	1.89	0.14	0.25	0.00	4.06E-01	5.20E-01	1.00E+00	5.64E-02	6.26E-01	4.81E-01	1.00E+00	two-component system, cell cycle response regulator
KO:K02489	0.00	0.00	0.00	0.31	0.00	0.00	0.00	1.00E+00	1.00E+00	1.00E+00	7.14E-02	1.00E+00	1.00E+00	1.00E+00	two-component system, cell cycle sensor kinase and response
KO:K02492	5.11	0.38	3.80	1.58	11.86	5.27	3.56	1.70E-01	9.46E-01	3.26E-01	8.11E-01	1.79E-02	1.56E-01	3.70E-01	glutamyl-tRNA reductase [EC:1.2.1.7]
KO:K02493	2.09	0.94	4.58	1.01	4.58	0.48	1.57	3.21E-01	7.66E-01	5.50E-02	7.36E-01	5.50E-02	9.07E-01	5.05E-01	methyltransferase [EC:2.1.1.-]
KO:K02494	0.13	0.07	0.03	0.09	0.00	5.72	0.00	3.75E-01	4.84E-01	5.68E-01	4.41E-01	1.00E+00	6.71E-03	1.00E+00	outer membrane lipoprotein LolB
KO:K02495	4.01	2.72	12.86	0.77	7.11	0.02	1.22	2.31E-01	4.90E-01	1.13E-02	9.19E-01	5.76E-02	9.99E-01	8.54E-01	oxygen-independent coproporphyrinogen III oxidase [EC:1.3.99.22]
KO:K02496	4.20	0.19	0.00	0.09	0.00	0.00	0.00	1.80E-02	4.87E-01	1.00E+00	6.07E-01	1.00E+00	1.00E+00	1.00E+00	uroporphyrin-III C-methyltransferase [EC:2.1.1.17]
KO:K02498	4.61	13.58	0.00	0.24	0.00	0.00	0.00	7.29E-02	5.14E-03	1.00E+00	8.93E-01	1.00E+00	1.00E+00	9.68E-01	HemY protein
KO:K02499	0.00	0.00	0.00	0.00	0.63	0.00	0.00	1.00E+00	1.00E+00	1.00E+00	1.00E+00	1.49E-02	1.00E+00	1.00E+00	tetrapyrrole methylase family protein / MazG family protein
KO:K02500	5.12	4.20	4.85	4.81	6.06	2.24	2.88	3.19E-01	5.27E-01	3.76E-01	3.85E-01	1.87E-01	8.90E-01	8.17E-01	cyclase HisF [EC:4.1.3.-]
KO:K02501	6.50	4.79	4.92	2.98	6.02	0.00	4.31	1.22E-01	3.10E-01	2.88E-01	7.34E-01	1.55E-01	1.00E+00	4.11E-01	glutamine amidotransferase [EC:2.4.2.-]
KO:K02502	7.38	5.72	0.20	3.56	3.40	1.77	3.71	1.13E-01	2.15E-01	9.72E-01	5.52E-01	5.88E-01	8.54E-01	5.22E-01	ATP phosphoribosyltransferase regulatory subunit
KO:K02503	3.54	2.57	1.48	2.38	1.80	0.66	3.34	2.36E-01	4.30E-01	7.34E-01	4.77E-01	6.49E-01	8.90E-01	2.67E-01	Hit-like protein involved in cell-cycle regulation
KO:K02509	1.37	4.48	0.00	0.15	0.00	0.00	0.00	1.81E-01	4.03E-02	1.00E+00	6.56E-01	1.00E+00	1.00E+00	1.00E+00	2-oxo-hept-3-ene-1,7-dioate hydratase [EC:4.2.1.-]

KO:K02510	0.41	4.51	0.88	1.99	1.05	0.05	0.55	7.64E-01	8.23E-02	5.89E-01	2.86E-01	5.34E-01	8.94E-01	7.16E-01	2,4-dihydroxyhept-2-ene-1,7-dioic acid aldolase [EC:4.1.2.-]
KO:K02517	9.18	4.55	1.20	1.24	0.46	4.95	1.02	2.70E-02	1.97E-01	8.78E-01	8.74E-01	9.55E-01	1.58E-01	9.02E-01	lipid A biosynthesis lauroyl acyltransferase [EC:2.3.1.-]
KO:K02518	4.44	2.96	4.81	3.82	2.56	4.81	3.62	3.68E-01	6.47E-01	3.10E-01	4.77E-01	7.31E-01	3.10E-01	5.17E-01	translation initiation factor IF-1
KO:K02519	11.47	12.51	16.11	8.49	8.68	6.07	5.61	2.34E-01	1.31E-01	1.51E-02	7.86E-01	7.58E-01	9.52E-01	9.64E-01	translation initiation factor IF-2
KO:K02520	8.23	6.72	15.87	5.72	6.40	3.76	2.92	2.39E-01	4.89E-01	1.21E-02	6.85E-01	5.49E-01	9.08E-01	9.49E-01	translation initiation factor IF-3
KO:K02521	1.14	0.00	0.00	0.08	0.00	0.00	0.20	7.31E-02	5.59E-01	1.00E+00	4.04E-01	1.00E+00	1.00E+00	2.77E-01	LysR family transcriptional regulator, positive regulator for ilvC
KO:K02523	4.90	10.78	7.35	5.37	0.76	0.54	2.16	4.54E-01	3.84E-02	1.41E-01	3.67E-01	9.72E-01	9.79E-01	8.93E-01	octaprenyl diphosphate synthase [EC:2.5.1.-]
KO:K02527	2.71	3.94	7.40	1.91	0.23	0.94	0.94	3.43E-01	1.38E-01	2.99E-02	5.97E-01	9.76E-01	8.79E-01	8.79E-01	3-deoxy-D-manno-octulosonic-acid transferase [EC:2.-.-.-]
KO:K02528	3.42	1.91	5.21	1.44	7.37	0.03	0.29	1.69E-01	5.45E-01	6.29E-02	7.15E-01	2.59E-02	9.96E-01	9.65E-01	dimethyladenosine transferase [EC:2.1.1.-]
KO:K02529	1.54	11.39	5.59	1.87	0.92	0.20	0.09	7.05E-01	1.67E-02	1.12E-01	6.28E-01	8.39E-01	9.48E-01	9.68E-01	LacI family transcriptional regulator
KO:K02533	5.51	5.23	0.80	1.40	1.21	0.13	0.76	7.27E-02	8.27E-02	8.61E-01	7.20E-01	7.74E-01	9.62E-01	8.68E-01	tRNA/rRNA methyltransferase [EC:2.1.1.-]
KO:K02535	13.29	12.18	8.87	5.08	1.40	6.85	5.30	5.61E-02	8.61E-02	3.22E-01	8.70E-01	9.88E-01	6.68E-01	8.54E-01	UDP-3-O-[3-hydroxymyristoyl] N-acetylglucosamine deacetylase
KO:K02536	9.37	8.14	6.77	5.02	3.01	5.34	4.43	1.16E-01	2.05E-01	4.01E-01	7.42E-01	9.33E-01	6.90E-01	8.25E-01	UDP-3-O-[3-hydroxymyristoyl] glucosamine N-acyltransferase
KO:K02542	0.00	0.00	0.00	0.00	0.00	0.00	2.50	1.00E+00	1.00E+00	1.00E+00	1.00E+00	1.00E+00	1.00E+00	2.44E-02	minichromosome maintenance protein 6
KO:K02548	0.33	0.10	2.65	0.47	7.79	2.09	0.38	7.02E-01	8.35E-01	1.03E-01	6.30E-01	1.51E-02	1.50E-01	6.73E-01	1,4-dihydroxy-2-naphthoate octaprenyltransferase [EC:2.5.1.74]
KO:K02549	0.44	0.00	0.26	0.65	2.89	0.00	0.00	2.82E-01	1.00E+00	3.70E-01	2.19E-01	5.93E-02	1.00E+00	1.00E+00	O-succinylbenzoate synthase [EC:4.2.1.113]
KO:K02551	0.54	0.56	2.73	0.77	2.46	3.93	0.00	6.13E-01	6.04E-01	1.26E-01	5.04E-01	1.48E-01	6.89E-02	1.00E+00	2-succinyl-5-enolpyruvyl-6-hydroxy-3-cyclohexene-1-carboxylate
KO:K02552	0.00	0.00	5.36	0.02	8.37	0.00	0.00	6.45E-01	1.00E+00	1.37E-02	6.14E-01	1.57E-03	1.00E+00	1.00E+00	menaquinone-specific isochorismate synthase [EC:5.4.4.2]
KO:K02553	4.32	0.35	0.26	0.62	0.00	1.87	0.57	7.23E-02	6.59E-01	6.96E-01	5.53E-01	1.00E+00	2.22E-01	5.75E-01	regulator of ribonuclease activity A
KO:K02554	1.86	0.00	0.00	0.33	0.00	0.00	0.04	7.47E-02	1.00E+00	1.00E+00	2.31E-01	1.00E+00	1.00E+00	4.07E-01	2-keto-4-pentenoate hydratase [EC:4.2.1.8]
KO:K02556	9.58	1.09	9.49	2.16	0.04	1.92	1.05	4.80E-02	8.20E-01	4.91E-02	6.09E-01	9.44E-01	6.60E-01	8.24E-01	chemotaxis protein MotA
KO:K02557	7.98	6.67	1.31	3.37	0.00	13.25	0.22	8.39E-02	1.37E-01	8.90E-01	5.68E-01	9.96E-01	1.77E-02	9.76E-01	chemotaxis protein MotB
KO:K02558	8.12	0.47	5.82	1.13	0.77	7.71	0.48	3.14E-02	9.13E-01	8.14E-02	8.06E-01	8.73E-01	3.81E-02	9.12E-01	UDP-N-acetylmuramate: L-alanyl-gamma-D-glutamyl-meso-diaminopimelate
KO:K02560	0.47	0.00	0.00	0.00	0.00	0.00	0.00	6.63E-02	1.00E+00	1.00E+00	1.00E+00	1.00E+00	1.00E+00	1.00E+00	lipid A biosynthesis (KDO)2-(lauroyl)-lipid iva acyltransferase
KO:K02563	3.57	5.85	4.84	2.33	2.89	3.48	0.81	4.14E-01	1.06E-01	1.84E-01	7.53E-01	5.99E-01	4.37E-01	9.62E-01	UDP-N-acetylglucosamine--N-acetylmuramyl-(pentapeptide)
KO:K02564	1.01	0.11	13.69	0.48	11.68	0.23	0.03	5.54E-01	8.68E-01	6.57E-03	7.49E-01	1.13E-02	8.28E-01	9.06E-01	glucosamine-6-phosphate deaminase [EC:3.5.99.6]
KO:K02566	0.00	0.00	0.21	0.01	0.84	0.00	1.50	5.12E-01	1.00E+00	2.15E-01	4.91E-01	7.23E-02	1.00E+00	3.59E-02	NagD protein
KO:K02567	0.00	0.05	0.71	0.02	0.00	0.00	0.08	1.00E+00	3.09E-01	3.60E-02	3.96E-01	1.00E+00	1.00E+00	2.59E-01	periplasmic nitrate reductase NapA [EC:1.7.99.4]
KO:K02569	0.00	0.01	0.00	0.00	0.00	0.00	0.74	1.00E+00	2.36E-01	1.00E+00	1.00E+00	1.00E+00	1.00E+00	2.76E-02	cytochrome c-type protein NapC
KO:K02573	0.04	0.04	0.00	0.03	0.00	0.00	0.00	1.85E-01	2.18E-01	1.00E+00	2.49E-01	1.00E+00	1.00E+00	1.00E+00	ferredoxin-type protein NapG
KO:K02574	0.04	0.04	0.00	0.03	0.00	0.00	0.00	1.77E-01	2.11E-01	1.00E+00	2.42E-01	1.00E+00	1.00E+00	1.00E+00	ferredoxin-type protein NapH
KO:K02575	0.02	0.01	0.00	0.00	16.00	0.00	0.01	6.89E-01	7.33E-01	1.00E+00	7.35E-01	7.14E-04	1.00E+00	7.24E-01	MFS transporter, NNP family, nitrate/nitrite transporter
KO:K02584	0.12	0.00	0.39	0.09	0.62	0.00	3.35	4.95E-01	7.04E-01	2.77E-01	5.41E-01	1.96E-01	1.00E+00	2.23E-02	Nif-specific regulatory protein
KO:K02585	0.00	0.00	0.63	0.00	0.00	0.00	0.00	1.00E+00	1.00E+00	1.64E-02	1.00E+00	1.00E+00	1.00E+00	1.00E+00	nitrogen fixation protein NifB
KO:K02589	0.00	0.00	0.00	0.00	0.00	0.00	0.83	1.00E+00	1.00E+00	1.00E+00	1.00E+00	1.00E+00	1.00E+00	2.84E-02	nitrogen regulatory protein PII 1
KO:K02600	11.31	10.46	16.85	9.64	4.64	10.63	4.89	2.06E-01	3.33E-01	1.19E-02	4.96E-01	9.81E-01	3.05E-01	9.76E-01	N utilization substance protein A
KO:K02601	10.57	10.92	10.63	8.30	5.83	6.29	5.38	2.15E-01	1.80E-01	2.09E-01	5.75E-01	8.92E-01	8.59E-01	9.15E-01	transcriptional antiterminator NusG
KO:K02609	1.36	7.27	0.00	1.59	0.00	1.12	0.00	4.19E-01	3.03E-02	1.00E+00	3.55E-01	1.00E+00	4.98E-01	1.00E+00	phenylacetic acid degradation protein
KO:K02610	0.17	1.72	0.00	0.31	0.00	3.86	0.00	6.35E-01	8.97E-02	1.00E+00	5.00E-01	1.00E+00	2.61E-02	1.00E+00	phenylacetic acid degradation protein
KO:K02611	1.15	4.51	0.00	1.32	0.00	0.00	0.00	2.83E-01	4.06E-02	1.00E+00	2.38E-01	1.00E+00	1.00E+00	1.00E+00	phenylacetic acid degradation protein
KO:K02612	0.81	1.49	0.00	0.57	0.00	0.24	0.00	2.15E-01	1.04E-01	1.00E+00	2.98E-01	1.00E+00	5.10E-01	1.00E+00	phenylacetic acid degradation protein
KO:K02613	2.44	3.86	0.00	2.62	0.00	6.06	0.00	2.64E-01	1.16E-01	1.00E+00	2.33E-01	1.00E+00	4.94E-02	1.00E+00	phenylacetic acid degradation NADH oxidoreductase
KO:K02614	0.74	1.22	0.00	0.39	0.00	0.29	0.00	2.12E-01	1.21E-01	1.00E+00	3.32E-01	1.00E+00	3.87E-01	1.00E+00	phenylacetic acid degradation protein
KO:K02616	0.41	1.41	0.00	0.00	1.16	0.00	0.00	2.56E-01	8.00E-02	1.00E+00	1.00E+00	9.67E-02	1.00E+00	1.00E+00	phenylacetic acid degradation operon negative regulatory protein
KO:K02617	0.00	0.00	0.00	0.82	0.00	0.00	0.00	1.00E+00	1.00E+00	1.00E+00	6.01E-02	1.00E+00	1.00E+00	1.00E+00	phenylacetic acid degradation protein
KO:K02618	0.38	9.93	0.00	0.33	0.00	0.89	0.00	6.99E-01	1.03E-02	1.00E+00	7.17E-01	1.00E+00	4.52E-01	1.00E+00	phenylacetic acid degradation protein
KO:K02619	1.01	0.15	0.04	0.14	1.17	6.85	0.52	2.39E-01	7.61E-01	8.71E-01	7.73E-01	2.00E-01	1.26E-02	4.67E-01	4-amino-4-deoxychorismate lyase [EC:4.1.3.38]
KO:K02621	12.60	10.05	3.03	2.91	0.34	8.92	1.76	2.14E-02	6.29E-02	8.85E-01	8.97E-01	9.94E-01	9.93E-02	9.59E-01	topoisomerase IV subunit A [EC:5.99.1.-]
KO:K02622	9.74	7.93	7.04	4.69	0.67	4.77	5.12	8.59E-02	1.87E-01	2.89E-01	7.25E-01	9.89E-01	7.10E-01	6.48E-01	topoisomerase IV subunit B [EC:5.99.1.-]
KO:K02623	0.00	0.66	0.00	0.17	0.00	0.00	0.00	1.00E+00	6.54E-02	1.00E+00	2.04E-01	1.00E+00	1.00E+00	1.00E+00	LysR family transcriptional regulator, pca operon transcriptional
KO:K02624	2.30	0.04	0.00	0.00	0.00	0.00	0.00	4.11E-02	3.25E-01	1.00E+00	4.18E-01	1.00E+00	1.00E+00	1.00E+00	IclR family transcriptional regulator, pca regulon regulatory
KO:K02626	0.00	0.00	0.26	0.00	0.00	0.00	2.92	1.00E+00	1.00E+00	1.00E+00	1.00E+00	1.00E+00	1.00E+00	1.14E-02	arginine decarboxylase [EC:4.1.1.19]
KO:K02634	0.00	0.00	0.00	0.00	40.00	0.00	0.00	1.00E+00	1.00E+00	1.00E+00	1.00E+00	0.00E+00	1.00E+00	1.00E+00	apocytochrome f
KO:K02635	0.00	0.00	0.00	0.00	51.88	0.00	0.00	1.00E+00	1.00E+00	1.00E+00	1.00E+00	1.43E-04	1.00E+00	1.00E+00	cytochrome b6
KO:K02636	0.01	0.00	0.00	0.05	24.56	0.00	0.00	5.68E-01	1.00E+00	1.00E+00	5.19E-01	0.00E+00	1.00E+00	1.00E+00	cytochrome b6-f complex iron-sulfur subunit [EC:1.1.99.1]
KO:K02637	0.00	0.00	0.00	0.00	41.25	0.00	0.00	1.00E+00	1.00E+00	1.00E+00	1.00E+00	0.00E+00	1.00E+00	1.00E+00	cytochrome b6-f complex subunit 4
KO:K02638	0.00	0.00	0.00	0.00	12.50	0.00	0.00	1.00E+00	1.00E+00	1.00E+00	1.00E+00	1.71E-03	1.00E+00	1.00E+00	plastocyanin
KO:K02639	0.09	0.39	0.00	0.05	22.60	0.00	0.00	5.93E-01	4.48E-01	1.00E+00	6.24E-01	1.29E-03	1.00E+00	1.00E+00	ferredoxin
KO:K02640	0.00	0.00	0.00	0.00	7.50	0.00	0.00	1.00E+00	1.00E+00	1.00E+00	1.00E+00	2.71E-03	1.00E+00	1.00E+00	cytochrome b6-f complex subunit 5
KO:K02641	0.00	0.00	0.01	0.00	41.88	0.00	0.00	1.00E+00	1.00E+00	5.22E-01	1.00E+00	0.00E+00	1.00E+00	1.00E+00	ferredoxin--NADP+ reductase [EC:1.18.1.2]
KO:K02643	0.00	0.00	0.00	0.00	3.75	0.00	0.00	1.00E+00	1.00E+00	1.00E+00	1.00E+00	2.71E-03	1.00E+00	1.00E+00	cytochrome b6-f complex subunit 7
KO:K02650	4.40	0.05	0.36	2.90	3.23	7.17	0.10	1.06E-01	9.00E-01	8.25E-01	3.66E-01	1.82E-01	4.29E-02	8.83E-01	type IV pilus assembly protein PilA
KO:K02651	0.01	0.00	13.23	0.57	1.00	0.00	1.36	7.07E-01	1.00E+00	1.31E-02	3.73E-01	2.80E-01	1.00E+00	2.25E-01	pilus assembly protein Flp/PilA
KO:K02652	3.09	0.00	30.66	1.07	5.31	1.04	1.86	3.55E-01	9.54E-01	1.00E-03	7.84E-01	1.42E-01	7.89E-01	6.17E-01	type IV pilus assembly protein PilB
KO:K02653	3.27	0.00	14.97	1.16	11.04	0.06	1.87	2.76E-01	9.61E-01	6.86E-03	7.37E-01	2.09E-02	9.40E-01	5.53E-01	type IV pilus assembly protein PilC
KO:K02654	2.63	0.01	7.39	0.84	5.76	0.00	1.05	1.72E-01	9.45E-01	2.41E-02	6.43E-01	3.93E-02	1.00E+00	5.55E-01	leader peptidase (prepilin peptidase) / N-methyltransferase
KO:K02655	1.43	0.00	3.05	0.36	1.90	2.16	0.09	2.07E-01	8.75E-01	7.39E-02	5.57E-01	1.41E-01	1.20E-01	7.68E-01	type IV pilus assembly protein PilE
KO:K02656	10.50	0.08	0.10	0.29	0.00	1.91	0.08	1.20E-02	8.21E-01	8.08E-01	7.15E-01	1.00E+00	2.31E-01	8.21E-01	type IV pilus assembly protein PilF
KO:K02657	0.53	0.00	0.54	0.25	0.00	3.81	1.49	3.25E-01	1.00E+00	3.22E-01	4.68E-01	1.00E+00	3.60E-02	1.28E-01	twitching motility two-component system response regulator PilG
KO:K02658	0.78	0.00	0.40	0.34	0.00	0.00	0.71	1.70E-01	1.00E+00	2.77E-01	3.04E-01	1.00E+00	1.00E+00	1.83E-01	twitching motility two-component system response regulator PilH
KO:K02659	0.94	0.00	1.45	0.07	0.00	0.00	0.45	1.16E-01	1.00E+00	7.01E-02	5.46E-01	1.00E+00	1.00E+00	2.41E-01	twitching motility protein PilI
KO:K02660	1.67	0.00	1.07	0.39	0.00	0.16	0.79	1.02E-01	1.00E+00	1.75E-01	4.32E-01	1.00E+00	6.26E-01	2.45E-01	twitching motility protein PilJ
KO:K02661	0.00	0.00	0.00	0.13	0.00	0.00	0.24	1.00E+00	1.00E+00	1.00E+00	1.20E-01	1.00E+00	1.00E+00	8.70E-02	type IV pilus assembly protein PilK
KO:K02662	11.87	0.02	5.73	0.63	1.28	0.79	0.33	1.99E-02	8.93E-01	1.01E-01	7.62E-01	6.08E-01	7.28E-01	8.24E-01	type IV pilus assembly protein PilM
KO:K02663	7.73	0.00	0.73	0.99	0.01	0.00	1.14	2.87E-02	8.66E-01	5.38E-01	4.42E-01	8.47E-01	1.00E+00	3.98E-01	type IV pilus assembly protein PilN
KO:K02664	6.48	0.01	1.02	0.15	1.52	0.09	0.00	2.71E-02	8.75E-01	3.21E-01	7.43E-01	2.13E-01	7.90E-01	1.00E+00	type IV pilus assembly protein PilO
KO:K02665	7.76	0.03	0.00	0.14	0.00	3.15	0.27	1.83E-02	8.24E-01	1.00E+00	7.42E-01	1.00E+00	8.93E-02	6.73E-01	type IV pilus assembly protein PilP
KO:K02666	9.70	0.02	2.87	2.41	2.44	0.27	1.00	3.00E-02	9.23E-01	3.26E-01	4.10E-01	4.03E-01	8.78E-01	7.48E-01	type IV pilus assembly protein PilQ
KO:K02667	1.69	0.01	1.26	0.15	0.00	1.78	0.56	9.24E-02	8.49E-01	1.35E-01	6.79E-01	1.00E+00	8.64E-02	3.50E-01	two-component system, NtrC family, response regulator PilR
KO:K02668	3.88	0.00	0.00	0.15	0.00	0.00	0.00	1.61E-02	1.00E+00	1.00E+00	4.74E-01	1.00E+00	1.00E+00	1.00E+00	two-component system, NtrC family, sensor histidine kinase PilS
KO:K02669	2.67	0.01	17.80	0.97	14.98	1.91	2.22	4.58E-01	9.63E-01	6.71E-03	8.21E-01	1.14E-02	6.33E-01	5.61E-01	twitching motility protein PilT
KO:K02670	8.56	0.04	0.78	0.49	0.00	0.07	1.23	1.81E-02	8.53E-01	5.02E-01	6.27E-01	1.00E+00	8.32E-01	3.65E-01	twitching motility protein PilU
KO:K02671	5.75	0.06	0.27	0.20	0.00	0.00	0.00	2.20E-02	6.83E-01	5.08E-01	5.55E-01	1.00E+00	1.00E+00	1.00E+00	type IV pilus assembly protein PilV
KO:K02672	2.34	0.04	0.00	0.26	0.00	0.32	0.02	4.57E-02	6.90E-01	1.00E+00	4.09E-01	1.00E+00	3.73E-01	7.16E-01	type IV pilus assembly protein PilW
KO:K02673	0.55	0.06	0.00	0.19	0.00	0.00	0.00	7.47E-02	4.12E-01	1.00E+00	2.25E-01	1.00E+00	1.00E+00	1.00E+00	type IV pilus assembly protein PilX
KO:K02674	7.06	0.00	0.12	2.37	0.00	0.00	0.39	3.10E-02	8.33E-01	7.82E-01	1.65E-01	1.00E+00	1.00E+00	6.49E-01	type IV pilus assembly protein PilY1
KO:K02676	4.32	0.00	0.04	0.27	0.00	13.14	0.00	4.86E-02	8.38E-01	8.00E-01	6.46E-01	1.00E+00	7.14E-03	1.00E+00	type IV pilus assembly protein PilZ
KO:K02683	0.00	0.00	0.00	0.00	0.00	0.00	1.25	1.00E+00	1.00E+00	1.00E+00	1.00E+00	1.00E+00	1.00E+00	2.94E-02	DNA primase [EC:2.7.7.-]
KO:K02685	0.00	0.00	0.00	0.00	0.00	0.00	1.67	1.00E+00	1.00E+00	1.00E+00	1.00E+00	1.00E+00	1.00E+00	2.90E-02	DNA primase large subunit [EC:2.7.7.-]
KO:K02686	0.05	0.00	0.00	3.36	0.00	0.00	0.00	3.78E-01	1.00E+00	1.00E+00	5.53E-02	1.00E+00	1.00E+00	1.00E+00	primosomal replication protein N
KO:K02687	3.10	7.78	2.62	1.04	8.79	1.67	0.02	4.14E-01	3.31E-02	5.63E-01	9.15E-01	2.19E-02	8.26E-01	9.96E-01	ribosomal protein L11 methyltransferase [EC:2.1.1.-]
KO:K02688	0.81	0.00	0.00	0.00	0.00	0.00	0.00	6.61E-02	1.00E+00	1.00E+00	1.00E+00	1.00E+00	1.00E+00	1.00E+00	transcriptional regulator, propionate catabolism operon regulatory
KO:K02689	0.00	0.00	0.00	0.00	61.88	0.00	0.00	1.00E+00	1.00E+00	1.00E+00	1.00E+00	0.00E+00	1.00E+00	1.00E+00	photosystem I core protein Ia
KO:K02690	0.00	0.00	0.00	0.00	55.63	0.00	0.00	1.00E+00	1.00E+00	1.00E+00	1.00E+00	0.00E+00	1.00E+00	1.00E+00	photosystem I core protein Ib
KO:K02691	0.00	0.00	0.00	0.00	5.00	0.00	0.00	1.00E+00	1.00E+00	1.00E+00	1.00E+00	2.29E-03	1.00E+00	1.00E+00	photosystem I subunit VII
KO:K02692	0.00	0.00	0.00	0.00	27.50	0.00	0.00	1.00E+00	1.00E+00	1.00E+00	1.00E+00	1.43E-04	1.00E+00	1.00E+00	photosystem I subunit II

KO:K02693	0.00	0.00	0.00	0.00	25.63	0.00	0.00	1.00E+00	1.00E+00	1.00E+00	1.00E+00	0.00E+00	1.00E+00	1.00E+00	photosystem I subunit IV
KO:K02694	0.00	0.00	0.00	0.00	32.50	0.00	0.00	1.00E+00	1.00E+00	1.00E+00	1.00E+00	0.00E+00	1.00E+00	1.00E+00	photosystem I subunit III
KO:K02696	0.00	0.00	0.00	0.00	7.50	0.00	0.00	1.00E+00	1.00E+00	1.00E+00	1.00E+00	2.43E-03	1.00E+00	1.00E+00	photosystem I subunit VIII
KO:K02697	0.00	0.00	0.00	0.00	16.88	0.00	0.00	1.00E+00	1.00E+00	1.00E+00	1.00E+00	2.86E-04	1.00E+00	1.00E+00	photosystem I subunit IX
KO:K02698	0.00	0.00	0.00	0.00	15.63	0.00	0.00	1.00E+00	1.00E+00	1.00E+00	1.00E+00	1.43E-04	1.00E+00	1.00E+00	photosystem I subunit X
KO:K02699	0.00	0.00	0.00	0.00	35.63	0.00	0.00	1.00E+00	1.00E+00	1.00E+00	1.00E+00	0.00E+00	1.00E+00	1.00E+00	photosystem I subunit XI
KO:K02703	0.00	0.00	0.00	0.00	60.63	0.00	0.00	1.00E+00	1.00E+00	1.00E+00	1.00E+00	1.43E-04	1.00E+00	1.00E+00	photosystem II reaction center D1 protein
KO:K02704	0.00	0.00	0.00	0.00	57.50	0.00	0.00	1.00E+00	1.00E+00	1.00E+00	1.00E+00	0.00E+00	1.00E+00	1.00E+00	photosystem II CP47 chlorophyll apoprotein
KO:K02705	0.00	0.00	0.00	0.00	56.25	0.00	0.00	1.00E+00	1.00E+00	1.00E+00	1.00E+00	0.00E+00	1.00E+00	1.00E+00	photosystem II CP43 chlorophyll apoprotein
KO:K02706	0.00	0.00	0.00	0.00	43.13	0.00	0.00	1.00E+00	1.00E+00	1.00E+00	1.00E+00	1.43E-04	1.00E+00	1.00E+00	photosystem II reaction center D2 protein
KO:K02707	0.00	0.00	0.00	0.00	34.38	0.00	0.00	1.00E+00	1.00E+00	1.00E+00	1.00E+00	0.00E+00	1.00E+00	1.00E+00	photosystem II cytochrome b559 subunit alpha
KO:K02708	0.00	0.00	0.00	0.00	22.50	0.00	0.00	1.00E+00	1.00E+00	1.00E+00	1.00E+00	0.00E+00	1.00E+00	1.00E+00	photosystem II cytochrome b559 subunit beta
KO:K02709	0.00	0.00	0.00	0.00	21.25	0.00	0.00	1.00E+00	1.00E+00	1.00E+00	1.00E+00	1.43E-04	1.00E+00	1.00E+00	photosystem II PsbH protein
KO:K02710	0.00	0.00	0.00	0.00	5.63	0.00	0.00	1.00E+00	1.00E+00	1.00E+00	1.00E+00	1.49E-02	1.00E+00	1.00E+00	photosystem II PsbI protein
KO:K02711	0.00	0.00	0.00	0.00	30.00	0.00	0.00	1.00E+00	1.00E+00	1.00E+00	1.00E+00	0.00E+00	1.00E+00	1.00E+00	photosystem II PsbJ protein
KO:K02712	0.00	0.00	0.00	0.00	9.38	0.00	0.00	1.00E+00	1.00E+00	1.00E+00	1.00E+00	0.00E+00	1.00E+00	1.00E+00	photosystem II PsbK protein
KO:K02713	0.00	0.00	0.00	0.00	15.63	0.00	0.00	1.00E+00	1.00E+00	1.00E+00	1.00E+00	1.86E-03	1.00E+00	1.00E+00	photosystem II PsbL protein
KO:K02714	0.00	0.00	0.00	0.00	14.38	0.00	0.00	1.00E+00	1.00E+00	1.00E+00	1.00E+00	0.00E+00	1.00E+00	1.00E+00	photosystem II PsbM protein
KO:K02715	0.00	0.00	0.00	0.00	10.63	0.00	0.00	1.00E+00	1.00E+00	1.00E+00	1.00E+00	1.29E-03	1.00E+00	1.00E+00	PsbN protein
KO:K02716	0.00	0.00	0.00	0.00	35.00	0.00	0.00	1.00E+00	1.00E+00	1.00E+00	1.00E+00	5.71E-04	1.00E+00	1.00E+00	photosystem II oxygen-evolving enhancer protein 1
KO:K02717	0.00	0.00	0.00	0.00	28.13	0.00	0.00	1.00E+00	1.00E+00	1.00E+00	1.00E+00	1.43E-04	1.00E+00	1.00E+00	photosystem II oxygen-evolving enhancer protein 2
KO:K02718	0.00	0.00	0.00	0.00	2.50	0.00	0.00	1.00E+00	1.00E+00	1.00E+00	1.00E+00	1.61E-02	1.00E+00	1.00E+00	photosystem II PsbT protein
KO:K02719	0.00	0.00	0.00	0.00	35.63	0.00	0.00	1.00E+00	1.00E+00	1.00E+00	1.00E+00	0.00E+00	1.00E+00	1.00E+00	photosystem II PsbU protein
KO:K02720	0.00	0.00	0.00	0.00	30.63	0.00	0.00	1.00E+00	1.00E+00	1.00E+00	1.00E+00	1.43E-04	1.00E+00	1.00E+00	photosystem II cytochrome c55
KO:K02722	0.00	0.00	0.00	0.00	15.00	0.00	0.00	1.00E+00	1.00E+00	1.00E+00	1.00E+00	2.71E-03	1.00E+00	1.00E+00	photosystem II PsbX protein
KO:K02723	0.00	0.00	0.00	0.00	15.00	0.00	0.00	1.00E+00	1.00E+00	1.00E+00	1.00E+00	1.71E-03	1.00E+00	1.00E+00	photosystem II PsbY protein
KO:K02724	0.00	0.00	0.00	0.00	28.75	0.00	0.00	1.00E+00	1.00E+00	1.00E+00	1.00E+00	1.43E-04	1.00E+00	1.00E+00	photosystem II PsbZ protein
KO:K02742	1.63	0.01	0.00	0.04	0.00	0.00	0.00	3.40E-02	5.12E-01	1.00E+00	4.30E-01	1.00E+00	1.00E+00	1.00E+00	SprT protein
KO:K02768	0.00	0.00	7.58	0.10	2.81	0.00	0.23	1.00E+00	1.00E+00	1.34E-02	4.23E-01	7.40E-02	1.00E+00	3.62E-01	PTS system, fructose-specific IIA component [EC:2.7.1.69]
KO:K02769	0.00	0.00	0.00	0.61	0.00	0.00	0.00	1.00E+00	1.00E+00	1.00E+00	1.43E-01	1.00E+00	1.00E+00	1.00E+00	PTS system, fructose-specific IIB component [EC:2.7.1.69]
KO:K02770	0.00	0.00	2.14	0.45	0.00	0.00	0.00	1.00E+00	1.00E+00	4.03E-02	1.66E-01	1.00E+00	1.00E+00	1.00E+00	PTS system, fructose-specific IIC component
KO:K02784	0.00	0.00	0.00	0.00	0.94	0.00	0.10	1.00E+00	1.00E+00	1.00E+00	1.00E+00	2.19E-02	1.00E+00	1.45E-01	phosphocarrier protein HPr
KO:K02793	0.82	19.82	0.00	0.17	0.00	0.00	0.00	6.19E-01	1.57E-03	1.00E+00	8.56E-01	1.00E+00	1.00E+00	1.00E+00	PTS system, mannose-specific IIA component [EC:2.7.1.69]
KO:K02800	0.00	0.00	0.00	2.04	0.00	0.00	0.00	1.00E+00	1.00E+00	1.00E+00	1.43E-01	1.00E+00	1.00E+00	1.00E+00	PTS system, mannitol-specific IIC component
KO:K02805	1.46	0.03	0.26	0.38	0.00	0.00	1.30	1.37E-01	6.56E-01	4.45E-01	3.75E-01	1.00E+00	1.00E+00	1.52E-01	lipopolysaccharide biosynthesis protein
KO:K02806	3.15	11.73	1.10	2.59	0.02	0.97	1.42	4.22E-01	1.59E-02	8.66E-01	5.57E-01	9.88E-01	8.82E-01	8.20E-01	PTS system, nitrogen regulatory IIA component [EC:2.7.1.69]
KO:K02821	0.35	0.00	0.00	0.00	0.00	0.00	0.00	4.93E-02	1.00E+00	1.00E+00	1.00E+00	1.00E+00	1.00E+00	1.00E+00	PTS system, ascorbate-specific IIA component [EC:2.7.1.69]
KO:K02823	0.23	0.00	1.81	0.10	0.00	0.00	11.34	5.04E-01	6.78E-01	1.45E-01	5.98E-01	1.00E+00	1.00E+00	9.43E-03	dihydroorotate dehydrogenase electron transfer subunit
KO:K02824	0.00	2.35	0.00	0.00	0.00	0.00	0.00	1.00E+00	4.41E-02	1.00E+00	3.91E-01	1.00E+00	1.00E+00	1.00E+00	uracil permease
KO:K02825	2.33	0.96	0.36	1.92	6.74	2.52	0.05	2.99E-01	6.56E-01	8.32E-01	3.80E-01	4.39E-02	2.68E-01	9.00E-01	pyrimidine operon attenuation protein / uracil
KO:K02834	4.07	2.39	5.53	2.59	8.19	2.68	0.04	2.55E-01	6.29E-01	1.23E-01	5.79E-01	4.23E-02	5.57E-01	9.90E-01	ribosome-binding factor A
KO:K02835	5.65	7.25	9.58	2.84	4.43	5.86	0.59	3.02E-01	1.30E-01	5.00E-02	8.49E-01	5.55E-01	2.68E-01	9.83E-01	peptide chain release factor RF-1
KO:K02836	8.90	5.74	12.73	5.25	5.66	1.35	0.96	1.01E-01	4.45E-01	2.39E-02	5.52E-01	4.62E-01	9.73E-01	9.84E-01	peptide chain release factor RF-2
KO:K02837	9.41	11.05	0.42	1.87	6.78	4.34	1.69	6.64E-02	3.66E-02	9.87E-01	9.32E-01	1.94E-01	6.05E-01	9.44E-01	peptide chain release factor RF-3
KO:K02838	7.21	6.81	11.20	4.49	5.66	1.40	1.40	1.80E-01	2.22E-01	3.01E-02	6.77E-01	4.08E-01	9.75E-01	9.75E-01	ribosome recycling factor
KO:K02839	0.00	0.06	0.00	0.05	0.00	0.00	0.00	1.00E+00	1.45E-01	1.00E+00	1.87E-01	1.00E+00	1.00E+00	1.00E+00	peptide chain release factor RF-H
KO:K02841	1.16	0.00	1.20	1.09	0.00	0.00	0.53	1.83E-01	7.84E-01	1.76E-01	1.94E-01	1.00E+00	1.00E+00	3.38E-01	heptosyltransferase I [EC:2.4.-.-]
KO:K02843	0.91	0.04	6.03	0.94	0.13	1.49	0.13	3.63E-01	8.58E-01	2.04E-02	3.51E-01	7.83E-01	2.07E-01	7.83E-01	heptosyltransferase II [EC:2.4.-.-]
KO:K02844	0.38	0.00	0.03	0.23	0.48	1.92	0.05	2.64E-01	6.97E-01	6.09E-01	3.55E-01	2.17E-01	6.43E-02	5.49E-01	UDP-glucose:(heptosyl)LPS alpha-1,3-glucosyltransferase [EC:2.4.1.-]
KO:K02848	0.16	0.00	0.00	0.04	0.00	5.26	0.00	2.72E-01	1.00E+00	1.00E+00	3.74E-01	1.00E+00	7.29E-03	1.00E+00	heptose (I) phosphotransferase [EC:2.7.1.-]
KO:K02849	0.02	0.00	0.00	0.00	0.00	0.00	2.70	2.35E-01	1.00E+00	1.00E+00	1.00E+00	1.00E+00	1.00E+00	3.04E-02	heptosyltransferase III [EC:2.4.-.-]
KO:K02851	1.78	0.27	3.39	1.85	7.63	5.68	0.40	4.79E-01	8.46E-01	2.25E-01	4.62E-01	5.64E-02	1.02E-01	8.24E-01	undecaprenyl-phosphate alpha-N-acetylglucosaminyltransferase
KO:K02852	0.02	0.00	0.00	0.00	24.25	0.00	0.00	6.01E-01	6.28E-01	1.00E+00	6.29E-01	0.00E+00	1.00E+00	1.00E+00	UDP-N-acetyl-D-mannosaminuronic acid transferase [EC:2.4.1.-]
KO:K02854	0.00	0.00	0.63	0.00	0.00	0.00	0.00	1.00E+00	1.00E+00	1.47E-02	1.00E+00	1.00E+00	1.00E+00	1.00E+00	AraC family transcriptional regulator, L-rhamnose operon
KO:K02855	0.00	0.00	0.38	0.00	0.00	0.00	0.17	1.00E+00	1.00E+00	3.07E-02	1.00E+00	1.00E+00	1.00E+00	8.54E-02	AraC family transcriptional regulator, L-rhamnose operon regulatory
KO:K02856	0.00	1.00	0.00	0.00	0.00	0.00	0.00	1.00E+00	6.87E-02	1.00E+00	1.00E+00	1.00E+00	1.00E+00	1.00E+00	L-rhamnose-H+ transport protein
KO:K02858	7.45	11.72	7.30	4.45	5.39	1.74	1.42	2.38E-01	4.54E-02	2.53E-01	7.22E-01	5.51E-01	9.56E-01	9.69E-01	3,4-dihydroxy 2-butanone 4-phosphate synthase [EC:4.1.99.12]
KO:K02860	19.19	6.33	0.01	7.73	1.18	1.01	1.92	4.14E-03	3.10E-01	9.99E-01	1.63E-01	9.65E-01	9.71E-01	9.35E-01	16S rRNA processing protein RimM
KO:K02862	0.00	0.00	14.38	0.00	0.00	0.00	0.00	1.00E+00	1.00E+00	3.14E-03	1.00E+00	1.00E+00	1.00E+00	1.00E+00	putative membrane protein
KO:K02863	11.39	14.67	10.14	9.08	5.96	4.94	8.51	2.01E-01	2.49E-02	4.37E-01	7.00E-01	9.70E-01	9.86E-01	8.08E-01	large subunit ribosomal protein L1
KO:K02864	9.97	15.82	7.39	8.57	9.91	0.28	5.69	2.73E-01	2.13E-02	7.29E-01	5.09E-01	2.79E-01	1.00E+00	8.98E-01	large subunit ribosomal protein L1
KO:K02866	0.00	0.00	0.00	0.00	0.00	0.00	4.17	1.00E+00	1.00E+00	1.00E+00	1.00E+00	1.00E+00	1.00E+00	2.50E-02	large subunit ribosomal protein L1e
KO:K02867	8.73	7.51	6.69	7.57	8.26	2.97	3.06	1.98E-01	3.57E-01	5.05E-01	3.48E-01	2.52E-01	9.43E-01	9.40E-01	large subunit ribosomal protein L11
KO:K02869	0.00	0.00	0.00	0.00	0.00	0.00	3.75	1.00E+00	1.00E+00	1.00E+00	1.00E+00	1.00E+00	1.00E+00	2.73E-02	large subunit ribosomal protein L12
KO:K02871	7.89	8.84	9.20	4.93	10.81	9.92	4.26	4.14E-01	2.75E-01	2.35E-01	8.67E-01	1.20E-01	1.73E-01	9.09E-01	large subunit ribosomal protein L13
KO:K02874	9.21	3.37	8.17	8.12	6.72	0.66	5.83	1.23E-01	8.82E-01	1.94E-01	1.99E-01	3.67E-01	9.88E-01	5.23E-01	large subunit ribosomal protein L14
KO:K02876	6.60	10.97	10.92	5.02	7.24	5.58	3.47	5.80E-01	7.26E-02	7.53E-02	8.40E-01	4.48E-01	7.70E-01	9.43E-01	large subunit ribosomal protein L15
KO:K02877	0.00	0.00	0.00	0.00	0.00	0.00	5.42	1.00E+00	1.00E+00	1.00E+00	1.00E+00	1.00E+00	1.00E+00	1.71E-02	large subunit ribosomal protein L15e
KO:K02878	9.06	7.67	10.28	8.34	6.58	0.04	3.75	1.59E-01	3.06E-01	9.37E-02	2.19E-01	4.96E-01	9.99E-01	9.04E-01	large subunit ribosomal protein L16
KO:K02879	7.99	4.84	13.67	4.97	7.74	3.81	1.93	1.76E-01	7.16E-01	1.41E-02	6.90E-01	1.97E-01	8.59E-01	9.67E-01	large subunit ribosomal protein L17
KO:K02881	7.40	6.53	6.68	4.70	4.93	1.40	4.19	1.70E-01	2.69E-01	2.48E-01	6.37E-01	5.86E-01	9.72E-01	7.37E-01	large subunit ribosomal protein L18
KO:K02883	0.00	0.00	0.00	0.00	0.00	0.00	2.92	1.00E+00	1.00E+00	1.00E+00	1.00E+00	1.00E+00	1.00E+00	1.67E-02	large subunit ribosomal protein L18e
KO:K02884	8.69	11.20	6.33	5.88	4.84	6.80	3.38	2.58E-01	9.29E-02	6.29E-01	7.03E-01	8.30E-01	5.50E-01	9.29E-01	large subunit ribosomal protein L19
KO:K02885	0.00	0.00	0.00	0.00	0.00	0.00	3.33	1.00E+00	1.00E+00	1.00E+00	1.00E+00	1.00E+00	1.00E+00	1.83E-02	large subunit ribosomal protein L19e
KO:K02886	12.93	12.27	12.72	8.22	5.14	5.84	6.04	8.76E-02	1.20E-01	9.63E-02	7.38E-01	9.58E-01	9.36E-01	9.29E-01	large subunit ribosomal protein L2
KO:K02887	8.83	6.71	6.54	6.74	11.54	4.61	2.52	2.08E-01	5.06E-01	5.41E-01	5.01E-01	7.10E-02	8.37E-01	9.58E-01	large subunit ribosomal protein L2
KO:K02888	8.85	15.70	7.74	7.25	7.03	6.26	2.98	4.23E-01	3.30E-02	6.14E-01	6.99E-01	7.33E-01	8.29E-01	9.75E-01	large subunit ribosomal protein L21
KO:K02889	0.00	0.00	0.00	0.00	0.00	0.00	4.17	1.00E+00	1.00E+00	1.00E+00	1.00E+00	1.00E+00	1.00E+00	1.69E-02	large subunit ribosomal protein L21e
KO:K02890	8.01	11.05	5.27	5.52	6.49	5.27	3.13	2.75E-01	6.99E-02	7.71E-01	7.30E-01	5.41E-01	7.71E-01	9.46E-01	large subunit ribosomal protein L22
KO:K02892	7.53	6.91	5.39	5.15	6.33	4.26	2.56	2.15E-01	2.85E-01	5.51E-01	5.99E-01	3.73E-01	7.54E-01	9.19E-01	large subunit ribosomal protein L23
KO:K02894	0.00	0.00	0.00	0.00	0.00	0.00	1.67	1.00E+00	1.00E+00	1.00E+00	1.00E+00	1.00E+00	1.00E+00	2.74E-02	large subunit ribosomal protein L23e
KO:K02895	8.15	7.44	8.44	5.50	5.36	0.27	5.06	1.75E-01	2.42E-01	1.56E-01	5.64E-01	5.94E-01	9.95E-01	6.52E-01	large subunit ribosomal protein L24
KO:K02896	0.00	0.00	0.00	0.00	0.00	0.00	7.08	1.00E+00	1.00E+00	1.00E+00	1.00E+00	1.00E+00	1.00E+00	4.29E-03	large subunit ribosomal protein L24e
KO:K02897	10.05	13.04	6.16	9.46	0.89	5.43	5.11	1.93E-01	6.21E-02	7.66E-01	2.53E-01	9.94E-01	8.47E-01	8.71E-01	large subunit ribosomal protein L25
KO:K02899	9.62	7.93	6.97	6.40	5.91	4.15	5.91	1.78E-01	3.60E-01	5.11E-01	6.11E-01	6.92E-01	8.86E-01	6.92E-01	large subunit ribosomal protein L27
KO:K02902	7.93	8.19	6.86	4.79	8.12	0.26	5.70	2.16E-01	1.91E-01	3.45E-01	7.06E-01	1.98E-01	9.93E-01	5.45E-01	large subunit ribosomal protein L28
KO:K02904	3.89	5.28	9.47	3.05	6.54	0.00	2.39	4.64E-01	2.33E-01	4.97E-02	6.53E-01	1.34E-01	1.00E+00	7.81E-01	large subunit ribosomal protein L29
KO:K02906	11.71	10.89	12.48	8.43	9.58	7.85	5.55	1.62E-01	2.59E-01	1.06E-01	7.56E-01	5.21E-01	8.30E-01	9.56E-01	large subunit ribosomal protein L3
KO:K02907	9.68	7.17	0.00	4.71	0.00	0.96	1.60	3.33E-02	8.00E-02	1.00E+00	2.44E-01	1.00E+00	9.06E-01	8.35E-01	large subunit ribosomal protein L3
KO:K02908	0.00	0.00	0.00	0.00	0.00	0.00	3.75	1.00E+00	1.00E+00	1.00E+00	1.00E+00	1.00E+00	1.00E+00	1.77E-02	large subunit ribosomal protein L3e
KO:K02909	4.94	8.57	8.63	3.04	4.01	1.70	0.79	3.84E-01	7.49E-02	7.30E-02	7.55E-01	5.69E-01	9.03E-01	9.58E-01	large subunit ribosomal protein L31
KO:K02911	7.88	8.31	9.53	3.62	1.18	1.94	1.48	1.37E-01	1.17E-01	8.00E-02	7.21E-01	9.43E-01	9.04E-01	9.28E-01	large subunit ribosomal protein L32
KO:K02912	0.00	0.00	0.00	0.00	0.00	0.00	3.75	1.00E+00	1.00E+00	1.00E+00	1.00E+00	1.00E+00	1.00E+00	1.69E-02	large subunit ribosomal protein L32e
KO:K02913	5.13	1.97	3.86	3.68	10.15	6.56	1.04	2.81E-01	8.23E-01	4.87E-01	5.24E-01	4.91E-02	1.48E-01	9.12E-01	large subunit ribosomal protein L33
KO:K02914	1.56	3.82	1.31	2.66	0.70	0.04	1.37	5.17E-01	1.46E-01	5.89E-01	2.73E-01	7.72E-01	9.51E-01	5.70E-01	large subunit ribosomal protein L34
KO:K02916	5.05	5.28	4.49	4.36	7.12	2.87	2.68	4.03E-01	3.59E-01	5.06E-01	5.34E-01	1.57E-01	8.06E-01	8.29E-01	large subunit ribosomal protein L35

KO:K02919	1.63	3.18	7.09	3.52	3.09	2.44	0.67	7.38E-01	3.95E-01	8.11E-02	3.36E-01	4.12E-01	5.58E-01	8.89E-01	large subunit ribosomal protein L36
KO:K02921	0.00	0.00	0.00	0.00	0.00	0.00	2.92	1.00E+00	1.00E+00	1.00E+00	1.00E+00	1.00E+00	1.00E+00	2.34E-02	large subunit ribosomal protein L37Ae
KO:K02922	0.00	0.00	0.00	0.00	0.00	0.00	4.17	1.00E+00	1.00E+00	1.00E+00	1.00E+00	1.00E+00	1.00E+00	2.66E-02	large subunit ribosomal protein L37e
KO:K02924	0.00	0.00	0.00	0.00	0.00	0.00	2.50	1.00E+00	1.00E+00	1.00E+00	1.00E+00	1.00E+00	1.00E+00	2.60E-02	large subunit ribosomal protein L39e
KO:K02926	10.27	10.97	16.71	6.43	8.87	3.46	3.00	1.91E-01	1.42E-01	8.29E-03	8.28E-01	3.93E-01	9.74E-01	9.82E-01	large subunit ribosomal protein L4
KO:K02927	0.00	0.00	0.00	0.00	0.00	0.00	2.50	1.00E+00	1.00E+00	1.00E+00	1.00E+00	1.00E+00	1.00E+00	2.67E-02	large subunit ribosomal protein L4e
KO:K02929	0.00	0.00	0.00	0.00	0.00	0.00	6.67	1.00E+00	1.00E+00	1.00E+00	1.00E+00	1.00E+00	1.00E+00	6.57E-03	large subunit ribosomal protein L44e
KO:K02930	0.00	0.00	0.00	0.00	0.00	0.00	3.33	1.00E+00	1.00E+00	1.00E+00	1.00E+00	1.00E+00	1.00E+00	1.56E-02	large subunit ribosomal protein L4e
KO:K02931	9.19	10.68	12.55	7.35	8.26	2.03	3.42	2.47E-01	1.14E-01	4.59E-02	5.97E-01	3.95E-01	9.90E-01	9.70E-01	large subunit ribosomal protein L5
KO:K02933	10.62	10.87	12.66	7.86	5.84	2.19	5.42	1.39E-01	1.20E-01	4.63E-02	5.94E-01	8.95E-01	9.93E-01	9.22E-01	large subunit ribosomal protein L6
KO:K02935	9.22	9.04	8.96	9.98	11.87	1.69	1.04	2.52E-01	2.70E-01	2.78E-01	1.81E-01	8.24E-02	9.82E-01	9.89E-01	large subunit ribosomal protein L7/L12
KO:K02936	0.00	0.00	0.00	0.00	0.00	0.00	9.17	1.00E+00	1.00E+00	1.00E+00	1.00E+00	1.00E+00	1.00E+00	7.00E-03	large subunit ribosomal protein L7Ae
KO:K02939	9.67	10.19	11.99	5.06	5.33	5.54	2.88	1.62E-01	1.31E-01	6.64E-02	8.43E-01	8.15E-01	7.92E-01	9.59E-01	large subunit ribosomal protein L9
KO:K02945	11.66	12.62	18.04	10.36	12.78	6.95	8.02	5.25E-01	3.35E-01	4.14E-03	7.78E-01	3.05E-01	9.69E-01	9.39E-01	small subunit ribosomal protein S1
KO:K02946	9.25	3.72	10.65	6.11	3.80	7.01	5.37	1.55E-01	8.56E-01	9.60E-02	5.19E-01	8.48E-01	3.73E-01	6.46E-01	small subunit ribosomal protein S1
KO:K02948	7.22	11.00	6.79	6.38	6.79	3.69	6.56	5.06E-01	9.46E-02	5.95E-01	6.69E-01	5.95E-01	9.33E-01	6.36E-01	small subunit ribosomal protein S11
KO:K02950	8.41	2.81	10.66	6.13	4.88	14.30	6.96	2.62E-01	9.21E-01	1.19E-01	5.70E-01	7.50E-01	3.64E-02	4.39E-01	small subunit ribosomal protein S12
KO:K02952	8.53	9.68	16.39	5.55	11.09	1.49	4.64	3.37E-01	1.92E-01	1.34E-02	8.38E-01	9.93E-02	9.92E-01	9.07E-01	small subunit ribosomal protein S13
KO:K02954	8.22	4.89	5.88	4.61	8.16	4.35	1.91	1.22E-01	5.95E-01	3.90E-01	6.56E-01	1.25E-01	7.02E-01	9.47E-01	small subunit ribosomal protein S14
KO:K02956	8.55	7.10	10.33	4.52	5.27	0.00	2.98	1.27E-01	2.30E-01	7.03E-02	6.32E-01	4.89E-01	1.00E+00	8.57E-01	small subunit ribosomal protein S15
KO:K02959	8.50	4.69	4.79	6.11	8.52	2.06	4.74	1.53E-01	6.91E-01	6.74E-01	4.36E-01	1.52E-01	9.38E-01	6.83E-01	small subunit ribosomal protein S16
KO:K02961	5.77	5.23	7.49	3.01	4.01	5.23	3.43	2.96E-01	3.80E-01	1.29E-01	8.09E-01	6.32E-01	3.80E-01	7.44E-01	small subunit ribosomal protein S17
KO:K02962	0.00	0.00	0.00	0.00	0.00	0.00	3.33	1.00E+00	1.00E+00	1.00E+00	1.00E+00	1.00E+00	1.00E+00	6.86E-03	small subunit ribosomal protein S17e
KO:K02963	6.14	3.39	4.29	4.51	6.45	9.13	3.71	3.09E-01	7.57E-01	6.06E-01	5.69E-01	2.71E-01	1.00E-01	7.05E-01	small subunit ribosomal protein S18
KO:K02965	8.86	5.38	10.89	8.58	4.24	0.27	3.97	1.38E-01	6.44E-01	6.03E-02	1.56E-01	8.25E-01	9.96E-01	8.51E-01	small subunit ribosomal protein S19
KO:K02966	0.00	0.00	0.00	0.00	0.00	0.00	5.83	1.00E+00	1.00E+00	1.00E+00	1.00E+00	1.00E+00	1.00E+00	1.36E-02	small subunit ribosomal protein S19e
KO:K02967	12.10	12.73	12.33	7.88	7.93	2.61	8.10	1.40E-01	1.02E-01	1.23E-01	7.91E-01	7.86E-01	9.89E-01	7.63E-01	small subunit ribosomal protein S2
KO:K02968	4.52	7.52	3.66	3.38	14.30	2.17	0.67	5.01E-01	1.26E-01	6.80E-01	7.30E-01	1.31E-02	8.85E-01	9.75E-01	small subunit ribosomal protein S2
KO:K02970	7.95	5.26	13.38	4.08	0.38	0.00	2.93	1.06E-01	3.20E-01	2.16E-02	5.17E-01	9.70E-01	1.00E+00	7.37E-01	small subunit ribosomal protein S21
KO:K02975	0.00	0.00	0.00	0.00	0.00	0.00	2.50	1.00E+00	1.00E+00	1.00E+00	1.00E+00	1.00E+00	1.00E+00	2.46E-02	small subunit ribosomal protein S25e
KO:K02976	0.00	0.00	0.00	0.00	0.00	0.00	3.33	1.00E+00	1.00E+00	1.00E+00	1.00E+00	1.00E+00	1.00E+00	1.44E-02	small subunit ribosomal protein S26e
KO:K02977	0.00	0.00	0.00	0.00	0.00	0.00	2.92	1.00E+00	1.00E+00	1.00E+00	1.00E+00	1.00E+00	1.00E+00	1.79E-02	small subunit ribosomal protein S27Ae
KO:K02978	0.00	0.00	0.00	0.00	0.00	0.00	2.92	1.00E+00	1.00E+00	1.00E+00	1.00E+00	1.00E+00	1.00E+00	1.31E-02	small subunit ribosomal protein S27e
KO:K02979	0.00	0.00	0.00	0.00	0.00	0.00	4.58	1.00E+00	1.00E+00	1.00E+00	1.00E+00	1.00E+00	1.00E+00	1.64E-02	small subunit ribosomal protein S28e
KO:K02982	11.72	10.37	11.19	7.86	8.07	3.12	5.75	1.09E-01	2.17E-01	1.44E-01	6.67E-01	6.25E-01	9.84E-01	9.09E-01	small subunit ribosomal protein S3
KO:K02983	0.00	0.00	0.00	0.00	0.00	0.00	3.33	1.00E+00	1.00E+00	1.00E+00	1.00E+00	1.00E+00	1.00E+00	1.46E-02	small subunit ribosomal protein S3e
KO:K02984	0.00	0.00	0.00	0.00	0.00	0.00	2.92	1.00E+00	1.00E+00	1.00E+00	1.00E+00	1.00E+00	1.00E+00	9.86E-03	small subunit ribosomal protein S3Ae
KO:K02986	9.34	13.03	8.26	8.79	7.72	4.79	5.36	3.84E-01	5.97E-02	6.12E-01	4.99E-01	7.16E-01	9.53E-01	9.33E-01	small subunit ribosomal protein S4
KO:K02987	0.00	0.00	0.00	0.00	0.00	0.00	3.75	1.00E+00	1.00E+00	1.00E+00	1.00E+00	1.00E+00	1.00E+00	1.31E-02	small subunit ribosomal protein S4e
KO:K02988	9.51	10.51	12.33	7.45	4.53	2.08	5.59	2.10E-01	1.28E-01	5.71E-02	5.56E-01	9.23E-01	9.87E-01	8.46E-01	small subunit ribosomal protein S5
KO:K02990	8.30	11.04	5.35	6.41	6.07	2.62	1.79	2.30E-01	7.89E-02	6.82E-01	4.95E-01	5.58E-01	9.37E-01	9.63E-01	small subunit ribosomal protein S6
KO:K02991	0.00	0.00	0.00	0.00	0.00	0.00	6.25	1.00E+00	1.00E+00	1.00E+00	1.00E+00	1.00E+00	1.00E+00	5.71E-03	small subunit ribosomal protein S6e
KO:K02992	9.73	11.60	14.87	8.61	7.49	1.99	6.93	3.63E-01	1.58E-01	4.29E-02	5.62E-01	7.48E-01	9.91E-01	8.14E-01	small subunit ribosomal protein S7
KO:K02994	9.12	9.21	8.95	6.81	5.97	1.85	2.66	1.53E-01	1.46E-01	1.66E-01	4.72E-01	6.48E-01	9.83E-01	9.61E-01	small subunit ribosomal protein S8
KO:K02995	0.00	0.00	0.00	0.00	0.00	0.00	3.33	1.00E+00	1.00E+00	1.00E+00	1.00E+00	1.00E+00	1.00E+00	1.67E-02	small subunit ribosomal protein S8e
KO:K02996	10.83	10.78	11.43	5.62	5.03	2.03	2.45	1.07E-01	1.09E-01	8.47E-02	7.56E-01	8.30E-01	9.76E-01	9.69E-01	small subunit ribosomal protein S9
KO:K03014	0.00	0.00	0.00	0.00	0.00	0.00	4.17	1.00E+00	1.00E+00	1.00E+00	1.00E+00	1.00E+00	1.00E+00	2.47E-02	DNA-directed RNA Polymerase II subunit F
KO:K03021	0.00	0.00	0.00	0.00	0.00	0.00	4.58	1.00E+00	1.00E+00	1.00E+00	1.00E+00	1.00E+00	1.00E+00	2.59E-02	DNA-directed RNA polymerase III subunit C2 [EC:2.7.7.6]
KO:K03040	10.01	12.60	19.08	6.77	10.73	3.93	4.85	4.11E-01	1.17E-01	2.71E-03	8.98E-01	2.91E-01	9.79E-01	9.66E-01	DNA-directed RNA polymerase subunit alpha [EC:2.7.7.6]
KO:K03042	0.00	0.00	0.00	0.00	0.00	0.00	7.50	1.00E+00	1.00E+00	1.00E+00	1.00E+00	1.00E+00	1.00E+00	6.43E-03	DNA-directed RNA polymerase subunit A" [EC:2.7.7.6]
KO:K03043	12.51	14.06	14.42	12.35	9.88	15.21	7.03	4.76E-01	1.42E-01	9.43E-02	5.22E-01	9.28E-01	3.16E-02	9.84E-01	DNA-directed RNA polymerase subunit beta [EC:2.7.7.6]
KO:K03046	12.17	14.38	14.48	12.31	13.88	6.52	6.97	5.06E-01	9.49E-02	8.50E-02	4.72E-01	1.57E-01	9.89E-01	9.82E-01	DNA-directed RNA polymerase subunit beta' [EC:2.7.7.6]
KO:K03047	0.00	0.00	0.00	0.00	0.00	0.00	5.42	1.00E+00	1.00E+00	1.00E+00	1.00E+00	1.00E+00	1.00E+00	1.80E-02	DNA-directed RNA polymerase subunit D [EC:2.7.7.6]
KO:K03049	0.00	0.00	0.00	0.00	0.00	0.00	6.25	1.00E+00	1.00E+00	1.00E+00	1.00E+00	1.00E+00	1.00E+00	6.71E-03	DNA-directed RNA polymerase subunit E' [EC:2.7.7.6]
KO:K03050	0.00	0.00	0.00	0.00	0.00	0.00	2.08	1.00E+00	1.00E+00	1.00E+00	1.00E+00	1.00E+00	1.00E+00	2.77E-02	DNA-directed RNA polymerase subunit E" [EC:2.7.7.6]
KO:K03051	0.00	0.00	0.00	0.00	0.00	0.00	5.00	1.00E+00	1.00E+00	1.00E+00	1.00E+00	1.00E+00	1.00E+00	1.56E-02	DNA-directed RNA polymerase subunit F [EC:2.7.7.6]
KO:K03053	0.00	0.00	0.00	0.00	0.00	0.00	1.67	1.00E+00	1.00E+00	1.00E+00	1.00E+00	1.00E+00	1.00E+00	2.29E-02	DNA-directed RNA polymerase subunit H [EC:2.7.7.6]
KO:K03056	0.00	0.00	0.00	0.00	0.00	0.00	3.75	1.00E+00	1.00E+00	1.00E+00	1.00E+00	1.00E+00	1.00E+00	1.49E-02	DNA-directed RNA polymerase subunit L [EC:2.7.7.6]
KO:K03057	0.00	0.00	0.00	0.00	0.00	0.00	2.92	1.00E+00	1.00E+00	1.00E+00	1.00E+00	1.00E+00	1.00E+00	2.73E-02	transcription elongation factor
KO:K03058	0.00	0.00	0.00	0.00	0.00	0.00	2.50	1.00E+00	1.00E+00	1.00E+00	1.00E+00	1.00E+00	1.00E+00	1.60E-02	DNA-directed RNA polymerase subunit N [EC:2.7.7.6]
KO:K03059	0.00	0.00	0.00	0.00	0.00	0.00	2.50	1.00E+00	1.00E+00	1.00E+00	1.00E+00	1.00E+00	1.00E+00	2.69E-02	DNA-directed RNA polymerase subunit P [EC:2.7.7.6]
KO:K03060	5.89	12.07	0.79	5.51	4.07	0.62	0.57	2.59E-01	3.29E-02	9.59E-01	3.06E-01	5.64E-01	9.66E-01	9.67E-01	DNA-directed RNA polymerase subunit omega [EC:2.7.7.6]
KO:K03070	10.59	11.62	12.28	9.13	7.50	8.82	2.84	2.21E-01	1.16E-01	7.89E-02	5.18E-01	8.31E-01	5.95E-01	9.95E-01	preprotein translocase subunit SecA
KO:K03071	6.55	6.71	0.01	4.78	0.00	0.00	1.07	7.00E-02	6.59E-02	9.87E-01	1.44E-01	1.00E+00	1.00E+00	8.24E-01	preprotein translocase subunit SecB
KO:K03072	10.25	9.97	10.70	5.56	4.95	1.80	3.11	9.87E-02	1.09E-01	9.77E-01	5.08E-01	6.15E-01	9.40E-01	8.56E-01	preprotein translocase subunit SecD
KO:K03073	7.05	2.84	14.08	6.07	3.45	2.09	5.27	2.47E-01	8.65E-01	2.36E-02	3.70E-01	8.07E-01	9.17E-01	5.06E-01	preprotein translocase subunit SecE
KO:K03074	8.86	8.37	0.68	4.14	5.20	2.40	1.96	9.99E-02	1.19E-01	9.70E-01	6.17E-01	4.22E-01	8.67E-01	9.05E-01	preprotein translocase subunit SecF
KO:K03075	7.26	6.94	2.00	3.42	1.03	10.90	1.46	1.39E-01	1.63E-01	8.79E-01	6.88E-01	9.47E-01	4.29E-02	9.21E-01	preprotein translocase subunit SecG
KO:K03076	11.28	13.59	13.59	10.46	7.33	7.22	5.49	3.06E-01	8.56E-02	8.56E-02	4.75E-01	9.17E-01	9.23E-01	9.73E-01	preprotein translocase subunit SecY
KO:K03077	0.00	0.00	0.00	0.31	0.00	0.00	0.00	1.00E+00	1.00E+00	1.00E+00	1.43E-01	1.00E+00	1.00E+00	1.00E+00	L-ribulose-5-phosphate 4-epimerase [EC:5.1.3.4]
KO:K03082	0.05	0.00	11.18	0.20	2.30	0.00	1.87	5.85E-01	1.00E+00	1.44E-02	5.05E-01	1.34E-01	1.00E+00	1.68E-01	hexulose-6-phosphate isomerase [EC:5.-.-.-]
KO:K03086	7.05	6.72	18.74	6.20	15.03	11.26	4.83	8.23E-01	8.50E-01	8.71E-03	8.89E-01	4.03E-02	2.06E-01	9.52E-01	RNA polymerase primary sigma factor
KO:K03087	2.56	0.14	0.18	0.26	25.72	0.00	0.72	2.10E-01	8.64E-01	8.53E-01	8.33E-01	8.57E-04	1.00E+00	6.78E-01	RNA polymerase nonessential primary-like sigma factor
KO:K03088	10.19	4.80	22.63	6.63	2.83	2.92	3.23	1.70E-01	7.91E-01	6.43E-03	5.46E-01	9.27E-01	9.24E-01	9.06E-01	RNA polymerase sigma-7 factor, ECF subfamily
KO:K03089	11.01	26.14	1.20	9.93	0.00	2.40	5.10	1.64E-01	5.71E-04	9.87E-01	2.48E-01	1.00E+00	9.59E-01	8.47E-01	RNA polymerase sigma-32 factor
KO:K03090	0.00	0.00	0.00	0.00	1.25	0.00	0.00	1.00E+00	1.00E+00	1.00E+00	1.00E+00	1.89E-02	1.00E+00	1.00E+00	RNA polymerase sigma-B factor
KO:K03092	6.42	1.51	12.38	4.09	0.73	5.79	1.38	1.25E-01	8.94E-01	1.47E-02	4.07E-01	9.57E-01	1.68E-01	9.05E-01	RNA polymerase sigma-54 factor
KO:K03093	0.00	0.00	35.53	0.05	0.97	0.00	0.69	6.38E-01	1.00E+00	8.57E-04	5.87E-01	2.88E-01	1.00E+00	3.41E-01	RNA polymerase sigma factor
KO:K03098	2.97	0.02	10.68	0.97	0.00	0.00	0.00	1.25E-01	7.55E-01	2.09E-02	3.95E-01	1.00E+00	1.00E+00	7.93E-01	outer membrane lipoprotein Blc
KO:K03100	7.34	12.63	5.80	5.13	5.84	1.34	0.85	2.37E-01	2.76E-02	5.08E-01	6.59E-01	5.01E-01	9.82E-01	9.90E-01	signal peptidase I [EC:3.4.21.89]
KO:K03101	6.79	3.99	5.56	2.54	3.08	0.34	0.23	7.23E-02	2.76E-01	1.20E-01	6.35E-01	4.85E-01	9.72E-01	9.81E-01	signal peptidase II [EC:3.4.23.36]
KO:K03105	0.00	0.00	0.00	0.00	0.00	0.00	1.25	1.00E+00	1.00E+00	1.00E+00	1.00E+00	1.00E+00	1.00E+00	6.86E-03	signal recognition particle subunit SRP19
KO:K03106	7.64	9.67	7.24	5.95	7.62	4.48	4.88	3.70E-01	1.26E-01	4.49E-01	7.21E-01	3.74E-01	8.93E-01	8.61E-01	signal recognition particle subunit SRP54
KO:K03110	4.46	7.54	7.04	3.25	8.75	0.22	2.64	4.97E-01	8.41E-02	1.07E-01	7.90E-01	5.10E-02	9.97E-01	8.76E-01	fused signal recognition particle receptor
KO:K03111	5.96	4.44	10.09	5.27	3.68	6.01	1.37	2.98E-01	6.05E-01	4.17E-02	4.27E-01	7.52E-01	2.90E-01	9.65E-01	single-strand DNA-binding protein
KO:K03112	0.03	0.00	0.00	0.00	0.00	2.93	0.00	2.89E-01	4.00E-01	1.00E+00	1.00E+00	1.00E+00	3.14E-03	1.00E+00	DamX protein
KO:K03113	1.50	0.20	5.54	0.32	1.50	0.00	0.67	2.13E-01	7.81E-01	2.33E-02	7.11E-01	2.14E-01	1.00E+00	5.06E-01	translation initiation factor SUI1
KO:K03116	3.83	6.13	7.83	2.65	4.01	3.30	1.70	5.39E-01	1.85E-01	9.93E-02	7.71E-01	5.04E-01	6.41E-01	8.85E-01	sec-independent protein translocase protein TatA
KO:K03117	5.84	11.30	0.01	1.57	0.00	0.00	2.13	9.80E-02	1.97E-02	9.67E-01	6.86E-01	1.00E+00	1.00E+00	5.53E-01	sec-independent protein translocase protein TatB
KO:K03118	5.61	9.17	3.20	3.91	4.53	5.16	1.23	3.32E-01	6.13E-02	8.21E-01	6.99E-01	5.51E-01	4.19E-01	9.66E-01	sec-independent protein translocase protein TatC
KO:K03119	9.17	0.19	0.00	12.44	0.00	0.00	0.00	5.63E-02	8.07E-01	1.00E+00	2.86E-02	1.00E+00	1.00E+00	1.00E+00	taurine dioxygenase [EC:1.14.11.17]
KO:K03120	0.00	0.00	0.00	0.00	0.00	0.00	10.00	1.00E+00	1.00E+00	1.00E+00	1.00E+00	1.00E+00	1.00E+00	8.14E-03	transcription initiation factor TFIID TATA-box-binding protein
KO:K03124	0.00	0.00	0.00	0.00	0.00	0.00	13.75	1.00E+00	1.00E+00	1.00E+00	1.00E+00	1.00E+00	1.00E+00	6.43E-03	transcription initiation factor TFIIB
KO:K03136	0.00	0.00	0.00	0.00	0.00	0.00	4.58	1.00E+00	1.00E+00	1.00E+00	1.00E+00	1.00E+00	1.00E+00	7.00E-03	transcription initiation factor TFIIE alpha subunit
KO:K03146	0.00	0.00	0.00	0.00	0.00	0.00	5.00	1.00E+00	1.00E+00	1.00E+00	1.00E+00	1.00E+00	1.00E+00	1.50E-02	thiamine biosynthetic enzyme
KO:K03147	1.88	0.19	4.12	1.79	18.11	0.00	10.24	6.38E-01	9.49E-01	2.03E-01	6.61E-01	4.29E-03	1.00E+00	2.46E-02	thiamine biosynthesis protein ThiC

KO:K03148	3.37	0.00	0.00	0.00	0.00	0.00	0.00	4.94E-02	1.00E+00	1.00E+00	1.00E+00	1.00E+00	1.00E+00	1.00E+00	adenylyltransferase ThiF [EC:2.7.7.-]
KO:K03149	0.75	0.26	3.75	3.10	9.80	1.48	2.42	8.13E-01	9.14E-01	1.58E-01	2.26E-01	1.77E-02	6.14E-01	3.56E-01	thiamine biosynthesis ThiG
KO:K03150	0.18	0.05	0.02	0.20	5.82	0.00	0.25	4.71E-01	6.11E-01	6.61E-01	4.54E-01	1.61E-02	1.00E+00	4.10E-01	thiamine biosynthesis ThiH
KO:K03151	3.34	0.04	0.00	0.10	0.00	0.00	0.81	3.63E-02	6.56E-01	1.00E+00	5.94E-01	1.00E+00	1.00E+00	2.11E-01	thiamine biosynthesis protein ThiI
KO:K03152	0.04	0.00	7.86	0.03	0.00	0.00	0.69	5.29E-01	1.00E+00	5.71E-03	5.46E-01	1.00E+00	1.00E+00	1.56E-01	4-methyl-5(b-hydroxyethyl)-thiazole monophosphate biosynthesis
KO:K03153	0.37	8.66	0.38	0.37	3.17	0.00	2.09	8.56E-01	1.69E-02	8.49E-01	8.55E-01	1.51E-01	1.00E+00	3.13E-01	glycine oxidase [EC:1.4.3.19]
KO:K03154	0.06	0.03	1.13	0.95	2.13	0.00	0.63	8.29E-01	8.65E-01	1.19E-01	1.56E-01	4.61E-02	1.00E+00	2.54E-01	thiamine biosynthesis ThiS
KO:K03163	0.00	0.00	0.00	0.00	0.00	0.00	5.00	1.00E+00	1.00E+00	1.00E+00	1.00E+00	1.00E+00	1.00E+00	7.14E-03	DNA topoisomerase I [EC:5.99.1.2]
KO:K03166	0.00	0.00	6.49	0.00	0.00	0.00	1.28	1.00E+00	1.00E+00	7.57E-03	1.00E+00	1.00E+00	1.00E+00	9.46E-02	DNA topoisomerase VI subunit A [EC:5.99.1.3]
KO:K03167	0.00	0.00	7.42	0.00	0.00	0.00	1.38	1.00E+00	1.00E+00	6.00E-03	1.00E+00	1.00E+00	1.00E+00	9.20E-02	DNA topoisomerase VI subunit B [EC:5.99.1.3]
KO:K03168	13.23	11.65	3.19	7.65	7.44	6.78	2.40	3.96E-02	8.40E-02	9.70E-01	5.72E-01	6.14E-01	7.37E-01	9.85E-01	DNA topoisomerase I [EC:5.99.1.2]
KO:K03169	0.03	0.00	18.94	0.05	2.29	0.00	0.10	6.14E-01	6.98E-01	6.29E-03	5.80E-01	1.08E-01	1.00E+00	5.44E-01	DNA topoisomerase III [EC:5.99.1.2]
KO:K03177	7.19	4.40	10.99	2.23	0.71	5.02	2.17	1.02E-01	4.20E-01	2.44E-02	8.53E-01	9.73E-01	3.03E-01	8.59E-01	tRNA pseudouridine synthase B [EC:5.4.99.12]
KO:K03179	3.53	4.69	0.63	3.60	5.88	2.85	0.97	3.80E-01	1.78E-01	9.67E-01	3.64E-01	9.19E-02	5.72E-01	9.38E-01	4-hydroxybenzoate octaprenyltransferase [EC:2.5.1.-]
KO:K03181	1.35	0.00	0.00	0.17	0.00	0.00	1.17	5.46E-02	7.49E-01	1.00E+00	4.72E-01	1.00E+00	1.00E+00	7.07E-02	chorismate--pyruvate lyase [EC:4.1.3.4]
KO:K03182	3.99	0.13	0.95	0.50	7.78	0.31	2.50	9.26E-02	9.02E-01	6.42E-01	7.95E-01	2.37E-02	8.50E-01	2.18E-01	3-octaprenyl-4-hydroxybenzoate carboxy-lyase UbiD [EC:4.1.1.-]
KO:K03183	6.09	6.47	6.14	4.24	4.36	1.63	2.06	2.08E-01	1.65E-01	2.01E-01	5.84E-01	5.54E-01	9.54E-01	9.26E-01	ubiquinone/menaquinone biosynthesis methyltransferase [EC:2.1.1.-]
KO:K03184	0.16	0.00	0.13	0.04	0.00	7.53	1.47	4.88E-01	7.12E-01	5.24E-01	6.49E-01	1.00E+00	5.14E-03	8.66E-02	2-octaprenyl-3-methyl-6-methoxy-1,4-benzoquinol hydroxylase
KO:K03185	3.39	18.86	0.03	1.88	4.01	0.02	0.06	4.18E-01	1.43E-03	9.87E-01	7.83E-01	2.98E-01	9.87E-01	9.86E-01	2-octaprenyl-6-methoxyphenol hydroxylase [EC:1.14.13.-]
KO:K03186	3.11	0.05	0.52	0.28	10.33	0.00	1.79	1.04E-01	9.13E-01	6.80E-01	7.99E-01	1.00E-02	1.00E+00	2.38E-01	3-octaprenyl-4-hydroxybenzoate carboxy-lyase UbiX [EC:4.1.1.-]
KO:K03187	0.38	2.89	0.14	0.01	3.85	0.11	0.00	5.82E-01	4.61E-02	7.74E-01	8.83E-01	2.77E-02	8.00E-01	1.00E+00	urease accessory protein
KO:K03188	0.19	2.08	0.00	0.15	7.99	0.00	0.00	7.75E-01	5.84E-02	1.00E+00	8.10E-01	1.43E-03	1.00E+00	1.00E+00	urease accessory protein
KO:K03189	0.27	2.00	0.12	0.81	2.32	0.39	0.00	7.71E-01	8.49E-02	8.59E-01	3.65E-01	6.83E-02	6.73E-01	1.00E+00	urease accessory protein
KO:K03190	0.38	3.46	0.06	0.34	1.23	0.11	0.11	6.28E-01	4.06E-02	8.93E-01	6.67E-01	2.15E-01	8.45E-01	8.45E-01	urease accessory protein
KO:K03192	0.29	1.58	0.00	1.59	5.88	0.00	0.45	6.39E-01	2.76E-01	1.00E+00	2.75E-01	5.61E-02	1.00E+00	5.79E-01	urease accessory protein
KO:K03196	0.00	0.00	0.00	0.10	0.00	0.00	0.00	1.00E+00	1.00E+00	1.00E+00	1.43E-01	1.00E+00	1.00E+00	1.00E+00	type IV secretion system protein VirB11
KO:K03197	0.00	0.00	0.00	0.41	0.00	0.00	0.00	1.00E+00	1.00E+00	1.00E+00	7.77E-02	1.00E+00	1.00E+00	1.00E+00	type IV secretion system protein VirB2
KO:K03198	0.00	0.11	0.00	0.00	0.00	0.00	0.00	1.00E+00	1.01E-01	1.00E+00	1.00E+00	1.00E+00	1.00E+00	1.00E+00	type IV secretion system protein VirB3
KO:K03199	0.06	0.00	0.00	0.05	0.00	0.00	0.00	1.03E-01	1.00E+00	1.00E+00	2.12E-01	1.00E+00	1.00E+00	1.00E+00	type IV secretion system protein VirB4
KO:K03201	0.00	0.00	0.00	0.10	0.00	0.00	0.00	1.00E+00	1.00E+00	1.00E+00	1.43E-01	1.00E+00	1.00E+00	1.00E+00	type IV secretion system protein VirB6
KO:K03205	0.00	0.00	0.54	0.01	0.00	0.00	0.00	1.00E+00	1.00E+00	1.24E-02	2.49E-01	1.00E+00	1.00E+00	1.00E+00	type IV secretion system protein VirD4
KO:K03207	1.63	0.00	0.00	0.00	0.00	0.00	0.00	6.29E-02	1.00E+00	1.00E+00	1.00E+00	1.00E+00	1.00E+00	1.00E+00	colanic acid biosynthesis protein WcaH [EC:3.6.1.-]
KO:K03208	0.04	0.01	0.07	0.00	3.31	0.00	0.00	3.63E-01	4.65E-01	3.12E-01	5.31E-01	1.24E-02	1.00E+00	1.00E+00	colanic acid biosynthesis glycosyl transferase WcaI
KO:K03210	5.55	8.47	10.17	3.78	0.27	0.56	2.75	2.78E-01	9.11E-02	4.96E-02	5.93E-01	9.80E-01	9.66E-01	7.87E-01	preprotein translocase subunit YajC
KO:K03214	0.00	0.00	0.63	0.00	0.00	0.00	0.00	1.00E+00	1.00E+00	1.53E-02	1.00E+00	1.00E+00	1.00E+00	1.00E+00	RNA methyltransferase, TrmH family [EC:2.1.1.-]
KO:K03215	2.69	2.53	0.56	1.15	0.00	8.49	1.40	2.33E-01	2.64E-01	8.50E-01	6.82E-01	1.00E+00	1.71E-02	5.90E-01	RNA methyltransferase, TrmA family [EC:2.1.1.-]
KO:K03216	1.02	0.24	1.85	1.83	10.47	2.98	0.23	6.46E-01	9.00E-01	3.48E-01	3.53E-01	7.43E-03	1.50E-01	9.03E-01	RNA methyltransferase, TrmH family, group 2 [EC:2.1.1.-]
KO:K03217	8.49	13.47	7.21	6.42	7.40	2.80	2.96	2.86E-01	2.63E-02	5.37E-01	7.05E-01	4.95E-01	9.77E-01	9.74E-01	preprotein translocase subunit YidC
KO:K03218	6.42	4.80	0.23	2.31	6.45	1.61	1.20	8.60E-02	1.86E-01	9.80E-01	7.17E-01	8.51E-02	8.48E-01	9.00E-01	RNA methyltransferase, TrmH family [EC:2.1.1.-]
KO:K03222	0.02	0.00	0.00	0.61	0.00	0.00	0.00	3.12E-01	1.00E+00	1.00E+00	6.04E-02	1.00E+00	1.00E+00	1.00E+00	type III secretion protein SctJ
KO:K03223	0.12	0.00	0.00	0.00	0.00	0.00	0.00	6.24E-02	1.00E+00	1.00E+00	1.00E+00	1.00E+00	1.00E+00	1.00E+00	type III secretion protein SctL
KO:K03224	0.16	0.00	0.00	0.03	0.00	0.00	0.00	9.00E-02	1.00E+00	1.00E+00	2.34E-01	1.00E+00	1.00E+00	1.00E+00	ATP synthase in type III secretion protein SctN [EC:3.6.3.14]
KO:K03226	0.00	0.00	0.00	0.10	0.00	0.00	0.00	1.00E+00	1.00E+00	1.00E+00	1.43E-01	1.00E+00	1.00E+00	1.00E+00	type III secretion protein SctR
KO:K03229	0.03	0.00	0.00	0.22	0.00	0.00	0.00	2.17E-01	1.00E+00	1.00E+00	1.12E-01	1.00E+00	1.00E+00	1.00E+00	type III secretion protein SctU
KO:K03230	0.03	0.00	0.00	0.32	0.00	0.00	0.00	2.67E-01	1.00E+00	1.00E+00	7.44E-02	1.00E+00	1.00E+00	1.00E+00	type III secretion protein SctV
KO:K03231	0.00	0.00	0.00	0.00	0.00	0.00	12.50	1.00E+00	1.00E+00	1.00E+00	1.00E+00	1.00E+00	1.00E+00	6.00E-03	elongation factor EF-1 alpha subunit [EC:3.6.5.3]
KO:K03232	0.00	0.00	0.00	0.00	0.00	0.00	7.50	1.00E+00	1.00E+00	1.00E+00	1.00E+00	1.00E+00	1.00E+00	6.14E-03	elongation factor EF-1 beta subunit
KO:K03234	0.00	0.00	0.00	0.00	0.00	0.00	9.58	1.00E+00	1.00E+00	1.00E+00	1.00E+00	1.00E+00	1.00E+00	6.57E-03	elongation factor EF-2 [EC:3.6.5.3]
KO:K03236	0.00	0.00	0.00	0.00	0.00	0.00	4.17	1.00E+00	1.00E+00	1.00E+00	1.00E+00	1.00E+00	1.00E+00	5.57E-03	translation initiation factor eIF-1A
KO:K03237	0.00	0.00	0.00	0.00	0.00	0.00	2.08	1.00E+00	1.00E+00	1.00E+00	1.00E+00	1.00E+00	1.00E+00	1.63E-02	translation initiation factor eIF-2 alpha subunit
KO:K03238	0.00	0.00	0.00	0.00	0.00	0.00	4.58	1.00E+00	1.00E+00	1.00E+00	1.00E+00	1.00E+00	1.00E+00	5.57E-03	translation initiation factor eIF-2 beta subunit
KO:K03242	0.00	0.00	0.00	0.00	0.00	0.00	6.25	1.00E+00	1.00E+00	1.00E+00	1.00E+00	1.00E+00	1.00E+00	5.57E-03	translation initiation factor eIF-2 gamma subunit
KO:K03243	0.00	0.00	0.00	0.00	0.00	0.00	3.33	1.00E+00	1.00E+00	1.00E+00	1.00E+00	1.00E+00	1.00E+00	5.71E-03	translation initiation factor IF-2 unclassified subunit
KO:K03263	0.00	0.00	0.00	0.00	0.00	0.00	4.58	1.00E+00	1.00E+00	1.00E+00	1.00E+00	1.00E+00	1.00E+00	3.01E-02	translation initiation factor eIF-5A
KO:K03264	0.00	0.00	0.00	0.00	0.00	0.00	2.50	1.00E+00	1.00E+00	1.00E+00	1.00E+00	1.00E+00	1.00E+00	2.49E-02	translation initiation factor eIF-6
KO:K03265	0.00	0.00	0.00	0.00	0.00	0.00	2.50	1.00E+00	1.00E+00	1.00E+00	1.00E+00	1.00E+00	1.00E+00	2.86E-02	peptide chain release factor eRF subunit 1
KO:K03268	0.23	0.00	0.00	0.00	0.00	0.00	0.00	6.71E-02	1.00E+00	1.00E+00	1.00E+00	1.00E+00	1.00E+00	1.00E+00	benzene 1,2-dioxygenase [EC:1.14.12.3]
KO:K03269	8.66	0.50	0.04	1.56	0.00	5.72	5.12	2.67E-02	8.88E-01	9.60E-01	6.41E-01	1.00E+00	7.34E-02	9.60E-02	UDP-2,3-diacylglucosamine hydrolase [EC:3.6.1.-]
KO:K03270	4.71	0.25	4.61	0.65	0.79	0.30	3.27	6.91E-02	9.00E-01	7.24E-02	7.87E-01	7.46E-01	8.86E-01	1.39E-01	3-deoxy-D-manno-octulosonate 8-phosphate phosphatase (KDO 8-P
KO:K03271	1.71	0.10	1.92	3.74	0.94	1.23	2.82	4.18E-01	8.84E-01	3.63E-01	1.37E-01	6.36E-01	5.49E-01	2.11E-01	phosphoheptose isomerase [EC:5.-.-.-]
KO:K03272	4.20	0.14	1.41	2.35	3.41	3.74	0.23	1.40E-01	8.74E-01	5.55E-01	3.32E-01	1.97E-01	1.69E-01	8.58E-01	D-beta-D-heptose 7-phosphate kinase / D-beta-D-heptose 1-phosphate
KO:K03273	2.94	0.08	1.56	1.33	0.00	0.06	4.09	1.13E-01	8.83E-01	2.85E-01	3.48E-01	1.00E+00	9.02E-01	6.67E-02	D-glycero-D-manno-heptose 1,7-bisphosphate phosphatase [EC:3.1.3.-]
KO:K03274	1.03	0.10	0.00	2.48	1.94	1.76	0.44	3.86E-01	8.08E-01	1.00E+00	1.31E-01	1.87E-01	2.14E-01	6.45E-01	ADP-L-glycero-D-manno-heptose 6-epimerase [EC:5.1.3.2]
KO:K03281	1.34	3.52	1.21	0.89	9.40	2.88	0.86	7.01E-01	1.93E-01	7.38E-01	8.20E-01	2.00E-02	2.85E-01	8.25E-01	chloride channel protein, ClC family
KO:K03282	0.17	3.70	0.01	0.22	5.43	0.00	0.14	7.86E-01	4.70E-02	9.10E-01	7.54E-01	2.63E-02	1.00E+00	8.07E-01	large conductance mechanosensitive channel
KO:K03284	2.60	2.16	0.36	0.19	3.58	0.00	0.07	1.37E-01	1.77E-01	6.98E-01	7.89E-01	8.36E-02	1.00E+00	8.57E-01	metal ion transporter, MIT family
KO:K03285	0.00	0.00	0.00	1.84	0.00	0.00	0.00	1.00E+00	1.00E+00	1.00E+00	1.43E-01	1.00E+00	1.00E+00	1.00E+00	general bacterial porin, GBP family
KO:K03286	1.52	12.99	0.25	0.35	0.00	0.00	0.04	3.84E-01	1.00E-03	9.01E-01	8.79E-01	1.00E+00	1.00E+00	9.58E-01	OmpA-OmpF porin, OOP family
KO:K03287	0.14	0.94	0.26	1.62	17.41	0.00	0.00	7.86E-01	4.70E-01	7.36E-01	2.90E-01	3.71E-03	1.00E+00	1.00E+00	outer membrane factor, OMF family
KO:K03292	1.06	3.79	4.62	0.40	5.76	0.00	0.01	5.95E-01	1.27E-01	8.93E-02	8.07E-01	5.83E-02	1.00E+00	9.07E-01	glycoside/pentoside/hexuronide:cation symporter, GPH family
KO:K03294	2.83	0.00	2.97	0.14	5.57	0.00	0.23	1.29E-01	8.19E-01	1.20E-01	6.81E-01	4.67E-02	1.00E+00	6.19E-01	basic amino acid/polyamine antiporter, APA family
KO:K03295	1.93	0.64	1.57	0.25	1.74	0.00	0.02	1.35E-01	4.29E-01	1.82E-01	6.73E-01	1.55E-01	1.00E+00	8.70E-01	cation efflux system protein, CDF family
KO:K03296	3.98	0.85	6.56	4.00	12.86	0.55	0.46	3.16E-01	8.90E-01	1.00E-01	3.12E-01	1.69E-02	9.20E-01	9.29E-01	hydrophobic/amphiphilic exporter-1 (mainly G- bacteria), HAE1 family
KO:K03297	0.04	0.17	0.02	3.77	2.20	0.00	0.03	7.07E-01	5.14E-01	7.80E-01	7.31E-02	1.22E-01	1.00E+00	7.29E-01	small multidrug resistance protein, SMR family
KO:K03298	0.26	0.03	0.00	0.00	0.00	0.00	0.00	7.04E-02	2.58E-01	1.00E+00	1.00E+00	1.00E+00	1.00E+00	1.00E+00	drug/metabolite transporter, DME family
KO:K03299	0.20	0.01	6.33	0.19	0.96	0.00	0.00	4.18E-01	5.88E-01	2.47E-02	4.21E-01	1.85E-01	1.00E+00	1.00E+00	gluconate:H+ symporter, GntP family
KO:K03303	0.35	0.01	4.47	0.02	0.00	0.00	0.00	2.30E-01	5.64E-01	1.37E-02	5.45E-01	1.00E+00	1.00E+00	1.00E+00	lactate transporter, LctP family
KO:K03304	0.00	0.30	0.00	0.11	0.00	4.75	0.00	1.00E+00	2.45E-01	1.00E+00	3.94E-01	1.00E+00	5.00E-03	1.00E+00	tellurite resistance/dicarboxylate transporter, TDT family
KO:K03305	12.72	0.70	2.77	4.20	2.44	4.33	0.00	3.23E-02	8.81E-01	5.68E-01	3.35E-01	6.33E-01	3.16E-01	1.00E+00	proton-dependent oligopeptide transporter, POT family
KO:K03306	8.58	17.59	1.17	2.45	0.00	4.56	1.21	1.22E-01	1.43E-02	9.18E-01	8.18E-01	1.00E+00	5.01E-01	9.16E-01	inorganic phosphate transporter, PiT family
KO:K03307	5.47	3.00	23.36	2.75	0.80	4.58	0.87	2.99E-01	6.66E-01	4.86E-03	7.04E-01	9.18E-01	4.20E-01	9.13E-01	solute:Na+ symporter, SSS family
KO:K03308	19.40	0.43	0.71	3.02	5.02	1.25	1.12	1.27E-02	9.14E-01	8.85E-01	5.45E-01	2.74E-01	8.26E-01	8.41E-01	neurotransmitter:Na+ symporter, NSS family
KO:K03309	2.21	0.00	2.55	2.32	0.00	0.00	0.01	1.60E-01	1.00E+00	1.36E-01	1.52E-01	1.00E+00	1.00E+00	6.67E-01	dicarboxylate/amino acid:cation (Na+ or H+) symporter, DAACS family
KO:K03310	14.80	3.04	1.84	5.27	5.55	0.35	0.09	2.29E-02	6.43E-01	8.19E-01	3.10E-01	2.79E-01	9.49E-01	9.74E-01	alanine or glycine:cation symporter, AGCS family
KO:K03311	0.94	0.16	0.00	0.09	0.00	0.00	0.00	8.11E-02	3.01E-01	1.00E+00	3.58E-01	1.00E+00	1.00E+00	1.00E+00	branched-chain amino acid:cation transporter, LIVCS family
KO:K03312	0.32	0.00	0.13	0.00	4.58	0.00	0.13	2.23E-01	6.58E-01	3.22E-01	6.31E-01	2.94E-02	1.00E+00	3.22E-01	glutamate:Na+ symporter, ESS family
KO:K03313	1.02	2.29	5.75	3.40	0.00	0.00	0.14	5.46E-01	2.87E-01	8.54E-02	1.76E-01	1.00E+00	1.00E+00	7.97E-01	Na+:H+ antiporter, NhaA family
KO:K03314	16.20	0.08	3.96	0.87	0.00	0.00	0.00	7.43E-03	7.90E-01	1.73E-01	6.29E-01	1.00E+00	1.00E+00	1.00E+00	Na+:H+ antiporter, NhaB family
KO:K03315	10.34	0.90	1.05	1.18	0.00	2.65	0.00	2.11E-02	6.80E-01	6.40E-01	6.02E-01	1.00E+00	2.86E-01	1.00E+00	Na+:H+ antiporter, NhaC family
KO:K03316	0.47	1.34	0.77	3.66	2.42	0.00	0.04	7.01E-01	3.70E-01	5.72E-01	8.56E-02	1.62E-01	1.00E+00	8.99E-01	monovalent cation:H+ antiporter, CPA1 family
KO:K03317	6.02	0.94	3.89	3.62	0.00	3.89	0.74	1.03E-01	7.97E-01	2.40E-01	2.71E-01	1.00E+00	2.40E-01	8.31E-01	concentrative nucleoside transporter, CNT family
KO:K03319	0.12	0.00	0.00	0.00	0.00	0.00	0.00	6.10E-02	1.00E+00	1.00E+00	1.00E+00	1.00E+00	1.00E+00	1.00E+00	divalent anion:Na+ symporter, DASS family
KO:K03320	6.28	4.59	6.89	2.41	1.92	4.13	11.34	3.00E-01	5.23E-01	2.42E-01	8.26E-01	8.70E-01	6.06E-01	7.34E-02	ammonium transporter, Amt family
KO:K03321	2.01	1.22	0.00	0.77	26.39	0.00	0.08	3.83E-01	5.63E-01	9.05E-01	6.82E-01	2.00E-03	1.00E+00	8.44E-01	sulfate permease, SulP family
KO:K03322	1.34	0.57	0.00	0.00	0.00	0.00	0.00	1.05E-01	1.84E-01	1.00E+00	1.00E+00	1.00E+00	1.00E+00	1.00E+00	manganese transport protein
KO:K03324	0.01	2.74	0.00	1.31	0.00	0.00	0.00	6.86E-01	7.31E-02	1.00E+00	1.50E-01	1.00E+00	1.00E+00	1.00E+00	phosphate:Na+ symporter, PNaS family

KO:K03325	2.43	0.65	0.05	0.72	0.29	2.99	0.01	7.34E-02	5.34E-01	9.27E-01	4.88E-01	7.80E-01	4.97E-02	9.51E-01	arsenite transporter, ACR3 family
KO:K03326	0.00	0.00	0.00	0.00	0.00	8.33	0.00	1.00E+00	1.00E+00	1.00E+00	1.00E+00	1.00E+00	3.00E-03	1.00E+00	C4-dicarboxylate transporter, DcuC family
KO:K03327	7.69	26.40	0.99	2.80	0.02	0.27	0.34	1.20E-01	1.00E-03	9.50E-01	7.95E-01	9.94E-01	9.85E-01	9.81E-01	multidrug resistance protein, MATE family
KO:K03328	1.32	0.55	0.45	0.09	0.00	0.79	0.00	1.33E-01	2.90E-01	3.29E-01	6.06E-01	1.00E+00	2.21E-01	1.00E+00	polysaccharide transporter, PST family
KO:K03330	0.00	0.00	0.00	0.00	0.00	0.00	2.08	1.00E+00	1.00E+00	1.00E+00	1.00E+00	1.00E+00	1.00E+00	2.64E-02	glutamyl-tRNA(Gln) amidotransferase subunit E [EC:6.3.5.7]
KO:K03331	0.00	0.22	0.00	0.00	0.00	0.00	0.00	1.00E+00	1.03E-01	1.00E+00	1.00E+00	1.00E+00	1.00E+00	1.00E+00	L-xylulose reductase [EC:1.1.1.1]
KO:K03333	0.00	0.00	0.00	0.04	0.97	1.72	0.00	4.54E-01	1.00E+00	1.00E+00	3.35E-01	4.93E-02	2.30E-02	1.00E+00	cholesterol oxidase [EC:1.1.3.6]
KO:K03335	1.47	9.36	0.01	2.32	0.00	0.00	0.07	4.47E-01	3.01E-02	8.69E-01	2.76E-01	1.00E+00	1.00E+00	8.43E-01	inosose dehydratase [EC:4.2.1.44]
KO:K03336	0.70	22.05	0.00	1.19	0.00	0.00	0.00	7.19E-01	3.00E-03	1.00E+00	5.89E-01	1.00E+00	1.00E+00	1.00E+00	3D-(3,5/4)-trihydroxycyclohexane-1,2-dione hydrolase [EC:3.7.1.-]
KO:K03337	0.48	15.12	0.00	1.18	0.00	0.00	0.00	6.89E-01	8.29E-03	1.00E+00	4.73E-01	1.00E+00	1.00E+00	1.00E+00	5-deoxy-glucuronate isomerase [EC:5.3.1.-]
KO:K03338	0.75	15.64	0.00	0.47	0.00	0.00	0.00	5.84E-01	6.14E-03	1.00E+00	6.76E-01	1.00E+00	1.00E+00	1.00E+00	5-dehydro-2-deoxygluconokinase [EC:2.7.1.92]
KO:K03342	0.00	0.00	0.00	0.61	0.00	0.00	0.00	1.00E+00	1.00E+00	1.00E+00	6.03E-02	1.00E+00	1.00E+00	1.00E+00	para-aminobenzoate synthetase / 4-amino-4-deoxychorismate lyase
KO:K03366	0.54	2.40	0.00	1.34	0.00	0.00	0.00	3.81E-01	9.57E-02	1.00E+00	1.85E-01	1.00E+00	1.00E+00	1.00E+00	(R,R)-butanediol dehydrogenase / diacetyl reductase [EC:1.1.1.4
KO:K03367	0.00	0.00	0.00	0.20	0.00	0.00	0.00	1.00E+00	1.00E+00	1.00E+00	1.43E-01	1.00E+00	1.00E+00	1.00E+00	D-alanine-poly(phosphoribitol) ligase [EC:6.1.1.13]
KO:K03379	4.05	0.31	0.33	5.41	0.00	0.00	0.00	1.34E-01	6.15E-01	6.13E-01	9.23E-02	1.00E+00	1.00E+00	1.00E+00	cyclohexanone monooxygenase [EC:1.14.13.22]
KO:K03380	1.43	3.95	0.00	1.20	0.00	0.00	0.00	2.43E-01	6.13E-02	1.00E+00	2.90E-01	1.00E+00	1.00E+00	1.00E+00	phenol 2-monooxygenase [EC:1.14.13.7]
KO:K03381	0.08	0.06	0.00	4.06	0.00	0.00	0.00	4.84E-01	5.22E-01	1.00E+00	3.41E-02	1.00E+00	1.00E+00	1.00E+00	catechol 1,2-dioxygenase [EC:1.13.11.1]
KO:K03382	1.44	17.28	0.00	0.47	0.00	0.00	0.00	4.53E-01	2.71E-03	1.00E+00	8.02E-01	1.00E+00	1.00E+00	1.00E+00	hydroxyatrazine ethylaminohydrolase [EC:3.5.99.3]
KO:K03384	1.90	0.55	0.00	0.84	0.00	0.00	0.00	1.07E-01	3.14E-01	1.00E+00	2.36E-01	1.00E+00	1.00E+00	1.00E+00	
KO:K03385	0.00	0.00	2.14	0.00	0.00	0.00	0.06	1.00E+00	1.00E+00	3.57E-03	1.00E+00	1.00E+00	1.00E+00	2.03E-01	formate-dependent nitrite reductase, periplasmic cytochrome c552
KO:K03386	3.72	0.45	1.39	3.21	15.94	0.30	3.84	2.75E-01	9.33E-01	8.11E-01	3.70E-01	4.14E-03	9.48E-01	2.55E-01	peroxiredoxin (alkyl hydroperoxide reductase subunit C)
KO:K03387	0.64	0.01	4.26	0.73	0.00	0.00	0.01	2.69E-01	6.99E-01	3.81E-02	2.45E-01	1.00E+00	1.00E+00	7.05E-01	alkyl hydroperoxide reductase subunit F [EC:1.6.4.-]
KO:K03388	0.00	0.10	0.00	0.01	0.00	0.00	3.83	1.00E+00	2.58E-01	1.00E+00	3.27E-01	1.00E+00	1.00E+00	2.53E-02	heterodisulfide reductase subunit A [EC:1.8.98.1]
KO:K03389	0.19	0.85	0.00	0.00	0.00	0.00	0.57	2.43E-01	1.45E-01	1.00E+00	1.00E+00	1.00E+00	1.00E+00	1.64E-01	heterodisulfide reductase subunit B [EC:1.8.98.1]
KO:K03390	0.09	0.46	0.00	0.00	0.00	0.00	0.29	2.35E-01	1.46E-01	1.00E+00	1.00E+00	1.00E+00	1.00E+00	1.73E-01	heterodisulfide reductase subunit C [EC:1.8.98.1]
KO:K03392	0.28	0.76	0.00	4.07	0.00	1.19	0.00	4.89E-01	3.56E-01	1.00E+00	9.29E-02	1.00E+00	2.89E-01	1.00E+00	aminocarboxymuconate-semialdehyde decarboxylase [EC:4.1.1.45]
KO:K03394	0.55	1.95	0.00	0.14	3.00	0.00	0.20	3.88E-01	1.42E-01	1.00E+00	6.36E-01	7.61E-02	1.00E+00	5.80E-01	precorrin-2 C2-methyltransferase / cobalt-factor-2
KO:K03399	0.00	0.02	0.00	0.00	0.00	0.00	2.71	1.00E+00	2.61E-01	1.00E+00	1.00E+00	1.00E+00	1.00E+00	1.83E-02	precorrin-6Y C5,15-methyltransferase [EC:2.1.1.132]
KO:K03403	0.90	4.55	0.00	1.46	17.20	0.00	0.00	6.71E-01	1.32E-01	1.00E+00	5.12E-01	7.43E-03	1.00E+00	1.00E+00	magnesium chelatase subunit H [EC:6.6.1.1]
KO:K03404	0.02	0.20	0.00	0.10	17.95	0.00	0.00	7.25E-01	5.26E-01	1.00E+00	6.39E-01	2.86E-04	1.00E+00	1.00E+00	magnesium chelatase subunit D [EC:6.6.1.1]
KO:K03405	1.76	2.66	0.32	1.46	7.56	2.84	0.14	5.01E-01	2.73E-01	8.89E-01	5.94E-01	2.71E-02	2.44E-01	9.24E-01	magnesium chelatase subunit I [EC:6.6.1.1]
KO:K03406	2.44	0.86	9.67	1.76	0.10	0.03	1.12	2.53E-01	6.76E-01	1.93E-02	3.91E-01	9.21E-01	9.50E-01	5.79E-01	methyl-accepting chemotaxis protein
KO:K03407	2.43	0.23	8.86	1.49	0.01	0.04	1.28	1.92E-01	8.40E-01	2.01E-02	3.78E-01	9.50E-01	9.27E-01	4.38E-01	two-component system, chemotaxis family, sensor kinase CheA
KO:K03408	2.72	0.11	0.94	0.35	0.06	0.10	0.79	5.60E-02	7.90E-01	2.72E-01	6.00E-01	8.43E-01	7.99E-01	3.27E-01	purine-binding chemotaxis protein CheW
KO:K03409	0.00	0.00	7.28	0.00	0.00	0.00	0.00	4.55E-01	1.00E+00	4.00E-03	4.10E-01	1.00E+00	1.00E+00	1.00E+00	chemotaxis protein CheX
KO:K03410	0.00	0.00	0.80	0.26	0.00	0.00	0.00	1.00E+00	1.00E+00	3.69E-02	1.37E-01	1.00E+00	1.00E+00	1.00E+00	chemotaxis protein CheC
KO:K03411	0.46	0.04	0.79	0.01	0.00	0.00	0.79	1.69E-01	5.75E-01	1.04E-01	6.59E-01	1.00E+00	1.00E+00	1.04E-01	chemotaxis protein CheD [EC:3.5.1.44]
KO:K03412	2.25	0.98	4.41	0.68	0.18	0.00	1.09	1.60E-01	4.93E-01	4.59E-02	6.43E-01	8.82E-01	1.00E+00	4.51E-01	two-component system, chemotaxis family, response regulator CheB
KO:K03413	0.80	0.11	2.90	3.11	0.01	0.05	2.61	5.29E-01	8.70E-01	1.07E-01	9.61E-02	9.48E-01	9.08E-01	1.27E-01	two-component system, chemotaxis family, response regulator CheY
KO:K03414	0.91	0.21	0.00	0.67	0.00	0.28	0.16	1.35E-01	5.26E-01	1.00E+00	2.04E-01	1.00E+00	4.48E-01	5.92E-01	chemotaxis protein CheZ
KO:K03415	1.41	0.16	1.01	0.33	0.00	0.51	0.34	1.40E-01	6.45E-01	2.13E-01	5.13E-01	1.00E+00	3.89E-01	5.11E-01	two-component system, chemotaxis family, response regulator CheV
KO:K03417	0.92	0.76	1.75	5.68	0.00	4.18	1.57	6.89E-01	7.32E-01	4.64E-01	8.47E-02	9.09E-01	1.51E-01	5.08E-01	methylisocitrate lyase [EC:4.1.3.3]
KO:K03418	0.56	2.03	0.00	0.90	0.00	0.00	0.00	3.05E-01	1.42E-01	1.00E+00	2.47E-01	1.00E+00	1.00E+00	1.00E+00	N,N-dimethylformamidase [EC:3.5.1.56]
KO:K03423	2.23	0.11	0.00	0.02	0.00	2.13	0.00	5.73E-02	5.39E-01	1.00E+00	6.24E-01	1.00E+00	5.97E-02	1.00E+00	
KO:K03424	5.34	2.37	6.61	2.12	8.33	0.27	1.89	1.14E-01	7.54E-01	5.80E-02	8.14E-01	2.57E-02	9.94E-01	8.56E-01	TatD DNase family protein [EC:3.1.21.-]
KO:K03426	10.95	4.90	0.26	1.24	0.00	0.00	0.03	1.30E-02	9.30E-02	9.08E-01	6.60E-01	1.00E+00	1.00E+00	9.66E-01	NAD+ diphosphatase [EC:3.6.1.22]
KO:K03427	1.18	0.19	0.63	1.31	0.40	6.47	0.73	3.63E-01	8.52E-01	6.24E-01	3.20E-01	7.48E-01	1.60E-02	5.76E-01	type I restriction enzyme M protein [EC:2.1.1.72]
KO:K03428	1.13	3.51	0.00	0.62	6.29	0.00	0.00	4.17E-01	1.01E-01	1.00E+00	6.04E-01	3.89E-02	1.00E+00	1.00E+00	magnesium-protoporphyrin O-methyltransferase [EC:2.1.1.11]
KO:K03429	0.00	0.00	0.00	0.01	37.41	0.00	0.00	6.08E-01	1.00E+00	1.00E+00	5.83E-01	0.00E+00	1.00E+00	1.00E+00	1,2-diacylglycerol 3-glucosyltransferase [EC:2.4.1.157]
KO:K03430	0.32	1.97	2.31	0.38	0.00	0.00	0.00	4.05E-01	1.60E-01	1.41E-01	3.87E-01	1.00E+00	1.00E+00	1.00E+00	2-aminoethylphosphonate-pyruvate transaminase [EC:2.6.1.37]
KO:K03431	7.36	8.58	6.12	4.52	3.48	2.64	7.23	2.65E-01	1.53E-01	4.62E-01	7.62E-01	8.80E-01	9.31E-01	2.79E-01	phosphoglucosamine mutase [EC:5.4.2.1]
KO:K03432	0.00	0.00	0.00	0.00	0.00	0.00	10.83	1.00E+00	1.00E+00	1.00E+00	1.00E+00	1.00E+00	1.00E+00	4.43E-03	proteasome alpha subunit [EC:3.4.25.1]
KO:K03433	0.00	0.00	0.00	0.00	0.41	0.00	12.29	1.00E+00	1.00E+00	1.00E+00	1.00E+00	2.31E-01	1.00E+00	9.29E-03	proteasome beta subunit [EC:3.4.25.1]
KO:K03435	0.00	0.11	0.00	0.00	0.00	0.00	0.00	1.00E+00	1.02E-01	1.00E+00	1.00E+00	1.00E+00	1.00E+00	1.00E+00	LacI family transcriptional regulator, fructose operon
KO:K03436	0.00	0.00	20.00	0.00	0.00	0.00	0.00	1.00E+00	1.00E+00	1.86E-03	1.00E+00	1.00E+00	1.00E+00	1.00E+00	DeoR family transcriptional regulator, fructose operon
KO:K03437	1.07	0.48	2.58	0.71	5.11	0.21	0.01	3.66E-01	6.37E-01	1.15E-01	5.18E-01	3.76E-02	7.96E-01	9.11E-01	RNA methyltransferase, TrmH family
KO:K03438	7.32	5.39	8.68	3.97	3.53	1.87	2.03	1.01E-01	3.24E-01	5.09E-02	6.84E-01	7.82E-01	9.51E-01	9.39E-01	S-adenosyl-methyltransferase [EC:2.1.1.-]
KO:K03439	5.51	7.96	3.79	1.07	2.31	0.00	0.37	9.61E-02	3.36E-02	2.46E-01	8.85E-01	5.93E-01	1.00E+00	9.59E-01	tRNA (guanine-N7-)-methyltransferase [EC:2.1.1.33]
KO:K03442	6.39	1.94	7.06	1.49	0.02	0.45	0.35	5.53E-02	4.97E-01	4.44E-02	6.35E-01	9.82E-01	9.04E-01	9.24E-01	small conductance mechanosensitive channel
KO:K03445	0.00	0.00	0.00	0.10	0.00	0.00	0.00	1.00E+00	1.00E+00	1.00E+00	1.43E-01	1.00E+00	1.00E+00	1.00E+00	MFS transporter, DHA1 family, purine ribonucleoside efflux pump
KO:K03446	0.26	0.10	0.24	0.98	1.29	0.00	0.05	3.94E-01	5.56E-01	4.13E-01	1.82E-01	1.35E-01	1.00E+00	6.19E-01	MFS transporter, DHA2 family, multidrug resistance protein B
KO:K03449	0.00	0.01	0.00	0.92	0.00	0.00	0.00	1.00E+00	2.38E-01	1.00E+00	6.59E-02	1.00E+00	1.00E+00	1.00E+00	MFS transporter, CP family, cyanate transporter
KO:K03451	5.87	1.87	1.33	0.76	0.00	0.07	0.00	3.91E-02	2.52E-01	3.77E-01	5.79E-01	1.00E+00	8.88E-01	9.41E-01	betaine/carnitine transporter, BCCT family
KO:K03453	0.38	1.27	3.80	4.33	2.36	0.87	0.20	8.34E-01	5.53E-01	1.29E-01	1.07E-01	2.84E-01	6.86E-01	8.84E-01	bile acid:Na+ symporter, BASS family
KO:K03455	0.57	2.44	0.86	3.41	0.18	1.27	0.54	7.83E-01	1.90E-01	6.75E-01	9.66E-02	9.02E-01	4.96E-01	7.94E-01	monovalent cation:H+ antiporter-2, CPA2 family
KO:K03457	0.39	1.74	2.47	1.97	0.70	0.00	0.00	5.91E-01	2.34E-01	1.61E-01	2.07E-01	4.67E-01	1.00E+00	1.00E+00	nucleobase:cation symporter-1, NCS1 family
KO:K03458	2.37	27.96	0.00	1.53	0.00	0.00	0.00	5.06E-01	1.43E-03	1.00E+00	6.61E-01	1.00E+00	1.00E+00	1.00E+00	nucleobase:cation symporter-2, NCS2 family
KO:K03462	0.06	0.35	0.00	0.01	0.00	0.00	0.00	2.63E-01	9.36E-02	1.00E+00	4.03E-01	1.00E+00	1.00E+00	1.00E+00	nicotinamide phosphoribosyltransferase [EC:2.4.2.12]
KO:K03463	0.00	1.44	0.00	0.00	0.00	0.00	0.00	1.00E+00	6.91E-02	1.00E+00	1.00E+00	1.00E+00	1.00E+00	1.00E+00	
KO:K03464	0.00	0.00	0.00	0.31	0.00	0.00	0.00	1.00E+00	1.00E+00	1.00E+00	1.43E-01	1.00E+00	1.00E+00	1.00E+00	muconolactone D-isomerase [EC:5.3.3.4]
KO:K03465	0.29	8.41	0.00	1.15	10.64	0.00	0.07	8.17E-01	4.27E-02	1.00E+00	5.86E-01	2.53E-02	1.00E+00	8.75E-01	thymidylate synthase (FAD) [EC:2.1.1.148]
KO:K03466	9.49	11.11	11.58	6.14	0.62	6.18	3.04	1.46E-01	6.97E-02	5.87E-02	6.51E-01	9.96E-01	6.47E-01	9.56E-01	DNA segregation ATPase FtsK/SpoIIIE, S-DNA-T family
KO:K03469	3.68	2.00	2.24	3.90	2.98	0.21	3.26	2.44E-01	6.91E-01	6.24E-01	2.09E-01	4.04E-01	9.82E-01	3.30E-01	ribonuclease HI [EC:3.1.26.4]
KO:K03470	4.17	2.49	1.10	1.28	3.05	1.25	0.08	8.13E-02	2.74E-01	7.58E-01	6.97E-01	1.69E-01	7.07E-01	9.87E-01	ribonuclease HII [EC:3.1.26.4]
KO:K03471	0.00	0.00	12.80	0.00	3.20	0.00	0.05	1.00E+00	1.00E+00	7.00E-03	1.00E+00	6.66E-02	1.00E+00	4.03E-01	ribonuclease HIII [EC:3.1.26.4]
KO:K03472	6.03	0.10	0.00	0.00	0.00	0.00	0.15	2.46E-02	5.32E-01	1.00E+00	6.68E-01	1.00E+00	1.00E+00	4.75E-01	D-erythrose 4-phosphate dehydrogenase [EC:1.2.1.72]
KO:K03473	3.70	0.09	0.02	0.12	0.00	0.00	2.65	3.33E-02	7.76E-01	8.43E-01	7.51E-01	1.00E+00	1.00E+00	5.57E-02	erythronate-4-phosphate dehydrogenase [EC:1.1.1.29]
KO:K03474	3.27	6.48	5.90	3.04	4.82	1.26	2.33	6.50E-01	9.86E-02	1.32E-01	7.04E-01	2.57E-01	9.48E-01	8.36E-01	pyridoxine 5-phosphate synthase [EC:2.6.99.2]
KO:K03477	0.02	0.57	0.00	0.00	0.00	0.00	0.00	2.84E-01	4.47E-02	1.00E+00	1.00E+00	1.00E+00	1.00E+00	1.00E+00	DeoR family transcriptional regulator, ulaG and ulaABCDEF operon
KO:K03478	0.05	0.00	1.77	2.76	0.00	0.00	0.00	4.68E-01	1.00E+00	1.18E-01	7.97E-02	1.00E+00	1.00E+00	1.00E+00	hypothetical protein
KO:K03490	0.00	0.33	0.00	0.00	0.00	0.00	0.00	1.00E+00	5.73E-02	1.00E+00	1.00E+00	1.00E+00	1.00E+00	1.00E+00	AraC family transcriptional regulator, cel operon repressor
KO:K03495	11.11	12.39	6.08	10.06	2.48	7.51	2.07	9.81E-02	5.46E-02	8.46E-01	1.70E-01	9.90E-01	6.12E-01	9.93E-01	glucose inhibited division protein A
KO:K03496	9.94	7.67	14.25	5.70	1.04	3.53	1.04	7.51E-02	2.13E-01	1.74E-02	5.32E-01	9.90E-01	8.86E-01	9.90E-01	chromosome partitioning protein
KO:K03497	6.89	5.67	4.93	2.81	0.62	5.00	0.52	7.53E-02	1.41E-01	2.23E-01	7.28E-01	9.75E-01	2.12E-01	9.81E-01	chromosome partitioning protein, ParB family
KO:K03498	5.41	19.12	3.76	5.14	0.00	3.11	3.06	5.07E-01	5.29E-03	7.93E-01	5.55E-01	9.98E-01	8.66E-01	8.71E-01	trk system potassium uptake protein TrkH
KO:K03499	5.60	11.26	4.04	5.43	3.78	6.13	2.24	5.41E-01	5.34E-02	8.06E-01	5.75E-01	8.39E-01	4.37E-01	9.43E-01	trk system potassium uptake protein TrkA
KO:K03500	3.29	17.41	0.12	1.36	6.17	0.00	0.12	4.14E-01	3.00E-03	9.89E-01	8.64E-01	8.83E-02	1.00E+00	9.89E-01	ribosomal RNA small subunit methyltransferase B [EC:2.1.1.-]
KO:K03501	4.59	2.73	2.05	3.03	0.33	0.91	0.06	5.76E-02	2.46E-01	4.74E-01	1.86E-01	9.65E-01	8.77E-01	9.92E-01	glucose inhibited division protein B [EC:2.1.-.-]
KO:K03502	0.62	0.35	0.00	1.40	2.50	9.66	0.05	5.54E-01	6.78E-01	1.00E+00	3.09E-01	1.45E-01	1.46E-02	8.43E-01	DNA polymerase V
KO:K03503	0.15	0.22	0.00	0.92	1.09	3.04	0.03	6.22E-01	5.64E-01	1.00E+00	2.14E-01	1.72E-01	4.69E-02	7.73E-01	DNA polymerase V [EC:3.4.21.-]
KO:K03517	4.24	0.31	13.72	3.98	6.26	0.02	4.21	2.95E-01	9.44E-01	1.36E-02	3.34E-01	1.19E-01	9.86E-01	2.99E-01	quinolinate synthase [EC:2.5.1.72]
KO:K03518	0.93	21.08	0.50	4.78	0.00	7.30	0.00	8.71E-01	8.57E-03	9.11E-01	2.96E-01	1.00E+00	1.25E-01	1.00E+00	carbon-monoxide dehydrogenase small subunit [EC:1.2.99.2]
KO:K03519	1.19	24.65	2.04	4.96	0.00	3.63	0.00	8.70E-01	5.57E-03	7.73E-01	3.19E-01	1.00E+00	5.07E-01	1.00E+00	carbon-monoxide dehydrogenase medium subunit [EC:1.2.99.2]
KO:K03520	0.61	25.49	0.00	3.18	0.00	7.54	0.00	8.58E-01	4.00E-03	1.00E+00	4.55E-01	1.00E+00	1.07E-01	1.00E+00	carbon-monoxide dehydrogenase large subunit [EC:1.2.99.2]
KO:K03521	15.44	25.03	1.53	9.07	0.00	2.37	0.00	2.81E-02	8.57E-04	9.73E-01	2.25E-01	1.00E+00	9.53E-01	1.00E+00	electron transfer flavoprotein beta subunit
KO:K03522	16.56	21.57	1.76	8.05	0.00	1.23	0.31	1.97E-02	3.14E-03	9.67E-01	2.97E-01	1.00E+00	9.79E-01	9.93E-01	electron transfer flavoprotein alpha subunit

KO:K03523	0.72	11.60	0.00	2.08	4.96	0.00	0.00	7.65E-01	1.83E-02	1.00E+00	4.32E-01	1.17E-01	1.00E+00	1.00E+00	putative biotin biosynthesis protein BioY
KO:K03524	2.32	3.14	7.10	1.30	3.62	0.03	0.46	3.89E-01	1.90E-01	1.84E-02	7.60E-01	1.31E-01	9.95E-01	9.35E-01	BirA family transcriptional regulator, biotin operon repressor /
KO:K03525	4.16	10.06	0.40	2.18	1.89	4.48	0.98	3.14E-01	2.73E-02	9.74E-01	7.74E-01	8.24E-01	2.60E-01	9.29E-01	type III pantothenate kinase [EC:2.7.1.33]
KO:K03526	7.82	4.61	9.94	5.08	8.76	0.26	5.93	1.76E-01	7.02E-01	7.26E-02	6.09E-01	1.14E-01	9.97E-01	4.33E-01	(E)-4-hydroxy-3-methylbut-2-enyl-diphosphate synthase [EC:1.17.7.1]
KO:K03527	6.43	3.59	5.86	4.69	10.66	1.04	1.55	1.75E-01	7.08E-01	2.41E-01	4.56E-01	3.10E-02	9.71E-01	9.45E-01	4-hydroxy-3-methylbut-2-enyl diphosphate reductase [EC:1.17.1.2]
KO:K03528	9.03	0.00	0.17	0.07	0.00	0.55	0.00	1.49E-02	8.19E-01	6.50E-01	7.43E-01	1.00E+00	4.20E-01	1.00E+00	cell division protein ZipA
KO:K03529	9.56	7.32	6.21	5.71	4.26	5.70	0.82	1.06E-01	2.71E-01	4.30E-01	5.17E-01	7.58E-01	5.19E-01	9.81E-01	chromosome segregation protein
KO:K03530	11.20	4.82	6.94	5.79	2.96	12.20	1.79	9.00E-02	7.11E-01	3.86E-01	5.60E-01	8.97E-01	6.93E-02	9.54E-01	DNA-binding protein HU-beta
KO:K03531	10.69	12.85	6.73	7.75	4.90	5.75	1.38	1.36E-01	5.97E-02	6.64E-01	4.83E-01	8.80E-01	8.06E-01	9.89E-01	cell division protein FtsZ
KO:K03532	0.00	0.00	0.07	0.00	0.00	4.37	0.00	1.00E+00	1.00E+00	2.06E-01	3.43E-01	1.00E+00	2.86E-03	1.00E+00	trimethylamine-N-oxide reductase (cytochrome c) 1, cytochrome c-type
KO:K03534	0.04	0.56	2.62	0.04	0.00	0.00	1.16	6.16E-01	2.64E-01	4.76E-02	6.15E-01	1.00E+00	1.00E+00	1.25E-01	L-rhamnose mutarotase [EC:5.1.3.-]
KO:K03535	0.03	0.00	0.00	0.00	0.00	0.00	0.33	2.12E-01	1.00E+00	1.00E+00	1.00E+00	1.00E+00	1.00E+00	4.59E-02	MFS transporter, ACS family, glucarate transporter
KO:K03536	4.17	3.04	1.49	1.99	7.44	0.22	1.35	1.72E-01	3.69E-01	7.95E-01	6.61E-01	3.17E-02	9.69E-01	8.21E-01	ribonuclease P protein component [EC:3.1.26.5]
KO:K03538	0.00	0.00	0.00	0.00	0.00	0.00	1.25	1.00E+00	1.00E+00	1.00E+00	1.00E+00	1.00E+00	1.00E+00	2.43E-02	ribonuclease P subunit P29 [EC:3.1.26.5]
KO:K03540	0.00	0.00	0.00	0.00	0.00	0.00	1.25	1.00E+00	1.00E+00	1.00E+00	1.00E+00	1.00E+00	1.00E+00	2.49E-02	ribonuclease P subunit RPR2 [EC:3.1.26.5]
KO:K03543	2.25	0.03	0.13	0.63	0.30	0.00	0.00	8.06E-02	6.93E-01	5.36E-01	2.60E-01	3.97E-01	1.00E+00	1.00E+00	multidrug resistance protein A
KO:K03544	9.54	10.16	16.26	10.65	4.46	5.53	3.19	3.32E-01	2.40E-01	9.86E-03	1.84E-01	9.64E-01	9.26E-01	9.85E-01	ATP-dependent Clp protease ATP-binding subunit ClpX
KO:K03545	9.22	11.03	15.98	7.00	6.62	8.45	4.67	4.13E-01	1.65E-01	1.69E-02	8.07E-01	8.45E-01	5.65E-01	9.51E-01	trigger factor
KO:K03546	0.30	0.01	0.00	0.19	0.00	0.00	1.56	2.30E-01	6.37E-01	1.00E+00	3.17E-01	1.00E+00	1.00E+00	4.50E-02	exonuclease SbcC
KO:K03547	0.65	0.00	0.00	0.06	0.00	1.84	0.00	9.06E-02	6.71E-01	1.00E+00	4.90E-01	1.00E+00	2.10E-02	1.00E+00	exonuclease SbcD
KO:K03548	11.33	0.40	1.79	0.53	0.00	9.35	0.41	2.27E-02	8.33E-01	5.03E-01	8.13E-01	1.00E+00	3.83E-02	8.33E-01	putative permease
KO:K03549	0.47	0.00	0.05	3.25	0.00	0.00	0.00	2.72E-01	1.00E+00	4.95E-01	6.26E-02	1.00E+00	1.00E+00	1.00E+00	KUP system potassium uptake protein
KO:K03550	5.78	5.52	7.51	1.15	1.06	4.36	1.16	1.30E-01	1.48E-01	6.40E-02	9.16E-01	9.28E-01	2.80E-01	9.15E-01	holliday junction DNA helicase RuvA
KO:K03551	9.64	4.39	6.93	3.08	3.35	1.42	1.96	4.31E-02	4.95E-01	1.29E-01	7.80E-01	7.30E-01	9.47E-01	9.12E-01	holliday junction DNA helicase RuvB
KO:K03553	12.37	9.70	15.55	12.13	7.55	2.68	0.71	8.60E-02	3.19E-01	2.01E-02	9.46E-02	7.30E-01	9.88E-01	9.98E-01	recombination protein RecA
KO:K03554	2.91	0.00	0.00	0.10	0.00	10.07	0.00	7.46E-02	7.81E-01	1.00E+00	6.30E-01	1.00E+00	9.86E-03	1.00E+00	recombination associated protein RdgC
KO:K03555	4.89	7.64	9.31	3.03	4.72	3.59	1.24	4.31E-01	8.13E-02	3.59E-02	8.53E-01	4.79E-01	7.66E-01	9.76E-01	DNA mismatch repair protein MutS
KO:K03556	0.00	0.00	0.25	0.01	0.00	0.00	0.45	1.00E+00	1.00E+00	7.80E-02	3.32E-01	1.00E+00	1.00E+00	3.01E-02	LuxR family transcriptional regulator, maltose regulon positive
KO:K03557	3.19	0.01	1.14	0.68	0.00	0.00	0.79	4.43E-02	8.72E-01	2.13E-01	3.84E-01	1.00E+00	1.00E+00	3.31E-01	Fis family transcriptional regulator, factor for inversion
KO:K03558	8.65	8.60	1.72	2.31	0.00	1.88	0.77	4.51E-02	4.57E-02	8.29E-01	7.26E-01	1.00E+00	8.05E-01	9.29E-01	membrane protein required for colicin V production
KO:K03559	11.50	1.62	17.83	2.39	0.43	1.56	0.78	4.70E-02	8.57E-01	1.16E-02	7.63E-01	9.62E-01	8.66E-01	9.37E-01	biopolymer transport protein ExbD
KO:K03560	6.33	0.68	0.21	0.82	0.00	0.98	1.10	4.31E-02	6.50E-01	8.08E-01	5.92E-01	1.00E+00	5.36E-01	4.95E-01	biopolymer transport protein TolR
KO:K03561	15.90	0.96	26.66	4.71	0.73	3.62	2.21	3.44E-02	9.51E-01	4.29E-03	6.23E-01	9.60E-01	7.64E-01	8.89E-01	biopolymer transport protein ExbB
KO:K03562	6.29	9.83	8.10	4.14	0.56	0.39	2.23	2.39E-01	6.44E-02	1.14E-01	5.72E-01	9.67E-01	9.72E-01	8.56E-01	biopolymer transport protein TolQ
KO:K03563	2.07	0.08	31.66	0.35	0.21	1.71	0.58	3.38E-01	8.74E-01	4.29E-04	7.95E-01	8.28E-01	4.08E-01	7.29E-01	carbon storage regulator
KO:K03564	3.62	2.25	6.37	2.96	17.64	3.07	1.22	5.76E-01	8.39E-01	1.38E-01	7.21E-01	2.57E-03	6.98E-01	9.38E-01	peroxiredoxin Q/BCP [EC:1.11.1.15]
KO:K03565	5.48	0.21	0.00	2.20	0.00	1.63	0.92	3.07E-02	8.65E-01	1.00E+00	1.87E-01	1.00E+00	3.01E-01	5.63E-01	regulatory protein
KO:K03566	0.68	5.76	0.00	0.29	0.00	0.00	0.00	3.45E-01	1.87E-02	1.00E+00	5.79E-01	1.00E+00	1.00E+00	1.00E+00	LysR family transcriptional regulator, glycine cleavage system
KO:K03567	0.39	0.00	0.00	0.71	0.00	0.00	0.56	2.32E-01	1.00E+00	1.00E+00	1.42E-01	1.00E+00	1.00E+00	1.77E-01	glycine cleavage system transcriptional repressor
KO:K03568	2.74	7.78	0.19	5.81	6.76	0.43	4.80	7.64E-01	7.77E-02	9.85E-01	1.92E-01	1.22E-01	9.76E-01	3.28E-01	TldD protein
KO:K03569	11.36	8.80	11.50	7.61	5.86	5.13	4.76	1.42E-01	4.26E-01	1.36E-01	6.21E-01	8.42E-01	8.90E-01	9.08E-01	rod shape-determining protein MreB and related proteins
KO:K03570	7.14	1.89	2.54	1.77	3.80	0.40	1.05	4.44E-02	6.75E-01	4.81E-01	7.10E-01	2.12E-01	9.53E-01	8.65E-01	rod shape-determining protein MreC
KO:K03571	3.34	0.61	0.00	0.32	0.00	3.65	1.05	7.79E-02	5.26E-01	1.00E+00	6.87E-01	1.00E+00	6.91E-02	3.53E-01	rod shape-determining protein MreD
KO:K03572	9.02	8.71	8.52	2.37	0.92	1.04	0.45	5.09E-02	5.57E-02	5.97E-02	8.56E-01	9.64E-01	9.58E-01	9.84E-01	DNA mismatch repair protein MutL
KO:K03573	0.00	0.00	0.00	0.31	0.00	0.00	0.00	1.00E+00	1.00E+00	1.00E+00	7.36E-02	1.00E+00	1.00E+00	1.00E+00	DNA mismatch repair protein MutH
KO:K03574	2.98	5.38	1.75	1.04	1.92	0.56	1.05	2.68E-01	6.90E-02	6.07E-01	8.06E-01	5.52E-01	8.96E-01	8.04E-01	7,8-dihydro-8-oxoguanine triphosphatase [EC:3.6.1.-]
KO:K03575	2.46	2.30	1.84	1.73	5.47	0.52	0.13	2.44E-01	2.96E-01	4.89E-01	5.37E-01	1.93E-02	9.45E-01	9.90E-01	A/G-specific adenine glycosylase [EC:3.2.2.-]
KO:K03576	0.52	23.86	0.17	0.00	0.00	0.00	0.00	7.35E-01	7.14E-04	8.41E-01	9.21E-01	1.00E+00	1.00E+00	9.21E-01	LysR family transcriptional regulator, regulator for metE and metH
KO:K03578	4.73	0.15	4.83	1.11	1.51	1.90	1.56	9.11E-02	9.05E-01	8.83E-02	6.52E-01	5.26E-01	4.16E-01	5.10E-01	ATP-dependent helicase HrpA [EC:3.6.1.-]
KO:K03579	0.09	1.68	9.50	0.06	5.15	0.00	0.19	9.11E-01	1.65E-01	5.00E-03	9.43E-01	1.83E-02	1.00E+00	8.71E-01	ATP-dependent helicase HrpB [EC:3.6.1.-]
KO:K03580	2.60	0.03	6.25	0.23	0.56	1.23	0.79	1.29E-01	8.63E-01	3.31E-02	7.51E-01	5.95E-01	3.39E-01	4.93E-01	ATP-dependent helicase HepA [EC:3.6.1.-]
KO:K03581	0.11	0.02	12.08	0.09	2.04	0.00	0.68	6.99E-01	8.08E-01	5.86E-03	7.36E-01	9.11E-02	1.00E+00	3.11E-01	exodeoxyribonuclease V alpha subunit [EC:3.1.11.5]
KO:K03582	0.02	0.01	0.41	0.18	5.98	0.00	1.24	7.75E-01	8.02E-01	3.36E-01	5.57E-01	6.29E-03	1.00E+00	9.41E-02	exodeoxyribonuclease V beta subunit [EC:3.1.11.5]
KO:K03583	0.10	0.00	1.53	0.09	8.12	0.00	0.11	6.19E-01	1.00E+00	7.57E-02	6.35E-01	4.29E-03	1.00E+00	6.12E-01	exodeoxyribonuclease V gamma subunit [EC:3.1.11.5]
KO:K03584	3.09	3.88	0.33	1.66	0.43	1.73	0.00	1.25E-01	7.14E-02	9.25E-01	4.31E-01	8.98E-01	4.02E-01	9.91E-01	DNA repair protein RecO (recombination protein O)
KO:K03585	0.30	0.12	0.54	0.36	0.00	0.00	0.11	2.62E-01	5.14E-01	1.26E-01	2.13E-01	1.00E+00	1.00E+00	5.41E-01	membrane fusion protein
KO:K03586	1.36	0.00	0.34	0.23	0.00	3.77	0.01	1.12E-01	7.62E-01	3.88E-01	4.69E-01	1.00E+00	3.19E-02	7.50E-01	cell division protein FtsL
KO:K03587	6.61	6.16	7.64	3.79	3.18	2.39	0.85	1.58E-01	1.94E-01	9.76E-02	6.39E-01	7.68E-01	8.75E-01	9.74E-01	cell division protein FtsI (penicillin-binding protein 3)
KO:K03588	5.90	15.76	5.04	3.39	4.49	0.82	1.54	3.85E-01	5.43E-03	5.57E-01	8.46E-01	6.80E-01	9.87E-01	9.67E-01	cell division protein FtsW
KO:K03589	2.91	8.45	1.52	1.22	1.87	0.53	0.01	2.90E-01	1.69E-02	7.02E-01	7.86E-01	5.87E-01	9.17E-01	9.92E-01	cell division protein FtsQ
KO:K03590	10.06	8.14	5.31	5.20	0.43	4.36	0.97	6.74E-02	1.35E-01	4.57E-01	4.76E-01	9.87E-01	6.35E-01	9.69E-01	cell division protein FtsA
KO:K03592	1.72	8.97	0.10	3.74	5.12	3.33	1.51	8.23E-01	4.33E-02	9.79E-01	3.97E-01	1.92E-01	4.84E-01	8.54E-01	PmbA protein
KO:K03593	6.50	10.26	7.18	7.40	4.18	6.71	6.26	6.22E-01	1.15E-01	4.86E-01	4.42E-01	9.09E-01	5.81E-01	6.68E-01	ATP-binding protein involved in chromosome partitioning
KO:K03594	6.14	0.07	1.76	1.90	5.58	0.00	0.29	5.64E-02	9.06E-01	4.09E-01	3.75E-01	6.90E-02	1.00E+00	8.46E-01	bacterioferritin
KO:K03595	6.69	7.78	0.01	2.46	3.28	1.73	1.46	6.49E-02	3.96E-02	9.99E-01	7.47E-01	5.10E-01	8.77E-01	9.08E-01	GTP-binding protein Era
KO:K03596	12.88	10.76	8.44	6.68	5.65	6.97	2.37	4.61E-02	1.23E-01	4.14E-01	7.61E-01	8.75E-01	7.12E-01	9.87E-01	GTP-binding protein LepA
KO:K03597	9.79	0.00	0.00	0.12	0.00	0.00	1.36	1.71E-02	1.00E+00	1.00E+00	6.65E-01	1.00E+00	1.00E+00	2.59E-01	sigma-E factor negative regulatory protein RseA
KO:K03598	2.46	0.00	0.88	3.28	0.00	0.13	0.00	9.47E-02	1.00E+00	3.52E-01	6.17E-02	1.00E+00	7.65E-01	1.00E+00	sigma-E factor negative regulatory protein RseB
KO:K03599	11.40	0.37	2.78	2.19	0.00	0.13	3.16	2.93E-02	8.45E-01	3.65E-01	4.70E-01	1.00E+00	8.81E-01	3.13E-01	stringent starvation protein A
KO:K03600	9.68	0.02	0.35	0.06	0.00	2.51	0.44	1.67E-02	8.58E-01	6.60E-01	8.24E-01	1.00E+00	1.52E-01	6.24E-01	stringent starvation protein B
KO:K03601	3.49	4.02	1.81	1.39	9.52	1.43	0.20	2.63E-01	1.87E-01	7.11E-01	8.17E-01	1.73E-02	8.07E-01	9.75E-01	exodeoxyribonuclease VII large subunit [EC:3.1.11.6]
KO:K03602	2.79	3.03	0.06	0.87	1.92	1.18	0.36	1.72E-01	1.47E-01	9.39E-01	6.65E-01	3.08E-01	5.46E-01	8.52E-01	exodeoxyribonuclease VII small subunit [EC:3.1.11.6]
KO:K03603	0.49	0.00	0.00	0.02	0.00	0.00	0.00	4.51E-02	1.00E+00	1.00E+00	2.84E-01	1.00E+00	1.00E+00	1.00E+00	GntR family transcriptional regulator, negative regulator for fad
KO:K03605	0.00	0.15	0.00	0.03	0.00	0.00	0.00	1.00E+00	1.11E-01	1.00E+00	2.23E-01	1.00E+00	1.00E+00	1.00E+00	hydrogenase 1 maturation protease [EC:3.4.24.-]
KO:K03606	0.32	0.17	15.02	0.23	0.26	0.00	0.07	5.58E-01	6.74E-01	2.43E-03	6.26E-01	6.06E-01	1.00E+00	7.77E-01	putative colanic acid biosynthesis UDP-glucose lipid carrier
KO:K03607	0.38	0.00	0.00	0.02	0.00	0.00	0.00	3.61E-02	1.00E+00	1.00E+00	3.30E-01	1.00E+00	1.00E+00	1.00E+00	ProP effector
KO:K03608	0.23	0.00	0.00	0.01	9.63	0.00	1.26	4.14E-01	7.30E-01	1.00E+00	7.03E-01	4.57E-03	1.00E+00	1.16E-01	cell division topological specificity factor
KO:K03609	0.21	0.01	0.01	0.10	13.85	0.32	0.41	5.76E-01	8.51E-01	8.40E-01	7.13E-01	7.14E-04	4.76E-01	4.18E-01	septum site-determining protein MinD
KO:K03610	0.08	0.01	0.00	0.02	7.35	0.00	0.00	4.68E-01	6.70E-01	1.00E+00	6.55E-01	5.71E-04	1.00E+00	1.00E+00	septum site-determining protein MinC
KO:K03611	5.03	0.14	1.41	3.26	0.00	0.28	0.04	7.30E-02	8.15E-01	3.83E-01	1.40E-01	1.00E+00	7.64E-01	8.54E-01	disulfide bond formation protein DsbB
KO:K03612	2.85	0.00	0.11	0.30	0.00	0.20	0.45	4.33E-02	8.39E-01	6.47E-01	4.47E-01	1.00E+00	5.38E-01	3.54E-01	electron transport complex protein RnfG
KO:K03613	4.23	0.00	0.00	0.34	0.00	0.10	4.92	5.06E-02	8.47E-01	1.00E+00	5.78E-01	1.00E+00	7.33E-01	4.09E-02	electron transport complex protein RnfE
KO:K03614	4.54	0.02	0.01	0.39	0.00	0.10	3.87	3.23E-02	8.61E-01	8.65E-01	5.97E-01	1.00E+00	7.99E-01	4.21E-02	electron transport complex protein RnfD
KO:K03615	1.92	0.01	1.00	0.50	0.00	0.35	3.43	1.25E-01	8.80E-01	2.82E-01	4.97E-01	1.00E+00	5.91E-01	5.36E-02	electron transport complex protein RnfC
KO:K03616	3.88	0.02	1.19	0.73	0.00	0.00	2.80	6.23E-02	8.78E-01	3.02E-01	4.70E-01	1.00E+00	1.00E+00	9.94E-02	electron transport complex protein RnfB
KO:K03617	4.65	0.02	0.05	1.00	0.00	0.00	4.78	5.89E-02	8.34E-01	8.20E-01	3.93E-01	1.00E+00	1.00E+00	5.63E-02	electron transport complex protein RnfA
KO:K03618	0.12	0.00	0.00	0.00	0.00	0.00	0.00	5.97E-02	1.00E+00	1.00E+00	1.00E+00	1.00E+00	1.00E+00	1.00E+00	hydrogenase-1 operon protein HyaF
KO:K03621	2.00	7.84	8.28	5.49	10.74	1.37	3.09	9.16E-01	1.41E-01	1.18E-01	3.97E-01	5.29E-02	9.48E-01	8.25E-01	glycerol-3-phosphate acyltransferase PlsX [EC:2.3.1.15]
KO:K03622	0.00	0.00	0.00	0.00	0.00	0.00	9.17	1.00E+00	1.00E+00	1.00E+00	1.00E+00	1.00E+00	1.00E+00	5.71E-03	archaea-specific DNA-binding protein
KO:K03624	9.20	5.87	0.50	8.42	0.01	4.10	1.32	4.66E-02	2.14E-01	9.81E-01	6.43E-02	9.99E-01	5.36E-01	9.45E-01	transcription elongation factor GreA
KO:K03625	3.85	5.28	3.47	1.16	8.61	0.00	0.88	2.52E-01	1.17E-01	3.10E-01	8.51E-01	3.43E-02	1.00E+00	8.89E-01	N utilization substance protein B
KO:K03626	0.00	0.00	0.00	0.00	0.00	0.00	3.33	1.00E+00	1.00E+00	1.00E+00	1.00E+00	1.00E+00	1.00E+00	1.47E-02	nascent polypeptide-associated complex subunit alpha
KO:K03628	8.03	11.02	15.80	8.47	1.45	4.18	4.05	4.83E-01	1.29E-01	2.09E-02	4.03E-01	9.92E-01	9.36E-01	9.40E-01	transcription termination factor Rho
KO:K03629	6.20	2.40	5.46	2.13	1.81	1.97	0.01	5.46E-02	5.31E-01	7.99E-02	6.12E-01	7.12E-01	6.66E-01	9.97E-01	DNA replication and repair protein RecF
KO:K03630	0.39	0.25	10.48	0.10	0.10	0.33	0.05	4.66E-01	5.78E-01	4.86E-03	7.75E-01	7.67E-01	5.08E-01	8.63E-01	DNA repair protein RadC
KO:K03631	4.40	9.31	1.75	1.94	6.20	1.28	0.10	2.40E-01	2.19E-02	8.73E-01	8.48E-01	8.09E-02	9.23E-01	9.93E-01	DNA repair protein RecN (Recombination protein N)

KO:K03634	2.06	0.44	0.06	1.80	0.00	0.11	0.99	1.27E-01	6.17E-01	8.60E-01	1.54E-01	1.00E+00	8.15E-01	3.40E-01	outer membrane lipoprotein carrier protein
KO:K03635	3.86	2.62	0.31	1.98	3.52	0.14	1.45	1.22E-01	2.84E-01	9.47E-01	4.76E-01	1.55E-01	9.70E-01	6.68E-01	molybdenum cofactor biosynthesis protein E
KO:K03636	2.70	2.58	0.17	1.05	0.85	0.00	0.23	1.26E-01	1.36E-01	8.69E-01	4.56E-01	5.40E-01	1.00E+00	8.35E-01	molybdenum cofactor biosynthesis protein D
KO:K03637	3.53	2.89	1.61	3.60	2.79	0.00	0.88	1.89E-01	3.02E-01	7.04E-01	1.78E-01	3.24E-01	1.00E+00	8.83E-01	molybdenum cofactor biosynthesis protein C
KO:K03638	5.62	4.09	0.51	3.72	0.87	0.04	0.33	8.23E-02	1.68E-01	9.04E-01	2.08E-01	8.45E-01	9.79E-01	9.34E-01	molybdenum cofactor biosynthesis protein B
KO:K03639	4.11	11.54	2.07	6.16	1.94	3.01	0.81	5.85E-01	2.91E-02	8.94E-01	2.30E-01	9.04E-01	7.97E-01	9.68E-01	molybdenum cofactor biosynthesis protein A
KO:K03640	7.99	10.06	5.77	10.47	0.00	0.23	3.60	1.79E-01	8.60E-02	4.61E-01	7.60E-02	1.00E+00	9.92E-01	8.11E-01	peptidoglycan-associated lipoprotein
KO:K03641	8.91	13.29	3.51	6.70	0.00	0.41	1.64	9.97E-02	2.70E-02	7.62E-01	2.35E-01	1.00E+00	9.84E-01	9.32E-01	TolB protein
KO:K03642	4.01	0.05	1.25	1.70	4.13	1.13	0.00	8.77E-02	9.09E-01	4.62E-01	3.27E-01	8.41E-02	5.05E-01	9.35E-01	rare lipoprotein A
KO:K03643	2.70	0.62	0.00	0.64	0.00	0.00	0.54	6.63E-02	3.55E-01	1.00E+00	3.45E-01	1.00E+00	1.00E+00	3.93E-01	LPS-assembly lipoprotein
KO:K03644	8.12	13.67	5.44	4.35	4.28	8.78	0.48	2.05E-01	2.14E-02	6.79E-01	8.45E-01	8.53E-01	1.47E-01	9.96E-01	lipoic acid synthetase [EC:2.8.1.8]
KO:K03646	3.19	0.07	0.72	0.14	0.00	0.95	0.80	5.41E-02	7.89E-01	3.55E-01	7.21E-01	1.00E+00	2.75E-01	3.26E-01	colicin import membrane protein
KO:K03648	3.58	0.09	0.88	0.15	0.00	0.39	1.38	2.83E-02	8.67E-01	3.02E-01	8.05E-01	1.00E+00	6.10E-01	1.56E-01	uracil-DNA glycosylase [EC:3.2.2.-]
KO:K03649	0.01	0.00	0.00	0.07	0.00	0.00	0.55	3.08E-01	1.00E+00	1.00E+00	2.12E-01	1.00E+00	1.00E+00	5.31E-02	TDG/mug DNA glycosylase family protein [EC:3.2.2.-]
KO:K03650	5.73	7.37	2.04	3.52	5.65	0.58	2.51	1.70E-01	6.81E-02	9.16E-01	6.60E-01	1.77E-01	9.91E-01	8.62E-01	tRNA modification GTPase
KO:K03651	4.49	0.00	0.92	0.12	1.74	0.00	0.16	4.21E-02	8.64E-01	3.00E-01	7.34E-01	1.47E-01	1.00E+00	7.01E-01	Icc protein
KO:K03652	0.00	0.00	7.92	0.00	6.15	0.00	0.05	6.61E-01	1.00E+00	5.71E-03	1.00E+00	1.09E-02	1.00E+00	5.83E-01	DNA-3-methyladenine glycosylase [EC:3.2.2.21]
KO:K03654	3.37	8.29	1.03	2.03	4.02	3.30	0.58	4.40E-01	4.37E-02	9.13E-01	7.66E-01	3.05E-01	4.57E-01	9.55E-01	ATP-dependent DNA helicase RecQ [EC:3.6.1.-]
KO:K03655	3.62	4.38	1.34	3.14	4.22	3.87	0.83	3.19E-01	1.71E-01	9.18E-01	4.66E-01	1.96E-01	2.57E-01	9.62E-01	ATP-dependent DNA helicase RecG [EC:3.6.1.-]
KO:K03656	0.86	0.09	1.39	0.20	3.36	0.00	0.01	2.23E-01	7.41E-01	1.16E-01	6.01E-01	3.11E-02	1.00E+00	8.45E-01	ATP-dependent DNA helicase Rep [EC:3.6.1.-]
KO:K03657	7.56	9.34	11.31	3.91	7.13	1.45	1.94	1.94E-01	7.99E-02	3.37E-02	8.68E-01	2.52E-01	9.87E-01	9.78E-01	DNA helicase II / ATP-dependent DNA helicase PcrA [EC:3.6.1.-]
KO:K03658	0.69	0.07	0.00	0.03	0.00	0.00	0.00	5.20E-02	3.23E-01	1.00E+00	4.23E-01	1.00E+00	1.00E+00	1.00E+00	DNA helicase IV [EC:3.6.1.-]
KO:K03660	0.00	0.00	6.35	0.00	0.62	0.00	0.40	5.35E-01	1.00E+00	6.00E-03	1.00E+00	1.46E-01	1.00E+00	1.97E-01	N-glycosylase/DNA lyase [EC:3.2.2.- 4.2.99.18]
KO:K03664	3.35	6.73	4.21	1.65	3.76	0.09	0.90	3.51E-01	6.44E-02	2.03E-01	7.88E-01	2.70E-01	9.88E-01	9.00E-01	SsrA-binding protein
KO:K03665	9.33	6.98	6.17	2.35	2.50	1.45	1.76	4.40E-02	1.25E-01	1.88E-01	8.85E-01	8.70E-01	9.55E-01	9.34E-01	GTP-binding protein HflX
KO:K03666	8.78	8.52	0.02	2.68	0.11	1.51	0.07	4.54E-02	4.97E-02	9.71E-01	5.19E-01	9.49E-01	7.64E-01	9.60E-01	host factor-I protein
KO:K03667	3.59	12.34	0.73	7.88	0.01	0.42	0.17	4.44E-01	1.14E-02	9.56E-01	5.46E-02	9.96E-01	9.76E-01	9.88E-01	ATP-dependent HslUV protease ATP-binding subunit HslU
KO:K03669	0.10	0.14	0.00	0.67	0.00	0.00	0.00	3.79E-01	3.05E-01	1.00E+00	8.13E-02	1.00E+00	1.00E+00	1.00E+00	membrane glycosyltransferase [EC:2.4.1.-]
KO:K03670	0.00	0.07	0.93	0.01	0.00	0.00	0.00	1.00E+00	2.38E-01	1.73E-02	4.40E-01	1.00E+00	1.00E+00	1.00E+00	periplasmic glucans biosynthesis protein
KO:K03671	4.96	5.70	5.38	7.66	9.31	1.40	2.04	5.78E-01	4.27E-01	4.86E-01	1.76E-01	8.81E-02	9.69E-01	9.43E-01	thioredoxin 1
KO:K03672	0.02	4.48	0.00	0.04	0.00	0.00	0.00	6.50E-01	1.99E-02	1.00E+00	5.87E-01	1.00E+00	1.00E+00	1.00E+00	thioredoxin 2 [EC:1.8.1.8]
KO:K03673	13.42	0.41	0.02	2.71	0.00	0.34	3.08	1.81E-02	8.44E-01	9.12E-01	3.59E-01	1.00E+00	8.55E-01	3.05E-01	thiol:disulfide interchange protein DsbA
KO:K03674	0.00	0.04	0.00	0.84	0.00	0.00	0.00	1.00E+00	2.42E-01	1.00E+00	6.83E-02	1.00E+00	1.00E+00	1.00E+00	glutaredoxin 1
KO:K03675	0.12	0.00	0.00	0.00	0.00	0.00	0.00	6.70E-02	1.00E+00	1.00E+00	1.00E+00	1.00E+00	1.00E+00	1.00E+00	glutaredoxin 2
KO:K03676	2.90	3.96	0.22	2.97	6.79	0.00	0.72	3.08E-01	1.62E-01	9.55E-01	2.94E-01	4.53E-02	1.00E+00	8.76E-01	glutaredoxin 3
KO:K03677	3.52	4.52	1.23	0.90	3.08	1.56	2.43	2.32E-01	1.36E-01	8.13E-01	8.76E-01	3.12E-01	7.34E-01	4.75E-01	CysQ protein
KO:K03679	0.00	0.00	0.00	0.00	0.00	0.00	6.67	1.00E+00	1.00E+00	1.00E+00	1.00E+00	1.00E+00	1.00E+00	4.86E-03	exosome complex component RRP4
KO:K03680	0.00	0.00	0.00	0.10	0.00	0.00	0.00	1.00E+00	1.00E+00	1.00E+00	1.43E-01	1.00E+00	1.00E+00	1.00E+00	translation initiation factor eIF-2B delta subunit
KO:K03683	6.24	0.24	0.41	0.16	0.00	2.16	1.66	3.31E-02	7.58E-01	6.89E-01	7.89E-01	1.00E+00	2.02E-01	2.86E-01	ribonuclease T [EC:3.1.13.-]
KO:K03684	4.50	17.94	2.74	2.21	6.77	0.00	0.12	4.03E-01	3.86E-03	7.62E-01	8.39E-01	1.40E-01	1.00E+00	9.86E-01	ribonuclease D [EC:3.1.13.5]
KO:K03685	6.05	7.98	1.94	2.73	2.24	0.08	2.46	9.60E-02	3.56E-02	8.80E-01	7.27E-01	8.34E-01	9.98E-01	7.89E-01	ribonuclease III [EC:3.1.26.3]
KO:K03686	9.85	12.80	6.97	7.53	8.67	4.25	3.73	2.00E-01	3.70E-02	7.88E-01	6.88E-01	4.13E-01	9.70E-01	9.80E-01	molecular chaperone DnaJ
KO:K03687	9.44	8.44	3.76	3.93	7.95	0.22	1.00	5.23E-02	8.07E-02	7.50E-01	7.19E-01	1.02E-01	9.96E-01	9.79E-01	molecular chaperone GrpE
KO:K03688	5.71	11.14	2.15	2.32	0.00	1.20	0.61	1.19E-01	1.71E-02	7.46E-01	7.06E-01	1.00E+00	8.96E-01	9.55E-01	ubiquinone biosynthesis protein
KO:K03689	0.00	0.00	0.00	0.00	18.13	0.00	0.00	1.00E+00	1.00E+00	1.00E+00	1.00E+00	0.00E+00	1.00E+00	1.00E+00	cytochrome b6-f complex subunit 8
KO:K03690	2.81	0.01	0.02	0.29	0.00	2.33	0.15	6.09E-02	8.19E-01	7.95E-01	4.94E-01	1.00E+00	7.66E-02	6.17E-01	hypothetical protein
KO:K03692	0.00	0.00	0.00	0.00	30.30	0.00	0.00	5.99E-01	6.00E-01	1.00E+00	6.01E-01	0.00E+00	1.00E+00	1.00E+00	glucosylglycerol-phosphate synthase [EC:2.4.1.213]
KO:K03694	12.67	18.84	0.72	8.26	0.00	1.95	6.24	6.60E-02	1.04E-02	9.87E-01	3.22E-01	1.00E+00	9.62E-01	6.29E-01	ATP-dependent Clp protease ATP-binding subunit ClpA
KO:K03695	9.67	17.48	8.93	4.62	7.80	3.97	3.97	2.38E-01	3.86E-03	3.59E-01	9.50E-01	5.95E-01	9.68E-01	9.68E-01	ATP-dependent Clp protease ATP-binding subunit ClpB
KO:K03696	1.31	0.52	21.11	1.22	26.82	3.18	0.17	7.58E-01	8.74E-01	4.14E-03	7.73E-01	1.86E-03	4.41E-01	8.98E-01	ATP-dependent Clp protease ATP-binding subunit ClpC
KO:K03698	0.00	0.00	7.64	0.05	0.66	0.00	0.00	1.00E+00	1.00E+00	4.00E-03	4.25E-01	1.52E-01	1.00E+00	1.00E+00	CMP-binding protein
KO:K03699	0.06	0.00	0.99	0.42	0.00	0.00	0.00	3.76E-01	1.00E+00	5.20E-02	1.47E-01	1.00E+00	1.00E+00	1.00E+00	putative hemolysin
KO:K03701	7.45	9.35	9.17	7.24	6.36	6.64	4.18	5.08E-01	1.66E-01	1.84E-01	5.60E-01	7.58E-01	7.00E-01	9.55E-01	excinuclease ABC subunit A
KO:K03702	8.46	7.18	4.37	5.37	7.31	0.43	3.55	8.14E-02	1.74E-01	7.67E-01	5.18E-01	1.61E-01	9.99E-01	8.81E-01	excinuclease ABC subunit B
KO:K03703	5.89	5.91	4.93	2.87	4.15	4.73	0.66	1.60E-01	1.57E-01	2.90E-01	7.90E-01	4.63E-01	3.28E-01	9.84E-01	excinuclease ABC subunit C
KO:K03704	9.74	17.99	2.44	11.16	1.16	3.26	6.63	2.49E-01	1.61E-02	9.67E-01	1.47E-01	9.89E-01	9.45E-01	6.78E-01	cold shock protein (beta-ribbon, CspA family)
KO:K03705	2.77	9.57	0.71	2.47	0.92	7.31	0.07	5.20E-01	4.10E-02	9.04E-01	5.96E-01	8.85E-01	8.07E-02	9.67E-01	heat-inducible transcriptional repressor
KO:K03707	0.18	4.44	0.00	0.78	0.00	0.00	0.00	6.00E-01	3.43E-02	1.00E+00	2.91E-01	1.00E+00	1.00E+00	1.00E+00	transcriptional activator TenA [EC:3.5.99.2]
KO:K03709	0.06	0.16	9.64	0.23	0.43	1.01	0.57	7.72E-01	6.89E-01	6.14E-03	6.42E-01	4.98E-01	2.49E-01	4.20E-01	DtxR family transcriptional regulator, Mn-dependent transcriptional
KO:K03710	1.56	3.54	0.01	4.45	0.00	0.00	0.46	3.84E-01	1.16E-01	9.10E-01	7.80E-02	1.00E+00	1.00E+00	7.52E-01	GntR family transcriptional regulator
KO:K03711	4.43	2.32	9.73	2.19	7.49	2.14	2.14	3.27E-01	7.97E-01	3.06E-02	8.19E-01	7.10E-02	8.27E-01	8.27E-01	Fur family transcriptional regulator, ferric uptake regulator
KO:K03712	0.00	0.21	1.23	0.01	0.00	0.00	0.00	1.00E+00	1.70E-01	3.13E-02	4.21E-01	1.00E+00	1.00E+00	1.00E+00	MarR family transcriptional regulator
KO:K03715	0.00	0.00	0.00	0.10	0.00	0.00	0.00	1.00E+00	1.00E+00	1.00E+00	1.43E-01	1.00E+00	1.00E+00	1.00E+00	1,2-diacylglycerol 3-beta-galactosyltransferase [EC:2.4.1.46]
KO:K03716	4.42	0.00	0.00	0.00	0.00	0.00	0.00	4.71E-02	1.00E+00	1.00E+00	1.00E+00	1.00E+00	1.00E+00	1.00E+00	spore photoproduct lyase [EC:4.1.99.-]
KO:K03717	0.00	0.05	3.98	0.13	0.00	0.00	0.28	5.66E-01	4.54E-01	1.70E-02	3.53E-01	1.00E+00	1.00E+00	2.52E-01	LysR family transcriptional regulator, transcriptional activator of
KO:K03718	1.08	4.21	0.00	0.54	0.00	0.10	0.53	3.34E-01	7.30E-02	1.00E+00	5.06E-01	1.00E+00	7.08E-01	5.09E-01	Lrp/AsnC family transcriptional regulator, regulator for asnA, asnC
KO:K03719	4.81	7.34	0.00	0.72	0.00	0.00	0.39	6.76E-02	2.83E-02	1.00E+00	7.14E-01	1.00E+00	1.00E+00	8.23E-01	Lrp/AsnC family transcriptional regulator, leucine-responsive
KO:K03721	0.64	0.00	0.98	0.01	0.00	0.00	0.00	9.31E-02	1.00E+00	6.23E-02	4.64E-01	1.00E+00	1.00E+00	1.00E+00	transcriptional regulator of aroF, aroG, tyrA and aromatic amino
KO:K03722	4.44	0.11	7.31	1.01	8.27	0.71	0.85	1.22E-01	9.42E-01	4.00E-02	7.47E-01	2.81E-02	8.22E-01	7.86E-01	ATP-dependent DNA helicase DinG [EC:3.6.1.-]
KO:K03723	5.52	10.64	4.64	2.83	6.81	3.95	1.10	3.51E-01	2.13E-02	5.87E-01	9.04E-01	1.53E-01	7.56E-01	9.88E-01	transcription-repair coupling factor (superfamily II helicase)
KO:K03724	1.23	2.20	2.74	0.80	6.50	1.53	0.19	6.28E-01	2.82E-01	1.80E-01	7.89E-01	2.27E-02	5.05E-01	9.39E-01	ATP-dependent helicase Lhr and Lhr-like helicase [EC:3.6.1.-]
KO:K03726	0.00	0.00	0.00	0.00	0.00	0.00	4.17	1.00E+00	1.00E+00	1.00E+00	1.00E+00	1.00E+00	1.00E+00	6.86E-03	helicase [EC:3.6.1.-]
KO:K03732	12.20	0.23	1.17	0.59	0.00	5.34	0.28	1.99E-02	8.30E-01	6.01E-01	7.47E-01	1.00E+00	1.04E-01	8.19E-01	ATP-dependent RNA helicase RhlB [EC:3.6.4.13]
KO:K03733	2.48	2.35	7.84	2.32	0.77	4.74	0.05	4.16E-01	4.52E-01	2.70E-02	4.61E-01	8.80E-01	1.01E-01	9.85E-01	integrase/recombinase XerC
KO:K03734	4.44	0.84	8.04	0.74	2.31	1.19	2.33	1.12E-01	8.50E-01	2.86E-02	8.68E-01	4.25E-01	7.71E-01	4.17E-01	thiamine biosynthesis lipoprotein
KO:K03735	0.19	0.00	0.00	0.14	0.00	0.00	0.00	1.15E-01	1.00E+00	1.00E+00	1.76E-01	1.00E+00	1.00E+00	1.00E+00	ethanolamine ammonia-lyase large subunit [EC:4.3.1.7]
KO:K03736	0.12	0.00	0.00	0.00	0.00	0.00	0.00	6.01E-02	1.00E+00	1.00E+00	1.00E+00	1.00E+00	1.00E+00	1.00E+00	ethanolamine ammonia-lyase small subunit [EC:4.3.1.7]
KO:K03737	0.02	0.00	9.74	0.01	0.22	0.00	0.00	4.43E-01	5.70E-01	1.07E-02	5.18E-01	2.27E-01	1.00E+00	1.00E+00	putative pyruvate-flavodoxin oxidoreductase [EC:1.2.7.-]
KO:K03738	0.00	0.00	0.00	0.62	0.00	28.15	0.00	4.03E-01	1.00E+00	1.00E+00	2.10E-01	1.00E+00	3.29E-03	1.00E+00	aldehyde:ferredoxin oxidoreductase [EC:1.2.7.5]
KO:K03741	0.23	2.89	5.62	0.31	0.13	3.85	0.03	8.38E-01	9.43E-02	2.47E-02	7.94E-01	8.77E-01	5.51E-02	9.36E-01	arsenate reductase [EC:1.2.4.1]
KO:K03742	1.08	2.41	15.23	1.24	5.95	1.02	0.22	8.16E-01	4.29E-01	2.86E-03	7.74E-01	5.91E-02	8.28E-01	9.53E-01	competence/damage-inducible protein CinA
KO:K03743	1.11	2.10	0.01	1.21	0.00	0.89	0.40	2.96E-01	1.05E-01	9.53E-01	2.59E-01	1.00E+00	3.94E-01	6.99E-01	
KO:K03744	2.27	0.20	11.09	0.93	0.01	0.23	0.01	1.49E-01	8.20E-01	6.14E-03	4.71E-01	9.37E-01	8.05E-01	9.24E-01	LemA protein
KO:K03745	1.90	0.75	0.00	0.21	0.00	0.00	0.00	6.93E-02	1.77E-01	1.00E+00	4.44E-01	1.00E+00	1.00E+00	1.00E+00	SlyX protein
KO:K03746	0.87	4.50	1.31	0.00	0.00	0.00	0.00	2.79E-01	4.29E-02	1.86E-01	8.55E-01	1.00E+00	1.00E+00	1.00E+00	DNA-binding protein H-NS
KO:K03747	0.01	0.00	0.00	0.94	0.00	0.00	14.38	6.03E-01	1.00E+00	1.00E+00	2.32E-01	1.00E+00	1.00E+00	1.09E-02	Smg protein
KO:K03748	0.02	0.08	0.00	0.15	0.00	0.00	0.00	3.32E-01	2.26E-01	1.00E+00	1.57E-01	1.00E+00	1.00E+00	1.00E+00	SanA protein
KO:K03749	5.26	0.00	0.02	0.11	0.00	3.26	0.00	3.14E-02	8.14E-01	7.84E-01	6.65E-01	1.00E+00	5.76E-02	1.00E+00	DedD protein
KO:K03750	3.94	13.24	0.89	3.49	3.59	0.44	0.99	4.56E-01	1.11E-02	9.48E-01	5.61E-01	5.36E-01	9.73E-01	9.41E-01	molybdopterin biosynthesis protein MoeA
KO:K03751	0.00	0.00	0.00	1.53	0.00	0.00	0.00	1.00E+00	1.00E+00	1.00E+00	1.43E-01	1.00E+00	1.00E+00	1.00E+00	molybdopterin biosynthesis protein MoeB
KO:K03752	1.11	5.73	0.03	1.47	1.04	0.00	0.75	5.57E-01	3.20E-02	9.64E-01	4.13E-01	5.89E-01	1.00E+00	7.09E-01	molybdopterin-guanine dinucleotide biosynthesis protein A
KO:K03753	0.67	2.95	0.00	0.88	0.00	2.66	0.00	4.57E-01	7.61E-02	1.00E+00	3.64E-01	1.00E+00	8.79E-02	1.00E+00	molybdopterin-guanine dinucleotide biosynthesis protein B
KO:K03759	0.06	0.00	0.00	0.44	0.00	0.00	0.00	2.24E-01	1.00E+00	1.00E+00	1.04E-01	1.00E+00	1.00E+00	1.00E+00	arginine:agmatine antiporter
KO:K03760	0.38	1.25	0.05	0.44	0.00	0.00	0.08	3.49E-01	1.18E-01	6.58E-01	3.21E-01	1.00E+00	1.00E+00	6.03E-01	phosphoethanolamine transferase
KO:K03762	0.40	0.00	0.00	0.18	1.73	0.00	0.00	1.97E-01	1.00E+00	1.00E+00	2.61E-01	5.21E-02	1.00E+00	1.00E+00	MFS transporter, MHS family, proline/betaine transporter
KO:K03767	3.96	1.59	0.08	0.52	0.00	2.15	0.73	9.00E-02	3.13E-01	8.29E-01	6.41E-01	1.00E+00	2.19E-01	5.64E-01	peptidyl-prolyl cis-trans isomerase A (cyclophilin A) [EC:5.2.1.8]

KO:K03768	4.52	0.16	3.12	1.38	17.93	0.00	4.73	1.65E-01	9.53E-01	3.51E-01	7.64E-01	4.29E-03	1.00E+00	1.51E-01	peptidyl-prolyl cis-trans isomerase B (cyclophilin B) [EC:5.2.1.8]
KO:K03769	1.50	13.02	0.01	1.82	0.28	0.00	0.03	5.40E-01	6.86E-03	9.69E-01	4.37E-01	8.95E-01	1.00E+00	9.63E-01	peptidyl-prolyl cis-trans isomerase C [EC:5.2.1.8]
KO:K03770	11.92	11.06	0.61	4.91	0.00	7.75	3.23	4.47E-02	5.79E-02	9.81E-01	5.85E-01	1.00E+00	1.92E-01	8.44E-01	peptidyl-prolyl cis-trans isomerase D [EC:5.2.1.8]
KO:K03771	11.28	6.23	4.90	2.17	0.10	5.79	1.38	2.87E-02	1.92E-01	3.54E-01	8.63E-01	9.92E-01	2.35E-01	9.29E-01	peptidyl-prolyl cis-trans isomerase SurA [EC:5.2.1.8]
KO:K03772	2.31	0.14	4.44	0.69	0.00	0.00	0.03	1.54E-01	6.10E-01	6.31E-02	3.92E-01	1.00E+00	1.00E+00	6.78E-01	FKBP-type peptidyl-prolyl cis-trans isomerase FkpA [EC:5.2.1.8]
KO:K03773	7.36	0.05	8.52	1.09	0.58	0.00	3.52	5.90E-02	9.06E-01	4.59E-02	6.66E-01	7.92E-01	1.00E+00	2.11E-01	FKBP-type peptidyl-prolyl cis-trans isomerase FklB [EC:5.2.1.8]
KO:K03774	12.86	0.44	0.00	0.08	0.00	0.08	1.39	1.09E-02	6.73E-01	1.00E+00	8.10E-01	1.00E+00	8.13E-01	3.66E-01	FKBP-type peptidyl-prolyl cis-trans isomerase SlpA [EC:5.2.1.8]
KO:K03775	3.78	0.18	2.08	1.43	0.00	0.73	3.19	9.40E-02	8.72E-01	2.52E-01	4.14E-01	1.00E+00	6.73E-01	1.27E-01	FKBP-type peptidyl-prolyl cis-trans isomerase SlyD [EC:5.2.1.8]
KO:K03776	0.55	0.21	0.00	0.16	0.00	0.00	0.17	1.42E-01	3.09E-01	1.00E+00	3.58E-01	1.00E+00	1.00E+00	3.51E-01	aerotaxis receptor
KO:K03777	0.40	0.13	0.00	1.46	0.00	0.00	0.00	2.48E-01	4.55E-01	1.00E+00	6.69E-02	1.00E+00	1.00E+00	1.00E+00	D-lactate dehydrogenase [EC:1.1.1.28]
KO:K03778	0.21	0.02	0.90	0.14	0.00	0.26	0.78	3.88E-01	7.39E-01	8.01E-02	4.95E-01	1.00E+00	3.29E-01	9.94E-02	D-lactate dehydrogenase [EC:1.1.1.28]
KO:K03779	0.00	7.22	0.00	0.00	0.00	0.00	0.00	1.00E+00	2.63E-02	1.00E+00	1.00E+00	1.00E+00	1.00E+00	1.00E+00	L(+)-tartrate dehydratase alpha subunit [EC:4.2.1.32]
KO:K03780	0.00	4.89	0.00	0.31	0.00	0.00	0.00	1.00E+00	4.20E-02	1.00E+00	3.66E-01	1.00E+00	1.00E+00	1.00E+00	L(+)-tartrate dehydratase beta subunit [EC:4.2.1.32]
KO:K03781	0.26	0.00	0.30	0.01	0.00	0.00	0.00	9.81E-02	1.00E+00	8.83E-02	4.21E-01	1.00E+00	1.00E+00	1.00E+00	catalase [EC:1.11.1.6]
KO:K03782	10.17	12.74	14.10	8.83	2.03	8.16	1.02	2.44E-01	9.70E-02	6.30E-02	4.12E-01	9.81E-01	5.13E-01	9.91E-01	catalase/peroxidase [EC:1.11.1.6 1.11.1.7]
KO:K03783	0.50	0.46	6.02	0.68	0.69	0.98	0.02	5.92E-01	6.14E-01	1.60E-02	4.89E-01	4.79E-01	3.54E-01	8.83E-01	purine-nucleoside phosphorylase [EC:2.4.2.1]
KO:K03784	0.03	9.95	0.03	0.02	0.00	0.00	0.00	8.16E-01	1.86E-03	8.12E-01	8.25E-01	1.00E+00	1.00E+00	1.00E+00	purine-nucleoside phosphorylase [EC:2.4.2.1]
KO:K03785	0.00	0.00	4.37	0.03	0.00	0.00	1.03	1.00E+00	1.00E+00	8.43E-03	4.37E-01	1.00E+00	1.00E+00	8.10E-02	3-dehydroquinate dehydratase I [EC:4.2.1.1]
KO:K03786	4.59	1.85	2.01	5.58	4.30	2.56	0.22	1.81E-01	7.87E-01	7.49E-01	9.94E-02	2.11E-01	6.05E-01	9.82E-01	3-dehydroquinate dehydratase II [EC:4.2.1.1]
KO:K03787	1.42	7.93	4.32	2.03	3.03	0.81	0.95	8.39E-01	3.09E-02	1.79E-01	7.01E-01	4.06E-01	9.25E-01	9.13E-01	5'-nucleotidase [EC:3.1.3.5]
KO:K03789	0.79	0.14	4.08	0.38	2.44	0.06	1.21	4.21E-01	8.37E-01	3.06E-02	6.87E-01	7.99E-02	8.99E-01	2.44E-01	ribosomal-protein-alanine N-acetyltransferase [EC:2.3.1.128]
KO:K03790	0.11	8.50	0.11	0.04	0.00	0.00	0.00	7.98E-01	2.00E-03	8.04E-01	8.64E-01	1.00E+00	1.00E+00	1.00E+00	ribosomal-protein-alanine N-acetyltransferase [EC:2.3.1.128]
KO:K03791	0.00	0.00	0.00	0.10	0.00	0.00	0.00	1.00E+00	1.00E+00	1.00E+00	1.43E-01	1.00E+00	1.00E+00	1.00E+00	putative chitinase
KO:K03793	2.58	0.05	2.90	0.37	0.00	0.00	1.86	1.04E-01	7.78E-01	8.67E-02	5.86E-01	1.00E+00	1.00E+00	1.62E-01	pteridine reductase [EC:1.5.1.33]
KO:K03795	0.54	1.33	0.00	0.80	19.94	0.00	0.03	6.88E-01	3.63E-01	1.00E+00	5.64E-01	1.00E-03	1.00E+00	8.86E-01	sirohydrochlorin cobaltochelatase [EC:4.99.1.3]
KO:K03796	4.79	0.03	4.01	0.82	0.00	0.00	0.87	6.24E-02	8.02E-01	8.06E-02	4.87E-01	1.00E+00	1.00E+00	4.72E-01	Bax protein
KO:K03797	10.34	9.40	15.68	7.50	11.24	2.02	3.80	2.11E-01	3.34E-01	2.17E-02	6.85E-01	1.33E-01	9.95E-01	9.70E-01	carboxyl-terminal processing protease [EC:3.4.21.12]
KO:K03798	8.98	11.46	10.73	9.74	20.38	5.91	5.04	8.22E-01	3.66E-01	5.13E-01	7.13E-01	2.86E-03	9.71E-01	9.84E-01	cell division protease FtsH [EC:3.4.24.-]
KO:K03799	9.32	0.76	0.04	2.12	0.00	10.32	0.35	2.69E-02	8.20E-01	9.57E-01	4.74E-01	1.00E+00	2.13E-02	8.96E-01	heat shock protein HtpX [EC:3.4.24.-]
KO:K03800	0.00	0.00	3.18	0.01	11.05	0.56	0.14	7.75E-01	7.76E-01	3.44E-02	7.73E-01	3.14E-03	2.70E-01	5.56E-01	lipoate-protein ligase A [EC:2.7.7.63]
KO:K03801	3.66	4.26	0.53	1.86	2.11	4.39	0.53	1.88E-01	1.19E-01	9.52E-01	6.79E-01	5.85E-01	1.09E-01	9.52E-01	lipoyl(octanoyl) transferase [EC:2.3.1.181]
KO:K03802	1.15	0.39	0.00	0.21	0.22	0.00	0.00	1.24E-01	2.95E-01	1.00E+00	3.99E-01	3.94E-01	1.00E+00	1.00E+00	cyanophycin synthetase [EC:6.-.-.-]
KO:K03803	1.22	0.00	0.01	0.12	0.00	7.78	1.01	1.75E-01	7.85E-01	7.51E-01	5.87E-01	1.00E+00	1.06E-02	2.12E-01	sigma-E factor negative regulatory protein RseC
KO:K03806	2.94	0.03	0.06	0.44	0.00	0.43	2.68	6.76E-02	8.22E-01	7.99E-01	5.34E-01	1.00E+00	5.40E-01	7.60E-02	AmpD protein
KO:K03807	4.90	0.00	0.00	0.00	0.00	0.00	0.00	2.71E-02	1.00E+00	1.00E+00	6.04E-01	1.00E+00	1.00E+00	1.00E+00	AmpE protein
KO:K03808	0.03	0.00	4.58	0.10	0.00	0.00	0.00	4.64E-01	1.00E+00	4.57E-03	3.31E-01	1.00E+00	1.00E+00	1.00E+00	paraquat-inducible protein A
KO:K03809	7.58	0.26	1.89	1.67	0.00	0.24	0.09	2.77E-02	8.15E-01	2.95E-01	3.42E-01	1.00E+00	8.22E-01	8.67E-01	Trp repressor binding protein
KO:K03811	8.56	0.33	0.07	0.09	0.00	0.00	0.60	1.66E-02	6.31E-01	8.01E-01	7.84E-01	1.00E+00	1.00E+00	4.95E-01	nicotinamide mononucleotide transporter
KO:K03812	5.29	0.19	5.89	0.03	0.00	0.00	0.01	4.27E-02	6.51E-01	3.70E-02	7.77E-01	1.00E+00	1.00E+00	8.14E-01	ribosome modulation factor
KO:K03814	0.01	7.66	0.00	0.08	0.00	0.00	0.02	8.62E-01	5.00E-03	1.00E+00	7.49E-01	1.00E+00	1.00E+00	8.43E-01	monofunctional biosynthetic peptidoglycan transglycosylase
KO:K03815	0.02	0.00	0.53	0.00	0.00	0.00	0.00	2.36E-01	1.00E+00	2.71E-02	1.00E+00	1.00E+00	1.00E+00	1.00E+00	xanthosine phosphorylase [EC:2.4.2.-]
KO:K03818	0.06	0.13	0.14	0.13	0.14	0.00	2.62	5.80E-01	4.83E-01	4.75E-01	4.82E-01	4.75E-01	1.00E+00	3.13E-02	putative colanic acid biosynthesis acetyltransferase WcaF
KO:K03820	3.51	2.21	3.36	2.43	8.66	1.78	0.05	2.41E-01	5.90E-01	2.72E-01	5.21E-01	2.17E-02	7.25E-01	9.91E-01	apolipoprotein N-acyltransferase [EC:2.3.1.-]
KO:K03821	0.99	16.12	0.04	2.77	0.05	14.53	0.05	8.86E-01	1.20E-02	9.68E-01	6.07E-01	9.68E-01	1.80E-02	9.68E-01	polyhydroxyalkanoate synthase [EC:2.3.1.-]
KO:K03823	0.26	4.94	0.00	1.03	0.00	0.00	0.23	6.05E-01	3.84E-02	1.00E+00	2.87E-01	1.00E+00	1.00E+00	6.21E-01	phosphinothricin acetyltransferase [EC:2.3.1.183]
KO:K03824	2.57	0.18	0.00	0.01	0.00	0.00	0.00	4.14E-02	2.43E-01	1.00E+00	5.42E-01	1.00E+00	1.00E+00	1.00E+00	putative acetyltransferase [EC:2.3.1.-]
KO:K03826	0.00	0.10	0.37	0.01	0.00	0.00	0.00	1.00E+00	1.62E-01	3.74E-02	4.08E-01	1.00E+00	1.00E+00	1.00E+00	putative acetyltransferase [EC:2.3.1.-]
KO:K03828	0.37	0.13	0.00	0.13	0.00	5.26	0.00	2.95E-01	4.65E-01	1.00E+00	4.67E-01	1.00E+00	1.04E-02	1.00E+00	putative acetyltransferase [EC:2.3.1.-]
KO:K03829	0.23	1.50	0.00	1.43	0.00	0.00	0.00	4.32E-01	1.28E-01	1.00E+00	1.34E-01	1.00E+00	1.00E+00	1.00E+00	putative acetyltransferase [EC:2.3.1.-]
KO:K03830	0.00	0.05	0.00	0.00	0.00	0.00	0.66	1.00E+00	1.98E-01	1.00E+00	1.00E+00	1.00E+00	1.00E+00	3.01E-02	putative acetyltransferase [EC:2.3.1.-]
KO:K03831	0.00	0.01	3.56	0.44	3.56	0.00	0.01	1.00E+00	6.19E-01	4.81E-02	2.81E-01	4.81E-02	1.00E+00	6.44E-01	molybdopterin biosynthesis protein Mog
KO:K03832	13.02	1.21	0.48	4.14	0.00	2.60	1.03	2.21E-02	7.88E-01	8.90E-01	2.69E-01	1.00E+00	5.10E-01	8.20E-01	periplasmic protein TonB
KO:K03833	1.46	0.01	0.00	0.21	0.00	0.00	1.78	1.29E-01	6.10E-01	1.00E+00	3.97E-01	1.00E+00	1.00E+00	1.00E-01	selenocysteine-specific elongation factor
KO:K03834	0.00	0.00	0.00	0.00	0.00	5.00	0.00	1.00E+00	1.00E+00	1.00E+00	1.00E+00	1.00E+00	2.71E-03	1.00E+00	tyrosine-specific transport protein
KO:K03839	0.95	0.00	0.00	0.01	0.00	0.00	0.00	5.97E-02	1.00E+00	1.00E+00	3.12E-01	1.00E+00	1.00E+00	1.00E+00	flavodoxin I
KO:K03840	0.53	0.04	0.00	0.03	0.00	0.00	0.00	7.93E-02	3.40E-01	1.00E+00	3.55E-01	1.00E+00	1.00E+00	1.00E+00	flavodoxin II
KO:K03841	2.05	0.50	0.61	4.31	2.70	0.21	0.01	2.74E-01	7.52E-01	7.14E-01	8.06E-02	1.80E-01	8.50E-01	9.40E-01	fructose-1,6-bisphosphatase I [EC:3.1.3.11]
KO:K03851	0.34	25.86	0.00	0.09	0.00	0.00	0.00	7.23E-01	3.43E-03	1.00E+00	7.83E-01	1.00E+00	1.00E+00	1.00E+00	taurine-pyruvate aminotransferase [EC:2.6.1.77]
KO:K03852	0.74	38.90	0.00	2.34	0.00	0.00	0.00	8.47E-01	1.00E-03	1.00E+00	5.88E-01	1.00E+00	1.00E+00	1.00E+00	sulfoacetaldehyde acetyltransferase [EC:2.3.3.15]
KO:K03855	0.00	0.00	0.00	0.71	0.00	0.00	0.00	1.00E+00	1.00E+00	1.00E+00	1.43E-01	1.00E+00	1.00E+00	1.00E+00	ferredoxin like protein
KO:K03856	0.20	0.15	17.68	1.27	0.57	0.84	1.16	7.91E-01	8.05E-01	2.43E-03	4.17E-01	6.57E-01	5.48E-01	4.45E-01	3-deoxy-7-phosphoheptulonate synthase [EC:2.5.1.54]
KO:K03862	1.68	9.14	0.00	0.02	0.00	0.00	0.00	2.35E-01	2.53E-02	1.00E+00	7.58E-01	1.00E+00	1.00E+00	1.00E+00	vanillate monooxygenase [EC:1.14.13.82]
KO:K03863	0.19	1.01	0.00	0.19	0.00	0.00	0.00	3.40E-01	7.34E-02	1.00E+00	3.38E-01	1.00E+00	1.00E+00	1.00E+00	vanillate monooxygenase [EC:1.14.13.82]
KO:K03867	0.00	0.00	0.00	0.00	24.38	0.00	0.00	1.00E+00	1.00E+00	1.00E+00	1.00E+00	0.00E+00	1.00E+00	1.00E+00	UDP-glucose:tetrahydrobiopterin glucosyltransferase [EC:2.4.1.-]
KO:K03878	0.00	0.00	0.00	1.94	0.00	0.00	0.00	1.00E+00	1.00E+00	1.00E+00	1.43E-01	1.00E+00	1.00E+00	1.00E+00	NADH dehydrogenase I subunit 1 [EC:1.6.5.3]
KO:K03885	0.01	0.25	0.11	0.84	19.39	0.00	0.64	8.56E-01	6.61E-01	7.42E-01	4.07E-01	1.71E-03	1.00E+00	4.83E-01	NADH dehydrogenase [EC:1.6.99.3]
KO:K03886	0.00	0.00	1.54	0.09	6.16	0.00	0.00	1.00E+00	1.00E+00	1.12E-01	3.16E-01	2.07E-02	1.00E+00	1.00E+00	menaquinol-cytochrome c reductase iron-sulfur subunit [EC:1.1.2.-]
KO:K03889	0.00	0.00	0.00	0.41	0.00	0.00	0.00	1.00E+00	1.00E+00	1.00E+00	1.43E-01	1.00E+00	1.00E+00	1.00E+00	ubiquinol-cytochrome c reductase cytochrome c subunit
KO:K03892	5.13	1.59	7.19	1.31	2.64	0.00	5.68	1.52E-01	7.22E-01	7.31E-02	7.80E-01	4.72E-01	1.00E+00	1.22E-01	ArsR family transcriptional regulator
KO:K03896	0.00	0.00	0.00	0.10	0.00	0.00	0.00	1.00E+00	1.00E+00	1.00E+00	1.43E-01	1.00E+00	1.00E+00	1.00E+00	acetyl CoA:N6-hydroxylysine acetyl transferase [EC:2.3.1.12]
KO:K03918	0.47	0.00	0.00	1.04	0.00	0.00	0.00	1.71E-01	1.00E+00	1.00E+00	1.47E-01	1.00E+00	1.00E+00	1.00E+00	L-lysine 6-transaminase [EC:2.6.1.36]
KO:K03919	0.02	2.23	0.00	0.00	0.00	0.00	0.00	6.50E-01	8.86E-03	1.00E+00	1.00E+00	1.00E+00	1.00E+00	1.00E+00	alkylated DNA repair protein [EC:1.14.11.-]
KO:K03921	0.00	0.11	0.00	0.00	0.00	0.00	0.00	1.00E+00	1.05E-01	1.00E+00	1.00E+00	1.00E+00	1.00E+00	1.00E+00	acyl-[acyl-carrier-protein] desaturase [EC:1.14.19.2]
KO:K03923	0.00	0.00	0.63	0.00	0.00	0.00	0.00	1.00E+00	1.00E+00	1.50E-02	1.00E+00	1.00E+00	1.00E+00	1.00E+00	modulator of drug activity B
KO:K03924	6.56	1.55	22.15	2.13	3.16	1.97	5.14	2.57E-01	9.07E-01	5.14E-03	8.58E-01	7.35E-01	8.73E-01	4.32E-01	MoxR-like ATPase [EC:3.6.3.-]
KO:K03925	4.30	4.34	7.69	4.58	1.92	2.75	0.91	3.96E-01	3.88E-01	9.87E-02	3.50E-01	8.26E-01	6.85E-01	9.19E-01	MraZ protein
KO:K03926	0.01	0.00	5.00	0.05	8.44	0.00	0.02	7.81E-01	1.00E+00	2.31E-02	6.27E-01	1.00E-02	1.00E+00	7.24E-01	periplasmic divalent cation tolerance protein
KO:K03927	13.15	0.14	0.11	0.18	0.00	0.00	0.00	1.96E-02	6.30E-01	6.49E-01	6.09E-01	1.00E+00	1.00E+00	1.00E+00	carboxylesterase type B [EC:3.1.1.1]
KO:K03928	0.00	0.00	0.00	0.00	3.03	0.00	0.00	1.00E+00	1.00E+00	1.00E+00	2.50E-01	7.29E-03	1.00E+00	1.00E+00	carboxylesterase [EC:3.1.1.1]
KO:K03932	0.03	0.02	0.00	0.18	0.00	0.00	0.00	2.92E-01	3.14E-01	1.00E+00	1.32E-01	1.00E+00	1.00E+00	1.00E+00	polyhydroxybutyrate depolymerase
KO:K03934	0.00	0.00	0.00	1.33	0.00	0.00	0.00	1.00E+00	1.00E+00	1.00E+00	1.43E-01	1.00E+00	1.00E+00	1.00E+00	NADH dehydrogenase (ubiquinone) Fe-S protein 1 [EC:1.6.5.3 1.6.99.3]
KO:K03936	0.00	0.11	0.00	0.00	0.00	0.00	0.00	1.00E+00	1.03E-01	1.00E+00	1.00E+00	1.00E+00	1.00E+00	1.00E+00	NADH dehydrogenase (ubiquinone) Fe-S protein 3 [EC:1.6.5.3 1.6.99.3]
KO:K03941	0.00	0.00	0.00	2.45	0.00	0.00	0.00	1.00E+00	1.00E+00	1.00E+00	7.89E-02	1.00E+00	1.00E+00	1.00E+00	NADH dehydrogenase (ubiquinone) Fe-S protein 8 [EC:1.6.5.3 1.6.99.3]
KO:K03942	0.00	0.00	0.00	2.14	0.00	0.00	0.00	1.00E+00	1.00E+00	1.00E+00	7.70E-02	1.00E+00	1.00E+00	1.00E+00	NADH dehydrogenase (ubiquinone) flavoprotein 1 [EC:1.6.5.3 1.6.99.3]
KO:K03969	3.31	0.01	0.69	0.85	7.02	2.00	0.09	1.26E-01	8.65E-01	5.88E-01	5.34E-01	3.56E-02	2.51E-01	8.25E-01	phage shock protein A
KO:K03970	3.15	0.09	0.06	0.10	0.00	0.00	0.00	4.20E-02	4.55E-01	5.07E-01	4.44E-01	1.00E+00	1.00E+00	1.00E+00	phage shock protein B
KO:K03972	1.71	0.00	1.97	0.00	0.00	0.00	0.16	5.66E-02	1.00E+00	4.33E-02	1.00E+00	1.00E+00	1.00E+00	4.35E-01	phage shock protein E
KO:K03973	5.34	0.01	0.05	0.08	0.00	0.00	0.00	3.76E-02	6.39E-01	5.28E-01	4.81E-01	1.00E+00	1.00E+00	1.00E+00	phage shock protein C
KO:K03974	2.17	0.01	0.70	0.33	0.00	0.00	0.00	6.34E-02	7.23E-01	1.92E-01	3.30E-01	1.00E+00	1.00E+00	1.00E+00	psp operon transcriptional activator
KO:K03975	1.35	0.02	0.66	0.03	0.00	0.00	0.00	8.07E-02	4.77E-01	1.62E-01	4.43E-01	1.00E+00	1.00E+00	1.00E+00	membrane-associated protein
KO:K03976	0.00	0.11	0.00	0.00	0.00	0.00	0.00	1.00E+00	1.02E-01	1.00E+00	1.00E+00	1.00E+00	1.00E+00	1.00E+00	putative transcription regulator
KO:K03977	9.07	11.08	5.20	3.23	7.80	3.18	0.79	1.03E-01	4.74E-02	6.10E-01	8.87E-01	1.85E-01	8.90E-01	9.89E-01	GTP-binding protein
KO:K03978	6.22	4.70	0.22	1.52	0.00	0.18	1.33	3.83E-02	7.40E-02	9.54E-01	6.12E-01	1.00E+00	9.58E-01	6.81E-01	GTP-binding protein
KO:K03979	8.54	10.86	5.62	6.19	6.20	0.54	2.26	1.37E-01	4.77E-02	6.16E-01	4.85E-01	4.82E-01	9.96E-01	9.66E-01	GTP-binding protein
KO:K03980	5.62	4.48	3.11	3.13	6.37	1.03	2.42	1.94E-01	3.51E-01	6.53E-01	6.48E-01	1.34E-01	9.40E-01	7.85E-01	virulence factor
KO:K03981	10.55	0.23	0.14	0.49	0.00	5.56	1.76	1.64E-02	8.31E-01	8.59E-01	7.82E-01	1.00E+00	8.16E-02	4.30E-01	thiol:disulfide interchange protein DsbC [EC:5.3.4.1]

KO:K04014	0.00	0.00	0.63	0.00	0.00	0.00	0.00	1.00E+00	1.00E+00	1.70E-02	1.00E+00	1.00E+00	1.00E+00	1.00E+00	formate-dependent nitrite reductase, Fe-S protein
KO:K04019	0.00	0.00	0.00	2.04	0.00	0.00	0.00	1.00E+00	1.00E+00	1.00E+00	1.43E-01	1.00E+00	1.00E+00	1.00E+00	ethanolamine utilization protein EutA
KO:K04021	0.00	0.00	30.82	0.09	1.00	0.00	0.00	6.01E-01	1.00E+00	4.29E-04	5.23E-01	2.50E-01	1.00E+00	1.00E+00	aldehyde dehydrogenase
KO:K04027	0.00	0.00	12.91	0.17	0.00	0.00	0.00	1.00E+00	1.00E+00	7.14E-03	3.15E-01	1.00E+00	1.00E+00	1.00E+00	ethanolamine utilization protein EutM
KO:K04028	0.00	0.00	17.70	0.08	4.74	0.00	0.00	6.92E-01	6.97E-01	1.14E-03	6.25E-01	3.14E-02	1.00E+00	1.00E+00	ethanolamine utilization protein EutN
KO:K04033	0.00	0.11	0.00	0.00	0.00	0.00	0.00	1.00E+00	1.05E-01	1.00E+00	1.00E+00	1.00E+00	1.00E+00	1.00E+00	AraC family transcriptional regulator, ethanolamine operon
KO:K04034	0.06	0.48	0.00	0.05	0.00	0.00	0.08	3.52E-01	1.20E-01	1.00E+00	3.77E-01	1.00E+00	1.00E+00	3.13E-01	anaerobic magnesium-protoporphyrin IX monomethyl ester cyclase
KO:K04035	0.61	2.67	0.00	0.63	24.94	0.00	0.19	7.07E-01	2.36E-01	1.00E+00	7.01E-01	1.57E-03	1.00E+00	8.32E-01	magnesium-protoporphyrin IX monomethyl ester (oxidative) cyclase
KO:K04036	0.00	0.00	0.00	0.10	0.00	0.00	0.00	1.00E+00	1.00E+00	1.00E+00	1.43E-01	1.00E+00	1.00E+00	1.00E+00	divinyl protochlorophyllide a 8-vinyl-reductase [EC:1.-.-.-]
KO:K04037	0.73	2.64	0.00	0.76	25.54	0.00	0.00	6.69E-01	2.37E-01	1.00E+00	6.58E-01	1.14E-03	1.00E+00	1.00E+00	light-independent protochlorophyllide reductase subunit L
KO:K04038	0.87	3.61	0.00	0.83	15.75	0.00	0.00	6.16E-01	1.33E-01	1.00E+00	6.31E-01	5.00E-03	1.00E+00	1.00E+00	light-independent protochlorophyllide reductase subunit N
KO:K04039	0.91	3.47	0.00	0.72	17.28	0.00	0.00	6.01E-01	1.43E-01	1.00E+00	6.72E-01	3.43E-03	1.00E+00	1.00E+00	light-independent protochlorophyllide reductase subunit B
KO:K04040	0.47	4.87	0.00	0.59	15.56	0.00	0.00	7.34E-01	8.24E-02	1.00E+00	6.95E-01	6.43E-03	1.00E+00	1.00E+00	chlorophyll synthase [EC:2.5.1.62]
KO:K04042	7.40	5.07	0.83	3.72	5.83	2.49	3.66	9.54E-02	3.26E-01	9.72E-01	6.33E-01	2.11E-01	8.53E-01	6.47E-01	bifunctional protein GlmU [EC:2.7.7.23 2.3.1.157]
KO:K04043	11.75	14.97	11.85	10.93	13.03	3.09	8.66	4.43E-01	4.57E-02	4.20E-01	6.45E-01	1.98E-01	9.98E-01	9.25E-01	molecular chaperone DnaK
KO:K04044	8.94	0.07	0.79	0.78	0.00	0.00	1.73	2.86E-02	7.83E-01	5.69E-01	5.70E-01	1.00E+00	1.00E+00	3.15E-01	molecular chaperone HscA
KO:K04045	0.01	0.00	0.34	0.00	0.00	0.00	0.15	3.20E-01	1.00E+00	4.29E-02	1.00E+00	1.00E+00	1.00E+00	1.09E-01	molecular chaperone HscC
KO:K04046	0.48	0.16	0.00	0.00	0.04	0.00	3.98	2.34E-01	3.38E-01	1.00E+00	6.14E-01	4.67E-01	1.00E+00	4.59E-02	hypothetical chaperone protein
KO:K04047	0.32	0.09	1.13	0.84	1.13	0.00	0.04	4.77E-01	7.87E-01	1.07E-01	1.62E-01	1.07E-01	1.00E+00	8.27E-01	starvation-inducible DNA-binding protein
KO:K04056	0.12	0.00	0.00	0.00	0.00	0.00	0.00	6.40E-02	1.00E+00	1.00E+00	1.00E+00	1.00E+00	1.00E+00	1.00E+00	type III secretion protein SctO
KO:K04058	0.12	0.00	0.00	0.00	0.00	0.00	0.00	6.29E-02	1.00E+00	1.00E+00	1.00E+00	1.00E+00	1.00E+00	1.00E+00	type III secretion protein SctW
KO:K04061	3.31	0.00	0.04	0.11	0.00	0.29	0.02	4.41E-02	7.69E-01	6.15E-01	4.79E-01	1.00E+00	3.36E-01	7.15E-01	flagellar biosynthesis protein
KO:K04063	0.15	0.00	0.28	0.07	0.00	0.00	0.00	1.69E-01	1.00E+00	1.15E-01	2.41E-01	1.00E+00	1.00E+00	1.00E+00	osmotically inducible protein OsmC
KO:K04065	0.00	0.00	0.00	0.10	0.00	0.00	0.00	1.00E+00	1.00E+00	1.00E+00	1.43E-01	1.00E+00	1.00E+00	1.00E+00	hyperosmotically inducible periplasmic protein
KO:K04066	2.96	3.65	5.35	2.92	6.78	2.79	1.06	5.93E-01	3.80E-01	9.89E-02	6.09E-01	4.46E-02	6.49E-01	9.58E-01	primosomal protein N' (replication factor Y) (superfamily II
KO:K04069	0.04	0.00	0.29	0.28	0.00	0.00	1.15	5.13E-01	1.00E+00	2.04E-01	2.09E-01	1.00E+00	1.00E+00	5.04E-02	pyruvate formate lyase activating enzyme [EC:1.97.1.4]
KO:K04072	1.33	0.00	0.00	0.02	0.00	0.00	0.00	6.16E-02	1.00E+00	1.00E+00	2.03E-01	1.00E+00	1.00E+00	1.00E+00	acetaldehyde dehydrogenase / alcohol dehydrogenase [EC:1.2.1.1
KO:K04073	2.01	0.00	0.00	0.90	0.00	0.00	0.90	1.13E-01	1.00E+00	1.00E+00	2.27E-01	1.00E+00	1.00E+00	2.24E-01	acetaldehyde dehydrogenase [EC:1.2.1.1]
KO:K04074	0.00	0.00	0.00	1.12	0.00	0.00	0.00	1.00E+00	1.00E+00	1.00E+00	5.97E-02	1.00E+00	1.00E+00	1.00E+00	cell division initiation protein
KO:K04075	3.48	2.07	0.91	2.10	1.28	9.10	0.40	1.63E-01	5.43E-01	8.87E-01	5.34E-01	8.12E-01	9.29E-03	9.59E-01	tRNA(Ile)-lysidine synthase [EC:6.3.4.-]
KO:K04077	10.99	13.64	19.23	13.32	11.47	4.44	6.50	7.36E-01	2.32E-01	1.13E-02	2.80E-01	6.49E-01	9.95E-01	9.79E-01	chaperonin GroEL
KO:K04078	7.93	5.99	14.46	7.14	5.06	3.46	2.18	2.74E-01	5.89E-01	2.43E-02	3.82E-01	7.40E-01	8.98E-01	9.59E-01	chaperonin GroES
KO:K04079	13.52	2.21	7.18	5.10	7.97	3.02	6.67	3.61E-02	9.37E-01	3.39E-01	6.65E-01	2.48E-01	8.92E-01	4.11E-01	molecular chaperone HtpG
KO:K04080	3.34	19.68	2.77	12.59	0.00	0.79	0.15	7.73E-01	8.86E-03	8.37E-01	5.07E-02	1.00E+00	9.62E-01	9.79E-01	molecular chaperone IbpA
KO:K04082	1.47	0.08	0.02	0.34	0.00	0.00	6.17	1.83E-01	6.77E-01	7.24E-01	4.91E-01	1.00E+00	1.00E+00	2.36E-02	molecular chaperone HscB
KO:K04083	3.98	11.57	0.11	0.91	6.00	0.03	0.02	2.04E-01	1.19E-02	9.62E-01	8.58E-01	7.91E-02	9.75E-01	9.85E-01	molecular chaperone Hsp33
KO:K04084	8.95	0.06	0.65	1.63	0.00	0.15	7.78	3.87E-02	8.89E-01	7.58E-01	4.99E-01	1.00E+00	8.66E-01	4.87E-02	thiol:disulfide interchange protein DsbD [EC:1.8.1.8]
KO:K04085	3.03	3.26	0.12	0.41	0.00	0.87	2.67	1.33E-01	1.18E-01	8.90E-01	8.01E-01	1.00E+00	6.24E-01	1.69E-01	tRNA 2-thiouridine synthesizing protein A [EC:2.8.1.-]
KO:K04087	8.68	11.78	1.97	8.63	0.28	1.11	3.40	1.49E-01	5.76E-02	9.33E-01	1.51E-01	9.85E-01	9.64E-01	8.37E-01	membrane protease subunit HflC [EC:3.4.-.-]
KO:K04088	12.54	15.85	2.75	8.37	0.00	0.12	4.64	6.23E-02	2.41E-02	9.10E-01	2.46E-01	1.00E+00	9.91E-01	7.44E-01	membrane protease subunit HflK [EC:3.4.-.-]
KO:K04090	6.05	33.25	0.00	3.24	0.00	0.00	0.00	2.17E-01	1.00E-03	1.00E+00	5.99E-01	1.00E+00	1.00E+00	1.00E+00	indolepyruvate ferredoxin oxidoreductase [EC:1.2.7.8]
KO:K04091	0.82	10.96	0.00	1.07	0.22	0.00	0.78	6.28E-01	2.00E-02	1.00E+00	5.59E-01	8.00E-01	1.00E+00	6.40E-01	alkanesulfonate monooxygenase [EC:1.14.14.5]
KO:K04092	0.00	6.35	0.00	0.07	0.00	0.00	0.00	1.00E+00	1.21E-02	1.00E+00	6.18E-01	1.00E+00	1.00E+00	1.00E+00	chorismate mutase [EC:5.4.99.5]
KO:K04093	0.13	0.24	7.66	0.00	0.00	0.00	0.00	3.17E-01	2.91E-01	2.54E-02	1.00E+00	1.00E+00	1.00E+00	1.00E+00	chorismate mutase [EC:5.4.99.5]
KO:K04094	0.13	13.96	0.00	0.45	14.12	0.00	0.00	9.17E-01	5.57E-03	1.00E+00	8.62E-01	5.43E-03	1.00E+00	1.00E+00	glucose inhibited division protein Gid
KO:K04096	0.68	0.25	12.50	0.15	2.72	0.18	0.40	5.40E-01	8.36E-01	3.00E-03	8.91E-01	7.14E-02	8.76E-01	7.34E-01	DNA processing protein
KO:K04097	0.68	0.91	0.00	0.83	0.00	0.00	0.00	2.17E-01	1.67E-01	1.00E+00	1.82E-01	1.00E+00	1.00E+00	1.00E+00	glutathione S-transferase [EC:2.5.1.18]
KO:K04098	0.00	0.26	0.00	0.12	0.00	0.00	0.00	1.00E+00	1.23E-01	1.00E+00	1.70E-01	1.00E+00	1.00E+00	1.00E+00	hydroxyquinol 1,2-dioxygenase [EC:1.13.11.37]
KO:K04100	0.94	1.35	0.00	1.16	0.00	0.00	0.00	2.42E-01	1.90E-01	1.00E+00	2.07E-01	1.00E+00	1.00E+00	1.00E+00	protocatechuate 4,5-dioxygenase, alpha chain [EC:1.13.11.8]
KO:K04101	2.04	1.85	0.00	0.58	0.00	0.00	0.00	1.55E-01	1.70E-01	1.00E+00	3.54E-01	1.00E+00	1.00E+00	1.00E+00	protocatechuate 4,5-dioxygenase, beta chain [EC:1.13.11.8]
KO:K04102	0.00	1.95	0.12	2.47	0.00	0.00	0.00	1.00E+00	1.56E-01	4.47E-01	1.33E-01	1.00E+00	1.00E+00	1.00E+00	4,5-dihydroxyphthalate decarboxylase [EC:4.1.1.55]
KO:K04103	0.00	0.00	2.51	0.04	1.99	0.00	0.12	1.00E+00	1.00E+00	2.39E-02	4.37E-01	3.87E-02	1.00E+00	3.41E-01	indolepyruvate decarboxylase [EC:4.1.1.74]
KO:K04105	0.00	2.00	0.00	0.00	0.00	0.00	0.00	1.00E+00	9.97E-02	1.00E+00	1.00E+00	1.00E+00	1.00E+00	1.00E+00	4-hydroxybenzoate-CoA ligase [EC:6.2.1.27]
KO:K04108	0.00	0.00	0.00	0.41	0.00	0.00	0.00	1.00E+00	1.00E+00	1.00E+00	1.43E-01	1.00E+00	1.00E+00	1.00E+00	4-hydroxybenzoyl-CoA reductase subunit 2 [EC:1.3.99.2]
KO:K04110	0.00	5.10	0.00	0.28	0.00	0.00	0.00	1.00E+00	3.81E-02	1.00E+00	3.69E-01	1.00E+00	1.00E+00	1.00E+00	benzoate-CoA ligase [EC:6.2.1.25]
KO:K04116	0.00	0.00	0.00	0.51	0.00	0.00	0.00	1.00E+00	1.00E+00	1.00E+00	7.70E-02	1.00E+00	1.00E+00	1.00E+00	cyclohexanecarboxylate-CoA ligase [EC:6.2.1.-]
KO:K04117	0.00	1.42	0.00	0.33	0.00	0.00	0.00	1.00E+00	9.89E-02	1.00E+00	2.22E-01	1.00E+00	1.00E+00	1.00E+00	cyclohexanecarboxyl-CoA dehydrogenase [EC:1.3.99.-]
KO:K04118	0.00	0.00	0.00	1.63	0.00	0.00	0.00	1.00E+00	1.00E+00	1.00E+00	1.43E-01	1.00E+00	1.00E+00	1.00E+00	pimeloyl-CoA dehydrogenase [EC:1.3.1.62]
KO:K04126	6.32	0.01	1.66	0.00	0.00	0.00	0.00	3.51E-02	6.20E-01	1.60E-01	1.00E+00	1.00E+00	1.00E+00	1.00E+00	isopenicillin-N synthase [EC:1.21.3.1]
KO:K04127	0.00	0.00	0.00	0.00	3.00	0.00	0.15	1.00E+00	1.00E+00	1.00E+00	4.05E-01	6.00E-03	1.00E+00	1.73E-01	isopenicillin-N epimerase [EC:5.1.1.17]
KO:K04333	0.00	0.00	0.00	0.00	0.00	0.00	0.42	1.00E+00	1.00E+00	1.00E+00	1.00E+00	1.00E+00	1.00E+00	2.90E-02	LuxR family transcriptional regulator, csgAB operon transcriptional
KO:K04335	0.00	0.00	0.00	0.10	0.00	0.00	0.00	1.00E+00	1.00E+00	1.00E+00	1.43E-01	1.00E+00	1.00E+00	1.00E+00	minor curlin subunit
KO:K04338	0.22	0.00	0.00	0.08	0.00	0.00	0.00	1.04E-01	1.00E+00	1.00E+00	2.02E-01	1.00E+00	1.00E+00	1.00E+00	curli production assembly/transport component CsgF
KO:K04340	2.09	0.00	0.00	0.00	0.00	0.00	0.00	5.86E-02	1.00E+00	1.00E+00	1.00E+00	1.00E+00	1.00E+00	1.00E+00	scyllo-inosamine-4-phosphate amidinotransferase 1 [EC:2.1.4.2]
KO:K04477	0.28	0.00	9.73	0.36	2.58	0.00	0.57	5.55E-01	1.00E+00	1.33E-02	5.17E-01	1.07E-01	1.00E+00	4.24E-01	putative hydrolase
KO:K04479	0.00	0.00	0.00	0.00	0.00	0.00	2.92	1.00E+00	1.00E+00	1.00E+00	1.00E+00	1.00E+00	1.00E+00	2.93E-02	DNA polymerase IV (archaeal DinB-like DNA polymerase) [EC:2.7.7.7]
KO:K04482	0.00	0.00	0.00	0.00	0.00	0.00	0.42	1.00E+00	1.00E+00	1.00E+00	1.00E+00	1.00E+00	1.00E+00	2.70E-02	DNA repair protein RAD51
KO:K04483	0.00	0.00	0.00	0.00	0.00	0.00	8.75	1.00E+00	1.00E+00	1.00E+00	1.00E+00	1.00E+00	1.00E+00	4.29E-03	DNA repair protein RadA
KO:K04485	9.59	8.60	0.53	2.33	1.99	6.08	0.46	3.31E-02	4.81E-02	9.81E-01	8.55E-01	8.91E-01	1.56E-01	9.84E-01	DNA repair protein RadA/Sms
KO:K04486	0.00	0.00	5.08	0.00	3.25	0.00	0.00	4.05E-01	1.00E+00	3.19E-02	4.08E-01	4.64E-02	1.00E+00	1.00E+00	histidinol-phosphatase (PHP family) [EC:3.1.3.15]
KO:K04487	8.76	7.02	2.75	4.83	3.48	1.49	10.00	1.27E-01	2.75E-01	9.11E-01	6.67E-01	8.61E-01	9.66E-01	8.00E-02	cysteine desulfurase [EC:2.8.1.7]
KO:K04488	5.58	0.03	0.89	1.83	0.00	0.03	22.95	1.14E-01	9.14E-01	7.44E-01	5.11E-01	9.28E-01	9.14E-01	4.29E-03	nitrogen fixation protein NifU and related proteins
KO:K04496	0.00	0.00	0.00	0.00	0.00	0.00	6.25	1.00E+00	1.00E+00	1.00E+00	1.00E+00	1.00E+00	1.00E+00	2.63E-02	C-terminal binding protein
KO:K04509	0.60	0.00	0.00	0.00	0.00	26.06	0.00	2.38E-01	1.00E+00	1.00E+00	1.00E+00	1.00E+00	4.71E-03	1.00E+00	formate dehydrogenase (cytochrome) [EC:1.2.2.1]
KO:K04515	0.00	0.00	0.00	0.00	4.38	0.00	0.00	1.00E+00	1.00E+00	1.00E+00	1.00E+00	3.14E-03	1.00E+00	1.00E+00	calcium/calmodulin-dependent protein kinase (CaM kinase) II
KO:K04516	1.40	0.34	0.00	0.88	0.00	0.84	0.00	1.40E-01	4.67E-01	1.00E+00	2.35E-01	1.00E+00	2.47E-01	1.00E+00	chorismate mutase [EC:5.4.99.5]
KO:K04517	1.20	0.29	6.35	0.68	0.11	1.25	1.02	3.72E-01	7.75E-01	2.17E-02	5.82E-01	8.46E-01	3.55E-01	4.33E-01	prephenate dehydrogenase [EC:1.3.1.12]
KO:K04518	4.45	6.25	7.18	5.52	6.45	0.00	1.20	4.40E-01	1.81E-01	1.21E-01	2.56E-01	1.66E-01	1.00E+00	9.44E-01	prephenate dehydratase [EC:4.2.1.51]
KO:K04562	10.16	0.02	0.64	0.58	0.00	6.33	1.46	2.76E-02	8.83E-01	7.27E-01	7.45E-01	1.00E+00	6.31E-02	4.86E-01	flagellar biosynthesis protein FlhG
KO:K04564	5.82	9.69	6.63	5.59	4.16	3.22	1.71	4.35E-01	9.03E-02	3.15E-01	4.79E-01	7.41E-01	8.60E-01	9.52E-01	superoxide dismutase, Fe-Mn family [EC:1.15.1.1]
KO:K04565	0.50	0.46	0.18	0.25	4.48	0.00	0.00	3.75E-01	4.03E-01	6.16E-01	5.57E-01	1.60E-02	1.00E+00	1.00E+00	Cu/Zn superoxide dismutase [EC:1.15.1.1]
KO:K04566	0.45	21.04	0.00	5.07	0.00	0.00	1.64	9.06E-01	3.00E-03	1.00E+00	1.64E-01	1.00E+00	1.00E+00	7.29E-01	lysyl-tRNA synthetase, class I [EC:6.1.1.6]
KO:K04567	7.93	0.94	10.35	2.87	9.81	2.87	1.54	9.17E-02	9.42E-01	4.17E-02	7.20E-01	5.01E-02	7.20E-01	8.97E-01	lysyl-tRNA synthetase, class II [EC:6.1.1.6]
KO:K04568	4.53	0.21	0.92	0.59	0.00	2.37	0.41	3.87E-02	8.37E-01	4.68E-01	6.40E-01	1.00E+00	1.26E-01	7.40E-01	lysyl-tRNA synthetase, class II [EC:6.1.1.6]
KO:K04651	0.07	0.00	0.00	0.00	2.11	0.00	0.00	2.33E-01	1.00E+00	1.00E+00	1.00E+00	8.86E-03	1.00E+00	1.00E+00	hydrogenase nickel incorporation protein HypA
KO:K04652	0.15	0.01	0.00	0.01	2.24	0.00	0.00	2.48E-01	5.67E-01	1.00E+00	5.76E-01	6.29E-03	1.00E+00	1.00E+00	hydrogenase nickel incorporation protein HypB
KO:K04653	0.28	0.01	0.00	0.22	0.00	0.00	0.00	1.30E-01	2.98E-01	1.00E+00	1.98E-01	1.00E+00	1.00E+00	1.00E+00	hydrogenase expression/formation protein HypC
KO:K04654	0.07	1.09	0.08	0.01	0.31	0.00	0.00	4.84E-01	6.06E-02	4.59E-01	6.54E-01	2.39E-01	1.00E+00	1.00E+00	hydrogenase expression/formation protein HypD
KO:K04655	0.02	0.07	0.00	0.60	3.43	0.00	0.00	5.67E-01	4.43E-01	1.00E+00	1.88E-01	3.40E-02	1.00E+00	1.00E+00	hydrogenase expression/formation protein HypE
KO:K04656	0.12	0.07	0.00	0.09	0.85	0.00	0.04	3.39E-01	4.36E-01	1.00E+00	3.94E-01	5.11E-02	1.00E+00	5.36E-01	hydrogenase maturation protein HypF
KO:K04691	2.99	0.32	0.00	0.12	0.00	8.11	0.13	9.69E-02	5.68E-01	1.00E+00	6.90E-01	1.00E+00	1.57E-02	6.86E-01	serine protease DegS [EC:3.4.21.-]
KO:K04708	0.00	0.00	1.25	0.00	0.00	0.00	0.00	1.00E+00	1.00E+00	1.66E-02	1.00E+00	1.00E+00	1.00E+00	1.00E+00	3-dehydrosphinganine reductase [EC:1.1.1.102]
KO:K04712	1.35	0.00	0.00	0.12	0.00	0.00	0.00	6.46E-02	1.00E+00	1.00E+00	2.82E-01	1.00E+00	1.00E+00	1.00E+00	sphingolipid delta-4 desaturase [EC:1.14.-.-]
KO:K04719	0.02	0.02	0.00	0.00	0.00	0.00	1.25	4.02E-01	4.07E-01	1.00E+00	5.22E-01	1.00E+00	1.00E+00	1.23E-02	5,6-dimethylbenzimidazole biosynthesis protein BluB
KO:K04720	0.00	0.00	0.00	0.00	1.25	0.00	0.00	1.00E+00	1.00E+00	1.00E+00	1.00E+00	1.70E-02	1.00E+00	1.00E+00	threonine-phosphate decarboxylase [EC:4.1.1.81]
KO:K04744	5.57	4.29	1.37	3.86	0.00	0.48	1.81	9.74E-02	1.82E-01	7.95E-01	2.30E-01	1.00E+00	9.31E-01	6.91E-01	LPS-assembly protein

KO:K04748	0.24	0.04	0.00	1.04	0.00	3.13	7.85	6.10E-01	7.72E-01	1.00E+00	2.99E-01	1.00E+00	8.19E-02	2.21E-02	nitric-oxide reductase NorQ protein [EC:1.7.99.7]
KO:K04749	0.07	0.01	3.83	0.26	0.96	0.00	0.00	5.20E-01	6.57E-01	2.04E-02	3.30E-01	1.39E-01	1.00E+00	1.00E+00	anti-sigma B factor antagonist
KO:K04750	0.01	0.00	0.00	0.19	0.00	0.00	0.79	3.12E-01	1.00E+00	1.00E+00	1.74E-01	1.00E+00	1.00E+00	6.93E-02	PhnB protein
KO:K04751	1.13	8.29	6.27	1.59	4.22	1.72	2.70	8.86E-01	5.94E-02	1.22E-01	8.27E-01	3.12E-01	8.09E-01	6.21E-01	nitrogen regulatory protein P-II 1
KO:K04752	6.15	0.26	6.85	2.61	2.04	0.02	11.54	1.31E-01	9.25E-01	1.02E-01	5.45E-01	6.63E-01	9.58E-01	2.70E-02	nitrogen regulatory protein P-II 2
KO:K04753	0.12	0.00	0.00	0.10	0.00	0.00	0.00	1.03E-01	1.00E+00	1.00E+00	2.07E-01	1.00E+00	1.00E+00	1.00E+00	suppressor of ftsI
KO:K04754	10.07	6.35	0.17	8.49	0.00	2.32	1.73	5.91E-02	1.96E-01	9.73E-01	9.26E-02	1.00E+00	8.11E-01	8.74E-01	lipoprotein
KO:K04755	6.53	5.77	2.70	4.06	0.12	0.00	1.98	9.99E-02	1.36E-01	5.54E-01	2.92E-01	9.73E-01	1.00E+00	7.18E-01	ferredoxin, 2Fe-2S
KO:K04756	0.06	0.00	0.00	0.50	1.83	0.00	0.00	4.21E-01	1.00E+00	1.00E+00	1.58E-01	3.76E-02	1.00E+00	1.00E+00	alkyl hydroperoxide reductase subunit D
KO:K04757	0.12	0.00	0.54	4.31	0.00	0.00	0.01	5.13E-01	6.48E-01	3.06E-01	4.49E-02	1.00E+00	1.00E+00	6.20E-01	anti-sigma B factor [EC:2.7.11.1]
KO:K04758	0.47	0.13	2.23	1.58	0.18	0.00	0.01	4.04E-01	6.71E-01	7.81E-02	1.29E-01	6.04E-01	1.00E+00	8.32E-01	ferrous iron transport protein A
KO:K04759	6.58	0.96	6.98	3.21	0.40	2.84	0.33	8.70E-02	8.31E-01	7.73E-02	3.62E-01	9.05E-01	4.30E-01	9.14E-01	ferrous iron transport protein B
KO:K04760	1.93	0.05	0.28	1.28	0.00	0.00	0.06	6.69E-02	7.90E-01	5.30E-01	1.21E-01	1.00E+00	1.00E+00	7.83E-01	transcription elongation factor GreB
KO:K04761	6.48	3.19	0.27	2.76	0.00	5.02	0.36	5.41E-02	2.59E-01	9.51E-01	3.44E-01	1.00E+00	9.63E-02	9.38E-01	LysR family transcriptional regulator, hydrogen peroxide-inducible
KO:K04762	3.84	2.11	0.17	0.12	0.09	1.19	0.30	5.24E-02	1.52E-01	8.65E-01	8.83E-01	8.92E-01	3.46E-01	7.93E-01	ribosome-associated heat shock protein Hsp15
KO:K04763	6.49	2.42	6.52	2.22	1.70	0.57	0.21	6.69E-02	5.15E-01	6.59E-02	5.70E-01	7.04E-01	9.12E-01	9.64E-01	integrase/recombinase XerD
KO:K04764	7.54	4.17	1.97	4.04	0.00	7.41	1.67	9.70E-02	3.82E-01	7.96E-01	4.06E-01	1.00E+00	1.01E-01	8.37E-01	integration host factor subunit alpha
KO:K04767	0.47	0.00	0.00	0.55	0.00	0.00	0.58	2.01E-01	1.00E+00	1.00E+00	1.81E-01	1.00E+00	1.00E+00	1.76E-01	acetoin utilization protein AcuB
KO:K04768	0.01	5.41	0.00	0.13	0.00	0.00	0.00	7.24E-01	1.31E-02	1.00E+00	5.75E-01	1.00E+00	1.00E+00	1.00E+00	acetoin utilization protein AcuC
KO:K04771	0.31	0.05	22.36	0.10	0.00	0.00	0.74	5.11E-01	6.81E-01	1.14E-03	6.40E-01	1.00E+00	1.00E+00	3.63E-01	serine protease Do [EC:3.4.21.17]
KO:K04772	1.79	0.00	0.00	0.10	0.00	0.00	0.00	4.17E-02	1.00E+00	1.00E+00	3.40E-01	1.00E+00	1.00E+00	1.00E+00	serine protease DegQ [EC:3.4.21.-]
KO:K04773	7.16	0.39	4.52	2.48	15.38	0.46	0.37	7.34E-02	9.43E-01	2.04E-01	5.70E-01	7.43E-03	9.35E-01	9.44E-01	protease IV [EC:3.4.21.-]
KO:K04774	5.27	24.43	0.09	0.35	0.00	3.86	0.01	1.85E-01	4.29E-04	9.59E-01	9.37E-01	1.00E+00	3.51E-01	9.69E-01	serine protease SohB [EC:3.4.21.-]
KO:K04775	0.00	0.58	0.00	0.01	0.00	0.00	0.00	1.00E+00	3.51E-02	1.00E+00	4.02E-01	1.00E+00	1.00E+00	1.00E+00	protease YdgD [EC:3.4.21.-]
KO:K04782	0.00	0.75	0.00	1.12	0.00	0.00	0.00	1.00E+00	1.53E-01	1.00E+00	1.09E-01	1.00E+00	1.00E+00	1.00E+00	isochorismate pyruvate-lyase [EC:4.1.3.-]
KO:K04794	0.00	0.00	0.00	0.00	0.00	0.00	2.50	1.00E+00	1.00E+00	1.00E+00	1.00E+00	1.00E+00	1.00E+00	6.14E-03	peptidyl-tRNA hydrolase, PTH2 family [EC:3.1.1.29]
KO:K04795	0.00	0.00	0.00	0.00	0.00	0.00	2.50	1.00E+00	1.00E+00	1.00E+00	1.00E+00	1.00E+00	1.00E+00	2.61E-02	fibrillarin-like pre-rRNA processing protein
KO:K04796	0.00	0.00	0.00	0.00	0.00	0.00	6.67	1.00E+00	1.00E+00	1.00E+00	1.00E+00	1.00E+00	1.00E+00	4.29E-03	small nuclear ribonucleoprotein
KO:K04797	0.00	0.00	0.00	0.00	0.00	0.00	2.92	1.00E+00	1.00E+00	1.00E+00	1.00E+00	1.00E+00	1.00E+00	2.69E-02	prefoldin alpha subunit
KO:K04798	0.00	0.00	0.00	0.00	0.00	0.00	2.08	1.00E+00	1.00E+00	1.00E+00	1.00E+00	1.00E+00	1.00E+00	2.70E-02	prefoldin beta subunit
KO:K04799	0.00	0.00	0.00	0.00	0.00	0.00	1.67	1.00E+00	1.00E+00	1.00E+00	1.00E+00	1.00E+00	1.00E+00	1.44E-02	flap endonuclease-1 [EC:3.-.-.-]
KO:K04800	0.00	0.00	0.00	0.00	0.00	0.00	2.92	1.00E+00	1.00E+00	1.00E+00	1.00E+00	1.00E+00	1.00E+00	1.17E-02	replication factor C large subunit
KO:K04801	0.00	0.00	0.00	0.00	0.00	0.00	2.92	1.00E+00	1.00E+00	1.00E+00	1.00E+00	1.00E+00	1.00E+00	2.87E-02	replication factor C small subunit
KO:K04802	0.00	0.00	0.00	0.00	0.00	0.00	6.67	1.00E+00	1.00E+00	1.00E+00	1.00E+00	1.00E+00	1.00E+00	9.14E-03	proliferating cell nuclear antigen
KO:K04940	0.07	2.58	0.00	0.46	0.00	0.00	0.00	4.39E-01	7.87E-02	1.00E+00	2.84E-01	1.00E+00	1.00E+00	1.00E+00	opine dehydrogenase [EC:1.5.1.28]
KO:K05020	0.00	0.26	0.00	0.02	0.00	0.00	1.64	1.00E+00	1.98E-01	1.00E+00	3.22E-01	1.00E+00	1.00E+00	4.80E-02	glycine betaine transporter
KO:K05281	0.00	0.00	0.00	0.10	0.42	0.00	0.00	1.00E+00	1.00E+00	1.00E+00	1.65E-01	5.49E-02	1.00E+00	1.00E+00	2'-hydroxyisoflavone reductase [EC:1.3.1.45]
KO:K05296	3.95	0.00	0.00	0.00	0.00	0.00	0.00	5.60E-02	1.00E+00	1.00E+00	1.00E+00	1.00E+00	1.00E+00	1.00E+00	3(or 17)beta-hydroxysteroid dehydrogenase [EC:1.1.1.51]
KO:K05297	3.07	0.00	0.10	0.01	0.00	1.55	0.27	7.83E-02	1.00E+00	4.37E-01	6.31E-01	1.00E+00	1.34E-01	3.43E-01	rubredoxin-NAD+ reductase [EC:1.18.1.1]
KO:K05301	1.74	17.18	0.00	0.67	0.00	0.00	0.00	4.05E-01	7.71E-03	1.00E+00	6.82E-01	1.00E+00	1.00E+00	1.00E+00	sulfite dehydrogenase [EC:1.8.2.1]
KO:K05303	0.00	0.27	0.00	0.02	0.00	0.00	0.70	1.00E+00	1.64E-01	1.00E+00	3.17E-01	1.00E+00	1.00E+00	8.26E-02	macrocin O-methyltransferase [EC:2.1.1.11]
KO:K05304	0.00	0.00	0.00	1.02	0.00	0.00	0.00	1.00E+00	1.00E+00	1.00E+00	6.04E-02	1.00E+00	1.00E+00	1.00E+00	sialic acid synthase [EC:2.5.1.56 2.5.1.57]
KO:K05306	0.00	0.00	0.00	0.14	1.33	0.00	0.33	5.27E-01	1.00E+00	1.00E+00	2.52E-01	2.97E-02	1.00E+00	1.53E-01	phosphonoacetaldehyde hydrolase [EC:3.11.1.1]
KO:K05308	0.81	0.00	0.00	0.00	0.00	0.00	0.00	6.29E-02	1.00E+00	1.00E+00	1.00E+00	1.00E+00	1.00E+00	1.00E+00	gluconate dehydratase [EC:4.2.1.39]
KO:K05337	0.01	0.00	5.73	0.00	9.80	0.00	0.34	6.94E-01	1.00E+00	1.54E-02	7.14E-01	3.71E-03	1.00E+00	3.83E-01	ferredoxin
KO:K05340	0.35	0.00	0.00	0.00	0.00	0.00	0.00	4.89E-02	1.00E+00	1.00E+00	1.00E+00	1.00E+00	1.00E+00	1.00E+00	glucose uptake protein
KO:K05341	0.04	0.16	0.00	0.11	3.28	0.00	0.00	4.55E-01	3.44E-01	1.00E+00	3.82E-01	2.09E-02	1.00E+00	1.00E+00	amylosucrase [EC:2.4.1.4]
KO:K05343	0.00	0.00	0.40	0.02	16.54	0.00	0.00	6.82E-01	6.92E-01	2.64E-01	6.39E-01	1.43E-04	1.00E+00	1.00E+00	maltose alpha-D-glucosyltransferase [EC:5.4.99.16]
KO:K05345	1.37	0.00	0.00	0.18	0.00	0.00	2.21	1.33E-01	1.00E+00	1.00E+00	3.84E-01	1.00E+00	1.00E+00	8.71E-02	putative cyclase [EC:4.6.1.-]
KO:K05349	3.71	0.31	1.18	0.45	4.41	3.73	0.02	1.18E-01	7.59E-01	4.66E-01	7.13E-01	9.00E-02	1.17E-01	8.85E-01	beta-glucosidase [EC:3.2.1.21]
KO:K05350	0.51	4.82	0.00	0.21	0.00	0.00	0.05	4.23E-01	1.61E-02	1.00E+00	6.44E-01	1.00E+00	1.00E+00	7.96E-01	beta-glucosidase [EC:3.2.1.21]
KO:K05351	0.89	0.04	0.00	0.13	0.00	0.00	0.06	1.13E-01	3.60E-01	1.00E+00	2.40E-01	1.00E+00	1.00E+00	3.20E-01	D-xylulose reductase [EC:1.1.1.9]
KO:K05356	0.00	0.00	0.00	0.00	16.25	0.00	0.00	1.00E+00	1.00E+00	1.00E+00	1.00E+00	2.86E-04	1.00E+00	1.00E+00	solanesyl diphosphate synthase [EC:2.5.1.11]
KO:K05358	1.56	0.01	0.00	0.24	0.00	0.00	0.00	8.61E-02	3.74E-01	1.00E+00	2.57E-01	1.00E+00	1.00E+00	1.00E+00	quinate dehydrogenase (pyrroloquinoline-quinone) [EC:1.1.99.25]
KO:K05364	0.00	0.01	0.00	1.32	1.57	0.00	0.00	1.00E+00	4.76E-01	1.00E+00	1.03E-01	8.59E-02	1.00E+00	1.00E+00	peptidoglycan glycosyltransferase [EC:2.4.1.129]
KO:K05365	1.50	0.01	0.06	0.33	0.00	6.27	0.50	1.33E-01	8.12E-01	7.64E-01	5.28E-01	1.00E+00	1.16E-02	4.19E-01	penicillin-binding protein 1B [EC:2.4.1.129 3.4.-.-]
KO:K05366	10.09	16.91	0.71	6.60	0.39	5.06	0.22	6.41E-02	4.86E-03	9.89E-01	3.08E-01	9.96E-01	6.23E-01	9.98E-01	penicillin-binding protein 1A [EC:2.4.1.- 3.4.-.-]
KO:K05367	0.06	0.18	0.07	0.48	0.00	0.50	0.00	6.10E-01	3.61E-01	5.80E-01	1.21E-01	1.00E+00	1.14E-01	1.00E+00	penicillin-binding protein 1C [EC:2.4.1.-]
KO:K05368	0.35	0.00	0.00	0.00	0.00	0.00	0.00	4.84E-02	1.00E+00	1.00E+00	1.00E+00	1.00E+00	1.00E+00	1.00E+00	aquacobalamin reductase / NAD(P)H-flavin reductase [EC:1.16.1.3
KO:K05369	0.00	0.00	0.00	0.00	3.75	0.00	0.00	1.00E+00	1.00E+00	1.00E+00	1.00E+00	2.43E-03	1.00E+00	1.00E+00	15,16-dihydrobiliverdin:ferredoxin oxidoreductase [EC:1.3.7.2]
KO:K05370	0.00	0.00	0.00	0.00	10.63	0.00	0.00	1.00E+00	1.00E+00	1.00E+00	1.00E+00	1.43E-04	1.00E+00	1.00E+00	phycoerythrobilin:ferredoxin oxidoreductase [EC:1.3.7.3]
KO:K05371	0.00	0.00	0.00	0.14	16.01	0.00	0.00	1.00E+00	1.00E+00	1.00E+00	3.69E-01	2.86E-03	1.00E+00	1.00E+00	phycocyanobilin:ferredoxin oxidoreductase [EC:1.3.7.5]
KO:K05375	0.35	0.00	0.00	0.00	0.00	0.00	0.00	5.26E-02	1.00E+00	1.00E+00	1.00E+00	1.00E+00	1.00E+00	1.00E+00	MbtH protein
KO:K05376	0.00	0.00	0.00	0.00	31.25	0.00	0.00	1.00E+00	1.00E+00	1.00E+00	1.00E+00	0.00E+00	1.00E+00	1.00E+00	phycoerythrin alpha chain
KO:K05377	0.00	0.00	0.00	0.00	33.13	0.00	0.00	1.00E+00	1.00E+00	1.00E+00	1.00E+00	0.00E+00	1.00E+00	1.00E+00	phycoerythrin beta chain
KO:K05378	0.00	0.00	0.00	0.00	20.63	0.00	0.00	1.00E+00	1.00E+00	1.00E+00	1.00E+00	0.00E+00	1.00E+00	1.00E+00	phycoerythrin-associated linker protein
KO:K05379	0.00	0.00	0.00	0.00	12.50	0.00	0.00	1.00E+00	1.00E+00	1.00E+00	1.00E+00	2.43E-03	1.00E+00	1.00E+00	phycoerythrin-associated linker protein
KO:K05380	0.00	0.00	0.00	0.00	4.38	0.00	0.00	1.00E+00	1.00E+00	1.00E+00	1.00E+00	2.71E-03	1.00E+00	1.00E+00	phycoerythrin-associated linker protein
KO:K05381	0.00	0.00	0.00	0.00	2.50	0.00	0.00	1.00E+00	1.00E+00	1.00E+00	1.00E+00	2.57E-03	1.00E+00	1.00E+00	phycoerythrin-associated linker protein
KO:K05382	0.00	0.00	0.00	0.00	3.13	0.00	0.00	1.00E+00	1.00E+00	1.00E+00	1.00E+00	0.00E+00	1.00E+00	1.00E+00	phycoerythrin-associated linker protein
KO:K05383	0.00	0.00	0.00	0.00	4.38	0.00	0.00	1.00E+00	1.00E+00	1.00E+00	1.00E+00	5.00E-03	1.00E+00	1.00E+00	CpeT protein
KO:K05384	0.00	0.00	0.00	0.00	4.38	0.00	0.00	1.00E+00	1.00E+00	1.00E+00	1.00E+00	5.14E-03	1.00E+00	1.00E+00	bilin biosynthesis protein
KO:K05385	0.00	0.00	0.00	0.00	6.25	0.00	0.00	1.00E+00	1.00E+00	1.00E+00	1.00E+00	2.71E-03	1.00E+00	1.00E+00	bilin biosynthesis protein
KO:K05386	0.00	0.00	0.00	0.00	8.13	0.00	0.00	1.00E+00	1.00E+00	1.00E+00	1.00E+00	3.71E-03	1.00E+00	1.00E+00	bilin biosynthesis protein
KO:K05394	0.00	0.14	0.00	1.22	0.00	0.00	0.00	1.00E+00	2.28E-01	1.00E+00	8.04E-02	1.00E+00	1.00E+00	1.00E+00	atrazine chlorohydrolase [EC:3.8.1.8]
KO:K05396	6.16	8.69	0.00	0.49	0.00	0.00	0.04	6.84E-02	3.87E-02	1.00E+00	7.48E-01	1.00E+00	1.00E+00	8.67E-01	D-cysteine desulfhydrase [EC:4.4.1.15]
KO:K05499	0.05	0.97	0.80	1.51	0.00	0.00	0.00	5.88E-01	1.88E-01	2.17E-01	1.34E-01	1.00E+00	1.00E+00	1.00E+00	LacI family transcriptional regulator, repressor for deo operon,
KO:K05501	9.45	0.47	0.56	0.21	0.00	0.00	0.74	1.61E-02	6.62E-01	6.25E-01	7.60E-01	1.00E+00	1.00E+00	5.58E-01	TetR/AcrR family transcriptional regulator
KO:K05515	8.29	6.07	2.90	4.21	5.09	1.22	1.22	6.04E-02	1.86E-01	8.26E-01	5.41E-01	3.43E-01	9.68E-01	9.68E-01	penicillin-binding protein 2
KO:K05516	0.09	0.00	0.26	0.08	20.82	0.00	0.22	6.99E-01	8.27E-01	5.26E-01	7.15E-01	0.00E+00	1.00E+00	5.53E-01	curved DNA-binding protein
KO:K05520	0.83	0.01	7.93	0.77	0.82	0.00	0.11	3.40E-01	7.63E-01	2.51E-02	3.53E-01	3.40E-01	1.00E+00	6.36E-01	protease I [EC:3.2.-.-]
KO:K05521	0.18	0.09	19.56	0.13	0.01	0.00	0.00	5.02E-01	5.91E-01	4.57E-03	5.37E-01	7.14E-01	1.00E+00	7.31E-01	ADP-ribosylglycohydrolase [EC:3.2.-.-]
KO:K05522	4.22	0.00	0.36	0.00	2.22	0.00	0.00	4.74E-02	1.00E+00	4.04E-01	6.65E-01	1.05E-01	1.00E+00	1.00E+00	endonuclease VIII [EC:3.2.2.- 4.2.99.18]
KO:K05523	1.55	0.03	0.00	0.00	0.00	0.00	0.00	4.30E-02	3.58E-01	1.00E+00	1.00E+00	1.00E+00	1.00E+00	1.00E+00	molecular chaperone HchA (Hsp31)
KO:K05524	6.31	3.63	7.56	5.82	0.61	2.85	0.44	1.65E-01	5.30E-01	1.03E-01	2.03E-01	9.45E-01	6.80E-01	9.57E-01	ferredoxin
KO:K05525	0.80	0.86	0.00	1.65	0.00	0.00	0.00	2.43E-01	2.30E-01	1.00E+00	1.38E-01	1.00E+00	1.00E+00	1.00E+00	linalool 8-monooxygenase [EC:1.14.99.28]
KO:K05526	0.09	0.09	0.00	1.67	0.00	0.00	0.00	3.51E-01	3.64E-01	1.00E+00	5.16E-02	1.00E+00	1.00E+00	1.00E+00	succinylglutamate desuccinylase [EC:3.5.1.96]
KO:K05527	6.42	1.83	0.44	0.87	0.00	0.00	1.16	2.80E-02	3.01E-01	8.03E-01	6.43E-01	1.00E+00	1.00E+00	5.26E-01	BolA protein
KO:K05539	4.03	7.89	0.16	2.81	9.34	0.21	0.18	2.68E-01	4.53E-02	9.84E-01	5.39E-01	2.69E-02	9.77E-01	9.81E-01	tRNA-dihydrouridine synthase A [EC:1.-.-.-]
KO:K05540	5.36	10.46	4.14	1.10	0.00	0.00	0.77	1.08E-01	1.50E-02	2.04E-01	8.46E-01	1.00E+00	1.00E+00	8.94E-01	tRNA-dihydrouridine synthase B [EC:1.-.-.-]
KO:K05541	2.21	0.02	1.10	0.01	1.72	0.00	0.49	9.04E-02	7.40E-01	1.90E-01	7.57E-01	1.18E-01	1.00E+00	3.65E-01	tRNA-dihydrouridine synthase C [EC:1.-.-.-]
KO:K05549	0.29	0.43	0.00	1.26	0.00	0.00	0.00	3.35E-01	2.69E-01	1.00E+00	1.14E-01	1.00E+00	1.00E+00	1.00E+00	benzoate 1,2-dioxygenase alpha subunit [EC:1.14.12.1]
KO:K05550	0.00	0.00	0.00	0.92	0.00	0.00	0.00	1.00E+00	1.00E+00	1.00E+00	1.43E-01	1.00E+00	1.00E+00	1.00E+00	benzoate 1,2-dioxygenase beta subunit [EC:1.14.12.1]
KO:K05559	0.22	2.02	0.04	0.10	0.00	0.31	0.00	5.13E-01	2.11E-02	8.13E-01	7.46E-01	1.00E+00	3.65E-01	1.00E+00	multicomponent K+:H+ antiporter subunit A
KO:K05560	0.02	0.46	0.00	0.00	0.00	0.00	0.00	3.67E-01	2.76E-02	1.00E+00	1.00E+00	1.00E+00	1.00E+00	1.00E+00	multicomponent K+:H+ antiporter subunit C
KO:K05561	0.07	1.92	0.00	0.01	0.00	0.00	0.00	5.32E-01	1.63E-02	1.00E+00	7.33E-01	1.00E+00	1.00E+00	1.00E+00	multicomponent K+:H+ antiporter subunit D

KO:K05562	0.07	0.42	0.21	0.00	0.00	0.00	0.00	3.80E-01	7.31E-02	1.41E-01	1.00E+00	1.00E+00	1.00E+00	1.00E+00	multicomponent K+:H+ antiporter subunit E
KO:K05563	0.05	0.09	0.33	0.00	0.00	0.00	0.00	2.88E-01	2.03E-01	5.56E-02	1.00E+00	1.00E+00	1.00E+00	1.00E+00	multicomponent K+:H+ antiporter subunit F
KO:K05564	0.00	0.69	0.00	0.01	0.00	0.00	0.00	1.00E+00	4.06E-02	1.00E+00	4.20E-01	1.00E+00	1.00E+00	1.00E+00	multicomponent K+:H+ antiporter subunit G
KO:K05565	0.02	0.02	0.00	0.45	0.55	0.00	0.00	5.62E-01	5.69E-01	1.00E+00	1.02E-01	6.94E-02	1.00E+00	1.00E+00	multicomponent Na+:H+ antiporter subunit A
KO:K05566	1.62	0.17	5.38	1.23	0.57	0.00	0.02	2.50E-01	6.32E-01	6.27E-02	3.05E-01	4.65E-01	1.00E+00	7.23E-01	multicomponent Na+:H+ antiporter subunit B
KO:K05567	1.45	0.17	2.73	0.87	0.40	0.00	0.01	2.09E-01	5.94E-01	1.08E-01	3.16E-01	4.67E-01	1.00E+00	7.66E-01	multicomponent Na+:H+ antiporter subunit C
KO:K05568	2.82	0.13	11.53	2.61	0.00	0.00	0.00	2.12E-01	7.12E-01	2.90E-02	2.28E-01	7.83E-01	1.00E+00	1.00E+00	multicomponent Na+:H+ antiporter subunit D
KO:K05569	0.96	0.19	2.79	1.19	0.07	0.12	0.01	2.78E-01	5.67E-01	1.11E-01	2.36E-01	6.70E-01	6.19E-01	7.55E-01	multicomponent Na+:H+ antiporter subunit E
KO:K05570	1.82	0.23	1.59	0.77	0.00	0.00	0.00	1.50E-01	4.93E-01	1.69E-01	2.94E-01	1.00E+00	1.00E+00	1.00E+00	multicomponent Na+:H+ antiporter subunit F
KO:K05571	1.71	0.28	1.20	0.94	0.00	0.00	0.01	1.45E-01	4.89E-01	2.08E-01	2.54E-01	1.00E+00	1.00E+00	7.34E-01	multicomponent Na+:H+ antiporter subunit G
KO:K05572	0.00	0.00	0.00	0.00	43.13	0.00	0.00	1.00E+00	1.00E+00	1.00E+00	1.00E+00	0.00E+00	1.00E+00	1.00E+00	NADH dehydrogenase I subunit 1 [EC:1.6.5.3]
KO:K05573	0.00	0.00	0.00	0.00	47.50	0.00	0.00	1.00E+00	1.00E+00	1.00E+00	1.00E+00	0.00E+00	1.00E+00	1.00E+00	NADH dehydrogenase I subunit 2 [EC:1.6.5.3]
KO:K05574	0.00	0.00	0.00	0.00	35.63	0.00	0.00	1.00E+00	1.00E+00	1.00E+00	1.00E+00	0.00E+00	1.00E+00	1.00E+00	NADH dehydrogenase I subunit 3 [EC:1.6.5.3]
KO:K05575	0.00	0.00	0.00	0.00	52.50	0.00	0.00	1.00E+00	1.00E+00	1.00E+00	1.00E+00	0.00E+00	1.00E+00	1.00E+00	NADH dehydrogenase I subunit 4 [EC:1.6.5.3]
KO:K05576	0.00	0.00	0.00	0.00	28.10	0.00	0.48	5.42E-01	1.00E+00	1.00E+00	1.00E+00	2.86E-04	1.00E+00	2.84E-01	NADH dehydrogenase I subunit 4L [EC:1.6.5.3]
KO:K05577	0.00	0.00	0.00	0.04	49.13	0.00	0.01	7.15E-01	7.21E-01	1.00E+00	6.33E-01	1.43E-04	1.00E+00	7.00E-01	NADH dehydrogenase I subunit 5 [EC:1.6.5.3]
KO:K05578	0.00	0.00	0.00	0.00	45.84	0.00	0.00	1.00E+00	1.00E+00	1.00E+00	1.00E+00	0.00E+00	1.00E+00	5.18E-01	NADH dehydrogenase I subunit 6 [EC:1.6.5.3]
KO:K05579	0.00	0.00	0.00	0.00	33.13	0.00	0.00	1.00E+00	1.00E+00	1.00E+00	1.00E+00	1.43E-04	1.00E+00	1.00E+00	NADH dehydrogenase I subunit 7 [EC:1.6.5.3]
KO:K05580	0.00	0.00	0.00	0.00	33.13	0.00	0.00	1.00E+00	1.00E+00	1.00E+00	1.00E+00	0.00E+00	1.00E+00	1.00E+00	NADH dehydrogenase I subunit I [EC:1.6.5.3]
KO:K05581	0.00	0.00	0.00	0.00	26.25	0.00	0.00	1.00E+00	1.00E+00	1.00E+00	1.00E+00	0.00E+00	1.00E+00	1.00E+00	NADH dehydrogenase I subunit J [EC:1.6.5.3]
KO:K05582	0.00	0.00	0.00	0.00	41.88	0.00	0.00	1.00E+00	1.00E+00	1.00E+00	1.00E+00	0.00E+00	1.00E+00	1.00E+00	NADH dehydrogenase I subunit K [EC:1.6.5.3]
KO:K05583	0.00	0.00	0.00	0.00	33.13	0.00	0.00	1.00E+00	1.00E+00	1.00E+00	1.00E+00	1.43E-04	1.00E+00	1.00E+00	NADH dehydrogenase I subunit L [EC:1.6.5.3]
KO:K05584	0.00	0.00	0.00	0.00	16.25	0.00	0.00	1.00E+00	1.00E+00	1.00E+00	1.00E+00	1.43E-04	1.00E+00	1.00E+00	NADH dehydrogenase I subunit M [EC:1.6.5.3]
KO:K05585	0.00	0.00	0.00	0.00	19.38	0.00	0.00	1.00E+00	1.00E+00	1.00E+00	1.00E+00	1.43E-04	1.00E+00	1.00E+00	NADH dehydrogenase I subunit N [EC:1.6.5.3]
KO:K05587	0.00	0.00	0.00	0.01	0.54	0.00	0.00	1.00E+00	1.00E+00	1.00E+00	2.51E-01	1.46E-02	1.00E+00	1.00E+00	diaphorase subunit of the bidirectional hydrogenase [EC:1.6.5.3]
KO:K05588	0.00	0.00	0.00	0.00	1.88	0.00	0.00	1.00E+00	1.00E+00	1.00E+00	1.00E+00	2.71E-03	1.00E+00	1.00E+00	diaphorase subunit of the bidirectional hydrogenase [EC:1.6.5.3]
KO:K05589	4.95	0.03	0.01	0.37	0.00	9.84	0.02	3.81E-02	8.48E-01	8.68E-01	6.57E-01	1.00E+00	5.71E-03	8.54E-01	cell division protein FtsB
KO:K05590	0.27	0.00	0.00	0.02	0.00	0.00	0.00	8.29E-02	1.00E+00	1.00E+00	2.28E-01	1.00E+00	1.00E+00	1.00E+00	ATP-dependent RNA helicase SrmB [EC:2.7.7.-]
KO:K05591	2.70	0.04	0.00	0.06	0.00	0.26	0.41	3.01E-02	7.28E-01	1.00E+00	6.90E-01	1.00E+00	4.28E-01	3.14E-01	ATP-independent RNA helicase DbpA [EC:3.6.4.13]
KO:K05592	4.91	6.88	4.77	2.34	11.24	0.68	1.83	3.40E-01	1.19E-01	3.67E-01	8.61E-01	2.13E-02	9.75E-01	9.09E-01	ATP-dependent RNA helicase DeaD [EC:3.6.4.13]
KO:K05595	2.23	6.23	0.00	1.40	0.03	2.52	0.00	2.64E-01	3.47E-02	1.00E+00	4.85E-01	9.56E-01	2.14E-01	9.68E-01	multiple antibiotic resistance protein
KO:K05596	0.00	0.11	0.00	0.00	0.00	0.00	0.00	1.00E+00	1.01E-01	1.00E+00	1.00E+00	1.00E+00	1.00E+00	1.00E+00	LysR family transcriptional regulator, chromosome initiation
KO:K05601	0.00	0.00	0.00	0.20	0.00	0.00	0.00	1.00E+00	1.00E+00	1.00E+00	1.43E-01	1.00E+00	1.00E+00	1.00E+00	hydroxylamine reductase [EC:1.7.99.1]
KO:K05603	0.58	0.86	0.00	1.15	0.00	0.00	0.00	2.47E-01	1.74E-01	1.00E+00	1.30E-01	1.00E+00	1.00E+00	1.00E+00	formimidoylglutamate deiminase [EC:3.5.3.13]
KO:K05605	0.00	0.00	0.00	1.02	0.00	0.00	0.00	1.00E+00	1.00E+00	1.00E+00	5.17E-02	1.00E+00	1.00E+00	1.00E+00	3-hydroxyisobutyryl-CoA hydrolase [EC:3.1.2.4]
KO:K05606	0.03	14.27	0.00	0.37	0.00	0.00	0.00	8.72E-01	2.29E-03	1.00E+00	6.83E-01	1.00E+00	1.00E+00	1.00E+00	methylmalonyl-CoA epimerase [EC:5.1.99.1]
KO:K05642	0.00	0.00	0.00	0.00	0.00	0.00	0.42	1.00E+00	1.00E+00	1.00E+00	1.00E+00	1.00E+00	1.00E+00	2.76E-02	ATP-binding cassette, subfamily A (ABC1), member 2
KO:K05658	0.00	0.00	0.00	0.61	0.63	0.00	0.00	1.00E+00	1.00E+00	1.00E+00	1.47E-01	1.43E-01	1.00E+00	1.00E+00	ATP-binding cassette, subfamily B (MDR/TAP), member 1
KO:K05685	0.73	0.07	2.88	0.17	0.00	0.20	0.00	2.07E-01	5.02E-01	5.33E-02	4.05E-01	1.00E+00	3.74E-01	1.00E+00	macrolide transport system ATP-binding/permease protein [EC:3.6.3.-]
KO:K05708	0.37	0.14	0.00	0.98	0.00	0.00	0.00	2.24E-01	2.78E-01	1.00E+00	1.50E-01	1.00E+00	1.00E+00	1.00E+00	large terminal subunit of phenylpropionate dioxygenase
KO:K05709	0.17	0.62	0.00	0.05	0.00	0.00	0.00	2.41E-01	1.18E-01	1.00E+00	3.43E-01	1.00E+00	1.00E+00	1.00E+00	small terminal subunit of phenylpropionate dioxygenase
KO:K05710	1.95	0.01	2.06	0.37	0.05	0.00	2.71	1.41E-01	8.05E-01	1.33E-01	5.49E-01	7.65E-01	1.00E+00	9.54E-02	ferredoxin subunit of phenylpropionate dioxygenase
KO:K05711	0.00	0.00	0.00	1.33	0.00	0.00	0.00	1.00E+00	1.00E+00	1.00E+00	1.43E-01	1.00E+00	1.00E+00	1.00E+00	2,3-dihydroxy-2,3-dihydrophenylpropionate dehydrogenase [EC:1.3.1.-]
KO:K05712	0.36	9.57	0.00	1.81	0.00	0.00	0.00	7.15E-01	1.23E-02	1.00E+00	2.54E-01	1.00E+00	1.00E+00	1.00E+00	3-(3-hydroxy-phenyl)propionate hydroxylase [EC:1.14.13.-]
KO:K05713	0.00	0.22	0.00	0.00	0.00	0.00	0.00	1.00E+00	1.03E-01	1.00E+00	1.00E+00	1.00E+00	1.00E+00	1.00E+00	2,3-dihydroxyphenylpropionate 1,2-dioxygenase [EC:1.13.11.16]
KO:K05714	0.32	0.00	0.00	0.00	0.00	5.36	0.00	1.76E-01	1.00E+00	1.00E+00	1.00E+00	1.00E+00	1.47E-02	1.00E+00	2-hydroxy-6-ketonona-2,4-dienedioic acid hydrolase [EC:3.7.1.-]
KO:K05770	0.00	0.11	0.00	0.00	0.00	0.00	0.00	1.00E+00	1.04E-01	1.00E+00	1.00E+00	1.00E+00	1.00E+00	1.00E+00	benzodiazapine receptor
KO:K05772	0.28	11.69	0.01	2.29	0.00	0.29	0.00	8.08E-01	9.14E-03	8.90E-01	2.54E-01	1.00E+00	8.04E-01	1.00E+00	putative tungstate transport system substrate-binding protein
KO:K05773	0.11	12.18	0.00	1.36	0.00	0.35	0.00	8.35E-01	8.57E-03	1.00E+00	3.64E-01	1.00E+00	7.38E-01	1.00E+00	putative tungstate transport system permease protein
KO:K05774	0.00	1.59	0.00	0.02	0.00	0.00	0.00	1.00E+00	1.60E-02	1.00E+00	5.82E-01	1.00E+00	1.00E+00	1.00E+00	ribose 1,5-bisphosphokinase [EC:2.7.4.23]
KO:K05776	3.88	0.36	0.32	0.00	0.00	0.00	1.50	5.26E-02	4.89E-01	5.11E-01	7.46E-01	1.00E+00	1.00E+00	1.82E-01	molybdate transport system ATP-binding protein
KO:K05777	0.57	0.45	0.00	4.61	0.00	0.17	0.00	4.13E-01	4.69E-01	1.00E+00	4.53E-02	1.00E+00	6.41E-01	1.00E+00	putative thiamine transport system substrate-binding protein
KO:K05778	1.04	0.46	0.00	0.87	0.00	0.00	0.00	1.30E-01	2.74E-01	1.00E+00	1.62E-01	1.00E+00	1.00E+00	1.00E+00	putative thiamine transport system permease protein
KO:K05779	1.23	1.95	0.00	0.01	0.00	0.00	0.00	1.30E-01	8.63E-02	1.00E+00	6.24E-01	1.00E+00	1.00E+00	1.00E+00	putative thiamine transport system ATP-binding protein
KO:K05780	0.06	1.04	0.00	0.02	0.00	0.00	0.00	4.49E-01	2.67E-02	1.00E+00	5.64E-01	1.00E+00	1.00E+00	1.00E+00	putative phosphonate transport system ATP-binding protein
KO:K05781	0.00	3.07	0.00	0.02	0.00	0.00	0.00	7.07E-01	1.23E-02	1.00E+00	6.61E-01	1.00E+00	1.00E+00	1.00E+00	putative phosphonate transport system ATP-binding protein
KO:K05782	0.00	0.12	0.00	0.90	0.00	0.00	0.00	1.00E+00	2.13E-01	1.00E+00	9.99E-02	1.00E+00	1.00E+00	1.00E+00	benzoate membrane transport protein
KO:K05783	0.70	0.00	0.00	0.00	0.00	0.00	0.00	3.76E-02	1.00E+00	1.00E+00	1.00E+00	1.00E+00	1.00E+00	1.00E+00	1,6-dihydroxycyclohexa-2,4-diene-1-carboxylate dehydrogenase
KO:K05784	0.00	0.00	0.00	0.20	0.00	0.00	0.00	1.00E+00	1.00E+00	1.00E+00	1.43E-01	1.00E+00	1.00E+00	1.00E+00	benzoate 1,2-dioxygenase electron transfer component
KO:K05785	3.21	0.70	0.12	0.14	0.64	0.09	0.99	9.66E-02	4.26E-01	7.10E-01	6.97E-01	4.47E-01	7.26E-01	3.42E-01	transcriptional antiterminator RfaH
KO:K05786	2.57	8.69	1.06	1.15	0.00	0.00	0.00	2.33E-01	2.89E-02	5.95E-01	5.69E-01	1.00E+00	1.00E+00	1.00E+00	chloramphenicol-sensitive protein RarD
KO:K05787	0.39	0.16	0.00	0.18	0.00	0.00	0.06	1.64E-01	2.76E-01	1.00E+00	2.61E-01	1.00E+00	1.00E+00	3.94E-01	DNA-binding protein HU-alpha
KO:K05788	8.74	6.20	1.80	4.04	0.06	5.07	1.67	7.69E-02	1.92E-01	8.57E-01	4.84E-01	9.83E-01	3.10E-01	8.73E-01	integration host factor subunit beta
KO:K05791	0.04	0.34	0.00	0.17	0.00	0.00	0.00	3.25E-01	1.40E-01	1.00E+00	2.07E-01	1.00E+00	1.00E+00	1.00E+00	tellurium resistance protein TerZ
KO:K05793	0.06	0.00	0.00	0.05	0.00	0.00	0.00	1.02E-01	1.00E+00	1.00E+00	2.13E-01	1.00E+00	1.00E+00	1.00E+00	tellurite resistance protein TerB
KO:K05794	0.22	0.02	0.39	0.41	6.37	0.00	0.00	4.57E-01	6.95E-01	3.42E-01	3.35E-01	1.71E-02	1.00E+00	1.00E+00	tellurite resistance protein TerC
KO:K05795	1.01	0.01	0.00	0.91	0.00	0.00	0.00	1.15E-01	5.66E-01	1.00E+00	1.30E-01	1.00E+00	1.00E+00	1.00E+00	tellurium resistance protein TerD
KO:K05797	0.06	0.00	0.00	0.05	0.00	0.00	0.00	1.10E-01	1.00E+00	1.00E+00	2.09E-01	1.00E+00	1.00E+00	1.00E+00	4-cresol dehydrogenase (hydroxylating) [EC:1.17.99.1]
KO:K05799	0.63	7.04	0.00	0.01	0.00	18.03	0.00	6.32E-01	3.21E-02	1.00E+00	9.29E-01	1.00E+00	2.71E-03	9.32E-01	GntR family transcriptional regulator, transcriptional repressor for
KO:K05800	1.10	14.16	0.00	1.37	0.00	0.00	0.00	5.92E-01	4.00E-03	1.00E+00	4.87E-01	1.00E+00	1.00E+00	9.53E-01	Lrp/AsnC family transcriptional regulator
KO:K05801	5.02	11.04	0.12	2.00	0.00	1.67	0.17	1.26E-01	1.59E-02	9.65E-01	6.22E-01	1.00E+00	7.09E-01	9.61E-01	DnaJ like chaperone protein
KO:K05802	0.49	0.11	1.74	0.21	0.00	0.25	0.00	2.45E-01	4.85E-01	7.30E-02	3.92E-01	1.00E+00	3.59E-01	1.00E+00	potassium efflux system protein KefA
KO:K05803	0.23	0.00	0.00	0.00	0.00	0.00	0.00	5.46E-02	1.00E+00	1.00E+00	1.00E+00	1.00E+00	1.00E+00	1.00E+00	lipoprotein NlpI
KO:K05805	0.00	0.00	0.00	0.10	0.00	0.00	0.00	1.00E+00	1.00E+00	1.00E+00	1.43E-01	1.00E+00	1.00E+00	1.00E+00	CreA protein
KO:K05807	11.36	10.52	1.90	5.96	0.00	0.18	0.85	3.07E-02	3.83E-02	8.94E-01	2.23E-01	1.00E+00	9.91E-01	9.63E-01	putative lipoprotein
KO:K05808	1.18	0.11	13.26	0.58	8.89	0.21	2.58	6.12E-01	9.29E-01	4.57E-03	8.18E-01	1.67E-02	9.01E-01	2.40E-01	putative sigma-54 modulation protein
KO:K05810	1.81	3.05	0.00	1.43	5.42	0.21	0.03	2.92E-01	9.93E-02	1.00E+00	4.29E-01	2.30E-02	9.20E-01	9.80E-01	conserved hypothetical protein
KO:K05812	0.81	0.00	0.00	0.00	0.00	0.00	0.00	3.69E-02	1.00E+00	1.00E+00	1.00E+00	1.00E+00	1.00E+00	1.00E+00	conserved hypothetical protein
KO:K05813	0.51	37.65	0.00	3.94	0.00	0.00	0.00	8.74E-01	1.14E-03	1.00E+00	3.59E-01	1.00E+00	1.00E+00	1.00E+00	sn-glycerol 3-phosphate transport system substrate-binding protein
KO:K05814	0.66	20.01	0.00	3.11	0.00	0.00	0.00	7.65E-01	6.57E-03	1.00E+00	2.78E-01	1.00E+00	1.00E+00	1.00E+00	sn-glycerol 3-phosphate transport system permease protein
KO:K05815	0.39	16.49	0.00	2.94	0.00	0.00	0.00	7.93E-01	8.00E-03	1.00E+00	2.34E-01	1.00E+00	1.00E+00	1.00E+00	sn-glycerol 3-phosphate transport system permease protein
KO:K05816	0.70	15.06	0.01	1.03	0.00	0.00	0.00	6.48E-01	6.29E-03	9.08E-01	5.37E-01	1.00E+00	1.00E+00	1.00E+00	sn-glycerol 3-phosphate transport system ATP-binding protein
KO:K05817	0.00	0.44	0.00	0.00	0.00	0.00	0.00	1.00E+00	6.97E-02	1.00E+00	1.00E+00	1.00E+00	1.00E+00	1.00E+00	LysR family transcriptional regulator, hca operon transcriptional
KO:K05818	0.00	0.01	0.00	0.92	0.00	0.00	0.00	1.00E+00	3.64E-01	1.00E+00	5.74E-02	1.00E+00	1.00E+00	1.00E+00	IclR family transcriptional regulator, mhp operon transcriptional
KO:K05820	0.59	0.00	0.12	1.51	0.00	6.28	0.22	3.86E-01	1.00E+00	7.08E-01	1.45E-01	1.00E+00	1.37E-02	6.34E-01	MFS transporter, PPP family, 3-phenylpropionic acid transporter
KO:K05826	0.00	0.00	0.00	0.00	0.00	0.00	3.75	1.00E+00	1.00E+00	1.00E+00	1.00E+00	1.00E+00	1.00E+00	1.79E-02	lysine biosynthesis protein LysW
KO:K05827	0.00	0.00	0.00	0.00	0.00	0.00	5.83	1.00E+00	1.00E+00	1.00E+00	1.00E+00	1.00E+00	1.00E+00	1.50E-02	lysine biosynthesis protein LysX
KO:K05830	0.00	0.00	0.00	1.63	0.00	0.00	0.00	1.00E+00	1.00E+00	1.00E+00	7.83E-02	1.00E+00	1.00E+00	1.00E+00	acetylornithine/acetyl-lysine aminotransferase [EC:2.6.1.11 2.6.1.-]
KO:K05831	0.00	0.00	0.00	0.00	0.00	0.00	3.33	1.00E+00	1.00E+00	1.00E+00	1.00E+00	1.00E+00	1.00E+00	5.00E-03	acetyl-lysine deacetylase [EC:3.5.1.-]
KO:K05832	0.16	0.04	0.00	0.00	0.00	0.00	0.00	9.99E-02	2.13E-01	1.00E+00	1.00E+00	1.00E+00	1.00E+00	1.00E+00	putative ABC transport system permease protein
KO:K05834	0.03	0.74	0.00	0.22	0.00	0.00	0.18	6.15E-01	8.70E-02	1.00E+00	2.91E-01	1.00E+00	1.00E+00	3.32E-01	homoserine/homoserine lactone efflux protein
KO:K05835	0.21	0.09	0.00	0.00	0.00	0.00	0.00	1.12E-01	1.93E-01	1.00E+00	1.00E+00	1.00E+00	1.00E+00	1.00E+00	threonine efflux protein
KO:K05836	0.62	0.88	0.00	0.00	0.00	0.00	0.00	9.64E-02	6.76E-02	1.00E+00	7.01E-01	1.00E+00	1.00E+00	1.00E+00	GntR family transcriptional regulator, histidine utilization
KO:K05837	5.41	4.23	9.76	3.86	5.39	3.66	1.20	2.79E-01	5.39E-01	3.16E-02	6.33E-01	2.81E-01	6.83E-01	9.66E-01	rod shape determining protein RodA
KO:K05838	3.49	16.55	0.19	2.09	0.00	4.67	0.00	3.76E-01	7.43E-03	9.54E-01	6.76E-01	1.00E+00	2.08E-01	9.80E-01	putative thioredoxin
KO:K05841	0.00	0.00	0.38	0.16	0.00	0.00	0.00	1.00E+00	1.00E+00	4.27E-02	1.40E-01	1.00E+00	1.00E+00	1.00E+00	sterol 3beta-glucosyltransferase [EC:2.4.1.173]

KO:K05844	5.32	3.31	7.60	3.36	0.13	0.23	0.27	1.09E-01	3.23E-01	4.61E-02	3.15E-01	9.67E-01	9.53E-01	9.50E-01	ribosomal protein S6 modification protein
KO:K05845	0.00	0.11	0.00	0.01	0.87	0.00	0.00	1.00E+00	2.02E-01	1.00E+00	4.39E-01	2.44E-02	1.00E+00	1.00E+00	osmoprotectant transport system substrate-binding protein
KO:K05846	0.93	0.00	0.00	0.62	0.00	0.00	0.00	1.21E-01	4.63E-01	1.00E+00	1.59E-01	1.00E+00	1.00E+00	1.00E+00	osmoprotectant transport system permease protein
KO:K05847	0.16	0.10	0.00	1.01	0.00	0.00	0.00	2.96E-01	3.48E-01	1.00E+00	7.60E-02	1.00E+00	1.00E+00	1.00E+00	osmoprotectant transport system ATP-binding protein
KO:K05851	0.34	0.00	0.00	0.17	0.00	0.00	0.00	7.47E-02	1.00E+00	1.00E+00	1.79E-01	1.00E+00	1.00E+00	1.00E+00	adenylate cyclase, class 1 [EC:4.6.1.1]
KO:K05873	0.00	0.00	8.13	0.00	0.00	0.00	0.00	1.00E+00	1.00E+00	4.00E-03	1.00E+00	1.00E+00	1.00E+00	1.00E+00	adenylate cyclase, class 2 [EC:4.6.1.1]
KO:K05874	0.06	0.05	0.00	0.00	0.00	0.00	0.00	1.22E-01	1.65E-01	1.00E+00	1.00E+00	1.00E+00	1.00E+00	1.00E+00	methyl-accepting chemotaxis protein I, serine sensor receptor
KO:K05878	0.91	0.00	0.00	2.82	0.00	0.00	0.28	2.21E-01	1.00E+00	1.00E+00	8.96E-02	1.00E+00	1.00E+00	3.98E-01	dihydroxyacetone kinase, N-terminal domain [EC:2.7.1.-]
KO:K05879	0.06	2.26	0.00	1.29	0.00	0.00	0.00	4.86E-01	1.09E-01	1.00E+00	1.66E-01	1.00E+00	1.00E+00	1.00E+00	dihydroxyacetone kinase, C-terminal domain [EC:2.7.1.-]
KO:K05882	0.71	0.41	0.00	1.28	0.00	0.00	0.00	2.21E-01	2.88E-01	1.00E+00	1.55E-01	1.00E+00	1.00E+00	1.00E+00	aryl-alcohol dehydrogenase (NADP+) [EC:1.1.1.91]
KO:K05883	0.00	0.00	0.00	0.10	0.00	0.00	0.00	1.00E+00	1.00E+00	1.00E+00	1.43E-01	1.00E+00	1.00E+00	1.00E+00	
KO:K05884	0.00	0.00	20.09	0.07	0.00	0.00	0.00	1.00E+00	1.00E+00	4.57E-03	3.31E-01	1.00E+00	1.00E+00	1.00E+00	(R)-2-hydroxyacid dehydrogenase [EC:1.1.1.272]
KO:K05885	0.00	0.00	0.00	1.09	0.00	0.00	0.73	1.00E+00	1.00E+00	1.00E+00	6.83E-02	1.00E+00	1.00E+00	1.03E-01	2,5-diketo-D-gluconate reductase [EC:1.1.1.274]
KO:K05886	0.00	0.00	0.00	1.12	0.00	0.00	0.00	1.00E+00	1.00E+00	1.00E+00	5.66E-02	1.00E+00	1.00E+00	1.00E+00	serine 3-dehydrogenase [EC:1.1.1.276]
KO:K05888	0.00	0.00	0.00	0.61	0.00	0.00	0.00	1.00E+00	1.00E+00	1.00E+00	1.43E-01	1.00E+00	1.00E+00	1.00E+00	
KO:K05889	8.32	0.00	0.00	0.63	0.00	0.00	0.00	3.67E-02	1.00E+00	1.00E+00	3.45E-01	1.00E+00	1.00E+00	1.00E+00	polyvinyl alcohol dehydrogenase (cytochrome) [EC:1.1.2.6]
KO:K05895	0.01	4.42	0.00	0.01	5.33	0.00	0.00	8.66E-01	2.01E-02	1.00E+00	8.70E-01	1.47E-02	1.00E+00	1.00E+00	precorrin-6X reductase [EC:1.3.1.54]
KO:K05896	6.62	2.10	5.86	3.68	3.34	2.50	0.79	1.05E-01	7.77E-01	1.46E-01	4.32E-01	5.05E-01	6.96E-01	9.35E-01	segregation and condensation protein A
KO:K05898	5.61	0.11	0.00	0.02	0.00	0.00	0.00	5.81E-02	3.87E-01	1.00E+00	4.58E-01	1.00E+00	1.00E+00	1.00E+00	3-oxosteroid 1-dehydrogenase [EC:1.3.99.4]
KO:K05903	4.11	1.98	0.04	2.42	0.00	0.64	0.00	1.16E-01	2.91E-01	8.09E-01	2.30E-01	1.00E+00	6.19E-01	8.46E-01	NADH dehydrogenase (quinone) [EC:1.6.99.5]
KO:K05905	0.70	0.27	0.00	1.15	0.00	22.96	0.00	5.04E-01	6.64E-01	1.00E+00	3.86E-01	1.00E+00	2.57E-03	1.00E+00	protein-disulfide reductase [EC:1.8.1.8]
KO:K05910	0.00	0.00	1.88	0.00	0.00	0.00	0.00	1.00E+00	1.00E+00	3.00E-03	1.00E+00	1.00E+00	1.00E+00	1.00E+00	NADH peroxidase [EC:1.11.1.1]
KO:K05911	0.00	2.00	0.00	0.00	0.00	0.00	0.00	1.00E+00	1.04E-01	1.00E+00	1.00E+00	1.00E+00	1.00E+00	1.00E+00	quinone-reactive Ni/Fe-hydrogenase [EC:1.12.5.1]
KO:K05912	0.00	0.00	0.00	0.00	36.88	0.00	0.00	1.00E+00	1.00E+00	1.00E+00	1.00E+00	1.43E-04	1.00E+00	1.00E+00	
KO:K05915	0.01	14.99	0.00	0.12	0.00	0.00	0.00	8.18E-01	5.71E-03	1.00E+00	7.42E-01	1.00E+00	1.00E+00	1.00E+00	
KO:K05916	0.01	0.26	0.00	0.15	0.00	0.00	0.10	5.53E-01	1.33E-01	1.00E+00	2.23E-01	1.00E+00	1.00E+00	2.97E-01	nitric oxide dioxygenase [EC:1.14.12.17]
KO:K05917	0.00	0.00	0.00	0.10	0.00	0.00	0.00	1.00E+00	1.00E+00	1.00E+00	1.43E-01	1.00E+00	1.00E+00	1.00E+00	cytochrome P45, family 51 (sterol 14-demethylase) [EC:1.14.13.7]
KO:K05918	3.00	0.00	1.93	0.00	0.00	0.00	0.00	6.96E-02	1.00E+00	1.15E-01	4.82E-01	1.00E+00	1.00E+00	1.00E+00	
KO:K05922	0.47	0.00	0.00	0.00	0.00	0.00	0.00	3.80E-02	1.00E+00	1.00E+00	1.00E+00	1.00E+00	1.00E+00	1.00E+00	quinone-reactive Ni/Fe-hydrogenase large subunit [EC:1.12.5.1]
KO:K05927	0.81	0.00	0.00	0.00	0.00	0.00	0.00	6.53E-02	1.00E+00	1.00E+00	1.00E+00	1.00E+00	1.00E+00	1.00E+00	quinone-reactive Ni/Fe-hydrogenase small subunit [EC:1.12.5.1]
KO:K05934	0.00	3.57	0.00	0.29	1.01	0.00	0.74	8.50E-01	3.63E-02	1.00E+00	5.73E-01	2.28E-01	1.00E+00	3.16E-01	precorrin-3B C17-methyltransferase [EC:2.1.1.131]
KO:K05936	0.41	1.33	0.00	0.30	11.20	0.00	0.22	6.22E-01	2.28E-01	1.00E+00	7.02E-01	4.29E-03	1.00E+00	7.55E-01	precorrin-4 C11-methyltransferase [EC:2.1.1.133]
KO:K05939	0.03	0.02	32.42	0.10	0.37	0.00	1.73	7.20E-01	7.25E-01	1.43E-04	6.96E-01	5.75E-01	1.00E+00	1.97E-01	acyl-[acyl-carrier-protein]-phospholipid O-acyltransferase /
KO:K05942	0.00	0.00	0.63	0.00	0.00	0.00	0.00	1.00E+00	1.00E+00	1.57E-02	1.00E+00	1.00E+00	1.00E+00	1.00E+00	
KO:K05945	0.00	0.33	0.00	0.00	0.00	0.00	0.00	1.00E+00	1.00E-01	1.00E+00	1.00E+00	1.00E+00	1.00E+00	1.00E+00	
KO:K05946	0.05	0.00	0.12	0.24	6.00	0.00	0.00	4.90E-01	6.56E-01	3.89E-01	3.13E-01	1.24E-02	1.00E+00	1.00E+00	N-acetylglucosaminyldiphosphoundecaprenol [EC:2.4.1.187]
KO:K05952	7.03	0.39	2.10	0.21	0.00	0.00	0.00	2.61E-02	5.96E-01	1.65E-01	7.00E-01	1.00E+00	1.00E+00	1.00E+00	hypothetical protein
KO:K05962	0.27	0.00	0.00	0.00	0.00	1.12	0.00	1.61E-01	1.00E+00	1.00E+00	1.00E+00	1.00E+00	3.24E-02	1.00E+00	protein-histidine pros-kinase [EC:2.7.13.1]
KO:K05966	0.00	0.00	1.13	0.00	0.50	0.00	0.00	1.00E+00	1.00E+00	9.00E-03	1.00E+00	5.93E-02	1.00E+00	1.00E+00	triphosphoribosyl-dephospho-CoA synthase [EC:2.7.8.25]
KO:K05970	0.32	0.00	23.00	0.23	0.56	0.00	4.54	6.05E-01	1.00E+00	4.29E-03	6.51E-01	5.13E-01	1.00E+00	8.57E-02	sialate O-acetylesterase [EC:3.1.1.53]
KO:K05971	0.00	0.00	0.54	0.01	11.34	0.00	0.01	1.00E+00	1.00E+00	1.69E-01	5.25E-01	1.43E-04	1.00E+00	5.13E-01	
KO:K05973	0.47	40.87	0.00	0.84	0.00	0.00	0.00	8.75E-01	2.86E-04	1.00E+00	8.27E-01	1.00E+00	1.00E+00	1.00E+00	poly(3-hydroxybutyrate) depolymerase [EC:3.1.1.75]
KO:K05978	0.00	0.00	0.00	0.00	11.88	0.00	0.00	1.00E+00	1.00E+00	1.00E+00	1.00E+00	1.43E-04	1.00E+00	1.00E+00	glucosylglycerol 3-phosphatase [EC:3.1.3.69]
KO:K05979	0.00	0.00	0.06	0.04	19.45	0.00	0.00	6.93E-01	7.03E-01	5.67E-01	6.11E-01	4.29E-04	1.00E+00	1.00E+00	2-phosphosulfolactate phosphatase [EC:3.1.3.71]
KO:K05984	1.98	0.00	0.00	0.00	0.00	0.00	0.00	3.06E-02	1.00E+00	1.00E+00	1.00E+00	1.00E+00	1.00E+00	1.00E+00	excinuclease Cho [EC:3.1.25.-]
KO:K05986	2.40	0.00	0.00	0.11	0.00	0.00	0.00	4.86E-02	1.00E+00	1.00E+00	3.46E-01	1.00E+00	1.00E+00	1.00E+00	
KO:K05987	0.00	1.78	0.00	0.00	0.00	0.00	0.00	1.00E+00	1.01E-01	1.00E+00	1.00E+00	1.00E+00	1.00E+00	1.00E+00	
KO:K05989	0.08	0.35	3.79	0.15	0.00	0.00	0.00	4.43E-01	2.88E-01	2.94E-02	3.93E-01	1.00E+00	1.00E+00	1.00E+00	alpha-L-rhamnosidase [EC:3.2.1.4]
KO:K05993	0.07	1.22	0.06	0.99	0.00	0.00	0.00	4.86E-01	1.24E-01	5.02E-01	1.54E-01	1.00E+00	1.00E+00	1.00E+00	isochorismatase [EC:3.3.2.1]
KO:K05994	0.00	0.00	0.00	0.51	0.00	0.00	0.00	1.00E+00	1.00E+00	1.00E+00	4.30E-02	1.00E+00	1.00E+00	1.00E+00	bacterial leucyl aminopeptidase [EC:3.4.11.1]
KO:K05995	0.89	0.01	0.26	0.06	0.00	0.46	0.00	7.29E-02	6.91E-01	2.70E-01	5.48E-01	1.00E+00	1.51E-01	1.00E+00	dipeptidase E [EC:3.4.13.21]
KO:K05997	4.15	0.00	0.00	0.01	0.00	0.00	0.90	3.21E-02	1.00E+00	1.00E+00	6.09E-01	1.00E+00	1.00E+00	1.90E-01	Fe-S cluster assembly protein SufA
KO:K06001	4.14	0.00	0.07	4.10	0.00	0.00	0.13	9.50E-02	1.00E+00	6.45E-01	9.70E-02	1.00E+00	1.00E+00	6.12E-01	tryptophan synthase beta chain [EC:4.2.1.2]
KO:K06013	0.34	0.07	10.44	0.95	0.69	0.20	1.48	6.80E-01	8.04E-01	9.86E-03	4.17E-01	5.16E-01	7.51E-01	2.74E-01	STE24 endopeptidase [EC:3.4.24.84]
KO:K06015	0.59	2.77	2.13	1.51	0.00	0.00	0.00	5.05E-01	1.38E-01	1.90E-01	2.72E-01	1.00E+00	1.00E+00	1.00E+00	
KO:K06016	0.65	14.22	1.61	0.71	5.47	0.00	0.00	8.32E-01	7.71E-03	5.92E-01	8.17E-01	9.13E-02	1.00E+00	1.00E+00	N-carbamoyl-L-amino-acid hydrolase [EC:3.5.1.87]
KO:K06017	0.00	0.00	1.25	0.00	0.00	0.00	0.00	1.00E+00	1.00E+00	1.61E-02	1.00E+00	1.00E+00	1.00E+00	1.00E+00	
KO:K06020	0.07	0.05	0.00	1.54	0.00	0.00	0.67	4.19E-01	4.53E-01	1.00E+00	9.60E-02	1.00E+00	1.00E+00	2.08E-01	sulfate-transporting ATPase [EC:3.6.3.25]
KO:K06021	0.03	0.00	0.00	0.32	0.00	0.00	0.00	2.67E-01	1.00E+00	1.00E+00	8.74E-02	1.00E+00	1.00E+00	1.00E+00	phosphate-transporting ATPase [EC:3.6.3.27]
KO:K06022	0.00	0.00	0.00	2.14	0.00	0.00	0.00	1.00E+00	1.00E+00	1.00E+00	8.27E-02	1.00E+00	1.00E+00	1.00E+00	molybdate-transporting ATPase [EC:3.6.3.29]
KO:K06023	0.09	0.25	10.29	0.11	2.26	0.00	0.16	6.74E-01	5.34E-01	1.30E-02	6.52E-01	1.13E-01	1.00E+00	6.03E-01	HPr kinase/phosphorylase [EC:2.7.11.- 2.7.4.-]
KO:K06024	7.61	1.13	2.41	2.01	4.05	1.15	0.99	4.27E-02	8.35E-01	5.29E-01	6.36E-01	2.08E-01	8.30E-01	8.62E-01	segregation and condensation protein B
KO:K06027	0.00	0.00	0.44	0.02	0.44	0.00	8.04	1.00E+00	1.00E+00	2.55E-01	4.52E-01	2.55E-01	1.00E+00	8.71E-03	vesicle-fusing ATPase [EC:3.6.4.6]
KO:K06031	0.06	0.00	9.56	0.03	4.66	0.00	2.14	5.52E-01	1.00E+00	2.10E-02	5.77E-01	6.89E-02	1.00E+00	1.60E-01	
KO:K06034	0.76	6.23	0.00	1.59	0.00	0.00	0.47	4.81E-01	5.69E-02	1.00E+00	3.14E-01	1.00E+00	1.00E+00	5.80E-01	sulfopyruvate decarboxylase subunit alpha [EC:4.1.1.79]
KO:K06036	0.12	0.00	0.00	0.00	0.00	0.00	0.00	6.26E-02	1.00E+00	1.00E+00	1.00E+00	1.00E+00	1.00E+00	1.00E+00	
KO:K06041	6.95	3.27	2.37	3.02	5.28	5.51	2.51	1.04E-01	6.78E-01	8.45E-01	7.32E-01	2.41E-01	2.11E-01	8.24E-01	arabinose-5-phosphate isomerase [EC:5.3.1.13]
KO:K06042	0.22	3.28	0.00	0.12	4.34	0.00	0.48	6.80E-01	6.69E-02	1.00E+00	7.62E-01	4.23E-02	1.00E+00	5.13E-01	precorrin-8X methylmutase [EC:5.4.1.2]
KO:K06045	0.09	0.00	10.48	0.27	4.61	0.00	1.70	6.24E-01	1.00E+00	2.07E-02	5.32E-01	6.41E-02	1.00E+00	2.12E-01	squalene-hopene cyclase [EC:5.4.99.17]
KO:K06048	0.06	0.22	0.28	0.90	0.00	0.00	0.03	6.02E-01	3.61E-01	3.01E-01	9.11E-02	1.00E+00	1.00E+00	6.86E-01	carboxylate-amine ligase [EC:6.3.-.-]
KO:K06049	0.46	6.90	0.00	0.01	0.00	0.00	0.00	4.09E-01	1.81E-02	1.00E+00	7.37E-01	1.00E+00	1.00E+00	1.00E+00	magnesium chelatase accessory protein
KO:K06074	0.23	0.00	0.00	0.00	0.00	0.00	0.00	6.14E-02	1.00E+00	1.00E+00	1.00E+00	1.00E+00	1.00E+00	1.00E+00	vitamin B12 transport system ATP-binding protein [EC:3.6.3.33]
KO:K06075	0.02	0.09	1.04	0.15	0.00	0.00	0.00	5.36E-01	3.37E-01	3.27E-02	2.52E-01	1.00E+00	1.00E+00	1.00E+00	MarR family transcriptional regulator, transcriptional regulator for
KO:K06076	5.86	0.22	1.39	1.07	0.00	0.07	0.73	4.59E-02	7.41E-01	3.53E-01	4.27E-01	1.00E+00	8.22E-01	5.35E-01	long-chain fatty acid transport protein
KO:K06077	0.06	0.41	0.45	0.23	0.00	0.00	0.56	4.72E-01	2.53E-01	2.37E-01	3.29E-01	1.00E+00	1.00E+00	2.08E-01	outer membrane lipoprotein SlyB
KO:K06113	0.23	0.00	0.00	0.00	0.00	0.00	0.00	6.19E-02	1.00E+00	1.00E+00	1.00E+00	1.00E+00	1.00E+00	1.00E+00	arabinan endo-1,5-alpha-L-arabinosidase [EC:3.2.1.99]
KO:K06118	0.34	0.03	0.00	0.06	33.19	0.00	0.00	4.88E-01	7.22E-01	1.00E+00	6.86E-01	2.86E-04	1.00E+00	1.00E+00	UDP-sulfoquinovose synthase [EC:3.13.1.1]
KO:K06126	1.40	0.00	0.00	0.00	0.00	0.00	0.00	6.64E-02	1.00E+00	1.00E+00	1.00E+00	1.00E+00	1.00E+00	1.00E+00	ubiquinone biosynthesis monooxygenase Coq6 [EC:1.14.13.-]
KO:K06127	0.00	0.00	0.00	1.12	0.00	0.00	0.00	1.00E+00	1.00E+00	1.00E+00	1.43E-01	1.00E+00	1.00E+00	1.00E+00	ubiquinone biosynthesis methyltransferase [EC:2.1.1.-]
KO:K06131	0.07	0.01	2.70	0.16	0.04	0.00	0.17	6.17E-01	7.95E-01	2.00E-03	4.21E-01	7.21E-01	1.00E+00	4.08E-01	cardiolipin synthase [EC:2.7.8.-]
KO:K06132	0.02	0.00	0.00	0.00	0.53	0.00	0.00	2.35E-01	1.00E+00	1.00E+00	1.00E+00	2.83E-02	1.00E+00	1.00E+00	putative cardiolipin synthase [EC:2.7.8.-]
KO:K06133	0.14	0.00	0.00	0.16	0.24	0.00	0.00	2.08E-01	1.00E+00	1.00E+00	1.83E-01	1.04E-01	1.00E+00	1.00E+00	4'-phosphopantetheinyl transferase [EC:2.7.8.-]
KO:K06134	9.88	0.04	0.01	4.45	0.00	0.00	1.98	1.90E-02	9.07E-01	9.17E-01	1.04E-01	1.00E+00	1.00E+00	3.64E-01	ubiquinone biosynthesis monooxygenase Coq7 [EC:1.14.13.-]
KO:K06135	0.23	0.00	0.00	0.00	0.00	0.00	0.00	6.26E-02	1.00E+00	1.00E+00	1.00E+00	1.00E+00	1.00E+00	1.00E+00	pyrroloquinoline quinone biosynthesis protein A
KO:K06136	3.50	1.58	0.00	5.00	0.00	0.00	0.09	1.32E-01	3.53E-01	1.00E+00	7.66E-02	1.00E+00	1.00E+00	8.32E-01	pyrroloquinoline quinone biosynthesis protein B
KO:K06137	3.48	0.11	0.00	4.07	0.00	0.00	0.81	1.04E-01	7.51E-01	1.00E+00	8.29E-02	1.00E+00	1.00E+00	4.65E-01	pyrroloquinoline-quinone synthase [EC:1.3.3.11]
KO:K06138	0.44	0.03	0.00	2.62	0.00	0.00	0.00	2.59E-01	5.75E-01	1.00E+00	5.94E-02	1.00E+00	1.00E+00	1.00E+00	pyrroloquinoline quinone biosynthesis protein D
KO:K06139	0.79	0.62	0.00	2.94	0.00	0.00	0.00	2.60E-01	3.24E-01	1.00E+00	6.71E-02	1.00E+00	1.00E+00	1.00E+00	pyrroloquinoline quinone biosynthesis protein E
KO:K06140	0.00	0.00	0.63	0.00	0.00	0.00	0.00	1.00E+00	1.00E+00	1.33E-02	1.00E+00	1.00E+00	1.00E+00	1.00E+00	regulator of nucleoside diphosphate kinase
KO:K06142	9.73	0.53	0.06	2.26	0.00	10.68	1.98	3.36E-02	8.70E-01	9.43E-01	5.08E-01	1.00E+00	2.73E-02	5.75E-01	outer membrane protein
KO:K06143	0.04	0.02	1.29	0.26	0.00	0.00	0.04	4.39E-01	4.97E-01	4.66E-02	2.24E-01	1.00E+00	1.00E+00	4.62E-01	inner membrane protein
KO:K06145	0.01	25.73	0.00	0.04	0.00	0.00	0.00	8.79E-01	8.57E-04	1.00E+00	8.54E-01	1.00E+00	1.00E+00	1.00E+00	LacI family transcriptional regulator, gluconate utilization system
KO:K06147	6.33	20.85	1.37	5.40	21.03	2.49	0.54	6.89E-01	9.71E-03	9.81E-01	8.00E-01	9.29E-03	9.57E-01	9.94E-01	ATP-binding cassette, subfamily B, bacterial
KO:K06148	2.19	6.15	0.74	1.78	0.05	2.12	0.00	3.35E-01	5.91E-02	7.52E-01	4.34E-01	9.42E-01	3.53E-01	9.72E-01	ATP-binding cassette, subfamily C, bacterial
KO:K06149	8.36	0.17	0.00	0.02	0.00	0.00	0.00	1.33E-02	5.63E-01	1.00E+00	7.25E-01	1.00E+00	1.00E+00	1.00E+00	universal stress protein A
KO:K06151	0.00	0.11	0.00	0.00	0.00	0.00	0.00	1.00E+00	1.02E-01	1.00E+00	1.00E+00	1.00E+00	1.00E+00	1.00E+00	gluconate 2-dehydrogenase alpha chain [EC:1.1.99.3]

KO:K06152	0.00	0.00	0.00	0.61	0.00	0.00	0.00	1.00E+00	1.00E+00	1.00E+00	1.43E-01	1.00E+00	1.00E+00	1.00E+00	gluconate 2-dehydrogenase gamma chain [EC:1.1.99.3]
KO:K06153	4.56	4.06	10.83	2.96	5.35	3.60	0.46	3.44E-01	4.54E-01	2.23E-02	7.12E-01	2.23E-01	5.61E-01	9.79E-01	undecaprenyl-diphosphatase [EC:3.6.1.27]
KO:K06154	0.00	0.00	0.00	0.00	0.00	0.00	1.25	1.00E+00	1.00E+00	1.00E+00	1.00E+00	1.00E+00	1.00E+00	2.41E-02	Lrp/AsnC family transcriptional regulator, involved in the
KO:K06155	0.00	1.01	0.00	1.91	0.00	0.00	0.00	4.64E-01	1.77E-01	1.00E+00	1.16E-01	1.00E+00	1.00E+00	1.00E+00	Gnt-I system high-affinity gluconate transporter
KO:K06156	0.02	0.00	0.00	0.51	0.00	0.00	0.00	3.07E-01	1.00E+00	1.00E+00	5.53E-02	1.00E+00	1.00E+00	1.00E+00	Gnt-I system low-affinity gluconate transporter
KO:K06158	2.75	6.00	9.79	0.96	7.57	0.24	0.87	6.06E-01	1.01E-01	2.31E-02	9.32E-01	5.49E-02	9.85E-01	9.41E-01	ATP-binding cassette, sub-family F, member 3
KO:K06159	0.23	0.00	0.00	0.00	0.00	0.00	0.00	6.30E-02	1.00E+00	1.00E+00	1.00E+00	1.00E+00	1.00E+00	1.00E+00	putative ATP-binding cassette transporter
KO:K06160	0.14	0.00	1.19	0.07	0.53	0.00	0.94	3.81E-01	1.00E+00	9.64E-02	4.61E-01	1.94E-01	1.00E+00	1.29E-01	putative ATP-binding cassette transporter
KO:K06161	0.00	0.00	1.25	0.00	0.00	0.00	0.00	1.00E+00	1.00E+00	1.80E-02	1.00E+00	1.00E+00	1.00E+00	1.00E+00	putative ATP-binding cassette transporter
KO:K06162	0.01	19.27	0.00	0.27	0.00	0.00	0.00	8.90E-01	8.57E-04	1.00E+00	7.61E-01	1.00E+00	1.00E+00	1.00E+00	PhnM protein
KO:K06163	0.01	1.26	0.00	0.15	0.00	0.00	0.00	7.00E-01	2.31E-02	1.00E+00	3.11E-01	1.00E+00	1.00E+00	1.00E+00	PhnJ protein
KO:K06164	0.01	2.07	0.00	0.29	0.00	0.00	0.00	7.49E-01	1.83E-02	1.00E+00	2.90E-01	1.00E+00	1.00E+00	1.00E+00	PhnI protein
KO:K06165	0.01	1.08	0.00	0.03	0.00	0.00	0.00	5.98E-01	2.70E-02	1.00E+00	5.19E-01	1.00E+00	1.00E+00	1.00E+00	PhnH protein
KO:K06166	0.00	2.70	0.00	0.07	0.00	0.00	0.00	7.65E-01	1.34E-02	1.00E+00	5.70E-01	1.00E+00	1.00E+00	1.00E+00	PhnG protein
KO:K06167	0.90	3.72	2.18	1.85	1.07	1.39	0.29	7.41E-01	1.11E-01	2.94E-01	3.79E-01	6.78E-01	5.47E-01	9.14E-01	PhnP protein
KO:K06168	9.92	8.40	11.47	6.82	1.85	2.17	4.35	1.04E-01	2.09E-01	5.51E-02	4.61E-01	9.86E-01	9.78E-01	8.80E-01	bifunctional enzyme involved in thiolation and methylation of tRNA
KO:K06169	7.66	0.92	1.41	0.56	3.03	0.04	0.00	2.60E-02	6.46E-01	4.78E-01	7.70E-01	1.75E-01	9.03E-01	9.33E-01	tRNA-(ms[2]io[6]A)-hydroxylase [EC:1.-.-.-]
KO:K06173	3.41	4.41	6.72	2.29	2.56	0.35	1.14	3.24E-01	1.63E-01	4.97E-02	6.50E-01	5.72E-01	9.78E-01	8.98E-01	tRNA pseudouridine synthase A [EC:5.4.99.12]
KO:K06174	0.00	0.00	0.00	0.00	0.00	0.00	3.33	1.00E+00	1.00E+00	1.00E+00	1.00E+00	1.00E+00	1.00E+00	1.47E-02	ATP-binding cassette, sub-family E, member 1
KO:K06175	0.24	0.08	9.22	0.02	0.00	0.00	0.00	3.85E-01	5.92E-01	1.86E-03	7.36E-01	1.00E+00	1.00E+00	1.00E+00	tRNA pseudouridine synthase C [EC:5.4.99.12]
KO:K06176	4.36	0.08	0.00	0.46	0.00	0.16	0.62	2.37E-02	7.98E-01	1.00E+00	4.91E-01	1.00E+00	7.22E-01	3.90E-01	tRNA pseudouridine synthase D [EC:5.4.99.12]
KO:K06177	0.72	9.50	0.20	0.19	0.44	0.00	0.02	5.85E-01	7.57E-03	8.45E-01	8.49E-01	7.29E-01	1.00E+00	9.43E-01	ribosomal large subunit pseudouridine synthase A [EC:5.4.99.12]
KO:K06178	8.12	4.19	4.13	2.59	1.44	1.56	1.01	5.03E-02	3.09E-01	3.20E-01	6.94E-01	8.93E-01	8.78E-01	9.37E-01	ribosomal large subunit pseudouridine synthase B [EC:5.4.99.12]
KO:K06179	6.87	13.15	0.69	2.12	0.00	0.03	1.22	7.57E-02	1.14E-02	9.35E-01	7.18E-01	1.00E+00	9.89E-01	8.72E-01	ribosomal large subunit pseudouridine synthase C [EC:5.4.99.12]
KO:K06180	4.21	3.98	5.34	2.98	5.69	3.07	2.18	3.90E-01	4.56E-01	1.83E-01	7.38E-01	1.46E-01	7.14E-01	8.79E-01	ribosomal large subunit pseudouridine synthase D [EC:5.4.99.12]
KO:K06181	2.61	0.93	0.00	0.84	5.61	0.07	0.04	1.00E-01	4.57E-01	1.00E+00	5.04E-01	2.53E-02	9.24E-01	9.49E-01	ribosomal large subunit pseudouridine synthase E [EC:5.4.99.12]
KO:K06182	0.26	0.54	0.00	1.71	0.00	2.33	0.08	5.75E-01	3.84E-01	1.00E+00	1.25E-01	1.00E+00	8.13E-02	7.40E-01	ribosomal large subunit pseudouridine synthase F [EC:5.4.99.12]
KO:K06183	0.22	0.06	0.08	0.17	0.70	0.00	0.00	2.78E-01	4.88E-01	4.55E-01	3.22E-01	8.30E-02	1.00E+00	1.00E+00	ribosomal small subunit pseudouridine synthase A [EC:5.4.99.12]
KO:K06186	5.25	0.01	0.02	0.55	0.00	0.00	0.96	2.87E-02	8.16E-01	7.96E-01	4.22E-01	1.00E+00	1.00E+00	2.78E-01	small protein A
KO:K06187	5.75	5.56	5.97	2.57	1.80	5.29	0.37	1.56E-01	1.71E-01	1.40E-01	7.63E-01	8.82E-01	1.98E-01	9.84E-01	recombination protein RecR
KO:K06188	0.00	0.10	2.64	0.02	0.56	0.00	3.79	1.00E+00	5.41E-01	4.49E-02	6.80E-01	2.48E-01	1.00E+00	3.23E-02	aquaporin Z
KO:K06189	5.33	12.22	0.52	0.59	0.00	1.64	3.38	1.42E-01	1.69E-02	9.32E-01	9.25E-01	1.00E+00	7.76E-01	3.72E-01	magnesium and cobalt transporter
KO:K06190	4.26	14.35	0.01	3.38	0.00	0.00	0.66	2.17E-01	1.06E-02	9.64E-01	3.37E-01	1.00E+00	1.00E+00	8.86E-01	intracellular septation protein
KO:K06192	0.02	0.00	2.46	0.01	1.38	0.00	0.02	5.45E-01	6.15E-01	1.93E-02	5.93E-01	4.70E-02	1.00E+00	5.58E-01	paraquat-inducible protein B
KO:K06193	0.61	0.36	0.04	0.24	0.00	1.13	0.00	1.61E-01	2.82E-01	7.71E-01	4.21E-01	1.00E+00	7.19E-02	1.00E+00	phosphonoacetate hydrolase [EC:3.11.1.2]
KO:K06194	7.01	0.18	2.10	1.37	0.00	0.53	0.23	2.39E-02	8.58E-01	2.45E-01	4.22E-01	1.00E+00	7.35E-01	8.39E-01	lipoprotein NlpD
KO:K06195	0.83	0.20	1.11	4.36	0.00	3.49	0.58	5.71E-01	8.46E-01	4.60E-01	5.40E-02	1.00E+00	8.24E-02	6.84E-01	ApaG protein
KO:K06196	0.09	17.09	0.00	3.22	2.43	0.00	0.08	9.12E-01	7.57E-03	1.00E+00	2.62E-01	3.86E-01	1.00E+00	9.14E-01	cytochrome c-type biogenesis protein
KO:K06199	2.82	3.09	0.45	1.11	6.43	0.66	1.80	2.96E-01	2.47E-01	9.18E-01	7.85E-01	3.96E-02	8.86E-01	5.77E-01	CrcB protein
KO:K06200	0.19	0.01	21.08	1.10	1.18	0.05	0.00	6.31E-01	7.42E-01	1.86E-03	3.51E-01	3.34E-01	7.12E-01	1.00E+00	carbon starvation protein
KO:K06201	0.20	1.32	0.00	1.70	0.00	0.00	0.00	4.98E-01	1.34E-01	1.00E+00	9.41E-02	1.00E+00	1.00E+00	1.00E+00	copper homeostasis protein
KO:K06202	4.00	0.01	0.00	0.00	0.00	0.00	0.00	3.31E-02	5.04E-01	1.00E+00	5.46E-01	1.00E+00	1.00E+00	1.00E+00	CyaY protein
KO:K06203	4.20	0.13	0.00	0.76	0.00	0.00	0.12	3.81E-02	6.58E-01	1.00E+00	2.92E-01	1.00E+00	1.00E+00	6.73E-01	CysZ protein
KO:K06204	7.48	10.95	4.49	3.84	0.00	1.25	3.71	1.47E-01	4.63E-02	4.98E-01	6.18E-01	1.00E+00	9.32E-01	6.43E-01	DnaK suppressor protein
KO:K06205	0.06	0.49	0.00	0.00	0.00	0.00	0.00	1.94E-01	9.86E-02	1.00E+00	1.00E+00	1.00E+00	1.00E+00	1.00E+00	MioC protein
KO:K06206	1.94	2.18	0.37	1.62	1.48	0.00	0.04	1.86E-01	1.49E-01	8.17E-01	2.59E-01	3.00E-01	1.00E+00	9.62E-01	sugar fermentation stimulation protein A
KO:K06207	11.23	8.65	8.80	6.50	9.19	6.06	2.72	9.99E-02	3.27E-01	3.04E-01	7.27E-01	2.47E-01	7.89E-01	9.77E-01	GTP-binding protein
KO:K06208	0.00	0.00	4.01	0.00	10.89	0.00	0.00	1.00E+00	1.00E+00	1.77E-02	1.00E+00	1.29E-03	1.00E+00	1.00E+00	chorismate mutase [EC:5.4.99.5]
KO:K06211	0.00	0.00	0.38	0.00	0.00	0.00	0.17	1.00E+00	1.00E+00	3.04E-02	1.00E+00	1.00E+00	1.00E+00	8.40E-02	HipB family transcriptional regulator, involved in the regulation of
KO:K06213	8.63	8.54	2.53	8.83	4.62	0.33	1.89	1.19E-01	1.23E-01	9.27E-01	1.09E-01	6.96E-01	9.94E-01	9.55E-01	magnesium transporter
KO:K06214	0.66	0.54	0.00	1.06	0.00	0.00	0.00	2.34E-01	2.63E-01	1.00E+00	1.56E-01	1.00E+00	1.00E+00	1.00E+00	curli production assembly/transport component CsgG
KO:K06215	0.00	0.00	0.00	0.00	0.00	0.00	7.08	1.00E+00	1.00E+00	1.00E+00	1.00E+00	1.00E+00	1.00E+00	6.00E-03	pyridoxine biosynthesis protein [EC:4.-.-.-]
KO:K06217	6.53	6.77	5.57	3.71	6.87	3.05	0.22	1.60E-01	1.43E-01	2.79E-01	6.93E-01	1.35E-01	8.15E-01	9.95E-01	phosphate starvation-inducible protein PhoH and related proteins
KO:K06218	0.00	0.00	1.13	0.16	0.00	0.00	0.06	4.21E-01	1.00E+00	4.40E-02	2.06E-01	1.00E+00	1.00E+00	2.92E-01	RelE protein
KO:K06219	0.72	0.01	0.00	0.00	0.00	0.00	0.00	4.06E-02	3.84E-01	1.00E+00	1.00E+00	1.00E+00	1.00E+00	1.00E+00	S-adenosylmethionine-dependent methyltransferase
KO:K06222	0.00	0.22	0.00	0.00	0.00	0.00	0.00	1.00E+00	6.83E-02	1.00E+00	1.00E+00	1.00E+00	1.00E+00	1.00E+00	2,5-diketo-D-gluconate reductase B [EC:1.1.1.274]
KO:K06223	0.01	0.00	0.00	0.05	0.87	0.00	1.95	5.92E-01	6.29E-01	1.00E+00	4.55E-01	6.50E-02	1.00E+00	2.19E-02	DNA adenine methylase [EC:2.1.1.72]
KO:K06236	0.00	0.00	0.00	0.00	0.00	0.00	5.83	1.00E+00	1.00E+00	1.00E+00	1.00E+00	1.00E+00	1.00E+00	2.73E-02	collagen, type I/II/III/V/XI, alpha
KO:K06281	1.17	0.01	0.00	0.72	0.00	0.00	0.00	1.02E-01	2.99E-01	1.00E+00	2.03E-01	1.00E+00	1.00E+00	1.00E+00	hydrogenase large subunit [EC:1.12.99.6]
KO:K06282	1.52	0.00	0.00	0.88	0.00	0.00	0.00	9.11E-02	1.00E+00	1.00E+00	1.75E-01	1.00E+00	1.00E+00	1.00E+00	hydrogenase small subunit [EC:1.12.99.6]
KO:K06287	3.18	12.19	0.50	2.68	2.97	4.12	0.26	5.44E-01	1.30E-02	9.70E-01	6.70E-01	5.97E-01	3.23E-01	9.85E-01	septum formation protein
KO:K06346	0.43	0.00	0.00	0.05	0.00	0.00	0.00	9.51E-02	1.00E+00	1.00E+00	2.30E-01	1.00E+00	1.00E+00	1.00E+00	spoIIIJ-associated protein
KO:K06350	0.12	0.06	0.00	0.57	0.00	0.00	0.00	2.66E-01	3.35E-01	1.00E+00	1.15E-01	1.00E+00	1.00E+00	1.00E+00	antagonist of KipI
KO:K06351	0.00	0.44	0.00	0.00	0.00	0.00	0.00	1.00E+00	6.51E-02	1.00E+00	1.00E+00	1.00E+00	1.00E+00	1.00E+00	inhibitor of KinA
KO:K06374	0.00	0.00	0.00	0.51	0.00	0.00	0.00	1.00E+00	1.00E+00	1.00E+00	1.43E-01	1.00E+00	1.00E+00	1.00E+00	spore maturation protein B
KO:K06378	0.14	0.00	0.00	0.79	0.00	0.00	0.00	2.07E-01	1.00E+00	1.00E+00	9.26E-02	1.00E+00	1.00E+00	1.00E+00	stage II sporulation protein AA (anti-sigma F factor antagonist)
KO:K06381	0.00	0.00	0.00	0.04	6.40	0.00	0.00	1.00E+00	1.00E+00	1.00E+00	3.63E-01	4.00E-03	1.00E+00	1.00E+00	stage II sporulation protein D
KO:K06400	0.00	0.00	0.00	0.02	0.00	0.00	0.33	1.00E+00	1.00E+00	1.00E+00	2.24E-01	1.00E+00	1.00E+00	3.71E-02	site-specific DNA recombinase
KO:K06412	0.00	0.00	3.03	0.00	0.00	0.00	0.00	1.00E+00	1.00E+00	7.71E-03	2.51E-01	1.00E+00	1.00E+00	1.00E+00	stage V sporulation protein G
KO:K06415	0.00	0.01	0.70	0.23	0.70	0.00	0.48	6.63E-01	6.24E-01	1.13E-01	2.92E-01	1.13E-01	1.00E+00	1.63E-01	stage V sporulation protein R
KO:K06442	0.28	0.00	2.87	0.32	16.09	0.00	0.03	5.97E-01	8.50E-01	8.77E-02	5.70E-01	3.71E-03	1.00E+00	7.89E-01	putative hemolysin
KO:K06443	2.89	0.30	0.00	1.32	2.54	4.95	0.00	1.46E-01	7.65E-01	1.00E+00	3.93E-01	1.75E-01	6.39E-02	1.00E+00	lycopene beta cyclase [EC:1.14.-.-]
KO:K06444	0.00	0.00	0.00	0.00	2.50	0.00	0.00	1.00E+00	1.00E+00	1.00E+00	1.00E+00	1.73E-02	1.00E+00	1.00E+00	lycopene epsilon cyclase [EC:1.14.-.-]
KO:K06445	1.65	0.07	0.09	1.80	0.00	0.00	1.16	1.32E-01	7.95E-01	7.80E-01	1.13E-01	1.00E+00	1.00E+00	2.07E-01	acyl-CoA dehydrogenase [EC:1.3.99.-]
KO:K06446	1.89	0.10	1.72	0.65	0.00	0.00	0.00	1.26E-01	5.50E-01	1.40E-01	3.10E-01	1.00E+00	1.00E+00	1.00E+00	acyl-CoA dehydrogenase [EC:1.3.99.-]
KO:K06447	1.19	0.07	0.04	0.59	0.40	0.00	0.00	9.06E-02	6.60E-01	6.98E-01	2.21E-01	3.15E-01	1.00E+00	1.00E+00	succinylglutamic semialdehyde dehydrogenase [EC:1.2.1.71]
KO:K06518	0.00	0.00	0.00	1.02	0.00	0.00	0.00	1.00E+00	1.00E+00	1.00E+00	1.43E-01	1.00E+00	1.00E+00	1.00E+00	holin-like protein
KO:K06596	2.91	0.00	2.98	0.23	0.00	0.00	0.64	7.64E-02	1.00E+00	7.39E-02	5.34E-01	1.00E+00	1.00E+00	3.75E-01	chemosensory pili system protein ChpA (sensor histidine
KO:K06597	0.00	0.00	0.00	0.31	0.00	0.00	0.00	1.00E+00	1.00E+00	1.00E+00	4.90E-02	1.00E+00	1.00E+00	1.00E+00	chemosensory pili system protein ChpB (putative protein-glutamate
KO:K06598	0.16	0.00	0.00	0.22	0.00	0.00	0.00	1.45E-01	1.00E+00	1.00E+00	1.13E-01	1.00E+00	1.00E+00	1.00E+00	chemosensory pili system protein ChpC
KO:K06600	0.12	0.00	0.00	0.00	0.00	0.00	0.00	6.39E-02	1.00E+00	1.00E+00	1.00E+00	1.00E+00	1.00E+00	1.00E+00	chemosensory pili system protein ChpE
KO:K06601	0.05	0.01	0.00	3.19	0.00	0.00	0.00	4.88E-01	5.92E-01	1.00E+00	4.17E-02	1.00E+00	1.00E+00	1.00E+00	flagellar protein FlbT
KO:K06602	0.00	0.10	0.00	1.83	0.00	0.00	0.00	1.00E+00	3.23E-01	1.00E+00	5.17E-02	1.00E+00	1.00E+00	1.00E+00	flagellar protein FlaF
KO:K06603	5.45	0.04	0.35	0.32	0.00	6.25	0.00	4.26E-02	8.25E-01	6.30E-01	6.50E-01	1.00E+00	3.46E-02	1.00E+00	flagellar protein FlaG
KO:K06606	0.03	0.08	7.17	0.17	0.88	0.00	0.03	6.24E-01	5.55E-01	9.71E-03	4.59E-01	1.90E-01	1.00E+00	6.22E-01	inosose isomerase [EC:5.3.99.-]
KO:K06718	0.05	0.04	0.00	0.58	0.00	0.00	0.94	5.36E-01	5.46E-01	1.00E+00	1.15E-01	1.00E+00	1.00E+00	7.14E-02	L-2,4-diaminobutyric acid acetyltransferase [EC:2.3.1.178]
KO:K06720	0.34	0.04	0.20	0.58	0.05	0.00	0.35	2.95E-01	6.40E-01	4.12E-01	1.78E-01	6.13E-01	1.00E+00	2.82E-01	L-ectoine synthase [EC:4.2.1.18]
KO:K06726	0.00	0.00	5.24	0.00	0.00	0.00	0.03	1.00E+00	1.00E+00	1.49E-02	1.00E+00	1.00E+00	1.00E+00	1.66E-01	D-ribose pyranase [EC:5.-.-.-]
KO:K06857	0.12	5.34	0.00	0.47	0.00	1.94	0.00	7.94E-01	1.91E-02	1.00E+00	5.40E-01	1.00E+00	1.16E-01	1.00E+00	putative tungstate transport system ATP-binding protein
KO:K06858	0.12	0.00	0.00	0.00	0.00	0.00	0.00	6.33E-02	1.00E+00	1.00E+00	1.00E+00	1.00E+00	1.00E+00	1.00E+00	vitamin B12 transport system substrate-binding protein
KO:K06859	0.00	0.33	0.00	0.00	0.00	0.00	0.00	1.00E+00	1.02E-01	1.00E+00	1.00E+00	1.00E+00	1.00E+00	1.00E+00	glucose-6-phosphate isomerase, archaeal [EC:5.3.1.9]
KO:K06860	0.07	0.12	0.00	0.28	6.72	3.25	0.00	6.10E-01	5.77E-01	1.00E+00	4.63E-01	1.46E-02	5.66E-02	1.00E+00	
KO:K06861	4.07	4.73	2.48	4.88	5.81	3.00	1.85	4.60E-01	3.00E-01	8.41E-01	2.71E-01	1.48E-01	7.46E-01	9.15E-01	lipopolysaccharide export system ATP-binding protein [EC:3.6.3.-]
KO:K06863	0.00	0.00	0.00	0.00	0.00	0.00	5.83	1.00E+00	1.00E+00	1.00E+00	1.00E+00	1.00E+00	1.00E+00	1.64E-02	5-formaminoimidazole-4-carboxamide-1-(beta)-D-ribofuranosyl
KO:K06864	0.00	0.02	0.80	0.00	19.08	0.00	0.74	1.00E+00	7.54E-01	2.05E-01	7.71E-01	1.86E-03	1.00E+00	2.25E-01	
KO:K06865	0.00	0.00	0.00	0.00	0.00	0.00	5.00	1.00E+00	1.00E+00	1.00E+00	1.00E+00	1.00E+00	1.00E+00	6.43E-03	

KO:K06867	0.00	0.03	0.23	0.40	0.00	0.00	0.00	1.00E+00	4.15E-01	1.34E-01	8.41E-02	1.00E+00	1.00E+00	1.00E+00	
KO:K06869	0.00	0.00	0.00	0.00	0.00	0.00	3.33	1.00E+00	1.00E+00	1.00E+00	1.00E+00	1.00E+00	1.00E+00	1.50E-02	
KO:K06871	0.02	0.03	0.00	0.05	8.69	0.00	0.11	6.12E-01	5.88E-01	1.00E+00	5.47E-01	4.29E-03	1.00E+00	4.30E-01	
KO:K06872	0.03	0.07	0.01	0.13	28.22	0.25	0.00	7.66E-01	7.29E-01	8.10E-01	6.60E-01	0.00E+00	5.65E-01	1.00E+00	
KO:K06873	0.01	0.00	0.19	0.00	34.37	0.00	0.65	7.22E-01	7.36E-01	5.02E-01	7.28E-01	2.86E-04	1.00E+00	3.04E-01	
KO:K06875	0.00	0.00	0.00	0.00	0.00	0.00	1.25	1.00E+00	1.00E+00	1.00E+00	1.00E+00	1.00E+00	1.00E+00	2.90E-02	
KO:K06876	5.40	3.75	6.64	5.94	3.24	1.36	0.38	2.07E-01	5.20E-01	1.14E-01	1.59E-01	6.49E-01	9.26E-01	9.84E-01	
KO:K06877	0.07	0.00	0.00	0.01	0.00	0.00	5.39	4.08E-01	1.00E+00	1.00E+00	5.17E-01	1.00E+00	1.00E+00	7.14E-03	
KO:K06878	0.75	5.62	0.00	6.41	0.04	0.00	0.00	6.85E-01	5.27E-02	1.00E+00	4.14E-02	9.48E-01	1.00E+00	9.64E-01	
KO:K06879	0.95	0.22	3.85	0.59	1.44	0.00	0.13	3.05E-01	6.90E-01	4.74E-02	4.53E-01	1.96E-01	1.00E+00	7.51E-01	7-cyano-7-deazaguanine reductase [EC:1.7.1.13]
KO:K06881	0.77	0.42	16.55	0.62	1.78	4.14	0.01	6.45E-01	7.58E-01	4.86E-03	6.98E-01	3.36E-01	1.02E-01	8.75E-01	
KO:K06883	0.00	0.00	0.07	0.01	23.14	0.00	0.62	7.28E-01	1.00E+00	5.98E-01	7.04E-01	2.86E-04	1.00E+00	2.58E-01	
KO:K06885	0.56	0.42	0.13	0.49	2.40	7.14	0.03	5.28E-01	6.07E-01	7.75E-01	5.63E-01	1.07E-01	1.21E-02	8.39E-01	
KO:K06886	0.16	0.42	0.57	2.59	0.25	0.45	0.11	6.93E-01	4.95E-01	4.15E-01	8.36E-02	6.17E-01	4.78E-01	7.47E-01	hemoglobin
KO:K06887	0.00	0.00	0.00	0.10	0.00	0.00	0.00	1.00E+00	1.00E+00	1.00E+00	1.43E-01	1.00E+00	1.00E+00	1.00E+00	
KO:K06888	0.95	0.23	6.64	0.84	0.45	3.22	0.64	5.00E-01	8.03E-01	2.56E-02	5.43E-01	7.16E-01	1.04E-01	6.27E-01	
KO:K06889	0.59	0.38	7.15	2.78	0.39	0.23	0.01	5.81E-01	6.70E-01	3.54E-02	1.51E-01	6.67E-01	7.45E-01	8.86E-01	
KO:K06890	5.92	10.31	3.00	12.56	6.76	9.67	3.29	7.63E-01	1.91E-01	9.44E-01	8.37E-02	6.47E-01	2.41E-01	9.35E-01	
KO:K06891	6.11	2.78	5.67	3.92	6.50	1.03	0.49	1.42E-01	6.83E-01	1.74E-01	4.30E-01	1.18E-01	9.29E-01	9.64E-01	ATP-dependent Clp protease adaptor protein ClpS
KO:K06892	4.65	8.07	0.00	2.65	0.00	1.51	0.01	1.27E-01	3.93E-02	1.00E+00	3.48E-01	1.00E+00	6.24E-01	9.43E-01	
KO:K06893	1.55	0.00	0.00	0.03	0.12	0.00	0.00	1.15E-01	1.00E+00	1.00E+00	2.50E-01	1.75E-01	1.00E+00	1.00E+00	
KO:K06894	0.56	0.86	0.18	1.85	0.32	0.14	0.00	4.50E-01	2.75E-01	7.77E-01	8.04E-02	6.53E-01	8.09E-01	1.00E+00	
KO:K06895	1.63	6.74	0.00	0.82	0.00	0.00	0.10	2.56E-01	2.96E-02	1.00E+00	4.93E-01	1.00E+00	1.00E+00	8.23E-01	
KO:K06896	0.29	0.10	1.51	0.60	0.18	0.73	2.10	5.60E-01	7.41E-01	1.51E-01	3.73E-01	6.52E-01	3.22E-01	1.00E-01	
KO:K06897	1.33	0.30	0.10	0.00	0.00	0.00	0.00	1.24E-01	2.26E-01	2.59E-01	1.00E+00	1.00E+00	1.00E+00	1.00E+00	
KO:K06898	0.00	0.00	1.77	0.01	20.45	0.00	1.13	7.28E-01	1.00E+00	1.26E-01	7.11E-01	7.14E-04	1.00E+00	1.94E-01	
KO:K06899	0.81	0.00	0.00	0.00	0.00	0.00	0.00	3.90E-02	1.00E+00	1.00E+00	1.00E+00	1.00E+00	1.00E+00	1.00E+00	
KO:K06900	5.05	0.00	0.06	0.00	0.00	0.00	0.00	5.04E-02	4.15E-01	3.54E-01	1.00E+00	1.00E+00	1.00E+00	1.00E+00	
KO:K06901	6.85	0.35	3.88	6.00	1.40	0.00	0.23	9.80E-02	8.51E-01	2.59E-01	1.27E-01	6.49E-01	1.00E+00	8.63E-01	putative MFS transporter, AGZA family, xanthine/uracil permease
KO:K06902	2.42	14.86	0.19	4.25	0.00	4.04	0.00	6.14E-01	1.31E-02	9.50E-01	2.63E-01	1.00E+00	2.91E-01	1.00E+00	MFS transporter, UMF1 family
KO:K06903	0.09	0.04	0.00	0.48	0.00	0.00	0.00	2.90E-01	3.82E-01	1.00E+00	1.01E-01	1.00E+00	1.00E+00	1.00E+00	
KO:K06904	0.00	0.30	0.00	0.13	0.00	0.00	1.72	1.00E+00	2.19E-01	1.00E+00	3.29E-01	1.00E+00	1.00E+00	4.06E-02	
KO:K06905	0.00	0.00	0.00	0.41	0.00	0.00	0.00	1.00E+00	1.00E+00	1.00E+00	1.43E-01	1.00E+00	1.00E+00	1.00E+00	
KO:K06906	0.00	0.11	0.00	0.00	0.00	0.00	0.00	1.00E+00	9.79E-02	1.00E+00	1.00E+00	1.00E+00	1.00E+00	1.00E+00	
KO:K06907	0.87	0.16	0.00	1.64	0.00	6.36	0.00	3.20E-01	5.97E-01	1.00E+00	1.81E-01	1.00E+00	3.26E-02	1.00E+00	
KO:K06909	0.00	0.22	0.00	0.00	0.00	0.00	0.00	1.00E+00	1.02E-01	1.00E+00	1.00E+00	1.00E+00	1.00E+00	1.00E+00	
KO:K06910	0.00	0.01	1.25	0.10	0.48	0.00	1.60	6.61E-01	6.48E-01	7.53E-02	4.39E-01	1.99E-01	1.00E+00	5.46E-02	
KO:K06911	5.85	22.78	2.76	6.66	1.07	2.28	0.22	4.73E-01	1.86E-03	9.03E-01	3.31E-01	9.74E-01	9.30E-01	9.92E-01	
KO:K06912	1.01	0.04	0.00	5.40	0.00	0.00	0.00	2.72E-01	5.23E-01	1.00E+00	5.97E-02	1.00E+00	1.00E+00	1.00E+00	alpha-ketoglutarate-dependent 2,4-dichlorophenoxyacetate dioxygenase
KO:K06915	0.12	2.68	0.00	0.64	0.00	0.00	0.07	7.36E-01	2.30E-02	1.00E+00	2.23E-01	1.00E+00	1.00E+00	8.02E-01	
KO:K06916	6.98	7.09	0.38	1.45	0.00	5.29	0.49	5.70E-02	5.47E-02	9.53E-01	8.02E-01	1.00E+00	1.11E-01	9.45E-01	
KO:K06917	2.28	0.46	0.70	0.08	4.60	1.55	0.00	9.80E-02	6.57E-01	4.96E-01	8.85E-01	2.34E-02	1.91E-01	1.00E+00	tRNA 2-selenouridine synthase [EC:2.9.1.-]
KO:K06918	0.03	5.37	0.00	0.00	0.00	0.00	0.00	7.83E-01	1.43E-03	1.00E+00	8.19E-01	1.00E+00	1.00E+00	1.00E+00	
KO:K06919	0.02	0.00	0.00	0.03	2.06	0.00	0.00	3.51E-01	1.00E+00	1.00E+00	3.34E-01	7.43E-03	1.00E+00	1.00E+00	
KO:K06920	3.55	0.05	11.45	0.70	1.44	0.03	4.63	1.24E-01	9.48E-01	6.43E-03	8.02E-01	5.48E-01	9.69E-01	6.67E-02	queuosine biosynthesis protein QueC
KO:K06921	0.16	0.00	0.00	0.03	0.00	0.00	0.00	8.90E-02	1.00E+00	1.00E+00	2.51E-01	1.00E+00	1.00E+00	1.00E+00	
KO:K06922	1.10	0.25	0.00	0.00	0.00	0.00	0.00	1.06E-01	2.07E-01	1.00E+00	1.00E+00	1.00E+00	1.00E+00	1.00E+00	
KO:K06923	0.41	12.40	0.03	1.92	0.00	1.80	0.15	8.49E-01	9.71E-03	9.46E-01	4.00E-01	1.00E+00	4.30E-01	9.15E-01	
KO:K06925	2.84	1.35	0.89	1.61	1.40	0.40	1.10	1.23E-01	5.19E-01	7.34E-01	4.10E-01	4.99E-01	9.03E-01	6.30E-01	
KO:K06927	0.00	0.01	0.00	0.00	0.00	0.00	1.56	1.00E+00	2.38E-01	1.00E+00	1.00E+00	1.00E+00	1.00E+00	2.39E-02	
KO:K06929	1.49	7.21	0.00	1.23	0.00	0.00	0.43	3.45E-01	2.69E-02	1.00E+00	4.26E-01	1.00E+00	1.00E+00	7.37E-01	
KO:K06932	0.00	0.00	0.00	0.00	0.00	0.00	3.33	1.00E+00	1.00E+00	1.00E+00	1.00E+00	1.00E+00	1.00E+00	1.74E-02	
KO:K06934	0.12	0.00	0.00	0.00	0.00	0.00	0.00	6.44E-02	1.00E+00	1.00E+00	1.00E+00	1.00E+00	1.00E+00	1.00E+00	
KO:K06936	0.00	0.00	1.88	0.00	0.00	0.00	0.00	1.00E+00	1.00E+00	1.69E-02	1.00E+00	1.00E+00	1.00E+00	1.00E+00	
KO:K06937	0.00	0.00	0.04	0.00	0.14	0.00	7.53	1.00E+00	4.52E-01	3.28E-01	1.00E+00	2.65E-01	1.00E+00	1.26E-02	
KO:K06938	8.82	2.23	0.01	0.25	0.00	0.00	0.02	1.13E-02	1.63E-01	9.14E-01	7.83E-01	1.00E+00	1.00E+00	9.11E-01	
KO:K06940	0.24	0.01	5.23	0.12	6.07	0.00	1.66	6.36E-01	8.90E-01	2.30E-02	7.44E-01	1.84E-02	1.00E+00	1.39E-01	
KO:K06941	12.29	8.80	1.34	4.06	5.55	2.55	3.98	3.10E-02	1.18E-01	9.85E-01	8.23E-01	5.58E-01	9.39E-01	8.32E-01	ribosomal RNA large subunit methyltransferase N [EC:2.1.1.-]
KO:K06942	6.29	9.70	7.11	3.82	7.50	2.50	4.45	4.19E-01	7.13E-02	2.68E-01	8.73E-01	2.18E-01	9.53E-01	8.00E-01	
KO:K06944	0.00	0.00	0.00	0.00	0.00	0.00	2.50	1.00E+00	1.00E+00	1.00E+00	1.00E+00	1.00E+00	1.00E+00	1.93E-02	
KO:K06946	0.19	0.00	0.00	0.14	0.00	0.00	0.00	1.01E-01	1.00E+00	1.00E+00	2.11E-01	1.00E+00	1.00E+00	1.00E+00	
KO:K06948	0.12	0.01	0.16	0.32	7.75	0.00	0.28	5.92E-01	7.30E-01	5.46E-01	4.05E-01	7.57E-03	1.00E+00	4.35E-01	
KO:K06949	2.90	0.69	1.01	0.95	6.31	0.59	0.83	1.37E-01	7.99E-01	6.80E-01	7.11E-01	2.26E-02	8.31E-01	7.53E-01	ribosome biogenesis GTPase [EC:3.6.1.-]
KO:K06950	2.23	0.90	2.11	1.89	0.99	2.04	0.13	2.68E-01	6.22E-01	2.94E-01	3.35E-01	5.91E-01	3.07E-01	8.55E-01	
KO:K06952	0.51	4.60	0.00	0.17	0.00	0.00	0.00	3.53E-01	2.21E-02	1.00E+00	6.44E-01	1.00E+00	1.00E+00	1.00E+00	
KO:K06953	0.07	7.80	0.00	0.11	0.00	0.00	0.05	7.89E-01	8.14E-03	1.00E+00	7.18E-01	1.00E+00	1.00E+00	8.01E-01	
KO:K06954	2.09	20.37	0.02	2.01	0.00	2.94	0.22	6.59E-01	4.43E-03	9.56E-01	6.78E-01	1.00E+00	4.71E-01	9.38E-01	
KO:K06955	1.60	0.56	11.81	1.51	4.02	0.00	0.00	4.20E-01	7.46E-01	1.06E-02	4.45E-01	1.17E-01	1.00E+00	9.08E-01	
KO:K06956	0.00	0.67	0.00	0.00	0.00	0.00	0.00	1.00E+00	6.64E-02	1.00E+00	1.00E+00	1.00E+00	1.00E+00	1.00E+00	
KO:K06957	0.02	0.07	0.00	0.22	0.00	0.00	0.00	2.74E-01	2.27E-01	1.00E+00	1.40E-01	1.00E+00	1.00E+00	1.00E+00	
KO:K06958	2.79	16.21	0.00	0.45	0.00	0.05	0.34	2.34E-01	3.00E-03	1.00E+00	8.76E-01	1.00E+00	9.57E-01	8.97E-01	
KO:K06959	1.98	0.09	5.72	0.72	0.04	0.00	1.27	1.30E-01	8.83E-01	2.03E-02	4.90E-01	9.32E-01	1.00E+00	2.61E-01	
KO:K06960	0.00	0.00	0.00	0.00	9.80	0.00	0.22	1.00E+00	1.00E+00	1.00E+00	1.00E+00	1.44E-02	1.00E+00	1.67E-01	
KO:K06961	0.00	0.00	0.00	0.00	0.00	0.00	4.17	1.00E+00	1.00E+00	1.00E+00	1.00E+00	1.00E+00	1.00E+00	1.66E-02	
KO:K06962	0.00	0.00	4.20	0.00	0.00	0.00	0.12	3.36E-01	1.00E+00	1.26E-02	1.00E+00	1.00E+00	1.00E+00	1.85E-01	
KO:K06963	0.00	0.00	0.00	0.00	0.00	0.00	2.50	1.00E+00	1.00E+00	1.00E+00	1.00E+00	1.00E+00	1.00E+00	1.69E-02	
KO:K06965	0.00	0.00	0.00	0.00	0.00	0.00	1.25	1.00E+00	1.00E+00	1.00E+00	1.00E+00	1.00E+00	1.00E+00	2.90E-02	
KO:K06966	3.57	2.78	4.65	2.77	6.31	3.92	1.11	4.04E-01	6.22E-01	2.11E-01	6.25E-01	8.84E-02	3.26E-01	9.17E-01	
KO:K06967	0.12	0.00	0.00	0.00	0.00	0.00	0.00	6.43E-02	1.00E+00	1.00E+00	1.00E+00	1.00E+00	1.00E+00	1.00E+00	
KO:K06968	0.15	0.09	1.06	0.01	0.00	0.00	0.00	2.46E-01	3.35E-01	3.24E-02	5.63E-01	1.00E+00	1.00E+00	1.00E+00	
KO:K06969	1.73	9.18	1.63	0.26	2.08	3.04	0.45	6.14E-01	1.91E-02	6.44E-01	9.32E-01	5.18E-01	2.93E-01	9.05E-01	putative SAM-dependent methyltransferase
KO:K06970	0.04	0.53	0.00	0.65	0.00	0.00	0.30	5.54E-01	1.75E-01	1.00E+00	1.47E-01	1.00E+00	1.00E+00	2.80E-01	ribosomal RNA large subunit methyltransferase F [EC:2.1.1.48]
KO:K06971	0.00	0.75	0.00	0.64	1.63	0.00	0.00	1.00E+00	1.76E-01	1.00E+00	1.98E-01	9.77E-02	1.00E+00	1.00E+00	
KO:K06972	13.31	0.20	0.00	0.89	0.00	0.00	4.05	1.31E-02	7.86E-01	1.00E+00	6.22E-01	1.00E+00	1.00E+00	1.70E-01	
KO:K06973	2.05	0.25	5.18	1.22	0.55	7.78	1.72	3.65E-01	8.81E-01	6.70E-02	6.02E-01	8.15E-01	2.71E-02	4.51E-01	
KO:K06974	0.00	0.00	1.61	0.03	0.00	0.00	0.00	1.00E+00	1.00E+00	1.03E-02	2.54E-01	1.00E+00	1.00E+00	1.00E+00	
KO:K06975	0.07	2.95	0.00	0.91	0.00	0.24	0.00	5.73E-01	7.81E-02	1.00E+00	2.40E-01	1.00E+00	4.68E-01	1.00E+00	
KO:K06977	0.75	0.00	0.00	2.03	0.00	0.00	0.00	1.82E-01	1.00E+00	1.00E+00	8.66E-02	1.00E+00	1.00E+00	1.00E+00	
KO:K06978	0.18	3.30	0.00	0.68	3.77	8.40	0.01	8.45E-01	1.03E-01	1.00E+00	6.27E-01	8.24E-02	2.23E-02	9.17E-01	
KO:K06979	9.14	2.73	0.06	5.15	0.00	0.00	0.01	3.47E-02	3.10E-01	9.33E-01	1.04E-01	1.00E+00	1.00E+00	9.50E-01	
KO:K06980	2.30	5.06	0.81	0.96	2.53	0.31	1.55	3.67E-01	6.94E-02	8.29E-01	7.91E-01	3.12E-01	9.27E-01	6.00E-01	
KO:K06981	0.00	0.00	0.00	0.00	0.00	0.00	2.92	1.00E+00	1.00E+00	1.00E+00	1.00E+00	1.00E+00	1.00E+00	1.59E-02	
KO:K06982	0.00	0.00	0.00	0.00	0.00	0.00	1.67	1.00E+00	1.00E+00	1.00E+00	1.00E+00	1.00E+00	1.00E+00	2.80E-02	
KO:K06985	1.37	5.59	0.00	0.31	0.00	0.00	0.08	1.98E-01	1.56E-02	1.00E+00	6.98E-01	1.00E+00	1.00E+00	8.77E-01	aspartyl protease family protein
KO:K06986	0.00	0.00	0.00	0.61	0.00	0.00	0.00	1.00E+00	1.00E+00	1.00E+00	1.43E-01	1.00E+00	1.00E+00	1.00E+00	
KO:K06987	3.26	2.63	1.32	1.45	0.01	0.89	0.01	1.15E-01	1.65E-01	4.52E-01	4.06E-01	9.61E-01	6.31E-01	9.59E-01	

KO:K06988	2.39	0.00	0.39	0.06	0.10	0.00	1.55	1.04E-01	6.18E-01	3.38E-01	5.14E-01	4.84E-01	1.00E+00	1.48E-01	
KO:K06989	0.00	0.28	0.00	1.09	0.00	0.00	1.64	1.00E+00	2.91E-01	1.00E+00	1.37E-01	1.00E+00	1.00E+00	8.69E-02	aspartate dehydrogenase [EC:1.4.1.21]
KO:K06990	0.13	0.00	0.00	0.14	0.00	0.00	1.20	3.01E-01	6.31E-01	1.00E+00	2.96E-01	1.00E+00	1.00E+00	2.84E-02	
KO:K06991	5.74	0.39	0.04	3.15	0.08	1.62	0.00	4.49E-02	7.70E-01	9.05E-01	1.34E-01	8.86E-01	3.57E-01	1.00E+00	
KO:K06992	0.04	0.00	0.00	0.13	0.00	0.00	0.00	1.67E-01	1.00E+00	1.00E+00	1.34E-01	1.00E+00	1.00E+00	1.00E+00	
KO:K06994	0.00	0.00	4.45	0.07	0.00	0.00	0.00	1.00E+00	1.00E+00	8.43E-03	2.53E-01	1.00E+00	1.00E+00	1.00E+00	putative drug exporter of the RND superfamily
KO:K06995	0.72	0.08	0.00	0.04	8.37	0.00	0.01	2.27E-01	6.27E-01	1.00E+00	6.88E-01	3.57E-03	1.00E+00	7.31E-01	
KO:K06996	0.03	0.04	0.85	0.57	0.00	0.00	0.38	6.02E-01	5.57E-01	9.40E-02	1.54E-01	1.00E+00	1.00E+00	2.32E-01	
KO:K06997	1.52	7.46	8.06	2.89	4.24	0.20	0.05	8.58E-01	2.94E-02	2.33E-02	4.84E-01	1.80E-01	9.91E-01	9.96E-01	
KO:K06998	0.35	6.34	1.25	1.36	1.07	0.00	0.65	8.09E-01	4.04E-02	4.98E-01	4.64E-01	5.69E-01	1.00E+00	7.17E-01	
KO:K06999	0.75	8.99	2.17	2.09	7.31	0.11	0.11	8.72E-01	3.04E-02	5.39E-01	5.62E-01	5.10E-02	9.62E-01	9.62E-01	
KO:K07000	0.44	0.02	0.51	0.10	0.00	0.00	0.63	1.84E-01	6.50E-01	1.58E-01	4.45E-01	1.00E+00	1.00E+00	1.24E-01	
KO:K07001	12.00	0.48	0.22	1.38	0.00	0.00	0.06	1.41E-02	7.07E-01	8.00E-01	4.17E-01	1.00E+00	1.00E+00	8.78E-01	
KO:K07003	8.85	0.35	2.85	5.36	0.66	4.96	5.28	7.04E-02	9.44E-01	6.26E-01	2.27E-01	9.21E-01	2.72E-01	2.35E-01	
KO:K07004	1.10	2.27	2.95	0.63	0.00	0.00	0.00	3.33E-01	1.74E-01	1.22E-01	4.56E-01	1.00E+00	1.00E+00	1.00E+00	
KO:K07005	0.12	0.44	0.05	0.47	0.00	0.00	0.60	5.27E-01	2.48E-01	6.25E-01	2.36E-01	1.00E+00	1.00E+00	1.86E-01	
KO:K07006	1.13	3.76	0.00	0.81	0.00	0.00	0.50	2.88E-01	4.80E-02	1.00E+00	4.06E-01	1.00E+00	1.00E+00	5.80E-01	
KO:K07007	2.44	4.60	6.67	1.40	3.24	1.53	0.02	4.54E-01	1.16E-01	4.20E-02	7.73E-01	2.64E-01	7.36E-01	9.93E-01	
KO:K07008	0.07	0.17	1.36	0.47	0.00	0.00	0.02	5.05E-01	3.72E-01	6.80E-02	1.93E-01	1.00E+00	1.00E+00	6.36E-01	
KO:K07010	0.32	18.65	0.35	1.07	0.06	0.00	0.45	8.82E-01	3.29E-03	8.76E-01	6.96E-01	9.33E-01	1.00E+00	8.59E-01	putative glutamine amidotransferase
KO:K07011	5.59	0.60	0.04	2.59	0.04	1.02	0.14	4.37E-02	7.08E-01	9.21E-01	1.70E-01	9.21E-01	5.47E-01	8.69E-01	
KO:K07012	0.25	0.03	0.09	0.02	0.00	0.63	0.04	1.90E-01	5.23E-01	3.66E-01	5.36E-01	1.00E+00	7.01E-02	4.87E-01	
KO:K07013	0.00	0.00	0.00	0.00	1.88	0.00	0.00	1.00E+00	1.00E+00	1.00E+00	1.00E+00	1.66E-02	1.00E+00	1.00E+00	
KO:K07014	0.05	0.00	1.58	0.00	0.00	0.00	0.00	2.44E-01	1.00E+00	1.43E-02	1.00E+00	1.00E+00	1.00E+00	1.00E+00	
KO:K07015	0.00	0.00	0.00	0.00	16.25	0.00	0.00	1.00E+00	1.00E+00	1.00E+00	1.00E+00	0.00E+00	1.00E+00	1.00E+00	
KO:K07016	0.06	0.00	0.00	0.54	0.00	0.00	0.00	2.11E-01	1.00E+00	1.00E+00	8.51E-02	1.00E+00	1.00E+00	1.00E+00	
KO:K07017	0.01	0.00	0.08	0.14	0.00	13.73	0.00	5.13E-01	5.65E-01	4.20E-01	3.62E-01	1.00E+00	5.71E-04	1.00E+00	
KO:K07018	0.50	13.53	0.01	8.15	0.00	0.00	0.75	8.65E-01	1.41E-02	9.61E-01	5.43E-02	1.00E+00	1.00E+00	8.32E-01	
KO:K07019	0.74	0.05	1.71	0.33	10.39	0.00	0.00	3.57E-01	8.78E-01	1.14E-01	6.41E-01	3.00E-03	1.00E+00	1.00E+00	
KO:K07020	0.02	0.01	0.11	0.19	4.82	0.00	0.20	7.19E-01	7.46E-01	5.27E-01	4.10E-01	8.00E-03	1.00E+00	3.99E-01	
KO:K07021	4.69	22.26	0.00	6.08	0.00	0.00	0.00	2.71E-01	3.14E-03	1.00E+00	1.49E-01	1.00E+00	1.00E+00	1.00E+00	
KO:K07023	0.00	0.05	6.77	0.00	0.00	0.00	0.25	6.21E-01	4.39E-01	3.86E-03	1.00E+00	1.00E+00	1.00E+00	2.32E-01	putative hydrolases of HD superfamily
KO:K07024	2.29	0.42	0.53	0.31	0.37	0.10	0.53	1.16E-01	5.13E-01	4.50E-01	5.81E-01	5.43E-01	7.41E-01	4.50E-01	
KO:K07025	5.64	2.48	1.05	5.30	1.92	2.64	0.42	1.04E-01	5.92E-01	8.90E-01	1.23E-01	7.40E-01	5.45E-01	9.64E-01	putative hydrolase of the HAD superfamily
KO:K07026	0.33	0.02	0.18	0.19	6.34	0.00	0.14	4.10E-01	8.00E-01	5.79E-01	5.63E-01	5.86E-03	1.00E+00	6.27E-01	mannosyl-3-phosphoglycerate phosphatase [EC:3.1.3.7]
KO:K07027	0.80	0.55	0.37	1.22	6.79	1.18	0.11	5.54E-01	6.65E-01	7.44E-01	3.84E-01	2.44E-02	3.97E-01	8.59E-01	
KO:K07028	0.20	0.28	0.00	0.83	0.99	0.00	0.07	4.64E-01	3.90E-01	1.00E+00	1.61E-01	1.27E-01	1.00E+00	6.20E-01	
KO:K07029	0.01	0.03	3.33	0.63	0.83	0.00	0.09	7.73E-01	7.42E-01	2.89E-02	2.12E-01	1.58E-01	1.00E+00	6.15E-01	
KO:K07031	0.09	0.13	0.51	2.17	0.00	0.00	2.04	5.90E-01	5.62E-01	3.75E-01	1.33E-01	1.00E+00	1.00E+00	1.43E-01	
KO:K07032	0.11	0.63	0.00	0.01	0.79	0.00	0.00	3.61E-01	1.27E-01	1.00E+00	5.81E-01	9.93E-02	1.00E+00	1.00E+00	
KO:K07033	0.00	0.00	0.00	0.03	8.27	0.00	0.00	1.00E+00	1.00E+00	1.00E+00	3.43E-01	3.29E-03	1.00E+00	1.00E+00	
KO:K07037	0.21	0.27	13.12	0.27	10.27	0.14	0.08	7.92E-01	7.61E-01	5.00E-03	7.58E-01	1.19E-02	8.16E-01	8.33E-01	
KO:K07038	0.00	0.00	0.00	1.12	0.00	0.00	0.00	1.00E+00	1.00E+00	1.00E+00	5.79E-02	1.00E+00	1.00E+00	1.00E+00	
KO:K07039	0.00	0.67	0.00	0.00	0.00	0.00	0.00	1.00E+00	3.27E-02	1.00E+00	1.00E+00	1.00E+00	1.00E+00	1.00E+00	
KO:K07040	8.02	0.23	0.72	0.65	6.20	3.85	2.63	5.33E-02	8.94E-01	8.16E-01	8.29E-01	8.91E-02	2.22E-01	3.88E-01	
KO:K07041	0.00	0.00	0.00	0.00	0.00	0.00	5.00	1.00E+00	1.00E+00	1.00E+00	1.00E+00	1.00E+00	1.00E+00	1.44E-02	
KO:K07042	1.23	1.69	2.70	0.88	3.21	1.43	0.16	5.18E-01	3.26E-01	1.25E-01	6.90E-01	8.34E-02	4.28E-01	9.48E-01	
KO:K07043	0.68	2.85	2.79	0.38	0.13	0.08	0.24	5.65E-01	5.40E-02	5.60E-02	7.61E-01	8.90E-01	9.19E-01	8.49E-01	
KO:K07044	1.66	0.00	1.54	0.18	0.00	0.00	0.00	8.67E-02	1.00E+00	9.67E-02	3.92E-01	1.00E+00	1.00E+00	1.00E+00	
KO:K07045	0.91	1.60	0.07	4.00	0.00	0.00	0.03	3.88E-01	2.62E-01	7.04E-01	9.29E-02	1.00E+00	1.00E+00	7.39E-01	
KO:K07046	0.91	2.48	8.35	2.90	1.20	0.00	1.91	7.25E-01	3.60E-01	4.30E-02	2.96E-01	6.52E-01	1.00E+00	4.80E-01	
KO:K07047	9.17	6.63	0.74	1.46	1.88	0.51	0.13	5.53E-02	1.11E-01	8.42E-01	7.21E-01	6.42E-01	8.77E-01	9.35E-01	
KO:K07048	0.32	0.00	0.00	0.44	0.00	0.00	0.00	1.58E-01	1.00E+00	1.00E+00	1.47E-01	1.00E+00	1.00E+00	1.00E+00	
KO:K07050	0.65	6.90	0.00	2.18	0.00	0.00	0.00	5.73E-01	2.44E-02	1.00E+00	1.78E-01	1.00E+00	1.00E+00	1.00E+00	
KO:K07051	0.04	0.00	4.11	0.16	2.85	0.00	1.44	5.64E-01	6.66E-01	5.07E-02	4.63E-01	7.86E-02	1.00E+00	1.53E-01	
KO:K07052	0.02	0.23	0.03	0.31	31.95	0.00	0.00	8.03E-01	6.67E-01	8.00E-01	6.21E-01	0.00E+00	1.00E+00	1.00E+00	
KO:K07053	2.08	2.48	0.72	1.65	6.16	0.14	0.57	3.81E-01	3.04E-01	7.50E-01	4.87E-01	6.31E-02	8.85E-01	7.86E-01	
KO:K07054	0.00	0.00	1.25	0.00	0.00	0.00	0.00	1.00E+00	1.00E+00	1.43E-02	1.00E+00	1.00E+00	1.00E+00	1.00E+00	
KO:K07055	0.00	0.00	0.00	0.00	0.00	0.00	2.08	1.00E+00	1.00E+00	1.00E+00	1.00E+00	1.00E+00	1.00E+00	1.80E-02	
KO:K07056	3.94	5.20	1.20	2.43	1.47	3.12	0.53	1.97E-01	8.73E-02	8.79E-01	5.58E-01	8.31E-01	3.49E-01	9.60E-01	
KO:K07058	1.92	0.16	0.77	0.37	14.99	1.86	0.00	2.24E-01	8.61E-01	5.93E-01	7.72E-01	2.57E-03	2.38E-01	9.49E-01	
KO:K07060	0.00	0.00	0.00	0.00	0.00	0.00	0.83	1.00E+00	1.00E+00	1.00E+00	1.00E+00	1.00E+00	1.00E+00	2.43E-02	
KO:K07061	0.06	0.05	0.00	0.00	0.00	0.00	0.00	1.25E-01	1.69E-01	1.00E+00	1.00E+00	1.00E+00	1.00E+00	1.00E+00	
KO:K07062	2.47	0.00	0.00	0.37	0.27	0.00	3.15	9.43E-02	7.51E-01	1.00E+00	4.71E-01	5.15E-01	1.00E+00	6.74E-02	
KO:K07064	0.00	0.00	0.00	0.00	0.00	0.00	3.75	1.00E+00	1.00E+00	1.00E+00	1.00E+00	1.00E+00	1.00E+00	2.74E-02	
KO:K07065	0.04	0.16	0.00	0.02	0.00	0.00	3.00	3.95E-01	2.95E-01	1.00E+00	4.30E-01	1.00E+00	1.00E+00	3.39E-02	
KO:K07066	0.00	0.00	0.00	0.00	0.00	0.00	0.42	1.00E+00	1.00E+00	1.00E+00	1.00E+00	1.00E+00	1.00E+00	2.46E-02	
KO:K07068	1.27	0.20	0.00	0.53	0.00	0.00	1.37	1.60E-01	4.92E-01	1.00E+00	3.21E-01	1.00E+00	1.00E+00	1.49E-01	
KO:K07069	0.00	0.00	0.00	0.00	1.88	0.00	0.00	1.00E+00	1.00E+00	1.00E+00	1.00E+00	1.66E-02	1.00E+00	1.00E+00	
KO:K07070	1.82	0.01	0.00	0.02	0.00	0.00	0.00	2.91E-02	5.61E-01	1.00E+00	5.15E-01	1.00E+00	1.00E+00	1.00E+00	
KO:K07071	3.21	0.29	13.60	0.37	9.10	0.90	1.69	2.26E-01	9.28E-01	4.86E-03	9.18E-01	1.66E-02	8.27E-01	6.02E-01	
KO:K07074	0.00	0.00	0.00	0.00	0.71	0.00	0.32	1.00E+00	1.00E+00	1.00E+00	3.35E-01	3.19E-02	1.00E+00	8.24E-02	
KO:K07075	0.13	0.01	0.00	0.67	0.00	0.00	0.00	2.59E-01	5.05E-01	1.00E+00	5.97E-02	1.00E+00	1.00E+00	1.00E+00	
KO:K07077	0.00	0.24	0.00	0.40	0.00	0.00	0.00	1.00E+00	1.54E-01	1.00E+00	1.02E-01	1.00E+00	1.00E+00	1.00E+00	
KO:K07079	0.00	0.00	0.07	0.00	12.54	0.00	0.93	1.00E+00	1.00E+00	4.86E-01	1.00E+00	1.86E-03	1.00E+00	1.19E-01	
KO:K07080	1.22	28.89	0.00	8.20	0.00	1.47	0.00	8.49E-01	2.43E-03	1.00E+00	1.22E-01	1.00E+00	8.24E-01	1.00E+00	
KO:K07081	0.00	0.00	2.73	0.03	5.48	0.00	0.13	1.00E+00	1.00E+00	3.77E-02	5.26E-01	1.33E-02	1.00E+00	3.89E-01	
KO:K07082	4.67	18.86	0.02	2.50	0.00	0.50	0.24	1.71E-01	1.00E-03	9.96E-01	6.26E-01	1.00E+00	9.61E-01	9.79E-01	
KO:K07083	0.00	0.00	1.57	0.05	8.53	0.00	2.56	1.00E+00	1.00E+00	1.26E-01	5.75E-01	1.16E-02	1.00E+00	7.30E-02	
KO:K07084	0.22	0.02	0.00	0.02	0.00	0.00	0.00	9.33E-02	3.03E-01	1.00E+00	3.25E-01	1.00E+00	1.00E+00	1.00E+00	
KO:K07086	0.28	0.40	0.08	0.24	3.45	0.00	0.33	4.93E-01	4.04E-01	7.34E-01	5.35E-01	2.84E-02	1.00E+00	4.59E-01	
KO:K07088	2.10	18.62	2.45	1.48	2.13	0.31	0.17	7.05E-01	4.86E-03	6.31E-01	8.17E-01	6.97E-01	9.49E-01	9.65E-01	
KO:K07089	0.87	4.09	1.99	0.66	3.49	0.00	0.15	5.77E-01	1.01E-01	2.81E-01	6.52E-01	1.29E-01	1.00E+00	8.64E-01	
KO:K07090	9.63	24.53	1.10	10.82	3.50	2.83	0.58	2.51E-01	3.57E-03	9.89E-01	1.62E-01	9.26E-01	9.51E-01	9.95E-01	
KO:K07091	5.95	8.44	0.83	1.23	0.00	1.26	0.18	7.50E-02	3.23E-02	8.51E-01	7.64E-01	1.00E+00	7.58E-01	9.55E-01	lipopolysaccharide export system permease protein
KO:K07093	0.15	1.91	6.18	2.35	0.31	0.00	0.18	7.56E-01	2.71E-01	4.91E-02	2.14E-01	6.91E-01	1.00E+00	7.43E-01	
KO:K07094	0.15	0.00	0.00	0.80	0.00	0.00	4.08	4.31E-01	1.00E+00	1.00E+00	1.85E-01	1.00E+00	1.00E+00	2.39E-02	
KO:K07095	0.64	0.08	0.00	0.69	2.18	0.00	1.57	3.36E-01	7.08E-01	1.00E+00	3.15E-01	9.26E-02	1.00E+00	1.38E-01	
KO:K07096	0.00	0.02	0.53	0.00	0.00	0.00	0.00	1.00E+00	2.37E-01	2.26E-02	1.00E+00	1.00E+00	1.00E+00	1.00E+00	
KO:K07098	1.89	0.88	3.32	0.96	0.00	0.25	0.48	2.23E-01	4.79E-01	1.03E-01	4.51E-01	1.00E+00	7.47E-01	6.52E-01	
KO:K07101	8.69	0.01	0.00	1.97	0.23	0.00	1.31	4.14E-02	7.91E-01	1.00E+00	2.82E-01	6.93E-01	1.00E+00	3.97E-01	
KO:K07102	3.36	0.49	0.25	3.19	0.00	4.01	0.45	1.05E-01	8.20E-01	8.88E-01	1.15E-01	1.00E+00	7.49E-02	8.32E-01	
KO:K07104	0.00	0.74	0.23	0.08	0.93	0.00	0.00	1.00E+00	1.35E-01	2.76E-01	3.87E-01	1.11E-01	1.00E+00	1.00E+00	
KO:K07106	0.50	0.40	0.01	1.09	9.11	4.69	0.10	6.65E-01	7.15E-01	9.13E-01	4.13E-01	1.63E-02	5.09E-02	8.45E-01	N-acetylmuramic acid 6-phosphate etherase [EC:4.2.-.-]
KO:K07107	7.52	7.73	4.99	3.72	0.49	0.28	0.20	7.51E-02	6.93E-02	2.18E-01	4.32E-01	9.68E-01	9.80E-01	9.86E-01	
KO:K07108	0.00	0.00	0.00	0.00	0.00	0.00	3.33	1.00E+00	1.00E+00	1.00E+00	1.00E+00	1.00E+00	1.00E+00	3.43E-03	

KO:K07109	0.12	0.00	0.00	0.00	0.00	0.00	0.00	6.26E-02	1.00E+00	1.00E+00	1.00E+00	1.00E+00	1.00E+00	1.00E+00	
KO:K07110	0.73	12.45	0.00	1.42	0.00	0.00	0.00	6.31E-01	7.00E-03	1.00E+00	3.85E-01	1.00E+00	1.00E+00	1.00E+00	
KO:K07112	7.30	22.29	0.13	7.53	0.00	11.10	0.02	2.79E-01	7.00E-03	9.87E-01	2.56E-01	1.00E+00	8.99E-02	9.91E-01	
KO:K07113	4.03	6.34	1.90	3.80	0.00	0.00	0.09	1.62E-01	6.46E-02	5.51E-01	1.84E-01	1.00E+00	1.00E+00	9.52E-01	
KO:K07114	8.88	0.59	7.03	2.66	1.15	0.30	0.91	3.41E-02	9.11E-01	6.27E-02	4.56E-01	8.28E-01	9.45E-01	8.66E-01	
KO:K07115	0.44	3.36	0.71	0.13	0.00	0.00	0.00	4.36E-01	2.64E-02	2.66E-01	7.39E-01	1.00E+00	1.00E+00	1.00E+00	
KO:K07116	8.56	0.45	0.00	0.39	0.00	0.00	0.43	3.07E-02	5.41E-01	1.00E+00	5.57E-01	1.00E+00	1.00E+00	5.45E-01	
KO:K07117	0.24	0.00	2.08	0.91	1.93	0.00	0.00	4.03E-01	1.00E+00	1.24E-01	2.33E-01	1.33E-01	1.00E+00	1.00E+00	
KO:K07118	1.18	0.00	0.00	0.02	0.00	0.00	0.08	4.93E-02	1.00E+00	1.00E+00	3.39E-01	1.00E+00	1.00E+00	2.37E-01	
KO:K07119	11.32	2.21	1.48	2.48	0.12	0.21	0.02	3.47E-02	4.49E-01	5.95E-01	4.05E-01	9.01E-01	8.73E-01	9.34E-01	
KO:K07120	0.30	16.91	0.00	1.10	0.83	0.00	0.00	8.01E-01	5.29E-03	1.00E+00	5.77E-01	6.59E-01	1.00E+00	1.00E+00	
KO:K07121	3.79	0.01	0.25	0.42	0.00	0.20	0.01	3.30E-02	8.09E-01	5.46E-01	4.13E-01	1.00E+00	5.93E-01	8.11E-01	
KO:K07122	0.12	0.00	0.00	0.10	0.00	0.00	0.00	1.27E-01	1.00E+00	1.00E+00	1.78E-01	1.00E+00	1.00E+00	1.00E+00	
KO:K07124	2.80	0.02	0.19	0.44	2.71	0.09	0.54	1.21E-01	7.70E-01	6.03E-01	4.70E-01	1.25E-01	6.92E-01	4.28E-01	
KO:K07126	2.54	2.27	0.59	1.52	0.00	0.59	0.07	1.87E-01	2.12E-01	5.82E-01	3.29E-01	1.00E+00	5.82E-01	8.15E-01	
KO:K07127	1.13	12.90	0.00	0.53	0.00	0.00	0.12	4.81E-01	7.00E-03	1.00E+00	7.26E-01	1.00E+00	1.00E+00	8.89E-01	5-hydroxyisourate hydrolase [EC:3.5.2.17]
KO:K07130	1.94	1.66	0.22	3.56	0.00	0.00	0.05	2.22E-01	2.68E-01	7.64E-01	8.59E-02	1.00E+00	1.00E+00	8.44E-01	
KO:K07131	0.00	0.22	0.00	0.00	0.00	0.00	0.00	1.00E+00	9.87E-02	1.00E+00	1.00E+00	1.00E+00	1.00E+00	1.00E+00	
KO:K07133	0.07	0.21	0.05	0.12	0.00	1.54	0.02	4.89E-01	3.04E-01	5.22E-01	3.94E-01	1.00E+00	3.81E-02	6.10E-01	
KO:K07136	0.33	0.55	0.03	3.81	0.00	0.00	0.00	4.58E-01	3.64E-01	6.84E-01	5.37E-02	1.00E+00	1.00E+00	1.00E+00	
KO:K07137	0.43	0.02	5.19	0.10	6.56	0.26	0.02	4.76E-01	8.53E-01	2.04E-02	7.80E-01	1.31E-02	6.11E-01	8.72E-01	
KO:K07139	0.00	0.00	4.26	0.00	0.00	0.00	0.00	3.96E-01	1.00E+00	2.71E-03	1.00E+00	1.00E+00	1.00E+00	1.00E+00	
KO:K07140	1.65	9.26	0.00	0.29	0.02	0.00	0.01	2.49E-01	1.16E-02	1.00E+00	7.51E-01	9.07E-01	1.00E+00	9.24E-01	
KO:K07141	0.77	7.43	0.01	2.95	0.03	0.97	0.03	7.20E-01	2.63E-02	9.53E-01	1.62E-01	9.46E-01	6.45E-01	9.46E-01	
KO:K07142	0.00	0.00	0.00	0.00	0.00	0.00	0.42	1.00E+00	1.00E+00	1.00E+00	1.00E+00	1.00E+00	1.00E+00	2.96E-02	
KO:K07143	0.00	0.00	0.00	0.00	0.00	0.00	1.25	1.00E+00	1.00E+00	1.00E+00	1.00E+00	1.00E+00	1.00E+00	2.37E-02	
KO:K07145	1.23	0.38	0.31	2.01	0.00	0.00	0.55	2.23E-01	5.10E-01	5.58E-01	1.36E-01	1.00E+00	1.00E+00	4.20E-01	
KO:K07146	8.74	7.53	1.45	5.25	2.57	0.64	0.75	6.09E-02	9.47E-02	9.32E-01	2.95E-01	8.23E-01	9.73E-01	9.67E-01	
KO:K07147	3.43	6.96	4.69	2.98	2.18	0.02	1.37	4.47E-01	8.21E-02	2.33E-01	5.50E-01	7.37E-01	9.87E-01	8.71E-01	
KO:K07148	2.35	0.00	0.00	1.21	0.00	0.00	0.00	7.59E-02	1.00E+00	1.00E+00	1.38E-01	1.00E+00	1.00E+00	1.00E+00	
KO:K07149	1.47	0.24	0.00	5.88	0.00	0.00	0.00	2.67E-01	5.39E-01	1.00E+00	5.49E-02	1.00E+00	1.00E+00	1.00E+00	
KO:K07150	0.00	0.00	0.00	0.00	0.63	0.00	0.00	1.00E+00	1.00E+00	1.00E+00	1.00E+00	1.53E-02	1.00E+00	1.00E+00	
KO:K07151	0.66	0.00	0.00	0.00	0.00	0.00	3.15	1.76E-01	1.00E+00	1.00E+00	1.00E+00	1.00E+00	1.00E+00	3.60E-02	dolichyl-diphosphooligosaccharide--protein glycosyltransferase
KO:K07152	4.62	6.38	3.64	3.18	1.29	5.14	1.29	2.97E-01	1.23E-01	4.96E-01	6.04E-01	9.19E-01	2.24E-01	9.19E-01	
KO:K07153	2.42	0.00	0.00	0.15	0.00	0.00	4.79	6.30E-02	8.50E-01	1.00E+00	6.19E-01	1.00E+00	1.00E+00	2.53E-02	
KO:K07154	0.09	0.05	0.18	0.04	3.37	1.28	0.08	5.40E-01	6.23E-01	4.27E-01	6.65E-01	2.64E-02	7.13E-02	5.58E-01	
KO:K07156	0.69	1.06	0.00	0.27	0.00	0.00	0.00	1.77E-01	1.10E-01	1.00E+00	3.59E-01	1.00E+00	1.00E+00	1.00E+00	
KO:K07157	4.53	15.77	0.00	4.71	0.53	0.13	0.00	2.43E-01	8.14E-03	1.00E+00	2.20E-01	9.29E-01	9.66E-01	9.82E-01	
KO:K07158	0.00	0.00	0.00	0.00	0.00	0.00	1.67	1.00E+00	1.00E+00	1.00E+00	1.00E+00	1.00E+00	1.00E+00	1.44E-02	
KO:K07159	0.00	0.00	0.00	0.00	0.00	0.00	2.08	1.00E+00	1.00E+00	1.00E+00	1.00E+00	1.00E+00	1.00E+00	2.57E-02	
KO:K07160	1.82	5.76	0.17	1.70	0.00	0.30	0.00	2.65E-01	2.70E-02	9.11E-01	2.99E-01	1.00E+00	8.62E-01	9.70E-01	
KO:K07161	3.51	4.32	0.00	2.93	0.00	0.00	0.00	1.19E-01	8.31E-02	1.00E+00	1.56E-01	1.00E+00	1.00E+00	1.00E+00	
KO:K07164	2.76	1.16	3.32	1.97	1.54	1.85	0.00	2.66E-01	5.70E-01	2.10E-01	3.77E-01	4.70E-01	4.02E-01	1.00E+00	
KO:K07165	0.40	0.00	0.00	0.22	0.00	0.00	0.00	8.93E-02	1.00E+00	1.00E+00	1.70E-01	1.00E+00	1.00E+00	1.00E+00	transmembrane sensor
KO:K07166	1.30	0.22	2.64	0.37	0.00	0.00	1.36	2.05E-01	5.94E-01	9.11E-02	5.05E-01	1.00E+00	1.00E+00	1.97E-01	ACT domain-containing protein
KO:K07167	8.49	20.09	0.04	1.09	0.00	0.00	0.17	6.53E-02	3.71E-03	9.54E-01	8.45E-01	1.00E+00	1.00E+00	9.43E-01	putative transcriptional regulator
KO:K07168	0.02	0.02	0.00	0.00	0.00	0.00	0.27	2.61E-01	2.71E-01	1.00E+00	1.00E+00	1.00E+00	1.00E+00	7.37E-02	CBS domain-containing membrane protein
KO:K07169	0.03	0.00	0.00	1.33	0.00	0.00	0.00	1.87E-01	1.00E+00	1.00E+00	1.15E-01	1.00E+00	1.00E+00	1.00E+00	FHA domain-containing protein
KO:K07170	0.44	13.24	0.00	0.12	0.00	1.32	0.00	7.52E-01	4.43E-03	1.00E+00	8.84E-01	1.00E+00	3.54E-01	1.00E+00	GAF domain-containing protein
KO:K07171	0.32	0.01	0.00	0.24	0.25	0.45	0.03	2.15E-01	6.88E-01	1.00E+00	2.63E-01	2.56E-01	1.52E-01	6.18E-01	
KO:K07172	0.25	0.00	0.00	0.01	0.00	0.35	2.68	2.42E-01	1.00E+00	1.00E+00	4.44E-01	1.00E+00	2.08E-01	4.37E-02	
KO:K07173	0.00	0.11	0.00	0.00	0.00	0.00	0.00	1.00E+00	1.04E-01	1.00E+00	1.00E+00	1.00E+00	1.00E+00	1.00E+00	S-ribosylhomocysteine lyase [EC:4.4.1.21]
KO:K07174	0.00	0.00	0.00	0.00	0.00	0.00	0.83	1.00E+00	1.00E+00	1.00E+00	1.00E+00	1.00E+00	1.00E+00	2.57E-02	Mn2+-dependent serine/threonine protein kinase [EC:2.7.1.-]
KO:K07175	3.49	0.10	1.48	0.92	9.27	0.00	0.66	1.12E-01	8.88E-01	3.95E-01	5.84E-01	1.96E-02	1.00E+00	6.84E-01	PhoH-like ATPase
KO:K07176	0.00	0.00	0.00	0.00	0.00	0.00	1.25	1.00E+00	1.00E+00	1.00E+00	1.00E+00	1.00E+00	1.00E+00	2.39E-02	putative serine/threonine protein kinase
KO:K07178	0.91	0.03	0.20	0.05	0.00	0.00	1.08	1.03E-01	6.57E-01	4.11E-01	6.16E-01	1.00E+00	1.00E+00	8.04E-02	RIO kinase 1 [EC:2.7.11.1]
KO:K07180	0.25	0.04	4.85	1.09	0.25	0.64	1.00	7.01E-01	8.65E-01	2.37E-02	2.46E-01	7.03E-01	4.36E-01	2.76E-01	serine protein kinase
KO:K07181	0.89	0.29	0.00	0.32	0.00	5.56	1.06	2.80E-01	5.13E-01	1.00E+00	4.97E-01	1.00E+00	3.37E-02	2.43E-01	putative signal transduction protein containing EAL and modified
KO:K07182	0.36	0.47	0.01	0.19	0.00	9.39	0.29	4.61E-01	3.98E-01	8.28E-01	5.79E-01	1.00E+00	7.71E-03	5.05E-01	CBS domain-containing protein
KO:K07183	0.00	0.00	0.00	0.01	0.00	0.00	5.53	1.00E+00	4.46E-01	1.00E+00	3.73E-01	1.00E+00	1.00E+00	5.00E-03	response regulator NasT
KO:K07184	4.22	0.08	0.00	0.09	0.00	0.00	0.17	2.46E-02	6.30E-01	1.00E+00	6.23E-01	1.00E+00	1.00E+00	5.28E-01	SH3 domain protein
KO:K07185	0.33	3.03	1.67	1.67	0.32	3.77	0.10	7.76E-01	1.51E-01	3.45E-01	3.45E-01	7.78E-01	1.03E-01	8.69E-01	tryptophan-rich sensory protein
KO:K07190	0.00	0.00	0.00	0.00	25.47	0.00	0.01	6.27E-01	1.00E+00	1.00E+00	6.15E-01	0.00E+00	1.00E+00	6.12E-01	phosphorylase kinase alpha/beta subunit
KO:K07192	0.10	2.07	1.87	0.19	1.56	0.00	0.28	7.07E-01	1.36E-01	1.53E-01	6.44E-01	1.90E-01	1.00E+00	5.84E-01	flotillin
KO:K07213	0.00	0.00	0.00	0.10	0.00	0.00	0.00	1.00E+00	1.00E+00	1.00E+00	1.43E-01	1.00E+00	1.00E+00	1.00E+00	
KO:K07214	0.30	0.08	12.83	1.37	1.22	0.00	0.00	6.12E-01	7.13E-01	8.86E-03	2.82E-01	3.09E-01	1.00E+00	1.00E+00	enterochelin esterase and related enzymes
KO:K07216	0.06	0.01	0.00	1.18	0.00	0.00	0.00	2.15E-01	3.26E-01	1.00E+00	1.05E-01	1.00E+00	1.00E+00	1.00E+00	hemerythrin
KO:K07219	0.61	0.00	0.00	0.01	0.00	0.00	0.00	7.16E-02	1.00E+00	1.00E+00	2.30E-01	1.00E+00	1.00E+00	1.00E+00	putative molybdopterin biosynthesis protein
KO:K07220	2.58	0.06	1.19	0.32	0.00	0.63	3.16	1.27E-01	7.58E-01	3.07E-01	6.08E-01	1.00E+00	4.72E-01	9.64E-02	hypothetical protein
KO:K07221	0.58	0.00	6.95	0.56	0.05	0.74	0.13	3.80E-01	1.00E+00	1.16E-02	3.91E-01	7.47E-01	3.15E-01	6.70E-01	outer membrane porin
KO:K07222	0.11	1.88	0.00	3.53	0.00	4.82	0.00	8.03E-01	1.88E-01	1.00E+00	8.20E-02	1.00E+00	5.06E-02	1.00E+00	putative flavoprotein involved in K+ transport
KO:K07223	0.03	0.01	0.98	0.00	0.00	0.00	0.00	2.80E-01	4.40E-01	1.51E-02	1.00E+00	1.00E+00	1.00E+00	1.00E+00	putative iron-dependent peroxidase
KO:K07224	0.00	0.11	0.00	0.00	0.00	0.00	0.00	1.00E+00	1.03E-01	1.00E+00	1.00E+00	1.00E+00	1.00E+00	1.00E+00	putative lipoprotein
KO:K07225	0.12	0.00	0.00	0.00	0.00	0.00	0.00	6.04E-02	1.00E+00	1.00E+00	1.00E+00	1.00E+00	1.00E+00	1.00E+00	putative hemin transport protein
KO:K07226	1.05	0.09	2.15	0.39	0.00	0.00	0.00	1.68E-01	5.18E-01	7.67E-02	3.02E-01	1.00E+00	1.00E+00	1.00E+00	hypothetical protein
KO:K07229	0.00	0.05	0.41	0.01	0.00	0.00	0.00	1.00E+00	2.09E-01	3.07E-02	3.59E-01	1.00E+00	1.00E+00	1.00E+00	
KO:K07231	0.05	2.25	0.00	0.75	0.00	0.00	0.00	6.91E-01	4.11E-02	1.00E+00	1.63E-01	1.00E+00	1.00E+00	1.00E+00	putative iron-regulated protein
KO:K07232	0.79	8.98	0.00	0.64	0.00	0.00	0.00	4.68E-01	9.86E-03	1.00E+00	5.39E-01	1.00E+00	1.00E+00	1.00E+00	cation transport protein ChaC
KO:K07233	0.06	0.01	0.82	0.02	0.00	0.00	0.00	2.68E-01	4.25E-01	3.70E-02	3.48E-01	1.00E+00	1.00E+00	1.00E+00	copper resistance protein B
KO:K07234	0.00	0.06	0.00	0.05	0.00	0.00	0.21	1.00E+00	2.34E-01	1.00E+00	2.48E-01	1.00E+00	1.00E+00	9.84E-02	uncharacterized protein involved in response to NO
KO:K07235	1.46	0.05	0.01	0.38	0.00	1.13	9.36	2.41E-01	7.66E-01	8.12E-01	5.50E-01	1.00E+00	3.01E-01	2.06E-02	tRNA 2-thiouridine synthesizing protein D [EC:2.8.1.-]
KO:K07236	0.50	0.00	0.00	0.41	0.00	0.11	10.33	3.22E-01	7.35E-01	1.00E+00	3.60E-01	1.00E+00	5.33E-01	1.69E-02	tRNA 2-thiouridine synthesizing protein C
KO:K07237	0.05	0.00	0.00	0.38	0.00	0.00	12.04	5.04E-01	6.41E-01	1.00E+00	3.22E-01	1.00E+00	1.00E+00	1.00E-02	tRNA 2-thiouridine synthesizing protein B
KO:K07238	2.13	0.74	0.01	0.20	4.03	3.79	0.00	1.99E-01	4.57E-01	8.13E-01	6.66E-01	8.44E-02	9.40E-02	1.00E+00	zinc transporter, ZIP family
KO:K07239	0.55	0.04	6.56	0.49	1.46	1.25	0.11	4.89E-01	8.67E-01	2.20E-02	5.24E-01	1.95E-01	2.37E-01	7.87E-01	heavy-metal exporter, HME family
KO:K07240	1.04	7.82	0.44	2.13	2.00	0.00	0.00	6.83E-01	2.53E-02	8.62E-01	3.36E-01	3.73E-01	1.00E+00	9.76E-01	chromate transporter
KO:K07241	0.00	0.00	0.00	0.00	2.76	0.00	0.05	1.00E+00	1.00E+00	1.00E+00	1.00E+00	1.74E-02	1.00E+00	1.68E-01	high-affinity nickel-transport protein
KO:K07242	0.00	0.00	0.00	0.00	3.13	0.00	0.00	1.00E+00	1.00E+00	1.00E+00	1.00E+00	1.70E-02	1.00E+00	1.00E+00	putative multicomponent Na+:H+ antiporter subunit B
KO:K07243	0.87	0.00	0.47	1.21	0.00	0.00	0.00	1.76E-01	1.00E+00	2.57E-01	1.28E-01	1.00E+00	1.00E+00	1.00E+00	high-affinity iron transporter
KO:K07245	0.55	1.62	0.00	0.12	0.00	0.00	0.00	1.99E-01	6.07E-02	1.00E+00	5.05E-01	1.00E+00	1.00E+00	1.00E+00	putative copper resistance protein D
KO:K07246	0.95	0.37	0.00	9.95	0.00	0.00	0.00	4.38E-01	6.09E-01	1.00E+00	2.87E-02	1.00E+00	1.00E+00	1.00E+00	tartrate dehydrogenase/decarboxylase / D-malate dehydrogenase
KO:K07248	0.12	0.44	0.00	0.00	0.00	0.00	0.00	2.20E-01	7.66E-02	1.00E+00	1.00E+00	1.00E+00	1.00E+00	1.00E+00	lactaldehyde dehydrogenase / glycolaldehyde dehydrogenase
KO:K07250	0.25	0.30	0.00	0.01	0.00	5.60	0.48	3.94E-01	3.59E-01	1.00E+00	6.56E-01	1.00E+00	1.13E-02	2.72E-01	4-aminobutyrate aminotransferase / (S)-3-amino-2-methylpropionate
KO:K07254	0.00	0.00	0.00	0.00	0.00	0.00	1.67	1.00E+00	1.00E+00	1.00E+00	1.00E+00	1.00E+00	1.00E+00	2.77E-02	hypothetical protein
KO:K07255	0.00	0.02	0.00	0.52	0.00	0.00	0.00	1.00E+00	2.03E-01	1.00E+00	1.09E-01	1.00E+00	1.00E+00	1.00E+00	taurine dehydrogenase small subunit [EC:1.4.2.-]
KO:K07256	0.00	0.11	0.00	0.00	0.00	0.00	0.00	1.00E+00	1.02E-01	1.00E+00	1.00E+00	1.00E+00	1.00E+00	1.00E+00	taurine dehydrogenase large subunit [EC:1.4.2.-]

KO:K07257	0.01	0.00	0.00	2.34	0.00	0.00	0.00	4.54E-01	1.00E+00	1.00E+00	4.09E-02	1.00E+00	1.00E+00	1.00E+00	spore coat polysaccharide biosynthesis protein SpsF
KO:K07258	9.31	6.75	0.75	4.29	0.00	5.33	1.84	5.69E-02	1.35E-01	9.49E-01	4.19E-01	1.00E+00	2.56E-01	8.69E-01	D-alanyl-D-alanine carboxypeptidase (penicillin-binding protein 5/6)
KO:K07259	0.16	2.59	0.00	0.52	6.96	1.58	0.00	8.76E-01	7.24E-02	1.00E+00	6.70E-01	6.57E-03	1.89E-01	1.00E+00	D-alanyl-D-alanine carboxypeptidase /
KO:K07260	1.87	0.07	0.00	0.55	8.51	0.00	0.00	1.37E-01	7.67E-01	1.00E+00	4.34E-01	1.17E-02	1.00E+00	1.00E+00	D-alanyl-D-alanine carboxypeptidase [EC:3.4.16.4]
KO:K07261	0.06	7.03	0.00	0.21	0.00	0.00	0.00	7.86E-01	1.03E-02	1.00E+00	6.07E-01	1.00E+00	1.00E+00	1.00E+00	penicillin-insensitive murein endopeptidase [EC:3.4.24.-]
KO:K07262	0.10	0.00	0.00	2.47	0.00	0.00	0.00	3.47E-01	1.00E+00	1.00E+00	5.04E-02	1.00E+00	1.00E+00	1.00E+00	D-alanyl-D-alanine endopeptidase (penicillin-binding protein 7)
KO:K07263	1.50	0.23	18.99	2.00	1.08	0.00	0.12	4.43E-01	8.13E-01	4.14E-03	3.38E-01	5.57E-01	1.00E+00	8.45E-01	zinc protease [EC:3.4.99.-]
KO:K07265	0.00	1.02	0.00	0.00	6.16	0.00	0.00	8.02E-01	8.71E-02	1.00E+00	1.00E+00	8.14E-03	1.00E+00	1.00E+00	capsular polysaccharide export protein
KO:K07266	0.02	0.27	0.00	0.15	4.62	0.00	0.00	6.19E-01	2.96E-01	1.00E+00	3.81E-01	1.46E-02	1.00E+00	1.00E+00	capsular polysaccharide export protein
KO:K07267	0.00	0.00	0.66	0.10	0.66	0.00	0.13	1.00E+00	1.00E+00	4.93E-02	3.28E-01	4.93E-02	1.00E+00	2.72E-01	porin
KO:K07270	0.00	2.13	0.00	0.03	0.00	0.00	0.32	1.00E+00	4.09E-02	1.00E+00	4.84E-01	1.00E+00	1.00E+00	2.42E-01	glycosyl transferase, family 25
KO:K07273	0.00	0.01	0.00	1.22	0.00	0.00	0.00	1.00E+00	2.81E-01	1.00E+00	4.21E-02	1.00E+00	1.00E+00	1.00E+00	lysozyme
KO:K07274	0.37	0.46	0.00	0.34	0.00	0.00	0.13	2.24E-01	1.87E-01	1.00E+00	2.42E-01	1.00E+00	1.00E+00	4.09E-01	outer membrane protein
KO:K07275	0.08	0.03	0.06	1.16	0.00	0.46	0.00	3.82E-01	5.27E-01	4.24E-01	9.86E-02	1.00E+00	1.98E-01	1.00E+00	outer membrane protein
KO:K07276	0.71	0.00	1.37	4.23	0.00	0.00	0.04	3.54E-01	1.00E+00	1.95E-01	5.16E-02	1.00E+00	1.00E+00	7.48E-01	hypothetical protein
KO:K07277	11.30	15.13	8.32	6.63	6.07	5.45	3.06	1.30E-01	2.37E-02	5.34E-01	8.13E-01	8.66E-01	9.10E-01	9.81E-01	outer membrane protein
KO:K07278	0.08	0.52	0.10	0.21	0.00	0.00	0.04	5.47E-01	8.07E-02	5.05E-01	3.03E-01	1.00E+00	1.00E+00	7.00E-01	outer membrane protein
KO:K07281	0.00	0.00	0.00	0.10	0.00	0.00	0.00	1.00E+00	1.00E+00	1.00E+00	1.43E-01	1.00E+00	1.00E+00	1.00E+00	
KO:K07282	0.08	0.01	0.00	0.63	0.00	0.00	0.00	2.56E-01	3.71E-01	1.00E+00	9.69E-02	1.00E+00	1.00E+00	1.00E+00	poly-gamma-glutamate synthesis protein (capsule biosynthesis
KO:K07283	0.10	0.20	0.00	0.00	0.00	0.00	0.00	1.79E-01	1.25E-01	1.00E+00	1.00E+00	1.00E+00	1.00E+00	1.00E+00	putative salt-induced outer membrane protein
KO:K07284	0.42	0.00	0.76	0.38	0.00	0.00	0.00	1.46E-01	1.00E+00	7.30E-02	1.68E-01	1.00E+00	1.00E+00	1.00E+00	sortase A
KO:K07285	0.16	0.00	0.00	0.03	0.00	0.00	0.00	8.56E-02	1.00E+00	1.00E+00	2.54E-01	1.00E+00	1.00E+00	1.00E+00	outer membrane lipoprotein
KO:K07287	9.45	0.00	0.00	1.23	0.00	0.00	2.51	1.87E-02	8.33E-01	1.00E+00	3.90E-01	1.00E+00	1.00E+00	1.80E-01	lipoprotein-34
KO:K07288	13.77	0.00	0.92	0.88	0.00	0.49	0.76	1.01E-02	1.00E+00	5.92E-01	6.05E-01	1.00E+00	7.23E-01	6.42E-01	uncharacterized membrane protein
KO:K07289	0.19	2.46	0.10	0.64	0.00	0.71	0.00	6.37E-01	5.76E-02	7.35E-01	3.30E-01	1.00E+00	2.98E-01	1.00E+00	AsmA protein
KO:K07290	0.38	0.00	0.00	0.11	0.00	0.00	0.00	9.23E-02	1.00E+00	1.00E+00	2.29E-01	1.00E+00	1.00E+00	1.00E+00	hypothetical protein
KO:K07300	0.00	0.00	0.00	0.06	6.85	0.00	0.00	1.00E+00	1.00E+00	1.00E+00	3.92E-01	1.43E-03	1.00E+00	1.00E+00	Ca2+:H+ antiporter
KO:K07301	3.17	15.29	0.90	2.74	9.07	4.60	1.40	7.89E-01	9.57E-03	9.64E-01	8.47E-01	7.63E-02	5.23E-01	9.43E-01	
KO:K07302	6.86	5.13	6.45	5.95	0.01	0.00	0.09	1.07E-01	1.97E-01	1.22E-01	1.47E-01	9.75E-01	1.00E+00	9.59E-01	isoquinoline 1-oxidoreductase, alpha subunit [EC:1.3.99.16]
KO:K07303	10.02	7.63	6.41	6.51	0.00	0.00	0.68	7.69E-02	1.54E-01	2.27E-01	2.18E-01	1.00E+00	1.00E+00	9.42E-01	isoquinoline 1-oxidoreductase, beta subunit [EC:1.3.99.16]
KO:K07304	4.41	8.53	3.44	5.96	9.76	0.76	0.81	5.91E-01	7.10E-02	7.91E-01	2.65E-01	4.40E-02	9.88E-01	9.87E-01	peptide-methionine (S)-S-oxide reductase [EC:1.8.4.11]
KO:K07305	2.46	5.53	0.20	3.95	7.89	1.14	2.56	6.77E-01	1.31E-01	9.84E-01	3.16E-01	4.71E-02	9.08E-01	6.51E-01	peptide-methionine (R)-S-oxide reductase [EC:1.8.4.12]
KO:K07308	0.63	24.36	0.00	0.89	0.00	0.00	0.06	7.82E-01	1.86E-03	1.00E+00	7.12E-01	1.00E+00	1.00E+00	8.84E-01	anaerobic dimethyl sulfoxide reductase subunit C (DMSO reductase
KO:K07313	0.03	2.96	1.37	0.17	5.98	0.00	0.02	8.79E-01	6.09E-02	2.08E-01	7.93E-01	1.51E-02	1.00E+00	8.82E-01	serine/threonine protein phosphatase 1 [EC:3.1.3.16]
KO:K07315	0.01	0.00	0.00	0.45	20.71	0.00	0.28	7.56E-01	1.00E+00	1.00E+00	3.97E-01	1.43E-04	1.00E+00	4.88E-01	sigma-B regulation protein RsbU (phosphoserine phosphatase)
KO:K07316	0.03	0.00	0.00	0.00	0.00	0.00	0.33	2.16E-01	1.00E+00	1.00E+00	1.00E+00	1.00E+00	1.00E+00	4.76E-02	adenine-specific DNA-methyltransferase [EC:2.1.1.72]
KO:K07318	0.07	0.00	0.00	0.01	0.00	0.00	0.23	1.85E-01	1.00E+00	1.00E+00	3.43E-01	1.00E+00	1.00E+00	9.43E-02	adenine-specific DNA-methyltransferase [EC:2.1.1.72]
KO:K07319	0.76	2.86	0.31	1.40	0.00	0.00	1.59	4.95E-01	1.33E-01	6.74E-01	3.12E-01	1.00E+00	1.00E+00	2.76E-01	putative adenine-specific DNA-methyltransferase [EC:2.1.1.72]
KO:K07320	7.50	0.06	0.72	0.50	0.00	2.28	0.32	2.33E-02	8.65E-01	5.80E-01	6.74E-01	1.00E+00	1.80E-01	7.61E-01	putative adenine-specific DNA-methyltransferase [EC:2.1.1.72]
KO:K07321	0.00	1.03	0.00	0.39	0.00	0.00	0.00	1.00E+00	1.15E-01	1.00E+00	2.10E-01	1.00E+00	1.00E+00	1.00E+00	CO dehydrogenase maturation factor
KO:K07322	0.00	0.00	0.00	0.20	0.00	0.00	0.00	1.00E+00	1.00E+00	1.00E+00	7.99E-02	1.00E+00	1.00E+00	1.00E+00	regulator of cell morphogenesis and NO signaling
KO:K07323	8.43	6.98	0.15	1.31	0.00	0.00	5.15	4.99E-02	7.99E-02	9.43E-01	7.86E-01	1.00E+00	1.00E+00	1.60E-01	putative toluene tolerance protein
KO:K07325	0.00	0.00	0.00	0.00	0.00	0.00	0.42	1.00E+00	1.00E+00	1.00E+00	1.00E+00	1.00E+00	1.00E+00	2.44E-02	archaeal flagellin FlaB
KO:K07334	0.04	0.02	0.00	0.05	0.31	0.55	0.14	5.33E-01	6.05E-01	1.00E+00	5.05E-01	1.32E-01	5.70E-02	2.77E-01	proteic killer suppression protein
KO:K07335	0.62	23.82	1.52	3.91	0.00	0.00	0.01	8.45E-01	4.57E-03	7.14E-01	3.12E-01	1.00E+00	1.00E+00	9.08E-01	basic membrane protein A and related proteins
KO:K07336	1.49	0.20	0.00	0.27	2.30	0.00	0.00	1.17E-01	5.08E-01	1.00E+00	4.49E-01	6.93E-02	1.00E+00	1.00E+00	PKHD-type hydroxylase [EC:1.14.11.-]
KO:K07337	0.05	0.00	0.74	0.24	0.51	0.00	4.40	5.81E-01	1.00E+00	2.03E-01	4.08E-01	2.79E-01	1.00E+00	3.04E-02	hypothetical protein
KO:K07338	0.00	0.72	0.00	0.22	0.00	0.00	0.00	1.00E+00	7.71E-02	1.00E+00	1.96E-01	1.00E+00	1.00E+00	1.00E+00	hypothetical protein
KO:K07339	0.00	0.00	0.00	0.00	0.52	0.00	0.92	1.00E+00	1.00E+00	1.00E+00	4.01E-01	5.33E-02	1.00E+00	2.76E-02	hypothetical protein
KO:K07340	1.70	0.00	4.50	0.03	0.00	0.00	0.05	8.30E-02	1.00E+00	2.44E-02	6.74E-01	1.00E+00	1.00E+00	6.42E-01	hypothetical protein
KO:K07341	1.65	1.71	0.00	0.18	0.00	0.00	0.14	1.34E-01	1.29E-01	1.00E+00	5.12E-01	1.00E+00	1.00E+00	5.46E-01	death on curing protein
KO:K07343	0.08	0.42	0.00	0.07	0.00	0.00	0.00	3.29E-01	8.79E-02	1.00E+00	3.71E-01	1.00E+00	1.00E+00	1.00E+00	DNA transformation protein and related proteins
KO:K07344	0.00	0.00	0.00	0.00	0.00	0.00	0.42	1.00E+00	1.00E+00	1.00E+00	1.00E+00	1.00E+00	1.00E+00	3.03E-02	type IV secretion system protein TrbL
KO:K07346	0.00	0.00	0.00	0.10	0.00	0.00	0.00	1.00E+00	1.00E+00	1.00E+00	1.43E-01	1.00E+00	1.00E+00	1.00E+00	fimbrial chaperone protein
KO:K07347	0.00	0.00	0.00	0.10	0.00	0.00	0.00	1.00E+00	1.00E+00	1.00E+00	1.43E-01	1.00E+00	1.00E+00	1.00E+00	outer membrane usher protein
KO:K07357	0.00	0.56	0.00	0.00	0.00	0.00	0.00	1.00E+00	6.86E-02	1.00E+00	1.00E+00	1.00E+00	1.00E+00	1.00E+00	type 1 fimbriae regulatory protein FimB
KO:K07386	3.05	0.07	0.37	0.57	1.21	0.42	0.00	7.39E-02	7.77E-01	5.50E-01	4.47E-01	2.42E-01	5.22E-01	1.00E+00	putative endopeptidase [EC:3.4.24.-]
KO:K07387	0.05	0.60	0.00	0.00	0.00	0.00	0.00	2.93E-01	6.46E-02	1.00E+00	1.00E+00	1.00E+00	1.00E+00	1.00E+00	putative metalloprotease [EC:3.4.24.-]
KO:K07389	0.00	0.00	0.00	1.12	0.00	0.00	0.00	1.00E+00	1.00E+00	1.00E+00	5.21E-02	1.00E+00	1.00E+00	1.00E+00	cytolysin-activating lysine-acyltransferase [EC:2.3.1.-]
KO:K07390	5.02	4.70	7.57	3.20	2.43	0.00	2.32	2.26E-01	2.64E-01	8.27E-02	5.33E-01	7.00E-01	1.00E+00	7.22E-01	monothiol glutaredoxin
KO:K07391	1.44	0.59	8.56	0.75	5.11	0.79	0.24	4.51E-01	8.28E-01	9.14E-03	7.62E-01	3.86E-02	7.48E-01	9.36E-01	magnesium chelatase family protein
KO:K07393	1.57	11.98	0.85	1.36	0.61	0.14	0.57	6.54E-01	8.86E-03	8.39E-01	7.14E-01	8.87E-01	9.50E-01	8.91E-01	putative glutathione S-transferase
KO:K07394	2.49	0.05	0.00	0.85	0.00	0.00	0.02	5.91E-02	6.22E-01	1.00E+00	1.96E-01	1.00E+00	1.00E+00	6.92E-01	SM-2-related protein
KO:K07395	2.66	1.31	0.00	0.71	0.00	4.32	0.51	1.42E-01	3.45E-01	1.00E+00	5.45E-01	1.00E+00	6.60E-02	6.40E-01	putative proteasome-type protease
KO:K07396	0.66	0.04	0.00	0.51	0.00	0.00	0.00	1.09E-01	5.33E-01	1.00E+00	1.39E-01	1.00E+00	1.00E+00	1.00E+00	putative protein-disulfide isomerase
KO:K07397	6.43	0.34	0.41	1.50	1.81	0.32	3.74	4.69E-02	8.88E-01	8.70E-01	5.50E-01	4.52E-01	8.92E-01	1.40E-01	putative redox protein
KO:K07398	0.00	0.00	0.00	0.00	0.00	0.00	1.67	1.00E+00	1.00E+00	1.00E+00	1.00E+00	1.00E+00	1.00E+00	2.64E-02	conserved protein with predicted RNA binding PUA domain
KO:K07399	0.00	0.00	0.00	0.11	25.68	0.00	0.00	6.43E-01	1.00E+00	1.00E+00	4.85E-01	0.00E+00	1.00E+00	1.00E+00	cytochrome c biogenesis protein
KO:K07400	15.59	0.59	0.64	0.22	0.00	0.00	3.30	9.86E-03	7.27E-01	7.15E-01	7.92E-01	1.00E+00	1.00E+00	2.33E-01	Fe/S biogenesis protein NfuA
KO:K07401	0.36	0.01	1.15	0.32	0.00	0.00	0.00	2.14E-01	5.93E-01	7.21E-02	2.31E-01	1.00E+00	1.00E+00	1.00E+00	selenoprotein W-related protein
KO:K07402	3.24	16.86	0.12	1.36	0.00	9.73	0.07	4.84E-01	8.00E-03	9.68E-01	8.48E-01	1.00E+00	4.47E-02	9.72E-01	xanthine dehydrogenase accessory factor
KO:K07403	0.16	0.00	25.30	0.32	0.00	0.00	0.05	5.42E-01	7.62E-01	4.29E-04	4.38E-01	1.00E+00	1.00E+00	7.55E-01	membrane-bound serine protease (ClpP class)
KO:K07404	0.08	0.00	28.19	0.10	1.32	0.00	1.13	6.99E-01	7.67E-01	2.86E-04	6.89E-01	2.52E-01	1.00E+00	2.89E-01	6-phosphogluconolactonase [EC:3.1.1.31]
KO:K07405	0.00	0.00	6.30	0.05	1.01	0.00	0.00	1.00E+00	1.00E+00	8.71E-03	4.19E-01	1.11E-01	1.00E+00	1.00E+00	alpha-amylase [EC:3.2.1.1]
KO:K07406	0.45	1.31	0.00	1.30	0.00	0.00	0.00	3.00E-01	1.85E-01	1.00E+00	1.85E-01	1.00E+00	1.00E+00	1.00E+00	alpha-galactosidase [EC:3.2.1.22]
KO:K07407	0.59	4.97	0.37	0.10	0.02	0.00	0.00	4.21E-01	2.76E-02	5.38E-01	7.36E-01	7.88E-01	1.00E+00	1.00E+00	alpha-galactosidase [EC:3.2.1.22]
KO:K07442	0.00	0.00	0.00	0.00	0.00	0.00	2.50	1.00E+00	1.00E+00	1.00E+00	1.00E+00	1.00E+00	1.00E+00	6.43E-03	tRNA (adenine-N1-)-methyltransferase [EC:2.1.1.36]
KO:K07443	2.68	0.24	0.00	0.46	1.53	0.00	0.00	4.86E-02	6.13E-01	1.00E+00	4.40E-01	1.21E-01	1.00E+00	1.00E+00	methylated-DNA-protein-cysteine methyltransferase related protein
KO:K07444	0.76	7.49	0.07	0.54	2.73	0.48	0.05	6.86E-01	1.83E-02	9.13E-01	7.66E-01	1.69E-01	7.86E-01	9.21E-01	putative N6-adenine-specific DNA methylase
KO:K07445	0.20	0.18	1.45	0.00	0.00	0.00	0.00	2.30E-01	2.36E-01	6.46E-02	1.00E+00	1.00E+00	1.00E+00	1.00E+00	putative DNA methylase
KO:K07446	0.00	0.00	0.00	0.00	0.00	0.00	0.83	1.00E+00	1.00E+00	1.00E+00	1.00E+00	1.00E+00	1.00E+00	7.14E-03	putative methyltransferase
KO:K07447	1.63	1.18	8.90	2.14	2.55	0.14	0.05	4.76E-01	6.52E-01	1.29E-02	3.07E-01	2.18E-01	9.69E-01	9.78E-01	putative holliday junction resolvase [EC:3.1.-.-]
KO:K07448	0.00	0.02	0.00	0.04	0.00	1.27	0.00	1.00E+00	3.15E-01	1.00E+00	2.80E-01	1.00E+00	7.29E-03	1.00E+00	restriction system protein
KO:K07453	0.00	0.11	0.00	0.00	0.00	0.00	0.00	1.00E+00	9.90E-02	1.00E+00	1.00E+00	1.00E+00	1.00E+00	1.00E+00	putative restriction endonuclease
KO:K07454	0.03	0.03	0.86	0.06	0.10	0.00	0.04	4.77E-01	4.86E-01	4.60E-02	3.91E-01	3.26E-01	1.00E+00	4.42E-01	putative restriction endonuclease
KO:K07456	0.36	0.58	0.00	0.54	9.04	0.33	0.02	5.96E-01	4.49E-01	1.00E+00	4.71E-01	7.00E-03	6.17E-01	8.34E-01	DNA mismatch repair protein MutS2
KO:K07457	0.01	0.00	1.14	0.00	0.00	0.00	0.00	2.41E-01	1.00E+00	1.83E-02	1.00E+00	1.00E+00	1.00E+00	1.00E+00	endonuclease III related protein
KO:K07458	0.22	0.03	0.26	0.01	0.00	0.00	0.00	1.05E-01	4.10E-01	1.01E-01	5.30E-01	1.00E+00	1.00E+00	1.00E+00	DNA mismatch endonuclease, patch repair protein [EC:3.1.-.-]
KO:K07459	0.01	0.01	0.00	0.64	0.00	0.00	0.00	2.21E-01	2.45E-01	1.00E+00	1.10E-01	1.00E+00	1.00E+00	1.00E+00	putative ATP-dependent endonuclease of the OLD family
KO:K07460	0.60	0.08	3.04	0.49	1.15	0.00	0.01	2.97E-01	8.10E-01	3.33E-02	3.67E-01	1.31E-01	1.00E+00	9.10E-01	putative endonuclease
KO:K07461	0.66	0.02	1.49	1.29	0.01	5.14	0.02	3.77E-01	8.86E-01	1.48E-01	1.79E-01	9.00E-01	1.86E-02	8.77E-01	putative endonuclease
KO:K07462	4.63	4.86	6.77	3.12	6.06	1.86	0.81	2.85E-01	2.47E-01	7.77E-02	6.82E-01	1.07E-01	9.10E-01	9.79E-01	single-stranded-DNA-specific exonuclease [EC:3.1.-.-]
KO:K07463	0.00	0.00	0.00	0.00	0.00	0.00	5.00	1.00E+00	1.00E+00	1.00E+00	1.00E+00	1.00E+00	1.00E+00	1.69E-02	archaea-specific RecJ-like exonuclease
KO:K07464	0.10	0.00	0.00	0.17	0.00	0.00	0.00	1.60E-01	1.00E+00	1.00E+00	1.48E-01	1.00E+00	1.00E+00	1.00E+00	putative RecB family exonuclease
KO:K07466	0.00	0.00	0.00	0.00	0.00	0.00	8.33	1.00E+00	1.00E+00	1.00E+00	1.00E+00	1.00E+00	1.00E+00	6.00E-03	replication factor A1
KO:K07473	0.00	0.00	0.00	0.10	0.00	0.00	0.00	1.00E+00	1.00E+00	1.00E+00	1.43E-01	1.00E+00	1.00E+00	1.00E+00	DNA-damage-inducible protein J

KO:K07477	0.00	0.00	0.00	0.00	0.00	0.00	1.67	1.00E+00	1.00E+00	1.00E+00	1.00E+00	1.00E+00	1.00E+00	1.69E-02	translin
KO:K07478	3.52	2.84	10.82	1.35	2.89	1.05	0.43	2.21E-01	3.85E-01	7.43E-03	8.53E-01	3.71E-01	9.02E-01	9.71E-01	putative ATPase
KO:K07479	0.58	0.10	0.00	0.00	0.00	0.00	0.00	8.73E-02	2.29E-01	1.00E+00	1.00E+00	1.00E+00	1.00E+00	1.00E+00	putative DNA topoisomerase
KO:K07480	0.35	0.00	0.00	0.00	0.00	0.00	0.00	6.41E-02	1.00E+00	1.00E+00	1.00E+00	1.00E+00	1.00E+00	1.00E+00	insertion element IS1 protein InsB
KO:K07481	0.02	0.02	0.00	0.03	6.56	0.00	0.00	4.13E-01	4.15E-01	1.00E+00	3.89E-01	7.57E-03	1.00E+00	1.00E+00	transposase, IS5 family
KO:K07482	0.00	0.00	0.00	0.41	0.00	0.00	0.00	1.00E+00	1.00E+00	1.00E+00	8.13E-02	1.00E+00	1.00E+00	1.00E+00	transposase, IS3 family
KO:K07483	1.24	2.64	0.42	0.16	0.00	0.00	0.00	1.69E-01	6.71E-02	4.37E-01	6.51E-01	1.00E+00	1.00E+00	1.00E+00	transposase
KO:K07484	0.34	0.44	0.03	0.00	0.00	4.84	0.01	3.10E-01	2.58E-01	6.61E-01	7.17E-01	1.00E+00	1.13E-02	7.01E-01	transposase
KO:K07485	0.00	0.00	0.00	0.00	0.00	0.00	5.42	1.00E+00	1.00E+00	1.00E+00	1.00E+00	1.00E+00	1.00E+00	2.70E-02	transposase
KO:K07486	0.04	0.00	1.16	0.00	1.16	0.00	0.19	4.98E-01	6.40E-01	4.43E-02	6.10E-01	4.43E-02	1.00E+00	2.47E-01	transposase
KO:K07487	0.00	0.00	10.00	0.00	0.00	0.00	0.00	1.00E+00	1.00E+00	1.46E-02	1.00E+00	1.00E+00	1.00E+00	1.00E+00	transposase
KO:K07489	0.13	0.05	0.00	0.00	0.00	3.79	0.00	2.70E-01	3.92E-01	1.00E+00	5.08E-01	1.00E+00	6.86E-03	1.00E+00	transposase
KO:K07491	0.09	0.12	0.67	0.07	0.30	0.00	0.00	4.34E-01	3.85E-01	6.63E-02	4.92E-01	1.93E-01	1.00E+00	1.00E+00	putative transposase
KO:K07492	0.03	0.01	0.00	3.34	0.00	0.00	0.00	4.18E-01	4.76E-01	1.00E+00	5.96E-02	1.00E+00	1.00E+00	1.00E+00	putative transposase
KO:K07493	0.06	0.05	0.00	0.01	0.77	0.00	0.00	2.81E-01	2.93E-01	1.00E+00	4.37E-01	4.76E-02	1.00E+00	1.00E+00	putative transposase
KO:K07494	0.01	0.00	0.00	0.00	0.34	0.00	0.15	3.18E-01	1.00E+00	1.00E+00	1.00E+00	4.93E-02	1.00E+00	1.04E-01	putative transposase
KO:K07495	1.63	0.00	0.00	0.01	0.00	0.00	0.00	7.06E-02	1.00E+00	1.00E+00	2.31E-01	1.00E+00	1.00E+00	1.00E+00	putative transposase
KO:K07496	0.06	0.00	0.00	0.05	0.00	0.00	0.00	1.05E-01	1.00E+00	1.00E+00	2.07E-01	1.00E+00	1.00E+00	1.00E+00	putative transposase
KO:K07497	6.73	0.71	2.93	2.31	0.32	0.00	0.17	4.74E-02	7.24E-01	2.00E-01	2.93E-01	8.45E-01	1.00E+00	8.95E-01	putative transposase
KO:K07498	1.91	0.03	0.00	0.05	0.00	0.00	0.00	3.54E-02	5.34E-01	1.00E+00	4.92E-01	1.00E+00	1.00E+00	1.00E+00	putative transposase
KO:K07499	0.00	0.00	0.00	0.10	0.00	0.00	0.00	1.00E+00	1.00E+00	1.00E+00	1.43E-01	1.00E+00	1.00E+00	1.00E+00	putative transposase
KO:K07501	0.29	0.14	13.58	0.93	0.01	0.60	0.00	6.49E-01	7.52E-01	5.57E-03	3.24E-01	8.59E-01	4.61E-01	1.00E+00	hypothetical protein
KO:K07504	0.24	0.00	0.14	0.00	0.00	0.00	0.56	1.64E-01	3.72E-01	2.09E-01	1.00E+00	1.00E+00	1.00E+00	1.04E-01	hypothetical protein
KO:K07505	0.06	0.05	0.00	0.00	0.00	0.00	0.00	1.24E-01	1.68E-01	1.00E+00	1.00E+00	1.00E+00	1.00E+00	1.00E+00	hypothetical protein
KO:K07506	0.00	0.00	0.89	0.15	0.00	0.00	0.00	1.00E+00	1.00E+00	2.67E-02	1.69E-01	1.00E+00	1.00E+00	1.00E+00	AraC family transcriptional regulator
KO:K07507	0.47	0.05	0.10	0.37	0.00	0.00	0.04	1.84E-01	3.68E-01	3.21E-01	2.13E-01	1.00E+00	1.00E+00	3.81E-01	putative Mg2+ transporter-C (MgtC) family protein
KO:K07508	0.00	0.00	0.00	0.20	0.00	0.00	0.00	1.00E+00	1.00E+00	1.00E+00	1.43E-01	1.00E+00	1.00E+00	1.00E+00	acetyl-CoA acyltransferase 2 [EC:2.3.1.16]
KO:K07512	0.01	0.00	8.52	0.00	0.00	0.00	0.00	2.40E-01	1.00E+00	1.49E-02	1.00E+00	1.00E+00	1.00E+00	1.00E+00	mitochondrial trans-2-enoyl-CoA reductase [EC:1.3.1.38]
KO:K07516	4.79	4.29	2.22	4.34	0.00	0.00	0.00	1.56E-01	1.85E-01	4.19E-01	1.82E-01	1.00E+00	1.00E+00	1.00E+00	3-hydroxyacyl-CoA dehydrogenase [EC:1.1.1.35]
KO:K07519	0.06	0.00	0.00	0.05	0.00	0.00	0.00	1.04E-01	1.00E+00	1.00E+00	2.10E-01	1.00E+00	1.00E+00	1.00E+00	phthalate 4,5-dioxygenase [EC:1.14.12.7]
KO:K07533	0.00	0.00	0.00	0.00	0.00	0.00	4.58	1.00E+00	1.00E+00	1.00E+00	1.00E+00	1.00E+00	1.00E+00	1.84E-02	foldase protein PrsA [EC:5.2.1.8]
KO:K07535	0.51	0.13	1.99	0.39	0.00	0.00	0.00	2.50E-01	4.13E-01	7.30E-02	2.81E-01	1.00E+00	1.00E+00	1.00E+00	2-hydroxycyclohexanecarboxyl-CoA dehydrogenase [EC:1.1.1.-]
KO:K07536	0.00	0.00	0.00	1.73	0.00	0.00	0.00	1.00E+00	1.00E+00	1.00E+00	1.43E-01	1.00E+00	1.00E+00	1.00E+00	2-ketocyclohexanecarboxyl-CoA hydrolase [EC:3.1.2.-]
KO:K07544	0.00	0.44	0.00	0.00	0.00	0.00	0.00	1.00E+00	1.02E-01	1.00E+00	1.00E+00	1.00E+00	1.00E+00	1.00E+00	benzylsuccinate CoA-transferase BbsF subunit [EC:2.8.3.15]
KO:K07552	1.93	13.91	0.94	3.50	0.00	0.14	0.14	6.33E-01	4.57E-03	8.67E-01	2.45E-01	1.00E+00	9.68E-01	9.68E-01	MFS transporter, DHA1 family, bicyclomycin/chloramphenicol
KO:K07558	0.00	0.00	0.00	0.00	0.00	0.00	1.67	1.00E+00	1.00E+00	1.00E+00	1.00E+00	1.00E+00	1.00E+00	1.71E-02	tRNA nucleotidyltransferase (CCA-adding enzyme) [EC:2.7.7.25
KO:K07559	0.00	0.04	0.00	0.13	0.00	0.00	0.00	1.00E+00	1.99E-01	1.00E+00	1.24E-01	1.00E+00	1.00E+00	1.00E+00	putative RNA 2'-phosphotransferase [EC:2.7.1.-]
KO:K07560	1.68	3.19	0.19	0.56	0.00	6.45	0.16	2.62E-01	6.90E-02	9.25E-01	7.90E-01	1.00E+00	1.07E-02	9.31E-01	D-tyrosyl-tRNA(Tyr) deacylase [EC:3.1.-.-]
KO:K07561	0.00	0.00	0.00	0.00	0.00	0.00	2.92	1.00E+00	1.00E+00	1.00E+00	1.00E+00	1.00E+00	1.00E+00	1.90E-02	diphthamide synthase subunit DPH2
KO:K07563	0.00	0.00	0.00	0.00	0.00	0.00	0.83	1.00E+00	1.00E+00	1.00E+00	1.00E+00	1.00E+00	1.00E+00	2.86E-02	ribosome biogenesis protein Nop1
KO:K07566	9.04	2.42	0.88	2.95	2.16	3.58	1.69	3.26E-02	6.90E-01	9.39E-01	5.43E-01	7.49E-01	3.88E-01	8.37E-01	putative translation factor
KO:K07567	2.67	0.13	0.44	1.85	7.82	2.55	0.71	2.10E-01	9.26E-01	8.25E-01	3.66E-01	2.76E-02	2.24E-01	7.38E-01	TdcF protein
KO:K07568	7.80	4.98	2.96	3.44	2.50	6.77	0.38	7.20E-02	2.94E-01	7.31E-01	6.24E-01	8.11E-01	1.16E-01	9.87E-01	S-adenosylmethionine:tRNA ribosyltransferase-isomerase [EC:5.-.-.-]
KO:K07569	0.00	0.00	0.00	0.00	0.00	0.00	5.42	1.00E+00	1.00E+00	1.00E+00	1.00E+00	1.00E+00	1.00E+00	1.80E-02	RNA-binding protein
KO:K07572	0.00	0.00	0.00	0.00	0.00	0.00	2.50	1.00E+00	1.00E+00	1.00E+00	1.00E+00	1.00E+00	1.00E+00	4.71E-03	putative nucleotide binding protein
KO:K07573	0.00	0.00	0.00	0.00	0.00	0.00	2.92	1.00E+00	1.00E+00	1.00E+00	1.00E+00	1.00E+00	1.00E+00	2.70E-02	exosome complex component CSL4
KO:K07574	6.48	0.15	0.57	0.52	0.29	0.00	0.74	2.57E-02	8.07E-01	5.96E-01	6.21E-01	7.38E-01	1.00E+00	5.18E-01	putative RNA-binding protein containing KH domain
KO:K07575	0.00	0.00	0.00	0.00	0.00	0.00	1.67	1.00E+00	1.00E+00	1.00E+00	1.00E+00	1.00E+00	1.00E+00	2.69E-02	PUA domain protein
KO:K07576	1.02	0.01	2.82	0.35	0.07	0.47	0.18	1.73E-01	8.53E-01	4.97E-02	4.53E-01	7.56E-01	3.68E-01	6.08E-01	metallo-beta-lactamase family protein
KO:K07577	0.27	1.23	0.18	0.29	8.21	0.81	0.78	8.08E-01	2.95E-01	8.56E-01	7.93E-01	7.43E-03	4.72E-01	4.88E-01	putative mRNA 3-end processing factor
KO:K07580	0.00	0.00	0.00	0.00	0.00	0.00	4.58	1.00E+00	1.00E+00	1.00E+00	1.00E+00	1.00E+00	1.00E+00	5.86E-03	hypothetical protein
KO:K07581	0.00	0.00	0.00	0.00	0.00	0.00	1.67	1.00E+00	1.00E+00	1.00E+00	1.00E+00	1.00E+00	1.00E+00	1.31E-02	hypothetical protein
KO:K07582	0.00	0.00	0.00	0.00	0.00	0.00	2.92	1.00E+00	1.00E+00	1.00E+00	1.00E+00	1.00E+00	1.00E+00	2.64E-02	hypothetical protein
KO:K07583	0.00	0.00	0.00	0.00	0.00	0.00	0.83	1.00E+00	1.00E+00	1.00E+00	1.00E+00	1.00E+00	1.00E+00	2.71E-02	hypothetical protein
KO:K07588	2.72	10.44	0.28	1.97	0.00	0.66	0.31	3.09E-01	1.49E-02	9.18E-01	4.98E-01	1.00E+00	8.53E-01	9.12E-01	LAO/AO transport system kinase [EC:2.7.-.-]
KO:K07589	0.34	0.64	0.07	0.13	0.00	3.29	0.53	4.13E-01	3.01E-01	5.74E-01	5.36E-01	1.00E+00	5.96E-02	3.36E-01	D-erythro-7,8-dihydroneopterin triphosphate epimerase [EC:5.-.-.-]
KO:K07636	3.12	3.99	1.01	2.47	3.80	3.60	0.09	2.99E-01	1.68E-01	8.74E-01	4.76E-01	1.90E-01	2.17E-01	9.89E-01	two-component system, OmpR family, phosphate regulon sensor
KO:K07637	1.81	0.11	0.00	0.03	0.00	9.29	0.00	1.05E-01	6.07E-01	1.00E+00	6.96E-01	1.00E+00	7.71E-03	1.00E+00	two-component system, OmpR family, sensor histidine kinase PhoQ
KO:K07638	0.43	7.88	0.00	1.56	3.30	0.00	0.00	7.61E-01	2.46E-02	1.00E+00	3.77E-01	1.27E-01	1.00E+00	1.00E+00	two-component system, OmpR family, osmolarity sensor histidine
KO:K07639	0.00	0.02	2.30	0.00	0.00	0.00	0.00	1.00E+00	3.44E-01	4.57E-03	1.00E+00	1.00E+00	1.00E+00	1.00E+00	two-component system, OmpR family, sensor histidine kinase RstB
KO:K07640	0.47	0.00	0.00	0.00	0.00	0.00	0.00	3.26E-02	1.00E+00	1.00E+00	1.00E+00	1.00E+00	1.00E+00	1.00E+00	two-component system, OmpR family, sensor histidine kinase CpxA
KO:K07641	0.00	0.00	0.38	0.00	0.00	0.00	0.17	1.00E+00	1.00E+00	3.00E-02	1.00E+00	1.00E+00	1.00E+00	8.00E-02	two-component system, OmpR family, sensor histidine kinase CreC
KO:K07642	0.52	0.40	0.00	0.34	0.00	0.00	0.00	1.79E-01	2.09E-01	1.00E+00	2.30E-01	1.00E+00	1.00E+00	1.00E+00	two-component system, OmpR family, sensor histidine kinase BaeS
KO:K07644	0.08	0.00	1.23	0.00	0.00	0.00	0.06	2.76E-01	1.00E+00	1.10E-02	5.33E-01	1.00E+00	1.00E+00	3.10E-01	two-component system, OmpR family, heavy metal sensor histidine
KO:K07645	2.36	0.00	0.00	0.08	0.00	0.00	0.00	3.61E-02	1.00E+00	1.00E+00	3.83E-01	1.00E+00	1.00E+00	1.00E+00	two-component system, OmpR family, sensor histidine kinase QseC
KO:K07646	0.04	0.00	0.00	0.13	0.00	0.00	0.00	1.64E-01	1.00E+00	1.00E+00	1.36E-01	1.00E+00	1.00E+00	1.00E+00	two-component system, OmpR family, sensor histidine kinase KdpD
KO:K07647	0.00	0.44	0.00	0.00	0.00	0.00	0.00	1.00E+00	3.54E-02	1.00E+00	1.00E+00	1.00E+00	1.00E+00	1.00E+00	two-component system, OmpR family, sensor histidine kinase TorS
KO:K07648	0.03	0.00	0.00	0.00	0.00	1.46	0.00	2.54E-01	1.00E+00	1.00E+00	1.00E+00	1.00E+00	5.29E-03	1.00E+00	two-component system, OmpR family, aerobic respiration control
KO:K07649	0.96	6.13	0.00	0.09	0.00	0.00	0.00	2.68E-01	2.89E-02	1.00E+00	7.06E-01	1.00E+00	1.00E+00	1.00E+00	two-component system, OmpR family, sensor histidine kinase TctE
KO:K07657	4.83	3.83	8.44	1.78	4.19	0.02	0.39	1.48E-01	2.57E-01	2.99E-02	7.58E-01	2.07E-01	9.92E-01	9.58E-01	two-component system, OmpR family, phosphate regulon response
KO:K07658	1.69	1.59	0.01	2.32	0.01	1.35	0.05	2.80E-01	3.00E-01	7.88E-01	1.92E-01	7.88E-01	3.46E-01	7.75E-01	two-component system, OmpR family, alkaline phosphatase synthesis
KO:K07659	0.32	3.28	0.09	1.68	5.53	0.00	0.00	8.01E-01	8.03E-02	8.96E-01	2.43E-01	2.70E-02	1.00E+00	9.56E-01	two-component system, OmpR family, phosphate regulon response
KO:K07660	4.58	0.16	0.02	0.32	0.00	2.04	0.00	4.60E-02	6.08E-01	7.32E-01	5.17E-01	1.00E+00	1.55E-01	1.00E+00	two-component system, OmpR family, response regulator PhoP
KO:K07661	0.06	0.01	1.65	0.01	0.00	0.00	0.47	3.97E-01	4.95E-01	2.43E-02	4.90E-01	1.00E+00	1.00E+00	1.22E-01	two-component system, OmpR family, response regulator RstA
KO:K07662	0.06	0.06	0.00	0.03	1.00	0.00	0.05	4.06E-01	4.24E-01	1.00E+00	5.13E-01	2.56E-02	1.00E+00	4.44E-01	two-component system, OmpR family, response regulator CpxR
KO:K07663	0.00	0.00	0.27	0.00	0.00	0.00	0.48	1.00E+00	1.00E+00	7.41E-02	1.00E+00	1.00E+00	1.00E+00	2.50E-02	two-component system, OmpR family, catabolic regulation response
KO:K07664	0.54	0.28	0.11	0.10	0.00	0.00	0.00	1.46E-01	2.41E-01	3.91E-01	3.98E-01	1.00E+00	1.00E+00	1.00E+00	two-component system, OmpR family, response regulator BaeR
KO:K07665	0.07	0.00	12.66	0.00	0.31	0.09	0.01	4.29E-01	1.00E+00	3.00E-03	6.67E-01	2.67E-01	4.10E-01	6.41E-01	two-component system, OmpR family, copper resistance phosphate
KO:K07666	0.00	0.00	0.00	0.00	0.00	0.00	0.42	1.00E+00	1.00E+00	1.00E+00	1.00E+00	1.00E+00	1.00E+00	2.71E-02	two-component system, OmpR family, response regulator QseB
KO:K07667	0.01	0.00	0.00	0.14	0.34	0.00	0.00	3.58E-01	1.00E+00	1.00E+00	1.61E-01	6.53E-02	1.00E+00	1.00E+00	two-component system, OmpR family, KDP operon response regulator
KO:K07668	0.01	0.00	0.60	0.26	0.00	0.00	0.07	4.28E-01	1.00E+00	8.27E-02	1.70E-01	1.00E+00	1.00E+00	2.77E-01	two-component system, OmpR family, response regulator VicR
KO:K07669	0.00	0.00	0.63	0.00	0.00	0.00	0.00	1.00E+00	1.00E+00	1.54E-02	1.00E+00	1.00E+00	1.00E+00	1.00E+00	two-component system, OmpR family, response regulator MprA
KO:K07670	0.12	0.00	0.00	0.00	0.00	0.00	0.00	6.47E-02	1.00E+00	1.00E+00	1.00E+00	1.00E+00	1.00E+00	1.00E+00	two-component system, OmpR family, response regulator MtrA
KO:K07673	0.14	0.00	0.00	0.29	0.00	0.00	3.67	3.12E-01	1.00E+00	1.00E+00	2.47E-01	1.00E+00	1.00E+00	3.64E-02	two-component system, NarL family, nitrate/nitrite sensor histidine
KO:K07675	0.00	0.00	0.00	0.82	0.00	0.00	0.00	1.00E+00	1.00E+00	1.00E+00	5.93E-02	1.00E+00	1.00E+00	1.00E+00	two-component system, NarL family, sensor histidine kinase UhpB
KO:K07677	0.32	0.00	0.00	0.06	0.00	0.00	0.00	9.90E-02	1.00E+00	1.00E+00	2.07E-01	1.00E+00	1.00E+00	1.00E+00	two-component system, NarL family, capsular synthesis sensor
KO:K07678	0.32	0.01	0.58	0.14	0.00	0.46	0.00	1.95E-01	7.06E-01	9.30E-02	3.79E-01	1.00E+00	1.24E-01	1.00E+00	two-component system, NarL family, sensor histidine kinase BarA
KO:K07684	0.58	0.00	0.00	1.15	0.00	0.00	5.77	3.35E-01	7.17E-01	1.00E+00	2.13E-01	1.00E+00	1.00E+00	2.34E-02	two-component system, NarL family, nitrate/nitrite response
KO:K07685	0.00	0.22	0.00	0.00	0.00	0.00	0.00	1.00E+00	7.07E-02	1.00E+00	1.00E+00	1.00E+00	1.00E+00	1.00E+00	two-component system, NarL family, nitrate/nitrite response
KO:K07686	0.12	0.00	0.00	0.00	0.00	0.00	0.00	6.04E-02	1.00E+00	1.00E+00	1.00E+00	1.00E+00	1.00E+00	1.00E+00	two-component system, NarL family, uhpT operon response regulator
KO:K07687	0.00	0.00	0.00	0.30	0.00	0.00	0.17	1.00E+00	1.00E+00	1.00E+00	1.14E-01	1.00E+00	1.00E+00	1.60E-01	two-component system, NarL family, captular synthesis response
KO:K07689	1.45	0.00	1.54	1.14	0.00	0.00	0.00	1.46E-01	1.00E+00	1.38E-01	1.80E-01	1.00E+00	1.00E+00	1.00E+00	two-component system, NarL family, invasion response regulator UvrY
KO:K07692	0.00	0.00	12.20	0.01	0.00	0.00	0.00	1.00E+00	1.00E+00	8.14E-03	2.54E-01	1.00E+00	1.00E+00	1.00E+00	two-component system, NarL family, response regulator DegU
KO:K07693	0.12	0.00	0.00	0.00	0.00	0.00	0.00	6.27E-02	1.00E+00	1.00E+00	1.00E+00	1.00E+00	1.00E+00	1.00E+00	two-component system, NarL family, response regulator DesR
KO:K07703	0.00	0.00	0.00	0.31	0.00	0.00	0.00	1.00E+00	1.00E+00	1.00E+00	1.43E-01	1.00E+00	1.00E+00	1.00E+00	two-component system, CitB family, response regulator DcuR
KO:K07708	6.89	3.78	0.72	1.88	0.00	2.27	0.32	2.91E-02	1.44E-01	8.88E-01	5.73E-01	1.00E+00	4.49E-01	9.46E-01	two-component system, NtrC family, nitrogen regulation sensor

KO:K07709	0.00	0.00	3.13	0.00	0.00	0.00	0.00	1.00E+00	1.00E+00	4.14E-03	1.00E+00	1.00E+00	1.00E+00	1.00E+00	two-component system, NtrC family, sensor histidine kinase HydH
KO:K07711	0.00	0.02	0.00	0.00	0.00	0.00	0.33	1.00E+00	2.21E-01	1.00E+00	1.00E+00	1.00E+00	1.00E+00	4.19E-02	two-component system, NtrC family, sensor histidine kinase YfhK
KO:K07712	4.33	3.66	8.03	2.44	1.68	2.93	0.79	2.73E-01	3.84E-01	5.67E-02	6.63E-01	8.19E-01	5.50E-01	9.25E-01	two-component system, NtrC family, nitrogen regulation response
KO:K07713	0.36	0.03	22.99	0.10	0.59	0.00	0.12	5.04E-01	7.70E-01	1.14E-03	6.78E-01	3.95E-01	1.00E+00	6.65E-01	two-component system, NtrC family, response regulator HydG
KO:K07714	0.01	0.00	0.08	0.05	1.99	0.00	0.04	4.15E-01	1.00E+00	3.08E-01	3.38E-01	2.16E-02	1.00E+00	3.61E-01	two-component system, NtrC family, response regulator AtoC
KO:K07715	0.16	0.19	0.00	0.36	0.00	0.00	0.37	3.50E-01	3.17E-01	1.00E+00	2.03E-01	1.00E+00	1.00E+00	1.92E-01	two-component system, NtrC family, response regulator YfhA
KO:K07716	0.06	0.00	0.00	1.36	0.00	0.00	0.00	3.50E-01	1.00E+00	1.00E+00	3.76E-02	1.00E+00	1.00E+00	1.00E+00	two-component system, cell cycle sensor histidine kinase PleC
KO:K07722	0.01	0.00	0.00	0.00	0.00	0.00	1.56	2.35E-01	1.00E+00	1.00E+00	1.00E+00	1.00E+00	1.00E+00	2.43E-02	CopG family transcriptional regulator, nickel-responsive regulator
KO:K07726	0.43	0.08	0.00	0.12	0.00	0.00	0.41	1.52E-01	4.25E-01	1.00E+00	3.57E-01	1.00E+00	1.00E+00	1.61E-01	putative transcriptional regulator
KO:K07727	0.19	0.13	0.00	0.03	0.00	0.80	0.00	2.15E-01	2.85E-01	1.00E+00	4.72E-01	1.00E+00	5.60E-02	1.00E+00	putative transcriptional regulator
KO:K07729	1.13	0.26	0.00	0.03	0.00	0.00	0.00	5.94E-02	2.54E-01	1.00E+00	5.24E-01	1.00E+00	1.00E+00	1.00E+00	putative transcriptional regulator
KO:K07732	0.00	0.00	0.00	0.00	0.00	0.00	2.08	1.00E+00	1.00E+00	1.00E+00	1.00E+00	1.00E+00	1.00E+00	2.74E-02	riboflavin kinase, archaea type [EC:2.7.1.161]
KO:K07733	0.01	1.29	0.00	0.02	0.00	0.00	0.00	3.80E-01	4.31E-02	1.00E+00	3.13E-01	1.00E+00	1.00E+00	1.00E+00	prophage regulatory protein
KO:K07734	0.25	2.97	0.00	0.00	0.00	0.00	1.43	4.77E-01	3.91E-02	1.00E+00	8.49E-01	1.00E+00	1.00E+00	9.86E-02	transcriptional regulator
KO:K07735	2.82	4.33	11.93	3.11	0.79	0.79	0.20	4.79E-01	1.72E-01	5.57E-03	3.91E-01	9.36E-01	9.36E-01	9.86E-01	putative transcriptional regulator
KO:K07736	0.72	24.25	0.00	3.70	0.00	0.00	0.00	8.18E-01	3.57E-03	1.00E+00	2.54E-01	1.00E+00	1.00E+00	1.00E+00	CarD family transcriptional regulator
KO:K07737	0.02	0.06	1.91	0.07	0.48	0.00	0.00	5.40E-01	4.21E-01	3.50E-02	4.07E-01	1.37E-01	1.00E+00	1.00E+00	putative transcriptional regulator
KO:K07738	4.57	5.82	11.35	2.81	8.19	0.06	0.64	3.71E-01	1.99E-01	2.87E-02	7.18E-01	7.64E-02	9.88E-01	9.61E-01	transcriptional repressor NrdR
KO:K07739	0.00	0.00	0.00	0.00	0.00	0.00	2.50	1.00E+00	1.00E+00	1.00E+00	1.00E+00	1.00E+00	1.00E+00	8.57E-03	elongator complex protein 3 [EC:2.3.1.48]
KO:K07740	1.67	0.24	0.00	0.21	0.00	0.00	0.21	6.81E-02	4.06E-01	1.00E+00	4.44E-01	1.00E+00	1.00E+00	4.35E-01	regulator of sigma D
KO:K07742	0.71	18.18	0.00	0.49	4.26	0.00	0.00	8.07E-01	3.29E-03	1.00E+00	8.51E-01	1.30E-01	1.00E+00	1.00E+00	hypothetical protein
KO:K07743	0.00	0.00	0.00	0.20	0.00	0.00	0.00	1.00E+00	1.00E+00	1.00E+00	1.43E-01	1.00E+00	1.00E+00	1.00E+00	hypothetical protein
KO:K07746	0.03	0.00	0.00	0.06	0.00	2.41	0.04	4.50E-01	1.00E+00	1.00E+00	3.62E-01	1.00E+00	2.43E-03	4.12E-01	hypothetical protein
KO:K07749	3.11	8.58	0.25	9.70	0.00	0.00	0.00	3.81E-01	6.56E-02	9.01E-01	4.99E-02	1.00E+00	1.00E+00	1.00E+00	formyl-CoA transferase [EC:2.8.3.16]
KO:K07751	0.20	0.00	0.36	0.00	0.00	0.00	0.00	1.52E-01	1.00E+00	1.02E-01	1.00E+00	1.00E+00	1.00E+00	1.00E+00	PepB aminopeptidase [EC:3.4.11.23]
KO:K07753	0.04	0.00	0.00	0.13	0.00	0.00	0.00	1.76E-01	1.00E+00	1.00E+00	1.45E-01	1.00E+00	1.00E+00	1.00E+00	peroxisomal trans-2-enoyl-CoA reductase [EC:1.3.1.38]
KO:K07769	0.00	0.00	0.00	0.00	29.38	0.00	0.00	1.00E+00	1.00E+00	1.00E+00	1.00E+00	0.00E+00	1.00E+00	1.00E+00	two-component system, OmpR family, sensor histidine kinase NblS
KO:K07771	1.05	0.00	0.00	0.00	0.00	0.00	0.00	6.21E-02	1.00E+00	1.00E+00	1.00E+00	1.00E+00	1.00E+00	1.00E+00	two-component system, OmpR family, response regulator BasR
KO:K07772	0.09	0.44	0.00	0.29	0.00	0.00	0.00	3.31E-01	1.50E-01	1.00E+00	1.97E-01	1.00E+00	1.00E+00	1.00E+00	two-component system, OmpR family, torCAD operon response regulator
KO:K07773	0.38	0.00	0.00	0.11	0.00	0.00	0.00	8.83E-02	1.00E+00	1.00E+00	2.26E-01	1.00E+00	1.00E+00	1.00E+00	two-component system, OmpR family, aerobic respiration control
KO:K07774	0.92	5.49	0.00	0.10	0.00	0.00	0.00	2.55E-01	1.77E-02	1.00E+00	7.39E-01	1.00E+00	1.00E+00	1.00E+00	two-component system, OmpR family, response regulator TctD
KO:K07775	0.12	0.00	0.00	0.00	0.00	0.00	0.00	6.27E-02	1.00E+00	1.00E+00	1.00E+00	1.00E+00	1.00E+00	1.00E+00	two-component system, OmpR family, response regulator ResD
KO:K07777	0.00	0.00	0.00	0.10	0.00	0.00	0.00	1.00E+00	1.00E+00	1.00E+00	1.43E-01	1.00E+00	1.00E+00	1.00E+00	two-component system, NarL family, sensor histidine kinase DegS
KO:K07782	0.00	11.35	0.00	0.00	0.00	0.00	0.00	1.00E+00	8.57E-04	1.00E+00	8.42E-01	1.00E+00	1.00E+00	1.00E+00	LuxR family transcriptional regulator
KO:K07785	0.00	1.49	0.00	0.18	0.00	0.00	0.00	1.00E+00	4.30E-02	1.00E+00	2.97E-01	1.00E+00	1.00E+00	1.00E+00	MFS transporter, NRE family, putaive nickel resistance protein
KO:K07787	1.94	0.16	4.99	0.75	0.03	0.00	1.16	1.77E-01	7.59E-01	4.50E-02	4.70E-01	8.62E-01	1.00E+00	3.30E-01	Cu(I)/Ag(I) efflux system membrane protein CusA
KO:K07793	0.70	34.41	0.00	8.53	0.00	0.00	0.53	9.05E-01	1.29E-03	1.00E+00	1.19E-01	1.00E+00	1.00E+00	9.17E-01	putative tricarboxylic transport membrane protein
KO:K07794	0.68	13.53	0.00	2.24	0.00	0.00	0.00	6.93E-01	6.86E-03	1.00E+00	2.73E-01	1.00E+00	1.00E+00	1.00E+00	putative tricarboxylic transport membrane protein
KO:K07795	0.77	29.26	0.00	9.78	0.00	0.00	0.00	8.79E-01	3.00E-03	1.00E+00	7.46E-02	1.00E+00	1.00E+00	1.00E+00	putative tricarboxylic transport membrane protein
KO:K07798	0.60	0.01	4.13	0.20	0.00	0.79	0.55	3.05E-01	8.31E-01	2.87E-02	5.47E-01	1.00E+00	2.40E-01	3.29E-01	Cu(I)/Ag(I) efflux system membrane protein CusB
KO:K07799	0.02	0.00	0.00	0.02	0.00	0.00	0.27	2.59E-01	1.00E+00	1.00E+00	2.85E-01	1.00E+00	1.00E+00	6.60E-02	putative multidrug efflux transporter MdtA
KO:K07806	0.00	0.00	0.00	0.31	0.00	0.00	0.00	1.00E+00	1.00E+00	1.00E+00	1.43E-01	1.00E+00	1.00E+00	1.00E+00	UDP-4-amino-4-deoxy-L-arabinose-oxoglutarate aminotransferase
KO:K07811	0.12	0.00	0.00	0.00	0.00	0.00	0.00	6.53E-02	1.00E+00	1.00E+00	1.00E+00	1.00E+00	1.00E+00	1.00E+00	trimethylamine-N-oxide reductase (cytochrome c) 1 [EC:1.7.2.3]
KO:K07812	2.12	3.54	0.00	0.90	0.00	0.00	0.00	1.99E-01	1.09E-01	1.00E+00	3.66E-01	1.00E+00	1.00E+00	1.00E+00	trimethylamine-N-oxide reductase (cytochrome c) 2 [EC:1.7.2.3]
KO:K07814	2.02	0.06	2.57	0.61	0.07	0.00	0.00	1.01E-01	7.00E-01	7.23E-02	3.35E-01	6.86E-01	1.00E+00	1.00E+00	putative two-component system response regulator
KO:K07823	0.70	0.00	0.00	0.17	0.00	0.00	0.00	9.36E-02	1.00E+00	1.00E+00	2.28E-01	1.00E+00	1.00E+00	1.00E+00	3-oxoadipyl-CoA thiolase [EC:2.3.1.174]
KO:K07824	0.00	1.44	0.00	0.00	0.00	0.00	0.00	1.00E+00	9.89E-02	1.00E+00	1.00E+00	1.00E+00	1.00E+00	1.00E+00	benzoate 4-monooxygenase [EC:1.14.13.12]
KO:K07862	1.12	0.01	0.17	0.00	0.00	0.00	0.00	5.06E-02	4.12E-01	1.97E-01	1.00E+00	1.00E+00	1.00E+00	1.00E+00	serine/threonine transporter
KO:K07978	0.00	0.00	0.00	0.00	19.38	0.00	0.00	1.00E+00	1.00E+00	1.00E+00	1.00E+00	0.00E+00	1.00E+00	1.00E+00	GntR family transcriptional regulator
KO:K07979	0.44	0.00	0.00	0.00	0.20	0.00	0.09	8.13E-02	1.00E+00	1.00E+00	1.00E+00	1.61E-01	1.00E+00	2.68E-01	GntR family transcriptional regulator
KO:K08068	0.01	3.78	0.00	0.00	0.00	0.00	0.00	2.89E-01	6.70E-02	1.00E+00	1.00E+00	1.00E+00	1.00E+00	1.00E+00	N-acetylglucosamine-6-phosphate 2-epimerase and phosphatase
KO:K08070	4.89	1.91	2.17	1.92	1.54	1.49	4.48	2.00E-01	5.98E-01	5.51E-01	5.95E-01	6.72E-01	6.83E-01	2.35E-01	2-alkenal reductase [EC:1.3.1.74]
KO:K08081	0.23	0.00	0.00	0.00	0.00	0.00	0.00	6.19E-02	1.00E+00	1.00E+00	1.00E+00	1.00E+00	1.00E+00	1.00E+00	tropine dehydrogenase [EC:1.1.1.26]
KO:K08082	0.40	0.00	0.58	0.06	0.00	0.00	0.00	9.19E-02	1.00E+00	5.50E-02	4.40E-01	1.00E+00	1.00E+00	1.00E+00	two-component system, LytT family, sensor histidine kinase AlgZ
KO:K08083	4.97	0.00	5.86	0.26	0.00	0.00	0.00	6.50E-02	1.00E+00	4.67E-02	5.10E-01	1.00E+00	1.00E+00	1.00E+00	two-component system, LytT family, response regulator AlgR
KO:K08084	4.79	0.01	0.00	0.29	0.00	0.21	0.05	2.84E-02	7.93E-01	1.00E+00	4.52E-01	1.00E+00	5.09E-01	6.98E-01	type IV fimbrial biogenesis protein FimT
KO:K08085	0.47	0.00	0.00	0.00	0.00	0.00	0.00	3.70E-02	1.00E+00	1.00E+00	1.00E+00	1.00E+00	1.00E+00	1.00E+00	type IV fimbrial biogenesis protein FimU
KO:K08092	0.00	0.00	0.00	0.71	0.00	0.00	0.00	1.00E+00	1.00E+00	1.00E+00	1.43E-01	1.00E+00	1.00E+00	1.00E+00	3-dehydro-L-gulonate 2-dehydrogenase [EC:1.1.1.13]
KO:K08093	0.03	0.00	0.00	4.06	0.00	0.00	0.00	3.33E-01	1.00E+00	1.00E+00	5.69E-02	1.00E+00	1.00E+00	1.00E+00	3-hexulose-6-phosphate synthase [EC:4.1.2.43]
KO:K08094	0.00	0.00	0.00	3.66	0.00	0.00	0.00	4.00E-01	4.09E-01	1.00E+00	6.37E-02	1.00E+00	1.00E+00	1.00E+00	6-phospho-3-hexuloisomerase [EC:5.3.1.27]
KO:K08096	0.00	0.00	0.00	0.00	0.00	0.00	1.25	1.00E+00	1.00E+00	1.00E+00	1.00E+00	1.00E+00	1.00E+00	7.00E-03	GTP cyclohydrolase IIa [EC:3.5.4.29]
KO:K08097	0.50	0.00	0.00	3.34	0.18	0.00	0.00	2.97E-01	1.00E+00	1.00E+00	6.83E-02	4.22E-01	1.00E+00	1.00E+00	phosphosulfolactate synthase [EC:4.4.1.19]
KO:K08100	0.00	0.00	0.00	0.00	0.00	0.00	0.42	1.00E+00	1.00E+00	1.00E+00	1.00E+00	1.00E+00	1.00E+00	2.81E-02	bilirubin oxidase [EC:1.3.3.5]
KO:K08138	0.13	0.35	0.00	0.08	0.00	5.45	0.00	4.50E-01	3.01E-01	1.00E+00	4.95E-01	1.00E+00	1.01E-02	1.00E+00	MFS transporter, SP family, xylose:H+ symportor
KO:K08139	0.77	0.08	0.00	0.68	0.00	0.00	0.00	1.51E-01	3.86E-01	1.00E+00	1.63E-01	1.00E+00	1.00E+00	1.00E+00	MFS transporter, SP family, sugar:H+ symporter
KO:K08151	0.50	6.73	1.06	0.29	0.10	0.00	0.79	6.70E-01	3.06E-02	4.40E-01	7.63E-01	8.44E-01	1.00E+00	5.40E-01	MFS transporter, DHA1 family, tetracycline resistance protein
KO:K08153	0.00	0.00	0.00	0.61	0.00	0.00	0.00	1.00E+00	1.00E+00	1.00E+00	1.43E-01	1.00E+00	1.00E+00	1.00E+00	MFS transporter, DHA1 family, multidrug resistance protein
KO:K08156	0.00	0.11	0.00	0.00	0.00	0.00	0.00	1.00E+00	9.74E-02	1.00E+00	1.00E+00	1.00E+00	1.00E+00	1.00E+00	MFS transporter, DHA1 family, arabinose polymer transporter
KO:K08166	0.00	0.00	0.00	0.10	0.00	0.00	0.00	1.00E+00	1.00E+00	1.00E+00	1.43E-01	1.00E+00	1.00E+00	1.00E+00	MFS transporter, DHA2 family, methylenomycin A resistance protein
KO:K08177	0.00	0.00	0.42	0.10	0.00	0.00	0.00	1.00E+00	1.00E+00	3.53E-02	1.56E-01	1.00E+00	1.00E+00	1.00E+00	MFS transporter, OFA family, oxalate/formate antiporter
KO:K08191	0.14	0.00	0.24	0.13	0.00	0.00	0.00	1.98E-01	1.00E+00	1.16E-01	2.02E-01	1.00E+00	1.00E+00	1.00E+00	MFS transporter, ACS family, hexuronate transporter
KO:K08193	4.30	0.00	0.00	0.00	0.00	0.00	0.00	5.91E-02	1.00E+00	1.00E+00	1.00E+00	1.00E+00	1.00E+00	1.00E+00	MFS transporter, ACS family, solute carrier family 17
KO:K08194	0.03	0.00	0.87	0.05	0.00	0.00	0.00	2.95E-01	1.00E+00	3.24E-02	2.68E-01	1.00E+00	1.00E+00	1.00E+00	MFS transporter, ACS family, D-galactonate transporter
KO:K08195	0.03	0.00	0.00	0.22	0.00	0.00	0.00	1.79E-01	1.00E+00	1.00E+00	1.43E-01	1.00E+00	1.00E+00	1.00E+00	MFS transporter, AAHS family, 4-hydroxybenzoate transporter
KO:K08217	0.00	0.00	0.00	0.20	0.00	0.00	0.00	1.00E+00	1.00E+00	1.00E+00	1.43E-01	1.00E+00	1.00E+00	1.00E+00	MFS transporter, DHA3 family, macrolide efflux protein
KO:K08218	10.45	0.16	1.16	5.44	0.00	0.92	0.45	2.31E-02	9.09E-01	7.17E-01	1.06E-01	1.00E+00	7.76E-01	8.61E-01	MFS transporter, PAT family, beta-lactamase induction signal
KO:K08223	0.01	0.20	0.00	0.17	0.00	0.00	0.00	3.64E-01	1.58E-01	1.00E+00	1.73E-01	1.00E+00	1.00E+00	1.00E+00	MFS transporter, FSR family, fosmidomycin resistance protein
KO:K08224	0.03	0.00	0.00	0.00	0.00	12.49	0.00	2.79E-01	1.00E+00	1.00E+00	3.40E-01	1.00E+00	1.43E-03	1.00E+00	MFS transporter, YNFM family, putative membrane transport protein
KO:K08225	0.00	0.00	0.00	0.00	1.88	0.00	0.21	1.00E+00	1.00E+00	1.00E+00	1.00E+00	1.61E-02	1.00E+00	1.46E-01	MFS transporter, ENTS family, enterobactin (siderophore) exporter
KO:K08226	1.15	8.48	0.00	1.38	6.29	0.00	0.00	5.78E-01	4.31E-02	1.00E+00	5.15E-01	7.76E-02	1.00E+00	1.00E+00	MFS transporter, BCD family, chlorophyll transporter
KO:K08234	2.34	0.00	1.52	0.06	0.00	0.00	0.00	7.94E-02	5.20E-01	1.22E-01	4.40E-01	1.00E+00	1.00E+00	1.00E+00	glyoxylase I family protein
KO:K08252	0.51	0.07	0.00	0.64	0.00	9.37	0.00	3.48E-01	6.64E-01	1.00E+00	2.89E-01	1.00E+00	6.43E-03	1.00E+00	receptor protein-tyrosine kinase [EC:2.7.1.1]
KO:K08253	0.00	0.00	0.00	2.76	0.00	0.00	0.00	1.00E+00	1.00E+00	1.00E+00	4.74E-02	1.00E+00	1.00E+00	1.00E+00	non-specific protein-tyrosine kinase [EC:2.7.1.2]
KO:K08256	0.04	0.00	0.00	3.11	0.00	0.00	0.13	4.83E-01	1.00E+00	1.00E+00	4.66E-02	1.00E+00	1.00E+00	3.91E-01	phosphatidylinositol alpha-mannosyltransferase [EC:2.4.1.57]
KO:K08261	0.28	12.22	0.00	1.13	0.00	0.00	0.00	6.87E-01	1.29E-02	1.00E+00	4.22E-01	1.00E+00	1.00E+00	1.00E+00	D-sorbitol dehydrogenase (acceptor) [EC:1.1.99.21]
KO:K08262	0.00	0.00	0.00	0.00	13.13	0.00	0.00	1.00E+00	1.00E+00	1.00E+00	1.00E+00	3.57E-03	1.00E+00	1.00E+00	delta12-fatty acid dehydrogenase [EC:1.14.99.33]
KO:K08276	0.00	0.00	0.00	0.00	1.25	0.00	0.00	1.00E+00	1.00E+00	1.00E+00	1.00E+00	1.71E-02	1.00E+00	1.00E+00	ecotin
KO:K08279	0.03	0.24	0.00	0.07	0.00	11.89	0.00	5.71E-01	3.54E-01	1.00E+00	5.11E-01	1.00E+00	4.29E-04	1.00E+00	carnitine operon protein CaiE
KO:K08281	0.09	9.24	0.81	0.15	0.11	0.00	0.36	8.77E-01	5.71E-03	4.87E-01	8.46E-01	8.62E-01	1.00E+00	7.42E-01	nicotinamidase/pyrazinamidase [EC:3.5.1.19 3.5.1.-]
KO:K08282	0.03	0.00	0.09	0.07	8.65	0.00	0.35	6.64E-01	1.00E+00	5.41E-01	5.68E-01	7.14E-04	1.00E+00	2.83E-01	non-specific serine/threonine protein kinase [EC:2.7.11.1]
KO:K08289	0.97	0.05	3.21	0.90	8.92	0.00	0.16	4.11E-01	8.46E-01	9.49E-02	4.36E-01	1.51E-02	1.00E+00	7.85E-01	phosphoribosylglycinamide formyltransferase 2 [EC:2.1.2.2]
KO:K08295	0.00	0.15	0.00	0.22	0.00	0.00	0.00	1.00E+00	1.70E-01	1.00E+00	1.29E-01	1.00E+00	1.00E+00	1.00E+00	2-aminobenzoate-CoA ligase [EC:6.2.1.32]
KO:K08296	2.32	0.55	0.18	0.76	2.33	3.27	0.20	1.36E-01	7.03E-01	8.92E-01	5.84E-01	1.35E-01	7.41E-02	8.81E-01	phosphohistidine phosphatase [EC:3.1.3.-]
KO:K08299	0.26	11.39	0.00	2.47	0.00	0.00	0.00	7.67E-01	1.81E-02	1.00E+00	2.05E-01	1.00E+00	1.00E+00	1.00E+00	carnitinyl-CoA dehydratase [EC:4.2.1.-]
KO:K08300	10.17	8.05	2.84	4.17	6.10	3.54	3.35	8.90E-02	1.98E-01	8.93E-01	7.73E-01	4.41E-01	8.39E-01	8.58E-01	ribonuclease E [EC:3.1.26.12]
KO:K08301	5.68	1.48	6.57	1.98	0.79	6.23	2.92	1.59E-01	7.90E-01	1.13E-01	7.02E-01	8.84E-01	1.28E-01	5.14E-01	ribonuclease G [EC:3.1.26.-]

KO:K08303	0.40	0.57	8.85	0.11	7.89	0.00	3.02	7.22E-01	6.52E-01	2.51E-02	8.28E-01	2.91E-02	1.00E+00	1.43E-01	putative protease [EC:3.4.-.-]
KO:K08304	0.10	3.75	0.00	1.81	0.00	0.17	0.00	7.98E-01	3.33E-02	1.00E+00	1.11E-01	1.00E+00	7.31E-01	1.00E+00	membrane-bound lytic murein transglycosylase A [EC:3.2.1.-]
KO:K08305	8.17	0.31	2.50	2.65	0.00	6.41	1.43	4.96E-02	9.08E-01	4.47E-01	4.16E-01	1.00E+00	8.81E-02	6.99E-01	membrane-bound lytic murein transglycosylase B [EC:3.2.1.-]
KO:K08306	0.03	0.04	1.93	0.00	0.00	0.00	0.86	4.20E-01	3.96E-01	3.87E-02	1.00E+00	1.00E+00	1.00E+00	8.69E-02	membrane-bound lytic murein transglycosylase C [EC:3.2.1.-]
KO:K08307	3.70	0.57	0.17	1.99	0.00	13.68	0.62	1.16E-01	8.23E-01	9.19E-01	3.52E-01	1.00E+00	4.86E-03	8.09E-01	membrane-bound lytic murein transglycosylase D [EC:3.2.1.-]
KO:K08309	0.92	8.76	0.32	0.85	4.78	0.88	0.11	7.75E-01	1.89E-02	9.05E-01	7.94E-01	8.33E-02	7.85E-01	9.52E-01	soluble lytic murein transglycosylase [EC:3.2.1.-]
KO:K08310	0.16	0.00	0.00	0.22	0.00	0.00	0.00	1.52E-01	1.00E+00	1.00E+00	1.25E-01	1.00E+00	1.00E+00	1.00E+00	dATP pyrophosphohydrolase [EC:3.6.1.-]
KO:K08311	4.42	6.14	0.02	1.71	0.00	4.04	3.71	1.62E-01	7.53E-02	9.88E-01	6.97E-01	1.00E+00	2.01E-01	2.37E-01	putative (di)nucleoside polyphosphate hydrolase [EC:3.6.1.-]
KO:K08312	0.14	0.01	0.00	0.00	0.00	0.00	5.53	2.84E-01	5.98E-01	1.00E+00	6.34E-01	1.00E+00	1.00E+00	1.26E-02	ADP-ribose diphosphatase [EC:3.6.1.-]
KO:K08316	1.00	0.10	0.76	0.58	0.00	1.35	0.04	1.62E-01	7.43E-01	2.29E-01	3.06E-01	1.00E+00	1.07E-01	8.08E-01	ribosomal RNA small subunit methyltransferase D [EC:2.1.1.171]
KO:K08318	0.00	0.00	0.00	0.10	0.00	0.00	0.00	1.00E+00	1.00E+00	1.00E+00	1.43E-01	1.00E+00	1.00E+00	1.00E+00	putative dehydrogenase [EC:1.1.-.-]
KO:K08319	0.00	3.11	0.00	0.00	0.00	0.00	0.00	1.00E+00	6.03E-02	1.00E+00	1.00E+00	1.00E+00	1.00E+00	1.00E+00	putative dehydrogenase [EC:1.1.-.-]
KO:K08320	0.00	0.00	0.00	0.00	0.00	0.00	0.42	1.00E+00	1.00E+00	1.00E+00	1.00E+00	1.00E+00	1.00E+00	2.41E-02	CTP pyrophosphohydrolase [EC:3.6.1.-]
KO:K08321	0.01	0.00	0.00	0.00	0.00	0.00	9.04	3.86E-01	3.99E-01	1.00E+00	1.00E+00	1.00E+00	1.00E+00	5.86E-03	putative autoinducer-2 (AI-2) aldolase [EC:4.1.2.-]
KO:K08323	0.82	0.16	0.00	2.14	0.00	0.00	0.00	2.26E-01	4.52E-01	1.00E+00	9.61E-02	1.00E+00	1.00E+00	1.00E+00	starvation sensing protein RspA
KO:K08325	0.00	0.00	0.00	1.12	0.00	0.00	0.00	1.00E+00	1.00E+00	1.00E+00	4.97E-02	1.00E+00	1.00E+00	1.00E+00	NADP-dependent alcohol dehydrogenase [EC:1.1.-.-]
KO:K08344	1.46	0.68	0.00	1.78	0.00	0.00	0.00	1.87E-01	3.28E-01	1.00E+00	1.59E-01	1.00E+00	1.00E+00	1.00E+00	suppressor for copper-sensitivity B
KO:K08351	0.00	0.23	0.00	0.20	0.00	0.00	0.00	1.00E+00	1.47E-01	1.00E+00	1.90E-01	1.00E+00	1.00E+00	1.00E+00	biotin sulfoxide reductase [EC:1.-.-.-]
KO:K08352	0.55	0.00	0.05	0.01	0.00	3.16	0.00	1.68E-01	1.00E+00	3.20E-01	4.29E-01	1.00E+00	2.87E-02	1.00E+00	thiosulfate reductase [EC:1.-.-.-]
KO:K08354	0.12	0.00	0.00	0.00	0.00	0.00	0.00	6.26E-02	1.00E+00	1.00E+00	1.00E+00	1.00E+00	1.00E+00	1.00E+00	thiosulfate reductase cytochrome b subunit
KO:K08355	0.00	0.00	0.00	0.20	0.00	0.00	0.00	1.00E+00	1.00E+00	1.00E+00	1.43E-01	1.00E+00	1.00E+00	1.00E+00	arsenite oxidase small subunit [EC:1.2.98.1]
KO:K08356	0.25	0.00	0.00	0.19	0.00	0.00	0.00	1.05E-01	1.00E+00	1.00E+00	2.10E-01	1.00E+00	1.00E+00	1.00E+00	arsenite oxidase large subunit [EC:1.2.98.1]
KO:K08363	0.47	0.00	0.00	0.00	0.00	0.00	0.00	6.31E-02	1.00E+00	1.00E+00	1.00E+00	1.00E+00	1.00E+00	1.00E+00	mercuric ion transport protein
KO:K08365	0.04	0.75	0.00	0.01	0.00	0.00	0.00	3.50E-01	4.74E-02	1.00E+00	4.65E-01	1.00E+00	1.00E+00	1.00E+00	MerR family transcriptional regulator, mercuric resistance operon
KO:K08369	0.12	0.00	0.00	0.00	0.00	0.00	0.00	5.81E-02	1.00E+00	1.00E+00	1.00E+00	1.00E+00	1.00E+00	1.00E+00	MFS transporter, putative metabolite:H+ symporter
KO:K08372	0.01	0.00	9.49	0.01	0.00	0.00	0.00	4.01E-01	1.00E+00	2.71E-03	3.89E-01	1.00E+00	1.00E+00	1.00E+00	putative serine protease PepD [EC:3.4.21.-]
KO:K08384	0.00	0.00	0.00	1.22	0.00	0.00	0.00	1.00E+00	1.00E+00	1.00E+00	1.43E-01	1.00E+00	1.00E+00	1.00E+00	stage V sporulation protein D (sporulation-specific
KO:K08479	0.00	0.00	0.00	0.00	13.75	0.00	0.00	1.00E+00	1.00E+00	1.00E+00	1.00E+00	0.00E+00	1.00E+00	1.00E+00	two-component system, OmpR family, clock-associated histidine kinase
KO:K08480	0.00	0.00	0.00	0.00	17.50	0.00	0.00	1.00E+00	1.00E+00	1.00E+00	1.00E+00	0.00E+00	1.00E+00	1.00E+00	circadian clock protein KaiA
KO:K08481	0.00	0.00	0.00	0.00	6.15	0.00	0.00	1.00E+00	1.00E+00	1.00E+00	4.06E-01	2.57E-03	1.00E+00	1.00E+00	circadian clock protein KaiB
KO:K08482	0.00	0.00	0.00	0.00	22.44	0.38	0.00	1.00E+00	1.00E+00	1.00E+00	5.95E-01	2.86E-04	2.69E-01	1.00E+00	circadian clock protein KaiC
KO:K08483	0.00	0.00	31.51	1.54	1.58	0.00	0.09	8.45E-01	8.46E-01	4.29E-04	2.72E-01	2.65E-01	1.00E+00	7.81E-01	phosphotransferase system, enzyme I, PtsI [EC:2.7.3.9]
KO:K08484	10.67	9.34	0.72	1.47	0.00	2.61	0.10	2.57E-02	3.51E-02	9.23E-01	8.34E-01	1.00E+00	6.05E-01	9.72E-01	phosphotransferase system, enzyme I, PtsP [EC:2.7.3.9]
KO:K08485	0.47	0.00	0.00	0.00	0.00	0.00	0.00	6.37E-02	1.00E+00	1.00E+00	1.00E+00	1.00E+00	1.00E+00	1.00E+00	phosphocarrier protein NPr
KO:K08567	0.12	0.00	0.00	0.00	0.00	0.00	0.00	6.16E-02	1.00E+00	1.00E+00	1.00E+00	1.00E+00	1.00E+00	1.00E+00	hydrogenase 2 maturation protease [EC:3.4.24.-]
KO:K08590	0.11	0.00	0.05	0.11	0.00	0.00	3.72	3.27E-01	1.00E+00	4.10E-01	3.29E-01	1.00E+00	1.00E+00	2.87E-02	carbon-nitrogen hydrolase family protein
KO:K08591	1.15	3.04	3.62	3.99	7.31	0.03	0.83	8.22E-01	3.22E-01	2.25E-01	1.77E-01	4.27E-02	9.84E-01	8.75E-01	glycerol-3-phosphate acyltransferase PlsY [EC:2.3.1.15]
KO:K08602	1.23	17.44	5.18	1.78	0.19	0.00	0.61	8.20E-01	6.14E-03	1.65E-01	7.17E-01	9.48E-01	1.00E+00	8.97E-01	oligoendopeptidase F [EC:3.4.24.-]
KO:K08604	0.00	0.00	0.00	2.35	0.00	0.00	0.00	1.00E+00	1.00E+00	1.00E+00	3.97E-02	1.00E+00	1.00E+00	1.00E+00	vibriolysin [EC:3.4.24.25]
KO:K08641	0.07	0.05	0.32	0.77	4.49	0.00	0.14	7.25E-01	7.42E-01	4.50E-01	2.14E-01	1.59E-02	1.00E+00	6.35E-01	D-alanyl-D-alanine dipeptidase [EC:3.4.13.-]
KO:K08642	0.00	0.00	0.00	0.10	0.00	0.00	0.00	1.00E+00	1.00E+00	1.00E+00	1.43E-01	1.00E+00	1.00E+00	1.00E+00	LasA protease [EC:3.4.24.-]
KO:K08646	0.00	0.00	0.00	0.10	0.00	0.00	0.00	1.00E+00	1.00E+00	1.00E+00	1.43E-01	1.00E+00	1.00E+00	1.00E+00	peptidyl-Lys metalloendopeptidase [EC:3.4.24.2]
KO:K08651	0.44	0.00	0.00	0.82	0.00	0.00	0.00	1.63E-01	1.00E+00	1.00E+00	1.46E-01	1.00E+00	1.00E+00	1.00E+00	thermitase [EC:3.4.21.66]
KO:K08675	1.74	0.00	0.00	0.00	0.00	0.00	0.00	6.06E-02	1.00E+00	1.00E+00	1.00E+00	1.00E+00	1.00E+00	1.00E+00	Lon-like ATP-dependent protease [EC:3.4.21.-]
KO:K08676	0.12	0.00	0.00	0.05	0.00	11.91	0.08	3.72E-01	6.42E-01	1.00E+00	4.73E-01	1.00E+00	4.71E-03	4.12E-01	tricorn protease [EC:3.4.21.-]
KO:K08678	0.00	0.00	0.00	0.92	0.00	0.00	0.00	1.00E+00	1.00E+00	1.00E+00	1.43E-01	1.00E+00	1.00E+00	1.00E+00	UDP-glucuronate decarboxylase [EC:4.1.1.35]
KO:K08680	0.15	0.00	0.00	0.11	6.87	0.00	0.00	3.37E-01	1.00E+00	1.00E+00	3.56E-01	1.04E-02	1.00E+00	1.00E+00	2-succinyl-6-hydroxy-2,4-cyclohexadiene-1-carboxylate synthase
KO:K08681	0.00	0.00	0.00	0.00	0.00	0.00	7.92	1.00E+00	1.00E+00	1.00E+00	1.00E+00	1.00E+00	1.00E+00	5.00E-03	glutamine amidotransferase [EC:2.6.-.-]
KO:K08684	3.30	0.00	0.00	0.17	0.00	0.00	0.00	4.97E-02	1.00E+00	1.00E+00	3.35E-01	1.00E+00	1.00E+00	1.00E+00	methane monooxygenase [EC:1.14.13.25]
KO:K08685	8.54	0.05	0.00	0.01	0.00	0.00	0.00	2.07E-02	5.07E-01	1.00E+00	5.73E-01	1.00E+00	1.00E+00	1.00E+00	amine dehydrogenase [EC:1.4.99.3]
KO:K08686	0.01	0.00	0.00	1.63	0.00	0.00	0.00	2.77E-01	1.00E+00	1.00E+00	7.97E-02	1.00E+00	1.00E+00	1.00E+00	2-chlorobenzoate 1,2-dioxygenase [EC:1.14.12.13]
KO:K08687	6.48	2.08	0.00	0.00	0.00	0.00	0.00	3.67E-02	1.59E-01	1.00E+00	7.25E-01	1.00E+00	1.00E+00	1.00E+00	N-carbamoylsarcosine amidase [EC:3.5.1.59]
KO:K08688	0.05	27.17	0.00	0.53	0.00	0.00	0.00	8.74E-01	7.14E-04	1.00E+00	7.58E-01	1.00E+00	1.00E+00	1.00E+00	creatinase [EC:3.5.3.3]
KO:K08689	0.00	0.00	0.00	1.94	0.00	0.00	0.00	1.00E+00	1.00E+00	1.00E+00	1.43E-01	1.00E+00	1.00E+00	1.00E+00	biphenyl 2,3-dioxygenase [EC:1.14.12.18]
KO:K08691	0.31	3.12	0.00	0.14	0.00	0.00	0.00	3.55E-01	4.31E-02	1.00E+00	4.90E-01	1.00E+00	1.00E+00	1.00E+00	malyl-CoA lyase [EC:4.1.3.24]
KO:K08692	0.02	0.00	0.00	0.42	0.00	0.00	0.00	1.80E-01	1.00E+00	1.00E+00	1.22E-01	1.00E+00	1.00E+00	1.00E+00	malate-CoA ligase subunit alpha [EC:6.2.1.9]
KO:K08697	0.00	0.00	0.00	1.33	0.00	0.00	0.00	1.00E+00	1.00E+00	1.00E+00	1.43E-01	1.00E+00	1.00E+00	1.00E+00	carbon dioxide concentrating mechanism protein CcmL
KO:K08700	0.00	0.00	0.00	0.00	8.75	0.00	0.00	1.00E+00	1.00E+00	1.00E+00	1.00E+00	1.66E-02	1.00E+00	1.00E+00	carbon dioxide concentrating mechanism protein CcmO
KO:K08714	2.56	5.93	2.13	1.88	0.00	0.67	0.13	3.04E-01	7.09E-02	3.87E-01	4.44E-01	1.00E+00	7.91E-01	9.13E-01	voltage-gated sodium channel
KO:K08715	0.11	0.01	0.00	0.00	0.36	0.00	0.00	1.73E-01	3.02E-01	1.00E+00	1.00E+00	6.94E-02	1.00E+00	1.00E+00	inward rectifier potassium channel
KO:K08720	0.33	19.84	0.00	3.64	0.00	0.00	0.00	8.32E-01	7.86E-03	1.00E+00	2.15E-01	1.00E+00	1.00E+00	1.00E+00	outer membrane protein OmpU
KO:K08722	0.34	0.00	0.00	0.00	0.00	0.00	0.17	5.93E-02	1.00E+00	1.00E+00	1.00E+00	1.00E+00	1.00E+00	1.47E-01	5'-nucleotidase [EC:3.1.3.5]
KO:K08723	0.00	0.02	0.00	0.32	0.00	0.00	0.00	1.00E+00	2.01E-01	1.00E+00	1.24E-01	1.00E+00	1.00E+00	1.00E+00	5'-nucleotidase [EC:3.1.3.5]
KO:K08726	1.30	0.01	0.05	0.85	0.00	0.00	0.00	1.36E-01	5.42E-01	4.31E-01	1.81E-01	1.00E+00	1.00E+00	1.00E+00	soluble epoxide hydrolase [EC:3.3.2.1]
KO:K08738	1.52	33.07	0.00	6.92	0.00	0.00	0.00	8.14E-01	1.00E-03	9.64E-01	1.50E-01	1.00E+00	1.00E+00	1.00E+00	cytochrome c
KO:K08776	0.00	0.00	0.00	0.00	0.00	0.00	2.50	1.00E+00	1.00E+00	1.00E+00	1.00E+00	1.00E+00	1.00E+00	2.67E-02	puromycin-sensitive aminopeptidase [EC:3.4.11.-]
KO:K08884	0.00	0.00	24.93	0.37	11.41	0.00	0.65	8.37E-01	1.00E+00	1.43E-03	5.91E-01	2.11E-02	1.00E+00	4.90E-01	serine/threonine protein kinase, bacterial [EC:2.7.11.1]
KO:K08902	0.00	0.00	0.00	0.00	27.50	0.00	0.00	1.00E+00	1.00E+00	1.00E+00	1.00E+00	0.00E+00	1.00E+00	1.00E+00	photosystem II Psb27 protein
KO:K08903	0.00	0.00	0.00	0.00	28.13	0.00	0.00	1.00E+00	1.00E+00	1.00E+00	1.00E+00	4.29E-04	1.00E+00	1.00E+00	photosystem II reaction center 13kDa protein
KO:K08906	0.00	0.00	0.00	0.00	28.75	0.00	0.00	1.00E+00	1.00E+00	1.00E+00	1.00E+00	0.00E+00	1.00E+00	1.00E+00	cytochrome c6
KO:K08921	0.00	0.00	0.00	0.00	1.25	0.00	0.00	1.00E+00	1.00E+00	1.00E+00	1.00E+00	1.47E-02	1.00E+00	1.00E+00	chlorophyll a/b binding light-harvesting protein PcbD
KO:K08926	2.08	6.52	0.00	2.22	0.00	0.00	0.00	2.83E-01	6.66E-02	1.00E+00	2.65E-01	1.00E+00	1.00E+00	1.00E+00	light-harvesting complex 1 alpha chain
KO:K08927	0.18	8.88	0.00	1.47	0.00	0.00	0.00	6.88E-01	2.81E-02	1.00E+00	2.90E-01	1.00E+00	1.00E+00	1.00E+00	light-harvesting complex 1 beta chain
KO:K08928	1.87	9.39	0.00	2.08	0.00	0.00	0.00	3.37E-01	2.21E-02	1.00E+00	3.02E-01	1.00E+00	1.00E+00	1.00E+00	photosynthetic reaction center L subunit
KO:K08929	1.94	8.49	0.00	2.11	0.00	0.00	0.00	3.10E-01	3.23E-02	1.00E+00	2.86E-01	1.00E+00	1.00E+00	1.00E+00	photosynthetic reaction center M subunit
KO:K08930	0.52	0.04	0.00	0.48	0.00	0.00	0.00	1.60E-01	3.66E-01	1.00E+00	1.70E-01	1.00E+00	1.00E+00	1.00E+00	light-harvesting protein B-8-85 alpha chain
KO:K08939	0.25	0.07	0.00	0.76	0.00	0.00	0.00	2.13E-01	2.83E-01	1.00E+00	1.43E-01	1.00E+00	1.00E+00	1.00E+00	light-harvesting protein B-8-85 beta chain
KO:K08963	0.27	0.33	0.63	3.55	6.05	0.40	1.01	8.62E-01	8.45E-01	7.37E-01	7.23E-02	2.29E-02	8.21E-01	5.60E-01	methylthioribose-1-phosphate isomerase [EC:5.3.1.23]
KO:K08964	0.00	0.00	0.25	0.00	10.10	0.00	0.00	6.28E-01	1.00E+00	2.33E-01	6.29E-01	1.43E-04	1.00E+00	1.00E+00	methylthioribulose-1-phosphate dehydratase [EC:4.2.1.19]
KO:K08966	0.00	0.00	0.00	0.00	2.50	0.00	0.00	1.00E+00	1.00E+00	1.00E+00	1.00E+00	1.74E-02	1.00E+00	1.00E+00	2-hydroxy-3-keto-5-methylthiopentenyl-1-phosphate phosphatase
KO:K08967	0.00	0.00	2.35	0.00	11.01	0.00	0.00	6.02E-01	1.00E+00	4.66E-02	1.00E+00	1.00E-03	1.00E+00	1.00E+00	1,2-dihydroxy-3-keto-5-methylthiopentene dioxygenase [EC:1.13.11.53
KO:K08970	0.36	0.01	0.65	0.00	0.00	0.00	0.00	1.39E-01	4.21E-01	8.11E-02	4.28E-01	1.00E+00	1.00E+00	1.00E+00	nickel/cobalt exporter
KO:K08972	0.11	0.01	2.38	0.21	0.00	0.00	0.00	3.89E-01	6.36E-01	1.64E-02	2.77E-01	1.00E+00	1.00E+00	1.00E+00	putative membrane protein
KO:K08973	3.31	4.76	0.02	3.78	4.55	9.31	0.53	4.43E-01	2.03E-01	9.92E-01	3.45E-01	2.27E-01	3.36E-02	9.48E-01	putative membrane protein
KO:K08974	6.31	0.86	3.64	1.01	0.79	2.59	0.40	5.29E-02	7.64E-01	1.74E-01	7.24E-01	7.85E-01	3.16E-01	8.69E-01	putative membrane protein
KO:K08975	0.00	0.00	0.00	0.00	0.00	0.00	4.58	1.00E+00	1.00E+00	1.00E+00	1.00E+00	1.00E+00	1.00E+00	1.69E-02	putative membrane protein
KO:K08976	1.28	0.80	0.27	1.09	1.48	1.34	1.48	3.87E-01	5.52E-01	7.69E-01	4.45E-01	3.32E-01	3.70E-01	3.32E-01	putative membrane protein
KO:K08977	0.00	0.00	0.00	1.63	0.00	0.00	0.00	1.00E+00	1.00E+00	1.00E+00	7.87E-02	1.00E+00	1.00E+00	1.00E+00	putative membrane protein
KO:K08978	0.00	0.00	4.18	0.01	0.00	0.00	0.00	1.00E+00	1.00E+00	1.14E-03	3.40E-01	1.00E+00	1.00E+00	1.00E+00	putative membrane protein
KO:K08979	0.00	0.00	0.00	0.00	0.00	0.00	2.92	1.00E+00	1.00E+00	1.00E+00	1.00E+00	1.00E+00	1.00E+00	1.61E-02	putative membrane protein
KO:K08981	0.08	0.00	0.57	0.02	0.00	1.79	0.00	3.25E-01	1.00E+00	9.20E-02	4.72E-01	1.00E+00	1.53E-02	1.00E+00	putative membrane protein
KO:K08983	0.00	0.00	0.00	0.01	22.41	0.00	0.00	1.00E+00	5.77E-01	1.00E+00	5.75E-01	0.00E+00	1.00E+00	1.00E+00	putative membrane protein
KO:K08984	0.19	0.17	1.01	0.00	0.00	0.00	0.00	2.17E-01	2.25E-01	4.77E-02	1.00E+00	1.00E+00	1.00E+00	1.00E+00	putative membrane protein
KO:K08987	0.12	0.00	0.00	0.00	0.00	0.00	0.00	6.24E-02	1.00E+00	1.00E+00	1.00E+00	1.00E+00	1.00E+00	1.00E+00	putative membrane protein
KO:K08988	0.13	0.03	0.00	0.53	0.00	0.00	0.00	2.37E-01	3.59E-01	1.00E+00	1.19E-01	1.00E+00	1.00E+00	1.00E+00	putative membrane protein
KO:K08989	0.03	0.00	0.00	1.43	0.00	0.00	0.00	4.52E-01	1.00E+00	1.00E+00	3.67E-02	1.00E+00	1.00E+00	1.00E+00	putative membrane protein

KO:K08990	0.00	3.35	0.00	0.00	0.00	0.00	0.00	1.00E+00	1.07E-02	1.00E+00	6.68E-01	1.00E+00	1.00E+00	1.00E+00	putative membrane protein
KO:K08992	0.56	0.00	0.00	0.00	0.00	0.94	0.00	9.13E-02	1.00E+00	1.00E+00	1.00E+00	1.00E+00	5.24E-02	1.00E+00	putative membrane protein
KO:K08993	1.74	0.00	0.00	0.00	0.00	0.00	0.00	4.67E-02	1.00E+00	1.00E+00	1.00E+00	1.00E+00	1.00E+00	1.00E+00	putative membrane protein
KO:K08994	0.12	0.01	0.14	1.79	2.77	0.00	0.00	5.39E-01	7.37E-01	5.20E-01	8.93E-02	5.49E-02	1.00E+00	1.00E+00	putative membrane protein
KO:K08997	1.28	0.03	0.00	0.30	0.00	0.00	1.25	1.05E-01	6.30E-01	1.00E+00	3.84E-01	1.00E+00	1.00E+00	1.08E-01	hypothetical protein
KO:K08998	1.81	1.41	0.91	1.88	0.91	1.62	0.55	3.04E-01	4.43E-01	6.64E-01	2.89E-01	6.64E-01	3.64E-01	8.19E-01	hypothetical protein
KO:K08999	0.47	0.12	12.64	0.34	6.06	0.83	0.35	6.79E-01	8.32E-01	4.71E-03	7.38E-01	3.64E-02	5.23E-01	7.31E-01	hypothetical protein
KO:K09000	0.25	0.00	0.00	0.19	0.00	0.00	0.00	1.03E-01	1.00E+00	1.00E+00	2.10E-01	1.00E+00	1.00E+00	1.00E+00	hypothetical protein
KO:K09001	3.29	2.90	0.01	4.35	2.27	7.19	0.40	2.81E-01	3.82E-01	9.98E-01	1.36E-01	5.74E-01	3.36E-02	9.64E-01	anhydro-N-acetylmuramic acid kinase [EC:2.7.1.-]
KO:K09002	0.00	0.00	0.00	1.22	0.00	0.00	0.00	1.00E+00	1.00E+00	1.00E+00	5.84E-02	1.00E+00	1.00E+00	1.00E+00	hypothetical protein
KO:K09003	3.04	0.00	0.00	0.00	0.00	0.00	0.00	5.59E-02	1.00E+00	1.00E+00	3.10E-01	1.00E+00	1.00E+00	1.00E+00	hypothetical protein
KO:K09004	2.16	4.36	0.05	0.24	0.01	0.00	0.60	2.10E-01	8.91E-02	7.23E-01	6.32E-01	7.54E-01	1.00E+00	4.99E-01	hypothetical protein
KO:K09005	0.12	2.25	1.88	0.50	2.16	0.07	0.52	8.81E-01	9.57E-02	1.31E-01	6.51E-01	1.02E-01	9.26E-01	6.36E-01	hypothetical protein
KO:K09007	0.05	21.47	12.40	0.54	0.23	0.00	4.26	9.49E-01	3.57E-03	2.87E-02	9.02E-01	9.31E-01	1.00E+00	3.04E-01	hypothetical protein
KO:K09008	0.43	12.01	0.00	0.19	0.00	0.00	0.18	6.68E-01	5.00E-03	1.00E+00	7.96E-01	1.00E+00	1.00E+00	8.01E-01	hypothetical protein
KO:K09009	4.06	0.01	0.00	0.02	0.00	0.00	0.00	3.04E-02	5.68E-01	1.00E+00	5.45E-01	1.00E+00	1.00E+00	1.00E+00	hypothetical protein
KO:K09011	2.13	1.17	0.00	0.41	0.00	0.00	0.00	1.45E-01	2.19E-01	1.00E+00	3.44E-01	1.00E+00	1.00E+00	1.00E+00	D-citramalate synthase [EC:2.3.1.182]
KO:K09012	0.00	0.00	0.00	0.00	16.88	0.00	0.00	1.00E+00	1.00E+00	1.00E+00	1.00E+00	0.00E+00	1.00E+00	1.00E+00	DeoR family transcriptional regulator, suf operon transcriptional
KO:K09013	4.20	8.29	8.29	4.34	7.83	3.29	1.16	7.06E-01	8.79E-02	8.79E-02	6.74E-01	1.10E-01	8.58E-01	9.80E-01	Fe-S cluster assembly ATP-binding protein
KO:K09014	5.62	9.73	12.26	7.00	10.93	4.39	3.80	8.03E-01	1.73E-01	6.17E-02	5.87E-01	1.00E-01	9.01E-01	9.29E-01	Fe-S cluster assembly protein SufB
KO:K09015	4.18	12.52	10.22	1.87	4.29	1.44	0.20	5.26E-01	1.44E-02	3.44E-02	9.09E-01	5.00E-01	9.38E-01	9.90E-01	Fe-S cluster assembly protein SufD
KO:K09017	0.35	9.42	0.00	0.29	0.00	0.00	0.35	6.96E-01	8.57E-03	1.00E+00	7.32E-01	1.00E+00	1.00E+00	6.95E-01	TetR/AcrR family transcriptional regulator
KO:K09018	2.06	0.00	0.00	0.04	0.00	0.00	0.00	4.27E-02	1.00E+00	1.00E+00	2.90E-01	1.00E+00	1.00E+00	1.00E+00	putative monooxygenase RutA [EC:1.14.-.-]
KO:K09019	0.58	0.04	0.00	1.73	0.00	0.00	0.00	2.11E-01	5.46E-01	1.00E+00	7.83E-02	1.00E+00	1.00E+00	1.00E+00	putative NADH dehydrogenase/NAD(P)H nitroreductase RutE [EC:1.-.-.-]
KO:K09020	0.04	1.47	0.00	0.01	0.00	0.00	0.05	4.36E-01	5.23E-02	1.00E+00	5.03E-01	1.00E+00	1.00E+00	4.09E-01	putative isochorismatase family protein RutB [EC:3.-.-.-]
KO:K09023	0.13	0.34	0.00	0.00	0.00	0.00	0.00	1.95E-01	9.63E-02	1.00E+00	1.00E+00	1.00E+00	1.00E+00	1.00E+00	protein RutD
KO:K09024	0.00	0.73	0.00	0.11	0.00	0.00	0.00	1.00E+00	6.34E-02	1.00E+00	2.62E-01	1.00E+00	1.00E+00	1.00E+00	putative flavin reductase RutF [EC:1.5.1.-]
KO:K09065	2.41	0.01	0.00	0.19	0.00	0.00	0.00	5.73E-02	4.39E-01	1.00E+00	2.88E-01	1.00E+00	1.00E+00	1.00E+00	N-acetylornithine carbamoyltransferase [EC:2.1.3.9]
KO:K09117	8.31	9.03	1.48	5.81	0.00	5.47	0.95	9.07E-02	6.83E-02	9.24E-01	2.64E-01	1.00E+00	3.09E-01	9.56E-01	hypothetical protein
KO:K09118	0.00	0.00	0.00	0.00	20.80	0.00	1.08	6.01E-01	1.00E+00	1.00E+00	1.00E+00	2.86E-04	1.00E+00	1.53E-01	hypothetical protein
KO:K09120	0.00	0.00	0.00	0.00	0.00	0.00	1.25	1.00E+00	1.00E+00	1.00E+00	1.00E+00	1.00E+00	1.00E+00	2.40E-02	hypothetical protein
KO:K09121	0.00	0.00	0.26	0.00	23.24	0.00	0.38	7.28E-01	7.29E-01	4.08E-01	7.28E-01	2.86E-04	1.00E+00	3.38E-01	hypothetical protein
KO:K09123	0.78	0.56	0.00	0.74	0.00	0.00	1.11	2.49E-01	3.09E-01	1.00E+00	2.59E-01	1.00E+00	1.00E+00	1.87E-01	hypothetical protein
KO:K09124	0.35	0.00	0.00	0.00	0.00	0.00	0.00	6.37E-02	1.00E+00	1.00E+00	1.00E+00	1.00E+00	1.00E+00	1.00E+00	hypothetical protein
KO:K09125	3.25	10.99	0.01	4.38	4.32	0.59	0.43	4.99E-01	2.84E-02	9.81E-01	2.92E-01	3.01E-01	9.34E-01	9.45E-01	hypothetical protein
KO:K09127	0.35	0.00	0.00	0.00	0.00	0.00	0.00	5.03E-02	1.00E+00	1.00E+00	1.00E+00	1.00E+00	1.00E+00	1.00E+00	hypothetical protein
KO:K09128	0.00	0.01	0.00	1.02	0.00	0.00	0.47	1.00E+00	3.12E-01	1.00E+00	1.37E-01	1.00E+00	1.00E+00	1.52E-01	hypothetical protein
KO:K09131	0.04	0.06	0.69	0.02	0.08	0.00	0.31	5.37E-01	4.69E-01	5.53E-02	6.22E-01	4.19E-01	1.00E+00	1.59E-01	hypothetical protein
KO:K09134	0.87	0.15	0.09	2.03	0.04	9.86	0.00	3.91E-01	7.65E-01	8.13E-01	1.54E-01	8.52E-01	1.07E-02	1.00E+00	hypothetical protein
KO:K09136	0.39	0.20	0.00	0.17	0.00	0.00	0.00	1.38E-01	2.33E-01	1.00E+00	2.55E-01	1.00E+00	1.00E+00	1.00E+00	hypothetical protein
KO:K09138	0.00	0.00	0.00	0.20	0.00	0.00	0.00	1.00E+00	1.00E+00	1.00E+00	1.43E-01	1.00E+00	1.00E+00	1.00E+00	hypothetical protein
KO:K09140	0.00	0.00	0.14	0.02	0.14	0.00	0.58	1.00E+00	1.00E+00	1.71E-01	3.74E-01	1.71E-01	1.00E+00	4.16E-02	hypothetical protein
KO:K09141	0.12	0.00	0.00	0.00	0.00	0.00	0.00	6.01E-02	1.00E+00	1.00E+00	1.00E+00	1.00E+00	1.00E+00	1.00E+00	hypothetical protein
KO:K09142	0.00	0.00	0.00	0.00	0.00	0.00	2.08	1.00E+00	1.00E+00	1.00E+00	1.00E+00	1.00E+00	1.00E+00	1.57E-02	hypothetical protein
KO:K09143	0.00	0.00	0.00	0.66	0.00	0.00	0.90	1.00E+00	1.00E+00	1.00E+00	1.43E-01	1.00E+00	1.00E+00	1.27E-01	hypothetical protein
KO:K09144	0.00	0.01	1.42	0.00	0.00	0.00	0.63	1.00E+00	3.71E-01	2.26E-02	1.00E+00	1.00E+00	1.00E+00	7.06E-02	hypothetical protein
KO:K09148	0.00	0.00	0.00	0.00	0.00	0.00	1.25	1.00E+00	1.00E+00	1.00E+00	1.00E+00	1.00E+00	1.00E+00	2.73E-02	hypothetical protein
KO:K09149	0.00	0.00	0.00	0.00	0.00	0.00	1.67	1.00E+00	1.00E+00	1.00E+00	1.00E+00	1.00E+00	1.00E+00	2.30E-02	hypothetical protein
KO:K09151	4.33	0.56	0.87	1.67	0.00	0.10	0.00	5.53E-02	5.95E-01	4.67E-01	2.41E-01	1.00E+00	8.04E-01	1.00E+00	hypothetical protein
KO:K09152	0.00	0.00	0.00	0.00	0.00	0.00	2.50	1.00E+00	1.00E+00	1.00E+00	1.00E+00	1.00E+00	1.00E+00	2.91E-02	hypothetical protein
KO:K09153	0.91	0.00	0.00	0.01	0.00	0.00	1.30	1.18E-01	1.00E+00	1.00E+00	4.16E-01	1.00E+00	1.00E+00	9.04E-02	hypothetical protein
KO:K09154	0.01	0.00	0.31	0.21	0.00	0.00	0.00	3.37E-01	1.00E+00	9.51E-02	1.50E-01	1.00E+00	1.00E+00	1.00E+00	hypothetical protein
KO:K09158	8.79	0.38	0.00	0.08	0.00	0.92	0.16	9.43E-03	6.65E-01	1.00E+00	8.38E-01	1.00E+00	3.91E-01	7.87E-01	hypothetical protein
KO:K09159	3.06	1.24	0.23	0.62	0.00	0.00	1.09	7.50E-02	2.80E-01	7.70E-01	5.33E-01	1.00E+00	1.00E+00	3.27E-01	hypothetical protein
KO:K09160	3.45	2.63	2.03	0.35	0.00	0.26	0.33	8.80E-02	1.42E-01	2.13E-01	7.93E-01	1.00E+00	8.28E-01	8.02E-01	hypothetical protein
KO:K09162	0.00	0.00	2.92	0.02	3.39	0.00	1.11	6.00E-01	1.00E+00	3.24E-02	5.62E-01	2.53E-02	1.00E+00	1.31E-01	hypothetical protein
KO:K09163	0.00	0.00	0.00	1.02	0.00	0.00	0.00	1.00E+00	1.00E+00	1.00E+00	1.43E-01	1.00E+00	1.00E+00	1.00E+00	hypothetical protein
KO:K09164	1.12	0.12	0.53	0.17	0.00	0.00	0.00	9.10E-02	4.79E-01	1.88E-01	4.19E-01	1.00E+00	1.00E+00	1.00E+00	hypothetical protein
KO:K09165	0.00	0.05	0.00	0.54	0.00	0.00	0.00	1.00E+00	2.21E-01	1.00E+00	7.71E-02	1.00E+00	1.00E+00	1.00E+00	hypothetical protein
KO:K09166	0.01	0.00	9.79	0.01	0.00	0.00	0.01	5.06E-01	1.00E+00	2.71E-03	5.16E-01	1.00E+00	1.00E+00	4.70E-01	hypothetical protein
KO:K09167	0.34	0.00	0.00	0.36	0.00	0.00	0.00	1.42E-01	1.00E+00	1.00E+00	1.37E-01	1.00E+00	1.00E+00	1.00E+00	hypothetical protein
KO:K09181	0.02	0.02	0.35	0.05	0.00	0.00	4.37	6.26E-01	6.34E-01	2.14E-01	5.22E-01	1.00E+00	1.00E+00	8.57E-03	hypothetical protein
KO:K09190	0.00	0.11	0.00	0.00	0.00	0.00	0.00	1.00E+00	9.96E-02	1.00E+00	1.00E+00	1.00E+00	1.00E+00	1.00E+00	hypothetical protein
KO:K09252	0.45	0.28	0.00	0.78	0.00	0.00	0.00	2.28E-01	3.11E-01	1.00E+00	1.40E-01	1.00E+00	1.00E+00	1.00E+00	feruloyl esterase [EC:3.1.1.73]
KO:K09384	0.28	0.04	0.00	0.01	0.00	0.89	0.00	1.52E-01	4.20E-01	1.00E+00	4.79E-01	1.00E+00	4.41E-02	1.00E+00	hypothetical protein
KO:K09386	0.25	15.00	0.00	3.48	0.00	0.00	0.00	8.31E-01	5.86E-03	1.00E+00	1.70E-01	1.00E+00	1.00E+00	1.00E+00	hypothetical protein
KO:K09456	0.03	0.69	0.72	0.06	0.00	0.32	0.08	5.91E-01	1.44E-01	1.38E-01	5.03E-01	1.00E+00	2.64E-01	4.54E-01	putative acyl-CoA dehydrogenase
KO:K09457	1.48	0.06	0.26	1.17	4.17	0.30	0.23	2.09E-01	8.63E-01	7.43E-01	2.80E-01	4.13E-02	7.19E-01	7.63E-01	7-cyano-7-deazaguanine reductase [EC:1.7.1.13]
KO:K09458	7.47	15.30	25.66	8.49	10.41	4.24	3.07	8.80E-01	5.19E-02	5.71E-04	7.89E-01	4.87E-01	9.83E-01	9.93E-01	3-oxoacyl-[acyl-carrier-protein] synthase II [EC:2.3.1.179]
KO:K09459	0.73	1.10	0.00	0.00	0.00	0.00	0.00	1.67E-01	1.23E-01	1.00E+00	1.00E+00	1.00E+00	1.00E+00	1.00E+00	phosphonopyruvate decarboxylase [EC:4.1.1.82]
KO:K09461	0.14	0.42	0.00	0.28	0.00	0.00	0.00	2.88E-01	1.67E-01	1.00E+00	2.12E-01	1.00E+00	1.00E+00	1.00E+00	anthraniloyl-CoA monooxygenase [EC:1.14.13.4]
KO:K09471	4.33	11.44	1.53	0.51	0.00	0.00	0.15	1.20E-01	1.31E-02	5.58E-01	8.38E-01	1.00E+00	1.00E+00	9.57E-01	gamma-glutamylputrescine oxidase [EC:1.4.3.-]
KO:K09472	0.76	5.26	0.00	0.11	0.00	0.00	0.00	2.91E-01	3.47E-02	1.00E+00	6.07E-01	1.00E+00	1.00E+00	1.00E+00	gamma-glutamyl-gamma-aminobutyraldehyde dehydrogenase [EC:1.2.1.-]
KO:K09474	1.66	0.00	0.00	0.00	1.53	0.00	0.00	8.64E-02	1.00E+00	1.00E+00	1.00E+00	8.86E-02	1.00E+00	1.00E+00	acid phosphatase (class A) [EC:3.1.3.2]
KO:K09482	0.00	0.00	0.00	0.00	0.00	0.00	2.92	1.00E+00	1.00E+00	1.00E+00	1.00E+00	1.00E+00	1.00E+00	1.50E-02	glutamyl-tRNA(Gln) amidotransferase subunit D [EC:6.3.5.7]
KO:K09493	0.00	0.00	0.00	0.00	0.00	0.00	0.42	1.00E+00	1.00E+00	1.00E+00	1.00E+00	1.00E+00	1.00E+00	2.40E-02	T-complex protein 1 subunit alpha
KO:K09516	0.10	0.00	0.00	0.14	0.00	0.00	0.15	2.19E-01	1.00E+00	1.00E+00	1.76E-01	1.00E+00	1.00E+00	1.64E-01	all-trans-retinol 13,14-reductase [EC:1.3.99.23]
KO:K09530	0.00	0.00	0.00	0.00	2.50	0.00	0.00	1.00E+00	1.00E+00	1.00E+00	1.00E+00	1.59E-02	1.00E+00	1.00E+00	DnaJ homolog subfamily C member 1
KO:K09607	0.70	0.00	0.00	0.00	0.00	0.00	0.00	6.53E-02	1.00E+00	1.00E+00	1.00E+00	1.00E+00	1.00E+00	1.00E+00	immune inhibitor A [EC:3.4.24.-]
KO:K09611	0.00	0.00	0.00	0.10	0.00	0.00	0.00	1.00E+00	1.00E+00	1.00E+00	1.43E-01	1.00E+00	1.00E+00	1.00E+00	probable aminopeptidase NPEPL1 [EC:3.4.11.-]
KO:K09650	0.23	0.00	0.00	0.00	0.00	0.00	0.00	6.16E-02	1.00E+00	1.00E+00	1.00E+00	1.00E+00	1.00E+00	1.00E+00	rhomboid-like protein [EC:3.4.21.15]
KO:K09667	2.21	0.00	0.00	0.00	0.00	0.00	0.00	5.91E-02	1.00E+00	1.00E+00	1.00E+00	1.00E+00	1.00E+00	1.00E+00	polypeptide N-acetylglucosaminyltransferase [EC:2.4.1.-]
KO:K09686	6.96	5.16	0.23	4.45	6.63	0.80	1.59	1.21E-01	2.54E-01	9.74E-01	3.52E-01	1.39E-01	9.38E-01	8.62E-01	antibiotic transport system permease protein
KO:K09687	8.12	3.53	6.89	5.90	11.51	0.38	4.52	1.56E-01	8.28E-01	2.74E-01	4.28E-01	4.61E-02	9.91E-01	6.88E-01	antibiotic transport system ATP-binding protein
KO:K09688	0.00	0.04	0.05	0.01	3.23	0.00	0.20	6.20E-01	4.49E-01	4.16E-01	5.79E-01	1.73E-02	1.00E+00	2.22E-01	capsular polysaccharide transport system permease protein
KO:K09689	0.00	0.72	0.00	0.02	2.54	0.00	0.28	7.82E-01	1.09E-01	1.00E+00	7.53E-01	2.40E-02	1.00E+00	3.00E-01	capsular polysaccharide transport system ATP-binding protein
KO:K09690	0.94	0.03	7.01	0.28	1.40	0.00	0.57	3.12E-01	8.33E-01	1.71E-02	6.33E-01	2.00E-01	1.00E+00	4.64E-01	lipopolysaccharide transport system permease protein
KO:K09691	2.83	0.01	1.22	1.23	0.28	0.00	1.35	8.34E-02	9.18E-01	2.71E-01	2.68E-01	7.31E-01	1.00E+00	2.40E-01	lipopolysaccharide transport system ATP-binding protein
KO:K09692	2.60	0.00	0.00	0.01	0.00	0.00	0.00	4.36E-02	1.00E+00	1.00E+00	3.03E-01	1.00E+00	1.00E+00	1.00E+00	teichoic acid transport system permease protein
KO:K09694	0.09	0.00	0.00	0.00	0.00	0.71	0.71	2.64E-01	1.00E+00	1.00E+00	1.00E+00	1.00E+00	7.01E-02	7.01E-02	lipooligosaccharide transport system permease protein
KO:K09695	0.00	0.00	0.00	0.99	0.00	0.60	0.15	1.00E+00	1.00E+00	1.00E+00	9.87E-02	1.00E+00	1.39E-01	2.68E-01	lipooligosaccharide transport system ATP-binding protein
KO:K09696	0.32	0.00	7.21	0.03	0.69	0.00	0.00	3.28E-01	6.91E-01	6.00E-03	6.22E-01	2.00E-01	1.00E+00	1.00E+00	sodium transport system permease protein
KO:K09697	0.16	0.00	5.68	0.03	1.02	0.00	0.08	4.61E-01	1.00E+00	7.29E-03	6.69E-01	1.04E-01	1.00E+00	5.66E-01	sodium transport system ATP-binding protein
KO:K09699	0.51	0.41	0.23	0.39	0.00	0.00	0.03	2.24E-01	2.75E-01	3.94E-01	2.83E-01	1.00E+00	1.00E+00	6.80E-01	2-oxoisovalerate dehydrogenase E2 component (dihydrolipoyl
KO:K09700	0.70	0.00	0.00	0.17	0.00	0.00	0.00	8.84E-02	1.00E+00	1.00E+00	2.22E-01	1.00E+00	1.00E+00	1.00E+00	hypothetical protein
KO:K09701	4.49	8.81	2.26	2.12	0.00	0.00	0.45	1.30E-01	2.26E-02	5.05E-01	5.51E-01	1.00E+00	1.00E+00	9.29E-01	hypothetical protein

KO:K09702	0.70	0.00	0.00	0.00	0.00	0.00	0.00	6.24E-02	1.00E+00	1.00E+00	1.00E+00	1.00E+00	1.00E+00	1.00E+00	hypothetical protein
KO:K09703	3.71	0.00	0.00	0.49	0.00	0.00	0.00	5.96E-02	5.16E-01	1.00E+00	2.54E-01	1.00E+00	1.00E+00	1.00E+00	hypothetical protein
KO:K09704	0.04	0.00	0.00	0.13	0.00	0.00	0.00	1.77E-01	1.00E+00	1.00E+00	1.44E-01	1.00E+00	1.00E+00	1.00E+00	hypothetical protein
KO:K09705	0.04	0.09	0.00	0.47	2.80	0.00	0.02	7.65E-01	6.34E-01	1.00E+00	1.52E-01	3.14E-03	1.00E+00	8.03E-01	hypothetical protein
KO:K09707	0.00	0.00	0.00	0.20	0.00	0.00	0.00	1.00E+00	1.00E+00	1.00E+00	1.43E-01	1.00E+00	1.00E+00	1.00E+00	hypothetical protein
KO:K09709	0.40	10.55	0.00	0.13	0.00	0.00	0.00	5.12E-01	2.01E-02	1.00E+00	6.59E-01	1.00E+00	1.00E+00	1.00E+00	hypothetical protein
KO:K09710	4.49	9.91	3.29	4.04	5.62	2.21	0.25	4.51E-01	3.79E-02	7.15E-01	5.55E-01	2.50E-01	8.73E-01	9.89E-01	hypothetical protein
KO:K09711	0.01	0.65	0.00	0.00	0.00	0.00	0.11	2.60E-01	1.23E-01	1.00E+00	1.00E+00	1.00E+00	1.00E+00	1.78E-01	hypothetical protein
KO:K09716	0.00	0.00	0.00	0.00	0.00	0.00	2.08	1.00E+00	1.00E+00	1.00E+00	1.00E+00	1.00E+00	1.00E+00	1.59E-02	hypothetical protein
KO:K09721	0.00	0.00	0.00	0.00	0.00	0.00	2.50	1.00E+00	1.00E+00	1.00E+00	1.00E+00	1.00E+00	1.00E+00	1.40E-02	hypothetical protein
KO:K09722	0.00	0.00	0.00	0.00	0.00	0.00	0.83	1.00E+00	1.00E+00	1.00E+00	1.00E+00	1.00E+00	1.00E+00	2.94E-02	hypothetical protein
KO:K09723	0.00	0.00	0.00	0.00	0.00	0.00	2.08	1.00E+00	1.00E+00	1.00E+00	1.00E+00	1.00E+00	1.00E+00	1.57E-02	hypothetical protein
KO:K09726	0.00	0.00	0.00	0.00	0.00	0.00	1.25	1.00E+00	1.00E+00	1.00E+00	1.00E+00	1.00E+00	1.00E+00	6.29E-03	hypothetical protein
KO:K09732	0.00	0.00	0.00	0.10	0.00	0.00	0.00	1.00E+00	1.00E+00	1.00E+00	1.43E-01	1.00E+00	1.00E+00	1.00E+00	hypothetical protein
KO:K09733	0.00	0.00	0.63	0.00	0.00	0.00	0.00	1.00E+00	1.00E+00	1.77E-02	1.00E+00	1.00E+00	1.00E+00	1.00E+00	hypothetical protein
KO:K09735	0.00	0.00	0.00	0.00	0.00	0.00	0.83	1.00E+00	1.00E+00	1.00E+00	1.00E+00	1.00E+00	1.00E+00	2.63E-02	hypothetical protein
KO:K09736	0.00	0.00	0.00	0.00	0.00	0.00	0.42	1.00E+00	1.00E+00	1.00E+00	1.00E+00	1.00E+00	1.00E+00	2.84E-02	hypothetical protein
KO:K09740	0.27	1.61	0.00	0.44	0.00	0.00	0.00	3.78E-01	7.34E-02	1.00E+00	2.66E-01	1.00E+00	1.00E+00	1.00E+00	hypothetical protein
KO:K09741	0.00	0.00	0.00	0.00	0.00	0.00	2.08	1.00E+00	1.00E+00	1.00E+00	1.00E+00	1.00E+00	1.00E+00	6.29E-03	hypothetical protein
KO:K09744	0.00	0.00	0.00	0.00	0.00	0.00	2.82	1.00E+00	1.00E+00	1.00E+00	2.39E-01	1.00E+00	1.00E+00	2.24E-02	hypothetical protein
KO:K09747	2.75	2.70	2.34	1.45	3.18	1.41	0.65	2.86E-01	3.01E-01	3.89E-01	6.72E-01	2.11E-01	6.84E-01	8.81E-01	hypothetical protein
KO:K09748	9.56	6.76	0.65	4.29	3.31	9.27	1.83	7.79E-02	2.29E-01	9.78E-01	6.31E-01	7.94E-01	8.57E-02	9.27E-01	hypothetical protein
KO:K09749	0.10	0.00	0.74	0.08	0.00	0.00	0.00	2.51E-01	1.00E+00	4.01E-02	2.81E-01	1.00E+00	1.00E+00	1.00E+00	hypothetical protein
KO:K09758	0.00	0.00	0.00	0.20	0.00	0.00	0.00	1.00E+00	1.00E+00	1.00E+00	8.17E-02	1.00E+00	1.00E+00	1.00E+00	aspartate 4-decarboxylase [EC:4.1.1.12]
KO:K09760	4.10	2.47	2.83	4.62	0.52	1.26	1.26	2.00E-01	5.25E-01	4.23E-01	1.49E-01	9.36E-01	8.28E-01	8.28E-01	DNA recombination protein RmuC
KO:K09761	3.16	2.78	5.50	1.94	7.99	0.20	0.32	3.09E-01	3.98E-01	6.69E-02	6.79E-01	2.16E-02	9.85E-01	9.74E-01	ribosomal RNA small subunit methyltransferase E [EC:2.1.1.-]
KO:K09763	0.00	0.00	1.88	0.00	12.15	0.00	0.03	1.00E+00	1.00E+00	5.80E-02	1.00E+00	7.14E-04	1.00E+00	5.63E-01	hypothetical protein
KO:K09765	0.01	0.00	0.00	0.06	0.00	0.00	8.54	4.27E-01	1.00E+00	1.00E+00	3.52E-01	1.00E+00	1.00E+00	1.69E-02	hypothetical protein
KO:K09767	1.50	0.06	0.01	3.93	13.77	0.00	0.03	3.75E-01	8.77E-01	9.09E-01	9.40E-02	5.86E-03	1.00E+00	8.96E-01	hypothetical protein
KO:K09768	1.81	8.47	0.55	0.06	0.00	0.00	0.41	2.19E-01	9.14E-03	7.37E-01	9.31E-01	1.00E+00	1.00E+00	8.04E-01	hypothetical protein
KO:K09769	0.44	7.48	4.92	2.74	1.07	0.00	0.02	8.68E-01	4.23E-02	9.49E-02	2.76E-01	7.04E-01	1.00E+00	9.56E-01	hypothetical protein
KO:K09771	0.00	0.11	0.00	0.00	0.00	0.00	0.00	1.00E+00	1.02E-01	1.00E+00	1.00E+00	1.00E+00	1.00E+00	1.00E+00	hypothetical protein
KO:K09772	0.00	0.00	0.00	0.00	26.88	0.00	0.00	1.00E+00	1.00E+00	1.00E+00	1.00E+00	0.00E+00	1.00E+00	1.00E+00	cell division inhibitor SepF
KO:K09773	0.72	0.04	0.16	5.89	0.00	0.00	0.65	4.07E-01	8.13E-01	6.83E-01	3.59E-02	1.00E+00	1.00E+00	4.29E-01	hypothetical protein
KO:K09774	1.48	4.89	0.00	0.99	4.49	0.00	0.06	3.93E-01	4.34E-02	1.00E+00	5.91E-01	5.20E-02	1.00E+00	9.34E-01	lipopolysaccharide export system protein LptA
KO:K09775	0.00	0.00	0.00	0.00	18.13	0.00	0.00	1.00E+00	1.00E+00	1.00E+00	1.00E+00	0.00E+00	1.00E+00	1.00E+00	hypothetical protein
KO:K09776	0.00	0.00	0.00	2.96	0.00	0.00	0.00	1.00E+00	1.00E+00	1.00E+00	8.27E-02	1.00E+00	1.00E+00	1.00E+00	hypothetical protein
KO:K09778	0.25	0.00	6.45	0.14	1.14	0.00	0.03	3.75E-01	7.43E-01	2.21E-02	4.72E-01	1.48E-01	1.00E+00	6.39E-01	hypothetical protein
KO:K09780	0.71	1.48	0.23	0.89	6.77	0.07	0.02	5.46E-01	2.56E-01	8.07E-01	4.59E-01	1.59E-02	9.07E-01	9.39E-01	hypothetical protein
KO:K09781	1.36	0.73	0.00	0.17	0.00	0.31	0.00	6.83E-02	1.61E-01	1.00E+00	5.73E-01	1.00E+00	3.83E-01	1.00E+00	hypothetical protein
KO:K09786	0.13	0.00	1.08	0.06	1.08	0.63	0.48	5.23E-01	7.63E-01	8.04E-02	6.41E-01	8.04E-02	1.69E-01	2.21E-01	hypothetical protein
KO:K09788	2.12	3.81	0.77	1.95	0.00	5.50	0.01	3.54E-01	1.72E-01	6.59E-01	3.81E-01	1.00E+00	9.49E-02	8.97E-01	hypothetical protein
KO:K09790	0.18	1.75	0.03	0.80	0.00	0.22	0.06	6.46E-01	6.87E-02	8.21E-01	2.04E-01	1.00E+00	5.97E-01	8.00E-01	hypothetical protein
KO:K09791	6.97	2.21	0.04	1.15	0.00	0.00	0.24	3.07E-02	2.17E-01	9.23E-01	4.74E-01	1.00E+00	1.00E+00	8.28E-01	hypothetical protein
KO:K09792	0.76	0.01	1.27	0.03	0.00	0.00	2.60	1.78E-01	8.01E-01	9.21E-02	7.32E-01	1.00E+00	1.00E+00	2.87E-02	hypothetical protein
KO:K09793	0.05	1.04	0.29	0.54	0.00	0.92	0.00	5.78E-01	1.71E-01	3.72E-01	2.77E-01	1.00E+00	1.90E-01	1.00E+00	hypothetical protein
KO:K09794	1.25	0.01	0.00	0.02	0.00	0.00	0.00	3.71E-02	5.01E-01	1.00E+00	4.41E-01	1.00E+00	1.00E+00	1.00E+00	hypothetical protein
KO:K09796	1.96	5.87	0.11	1.45	0.00	1.25	0.70	3.32E-01	4.03E-02	9.32E-01	4.81E-01	1.00E+00	5.54E-01	7.53E-01	hypothetical protein
KO:K09797	1.08	0.11	0.00	0.14	0.22	0.00	0.22	1.35E-01	4.36E-01	1.00E+00	4.00E-01	3.42E-01	1.00E+00	3.42E-01	hypothetical protein
KO:K09798	0.33	0.02	0.03	0.59	0.00	3.34	0.01	3.24E-01	7.53E-01	7.39E-01	1.95E-01	1.00E+00	1.89E-02	7.72E-01	hypothetical protein
KO:K09799	0.00	7.00	0.00	0.00	0.00	0.00	0.00	1.00E+00	2.10E-02	1.00E+00	1.00E+00	1.00E+00	1.00E+00	1.00E+00	hypothetical protein
KO:K09800	0.01	0.15	0.42	0.04	12.18	0.00	0.00	8.32E-01	5.36E-01	2.94E-01	7.14E-01	7.14E-04	1.00E+00	1.00E+00	hypothetical protein
KO:K09801	2.22	0.00	0.41	0.22	0.00	5.20	0.86	9.03E-02	8.59E-01	5.23E-01	6.51E-01	1.00E+00	2.34E-02	3.09E-01	hypothetical protein
KO:K09803	0.24	0.01	0.05	0.23	0.00	0.00	2.54	2.95E-01	5.24E-01	4.22E-01	2.98E-01	1.00E+00	1.00E+00	5.83E-02	hypothetical protein
KO:K09806	1.17	7.93	0.00	0.71	0.00	0.00	0.00	3.42E-01	1.74E-02	1.00E+00	4.98E-01	1.00E+00	1.00E+00	1.00E+00	hypothetical protein
KO:K09807	1.37	0.75	0.00	0.59	0.00	0.25	0.00	9.11E-02	2.04E-01	1.00E+00	2.77E-01	1.00E+00	5.31E-01	1.00E+00	hypothetical protein
KO:K09808	7.63	7.05	7.68	3.59	0.73	4.68	2.81	1.09E-01	1.46E-01	1.06E-01	7.63E-01	9.88E-01	5.16E-01	8.70E-01	lipoprotein-releasing system permease protein
KO:K09810	4.44	1.60	7.48	3.12	2.22	0.33	0.93	1.62E-01	7.65E-01	4.31E-02	3.64E-01	5.98E-01	9.65E-01	8.87E-01	lipoprotein-releasing system ATP-binding protein [EC:3.6.3.-]
KO:K09811	5.35	6.59	0.05	0.81	0.00	4.77	0.04	4.56E-02	2.53E-02	9.73E-01	8.56E-01	1.00E+00	6.29E-02	9.75E-01	cell division transport system permease protein
KO:K09812	7.47	7.11	0.36	1.87	0.00	3.19	0.03	4.39E-02	4.79E-02	9.47E-01	6.65E-01	1.00E+00	3.18E-01	9.83E-01	cell division transport system ATP-binding protein
KO:K09815	0.87	5.63	0.00	2.57	0.00	0.08	0.41	5.86E-01	3.01E-02	1.00E+00	1.40E-01	1.00E+00	9.17E-01	7.94E-01	zinc transport system substrate-binding protein
KO:K09816	1.73	6.52	0.01	1.53	0.00	0.96	5.33	5.32E-01	4.23E-02	9.75E-01	6.03E-01	1.00E+00	7.85E-01	6.71E-02	zinc transport system permease protein
KO:K09817	1.37	6.61	0.01	2.59	0.01	0.00	2.19	5.39E-01	3.19E-02	9.69E-01	2.13E-01	9.69E-01	1.00E+00	2.93E-01	zinc transport system ATP-binding protein [EC:3.6.3.-]
KO:K09818	0.23	1.72	0.00	0.40	8.55	0.00	0.00	6.61E-01	1.62E-01	1.00E+00	5.56E-01	1.09E-02	1.00E+00	1.00E+00	manganese/iron transport system substrate-binding protein
KO:K09819	0.05	0.00	0.00	0.07	20.19	0.09	0.05	5.99E-01	6.89E-01	1.00E+00	5.87E-01	1.43E-04	5.63E-01	6.01E-01	manganese/iron transport system permease protein
KO:K09820	0.00	0.00	0.00	0.00	17.38	0.00	0.00	5.09E-01	1.00E+00	1.00E+00	1.00E+00	1.43E-04	1.00E+00	1.00E+00	manganese/iron transport system ATP-binding protein
KO:K09822	0.14	0.00	0.11	0.03	2.62	0.00	0.78	4.30E-01	7.46E-01	4.74E-01	6.87E-01	2.37E-02	1.00E+00	9.84E-02	hypothetical protein
KO:K09823	1.04	6.87	0.00	1.40	0.00	1.22	0.00	4.99E-01	2.20E-02	1.00E+00	3.58E-01	1.00E+00	4.22E-01	9.60E-01	Fur family transcriptional regulator, zinc uptake regulator
KO:K09825	0.00	0.00	15.89	0.00	2.19	0.00	0.00	4.56E-01	1.00E+00	7.29E-03	1.00E+00	9.74E-02	1.00E+00	1.00E+00	Fur family transcriptional regulator, peroxide stress response
KO:K09826	1.61	7.58	0.00	4.25	0.00	1.81	0.00	5.11E-01	5.07E-02	1.00E+00	1.49E-01	1.00E+00	4.58E-01	1.00E+00	Fur family transcriptional regulator, iron response regulator
KO:K09835	0.00	0.00	0.00	0.00	19.38	0.00	0.00	1.00E+00	1.00E+00	1.00E+00	1.00E+00	1.43E-04	1.00E+00	1.00E+00	carotenoid isomerase [EC:5.-.-.-]
KO:K09836	0.00	0.00	0.00	0.07	8.62	0.00	0.00	1.00E+00	1.00E+00	1.00E+00	3.41E-01	2.14E-03	1.00E+00	1.00E+00	beta-carotene ketolase (CrtW type)
KO:K09841	2.33	0.00	0.00	0.00	0.00	0.00	0.00	5.90E-02	1.00E+00	1.00E+00	1.00E+00	1.00E+00	1.00E+00	1.00E+00	xanthoxin dehydrogenase [EC:1.1.1.288]
KO:K09844	1.17	6.06	0.00	0.43	0.00	0.00	0.00	2.87E-01	3.04E-02	1.00E+00	5.61E-01	1.00E+00	1.00E+00	1.00E+00	hydroxyneurosporene dehydrogenase
KO:K09845	1.10	8.02	0.00	0.45	0.00	0.00	0.00	3.50E-01	1.40E-02	1.00E+00	6.09E-01	1.00E+00	1.00E+00	1.00E+00	methoxyneurosporene dehydrogenase [EC:1.14.99.-]
KO:K09846	1.16	6.49	0.00	0.22	0.00	0.00	0.00	2.69E-01	1.94E-02	1.00E+00	6.84E-01	1.00E+00	1.00E+00	1.00E+00	hydroxyneurosporene methyltransferase [EC:2.1.1.-]
KO:K09847	0.03	3.72	0.00	0.17	0.00	4.60	0.00	6.80E-01	6.10E-02	1.00E+00	5.91E-01	1.00E+00	4.66E-02	1.00E+00	spheroidene monooxygenase [EC:1.-.-.-]
KO:K09857	0.70	0.00	0.00	0.00	0.00	0.00	0.00	3.53E-02	1.00E+00	1.00E+00	1.00E+00	1.00E+00	1.00E+00	1.00E+00	hypothetical protein
KO:K09858	0.26	0.00	0.72	0.22	2.89	0.00	0.01	3.77E-01	8.01E-01	1.38E-01	4.16E-01	1.49E-02	1.00E+00	7.90E-01	SEC-C motif domain protein
KO:K09859	0.11	0.00	0.00	0.06	0.00	0.00	6.74	3.49E-01	5.67E-01	1.00E+00	3.97E-01	1.00E+00	1.00E+00	1.57E-02	hypothetical protein
KO:K09860	7.30	1.88	0.00	0.00	0.00	0.00	0.01	4.14E-02	2.24E-01	1.00E+00	6.54E-01	1.00E+00	1.00E+00	6.39E-01	hypothetical protein
KO:K09861	7.62	15.32	0.68	2.16	0.00	0.62	0.75	7.89E-02	7.57E-03	9.39E-01	7.73E-01	1.00E+00	9.45E-01	9.34E-01	hypothetical protein
KO:K09862	0.14	0.19	0.38	0.25	0.00	0.67	1.77	6.28E-01	5.53E-01	3.49E-01	4.71E-01	1.00E+00	1.96E-01	5.07E-02	hypothetical protein
KO:K09865	0.00	0.00	0.00	0.00	0.00	0.00	1.67	1.00E+00	1.00E+00	1.00E+00	1.00E+00	1.00E+00	1.00E+00	2.36E-02	aquaporin-2
KO:K09880	0.00	0.00	2.00	0.00	6.31	0.00	0.00	5.79E-01	1.00E+00	4.09E-02	1.00E+00	5.29E-03	1.00E+00	1.00E+00	enolase-phosphatase E1 [EC:3.1.3.77]
KO:K09882	1.28	20.86	0.00	7.38	0.00	0.00	0.00	7.90E-01	3.00E-03	1.00E+00	7.97E-02	1.00E+00	1.00E+00	1.00E+00	cobaltochelatase CobS [EC:6.6.1.2]
KO:K09883	1.19	13.97	0.00	5.87	0.00	0.00	0.11	7.27E-01	1.04E-02	1.00E+00	8.67E-02	1.00E+00	1.00E+00	9.29E-01	cobaltochelatase CobT [EC:6.6.1.2]
KO:K09888	4.46	4.27	0.08	2.80	0.00	7.66	0.02	1.29E-01	1.40E-01	9.62E-01	3.19E-01	1.00E+00	3.84E-02	9.77E-01	cell division protein ZapA
KO:K09889	2.61	0.21	0.00	1.12	0.00	0.00	0.06	6.31E-02	5.55E-01	1.00E+00	1.84E-01	1.00E+00	1.00E+00	7.20E-01	hypothetical protein
KO:K09890	0.00	0.00	0.00	0.51	0.00	0.00	0.00	1.00E+00	1.00E+00	1.00E+00	1.43E-01	1.00E+00	1.00E+00	1.00E+00	hypothetical protein
KO:K09891	0.07	0.39	0.00	0.00	0.00	0.00	0.00	2.22E-01	7.43E-02	1.00E+00	1.00E+00	1.00E+00	1.00E+00	1.00E+00	hypothetical protein
KO:K09892	0.00	1.89	0.00	0.00	0.00	0.00	0.00	1.00E+00	4.46E-02	1.00E+00	1.00E+00	1.00E+00	1.00E+00	1.00E+00	hypothetical protein
KO:K09893	0.12	0.00	0.00	0.00	0.00	0.00	0.00	5.90E-02	1.00E+00	1.00E+00	1.00E+00	1.00E+00	1.00E+00	1.00E+00	hypothetical protein
KO:K09895	2.95	0.08	0.01	0.03	0.00	12.95	0.02	8.33E-02	7.16E-01	8.25E-01	7.71E-01	1.00E+00	5.29E-03	7.98E-01	hypothetical protein
KO:K09898	1.74	0.01	0.07	0.00	0.00	0.00	0.49	5.23E-02	5.85E-01	4.46E-01	1.00E+00	1.00E+00	1.00E+00	1.83E-01	hypothetical protein
KO:K09899	0.06	0.00	0.41	0.01	0.00	0.00	0.00	1.89E-01	1.00E+00	5.31E-02	3.08E-01	1.00E+00	1.00E+00	1.00E+00	hypothetical protein

KO:K09902	5.58	0.17	0.04	0.05	0.00	0.00	0.00	1.29E-02	5.79E-01	7.52E-01	7.35E-01	1.00E+00	1.00E+00	1.00E+00	hypothetical protein
KO:K09903	11.18	9.41	6.70	4.03	7.66	2.07	4.75	7.56E-02	1.49E-01	4.98E-01	8.89E-01	3.31E-01	9.73E-01	8.29E-01	uridylate kinase [EC:2.7.4.22]
KO:K09906	5.83	0.05	0.04	0.02	0.00	0.00	0.00	1.69E-02	6.46E-01	6.57E-01	7.12E-01	1.00E+00	1.00E+00	1.00E+00	hypothetical protein
KO:K09908	6.37	0.07	1.25	0.03	0.00	0.14	0.00	2.93E-02	6.59E-01	2.29E-01	6.92E-01	1.00E+00	6.04E-01	1.00E+00	hypothetical protein
KO:K09909	0.00	0.22	0.00	0.00	0.00	0.00	0.00	1.00E+00	9.99E-02	1.00E+00	1.00E+00	1.00E+00	1.00E+00	1.00E+00	hypothetical protein
KO:K09912	0.50	0.00	0.57	0.03	0.00	0.00	0.00	9.89E-02	1.00E+00	8.74E-02	4.25E-01	1.00E+00	1.00E+00	1.00E+00	hypothetical protein
KO:K09913	1.41	0.02	1.00	0.00	0.00	0.00	0.64	8.71E-02	5.99E-01	1.25E-01	1.00E+00	1.00E+00	1.00E+00	1.89E-01	hypothetical protein
KO:K09914	0.00	0.56	0.00	0.00	0.00	0.00	0.00	1.00E+00	3.66E-02	1.00E+00	1.00E+00	1.00E+00	1.00E+00	1.00E+00	putative lipoprotein
KO:K09915	0.00	0.01	10.18	0.00	0.00	0.00	0.01	6.12E-01	5.87E-01	3.14E-03	6.20E-01	1.00E+00	1.00E+00	5.58E-01	hypothetical protein
KO:K09916	4.38	0.25	0.00	0.01	0.00	0.00	0.00	3.41E-02	3.63E-01	1.00E+00	6.34E-01	1.00E+00	1.00E+00	1.00E+00	hypothetical protein
KO:K09918	0.81	0.00	0.00	0.00	0.00	0.00	0.00	4.64E-02	1.00E+00	1.00E+00	1.00E+00	1.00E+00	1.00E+00	1.00E+00	hypothetical protein
KO:K09919	7.46	6.56	0.08	3.41	2.18	0.00	0.00	3.40E-02	5.09E-02	9.81E-01	2.72E-01	5.98E-01	1.00E+00	1.00E+00	hypothetical protein
KO:K09920	11.21	0.12	0.00	0.03	0.00	0.00	0.32	9.71E-03	6.98E-01	1.00E+00	7.70E-01	1.00E+00	1.00E+00	5.91E-01	hypothetical protein
KO:K09921	4.74	12.14	0.03	0.54	0.00	0.00	0.40	9.83E-02	8.29E-03	9.56E-01	8.42E-01	1.00E+00	1.00E+00	8.76E-01	hypothetical protein
KO:K09922	0.00	0.00	0.00	0.31	0.00	0.00	0.00	1.00E+00	1.00E+00	1.00E+00	7.21E-02	1.00E+00	1.00E+00	1.00E+00	hypothetical protein
KO:K09924	0.00	0.00	0.00	1.02	0.00	0.00	0.00	1.00E+00	1.00E+00	1.00E+00	1.43E-01	1.00E+00	1.00E+00	1.00E+00	hypothetical protein
KO:K09926	0.90	0.28	0.00	0.00	0.00	0.00	0.00	4.94E-02	1.77E-01	1.00E+00	1.00E+00	1.00E+00	1.00E+00	1.00E+00	hypothetical protein
KO:K09927	0.09	0.34	0.00	0.07	0.00	0.00	0.00	2.95E-01	9.89E-02	1.00E+00	3.39E-01	1.00E+00	1.00E+00	1.00E+00	hypothetical protein
KO:K09928	3.28	7.39	0.28	0.89	0.47	0.04	1.74	1.96E-01	3.29E-02	9.11E-01	7.85E-01	8.77E-01	9.56E-01	5.23E-01	hypothetical protein
KO:K09929	0.62	0.04	0.32	0.01	0.00	0.00	0.32	1.05E-01	5.31E-01	2.11E-01	6.21E-01	1.00E+00	1.00E+00	2.11E-01	hypothetical protein
KO:K09930	0.96	5.99	0.43	1.19	0.00	0.09	0.05	4.99E-01	2.90E-02	7.41E-01	4.08E-01	1.00E+00	8.72E-01	9.07E-01	hypothetical protein
KO:K09931	0.62	0.88	1.38	0.27	1.66	0.00	0.39	4.40E-01	2.84E-01	1.39E-01	7.30E-01	9.80E-02	1.00E+00	6.22E-01	hypothetical protein
KO:K09932	0.01	0.01	0.00	0.01	0.41	0.00	0.00	3.29E-01	3.51E-01	1.00E+00	3.65E-01	3.83E-02	1.00E+00	1.00E+00	hypothetical protein
KO:K09933	0.14	0.04	1.34	0.18	0.00	0.00	0.00	3.27E-01	5.51E-01	3.39E-02	2.87E-01	1.00E+00	1.00E+00	1.00E+00	hypothetical protein
KO:K09935	0.14	0.00	0.00	0.01	0.00	0.00	0.20	1.40E-01	1.00E+00	1.00E+00	3.54E-01	1.00E+00	1.00E+00	1.17E-01	hypothetical protein
KO:K09936	0.02	6.12	0.00	0.02	0.00	0.00	0.26	7.43E-01	1.50E-02	1.00E+00	7.37E-01	1.00E+00	1.00E+00	4.78E-01	hypothetical protein
KO:K09937	2.68	1.09	0.00	1.89	0.00	0.00	1.12	1.18E-01	3.67E-01	1.00E+00	1.99E-01	1.00E+00	1.00E+00	3.57E-01	hypothetical protein
KO:K09938	5.82	0.00	0.11	0.21	0.00	0.00	0.31	2.49E-02	1.00E+00	6.40E-01	5.45E-01	1.00E+00	1.00E+00	4.81E-01	hypothetical protein
KO:K09939	0.09	0.00	0.28	0.00	0.00	0.00	0.12	1.92E-01	1.00E+00	8.39E-02	1.00E+00	1.00E+00	1.00E+00	1.49E-01	hypothetical protein
KO:K09940	0.17	0.00	0.31	0.02	0.00	0.56	0.14	2.18E-01	1.00E+00	1.39E-01	5.44E-01	1.00E+00	8.16E-02	2.64E-01	hypothetical protein
KO:K09941	2.65	0.07	0.06	0.01	0.00	0.00	0.10	2.03E-02	6.18E-01	6.39E-01	7.44E-01	1.00E+00	1.00E+00	5.57E-01	hypothetical protein
KO:K09942	0.00	0.00	6.69	0.00	23.41	0.00	0.00	1.00E+00	1.00E+00	2.71E-02	1.00E+00	8.57E-04	1.00E+00	6.27E-01	hypothetical protein
KO:K09946	0.24	0.61	0.00	0.02	0.00	0.00	0.00	1.95E-01	6.53E-02	1.00E+00	5.63E-01	1.00E+00	1.00E+00	1.00E+00	hypothetical protein
KO:K09947	0.01	2.48	0.00	0.52	0.00	0.00	0.00	7.64E-01	2.97E-02	1.00E+00	2.12E-01	1.00E+00	1.00E+00	1.00E+00	hypothetical protein
KO:K09948	3.81	3.05	0.03	4.11	0.00	2.42	0.00	1.29E-01	1.98E-01	9.59E-01	1.13E-01	1.00E+00	2.96E-01	9.67E-01	hypothetical protein
KO:K09949	0.25	1.73	17.18	0.47	2.42	0.00	0.06	7.87E-01	3.07E-01	3.29E-03	6.99E-01	2.00E-01	1.00E+00	8.76E-01	hypothetical protein
KO:K09950	0.78	0.07	0.00	0.93	0.00	0.00	0.00	1.16E-01	5.64E-01	1.00E+00	9.17E-02	1.00E+00	1.00E+00	1.00E+00	hypothetical protein
KO:K09951	0.24	0.00	0.97	0.18	0.00	0.00	0.00	2.15E-01	1.00E+00	7.80E-02	2.36E-01	1.00E+00	1.00E+00	1.00E+00	CRISPR-associated protein Cas2
KO:K09952	0.37	0.07	2.29	0.01	0.00	0.00	0.00	2.04E-01	3.60E-01	3.74E-02	5.00E-01	1.00E+00	1.00E+00	1.00E+00	hypothetical protein
KO:K09953	0.11	0.14	0.00	0.48	0.13	0.00	0.00	3.69E-01	3.19E-01	1.00E+00	1.26E-01	3.37E-01	1.00E+00	1.00E+00	lipid A 3-O-deacylase
KO:K09954	0.20	0.00	0.12	0.62	0.00	0.00	0.05	2.06E-01	1.00E+00	2.58E-01	1.52E-01	1.00E+00	1.00E+00	3.02E-01	hypothetical protein
KO:K09955	0.03	0.00	1.43	0.01	0.00	0.00	1.25	4.08E-01	5.36E-01	4.60E-02	4.94E-01	1.00E+00	1.00E+00	5.20E-02	hypothetical protein
KO:K09956	0.00	0.00	0.63	0.00	0.00	0.00	0.00	1.00E+00	1.00E+00	1.61E-02	1.00E+00	1.00E+00	1.00E+00	1.00E+00	hypothetical protein
KO:K09958	0.00	0.00	0.00	0.00	0.63	0.00	0.00	1.00E+00	1.00E+00	1.00E+00	1.00E+00	1.69E-02	1.00E+00	1.00E+00	hypothetical protein
KO:K09959	0.00	0.08	0.00	6.25	0.00	0.00	0.00	6.33E-01	5.02E-01	1.00E+00	2.81E-02	1.00E+00	1.00E+00	1.00E+00	hypothetical protein
KO:K09964	0.29	0.04	0.52	0.09	0.00	0.00	0.00	1.56E-01	5.54E-01	7.24E-02	4.09E-01	1.00E+00	1.00E+00	1.00E+00	hypothetical protein
KO:K09966	2.87	1.20	1.36	1.53	8.69	0.48	0.60	2.56E-01	6.77E-01	6.27E-01	5.71E-01	2.51E-02	8.58E-01	8.28E-01	hypothetical protein
KO:K09967	0.13	20.79	0.00	0.41	0.00	0.00	0.00	8.47E-01	2.00E-03	1.00E+00	7.38E-01	1.00E+00	1.00E+00	1.00E+00	hypothetical protein
KO:K09968	0.00	0.21	0.00	0.27	0.00	0.00	0.00	1.00E+00	1.53E-01	1.00E+00	1.30E-01	1.00E+00	1.00E+00	1.00E+00	hypothetical protein
KO:K09969	2.02	20.74	0.00	12.99	3.83	0.00	0.00	8.36E-01	1.19E-02	1.00E+00	5.90E-02	5.99E-01	1.00E+00	1.00E+00	general L-amino acid transport system substrate-binding protein
KO:K09970	1.41	35.20	0.00	7.50	3.22	0.00	0.00	9.05E-01	0.00E+00	1.00E+00	1.84E-01	7.20E-01	1.00E+00	1.00E+00	general L-amino acid transport system permease protein
KO:K09971	0.96	30.00	0.00	7.65	2.57	0.00	0.00	9.02E-01	1.86E-03	1.00E+00	1.34E-01	7.42E-01	1.00E+00	1.00E+00	general L-amino acid transport system permease protein
KO:K09972	1.62	25.30	0.00	5.98	0.26	0.00	0.00	7.76E-01	1.71E-03	1.00E+00	1.63E-01	9.26E-01	1.00E+00	1.00E+00	general L-amino acid transport system ATP-binding protein
KO:K09973	0.25	0.00	1.78	0.64	0.00	0.00	0.02	3.25E-01	7.22E-01	5.17E-02	1.58E-01	1.00E+00	1.00E+00	6.57E-01	hypothetical protein
KO:K09974	0.10	0.13	1.00	0.05	0.00	0.00	0.00	3.31E-01	2.91E-01	4.83E-02	4.24E-01	1.00E+00	1.00E+00	1.00E+00	hypothetical protein
KO:K09975	0.58	0.00	0.00	0.00	0.00	0.00	0.00	6.34E-02	1.00E+00	1.00E+00	1.00E+00	1.00E+00	1.00E+00	1.00E+00	hypothetical protein
KO:K09977	0.08	0.03	3.12	0.06	0.00	0.00	0.00	4.55E-01	5.83E-01	4.71E-03	5.02E-01	1.00E+00	1.00E+00	1.00E+00	hypothetical protein
KO:K09978	0.28	0.00	0.00	0.12	0.00	0.00	0.00	9.09E-02	1.00E+00	1.00E+00	1.99E-01	1.00E+00	1.00E+00	1.00E+00	hypothetical protein
KO:K09979	0.35	0.00	0.00	0.00	0.00	0.00	0.00	6.16E-02	1.00E+00	1.00E+00	1.00E+00	1.00E+00	1.00E+00	1.00E+00	hypothetical protein
KO:K09980	0.38	0.00	0.00	0.02	0.00	0.00	0.00	4.44E-02	1.00E+00	1.00E+00	2.82E-01	1.00E+00	1.00E+00	1.00E+00	hypothetical protein
KO:K09981	0.00	0.06	0.00	0.05	0.00	0.00	0.00	1.00E+00	1.47E-01	1.00E+00	1.91E-01	1.00E+00	1.00E+00	1.00E+00	hypothetical protein
KO:K09983	1.22	0.00	0.00	2.31	0.00	0.00	0.00	1.57E-01	6.24E-01	1.00E+00	9.40E-02	1.00E+00	1.00E+00	1.00E+00	hypothetical protein
KO:K09984	0.06	0.20	0.00	0.10	0.00	0.00	0.00	2.83E-01	1.57E-01	1.00E+00	2.49E-01	1.00E+00	1.00E+00	1.00E+00	hypothetical protein
KO:K09985	0.58	11.41	0.00	1.12	0.00	0.00	0.00	6.38E-01	9.29E-03	1.00E+00	4.35E-01	1.00E+00	1.00E+00	1.00E+00	hypothetical protein
KO:K09986	6.92	6.95	0.00	0.47	0.00	0.00	0.21	3.41E-02	3.37E-02	1.00E+00	8.14E-01	1.00E+00	1.00E+00	8.92E-01	hypothetical protein
KO:K09987	0.45	20.98	0.00	5.47	0.00	0.00	0.00	8.71E-01	2.71E-03	1.00E+00	1.20E-01	1.00E+00	1.00E+00	1.00E+00	hypothetical protein
KO:K09988	0.02	7.60	0.00	0.04	0.00	0.00	0.00	6.03E-01	2.07E-02	1.00E+00	5.68E-01	1.00E+00	1.00E+00	1.00E+00	hypothetical protein
KO:K09989	8.44	11.33	0.55	2.78	0.00	0.00	0.25	5.19E-02	2.54E-02	9.17E-01	5.09E-01	9.77E-01	1.00E+00	9.52E-01	hypothetical protein
KO:K09990	0.23	0.00	0.00	0.00	0.00	0.00	0.00	5.84E-02	1.00E+00	1.00E+00	1.00E+00	1.00E+00	1.00E+00	1.00E+00	hypothetical protein
KO:K09991	0.06	0.00	0.00	1.25	0.00	0.00	0.00	3.82E-01	1.00E+00	1.00E+00	3.80E-02	1.00E+00	1.00E+00	1.00E+00	hypothetical protein
KO:K09992	2.65	0.09	9.05	0.52	0.50	0.00	1.91	1.84E-01	8.06E-01	2.83E-02	6.33E-01	6.41E-01	1.00E+00	2.66E-01	hypothetical protein
KO:K10001	0.00	5.89	0.00	0.00	0.00	0.00	0.00	1.00E+00	4.26E-02	1.00E+00	1.00E+00	1.00E+00	1.00E+00	1.00E+00	glutamate/aspartate transport system substrate-binding protein
KO:K10002	0.00	0.83	0.00	0.03	0.00	0.00	0.00	1.00E+00	5.14E-02	1.00E+00	3.49E-01	1.00E+00	1.00E+00	1.00E+00	glutamate/aspartate transport system permease protein
KO:K10003	0.00	2.49	0.00	0.10	0.00	0.00	0.00	1.00E+00	4.87E-02	1.00E+00	3.73E-01	1.00E+00	1.00E+00	1.00E+00	glutamate/aspartate transport system permease protein
KO:K10004	0.00	0.26	0.00	0.02	0.00	0.00	0.00	1.00E+00	7.56E-02	1.00E+00	2.68E-01	1.00E+00	1.00E+00	1.00E+00	glutamate/aspartate transport system ATP-binding protein
KO:K10008	0.00	0.00	0.00	0.10	0.00	0.00	0.00	1.00E+00	1.00E+00	1.00E+00	1.43E-01	1.00E+00	1.00E+00	1.00E+00	glutamate transport system ATP-binding protein [EC:3.6.3.-]
KO:K10010	0.00	0.11	0.00	0.00	0.00	0.00	0.00	1.00E+00	1.01E-01	1.00E+00	1.00E+00	1.00E+00	1.00E+00	1.00E+00	cystine transport system ATP-binding protein [EC:3.6.3.-]
KO:K10012	1.52	0.00	0.11	0.11	0.00	0.00	0.00	8.44E-02	1.00E+00	3.09E-01	3.13E-01	1.00E+00	1.00E+00	1.00E+00	undecaprenyl-phosphate 4-deoxy-4-formamido-L-arabinose transferase
KO:K10013	0.00	1.33	0.00	0.00	0.00	0.00	0.00	1.00E+00	1.01E-01	1.00E+00	1.00E+00	1.00E+00	1.00E+00	1.00E+00	lysine/arginine/ornithine transport system substrate-binding protein
KO:K10014	0.00	0.00	0.00	2.04	0.00	0.00	0.00	1.00E+00	1.00E+00	1.00E+00	1.43E-01	1.00E+00	1.00E+00	1.00E+00	histidine transport system substrate-binding protein
KO:K10016	0.00	1.67	0.00	0.00	0.00	0.00	0.00	1.00E+00	1.02E-01	1.00E+00	1.00E+00	1.00E+00	1.00E+00	1.00E+00	histidine transport system permease protein
KO:K10017	0.37	0.13	0.00	0.07	0.00	0.00	0.08	1.54E-01	2.69E-01	1.00E+00	3.20E-01	1.00E+00	1.00E+00	3.13E-01	histidine transport system ATP-binding protein [EC:3.6.3.21]
KO:K10018	0.33	0.00	0.00	4.96	0.00	0.00	0.00	3.27E-01	1.00E+00	1.00E+00	5.23E-02	1.00E+00	1.00E+00	1.00E+00	octopine/nopaline transport system substrate-binding protein
KO:K10019	0.17	0.00	0.00	2.42	0.00	0.00	0.00	2.86E-01	1.00E+00	1.00E+00	6.30E-02	1.00E+00	1.00E+00	1.00E+00	octopine/nopaline transport system permease protein
KO:K10020	0.00	0.00	0.00	4.59	0.00	0.00	0.00	1.00E+00	1.00E+00	1.00E+00	4.94E-02	1.00E+00	1.00E+00	1.00E+00	octopine/nopaline transport system permease protein
KO:K10021	0.16	0.04	0.00	0.00	0.00	0.00	0.00	9.79E-02	2.09E-01	1.00E+00	1.00E+00	1.00E+00	1.00E+00	1.00E+00	octopine/nopaline transport system ATP-binding protein [EC:3.6.3.-]
KO:K10022	0.70	0.00	0.00	0.00	0.00	0.00	0.00	6.29E-02	1.00E+00	1.00E+00	1.00E+00	1.00E+00	1.00E+00	1.00E+00	arginine/ornithine transport system substrate-binding protein
KO:K10024	0.00	0.67	0.00	0.00	0.00	0.00	0.00	1.00E+00	9.96E-02	1.00E+00	1.00E+00	1.00E+00	1.00E+00	1.00E+00	arginine/ornithine transport system permease protein
KO:K10026	7.73	0.04	0.61	0.46	1.70	0.00	2.10	2.41E-02	8.92E-01	6.89E-01	7.43E-01	3.18E-01	1.00E+00	2.41E-01	queuosine biosynthesis protein QueE
KO:K10027	8.97	3.86	0.46	8.10	0.00	3.86	0.32	6.74E-02	4.23E-01	9.35E-01	8.44E-02	1.00E+00	4.23E-01	9.43E-01	phytoene dehydrogenase [EC:1.14.99.-]
KO:K10036	0.00	1.02	0.00	0.01	0.00	0.00	0.00	1.00E+00	5.63E-02	1.00E+00	3.05E-01	1.00E+00	1.00E+00	1.00E+00	glutamine transport system substrate-binding protein
KO:K10037	0.00	0.11	0.00	0.00	0.00	0.00	0.00	1.00E+00	9.89E-02	1.00E+00	1.00E+00	1.00E+00	1.00E+00	1.00E+00	glutamine transport system permease protein
KO:K10039	0.64	1.66	0.04	0.50	0.04	0.00	0.00	2.49E-01	1.55E-01	5.06E-01	2.86E-01	5.06E-01	1.00E+00	1.00E+00	putative glutamine transport system substrate-binding protein
KO:K10040	0.33	0.67	0.00	0.96	0.00	0.00	0.00	2.69E-01	1.97E-01	1.00E+00	1.62E-01	1.00E+00	1.00E+00	1.00E+00	putative glutamine transport system permease protein
KO:K10041	0.11	0.88	0.00	0.74	0.00	0.00	0.00	3.68E-01	1.49E-01	1.00E+00	1.64E-01	1.00E+00	1.00E+00	1.00E+00	putative glutamine transport system ATP-binding protein [EC:3.6.3.-]
KO:K10046	0.15	0.00	0.00	0.00	0.00	24.73	0.00	2.50E-01	1.00E+00	1.00E+00	1.00E+00	1.00E+00	4.71E-03	1.00E+00	GDP-D-mannose 3', 5'-epimerase [EC:5.1.3.18 5.1.3.-]

KO:K10094	0.23	0.00	0.00	0.00	0.00	0.00	0.00	6.03E-02	1.00E+00	1.00E+00	1.00E+00	1.00E+00	1.00E+00	1.00E+00	nickel transport system substrate-binding protein
KO:K10107	0.00	0.93	0.00	0.00	5.25	0.00	0.00	1.00E+00	8.30E-02	1.00E+00	1.00E+00	8.43E-03	1.00E+00	1.00E+00	capsular polysaccharide transport system permease protein
KO:K10108	0.00	0.65	0.32	0.58	0.00	0.00	0.00	4.82E-01	1.73E-01	2.22E-01	1.83E-01	1.00E+00	1.00E+00	1.00E+00	maltose/maltodextrin transport system substrate-binding protein
KO:K10109	0.02	0.50	0.00	0.01	0.00	0.00	0.00	2.28E-01	9.70E-02	1.00E+00	2.72E-01	1.00E+00	1.00E+00	1.00E+00	maltose/maltodextrin transport system permease protein
KO:K10110	0.00	0.51	0.00	0.05	0.00	0.00	0.00	1.00E+00	6.21E-02	1.00E+00	2.38E-01	1.00E+00	1.00E+00	1.00E+00	maltose/maltodextrin transport system permease protein
KO:K10111	0.00	5.81	0.00	0.82	0.00	0.00	0.26	7.15E-01	4.01E-02	1.00E+00	3.16E-01	1.00E+00	1.00E+00	5.17E-01	maltose/maltodextrin transport system ATP-binding protein
KO:K10112	0.25	0.53	2.50	0.45	0.02	0.00	0.10	4.76E-01	3.24E-01	5.76E-02	3.62E-01	7.19E-01	1.00E+00	6.18E-01	maltose/maltodextrin transport system ATP-binding protein
KO:K10116	0.00	2.11	0.00	0.00	0.00	0.00	0.00	1.00E+00	4.80E-02	1.00E+00	1.00E+00	1.00E+00	1.00E+00	1.00E+00	maltooligosaccharide transport system ATP-binding protein
KO:K10117	0.00	0.11	0.00	0.00	0.00	0.00	0.00	1.00E+00	1.02E-01	1.00E+00	1.00E+00	1.00E+00	1.00E+00	1.00E+00	multiple sugar transport system substrate-binding protein
KO:K10119	0.00	0.11	0.00	0.00	0.00	0.00	0.00	1.00E+00	1.01E-01	1.00E+00	1.00E+00	1.00E+00	1.00E+00	1.00E+00	multiple sugar transport system permease protein
KO:K10125	0.18	26.04	0.00	0.02	0.00	0.00	0.00	8.37E-01	4.29E-04	1.00E+00	9.11E-01	1.00E+00	1.00E+00	1.00E+00	two-component system, NtrC family, C4-dicarboxylate transport sensor
KO:K10126	0.24	3.77	0.00	0.11	0.00	0.00	0.07	5.14E-01	1.36E-02	1.00E+00	7.07E-01	1.00E+00	1.00E+00	7.63E-01	two-component system, NtrC family, C4-dicarboxylate transport
KO:K10133	0.00	0.00	0.00	0.10	0.00	0.00	0.00	1.00E+00	1.00E+00	1.00E+00	1.43E-01	1.00E+00	1.00E+00	1.00E+00	tumor protein p53-inducible protein 3 [EC:1.-.-.-]
KO:K10188	0.00	0.00	0.00	0.31	0.00	0.00	0.00	1.00E+00	1.00E+00	1.00E+00	1.43E-01	1.00E+00	1.00E+00	1.00E+00	lactose/L-arabinose transport system substrate-binding protein
KO:K10189	0.00	0.00	0.00	0.00	20.93	0.00	1.35	1.00E+00	1.00E+00	1.00E+00	1.00E+00	1.29E-03	1.00E+00	1.31E-01	lactose/L-arabinose transport system permease protein
KO:K10190	0.00	0.00	0.00	0.00	20.00	0.00	0.00	1.00E+00	1.00E+00	1.00E+00	1.00E+00	0.00E+00	1.00E+00	1.00E+00	lactose/L-arabinose transport system permease protein
KO:K10191	0.00	0.58	0.00	0.86	0.00	0.00	0.00	5.66E-01	1.54E-01	1.00E+00	1.06E-01	1.00E+00	1.00E+00	1.00E+00	lactose/L-arabinose transport system ATP-binding protein
KO:K10195	0.00	0.22	0.00	0.00	0.00	0.00	0.00	1.00E+00	1.03E-01	1.00E+00	1.00E+00	1.00E+00	1.00E+00	1.00E+00	oligogalacturonide transport system ATP-binding protein
KO:K10206	0.02	0.00	0.28	0.01	24.82	0.00	0.02	6.30E-01	1.00E+00	4.07E-01	6.36E-01	2.86E-04	1.00E+00	6.28E-01	LL-diaminopimelate aminotransferase [EC:2.6.1.83]
KO:K10210	0.10	0.00	0.00	0.67	0.00	0.00	0.00	1.77E-01	1.00E+00	1.00E+00	1.44E-01	1.00E+00	1.00E+00	1.00E+00	4,4'-diaponeurosporene oxidase [EC:1.-.-.-]
KO:K10217	1.32	0.00	0.00	0.60	0.00	0.00	0.00	1.18E-01	1.00E+00	1.00E+00	1.87E-01	1.00E+00	1.00E+00	1.00E+00	aminomuconate-semialdehyde dehydrogenase [EC:1.2.1.32]
KO:K10218	0.00	1.32	0.00	0.25	0.00	0.00	0.00	1.00E+00	9.13E-02	1.00E+00	2.15E-01	1.00E+00	1.00E+00	1.00E+00	4-hydroxy-4-methyl-2-oxoglutarate aldolase [EC:4.1.3.17]
KO:K10219	2.30	1.75	0.00	0.57	0.00	0.00	0.00	1.45E-01	1.83E-01	1.00E+00	3.51E-01	1.00E+00	1.00E+00	1.00E+00	4-carboxy-2-hydroxymuconate-6-semialdehyde dehydrogenase
KO:K10220	2.56	0.03	0.00	1.61	0.00	0.00	0.00	1.09E-01	5.24E-01	1.00E+00	1.53E-01	1.00E+00	1.00E+00	1.00E+00	4-oxalmesaconate hydratase [EC:4.2.1.83]
KO:K10221	0.55	1.65	0.00	1.18	0.00	0.00	0.00	3.10E-01	1.64E-01	1.00E+00	2.11E-01	1.00E+00	1.00E+00	1.00E+00	2-pyrone-4,6-dicarboxylate lactonase [EC:3.1.1.57]
KO:K10222	1.63	0.00	0.00	0.00	0.00	0.00	0.00	6.11E-02	1.00E+00	1.00E+00	1.00E+00	1.00E+00	1.00E+00	1.00E+00	2,6-dioxo-6-phenylhexa-3-enoate hydrolase [EC:3.7.1.8]
KO:K10227	2.91	24.30	0.00	2.88	0.00	0.00	0.06	4.62E-01	5.14E-03	1.00E+00	4.67E-01	1.00E+00	1.00E+00	9.16E-01	sorbitol/mannitol transport system substrate-binding protein
KO:K10228	1.40	23.04	0.00	2.64	0.00	0.00	0.00	6.53E-01	4.71E-03	1.00E+00	4.01E-01	1.00E+00	1.00E+00	1.00E+00	sorbitol/mannitol transport system permease protein
KO:K10229	0.83	16.14	0.00	2.46	0.00	0.00	0.00	6.97E-01	6.29E-03	1.00E+00	2.99E-01	1.00E+00	1.00E+00	1.00E+00	sorbitol/mannitol transport system permease protein
KO:K10230	1.72	16.32	0.00	3.51	0.00	0.00	0.00	5.43E-01	9.43E-03	1.00E+00	2.43E-01	1.00E+00	1.00E+00	1.00E+00	sorbitol/mannitol transport system ATP-binding protein
KO:K10231	4.13	0.37	0.00	0.45	0.03	0.00	0.00	5.67E-02	4.46E-01	1.00E+00	4.16E-01	6.21E-01	1.00E+00	1.00E+00	kojibiose phosphorylase [EC:2.4.1.23]
KO:K10232	0.60	26.34	0.00	1.32	0.00	0.00	0.00	7.69E-01	3.71E-03	1.00E+00	5.97E-01	1.00E+00	1.00E+00	1.00E+00	alpha-glucoside transport system substrate-binding protein
KO:K10233	0.05	7.26	0.00	0.64	0.00	0.00	0.00	7.62E-01	1.74E-02	1.00E+00	3.68E-01	1.00E+00	1.00E+00	1.00E+00	alpha-glucoside transport system permease protein
KO:K10234	0.02	3.23	0.00	0.45	0.00	0.00	0.00	7.02E-01	3.37E-02	1.00E+00	2.73E-01	1.00E+00	1.00E+00	1.00E+00	alpha-glucoside transport system permease protein
KO:K10235	0.37	2.52	0.00	0.46	0.00	0.00	0.00	3.57E-01	6.97E-02	1.00E+00	3.17E-01	1.00E+00	1.00E+00	1.00E+00	alpha-glucoside transport system ATP-binding protein
KO:K10236	0.00	3.12	0.00	1.56	0.00	0.00	0.00	1.00E+00	8.04E-02	1.00E+00	1.63E-01	1.00E+00	1.00E+00	1.00E+00	trehalose/maltose transport system substrate-binding protein
KO:K10237	0.00	0.87	0.00	0.73	0.00	0.00	0.00	1.00E+00	1.31E-01	1.00E+00	1.46E-01	1.00E+00	1.00E+00	1.00E+00	trehalose/maltose transport system permease protein
KO:K10238	0.00	0.20	0.00	0.66	0.00	0.00	0.00	1.00E+00	2.09E-01	1.00E+00	6.99E-02	1.00E+00	1.00E+00	1.00E+00	trehalose/maltose transport system permease protein
KO:K10239	1.52	0.42	0.00	0.00	0.00	0.00	0.00	1.04E-01	1.90E-01	1.00E+00	1.00E+00	1.00E+00	1.00E+00	1.00E+00	trehalose/maltose transport system ATP-binding protein
KO:K10253	0.00	0.25	0.00	0.07	0.00	0.00	0.64	1.00E+00	1.63E-01	1.00E+00	3.59E-01	1.00E+00	1.00E+00	5.26E-02	DOPA 4,5-dioxygenase [EC:1.14.99.-]
KO:K10255	0.35	8.16	0.31	0.41	3.80	0.00	0.02	7.56E-01	2.24E-02	7.68E-01	7.33E-01	1.02E-01	1.00E+00	8.82E-01	omega-6 fatty acid desaturase (delta-12 desaturase) [EC:1.14.19.-]
KO:K10353	0.00	0.00	0.00	0.10	0.00	0.00	0.00	1.00E+00	1.00E+00	1.00E+00	1.43E-01	1.00E+00	1.00E+00	1.00E+00	deoxyadenosine kinase [EC:2.7.1.76]
KO:K10439	0.11	0.99	6.14	0.66	7.38	0.00	3.09	8.19E-01	5.32E-01	6.77E-02	6.30E-01	5.26E-02	1.00E+00	1.84E-01	ribose transport system substrate-binding protein
KO:K10440	0.27	1.75	5.02	1.03	4.67	0.00	0.05	7.47E-01	2.88E-01	5.83E-02	4.58E-01	6.73E-02	1.00E+00	8.60E-01	ribose transport system permease protein
KO:K10441	0.07	1.27	12.86	0.21	1.99	0.00	2.32	8.20E-01	3.64E-01	1.49E-02	7.35E-01	2.37E-01	1.00E+00	1.96E-01	ribose transport system ATP-binding protein [EC:3.6.3.17]
KO:K10533	2.25	0.00	0.00	0.08	0.00	0.00	0.00	5.29E-02	1.00E+00	1.00E+00	3.03E-01	1.00E+00	1.00E+00	1.00E+00	limonene-1,2-epoxide hydrolase [EC:3.3.2.8]
KO:K10536	2.29	0.01	0.65	0.70	0.02	0.00	1.57	8.91E-02	8.49E-01	3.60E-01	3.41E-01	8.28E-01	1.00E+00	1.50E-01	agmatine deiminase [EC:3.5.3.12]
KO:K10538	0.00	1.63	0.00	0.04	0.00	0.00	0.00	1.00E+00	6.96E-02	1.00E+00	2.34E-01	1.00E+00	1.00E+00	1.00E+00	L-arabinose transport system permease protein
KO:K10539	0.00	0.00	0.00	0.00	3.65	0.00	0.00	1.00E+00	1.00E+00	1.00E+00	2.53E-01	6.43E-03	1.00E+00	1.00E+00	L-arabinose transport system ATP-binding protein [EC:3.6.3.17]
KO:K10543	0.85	1.96	0.14	0.24	0.00	0.00	0.02	2.32E-01	1.07E-01	5.39E-01	4.55E-01	1.00E+00	1.00E+00	6.99E-01	D-xylose transport system substrate-binding protein
KO:K10544	1.16	1.54	0.00	0.47	0.00	0.00	0.02	1.80E-01	1.33E-01	1.00E+00	3.44E-01	1.00E+00	1.00E+00	7.04E-01	D-xylose transport system permease protein
KO:K10545	0.08	0.83	0.00	2.45	0.00	0.00	0.00	5.98E-01	1.92E-01	1.00E+00	5.77E-02	1.00E+00	1.00E+00	1.00E+00	D-xylose transport system ATP-binding protein [EC:3.6.3.17]
KO:K10546	0.00	0.87	0.00	0.73	0.00	0.00	0.00	1.00E+00	1.02E-01	1.00E+00	1.21E-01	1.00E+00	1.00E+00	1.00E+00	putative multiple sugar transport system substrate-binding protein
KO:K10547	0.00	2.01	0.00	0.00	0.00	0.00	0.00	1.00E+00	4.17E-02	1.00E+00	4.11E-01	1.00E+00	1.00E+00	1.00E+00	putative multiple sugar transport system permease protein
KO:K10548	0.00	0.04	0.00	0.13	0.00	0.00	0.00	1.00E+00	1.92E-01	1.00E+00	1.27E-01	1.00E+00	1.00E+00	1.00E+00	putative multiple sugar transport system ATP-binding protein
KO:K10552	0.82	19.91	0.00	3.75	0.00	0.00	0.00	7.44E-01	1.01E-02	1.00E+00	2.52E-01	1.00E+00	1.00E+00	1.00E+00	fructose transport system substrate-binding protein
KO:K10553	0.49	15.49	0.00	1.74	0.00	0.00	0.00	7.26E-01	6.57E-03	1.00E+00	3.79E-01	1.00E+00	1.00E+00	1.00E+00	fructose transport system permease protein
KO:K10554	0.19	14.31	0.00	1.42	0.00	0.00	0.00	8.14E-01	3.57E-03	1.00E+00	3.83E-01	1.00E+00	1.00E+00	1.00E+00	fructose transport system ATP-binding protein
KO:K10555	0.00	0.01	0.00	1.94	0.00	0.00	0.00	1.00E+00	1.96E-01	1.00E+00	1.15E-01	1.00E+00	1.00E+00	1.00E+00	AI-2 transport system substrate-binding protein
KO:K10556	0.00	0.00	0.00	1.94	0.00	0.00	0.00	1.00E+00	1.00E+00	1.00E+00	1.43E-01	1.00E+00	1.00E+00	1.00E+00	AI-2 transport system permease protein
KO:K10559	0.00	3.96	0.00	0.28	0.00	0.00	0.02	1.00E+00	5.36E-02	1.00E+00	3.27E-01	1.00E+00	1.00E+00	4.95E-01	rhamnose transport system substrate-binding protein
KO:K10560	0.00	1.66	0.00	0.07	0.00	0.00	0.00	1.00E+00	3.04E-02	1.00E+00	3.85E-01	1.00E+00	1.00E+00	1.00E+00	rhamnose transport system permease protein
KO:K10561	0.00	0.62	0.00	0.04	0.00	0.00	0.00	1.00E+00	7.31E-02	1.00E+00	2.67E-01	1.00E+00	1.00E+00	1.00E+00	rhamnose transport system permease protein
KO:K10562	0.01	2.18	0.00	0.11	0.00	0.00	0.00	5.65E-01	4.34E-02	1.00E+00	3.83E-01	1.00E+00	1.00E+00	1.00E+00	rhamnose transport system ATP-binding protein [EC:3.6.3.17]
KO:K10563	5.74	2.34	6.05	1.19	3.96	0.02	1.11	5.73E-02	5.31E-01	4.94E-02	8.55E-01	1.57E-01	9.95E-01	8.70E-01	formamidopyrimidine-DNA glycosylase [EC:3.2.2.23 4.2.99.18]
KO:K10616	0.00	0.00	0.00	3.57	0.00	0.00	0.00	1.00E+00	1.00E+00	1.00E+00	7.80E-02	1.00E+00	1.00E+00	1.00E+00	p-cymene monooxygenase [EC:1.14.13.-]
KO:K10617	0.00	0.00	0.00	2.04	0.00	0.00	0.00	1.00E+00	1.00E+00	1.00E+00	1.43E-01	1.00E+00	1.00E+00	1.00E+00	p-cumic alcohol dehydrogenase
KO:K10674	0.02	0.05	0.00	3.47	0.00	0.00	0.03	5.07E-01	4.31E-01	1.00E+00	5.26E-02	1.00E+00	1.00E+00	4.69E-01	ectoine hydroxylase [EC:1.14.11.-]
KO:K10676	0.00	3.14	0.00	0.51	0.00	0.00	0.00	1.00E+00	5.29E-02	1.00E+00	2.65E-01	1.00E+00	1.00E+00	1.00E+00	2,4-dichlorophenol 6-monooxygenase [EC:1.14.13.2]
KO:K10678	1.49	1.67	0.00	0.85	0.00	0.00	0.00	1.95E-01	1.73E-01	1.00E+00	2.95E-01	1.00E+00	1.00E+00	1.00E+00	nitroreductase [EC:1.-.-.-]
KO:K10680	0.70	0.00	2.92	0.06	0.00	0.00	0.02	1.34E-01	1.00E+00	2.53E-02	4.83E-01	1.00E+00	1.00E+00	5.94E-01	N-ethylmaleimide reductase [EC:1.-.-.-]
KO:K10697	0.00	0.00	0.00	0.00	27.50	0.00	0.00	1.00E+00	1.00E+00	1.00E+00	1.00E+00	0.00E+00	1.00E+00	1.00E+00	two-component system, OmpR family, response regulator RpaA
KO:K10713	0.21	0.00	14.06	1.21	2.25	0.00	0.44	6.28E-01	1.00E+00	9.86E-03	3.42E-01	1.91E-01	1.00E+00	5.45E-01	formaldehyde-activating enzyme [EC:4.3.-.-]
KO:K10714	0.68	0.00	0.00	0.52	0.00	0.00	0.00	1.20E-01	1.00E+00	1.00E+00	1.44E-01	1.00E+00	1.00E+00	1.00E+00	methylene-tetrahydromethanopterin dehydrogenase [EC:1.5.1.-]
KO:K10715	0.03	0.00	0.00	0.05	0.00	0.00	0.87	3.29E-01	1.00E+00	1.00E+00	2.90E-01	1.00E+00	1.00E+00	2.79E-02	two-component system, sensor histidine kinase RpfC [EC:2.7.13.3]
KO:K10725	0.00	0.00	0.00	0.00	0.00	0.00	4.58	1.00E+00	1.00E+00	1.00E+00	1.00E+00	1.00E+00	1.00E+00	1.40E-02	archaeal cell division control protein 6
KO:K10726	0.00	0.00	0.00	0.00	0.00	0.00	3.33	1.00E+00	1.00E+00	1.00E+00	1.00E+00	1.00E+00	1.00E+00	2.93E-02	replicative DNA helicase Mcm [EC:3.6.1.-]
KO:K10747	0.00	0.00	0.00	0.00	0.00	0.00	2.50	1.00E+00	1.00E+00	1.00E+00	1.00E+00	1.00E+00	1.00E+00	2.66E-02	DNA ligase 1 [EC:6.5.1.1]
KO:K10763	5.64	0.10	0.57	0.55	0.00	0.00	0.57	2.17E-02	8.06E-01	5.23E-01	5.35E-01	1.00E+00	1.00E+00	5.23E-01	DnaA-homolog protein
KO:K10764	6.61	12.60	0.14	4.63	0.00	3.32	2.06	1.41E-01	1.70E-02	9.90E-01	3.70E-01	1.00E+00	6.56E-01	8.69E-01	O-succinylhomoserine sulfhydrylase [EC:2.5.1.-]
KO:K10773	3.23	6.28	2.59	1.52	4.21	0.35	2.19	3.80E-01	6.61E-02	5.69E-01	8.36E-01	1.96E-01	9.70E-01	6.79E-01	endonuclease III [EC:4.2.99.18]
KO:K10775	0.00	0.00	0.00	0.00	1.25	0.00	0.00	1.00E+00	1.00E+00	1.00E+00	1.00E+00	1.64E-02	1.00E+00	1.00E+00	phenylalanine ammonia-lyase [EC:4.3.1.24]
KO:K10778	0.11	4.58	3.78	0.31	0.00	0.00	0.03	8.71E-01	1.66E-02	2.34E-02	7.40E-01	1.00E+00	1.00E+00	9.32E-01	AraC family transcriptional regulator, regulatory protein of
KO:K10780	0.00	0.00	0.94	0.00	0.00	0.00	0.10	1.00E+00	1.00E+00	2.11E-02	1.00E+00	1.00E+00	1.00E+00	1.46E-01	enoyl-[acyl carrier protein] reductase III [EC:1.3.1.-]
KO:K10783	0.25	0.00	6.41	0.15	0.00	0.00	0.28	3.50E-01	1.00E+00	1.64E-02	4.00E-01	1.00E+00	1.00E+00	3.38E-01	trans-2-enoyl-CoA reductase (NAD+) [EC:1.3.1.44]
KO:K10800	0.09	0.00	24.36	0.01	0.00	0.00	0.00	4.34E-01	1.00E+00	7.14E-04	4.91E-01	1.00E+00	1.00E+00	1.00E+00	single-strand selective monofunctional uracil DNA glycosylase
KO:K10804	2.55	0.22	6.84	0.21	0.00	0.70	0.00	9.13E-02	7.28E-01	1.37E-02	7.30E-01	1.00E+00	4.46E-01	1.00E+00	acyl-CoA thioesterase I [EC:3.1.2.- 3.1.1.5]
KO:K10805	2.74	1.15	0.59	4.40	0.00	0.00	0.01	1.49E-01	4.30E-01	6.45E-01	6.96E-02	1.00E+00	1.00E+00	9.13E-01	acyl-CoA thioesterase II [EC:3.1.2.-]
KO:K10806	5.21	0.28	0.79	0.28	0.00	0.00	0.00	3.19E-02	5.79E-01	3.34E-01	5.75E-01	1.00E+00	1.00E+00	1.00E+00	acyl-CoA thioesterase YciA [EC:3.1.2.-]
KO:K10807	2.90	3.32	0.00	0.19	0.00	0.00	0.00	1.34E-01	1.18E-01	1.00E+00	5.14E-01	1.00E+00	1.00E+00	1.00E+00	ribonucleoside-diphosphate reductase subunit M1 [EC:1.17.4.1]
KO:K10808	1.26	2.16	0.00	1.08	0.00	0.00	0.00	2.50E-01	1.62E-01	1.00E+00	2.74E-01	1.00E+00	1.00E+00	1.00E+00	ribonucleoside-diphosphate reductase subunit M2 [EC:1.17.4.1]
KO:K10815	0.12	0.00	0.00	0.00	0.00	0.00	0.00	6.34E-02	1.00E+00	1.00E+00	1.00E+00	1.00E+00	1.00E+00	1.00E+00	hydrogen cyanide synthase HcnB [EC:1.4.99.5]
KO:K10817	1.71	0.00	0.00	0.76	0.00	0.00	0.00	8.70E-02	1.00E+00	1.00E+00	2.01E-01	1.00E+00	1.00E+00	1.00E+00	erythronolide synthase [EC:2.3.1.94]
KO:K10819	0.60	0.24	0.05	0.12	0.05	0.38	0.00	1.46E-01	3.26E-01	6.12E-01	4.84E-01	6.12E-01	2.25E-01	1.00E+00	histidine kinase
KO:K10820	0.02	1.90	0.00	0.00	0.00	0.00	0.00	4.25E-01	3.59E-02	1.00E+00	1.00E+00	1.00E+00	1.00E+00	1.00E+00	monosaccharide-transporting ATPase [EC:3.6.3.17]
KO:K10826	0.09	0.00	0.00	0.00	5.53	0.00	0.00	2.72E-01	1.00E+00	1.00E+00	1.00E+00	4.00E-03	1.00E+00	1.00E+00	Fe3+-transporting ATPase [EC:3.6.3.3]

KO:K10834	0.67	0.00	0.00	0.41	0.00	0.00	3.49	2.20E-01	1.00E+00	1.00E+00	2.87E-01	1.00E+00	1.00E+00	5.63E-02	heme-transporting ATPase [EC:3.6.3.41]
KO:K10843	0.00	0.00	0.00	0.00	0.00	0.00	0.42	1.00E+00	1.00E+00	1.00E+00	1.00E+00	1.00E+00	1.00E+00	2.41E-02	DNA excision repair protein ERCC-3 [EC:3.6.1.-]
KO:K10844	0.16	0.00	0.00	0.03	0.00	0.00	0.00	9.31E-02	1.00E+00	1.00E+00	2.30E-01	1.00E+00	1.00E+00	1.00E+00	DNA excision repair protein ERCC-2 [EC:3.6.1.-]
KO:K10848	0.00	0.00	0.00	0.00	0.00	0.00	2.08	1.00E+00	1.00E+00	1.00E+00	1.00E+00	1.00E+00	1.00E+00	2.76E-02	DNA excision repair protein ERCC-4 [EC:3.1.-.-]
KO:K10852	0.00	0.00	0.00	0.20	0.00	0.00	0.00	1.00E+00	1.00E+00	1.00E+00	8.30E-02	1.00E+00	1.00E+00	1.00E+00	isopenicillin-N N-acyltransferase [EC:2.3.1.164]
KO:K10854	0.00	0.08	0.00	0.07	0.00	1.10	0.00	1.00E+00	2.51E-01	1.00E+00	2.61E-01	1.00E+00	4.86E-02	1.00E+00	acetone carboxylase, alpha subunit [EC:6.4.1.6]
KO:K10855	0.00	0.11	0.00	0.00	0.00	7.44	0.00	1.00E+00	2.65E-01	1.00E+00	1.00E+00	1.00E+00	3.71E-03	1.00E+00	acetone carboxylase, beta subunit [EC:6.4.1.6]
KO:K10856	0.00	0.00	0.00	0.01	0.00	3.14	0.00	1.00E+00	1.00E+00	1.00E+00	2.53E-01	1.00E+00	1.57E-03	1.00E+00	acetone carboxylase, gamma subunit [EC:6.4.1.6]
KO:K10857	0.00	0.00	0.00	1.20	0.00	0.00	0.09	1.00E+00	1.00E+00	1.00E+00	1.15E-01	1.00E+00	1.00E+00	1.74E-01	exodeoxyribonuclease X [EC:3.1.11.-]
KO:K10896	0.00	0.00	0.00	0.00	0.00	0.00	2.92	1.00E+00	1.00E+00	1.00E+00	1.00E+00	1.00E+00	1.00E+00	1.13E-02	fanconi anemia group M protein [EC:3.6.1.-]
KO:K10907	5.49	0.05	6.83	2.11	0.00	0.00	5.76	9.99E-02	9.00E-01	7.06E-02	3.91E-01	1.00E+00	1.00E+00	9.14E-02	aminotransferase [EC:2.6.1.-]
KO:K10908	0.37	0.00	0.00	0.00	1.20	0.00	0.00	1.57E-01	1.00E+00	1.00E+00	1.00E+00	7.13E-02	1.00E+00	1.00E+00	DNA-directed RNA polymerase, mitochondrial [EC:2.7.7.6]
KO:K10910	0.00	3.11	0.00	0.00	0.00	0.00	0.00	1.00E+00	4.21E-02	1.00E+00	1.00E+00	1.00E+00	1.00E+00	1.00E+00	autoinducer 2-binding periplasmic protein LuxP
KO:K10912	6.33	0.00	0.00	0.64	0.00	0.00	0.01	3.19E-02	6.78E-01	1.00E+00	3.33E-01	1.00E+00	1.00E+00	6.54E-01	two-component system, repressor protein LuxO
KO:K10913	0.00	0.00	0.00	0.00	0.00	0.00	0.42	1.00E+00	1.00E+00	1.00E+00	1.00E+00	1.00E+00	1.00E+00	2.64E-02	TetR/AcrR family transcriptional regulator, hemagglutinin/protease
KO:K10914	7.28	0.00	1.05	0.48	0.00	0.00	0.18	2.64E-02	1.00E+00	3.10E-01	5.11E-01	1.00E+00	1.00E+00	6.62E-01	CRP/FNR family transcriptional regulator, cyclic AMP receptor
KO:K10918	0.12	0.00	0.00	0.00	0.00	0.00	0.00	6.20E-02	1.00E+00	1.00E+00	1.00E+00	1.00E+00	1.00E+00	1.00E+00	LysR family transcriptional regulator, transcriptional activator
KO:K10924	0.65	0.00	0.00	0.41	0.00	0.00	0.00	1.21E-01	1.00E+00	1.00E+00	1.68E-01	1.00E+00	1.00E+00	1.00E+00	MSHA pilin protein MshA
KO:K10925	0.28	0.00	3.27	0.01	0.00	0.00	0.00	2.05E-01	1.00E+00	2.06E-02	4.54E-01	1.00E+00	1.00E+00	1.00E+00	MSHA pilin protein MshB
KO:K10926	0.04	0.00	0.31	0.13	0.00	0.00	0.00	2.87E-01	1.00E+00	7.06E-02	1.94E-01	1.00E+00	1.00E+00	1.00E+00	MSHA pilin protein MshC
KO:K10927	0.59	0.00	0.00	0.08	0.00	0.00	0.00	4.89E-02	1.00E+00	1.00E+00	3.00E-01	1.00E+00	1.00E+00	1.00E+00	MSHA pilin protein MshD
KO:K10941	11.92	0.18	0.68	0.35	0.33	1.21	0.37	1.43E-02	8.26E-01	6.81E-01	7.91E-01	7.95E-01	5.26E-01	7.87E-01	sigma-54 specific transcriptional regulator, flagellar regulatory
KO:K10942	5.77	0.01	1.67	0.16	0.00	0.00	0.04	2.44E-02	7.84E-01	1.55E-01	6.37E-01	1.00E+00	1.00E+00	7.37E-01	two-component system, sensor histidine kinase FlrB [EC:2.7.13.3]
KO:K10943	10.85	0.74	1.08	0.64	0.00	5.75	0.42	2.90E-02	7.52E-01	6.65E-01	7.73E-01	1.00E+00	9.57E-02	8.15E-01	two component system, response regulator FlrC
KO:K10945	0.00	0.00	0.00	0.31	0.00	0.00	0.00	1.00E+00	1.00E+00	1.00E+00	1.43E-01	1.00E+00	1.00E+00	1.00E+00	ammonia monooxygenase subunit B [EC:1.13.12.-]
KO:K10947	0.09	0.20	0.00	0.59	0.00	2.10	0.23	6.01E-01	4.69E-01	1.00E+00	2.09E-01	1.00E+00	3.87E-02	4.35E-01	PadR family transcriptional regulator, regulatory protein PadR
KO:K10954	0.12	0.00	0.00	0.00	0.00	0.00	0.00	6.03E-02	1.00E+00	1.00E+00	1.00E+00	1.00E+00	1.00E+00	1.00E+00	zona occludens toxin
KO:K10956	0.00	0.00	0.00	0.00	0.00	0.00	1.25	1.00E+00	1.00E+00	1.00E+00	1.00E+00	1.00E+00	1.00E+00	2.74E-02	protein transport protein SEC61 subunit alpha
KO:K10960	0.48	3.31	0.00	0.26	27.35	0.00	0.00	7.32E-01	1.43E-01	1.00E+00	7.94E-01	1.43E-04	1.00E+00	1.00E+00	geranylgeranyl reductase [EC:1.3.1.83]
KO:K10974	1.05	0.00	0.00	0.00	0.00	0.00	0.00	3.90E-02	1.00E+00	1.00E+00	1.00E+00	1.00E+00	1.00E+00	1.00E+00	cytosine permease
KO:K10977	0.00	0.00	0.00	0.00	0.00	0.00	1.67	1.00E+00	1.00E+00	1.00E+00	1.00E+00	1.00E+00	1.00E+00	2.54E-02	trans-homoaconitate synthase [EC:4.1.3.-]
KO:K10979	0.00	0.00	0.00	0.10	0.00	0.00	0.00	1.00E+00	1.00E+00	1.00E+00	1.43E-01	1.00E+00	1.00E+00	1.00E+00	DNA end-binding protein Ku
KO:K11031	0.00	0.56	0.00	0.00	0.00	0.00	0.00	1.00E+00	5.01E-02	1.00E+00	1.00E+00	1.00E+00	1.00E+00	1.00E+00	thiol-activated cytolysin
KO:K11065	1.52	0.01	4.87	1.49	0.00	0.00	0.11	1.68E-01	8.46E-01	3.26E-02	1.73E-01	1.00E+00	1.00E+00	7.59E-01	thiol peroxidase, atypical 2-Cys peroxiredoxin [EC:1.11.1.15]
KO:K11066	0.00	1.11	0.00	0.00	0.00	0.00	0.00	1.00E+00	4.60E-02	1.00E+00	1.00E+00	1.00E+00	1.00E+00	1.00E+00	N-acetylmuramoyl-L-alanine amidase [EC:3.5.1.28]
KO:K11068	0.28	0.37	2.66	0.21	0.00	0.63	0.04	4.86E-01	4.02E-01	2.46E-02	5.63E-01	1.00E+00	2.42E-01	7.90E-01	hemolysin III
KO:K11069	0.00	7.78	0.00	2.33	0.00	0.00	0.00	7.27E-01	3.69E-02	1.00E+00	1.87E-01	1.00E+00	1.00E+00	1.00E+00	spermidine/putrescine transport system substrate-binding protein
KO:K11070	0.00	4.61	0.00	0.75	0.00	0.00	0.00	6.74E-01	3.90E-02	1.00E+00	2.60E-01	1.00E+00	1.00E+00	1.00E+00	spermidine/putrescine transport system permease protein
KO:K11071	0.02	6.76	0.03	0.63	0.00	0.00	0.00	6.85E-01	3.09E-02	6.79E-01	3.68E-01	1.00E+00	1.00E+00	1.00E+00	spermidine/putrescine transport system permease protein
KO:K11072	0.92	1.01	0.07	0.04	0.00	0.00	0.00	1.27E-01	1.16E-01	4.64E-01	5.26E-01	1.00E+00	1.00E+00	1.00E+00	spermidine/putrescine transport system ATP-binding protein
KO:K11073	2.49	13.39	1.56	1.90	0.00	0.00	0.24	4.21E-01	1.57E-02	6.28E-01	5.43E-01	1.00E+00	1.00E+00	8.91E-01	putrescine transport system substrate-binding protein
KO:K11074	1.46	6.32	0.08	0.17	0.00	0.00	0.00	1.97E-01	2.16E-02	8.35E-01	7.74E-01	1.00E+00	1.00E+00	1.00E+00	putrescine transport system permease protein
KO:K11075	1.88	3.42	0.00	0.12	0.00	0.00	0.10	9.84E-02	4.10E-02	1.00E+00	7.45E-01	1.00E+00	1.00E+00	7.76E-01	putrescine transport system permease protein
KO:K11076	2.15	4.38	0.22	1.04	0.00	0.00	0.06	1.51E-01	4.17E-02	8.35E-01	4.22E-01	1.00E+00	1.00E+00	9.06E-01	putrescine transport system ATP-binding protein
KO:K11085	6.59	1.79	5.87	4.36	1.94	5.49	1.59	1.41E-01	8.36E-01	1.92E-01	3.71E-01	8.17E-01	2.26E-01	8.61E-01	ATP-binding cassette, subfamily B, bacterial MsbA [EC:3.6.3.-]
KO:K11089	0.02	0.00	0.24	0.03	1.53	0.00	0.24	5.34E-01	1.00E+00	1.93E-01	5.11E-01	2.26E-02	1.00E+00	1.93E-01	6 kDa SS-A/Ro ribonucleoprotein
KO:K11105	0.02	0.00	0.10	0.13	0.00	4.46	0.90	6.16E-01	6.87E-01	4.35E-01	4.05E-01	1.00E+00	1.21E-02	1.02E-01	cell volume regulation protein A
KO:K11107	0.00	0.00	0.00	0.00	0.00	0.00	2.81	1.00E+00	3.95E-01	1.00E+00	1.00E+00	1.00E+00	1.00E+00	3.57E-03	ferredoxin
KO:K11145	0.00	0.00	0.00	0.00	13.13	0.00	0.00	1.00E+00	1.00E+00	1.00E+00	1.00E+00	0.00E+00	1.00E+00	1.00E+00	ribonuclease III family protein
KO:K11147	0.00	0.00	0.00	1.53	0.00	0.00	0.00	1.00E+00	1.00E+00	1.00E+00	1.43E-01	1.00E+00	1.00E+00	1.00E+00	dehydrogenase/reductase SDR family member 4 [EC:1.1.-.-]
KO:K11154	0.70	0.00	0.00	0.00	0.00	0.00	0.00	6.37E-02	1.00E+00	1.00E+00	1.00E+00	1.00E+00	1.00E+00	1.00E+00	retinol dehydrogenase 16 [EC:1.1.1.-]
KO:K11159	6.74	0.13	0.00	1.00	0.00	0.00	0.00	3.87E-02	5.71E-01	1.00E+00	3.12E-01	1.00E+00	1.00E+00	1.00E+00	carotenoid cleavage dioxygenase
KO:K11165	0.27	0.00	0.00	0.00	0.00	0.00	1.12	1.49E-01	1.00E+00	1.00E+00	1.00E+00	1.00E+00	1.00E+00	8.20E-02	dehydrogenase/reductase SDR family member 7 [EC:1.1.-.-]
KO:K11168	0.00	0.00	0.00	0.10	0.00	0.00	0.00	1.00E+00	1.00E+00	1.00E+00	1.43E-01	1.00E+00	1.00E+00	1.00E+00	dehydrogenase/reductase SDR family member 12 [EC:1.1.-.-]
KO:K11173	1.14	0.00	0.00	0.32	0.00	0.00	0.00	9.24E-02	1.00E+00	1.00E+00	2.30E-01	1.00E+00	1.00E+00	1.00E+00	hydroxyacid-oxoacid transhydrogenase [EC:1.1.99.24]
KO:K11175	2.21	4.65	1.96	2.00	4.55	0.42	1.03	5.40E-01	8.63E-02	6.36E-01	6.24E-01	9.11E-02	9.61E-01	8.80E-01	phosphoribosylglycinamide formyltransferase 1 [EC:2.1.2.2]
KO:K11176	0.00	0.00	9.60	0.00	0.00	0.00	0.02	1.00E+00	1.00E+00	1.41E-02	1.00E+00	1.00E+00	1.00E+00	1.66E-01	IMP cyclohydrolase [EC:3.5.4.1]
KO:K11177	0.01	0.03	4.28	0.00	0.00	0.00	0.48	4.71E-01	4.01E-01	1.76E-02	1.00E+00	1.00E+00	1.00E+00	1.52E-01	xanthine dehydrogenase YagR molybdenum-binding subunit [EC:1.17.1.4]
KO:K11178	0.00	0.07	1.24	0.09	0.00	0.00	0.00	1.00E+00	3.09E-01	2.11E-02	2.72E-01	1.00E+00	1.00E+00	1.00E+00	xanthine dehydrogenase YagS FAD-binding subunit [EC:1.17.1.4]
KO:K11179	2.20	0.02	0.00	0.30	0.00	6.36	9.96	2.15E-01	8.13E-01	8.49E-01	6.32E-01	1.00E+00	4.90E-02	2.21E-02	tRNA 2-thiouridine synthesizing protein E [EC:2.8.1.-]
KO:K11180	0.10	0.00	0.00	0.16	0.00	17.32	5.35	4.72E-01	1.00E+00	1.00E+00	4.54E-01	1.00E+00	1.61E-02	6.83E-02	sulfite reductase, dissimilatory-type alpha subunit [EC:1.8.99.3]
KO:K11181	0.09	0.00	0.00	0.46	0.00	15.53	5.11	5.16E-01	1.00E+00	1.00E+00	3.88E-01	1.00E+00	1.43E-02	6.60E-02	sulfite reductase, dissimilatory-type beta subunit [EC:1.8.99.3]
KO:K11183	0.02	0.00	0.00	0.42	0.00	0.00	0.62	4.13E-01	1.00E+00	1.00E+00	1.26E-01	1.00E+00	1.00E+00	8.67E-02	phosphocarrier protein FPr
KO:K11187	0.00	0.00	0.00	1.33	0.00	0.00	0.00	1.00E+00	1.00E+00	1.00E+00	1.43E-01	1.00E+00	1.00E+00	1.00E+00	peroxiredoxin 5, atypical 2-Cys peroxiredoxin [EC:1.11.1.15]
KO:K11189	0.53	3.68	4.89	0.46	1.42	0.00	0.39	7.30E-01	7.66E-02	4.46E-02	7.59E-01	3.65E-01	1.00E+00	7.91E-01	phosphocarrier protein
KO:K11208	0.00	0.11	0.00	1.09	0.00	0.00	0.00	1.00E+00	2.65E-01	1.00E+00	5.21E-02	1.00E+00	1.00E+00	1.00E+00	GST-like protein
KO:K11209	6.42	10.72	0.29	1.88	0.00	0.00	0.10	6.40E-02	1.79E-02	9.22E-01	5.86E-01	1.00E+00	1.00E+00	9.53E-01	GST-like protein
KO:K11211	2.23	0.00	0.00	0.00	0.00	0.00	0.00	1.97E-02	1.00E+00	1.00E+00	5.60E-01	1.00E+00	1.00E+00	1.00E+00	3-deoxy-D-manno-octulosonic acid kinase [EC:2.7.1.-]
KO:K11212	1.97	0.02	0.00	0.19	0.00	0.00	3.97	1.39E-01	5.90E-01	1.00E+00	4.55E-01	1.00E+00	1.00E+00	6.16E-02	LPPG:FO 2-phospho-L-lactate transferase [EC:2.7.8.-]
KO:K11258	0.06	0.00	0.00	0.05	0.00	0.00	0.00	1.06E-01	1.00E+00	1.00E+00	2.06E-01	1.00E+00	1.00E+00	1.00E+00	acetolactate synthase II small subunit [EC:2.2.1.6]
KO:K11263	1.50	0.02	0.00	0.13	0.00	0.00	0.00	9.21E-02	3.01E-01	1.00E+00	2.48E-01	1.00E+00	1.00E+00	1.00E+00	acetyl-/propionyl-CoA carboxylase, biotin carboxylase, biotin
KO:K11264	0.00	0.00	0.00	0.10	0.00	0.00	0.00	1.00E+00	1.00E+00	1.00E+00	1.43E-01	1.00E+00	1.00E+00	1.00E+00	methylmalonyl-CoA decarboxylase [EC:4.1.1.41]
KO:K11275	0.61	0.00	0.00	0.01	0.00	0.00	0.00	4.60E-02	1.00E+00	1.00E+00	2.81E-01	1.00E+00	1.00E+00	1.00E+00	histone H1/5
KO:K11312	0.00	0.00	0.00	0.00	13.86	0.00	0.01	4.97E-01	1.00E+00	1.00E+00	1.00E+00	1.43E-04	1.00E+00	4.47E-01	cupin 2 domain-containing protein
KO:K11325	0.00	0.00	0.00	0.00	11.88	0.00	0.00	1.00E+00	1.00E+00	1.00E+00	1.00E+00	1.43E-04	1.00E+00	1.00E+00	L-cysteine/cystine lyase
KO:K11326	0.00	0.00	0.00	0.00	2.50	0.00	0.00	1.00E+00	1.00E+00	1.00E+00	1.00E+00	1.37E-02	1.00E+00	1.00E+00	cation efflux system protein involved in nickel and cobalt tolerance
KO:K11327	0.00	0.00	0.00	0.00	0.63	0.00	0.00	1.00E+00	1.00E+00	1.00E+00	1.00E+00	1.71E-02	1.00E+00	1.00E+00	cation efflux system protein involved in nickel and cobalt tolerance
KO:K11329	0.04	0.00	0.00	0.00	29.65	0.00	0.00	4.98E-01	1.00E+00	1.00E+00	1.00E+00	0.00E+00	1.00E+00	1.00E+00	two-component system, OmpR family, response regulator RpaB
KO:K11333	1.41	8.62	0.00	1.38	0.00	0.00	0.00	3.75E-01	2.73E-02	1.00E+00	3.82E-01	1.00E+00	1.00E+00	1.00E+00	chlorophyllide reductase iron protein subunit X [EC:1.18.6.1]
KO:K11334	1.37	7.98	0.00	1.63	0.00	0.00	0.00	3.79E-01	2.69E-02	1.00E+00	3.20E-01	1.00E+00	1.00E+00	1.00E+00	chlorophyllide reductase subunit Y [EC:1.18.1.-]
KO:K11335	1.45	8.55	0.00	1.16	0.00	0.00	0.00	3.46E-01	2.10E-02	1.00E+00	4.26E-01	1.00E+00	1.00E+00	1.00E+00	chlorophyllide reductase subunit Z [EC:1.18.-.-]
KO:K11336	1.02	8.87	0.00	2.35	0.00	0.00	0.00	5.11E-01	2.26E-02	1.00E+00	2.37E-01	1.00E+00	1.00E+00	1.00E+00	3-vinyl bacteriochlorophyllide hydratase [EC:4.2.1.-]
KO:K11337	1.35	8.18	0.00	1.90	0.00	0.00	0.00	3.92E-01	2.23E-02	1.00E+00	2.75E-01	1.00E+00	1.00E+00	1.00E+00	3-hydroxyethyl bacteriochlorophyllide a dehydrogenase [EC:1.-.-.-]
KO:K11381	0.85	0.85	1.57	2.41	0.00	11.60	0.00	5.69E-01	5.69E-01	3.64E-01	2.20E-01	1.00E+00	1.37E-02	1.00E+00	2-oxoisovalerate dehydrogenase E1 component [EC:1.2.4.4]
KO:K11383	0.12	0.00	0.00	0.00	0.00	0.00	0.00	6.26E-02	1.00E+00	1.00E+00	1.00E+00	1.00E+00	1.00E+00	1.00E+00	two-component system, NtrC family, sensor histidine kinase KinB
KO:K11384	0.09	0.00	0.00	0.00	0.28	0.00	0.12	1.92E-01	1.00E+00	1.00E+00	1.00E+00	9.31E-02	1.00E+00	1.68E-01	two-component system, NtrC family, response regulator AlgB
KO:K11391	0.12	0.00	0.00	0.00	0.00	0.00	0.00	6.16E-02	1.00E+00	1.00E+00	1.00E+00	1.00E+00	1.00E+00	1.00E+00	ribosomal RNA large subunit methyltransferase G [EC:2.1.1.174]
KO:K11434	0.02	0.00	0.53	0.00	0.00	0.00	0.00	2.35E-01	1.00E+00	3.10E-02	1.00E+00	1.00E+00	1.00E+00	1.00E+00	protein arginine N-methyltransferase 1 [EC:2.1.1.-]
KO:K11438	0.00	0.11	0.00	0.00	0.00	0.00	0.00	1.00E+00	1.02E-01	1.00E+00	1.00E+00	1.00E+00	1.00E+00	1.00E+00	protein arginine N-methyltransferase 7 [EC:2.1.1.-]
KO:K11443	0.01	0.00	0.06	3.33	0.00	0.00	0.23	6.29E-01	1.00E+00	5.32E-01	2.99E-02	1.00E+00	1.00E+00	3.67E-01	two-component system, cell cycle response regulator DivK
KO:K11444	0.12	0.11	0.00	0.00	0.00	0.00	0.00	1.40E-01	1.60E-01	1.00E+00	1.00E+00	1.00E+00	1.00E+00	1.00E+00	two-component system, chemotaxis family, response regulator WspR
KO:K11472	1.08	9.60	0.55	2.21	0.39	5.19	2.92	8.66E-01	2.46E-02	9.28E-01	6.28E-01	9.40E-01	1.28E-01	4.47E-01	glycolate oxidase FAD binding subunit
KO:K11473	1.48	10.38	0.14	2.16	6.01	1.29	3.35	8.41E-01	2.79E-02	9.75E-01	7.19E-01	1.17E-01	8.66E-01	4.43E-01	glycolate oxidase iron-sulfur subunit
KO:K11474	0.12	0.00	0.00	0.00	0.00	0.00	0.00	6.26E-02	1.00E+00	1.00E+00	1.00E+00	1.00E+00	1.00E+00	1.00E+00	GntR family transcriptional regulator, glc operon transcriptional
KO:K11475	0.00	2.19	0.00	0.04	0.00	0.00	0.00	1.00E+00	3.96E-02	1.00E+00	2.63E-01	1.00E+00	1.00E+00	1.00E+00	GntR family transcriptional regulator, vanillate catabolism
KO:K11517	0.11	0.00	0.00	0.02	0.81	0.00	0.00	1.92E-01	1.00E+00	1.00E+00	3.10E-01	5.23E-02	1.00E+00	1.00E+00	(S)-2-hydroxy-acid oxidase [EC:1.1.3.15]

KO:K11527	0.11	0.00	0.02	1.28	0.00	0.00	4.28	5.14E-01	1.00E+00	6.08E-01	1.58E-01	1.00E+00	1.00E+00	4.21E-02	two-component system, unclassified family, sensor histidine kinase
KO:K11528	0.00	0.00	3.56	1.20	0.00	0.00	0.00	1.00E+00	1.00E+00	5.17E-02	1.21E-01	1.00E+00	1.00E+00	1.00E+00	UDP-N-acetylglucosamine pyrophosphorylase [EC:2.7.7.23]
KO:K11530	0.00	0.11	0.00	0.00	0.00	0.00	0.00	1.00E+00	1.04E-01	1.00E+00	1.00E+00	1.00E+00	1.00E+00	1.00E+00	autoinducer 2-degrading protein
KO:K11531	0.00	1.44	0.00	0.00	0.00	0.00	0.00	1.00E+00	5.54E-02	1.00E+00	1.00E+00	1.00E+00	1.00E+00	1.00E+00	lsr operon transcriptional repressor
KO:K11532	0.00	0.00	0.00	0.00	37.50	0.00	0.00	1.00E+00	1.00E+00	1.00E+00	1.00E+00	2.86E-04	1.00E+00	1.00E+00	fructose-1,6-bisphosphatase II / sedoheptulose-1,7-bisphosphatase
KO:K11537	0.47	0.00	0.00	0.00	0.00	0.00	0.00	6.51E-02	1.00E+00	1.00E+00	1.00E+00	1.00E+00	1.00E+00	1.00E+00	MFS transporter, NHS family, xanthosine permease
KO:K11600	0.00	0.00	0.00	0.00	0.00	0.00	7.08	1.00E+00	1.00E+00	1.00E+00	1.00E+00	1.00E+00	1.00E+00	6.86E-03	exosome complex component RRP41
KO:K11604	0.00	0.04	0.00	0.13	0.00	0.00	0.00	1.00E+00	1.99E-01	1.00E+00	1.36E-01	1.00E+00	1.00E+00	1.00E+00	manganese/iron transport system substrate-binding protein
KO:K11605	0.03	2.38	0.00	0.75	0.00	0.00	0.00	7.12E-01	4.01E-02	1.00E+00	1.74E-01	1.00E+00	1.00E+00	1.00E+00	manganese/iron transport system permease protein
KO:K11606	0.12	3.55	0.00	0.02	0.00	0.00	0.00	4.76E-01	2.66E-02	1.00E+00	5.79E-01	1.00E+00	1.00E+00	1.00E+00	manganese/iron transport system permease protein
KO:K11607	0.31	2.46	0.00	0.60	0.00	0.00	0.00	4.24E-01	5.03E-02	1.00E+00	2.83E-01	1.00E+00	1.00E+00	1.00E+00	manganese/iron transport system ATP-binding protein
KO:K11615	0.00	0.11	0.00	0.00	0.00	0.00	0.00	1.00E+00	9.81E-02	1.00E+00	1.00E+00	1.00E+00	1.00E+00	1.00E+00	two-component system, CitB family, response regulator MalR
KO:K11618	0.00	0.00	9.38	0.00	0.00	0.00	0.00	1.00E+00	1.00E+00	1.76E-02	1.00E+00	1.00E+00	1.00E+00	1.00E+00	two-component system, NarL family, response regulator LiaR
KO:K11646	0.00	0.00	0.00	0.00	0.00	0.00	3.75	1.00E+00	1.00E+00	1.00E+00	1.00E+00	1.00E+00	1.00E+00	4.57E-03	dehydroquinate synthase II [EC:1.4.1.-]
KO:K11688	0.96	32.00	0.00	2.65	0.00	0.00	0.00	7.85E-01	1.43E-03	1.00E+00	4.82E-01	1.00E+00	1.00E+00	1.00E+00	C4-dicarboxylate-binding protein DctP
KO:K11689	0.44	27.33	0.00	0.78	0.00	0.00	0.00	7.78E-01	3.43E-03	1.00E+00	7.04E-01	1.00E+00	1.00E+00	1.00E+00	C4-dicarboxylate transporter, DctQ subunit
KO:K11690	1.38	29.94	0.00	2.18	0.00	0.00	0.00	7.12E-01	7.14E-04	1.00E+00	5.53E-01	1.00E+00	1.00E+00	1.00E+00	C4-dicarboxylate transporter, DctM subunit
KO:K11707	0.00	7.63	8.78	0.42	0.00	0.00	0.29	8.60E-01	2.84E-02	1.99E-02	7.23E-01	1.00E+00	1.00E+00	7.65E-01	manganese/zinc/iron transport system substrate-binding protein
KO:K11708	0.00	1.50	9.98	0.37	0.00	0.00	0.28	8.64E-01	1.67E-01	8.43E-03	5.26E-01	1.00E+00	1.00E+00	5.87E-01	manganese/zinc/iron transport system permease protein
KO:K11709	0.00	2.30	8.48	0.74	0.00	0.00	0.25	1.00E+00	1.18E-01	9.43E-03	4.29E-01	1.00E+00	1.00E+00	6.79E-01	manganese/zinc/iron transport system permease protein
KO:K11710	0.00	1.46	13.72	0.15	0.01	0.00	0.64	8.35E-01	1.89E-01	4.00E-03	7.07E-01	8.26E-01	1.00E+00	4.31E-01	manganese/zinc/iron transport system ATP- binding protein
KO:K11711	0.12	0.00	0.00	0.00	0.00	0.00	0.00	6.47E-02	1.00E+00	1.00E+00	1.00E+00	1.00E+00	1.00E+00	1.00E+00	two-component system, LuxR family, sensor histidine kinase DctS
KO:K11712	0.00	0.00	0.00	0.10	0.00	0.00	0.00	1.00E+00	1.00E+00	1.00E+00	1.43E-01	1.00E+00	1.00E+00	1.00E+00	two-component system, LuxR family, response regulator DctR
KO:K11717	3.27	8.52	4.37	3.45	4.91	6.58	2.10	7.91E-01	6.04E-02	5.37E-01	7.58E-01	4.02E-01	1.52E-01	9.27E-01	cysteine desulfurase / selenocysteine lyase [EC:2.8.1.7 4.4.1.16]
KO:K11719	0.91	1.15	0.05	0.30	0.00	0.00	0.00	1.27E-01	8.90E-02	7.22E-01	3.61E-01	1.00E+00	1.00E+00	1.00E+00	lipopolysaccharide export system protein LptC
KO:K11720	8.23	4.29	0.18	2.92	0.00	0.00	0.15	2.94E-02	1.20E-01	9.36E-01	2.56E-01	1.00E+00	1.00E+00	9.39E-01	lipopolysaccharide export system permease protein
KO:K11731	0.06	0.00	0.07	0.61	0.00	0.00	1.13	4.15E-01	1.00E+00	4.03E-01	1.39E-01	1.00E+00	1.00E+00	8.11E-02	citronellyl-CoA dehydrogenase [EC:1.3.99.-]
KO:K11741	0.00	0.00	0.00	0.00	0.00	0.00	0.42	1.00E+00	1.00E+00	1.00E+00	1.00E+00	1.00E+00	1.00E+00	2.77E-02	quaternary ammonium compound-resistance protein SugE
KO:K11744	0.58	0.00	0.00	0.00	0.00	0.00	0.00	6.36E-02	1.00E+00	1.00E+00	1.00E+00	1.00E+00	1.00E+00	1.00E+00	AI-2 transport protein TqsA
KO:K11745	0.27	0.33	0.00	0.21	0.00	0.00	0.00	2.15E-01	1.90E-01	1.00E+00	2.51E-01	1.00E+00	1.00E+00	1.00E+00	glutathione-regulated potassium-efflux system ancillary protein KefC
KO:K11747	0.17	0.00	0.30	0.03	0.00	0.00	0.00	1.41E-01	1.00E+00	8.66E-02	3.02E-01	1.00E+00	1.00E+00	1.00E+00	glutathione-regulated potassium-efflux system protein KefB
KO:K11749	11.43	10.48	3.32	8.55	0.52	11.83	0.88	6.94E-02	9.61E-02	9.28E-01	2.18E-01	9.93E-01	5.99E-02	9.90E-01	regulator of sigma E protease [EC:3.4.24.-]
KO:K11751	0.00	0.11	0.00	0.00	0.00	0.00	0.00	1.00E+00	1.02E-01	1.00E+00	1.00E+00	1.00E+00	1.00E+00	1.00E+00	5'-nucleotidase / UDP-sugar diphosphatase [EC:3.1.3.5 3.6.1.45]
KO:K11752	4.72	6.36	5.59	2.14	5.80	2.88	1.26	2.85E-01	9.90E-02	1.57E-01	8.96E-01	1.37E-01	7.73E-01	9.62E-01	diaminohydroxyphosphoribosylaminopyrimidine deaminase /
KO:K11753	6.49	6.63	5.83	3.31	4.44	1.97	1.89	1.55E-01	1.44E-01	2.29E-01	7.69E-01	4.98E-01	9.26E-01	9.30E-01	riboflavin kinase / FMN adenylyltransferase [EC:2.7.1.26 2.7.7.2]
KO:K11754	6.23	5.28	0.12	2.65	0.40	0.04	1.72	4.30E-02	7.03E-02	9.81E-01	3.93E-01	9.56E-01	9.91E-01	7.03E-01	dihydrofolate synthase / folylpolyglutamate synthase [EC:6.3.2.12
KO:K11755	0.63	0.03	0.00	0.14	12.99	0.09	0.09	3.20E-01	7.90E-01	1.00E+00	6.80E-01	3.00E-03	7.36E-01	7.36E-01	phosphoribosyl-ATP pyrophosphohydrolase / phosphoribosyl-AMP
KO:K11777	0.00	0.00	0.00	0.00	12.40	0.00	0.00	1.00E+00	1.00E+00	1.00E+00	5.04E-01	2.86E-04	1.00E+00	1.00E+00	HAD superfamily phosphatase
KO:K11779	4.65	0.06	0.00	0.45	0.00	0.00	0.22	6.43E-02	5.45E-01	1.00E+00	3.40E-01	1.00E+00	1.00E+00	4.58E-01	FO synthase [EC:2.5.1.-]
KO:K11780	0.00	0.00	0.00	0.00	1.50	0.00	0.67	1.00E+00	1.00E+00	1.00E+00	1.00E+00	2.10E-02	1.00E+00	7.17E-02	FO synthase subunit 1 [EC:2.5.1.-]
KO:K11781	0.00	0.00	0.00	0.00	5.21	0.00	0.21	1.00E+00	1.00E+00	1.00E+00	1.00E+00	1.00E-02	1.00E+00	1.67E-01	FO synthase subunit 2 [EC:2.5.1.-]
KO:K11782	0.00	0.00	3.75	0.00	0.00	0.00	0.00	1.00E+00	1.00E+00	1.67E-02	1.00E+00	1.00E+00	1.00E+00	1.00E+00	chorismate futalosine-lyase [EC:4.1.99.-]
KO:K11787	0.52	0.95	0.00	0.44	0.00	0.00	1.39	3.33E-01	2.31E-01	1.00E+00	3.56E-01	1.00E+00	1.00E+00	1.70E-01	phosphoribosylamine--glycine ligase / phosphoribosylglycinamide
KO:K11788	0.35	0.00	0.00	0.00	0.00	0.00	0.00	6.17E-02	1.00E+00	1.00E+00	1.00E+00	1.00E+00	1.00E+00	1.00E+00	phosphoribosylamine--glycine ligase /
KO:K11811	0.07	2.54	0.54	0.01	0.00	0.00	0.45	6.90E-01	3.34E-02	2.60E-01	8.11E-01	1.00E+00	1.00E+00	3.06E-01	arsenical resistance protein ArsH
KO:K11890	0.23	0.00	0.00	0.00	0.00	0.00	0.00	5.74E-02	1.00E+00	1.00E+00	1.00E+00	1.00E+00	1.00E+00	1.00E+00	type VI secretion system protein ImpM
KO:K11891	0.18	0.11	0.00	0.14	0.00	0.00	0.26	2.50E-01	3.24E-01	1.00E+00	2.96E-01	1.00E+00	1.00E+00	1.96E-01	type VI secretion system protein ImpL
KO:K11892	0.05	0.10	2.14	0.02	0.00	0.00	0.00	4.34E-01	3.42E-01	1.33E-02	5.12E-01	1.00E+00	1.00E+00	1.00E+00	type VI secretion system protein ImpK
KO:K11893	0.10	0.16	2.96	0.05	0.74	0.00	0.01	5.27E-01	4.57E-01	1.96E-02	6.25E-01	1.46E-01	1.00E+00	7.23E-01	type VI secretion system protein ImpJ
KO:K11894	0.47	0.00	0.00	0.00	0.00	0.00	0.00	3.50E-02	1.00E+00	1.00E+00	1.00E+00	1.00E+00	1.00E+00	1.00E+00	type VI secretion system protein ImpI
KO:K11895	0.06	0.00	0.00	0.10	0.00	0.00	0.18	2.39E-01	1.00E+00	1.00E+00	1.96E-01	1.00E+00	1.00E+00	1.33E-01	type VI secretion system protein ImpH
KO:K11896	0.32	0.03	0.58	0.04	0.06	0.00	0.26	2.09E-01	6.27E-01	1.02E-01	5.99E-01	5.38E-01	1.00E+00	2.57E-01	type VI secretion system protein ImpG
KO:K11897	0.06	0.06	0.00	0.00	0.00	0.00	0.20	2.27E-01	2.35E-01	1.00E+00	1.00E+00	1.00E+00	1.00E+00	1.21E-01	type VI secretion system protein ImpF
KO:K11898	0.45	0.01	0.00	0.00	0.65	0.00	0.00	1.14E-01	3.84E-01	1.00E+00	1.00E+00	8.74E-02	1.00E+00	1.00E+00	type VI secretion system protein ImpE
KO:K11899	0.11	0.54	0.00	0.00	0.00	0.00	0.00	2.15E-01	9.64E-02	1.00E+00	1.00E+00	1.00E+00	1.00E+00	1.00E+00	type VI secretion system protein ImpD
KO:K11900	0.88	0.27	0.12	0.55	5.43	0.00	0.05	2.83E-01	5.15E-01	6.26E-01	3.74E-01	4.01E-02	1.00E+00	7.26E-01	type VI secretion system protein ImpC
KO:K11901	1.74	0.06	0.00	0.05	3.07	0.00	0.00	1.05E-01	5.19E-01	1.00E+00	5.28E-01	5.63E-02	1.00E+00	1.00E+00	type VI secretion system protein ImpB
KO:K11902	0.11	0.18	0.00	0.16	0.82	0.00	0.00	3.29E-01	2.67E-01	1.00E+00	2.81E-01	8.53E-02	1.00E+00	1.00E+00	type VI secretion system protein ImpA
KO:K11903	1.29	0.08	0.00	0.34	5.69	0.00	0.00	1.78E-01	6.33E-01	1.00E+00	4.38E-01	2.53E-02	1.00E+00	1.00E+00	type VI secretion system secreted protein Hcp
KO:K11904	0.00	0.06	0.00	0.05	0.00	0.00	0.00	1.00E+00	1.46E-01	1.00E+00	1.85E-01	1.00E+00	1.00E+00	1.00E+00	type VI secretion system secreted protein VgrG
KO:K11906	0.06	0.05	0.00	0.00	0.00	0.00	0.00	1.21E-01	1.66E-01	1.00E+00	1.00E+00	1.00E+00	1.00E+00	1.00E+00	type VI secretion system protein VasD
KO:K11907	0.07	0.20	4.25	0.02	0.63	0.00	0.04	5.67E-01	3.97E-01	1.81E-02	6.66E-01	1.89E-01	1.00E+00	6.20E-01	type VI secretion system protein VasG
KO:K11908	0.15	0.01	0.00	0.00	0.00	2.54	0.00	1.91E-01	3.15E-01	1.00E+00	1.00E+00	1.00E+00	2.34E-02	1.00E+00	sigma-54 specific transcriptional regulator
KO:K11912	0.38	0.01	0.00	0.05	0.00	0.00	0.00	9.31E-02	3.67E-01	1.00E+00	2.93E-01	1.00E+00	1.00E+00	1.00E+00	serine/threonine-protein kinase PpkA [EC:2.7.11.1]
KO:K11915	0.10	0.20	0.00	0.00	0.00	0.00	0.00	1.77E-01	1.28E-01	1.00E+00	1.00E+00	1.00E+00	1.00E+00	1.00E+00	serine/threonine protein phosphatase Stp1 [EC:3.1.3.16]
KO:K11919	0.23	0.00	0.00	0.00	0.00	0.00	0.00	5.97E-02	1.00E+00	1.00E+00	1.00E+00	1.00E+00	1.00E+00	1.00E+00	type VI secretion system lysozyme-related protein
KO:K11921	0.12	0.00	0.00	0.00	0.00	0.00	0.00	6.51E-02	1.00E+00	1.00E+00	1.00E+00	1.00E+00	1.00E+00	1.00E+00	LysR family transcriptional regulator
KO:K11923	0.12	0.00	0.00	0.00	0.00	0.00	0.00	6.40E-02	1.00E+00	1.00E+00	1.00E+00	1.00E+00	1.00E+00	1.00E+00	MerR family transcriptional regulator, copper efflux regulator
KO:K11924	1.12	0.32	0.18	0.86	0.00	0.00	0.00	1.51E-01	3.74E-01	4.65E-01	1.98E-01	1.00E+00	1.00E+00	1.00E+00	DtxR family transcriptional regulator, manganese transport regulator
KO:K11927	5.12	15.92	7.57	1.89	3.09	6.84	1.77	5.49E-01	1.03E-02	1.88E-01	9.49E-01	8.64E-01	2.60E-01	9.53E-01	ATP-dependent RNA helicase RhlE [EC:3.6.4.13]
KO:K11928	2.05	0.01	0.29	0.30	0.98	9.75	0.01	1.51E-01	8.21E-01	5.97E-01	5.94E-01	3.20E-01	1.37E-02	8.17E-01	sodium/proline symporter
KO:K11930	0.06	0.05	0.00	0.00	0.00	0.00	0.00	1.23E-01	1.66E-01	1.00E+00	1.00E+00	1.00E+00	1.00E+00	1.00E+00	periplasmic protein TorT
KO:K11936	0.12	0.00	0.00	0.00	0.00	0.00	0.00	6.40E-02	1.00E+00	1.00E+00	1.00E+00	1.00E+00	1.00E+00	1.00E+00	biofilm PGA synthesis N-glycosyltransferase PgaC [EC:2.4.-.-]
KO:K11940	0.72	9.38	2.31	3.67	0.00	0.00	0.00	7.57E-01	2.41E-02	3.50E-01	1.70E-01	1.00E+00	1.00E+00	9.50E-01	heat shock protein HspQ
KO:K11941	0.12	0.00	0.00	0.00	0.00	0.00	0.00	5.96E-02	1.00E+00	1.00E+00	1.00E+00	1.00E+00	1.00E+00	1.00E+00	glucans biosynthesis protein C [EC:2.1.-.-]
KO:K11942	2.42	1.02	0.00	2.45	0.00	10.54	0.11	2.60E-01	5.50E-01	1.00E+00	2.57E-01	1.00E+00	2.10E-02	8.09E-01	methylmalonyl-CoA mutase [EC:5.4.99.2]
KO:K11945	0.01	0.00	0.00	0.00	0.00	1.56	0.00	2.49E-01	1.00E+00	1.00E+00	1.00E+00	1.00E+00	5.57E-03	1.00E+00	extradiol dioxygenase [EC:1.13.11.-]
KO:K11956	0.23	1.72	0.00	0.02	0.00	0.00	0.00	2.71E-01	8.31E-02	1.00E+00	3.89E-01	1.00E+00	1.00E+00	1.00E+00	neutral amino acid transport system permease protein
KO:K11957	0.00	0.00	0.00	2.04	0.00	0.00	0.00	1.00E+00	1.00E+00	1.00E+00	1.43E-01	1.00E+00	1.00E+00	1.00E+00	neutral amino acid transport system ATP-binding protein
KO:K11958	0.00	1.44	0.00	0.00	0.00	0.00	0.00	1.00E+00	1.01E-01	1.00E+00	1.00E+00	1.00E+00	1.00E+00	1.00E+00	neutral amino acid transport system ATP-binding protein
KO:K11959	0.00	0.00	0.00	0.00	41.25	0.00	0.00	1.00E+00	1.00E+00	1.00E+00	1.00E+00	0.00E+00	1.00E+00	1.00E+00	urea transport system substrate-binding protein
KO:K11960	0.00	0.00	0.00	0.00	17.50	0.00	0.00	1.00E+00	1.00E+00	1.00E+00	1.00E+00	1.43E-04	1.00E+00	1.00E+00	urea transport system permease protein
KO:K11961	0.00	0.00	0.00	0.00	27.50	0.00	0.00	1.00E+00	1.00E+00	1.00E+00	1.00E+00	0.00E+00	1.00E+00	1.00E+00	urea transport system permease protein
KO:K11962	0.00	0.00	0.00	0.00	15.52	0.00	0.00	1.00E+00	1.00E+00	1.00E+00	5.03E-01	0.00E+00	1.00E+00	1.00E+00	urea transport system ATP-binding protein
KO:K11963	0.00	0.00	0.00	0.00	15.00	0.00	0.00	1.00E+00	1.00E+00	1.00E+00	1.00E+00	1.43E-04	1.00E+00	1.00E+00	urea transport system ATP-binding protein
KO:K11991	0.62	0.00	0.72	0.48	0.00	0.00	1.84	2.35E-01	1.00E+00	2.05E-01	2.89E-01	1.00E+00	1.00E+00	6.86E-02	tRNA-specific adenosine deaminase [EC:3.5.4.-]
KO:K11996	0.85	0.09	0.04	0.23	13.67	0.00	0.08	3.20E-01	7.48E-01	7.82E-01	6.46E-01	1.00E-03	1.00E+00	7.57E-01	adenylyltransferase and sulfurtransferase MOCS3/UBA4
KO:K12063	0.00	0.00	0.00	0.00	0.00	0.00	0.42	1.00E+00	1.00E+00	1.00E+00	1.00E+00	1.00E+00	1.00E+00	2.51E-02	conjugal transfer ATP-binding protein TraC
KO:K12065	0.00	0.00	0.00	0.20	0.00	0.00	0.00	1.00E+00	1.00E+00	1.00E+00	1.43E-01	1.00E+00	1.00E+00	1.00E+00	conjugal transfer pilus assembly protein TraB
KO:K12073	0.00	0.00	1.94	0.00	4.10	0.00	2.31	1.00E+00	1.00E+00	9.11E-02	1.00E+00	3.69E-02	1.00E+00	7.40E-02	1,4-dihydroxy-2-naphthoyl-CoA thioesterase [EC:3.1.2.-]
KO:K12150	0.00	0.00	0.17	0.00	0.00	0.00	1.21	1.00E+00	1.00E+00	1.61E-01	1.00E+00	1.00E+00	1.00E+00	2.91E-02	HIT-like protein HinT
KO:K12152	0.58	0.00	0.00	0.00	0.00	0.00	0.00	6.40E-02	1.00E+00	1.00E+00	1.00E+00	1.00E+00	1.00E+00	1.00E+00	phosphatase NudJ [EC:3.6.1.-]
KO:K12202	0.00	0.00	0.00	0.00	0.00	10.00	0.00	1.00E+00	1.00E+00	1.00E+00	1.00E+00	1.00E+00	2.14E-03	1.00E+00	defect in organelle trafficking protein DotA
KO:K12203	0.23	0.00	0.00	0.00	0.00	0.00	0.00	5.79E-02	1.00E+00	1.00E+00	1.00E+00	1.00E+00	1.00E+00	1.00E+00	defect in organelle trafficking protein DotB
KO:K12205	0.23	0.00	0.00	0.00	0.00	0.00	0.00	6.26E-02	1.00E+00	1.00E+00	1.00E+00	1.00E+00	1.00E+00	1.00E+00	defect in organelle trafficking protein DotD
KO:K12214	0.12	0.00	0.00	0.00	0.00	0.00	0.00	6.50E-02	1.00E+00	1.00E+00	1.00E+00	1.00E+00	1.00E+00	1.00E+00	intracellular multiplication protein IcmL

KO:K12217	0.47	0.00	0.00	0.00	0.00	0.00	0.00	3.91E-02	1.00E+00	1.00E+00	1.00E+00	1.00E+00	1.00E+00	1.00E+00	intracellular multiplication protein IcmO
KO:K12223	0.00	0.00	0.00	0.00	0.00	3.33	0.00	1.00E+00	1.00E+00	1.00E+00	1.00E+00	1.00E+00	2.14E-03	1.00E+00	intracellular multiplication protein IcmV
KO:K12234	3.17	0.46	0.00	0.25	0.00	0.00	0.92	9.83E-02	3.84E-01	1.00E+00	4.79E-01	1.00E+00	1.00E+00	2.77E-01	F42-:gamma-glutamyl ligase [EC:6.3.2.-]
KO:K12251	2.97	0.05	2.16	0.44	0.05	0.00	1.45	7.76E-02	8.29E-01	1.19E-01	5.53E-01	8.27E-01	1.00E+00	2.03E-01	N-carbamoylputrescine amidase [EC:3.5.1.53]
KO:K12252	0.15	11.69	0.00	0.00	0.00	0.00	0.00	6.96E-01	2.14E-03	1.00E+00	8.50E-01	1.00E+00	1.00E+00	1.00E+00	arginine:pyruvate transaminase [EC:2.6.1.84]
KO:K12253	0.01	7.67	0.00	0.00	0.00	0.00	0.00	6.33E-01	1.19E-02	1.00E+00	1.00E+00	1.00E+00	1.00E+00	1.00E+00	5-guanidino-2-oxopentanoate decarboxylase [EC:4.1.1.75]
KO:K12254	0.19	0.12	0.00	0.00	0.00	0.00	0.62	1.85E-01	2.40E-01	1.00E+00	1.00E+00	1.00E+00	1.00E+00	9.69E-02	4-guanidinobutyraldehyde dehydrogenase / NAD-dependent aldehyde
KO:K12255	0.52	0.80	0.00	0.00	0.00	0.00	0.00	1.36E-01	9.63E-02	1.00E+00	1.00E+00	1.00E+00	1.00E+00	1.00E+00	guanidinobutyrase [EC:3.5.3.7]
KO:K12256	5.20	14.22	0.18	0.85	0.00	0.00	0.00	1.18E-01	1.21E-02	8.92E-01	7.60E-01	1.00E+00	1.00E+00	1.00E+00	putrescine aminotransferase [EC:2.6.1.-]
KO:K12257	2.51	1.02	12.17	3.23	1.95	6.38	0.44	5.27E-01	7.91E-01	2.87E-02	4.10E-01	6.27E-01	1.32E-01	8.70E-01	SecD/SecF fusion protein
KO:K12262	0.31	1.48	0.19	3.09	0.00	0.00	0.08	6.11E-01	1.82E-01	7.00E-01	6.43E-02	1.00E+00	1.00E+00	7.98E-01	cytochrome b561
KO:K12266	0.43	0.02	0.00	0.01	0.00	0.00	0.00	6.01E-02	3.77E-01	1.00E+00	3.88E-01	1.00E+00	1.00E+00	1.00E+00	anaerobic nitric oxide reductase transcription regulator
KO:K12267	1.10	0.01	0.82	1.03	1.71	0.65	0.94	3.14E-01	7.60E-01	3.87E-01	3.30E-01	2.12E-01	4.47E-01	3.50E-01	peptide methionine sulfoxide reductase msrA/msrB [EC:1.8.4.11
KO:K12276	3.39	0.19	0.00	0.39	0.00	0.00	0.14	5.01E-02	5.16E-01	1.00E+00	3.89E-01	1.00E+00	1.00E+00	5.62E-01	MSHA biogenesis protein MshE
KO:K12278	0.79	0.18	0.00	0.50	0.00	0.00	0.75	1.90E-01	4.26E-01	1.00E+00	2.70E-01	1.00E+00	1.00E+00	1.98E-01	MSHA biogenesis protein MshG
KO:K12279	3.48	0.03	0.00	0.08	0.00	0.00	0.20	4.60E-02	5.82E-01	1.00E+00	4.91E-01	1.00E+00	1.00E+00	4.06E-01	MSHA biogenesis protein MshI
KO:K12280	0.07	0.02	0.00	0.00	0.00	0.00	0.23	1.83E-01	3.19E-01	1.00E+00	1.00E+00	1.00E+00	1.00E+00	1.01E-01	MSHA biogenesis protein MshJ
KO:K12282	2.23	0.06	0.00	0.22	0.00	0.00	0.35	5.06E-02	6.03E-01	1.00E+00	4.43E-01	1.00E+00	1.00E+00	3.58E-01	MSHA biogenesis protein MshL
KO:K12283	1.14	0.17	0.61	0.02	0.00	0.00	0.00	8.19E-02	3.72E-01	1.50E-01	5.97E-01	1.00E+00	1.00E+00	1.00E+00	MSHA biogenesis protein MshM
KO:K12284	0.70	0.00	0.00	0.00	0.00	0.00	0.00	3.07E-02	1.00E+00	1.00E+00	1.00E+00	1.00E+00	1.00E+00	1.00E+00	MSHA biogenesis protein MshN
KO:K12285	0.16	0.01	0.00	0.02	0.00	0.00	0.73	1.92E-01	5.02E-01	1.00E+00	4.36E-01	1.00E+00	1.00E+00	5.37E-02	MSHA biogenesis protein MshO
KO:K12286	0.38	0.00	0.00	0.02	0.00	0.00	0.00	3.79E-02	1.00E+00	1.00E+00	3.33E-01	1.00E+00	1.00E+00	1.00E+00	MSHA biogenesis protein MshP
KO:K12287	4.22	0.00	0.00	0.30	0.00	0.00	0.00	3.63E-02	1.00E+00	1.00E+00	3.49E-01	1.00E+00	1.00E+00	1.00E+00	MSHA biogenesis protein MshQ
KO:K12297	7.63	0.18	0.33	0.35	0.00	3.16	0.20	1.91E-02	8.24E-01	7.58E-01	7.46E-01	1.00E+00	1.03E-01	8.17E-01	ribosomal RNA large subunit methyltransferase L [EC:2.1.1.173]
KO:K12308	0.70	3.60	0.00	1.41	3.10	0.00	0.00	5.33E-01	8.57E-02	1.00E+00	3.18E-01	1.05E-01	1.00E+00	1.00E+00	beta-galactosidase [EC:3.2.1.23]
KO:K12339	8.69	0.22	0.60	1.75	0.00	1.06	2.39	2.04E-02	8.79E-01	7.99E-01	4.47E-01	1.00E+00	6.59E-01	2.99E-01	cysteine synthase B [EC:2.5.1.47]
KO:K12340	11.92	14.46	0.76	7.25	0.00	6.20	0.19	4.30E-02	2.11E-02	9.80E-01	2.47E-01	9.98E-01	3.83E-01	9.91E-01	outer membrane channel protein TolC
KO:K12343	2.91	0.00	0.00	0.00	0.00	0.00	0.00	5.94E-02	1.00E+00	1.00E+00	1.00E+00	1.00E+00	1.00E+00	1.00E+00	3-oxo-5-alpha-steroid 4-dehydrogenase 1 [EC:1.3.99.5]
KO:K12368	0.03	4.56	0.00	3.54	0.00	0.00	0.00	6.76E-01	7.83E-02	1.00E+00	1.14E-01	1.00E+00	1.00E+00	1.00E+00	dipeptide transport system substrate-binding protein
KO:K12369	0.01	7.59	0.00	0.04	0.00	0.00	0.00	5.76E-01	2.14E-02	1.00E+00	5.18E-01	1.00E+00	1.00E+00	1.00E+00	dipeptide transport system permease protein
KO:K12370	0.16	6.15	0.00	0.12	0.00	0.00	0.00	4.98E-01	2.99E-02	1.00E+00	5.29E-01	1.00E+00	1.00E+00	1.00E+00	dipeptide transport system permease protein
KO:K12371	0.00	3.54	0.00	0.04	0.00	0.00	0.00	5.19E-01	4.31E-02	1.00E+00	4.33E-01	1.00E+00	1.00E+00	1.00E+00	dipeptide transport system ATP-binding protein
KO:K12372	0.00	4.31	0.00	0.03	0.00	0.00	0.00	4.63E-01	4.93E-02	1.00E+00	3.99E-01	1.00E+00	1.00E+00	1.00E+00	dipeptide transport system ATP-binding protein
KO:K12373	2.53	0.69	0.08	1.88	0.11	0.00	0.00	1.08E-01	4.19E-01	7.68E-01	1.61E-01	7.39E-01	1.00E+00	1.00E+00	beta-hexosaminidase [EC:3.2.1.52]
KO:K12410	1.85	0.03	1.44	1.54	0.01	0.00	1.25	1.41E-01	8.83E-01	1.90E-01	1.73E-01	9.04E-01	1.00E+00	2.27E-01	NAD-dependent deacetylase [EC:3.5.1.-]
KO:K12420	0.51	0.70	0.00	0.33	0.00	0.00	0.00	1.90E-01	1.41E-01	1.00E+00	2.66E-01	1.00E+00	1.00E+00	1.00E+00	ketoacyl reductase [EC:1.3.1.-]
KO:K12429	0.00	0.00	0.00	0.20	0.00	0.00	0.00	1.00E+00	1.00E+00	1.00E+00	1.43E-01	1.00E+00	1.00E+00	1.00E+00	fatty acid CoA ligase FadD36
KO:K12447	0.00	0.00	0.00	0.00	0.00	0.00	0.83	1.00E+00	1.00E+00	1.00E+00	1.00E+00	1.00E+00	1.00E+00	2.73E-02	UDP-sugar pyrophosphorylase [EC:2.7.7.64]
KO:K12452	1.27	0.54	2.99	2.26	1.26	0.00	0.56	4.01E-01	6.42E-01	1.63E-01	2.30E-01	4.03E-01	1.00E+00	6.35E-01	CDP-6-deoxy-D-xylo-4-hexulose-3-dehydrase
KO:K12454	0.28	0.00	8.04	0.13	0.00	0.00	0.00	2.99E-01	5.95E-01	1.14E-02	4.00E-01	1.00E+00	1.00E+00	1.00E+00	CDP-paratose 2-epimerase [EC:5.1.3.1]
KO:K12500	1.82	0.02	0.00	0.00	0.00	0.00	0.06	6.46E-02	3.33E-01	1.00E+00	1.00E+00	1.00E+00	1.00E+00	2.96E-01	thioesterase III [EC:3.1.2.-]
KO:K12506	0.48	10.99	0.00	2.74	0.00	0.00	0.01	7.67E-01	1.09E-02	1.00E+00	1.59E-01	1.00E+00	1.00E+00	9.48E-01	2-C-methyl-D-erythritol 4-phosphate cytidylyltransferase /
KO:K12507	0.00	0.89	0.00	0.00	0.00	0.00	0.00	1.00E+00	1.04E-01	1.00E+00	1.00E+00	1.00E+00	1.00E+00	1.00E+00	acyl-CoA synthetase [EC:6.2.1.-]
KO:K12508	0.00	2.86	0.00	3.48	0.00	0.00	0.00	7.77E-01	8.64E-02	1.00E+00	6.61E-02	1.00E+00	1.00E+00	1.00E+00	feruloyl-CoA synthase [EC:6.2.1.34]
KO:K12510	0.01	0.01	3.73	0.32	1.86	0.00	0.19	7.53E-01	7.46E-01	1.99E-02	3.60E-01	5.77E-02	1.00E+00	4.73E-01	tight adherence protein B
KO:K12511	0.01	0.07	7.26	0.48	0.39	0.00	0.11	8.62E-01	7.27E-01	5.00E-03	3.08E-01	3.60E-01	1.00E+00	6.54E-01	tight adherence protein C
KO:K12524	1.75	0.84	0.00	1.14	0.00	6.40	0.00	2.06E-01	4.16E-01	1.00E+00	3.21E-01	1.00E+00	3.27E-02	1.00E+00	bifunctional aspartokinase/homoserine dehydrogenase 1 [EC:2.7.2.4
KO:K12526	0.43	0.00	0.00	0.33	0.00	0.00	0.00	1.25E-01	1.00E+00	1.00E+00	1.78E-01	1.00E+00	1.00E+00	1.00E+00	bifunctional diaminopimelate decarboxylase/aspartate kinase
KO:K12535	0.22	0.00	0.00	0.27	0.00	0.00	0.00	1.56E-01	1.00E+00	1.00E+00	1.46E-01	1.00E+00	1.00E+00	1.00E+00	outer membrane protein RsaF
KO:K12536	0.04	0.00	0.00	4.51	0.00	0.00	0.00	4.33E-01	1.00E+00	1.00E+00	4.46E-02	1.00E+00	1.00E+00	1.00E+00	ATP-binding cassette, subfamily C, bacterial HasD
KO:K12541	0.12	0.00	0.00	0.00	0.00	0.00	0.00	6.39E-02	1.00E+00	1.00E+00	1.00E+00	1.00E+00	1.00E+00	1.00E+00	ATP-binding cassette, subfamily C, bacterial LapB
KO:K12542	0.16	2.56	0.00	0.02	0.00	0.00	0.00	3.03E-01	6.40E-02	1.00E+00	4.06E-01	1.00E+00	1.00E+00	1.00E+00	membrane fusion protein LapC
KO:K12543	0.22	0.00	0.00	0.28	0.00	0.00	4.76	3.04E-01	1.00E+00	1.00E+00	2.79E-01	1.00E+00	1.00E+00	4.84E-02	outer membrane protein LapE
KO:K12544	0.00	2.11	0.00	0.00	0.00	0.00	0.00	1.00E+00	1.02E-01	1.00E+00	1.00E+00	1.00E+00	1.00E+00	1.00E+00	S-layer protein
KO:K12573	9.03	6.98	5.82	2.12	6.63	2.67	2.01	6.19E-02	1.59E-01	3.00E-01	9.33E-01	1.90E-01	8.91E-01	9.40E-01	ribonuclease R [EC:3.1.-.-]
KO:K12574	0.00	0.00	0.00	0.00	41.88	0.00	0.00	1.00E+00	1.00E+00	1.00E+00	1.00E+00	0.00E+00	1.00E+00	1.00E+00	ribonuclease J [EC:3.1.-.-]
KO:K12582	0.04	0.15	0.00	0.00	0.00	0.00	0.00	1.81E-01	1.25E-01	1.00E+00	1.00E+00	1.00E+00	1.00E+00	1.00E+00	TDP-Fuc4NAc transferase [EC:2.4.1.-]
KO:K12589	0.00	0.00	0.00	0.00	0.00	0.00	6.67	1.00E+00	1.00E+00	1.00E+00	1.00E+00	1.00E+00	1.00E+00	1.96E-02	exosome complex component RRP42
KO:K12658	0.02	0.33	0.04	1.90	0.65	0.00	0.00	7.22E-01	3.53E-01	6.69E-01	7.11E-02	2.17E-01	1.00E+00	1.00E+00	4-hydroxyproline epimerase [EC:5.1.1.8]
KO:K12659	0.00	0.00	0.00	0.31	0.00	0.00	0.00	1.00E+00	1.00E+00	1.00E+00	1.43E-01	1.00E+00	1.00E+00	1.00E+00	N-acetyl-gamma-glutamyl-phosphate reductase / acetylglutamate kinase
KO:K12660	2.02	0.00	0.00	0.02	0.00	0.00	0.00	4.46E-02	1.00E+00	1.00E+00	2.88E-01	1.00E+00	1.00E+00	1.00E+00	2-dehydro-3-deoxy-L-rhamnonate aldolase [EC:4.1.2.-]
KO:K12661	0.00	0.00	0.00	1.63	0.00	0.00	0.00	1.00E+00	1.00E+00	1.00E+00	7.76E-02	1.00E+00	1.00E+00	1.00E+00	L-rhamnonate dehydratase [EC:4.2.1.9]
KO:K12686	0.04	0.00	0.00	0.13	0.00	0.00	0.00	1.80E-01	1.00E+00	1.00E+00	1.45E-01	1.00E+00	1.00E+00	1.00E+00	outer membrane lipase/esterase
KO:K12700	0.00	0.00	2.50	0.00	0.00	0.00	0.00	1.00E+00	1.00E+00	1.51E-02	1.00E+00	1.00E+00	1.00E+00	1.00E+00	non-specific ribonclеoside hydrolase [EC:3.2.-.-]
KO:K12858	0.00	0.00	0.00	0.00	0.00	0.00	1.25	1.00E+00	1.00E+00	1.00E+00	1.00E+00	1.00E+00	1.00E+00	2.47E-02	ATP-dependent RNA helicase DDX23/PRP28 [EC:3.6.4.13]
KO:K12941	0.62	2.50	0.00	1.59	0.00	0.00	0.00	3.47E-01	1.48E-01	1.00E+00	2.09E-01	1.00E+00	1.00E+00	1.00E+00	aminobenzoyl-glutamate utilization protein B
KO:K12942	1.48	0.00	0.05	1.76	0.00	0.00	0.00	1.33E-01	1.00E+00	4.74E-01	1.18E-01	1.00E+00	1.00E+00	1.00E+00	aminobenzoyl-glutamate transport protein
KO:K12957	0.06	0.00	0.45	0.07	0.45	0.00	0.00	3.54E-01	1.00E+00	1.02E-01	3.29E-01	1.02E-01	1.00E+00	1.00E+00	uncharacterized zinc-type alcohol dehydrogenase-like protein
KO:K12961	0.02	0.00	0.53	0.00	0.00	0.00	0.00	2.34E-01	1.00E+00	2.84E-02	1.00E+00	1.00E+00	1.00E+00	1.00E+00	DnaA initiator-associating protein
KO:K12972	0.59	8.98	0.00	0.34	0.00	0.00	0.00	4.81E-01	1.56E-02	1.00E+00	6.29E-01	1.00E+00	1.00E+00	1.00E+00	gyoxylate/hydroxypyruvate reductase A [EC:1.1.1.79 1.1.1.81]
KO:K12979	1.89	0.00	0.00	0.16	0.00	0.00	0.00	6.61E-02	1.00E+00	1.00E+00	2.25E-01	1.00E+00	1.00E+00	1.00E+00	beta-hydroxylase [EC:1.14.11.-]
KO:K12980	0.00	1.11	0.00	0.00	0.00	0.00	0.00	1.00E+00	6.87E-02	1.00E+00	1.00E+00	1.00E+00	1.00E+00	1.00E+00	lipid A oxidase
KO:K12982	0.10	0.00	3.02	0.00	0.00	0.00	0.00	2.82E-01	4.91E-01	8.29E-03	4.97E-01	1.00E+00	1.00E+00	1.00E+00	heptosyltransferase I [EC:2.4.-.-]
KO:K12984	0.00	0.00	0.00	0.01	0.00	0.00	1.57	1.00E+00	1.00E+00	1.00E+00	2.41E-01	1.00E+00	1.00E+00	2.34E-02	(heptosyl)LPS beta-1,4-glucosyltransferase [EC:2.4.1.-]
KO:K12989	0.80	0.01	0.00	0.03	0.00	0.00	0.00	6.10E-02	3.66E-01	1.00E+00	3.18E-01	1.00E+00	1.00E+00	1.00E+00	mannosyltransferase [EC:2.4.1.-]
KO:K12990	1.83	0.00	0.00	0.04	0.00	0.00	0.00	5.19E-02	1.00E+00	1.00E+00	2.36E-01	1.00E+00	1.00E+00	1.00E+00	rhamnosyltransferase [EC:2.4.1.-]
KO:K12992	0.00	0.11	0.00	0.00	0.00	0.00	0.00	1.00E+00	9.74E-02	1.00E+00	1.00E+00	1.00E+00	1.00E+00	1.00E+00	rhamnosyltransferase [EC:2.4.1.-]
KO:K12994	0.00	0.11	0.00	0.00	0.00	0.00	0.00	1.00E+00	9.84E-02	1.00E+00	1.00E+00	1.00E+00	1.00E+00	1.00E+00	alpha-1,3-rhamnosyltransferase [EC:2.4.1.-]
KO:K12995	0.00	0.00	0.00	0.41	0.00	0.00	0.00	1.00E+00	1.00E+00	1.00E+00	1.43E-01	1.00E+00	1.00E+00	1.00E+00	rhamnosyltransferase [EC:2.4.1.-]
KO:K12997	0.23	0.00	0.00	0.00	0.00	0.00	0.00	6.80E-02	1.00E+00	1.00E+00	1.00E+00	1.00E+00	1.00E+00	1.00E+00	rhamnosyltransferase [EC:2.4.1.-]
KO:K13002	0.10	0.64	0.00	0.00	0.00	0.00	0.00	1.88E-01	1.20E-01	1.00E+00	1.00E+00	1.00E+00	1.00E+00	1.00E+00	glycosyltransferase [EC:2.4.1.-]
KO:K13006	0.03	0.00	0.93	0.02	0.00	0.00	0.00	2.92E-01	1.00E+00	2.70E-02	3.23E-01	1.00E+00	1.00E+00	1.00E+00	UDP-perosamine 4-acetyltransferase [EC:2.3.1.-]
KO:K13007	4.41	0.00	0.00	0.11	0.00	0.00	0.00	4.40E-02	1.00E+00	1.00E+00	3.70E-01	1.00E+00	1.00E+00	1.00E+00	Fuc2NAc and GlcNAc transferase [EC:2.4.1.-]
KO:K13010	0.00	0.36	0.00	0.02	0.00	0.00	0.00	1.00E+00	1.01E-01	1.00E+00	2.17E-01	1.00E+00	1.00E+00	1.00E+00	perosamine synthetase
KO:K13012	0.22	0.00	0.00	0.00	0.00	0.00	2.21	2.12E-01	1.00E+00	1.00E+00	1.00E+00	1.00E+00	1.00E+00	4.73E-02	O-antigen biosynthesis protein WbqP
KO:K13013	0.00	0.00	0.00	3.16	0.00	0.00	0.00	1.00E+00	1.00E+00	1.00E+00	3.87E-02	1.00E+00	1.00E+00	1.00E+00	O-antigen biosynthesis protein WbqV
KO:K13015	0.23	0.44	0.00	0.00	4.96	0.00	0.00	3.10E-01	2.34E-01	1.00E+00	1.00E+00	3.03E-02	1.00E+00	1.00E+00	UDP-N-acetyl-D-glucosamine dehydrogenase [EC:1.1.1.-]
KO:K13016	0.61	0.27	0.00	0.00	2.81	0.00	0.00	1.98E-01	2.65E-01	1.00E+00	1.00E+00	6.56E-02	1.00E+00	1.00E+00	UDP-D-GlcNAcA oxidase [EC:1.1.1.-]
KO:K13017	0.00	0.25	0.00	0.70	0.00	0.00	0.00	1.00E+00	1.98E-01	1.00E+00	1.15E-01	1.00E+00	1.00E+00	1.00E+00	UDP-3-keto-D-GlcNAcA aminotransferase [EC:2.6.1.-]
KO:K13018	0.02	0.57	0.00	0.00	0.00	0.00	0.00	1.86E-01	1.04E-01	1.00E+00	1.00E+00	1.00E+00	1.00E+00	1.00E+00	UDP-D-GlcNAc3NA acetyltransferase [EC:2.3.1.-]
KO:K13019	0.00	0.35	0.00	0.11	2.34	0.00	0.00	1.00E+00	1.92E-01	1.00E+00	2.97E-01	5.47E-02	1.00E+00	1.00E+00	UDP-GlcNAc3NAcA epimerase [EC:5.1.3.23]
KO:K13020	0.00	0.00	0.00	1.43	0.00	0.00	0.00	1.00E+00	1.00E+00	1.00E+00	5.43E-02	1.00E+00	1.00E+00	1.00E+00	UDP-D-GlcNAcA oxidase [EC:1.1.1.-]
KO:K13038	3.72	7.86	4.82	3.38	2.43	0.02	0.09	3.48E-01	5.90E-02	1.99E-01	4.16E-01	6.42E-01	9.78E-01	9.72E-01	phosphopantothenoylcysteine decarboxylase /
KO:K13039	0.70	3.23	0.00	0.00	0.00	0.00	0.02	2.22E-01	7.40E-02	1.00E+00	1.00E+00	1.00E+00	1.00E+00	5.21E-01	sulfopyruvate decarboxylase subunit beta [EC:4.1.1.79]
KO:K13042	0.70	0.00	0.00	0.00	0.00	0.00	0.00	3.69E-02	1.00E+00	1.00E+00	1.00E+00	1.00E+00	1.00E+00	1.00E+00	dimethylglycine N-methyltransferase [EC:2.1.1.161]
KO:K13043	0.33	0.61	0.00	0.01	0.00	0.00	0.00	1.69E-01	1.01E-01	1.00E+00	5.13E-01	1.00E+00	1.00E+00	1.00E+00	N-succinyl-L-ornithine transcarbamylase [EC:2.1.3.11]

KO:K13049	0.09	0.04	0.30	0.01	0.00	0.00	0.00	2.11E-01	3.18E-01	8.30E-02	4.18E-01	1.00E+00	1.00E+00	1.00E+00	carboxypeptidase PM2D1 [EC:3.4.17.-]
KO:K13051	0.83	0.00	3.05	0.33	0.12	0.00	0.00	1.86E-01	1.00E+00	4.67E-02	3.42E-01	4.70E-01	1.00E+00	1.00E+00	beta-aspartyl-peptidase (threonine type) [EC:3.4.19.5]
KO:K13053	0.34	0.00	0.00	0.00	0.00	6.81	0.00	1.73E-01	1.00E+00	1.00E+00	1.00E+00	1.00E+00	1.56E-02	1.00E+00	cell division inhibitor SulA
KO:K13057	0.00	0.00	0.00	0.10	0.00	0.00	0.00	1.00E+00	1.00E+00	1.00E+00	1.43E-01	1.00E+00	1.00E+00	1.00E+00	trehalose synthase [EC:2.4.1.245]
KO:K13058	0.00	0.00	0.00	0.20	0.00	0.00	0.00	1.00E+00	1.00E+00	1.00E+00	1.43E-01	1.00E+00	1.00E+00	1.00E+00	mannosylfructose-phosphate synthase [EC:2.4.1.246]
KO:K13075	0.00	0.11	0.00	0.00	0.00	0.00	0.00	1.00E+00	1.01E-01	1.00E+00	1.00E+00	1.00E+00	1.00E+00	1.00E+00	N-acyl homoserine lactone hydrolase [EC:3.1.1.81]
KO:K13208	0.23	0.00	0.00	0.00	0.00	0.00	0.00	6.04E-02	1.00E+00	1.00E+00	1.00E+00	1.00E+00	1.00E+00	1.00E+00	ELAV like protein 2/3/4
KO:K13237	0.12	0.00	0.00	0.10	0.00	0.00	0.00	1.02E-01	1.00E+00	1.00E+00	2.14E-01	1.00E+00	1.00E+00	1.00E+00	peroxisomal 2,4-dienoyl-CoA reductase [EC:1.3.1.34]
KO:K13238	0.47	0.00	0.00	0.00	0.00	0.00	0.00	5.93E-02	1.00E+00	1.00E+00	1.00E+00	1.00E+00	1.00E+00	1.00E+00	3,2-trans-enoyl-CoA isomerase, mitochondrial [EC:5.3.3.8]
KO:K13256	2.18	0.00	1.06	3.07	0.00	0.00	0.00	1.70E-01	1.00E+00	2.90E-01	1.24E-01	1.00E+00	1.00E+00	1.00E+00	protein PsiE
KO:K13280	0.00	0.00	0.00	0.00	0.00	0.00	5.00	1.00E+00	1.00E+00	1.00E+00	1.00E+00	1.00E+00	1.00E+00	5.86E-03	signal peptidase, endoplasmic reticulum-type [EC:3.4.-.-]
KO:K13282	0.37	0.12	0.00	0.00	0.00	0.00	0.00	1.02E-01	2.00E-01	1.00E+00	1.00E+00	1.00E+00	1.00E+00	1.00E+00	cyanophycinase [EC:3.4.15.6]
KO:K13283	0.11	0.00	1.41	0.34	0.00	0.00	0.00	3.28E-01	1.00E+00	5.24E-02	1.92E-01	1.00E+00	1.00E+00	1.00E+00	ferrous-iron efflux pump FieF
KO:K13288	1.29	0.01	6.76	0.79	0.00	0.00	0.01	1.83E-01	7.99E-01	1.43E-02	2.97E-01	1.00E+00	1.00E+00	8.02E-01	oligoribonuclease [EC:3.1.-.-]
KO:K13292	2.25	5.37	2.41	2.37	6.17	0.00	0.44	5.25E-01	1.21E-01	4.92E-01	4.99E-01	9.07E-02	1.00E+00	8.96E-01	phosphatidylglycerol:prolipoprotein diacylglycerol transferase
KO:K13378	5.86	0.13	0.00	0.33	0.00	0.00	0.00	3.79E-02	4.93E-01	1.00E+00	3.91E-01	1.00E+00	1.00E+00	1.00E+00	NADH dehydrogenase I subunit C/D [EC:1.6.5.3]
KO:K13403	0.00	0.00	0.00	1.22	0.00	0.00	0.00	1.00E+00	1.00E+00	1.00E+00	1.43E-01	1.00E+00	1.00E+00	1.00E+00	methylenetetrahydrofolate dehydrogenase(NAD+) /
KO:K13421	0.00	0.00	0.00	0.00	6.25	0.00	0.00	1.00E+00	1.00E+00	1.00E+00	1.00E+00	3.43E-03	1.00E+00	1.00E+00	uridine monophosphate synthetase [EC:2.4.2.1 4.1.1.23]
KO:K13444	0.00	0.10	4.33	0.00	0.00	0.00	0.00	1.00E+00	2.45E-01	1.50E-02	1.00E+00	1.00E+00	1.00E+00	1.00E+00	sulfatase modifying factor 1
KO:K13472	0.07	0.00	0.92	0.01	0.00	0.00	0.00	2.25E-01	1.00E+00	3.04E-02	3.94E-01	1.00E+00	1.00E+00	1.00E+00	sulfotransferase
KO:K13481	2.50	15.52	0.00	0.10	0.00	0.00	0.00	2.22E-01	5.86E-03	1.00E+00	8.46E-01	1.00E+00	1.00E+00	1.00E+00	xanthine dehydrogenase small subunit [EC:1.17.1.4]
KO:K13482	1.36	14.51	0.05	0.25	0.00	0.00	0.00	4.17E-01	5.86E-03	8.34E-01	7.81E-01	1.00E+00	1.00E+00	1.00E+00	xanthine dehydrogenase large subunit [EC:1.17.1.4]
KO:K13483	0.08	0.00	2.43	0.02	0.00	0.00	0.00	2.74E-01	1.00E+00	1.47E-02	3.38E-01	1.00E+00	1.00E+00	1.00E+00	xanthine dehydrogenase YagT iron-sulfur-binding subunit
KO:K13487	0.23	0.00	0.00	0.00	0.00	0.00	0.00	6.04E-02	1.00E+00	1.00E+00	1.00E+00	1.00E+00	1.00E+00	1.00E+00	methyl-accepting chemotaxis protein WspA
KO:K13500	0.00	0.00	1.25	0.00	0.00	0.00	0.00	1.00E+00	1.00E+00	1.61E-02	1.00E+00	1.00E+00	1.00E+00	1.00E+00	chondroitin synthase [EC:2.4.1.175 2.4.1.226]
KO:K13503	0.00	0.00	0.00	1.84	0.00	0.00	0.00	1.00E+00	1.00E+00	1.00E+00	5.49E-02	1.00E+00	1.00E+00	1.00E+00	anthranilate synthase [EC:4.1.3.27]
KO:K13525	0.00	0.00	0.00	0.01	0.00	0.00	13.89	1.00E+00	1.00E+00	1.00E+00	3.36E-01	1.00E+00	1.00E+00	5.43E-03	transitional endoplasmic reticulum ATPase
KO:K13529	0.13	0.08	0.41	0.00	0.00	0.00	0.41	2.82E-01	3.46E-01	1.39E-01	5.33E-01	1.00E+00	1.00E+00	1.39E-01	AraC family transcriptional regulator, regulatory protein of
KO:K13541	0.21	0.25	0.00	0.08	7.80	0.00	0.00	4.54E-01	4.27E-01	1.00E+00	6.29E-01	2.29E-03	1.00E+00	1.00E+00	cobalamin biosynthesis protein CbiG / precorrin-3B
KO:K13542	0.00	0.00	20.05	0.00	0.37	0.00	0.01	1.00E+00	1.00E+00	3.00E-03	1.00E+00	2.54E-01	1.00E+00	4.69E-01	uroporphyrinogen III methyltransferase / synthase [EC:2.1.1.17
KO:K13566	0.00	0.00	0.00	0.00	1.88	0.00	0.00	1.00E+00	1.00E+00	1.00E+00	1.00E+00	1.53E-02	1.00E+00	1.00E+00	omega-amidase [EC:3.5.1.3]
KO:K13574	0.04	0.22	0.00	1.32	2.49	0.00	0.00	4.35E-01	3.50E-01	1.00E+00	1.64E-01	1.04E-01	1.00E+00	1.00E+00	uncharacterized oxidoreductase [EC:1.1.1.-]
KO:K13581	0.40	9.55	0.00	4.75	0.00	0.00	0.02	7.61E-01	2.91E-02	1.00E+00	1.04E-01	1.00E+00	1.00E+00	8.61E-01	modification methylase [EC:2.1.1.72]
KO:K13582	0.00	0.00	0.00	1.12	0.00	0.00	0.00	1.00E+00	1.00E+00	1.00E+00	3.09E-02	1.00E+00	1.00E+00	1.00E+00	localization factor PodJL
KO:K13583	0.58	7.65	0.00	3.29	0.00	0.00	0.00	6.15E-01	3.83E-02	1.00E+00	1.38E-01	1.00E+00	1.00E+00	1.00E+00	GcrA cell cycle regulator
KO:K13584	0.38	3.23	0.00	1.83	0.00	0.00	0.00	5.19E-01	6.11E-02	1.00E+00	1.28E-01	1.00E+00	1.00E+00	1.00E+00	two-component system, cell cycle response regulator CtrA
KO:K13586	0.04	0.00	0.00	0.13	0.00	0.00	0.00	1.79E-01	1.00E+00	1.00E+00	1.45E-01	1.00E+00	1.00E+00	1.00E+00	holdfast attachment protein HfaB
KO:K13587	0.00	0.39	0.00	1.32	0.00	0.00	0.00	1.00E+00	1.74E-01	1.00E+00	3.91E-02	1.00E+00	1.00E+00	1.00E+00	two-component system, cell cycle sensor histidine kinase and
KO:K13588	0.00	0.02	0.00	0.52	0.00	0.00	0.00	1.00E+00	2.40E-01	1.00E+00	6.86E-02	1.00E+00	1.00E+00	1.00E+00	histidine phosphotransferase ChpT
KO:K13590	1.11	0.00	0.00	0.67	0.00	0.00	0.00	5.74E-02	1.00E+00	1.00E+00	1.07E-01	1.00E+00	1.00E+00	1.00E+00	diguanylate cyclase
KO:K13592	0.04	0.00	0.00	0.13	0.00	0.00	0.00	1.57E-01	1.00E+00	1.00E+00	1.32E-01	1.00E+00	1.00E+00	1.00E+00	regulator of CtrA degradation
KO:K13593	0.00	0.00	0.00	0.20	0.00	0.00	0.00	1.00E+00	1.00E+00	1.00E+00	7.73E-02	1.00E+00	1.00E+00	1.00E+00	cyclic-di-GMP phosphodiesterase, flagellum assembly factor TipF
KO:K13598	0.51	13.99	0.00	1.95	0.00	0.00	0.00	7.28E-01	6.86E-03	1.00E+00	3.23E-01	1.00E+00	1.00E+00	1.00E+00	two-component system, NtrC family, nitrogen regulation sensor
KO:K13599	0.75	8.26	0.00	4.08	0.00	0.00	0.00	6.58E-01	2.40E-02	1.00E+00	9.09E-02	1.00E+00	1.00E+00	1.00E+00	two-component system, NtrC family, nitrogen regulation response
KO:K13606	0.12	0.00	0.00	0.00	0.00	0.00	0.00	6.46E-02	1.00E+00	1.00E+00	1.00E+00	1.00E+00	1.00E+00	1.00E+00	chlorophyll(ide) b reductase [EC:1.1.1.294]
KO:K13622	0.00	0.00	0.18	0.39	0.18	0.00	0.00	1.00E+00	1.00E+00	1.68E-01	1.14E-01	1.68E-01	1.00E+00	1.00E+00	S-adenosylmethionine-diacylglycerol 3-amino-3-carboxypropyl
KO:K13623	0.01	0.00	0.00	0.00	1.77	0.00	0.00	2.45E-01	1.00E+00	1.00E+00	1.00E+00	1.17E-02	1.00E+00	1.00E+00	S-adenosylmethionine-diacylgycerolhomoserine-N-methlytransferase
KO:K13626	0.00	0.00	21.64	0.00	0.00	0.00	0.00	3.40E-01	1.00E+00	6.14E-03	1.00E+00	1.00E+00	1.00E+00	1.00E+00	flagellar assembly factor FliW
KO:K13628	0.21	0.54	16.78	1.76	0.36	2.11	5.92	8.61E-01	7.99E-01	7.86E-03	4.82E-01	8.35E-01	4.05E-01	8.51E-02	iron-sulfur cluster assembly protein
KO:K13634	4.44	0.19	4.05	0.25	0.00	0.72	0.00	6.86E-02	6.93E-01	7.66E-02	6.64E-01	1.00E+00	4.61E-01	1.00E+00	LysR family transcriptional regulator, cys regulon transcriptional
KO:K13635	0.12	0.00	0.00	0.00	0.00	0.00	0.00	6.79E-02	1.00E+00	1.00E+00	1.00E+00	1.00E+00	1.00E+00	1.00E+00	LysR family transcriptional regulator, cys regulon transcriptional
KO:K13636	0.00	0.00	0.63	0.00	0.00	0.00	0.00	1.00E+00	1.00E+00	1.53E-02	1.00E+00	1.00E+00	1.00E+00	1.00E+00	LysR family transcriptional regulator, D-serine deaminase activator
KO:K13637	0.00	1.52	0.00	0.04	0.00	0.00	0.00	1.00E+00	7.01E-02	1.00E+00	3.05E-01	1.00E+00	1.00E+00	1.00E+00	GntR family transcriptional regulator, uxuAB operon transcriptional
KO:K13638	0.00	0.00	0.54	0.01	0.00	0.00	0.00	1.00E+00	1.00E+00	1.20E-02	2.52E-01	1.00E+00	1.00E+00	1.00E+00	MerR family transcriptional regulator, Zn(II)-responsive regulator
KO:K13639	0.00	0.61	0.00	0.13	0.00	0.00	0.00	1.00E+00	6.49E-02	1.00E+00	2.36E-01	1.00E+00	1.00E+00	1.00E+00	MerR family transcriptional regulator, redox-sensitive
KO:K13641	0.00	1.08	1.05	0.33	0.09	11.45	0.00	8.02E-01	2.54E-01	2.61E-01	5.00E-01	6.79E-01	8.00E-03	1.00E+00	IclR family transcriptional regulator, acetate operon repressor
KO:K13643	4.35	2.36	1.69	3.34	0.00	5.60	1.80	2.08E-01	5.00E-01	6.53E-01	3.24E-01	1.00E+00	1.26E-01	6.28E-01	Rrf2 family transcriptional regulator, iron-sulfur cluster assembly
KO:K13652	0.26	0.03	0.00	0.00	0.00	0.00	0.00	7.04E-02	2.56E-01	1.00E+00	1.00E+00	1.00E+00	1.00E+00	1.00E+00	AraC family transcriptional regulator
KO:K13657	0.12	0.00	0.00	0.00	0.00	0.00	0.00	6.69E-02	1.00E+00	1.00E+00	1.00E+00	1.00E+00	1.00E+00	1.00E+00	alpha-1,3-mannosyltransferase [EC:2.4.1.-]
KO:K13659	0.00	0.00	0.00	0.92	0.00	0.00	0.00	1.00E+00	1.00E+00	1.00E+00	6.17E-02	1.00E+00	1.00E+00	1.00E+00	glucuronosyltransferase [EC:2.4.1.-]
KO:K13665	0.12	0.00	0.00	0.00	0.00	0.00	0.00	6.00E-02	1.00E+00	1.00E+00	1.00E+00	1.00E+00	1.00E+00	1.00E+00	pyruvyltransferase
KO:K13668	0.00	0.00	7.92	0.09	0.00	0.00	0.00	1.00E+00	1.00E+00	7.57E-03	3.23E-01	1.00E+00	1.00E+00	1.00E+00	phosphatidylinositol alpha-1,6-mannosyltransferase [EC:2.4.1.-]
KO:K13670	0.47	0.00	0.00	0.00	0.00	0.00	0.00	6.14E-02	1.00E+00	1.00E+00	1.00E+00	1.00E+00	1.00E+00	1.00E+00	alpha-1,6-mannosyltransferase [EC:2.4.1.-]
KO:K13685	0.00	0.00	3.77	0.40	0.00	0.00	0.00	1.00E+00	1.00E+00	2.90E-02	1.70E-01	1.00E+00	1.00E+00	1.00E+00	UDP-N-acetylglucosamine:undecaprenyl-P N-acetylglucosaminyl 1-P
KO:K13688	0.02	0.02	0.00	0.35	0.00	0.00	0.00	2.70E-01	2.89E-01	1.00E+00	8.11E-02	1.00E+00	1.00E+00	1.00E+00	cyclic beta-1,2-glucan synthetase [EC:2.4.1.-]
KO:K13693	0.00	0.00	11.25	0.00	0.00	0.00	0.00	1.00E+00	1.00E+00	1.66E-02	1.00E+00	1.00E+00	1.00E+00	1.00E+00	glucosyl-3-phosphoglycerate synthase [EC:2.4.1.-]
KO:K13694	0.00	0.00	0.00	0.10	0.00	0.00	0.00	1.00E+00	1.00E+00	1.00E+00	1.43E-01	1.00E+00	1.00E+00	1.00E+00	lipoprotein Spr
KO:K13695	0.00	0.00	0.63	0.00	0.00	0.00	0.00	1.00E+00	1.00E+00	1.69E-02	1.00E+00	1.00E+00	1.00E+00	1.00E+00	probable lipoprotein NlpC
KO:K13722	0.00	0.00	0.00	0.00	0.00	0.00	3.33	1.00E+00	1.00E+00	1.00E+00	1.00E+00	1.00E+00	1.00E+00	2.99E-02	tricorn protease interacting factor F2/3 [EC:3.4.11.-]
KO:K13730	0.00	0.00	0.00	0.31	0.00	0.00	0.00	1.00E+00	1.00E+00	1.00E+00	1.43E-01	1.00E+00	1.00E+00	1.00E+00	internalin A
KO:K13735	0.00	0.00	0.00	0.82	0.00	0.00	0.00	1.00E+00	1.00E+00	1.00E+00	1.43E-01	1.00E+00	1.00E+00	1.00E+00	adhesin/invasin
KO:K13745	0.00	0.87	0.00	0.07	0.00	0.00	0.00	1.00E+00	7.53E-02	1.00E+00	2.80E-01	1.00E+00	1.00E+00	1.00E+00	L-2,4-diaminobutyrate decarboxylase [EC:4.1.1.86]
KO:K13747	0.10	2.97	0.31	0.58	0.00	0.00	0.00	6.23E-01	5.71E-02	4.60E-01	3.29E-01	1.00E+00	1.00E+00	1.00E+00	carboxynorspermidine decarboxylase [EC:4.1.1.-]
KO:K13766	0.23	4.39	0.03	1.28	0.00	0.00	0.00	6.18E-01	4.36E-02	7.98E-01	2.08E-01	1.00E+00	1.00E+00	1.00E+00	methylglutaconyl-CoA hydratase [EC:4.2.1.18]
KO:K13770	0.47	0.00	0.00	0.00	0.00	0.00	0.00	6.34E-02	1.00E+00	1.00E+00	1.00E+00	1.00E+00	1.00E+00	1.00E+00	TetR/AcrR family transcriptional regulator, fatty acid metabolism
KO:K13771	0.02	0.40	0.00	0.01	0.00	0.00	0.00	3.91E-01	4.63E-02	1.00E+00	4.07E-01	1.00E+00	1.00E+00	1.00E+00	Rrf2 family transcriptional regulator, nitric oxide-sensitive
KO:K13774	0.12	0.00	0.00	0.10	0.00	0.00	0.00	1.26E-01	1.00E+00	1.00E+00	1.80E-01	1.00E+00	1.00E+00	1.00E+00	citronellol/citronellal dehydrogenase
KO:K13775	1.81	0.00	0.04	1.54	0.00	0.26	0.00	1.33E-01	1.00E+00	5.52E-01	1.52E-01	1.00E+00	4.03E-01	1.00E+00	citronellol/citronellal dehydrogenase
KO:K13776	0.96	0.00	2.27	0.00	0.00	0.00	0.00	1.50E-01	1.00E+00	6.99E-02	1.00E+00	1.00E+00	1.00E+00	1.00E+00	citronellyl-CoA synthetase [EC:6.2.1.-]
KO:K13777	1.95	0.21	1.40	0.02	0.00	0.00	0.00	8.39E-02	4.23E-01	1.22E-01	5.84E-01	1.00E+00	1.00E+00	1.00E+00	geranyl-CoA carboxylase alpha subunit [EC:6.4.1.5]
KO:K13778	3.13	0.05	0.00	0.00	0.00	0.00	0.00	5.11E-02	3.53E-01	1.00E+00	4.42E-01	1.00E+00	1.00E+00	1.00E+00	geranyl-CoA carboxylase beta subunit [EC:6.4.1.5]
KO:K13779	1.22	0.00	0.00	0.03	0.00	0.00	0.00	4.01E-02	1.00E+00	1.00E+00	2.75E-01	1.00E+00	1.00E+00	1.00E+00	isohexenylglutaconyl-CoA hydratase [EC:4.2.1.57]
KO:K13787	0.00	0.00	0.00	0.00	0.00	0.00	3.75	1.00E+00	1.00E+00	1.00E+00	1.00E+00	1.00E+00	1.00E+00	1.50E-02	geranylgeranyl diphosphate synthase, type I [EC:2.5.1.1 2.5.1.1
KO:K13788	0.23	0.00	0.00	0.00	0.00	0.00	0.00	5.90E-02	1.00E+00	1.00E+00	1.00E+00	1.00E+00	1.00E+00	1.00E+00	phosphate acetyltransferase [EC:2.3.1.8]
KO:K13789	0.81	0.61	0.94	0.62	5.60	0.00	0.00	4.01E-01	4.78E-01	3.60E-01	4.74E-01	3.54E-02	1.00E+00	1.00E+00	geranylgeranyl diphosphate synthase, type II [EC:2.5.1.1 2.5.1.1
KO:K13795	0.00	0.01	0.00	1.83	0.00	0.00	0.00	1.00E+00	2.89E-01	1.00E+00	6.36E-02	1.00E+00	1.00E+00	1.00E+00	citrate/tricarballylate utilization protein
KO:K13796	0.00	0.08	0.00	2.37	0.00	0.00	0.00	1.00E+00	2.95E-01	1.00E+00	4.61E-02	1.00E+00	1.00E+00	1.00E+00	tricarballylate dehydrogenase
KO:K13798	0.00	0.00	0.00	0.00	0.00	0.00	5.00	1.00E+00	1.00E+00	1.00E+00	1.00E+00	1.00E+00	1.00E+00	2.66E-02	DNA-directed RNA polymerase subunit B [EC:2.7.7.6]
KO:K13799	0.00	0.00	0.00	0.00	18.75	0.00	0.00	1.00E+00	1.00E+00	1.00E+00	1.00E+00	4.29E-04	1.00E+00	1.00E+00	pantoate ligase / cytidylate kinase [EC:6.3.2.1 2.7.4.14]
KO:K13815	0.00	0.00	0.00	0.20	0.00	0.00	0.00	1.00E+00	1.00E+00	1.00E+00	1.43E-01	1.00E+00	1.00E+00	1.00E+00	two-component system, response regulator RpfG
KO:K13816	0.04	0.00	0.00	0.13	0.00	0.00	0.00	1.75E-01	1.00E+00	1.00E+00	1.45E-01	1.00E+00	1.00E+00	1.00E+00	DSF synthase
KO:K13819	0.00	0.00	0.00	0.00	0.00	0.00	0.42	1.00E+00	1.00E+00	1.00E+00	1.00E+00	1.00E+00	1.00E+00	2.81E-02	NifU-like protein
KO:K13821	2.07	1.12	0.03	0.54	0.23	0.00	0.00	1.28E-01	2.34E-01	7.07E-01	4.14E-01	5.61E-01	1.00E+00	1.00E+00	proline dehydrogenase / delta 1-pyrroline-5-carboxylate
KO:K13829	0.00	0.00	0.00	1.02	0.00	0.00	0.00	1.00E+00	1.00E+00	1.00E+00	5.73E-02	1.00E+00	1.00E+00	1.00E+00	shikimate kinase / 3-dehydroquinate synthase [EC:2.7.1.71 4.2.3.4]
KO:K13831	0.23	0.00	0.00	0.00	0.00	0.00	0.00	6.30E-02	1.00E+00	1.00E+00	1.00E+00	1.00E+00	1.00E+00	1.00E+00	3-hexulose-6-phosphate synthase / 6-phospho-3-hexuloisomerase
KO:K13832	0.00	0.00	0.00	0.00	2.50	0.00	0.00	1.00E+00	1.00E+00	1.00E+00	1.00E+00	1.60E-02	1.00E+00	1.00E+00	3-dehydroquinate dehydratase / shikimate dehydrogenase [EC:4.2.1.1

KO:K13888	2.02	0.00	0.00	0.02	0.00	0.00	0.00	4.19E-02	1.00E+00	1.00E+00	2.47E-01	1.00E+00	1.00E+00	1.00E+00	macrolide-specific efflux protein MacA
KO:K13889	0.00	0.11	0.00	0.00	0.00	0.00	0.00	1.00E+00	1.04E-01	1.00E+00	1.00E+00	1.00E+00	1.00E+00	1.00E+00	glutathione transport system substrate-binding protein
KO:K13890	0.00	0.22	0.00	0.00	0.00	0.00	0.00	1.00E+00	9.96E-02	1.00E+00	1.00E+00	1.00E+00	1.00E+00	1.00E+00	glutathione transport system permease protein
KO:K13892	0.00	1.89	0.00	0.00	0.00	0.00	0.00	1.00E+00	1.02E-01	1.00E+00	1.00E+00	1.00E+00	1.00E+00	1.00E+00	glutathione transport system ATP-binding protein
KO:K13893	0.86	0.58	0.00	0.99	0.00	9.87	0.00	3.62E-01	4.48E-01	1.00E+00	3.31E-01	1.00E+00	2.13E-02	1.00E+00	microcin C transport system substrate-binding protein
KO:K13894	1.14	0.79	0.00	0.44	0.00	0.00	0.00	1.54E-01	2.08E-01	1.00E+00	2.92E-01	1.00E+00	1.00E+00	1.00E+00	microcin C transport system permease protein
KO:K13895	0.00	0.19	0.00	1.61	0.00	0.00	0.00	3.95E-01	2.67E-01	1.00E+00	8.60E-02	1.00E+00	1.00E+00	1.00E+00	microcin C transport system permease protein
KO:K13896	0.38	1.03	0.00	0.20	0.00	0.00	0.00	2.33E-01	1.22E-01	1.00E+00	3.20E-01	1.00E+00	1.00E+00	1.00E+00	microcin C transport system ATP-binding protein
KO:K13924	0.32	0.03	0.00	1.22	0.00	0.00	0.00	2.38E-01	4.08E-01	1.00E+00	9.37E-02	1.00E+00	1.00E+00	1.00E+00	two-component system, chemotaxis family, CheB/CheR fusion protein
KO:K13932	0.00	0.00	0.00	0.10	0.00	0.00	0.00	1.00E+00	1.00E+00	1.00E+00	1.43E-01	1.00E+00	1.00E+00	1.00E+00	malonate decarboxylase beta subunit
KO:K13938	0.31	0.07	0.00	0.00	0.00	0.00	0.00	9.33E-02	2.19E-01	1.00E+00	1.00E+00	1.00E+00	1.00E+00	1.00E+00	dihydromonapterin reductase / dihydrofolate reductase
KO:K13950	0.12	0.00	0.00	0.00	0.00	0.00	0.00	6.31E-02	1.00E+00	1.00E+00	1.00E+00	1.00E+00	1.00E+00	1.00E+00	para-aminobenzoate synthetase [EC:2.6.1.85]
KO:K13953	0.01	0.00	0.00	0.09	0.00	0.00	12.44	4.10E-01	1.00E+00	1.00E+00	3.51E-01	1.00E+00	1.00E+00	1.20E-02	alcohol dehydrogenase, propanol-preferring [EC:1.1.1.1]
KO:K13954	0.00	0.00	0.00	0.00	5.00	0.00	0.00	1.00E+00	1.00E+00	1.00E+00	1.00E+00	1.77E-02	1.00E+00	1.00E+00	alcohol dehydrogenase [EC:1.1.1.1]
KO:K13979	0.45	0.00	4.47	0.03	0.00	0.00	0.00	1.98E-01	1.00E+00	1.79E-02	4.80E-01	1.00E+00	1.00E+00	1.00E+00	uncharacterized zinc-type alcohol dehydrogenase-like protein
KO:K13991	0.17	2.00	0.00	1.02	0.00	0.00	0.00	4.55E-01	1.14E-01	1.00E+00	2.07E-01	1.00E+00	1.00E+00	1.00E+00	photosynthetic reaction center H subunit
KO:K13992	0.00	4.41	0.00	0.74	0.00	0.00	0.00	1.00E+00	7.41E-02	1.00E+00	2.51E-01	1.00E+00	1.00E+00	1.00E+00	photosynthetic reaction center cytochrome c subunit
KO:K13993	0.45	0.26	0.78	0.68	0.60	0.35	1.57	5.30E-01	6.63E-01	3.54E-01	4.01E-01	4.40E-01	6.01E-01	1.44E-01	HSP2 family protein
KO:K14028	0.12	0.00	0.00	0.00	0.00	0.00	0.00	7.13E-02	1.00E+00	1.00E+00	1.00E+00	1.00E+00	1.00E+00	1.00E+00	methanol dehydrogenase (cytochrome c) subunit 1 [EC:1.1.2.7]
KO:K14055	0.12	0.00	0.00	0.00	0.00	0.00	0.00	6.53E-02	1.00E+00	1.00E+00	1.00E+00	1.00E+00	1.00E+00	1.00E+00	universal stress protein E
KO:K14058	2.75	0.71	0.00	0.09	0.00	2.36	0.00	7.11E-02	3.16E-01	1.00E+00	6.93E-01	1.00E+00	8.61E-02	1.00E+00	tRNA 2-thiocytidine biosynthesis protein TtcA
KO:K14059	0.12	0.00	0.00	0.00	0.00	0.00	0.00	6.17E-02	1.00E+00	1.00E+00	1.00E+00	1.00E+00	1.00E+00	1.00E+00	integrase
KO:K14067	0.03	0.17	0.00	0.58	0.00	0.00	0.00	2.81E-01	2.25E-01	1.00E+00	1.27E-01	1.00E+00	1.00E+00	1.00E+00	malate-CoA ligase subunit beta [EC:6.2.1.9]
KO:K14083	0.14	4.67	0.00	0.00	0.00	0.00	0.00	3.41E-01	5.36E-02	1.00E+00	1.00E+00	1.00E+00	1.00E+00	1.00E+00	
KO:K14128	0.00	0.00	0.63	0.00	0.00	0.00	0.00	1.00E+00	1.00E+00	1.86E-02	1.00E+00	1.00E+00	1.00E+00	1.00E+00	
KO:K14153	0.12	0.00	0.00	0.10	0.00	0.00	0.00	1.27E-01	1.00E+00	1.00E+00	1.74E-01	1.00E+00	1.00E+00	1.00E+00	
KO:K14155	0.23	2.03	0.22	0.09	0.00	0.00	0.00	3.64E-01	7.96E-02	3.73E-01	4.88E-01	1.00E+00	1.00E+00	1.00E+00	
KO:K14160	0.34	0.00	0.00	0.91	0.00	0.00	0.00	1.87E-01	4.94E-01	1.00E+00	9.64E-02	1.00E+00	1.00E+00	1.00E+00	
KO:K14161	0.02	0.04	0.00	0.02	0.03	8.15	0.00	6.06E-01	5.45E-01	1.00E+00	5.96E-01	5.59E-01	5.71E-04	1.00E+00	
KO:K14162	0.39	0.12	0.62	0.37	0.62	0.00	0.00	2.94E-01	5.24E-01	1.98E-01	3.03E-01	1.98E-01	1.00E+00	1.00E+00	
KO:K14164	0.00	0.00	0.00	1.22	0.00	0.00	0.00	1.00E+00	1.00E+00	1.00E+00	1.43E-01	1.00E+00	1.00E+00	1.00E+00	
KO:K14165	0.00	0.00	0.00	0.00	0.00	0.00	2.92	1.00E+00	1.00E+00	1.00E+00	1.00E+00	1.00E+00	1.00E+00	8.14E-03	
KO:K14166	0.00	0.00	0.00	0.00	0.00	0.00	5.00	1.00E+00	1.00E+00	1.00E+00	1.00E+00	1.00E+00	1.00E+00	5.71E-03	
KO:K14170	1.43	0.00	0.00	0.05	0.09	2.59	8.30	2.13E-01	1.00E+00	1.00E+00	6.56E-01	6.29E-01	1.24E-01	1.93E-02	
KO:K14189	0.00	0.00	0.00	0.20	0.00	0.00	0.00	1.00E+00	1.00E+00	1.00E+00	1.43E-01	1.00E+00	1.00E+00	1.00E+00	
KO:K14260	0.88	0.00	0.00	0.30	0.00	0.00	0.00	9.00E-02	1.00E+00	1.00E+00	2.09E-01	1.00E+00	1.00E+00	1.00E+00	
KO:K14261	0.44	0.00	0.32	0.35	0.66	15.13	1.41	4.77E-01	1.00E+00	5.24E-01	5.12E-01	4.06E-01	1.26E-02	2.52E-01	
KO:K14266	7.73	0.00	0.00	0.06	0.00	0.00	0.00	1.53E-02	1.00E+00	1.00E+00	5.48E-01	1.00E+00	1.00E+00	1.00E+00	
KO:K14267	0.87	0.01	0.00	0.05	0.00	4.94	1.04	1.89E-01	6.92E-01	1.00E+00	6.36E-01	1.00E+00	1.73E-02	1.58E-01	
KO:K14269	0.40	0.00	0.00	0.09	0.00	0.00	0.00	9.24E-02	1.00E+00	1.00E+00	2.30E-01	1.00E+00	1.00E+00	1.00E+00	
KO:K14287	0.97	0.02	0.00	0.16	0.00	0.00	0.00	6.29E-02	4.59E-01	1.00E+00	2.86E-01	1.00E+00	1.00E+00	1.00E+00	
KO:K14330	0.00	0.00	0.00	0.00	21.25	0.00	0.00	1.00E+00	1.00E+00	1.00E+00	1.00E+00	5.71E-04	1.00E+00	1.00E+00	
KO:K14331	0.00	0.00	0.00	0.00	25.63	0.00	0.00	1.00E+00	1.00E+00	1.00E+00	1.00E+00	1.43E-04	1.00E+00	1.00E+00	
KO:K14333	0.00	0.00	0.00	0.82	0.00	0.00	0.00	1.00E+00	1.00E+00	1.00E+00	1.43E-01	1.00E+00	1.00E+00	1.00E+00	
KO:K14392	0.35	0.00	0.00	0.00	0.00	0.00	0.00	6.56E-02	1.00E+00	1.00E+00	1.00E+00	1.00E+00	1.00E+00	1.00E+00	
KO:K14393	3.93	0.15	0.00	2.77	1.46	9.14	0.00	1.55E-01	7.76E-01	1.00E+00	2.38E-01	4.54E-01	4.79E-02	1.00E+00	
KO:K14414	0.23	0.00	0.00	0.00	0.00	0.00	0.00	6.27E-02	1.00E+00	1.00E+00	1.00E+00	1.00E+00	1.00E+00	1.00E+00	
KO:K14415	0.12	0.00	0.00	0.00	0.00	0.00	0.00	6.11E-02	1.00E+00	1.00E+00	1.00E+00	1.00E+00	1.00E+00	1.00E+00	
KO:K14441	0.92	0.00	0.00	0.36	0.00	0.00	0.97	1.31E-01	1.00E+00	1.00E+00	2.75E-01	1.00E+00	1.00E+00	1.19E-01	
KO:K14446	0.00	1.65	0.00	1.10	0.00	0.00	0.00	1.00E+00	8.39E-02	1.00E+00	1.37E-01	1.00E+00	1.00E+00	1.00E+00	
KO:K14447	0.08	1.57	0.00	0.25	0.00	0.00	0.00	4.32E-01	7.31E-02	1.00E+00	3.13E-01	1.00E+00	1.00E+00	1.00E+00	
KO:K14448	0.10	1.45	0.00	0.48	0.00	0.00	0.00	3.63E-01	1.09E-01	1.00E+00	2.30E-01	1.00E+00	1.00E+00	1.00E+00	
KO:K14449	0.13	2.90	0.00	0.05	0.00	0.00	0.00	4.52E-01	3.34E-02	1.00E+00	5.47E-01	1.00E+00	1.00E+00	1.00E+00	
KO:K14450	0.51	0.35	0.00	0.00	0.00	0.00	0.00	1.01E-01	1.88E-01	1.00E+00	1.00E+00	1.00E+00	1.00E+00	1.00E+00	
KO:K14451	0.00	2.11	0.00	0.00	0.00	0.00	0.00	1.00E+00	3.23E-02	1.00E+00	1.00E+00	1.00E+00	1.00E+00	1.00E+00	
KO:K14470	0.00	1.67	0.00	0.00	0.00	0.00	0.00	1.00E+00	1.02E-01	1.00E+00	1.00E+00	1.00E+00	1.00E+00	1.00E+00	
KO:K14472	0.00	0.00	0.00	1.12	0.00	0.00	0.00	1.00E+00	1.00E+00	1.00E+00	1.43E-01	1.00E+00	1.00E+00	1.00E+00	
KO:K14518	0.00	0.00	0.00	0.00	10.63	0.00	0.00	1.00E+00	1.00E+00	1.00E+00	1.00E+00	3.43E-03	1.00E+00	1.00E+00	
KO:K14519	0.03	1.49	6.55	0.38	0.00	0.00	0.00	7.07E-01	1.53E-01	1.69E-02	4.54E-01	1.00E+00	1.00E+00	1.00E+00	
KO:K14520	0.00	0.00	0.00	0.71	0.00	0.00	0.00	1.00E+00	1.00E+00	1.00E+00	1.43E-01	1.00E+00	1.00E+00	1.00E+00	
KO:K14534	0.45	0.00	0.00	0.00	0.00	0.00	3.71	2.10E-01	1.00E+00	1.00E+00	1.00E+00	1.00E+00	1.00E+00	4.97E-02	
KO:K14540	0.06	0.03	0.00	0.05	6.47	0.00	0.00	4.63E-01	5.43E-01	1.00E+00	4.94E-01	5.14E-03	1.00E+00	1.00E+00	
KO:K14541	0.06	0.00	0.00	0.05	0.00	0.00	0.00	1.05E-01	1.00E+00	1.00E+00	2.10E-01	1.00E+00	1.00E+00	1.00E+00	
KO:K14579	0.00	0.00	0.00	1.02	0.00	0.00	0.00	1.00E+00	1.00E+00	1.00E+00	1.43E-01	1.00E+00	1.00E+00	1.00E+00	
KO:K14583	0.00	0.00	0.00	0.61	0.00	0.00	0.00	1.00E+00	1.00E+00	1.00E+00	1.43E-01	1.00E+00	1.00E+00	1.00E+00	
KO:K14584	0.95	0.00	0.00	0.01	0.00	0.00	0.00	6.66E-02	1.00E+00	1.00E+00	2.36E-01	1.00E+00	1.00E+00	1.00E+00	
KO:K14585	0.00	0.00	0.00	0.51	0.00	0.00	0.00	1.00E+00	1.00E+00	1.00E+00	1.43E-01	1.00E+00	1.00E+00	1.00E+00	
KO:K14597	0.00	0.00	0.00	0.00	0.00	0.00	0.42	1.00E+00	1.00E+00	1.00E+00	1.00E+00	1.00E+00	1.00E+00	2.79E-02	
KO:K14647	0.00	0.00	0.00	0.00	0.00	0.00	2.08	1.00E+00	1.00E+00	1.00E+00	1.00E+00	1.00E+00	1.00E+00	2.97E-02	
KO:K14652	3.55	0.00	0.00	1.06	0.00	0.00	0.13	5.64E-02	1.00E+00	1.00E+00	2.25E-01	1.00E+00	1.00E+00	5.19E-01	
KO:K14682	1.72	0.00	0.00	0.39	0.00	0.00	0.04	6.97E-02	1.00E+00	1.00E+00	2.44E-01	1.00E+00	1.00E+00	4.49E-01	
KO:K14728	0.00	0.00	0.00	0.00	0.00	0.00	3.33	1.00E+00	1.00E+00	1.00E+00	1.00E+00	1.00E+00	1.00E+00	2.61E-02	
KO:K14742	0.50	0.00	0.00	0.38	0.00	0.00	0.00	1.27E-01	1.00E+00	1.00E+00	1.54E-01	1.00E+00	1.00E+00	1.00E+00	
KO:K14748	0.00	0.00	0.00	2.04	0.00	0.00	0.00	1.00E+00	1.00E+00	1.00E+00	1.43E-01	1.00E+00	1.00E+00	1.00E+00	
KO:K14749	0.00	0.00	0.00	1.02	0.00	0.00	0.00	1.00E+00	1.00E+00	1.00E+00	1.43E-01	1.00E+00	1.00E+00	1.00E+00	
KO:K14750	0.00	0.00	0.00	0.61	0.00	0.00	0.00	1.00E+00	1.00E+00	1.00E+00	1.43E-01	1.00E+00	1.00E+00	1.00E+00	
KO:K14751	0.00	0.00	0.00	1.02	0.00	0.00	0.00	1.00E+00	1.00E+00	1.00E+00	1.43E-01	1.00E+00	1.00E+00	1.00E+00	
KO:K14941	0.00	0.00	0.00	0.00	0.00	0.00	0.42	1.00E+00	1.00E+00	1.00E+00	1.00E+00	1.00E+00	1.00E+00	2.59E-02	
KO:K14977	0.93	0.00	0.00	0.00	0.00	0.00	0.00	6.29E-02	1.00E+00	1.00E+00	1.00E+00	1.00E+00	1.00E+00	1.00E+00	
KO:K15016	0.00	0.00	0.00	0.00	0.00	0.00	0.83	1.00E+00	1.00E+00	1.00E+00	1.00E+00	1.00E+00	1.00E+00	2.56E-02	
KO:K15034	0.12	0.00	0.00	0.10	0.00	0.00	0.00	1.03E-01	1.00E+00	1.00E+00	2.08E-01	1.00E+00	1.00E+00	1.00E+00	
